# Supplementary material for: Expanding tracer space for positron emission tomography with high molar activity 18F-labeled α,α-difluoromethylalkanes
Source: Nat Commun. 2025 Feb 13;16:1608. doi: 10.1038/s41467-025-56897-5 (PMC11825696; doi:10.1038/s41467-025-56897-5)
Supplement: Supplementary file 1 — Supplementary Information [file 41467_2025_56897_MOESM1_ESM.pdf]

Supplementary Information for

Expanding Tracer Space for Positron Emission Tomography with High Molar Activity ^{18}F-Labeled α,α-Difluoromethylalkanes

Qunchao Zhao[1], Sanjay Telu[1]*, Shuiyu Lu[1], Victor W. Pike[1]*

[1] *Molecular Imaging Branch, National Institute of Mental Health, National Institutes of Health*

10 Center Drive, Bethesda, MD 20892–1003, USA

Corresponding authors: sanjay.telu@nih.gov, pikev@mail.nih.gov

Table of Contents

1. Materials and Methods 4

2. Non-radioactive Experiments 4

2.1. Fluoride leaching experiments 4

2.2. Proposed mechanism for reaction of DBU with 1-fluorononyl triflate[1] 8

2.3. ^{19}F exchange experiments 8

2.4. Synthesis of precursors and standards 11

2.4.1. General procedure 1 for synthesis of α-halo-α-fluoroalkanes[2] 11

2.4.2. General procedure 2 for the synthesis of difluoromethyl compounds 11

2.4.3. General procedure 3 for the synthesis of non-activated alkenes 12

2.4.4. General procedure 4 for the synthesis of α-bromo-α-fluoroalkanes[3] 12

2.4.5. General procedure 5 for the synthesis of difluoromethyl compounds[4] 12

2.4.6. General procedure 6 for the synthesis of difluoromethyl compounds 13

2.4.7. General procedure 7 for the synthesis of difluoromethyl compounds[5] 13

2.4.8. Specific syntheses 13

3. Radiochemistry procedures 53

3.1. General HPLC methods 53

3.1.1. HPLC condition A 53

3.1.2. HPLC condition B 53

3.1.3. HPLC condition C 53

3.1.4. HPLC condition D 54

3.2. General procedures for radiofluorination 54

3.2.1. Preparation of anhydrous [^{18}F]fluoride/Et_4NHCO_3 solution 54

3.2.2. Radiofluorination of different precursors at set temperatures 54

3.2.3. General procedure 8 for manual substrates scope evaluation 55

3.2.4. General procedure 9 for automated radiosynthesis 55

3.3. Molar activity determination 56

3.3.1. Basic principle of molar activity determination 57

3.3.2. Optimization for molar activity measurement 57

3.3.3. Automated radiosyntheses 59

3.4. Mechanism study 75
3.4.1. Control experiment 75
3.4.2. Investigation of the potential formation of the vinyl bromide **10c** in the radiofluorination of **10a** 76
3.4.3. Proposed mechanism for the formation of α,α-difluoromethylalkane [^{18}F]**10** 77
4. HPLC analyses of radiolabeled compounds [^{18}F]1–[^{18}F]47 78
5. NMR spectra of precursors and standards 1–47 167
6. References 319

1. Materials and Methods

Commercially available chemicals, reagents, and solvents were purchased from Sigma-Aldrich, Alfa Aesar, TCI, AmBeed, or Chemscene, and used as received. ^{1}H- (400.13 MHz), ^{13}C- (100.62 MHz), and ^{19}F- (376.47 MHz) NMR spectra were recorded on an Avance 400 instrument (Bruker) at RT in deuterated solvents. All spectra are reported with chemical shifts in parts per million. NMR data are reported as follows: chemical shift (δ (ppm), multiplicity (s = singlet, d = doublet, t = triplet, q = quartet, quint = quintet, sept = septet, dd = doublet of doublets, dt = doublet of triplets, dq = doublet of quartets, qd = quartet of doublets, td = triplet of doublets, tt = triplet of triplets, ddd = doublet of doublet of doublets, dtd = doublet of triplet of doublets, tdd = triplet of doublet of doublets, m = multiplet, app = apparent), coupling constants (J in Hz), and integration. TMS (δ = 0 ppm) was used as an internal standard for ^{1}H and ^{13}C-NMR spectroscopy. NMR data were processed using MestReNova. HRMS data (ESI-TOF) were obtained at the Bioorganic Chemistry Laboratory of NIDDK (NIH). Melting points were measured with a digital SMP20 (Stuart) melting point apparatus. Radiochemistry was performed in lead-shielded hot-cells for protection of personnel from radiation. HPLC analyses were performed on Shimadzu HPLC systems, which were equipped with UV absorbance and radioactivity detectors. Semi-preparative HPLC purifications were performed on Knauer HPLC system, also equipped with UV absorbance and radioactivity detectors. HPLC chromatograms were processed using GraphPad Prism 8. Flash chromatography was performed on silica gel; eluent compositions are reported as *v*/*v*.

2. Non-radioactive Experiments

2.1. Fluoride leaching experiments

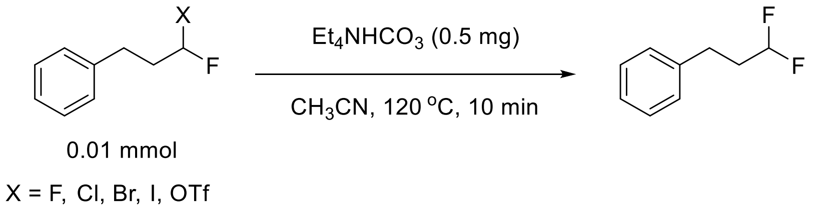

Precursor α-substituted fluoroalkane (0.01 mmol) and MeCN (0.5 mL) were added to a 1-mL glass vial equipped with a magnetic stirrer bar followed by Et_4NHCO_3 (0.5 mg). The vial was capped loosely and then the contents were stirred at 120 °C for 10 min. The reaction was then quenched with water (0.2 mL). The yield of (3,3-difluoropropyl)benzene (δ –116.4 ppm) was determined by ^{19}F-NMR analysis of the crude reaction mixture with $PhCF_3$ (δ –62.0 ppm) as internal standard. The ^{19}F-NMR spectra for the crude reaction mixtures are shown below.

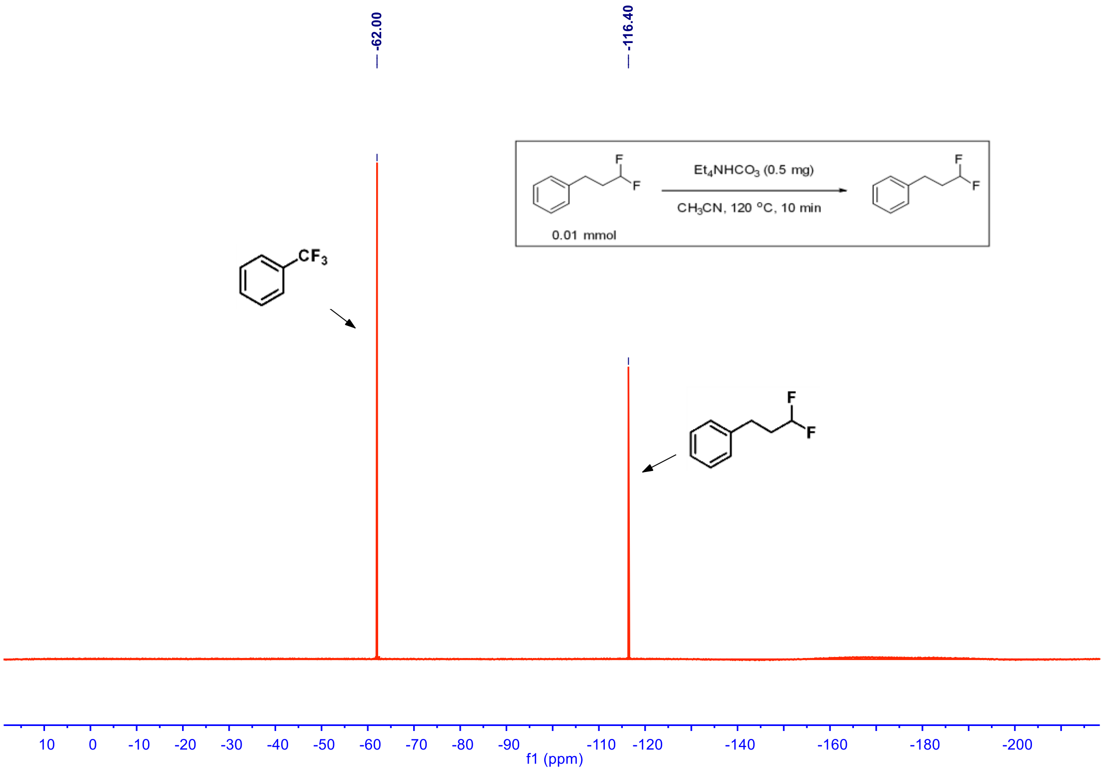

Supplementary Figure 1. Fluoride leaching experiment with **1** (X = F).

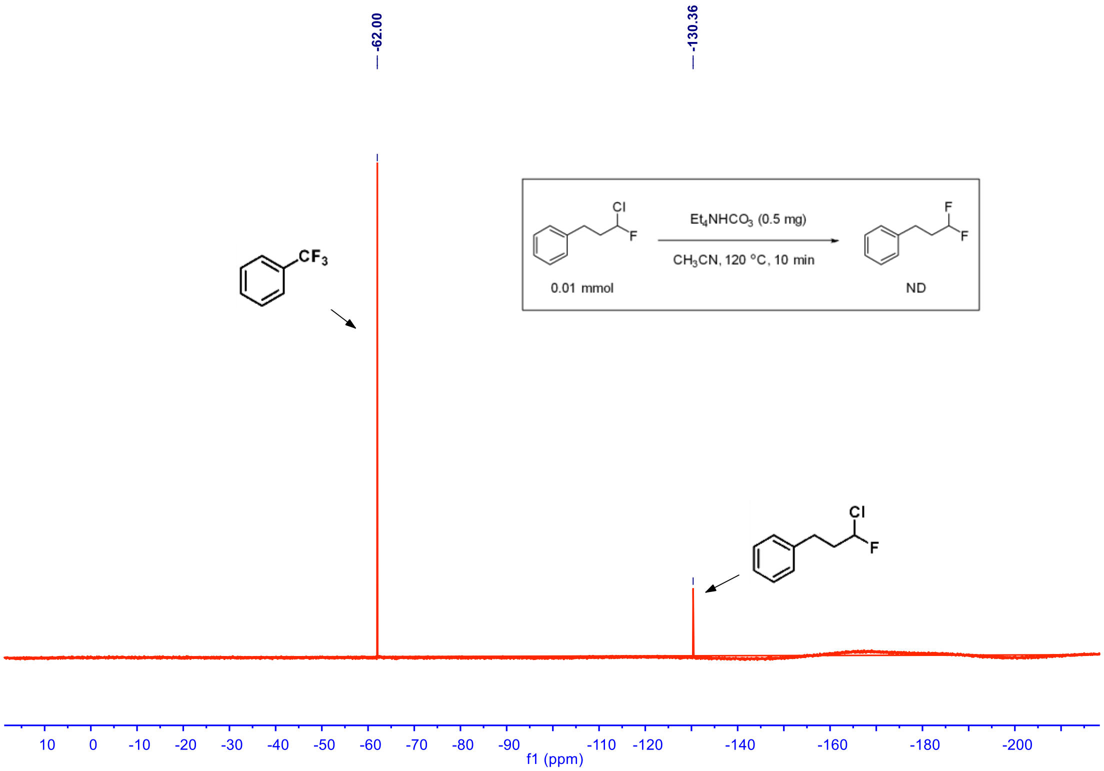

Supplementary Figure 2. Fluoride leaching experiment with **2** (X = Cl). ND = not detected.

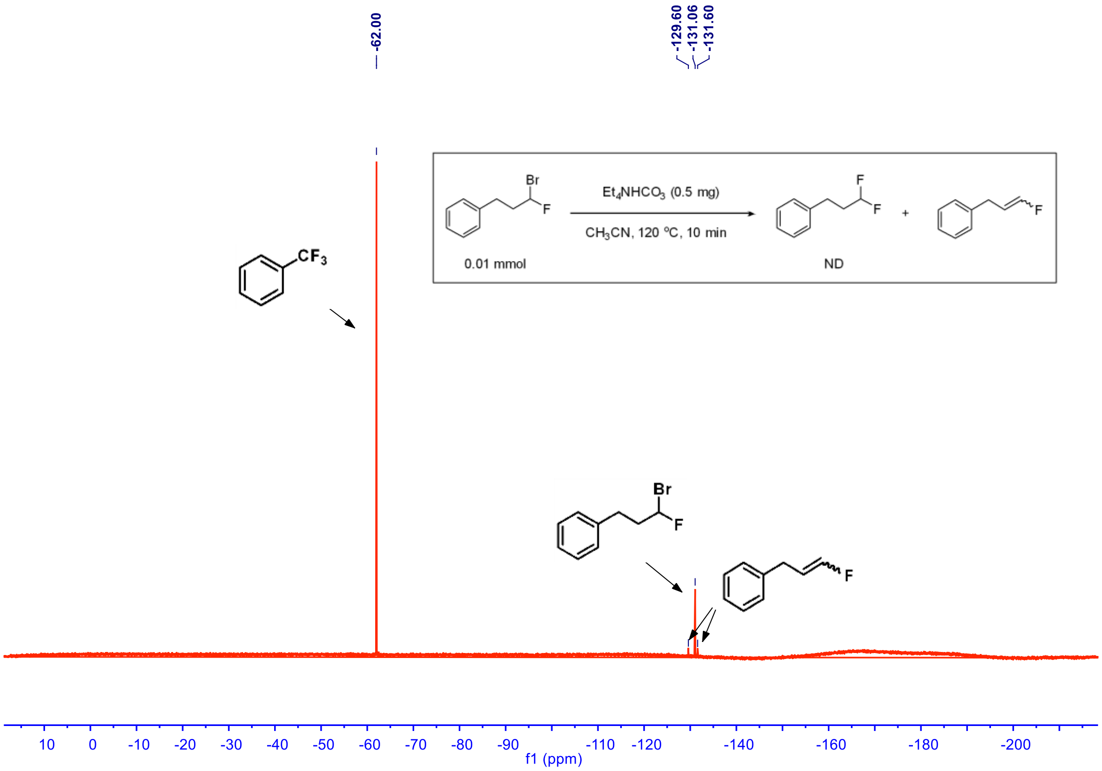

Supplementary Figure 3. Fluoride leaching experiment with **3** (X = Br). ND = not detected.

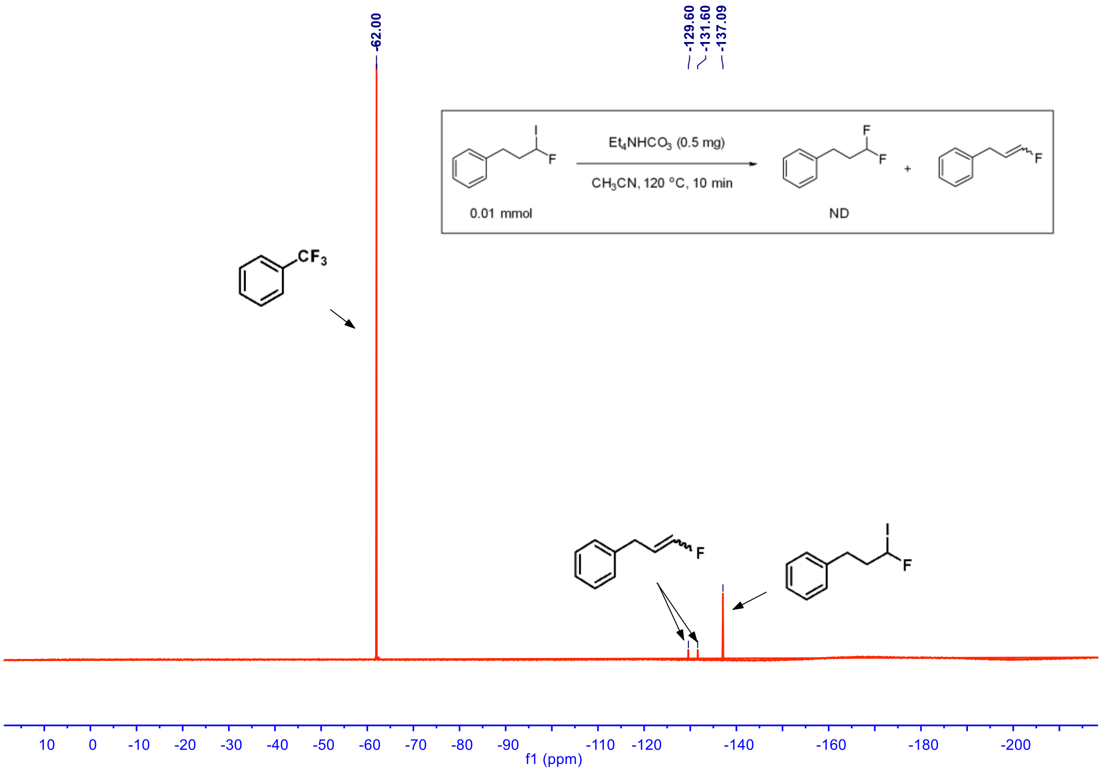

Supplementary Figure 4. Fluoride leaching experiment with **4** (X = I). ND = not detected.

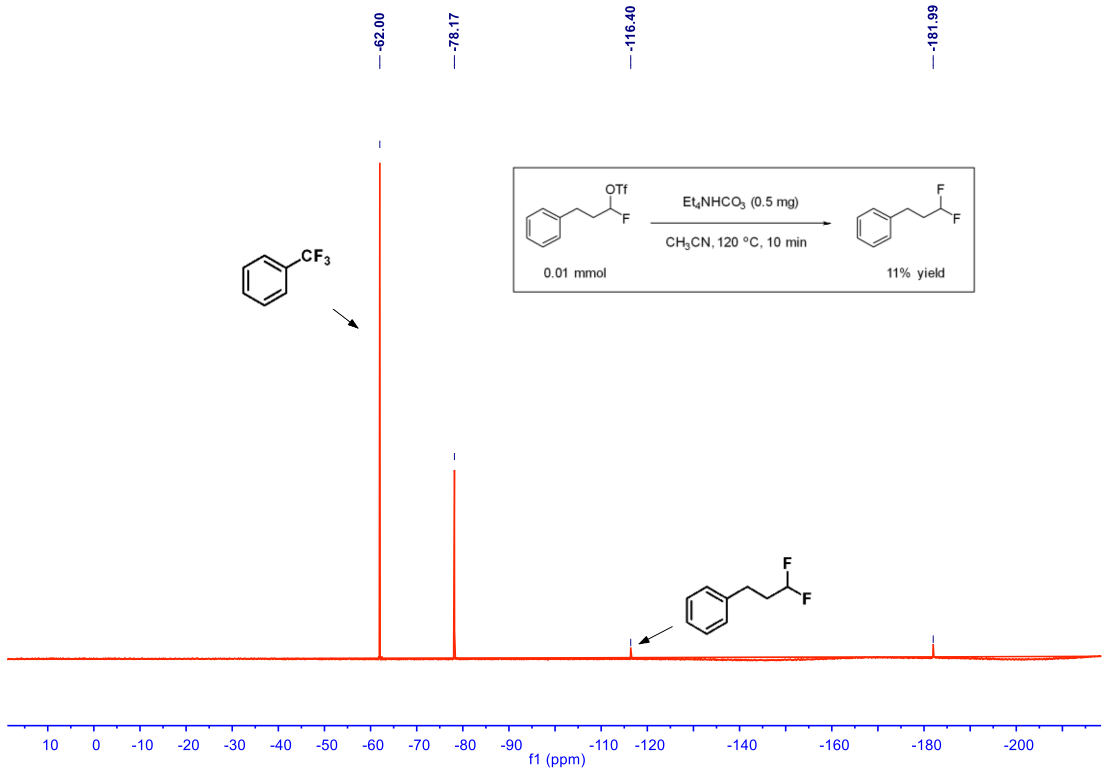

Supplementary Figure 5. Fluoride leaching experiment with **5** (X = OTf).

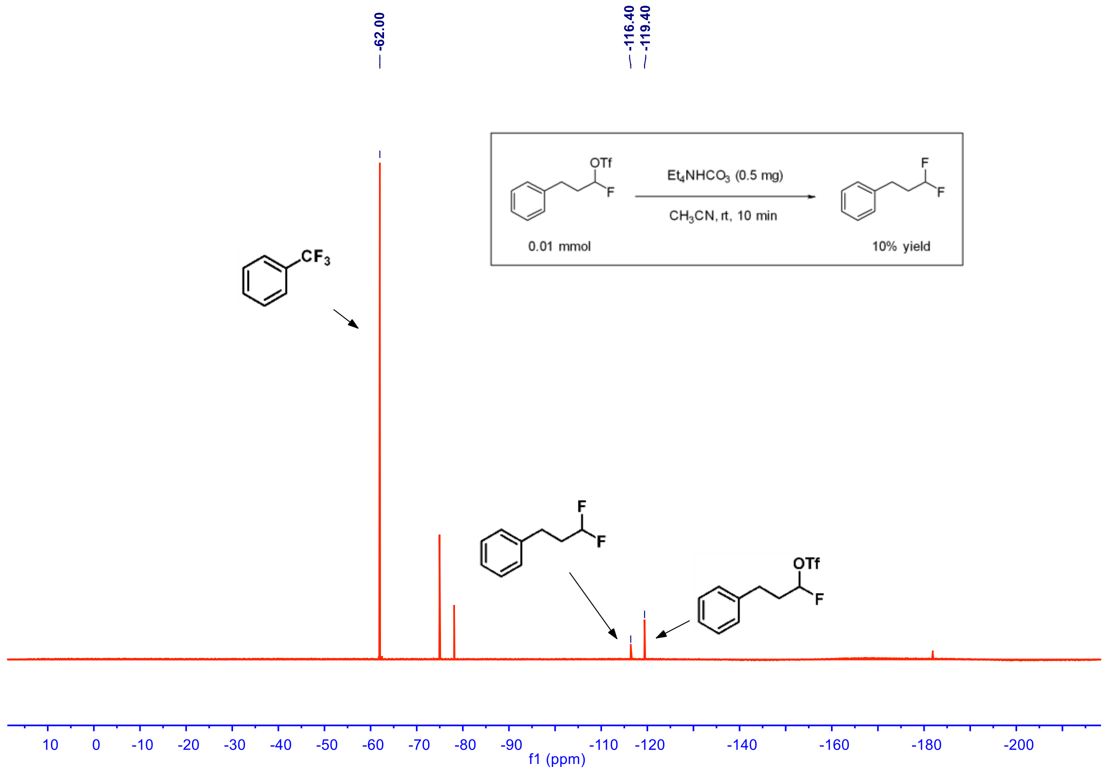

Supplementary Figure 6. Fluoride leaching experiment with **5** (X = OTf) at room temperature.

2.2. Proposed mechanism for reaction of DBU with 1-fluorononyl triflate[1]

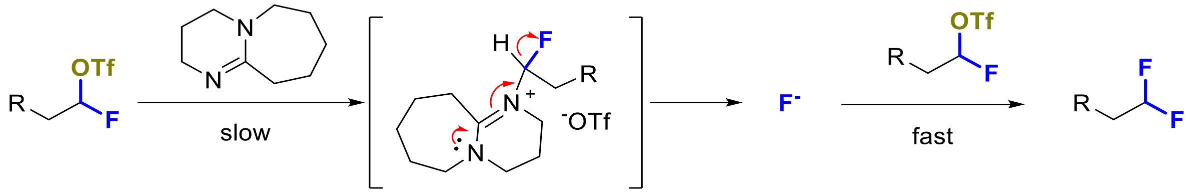

Supplementary Figure 7. Proposed mechanism for the reaction of DBU with 1-fluorononyl triflate.

2.3. ^{19}F exchange experiments

Procedure:

Precursor (3-bromo-3-fluoropropyl)benzene (**3**, 0.05 mmol), tetramethylammonium fluoride (x mmol) and MeCN (0.5 mL) were added to a 1-mL glass vial equipped with a magnetic stirrer bar. The vial was capped loosely and then the contents were stirred at 120 °C for 10 min. The reaction was then quenched with water (0.2 mL). The yields of (3,3-difluoropropyl)benzene (**1**, *δ* –116.4 ppm) and (*E*/*Z*)-(3-fluoroallyl)benzene (**1b**, *δ* –129.6 and –131.6 ppm) were determined based on the amounts of tetramethylammonium fluoride by ^{19}F-NMR analysis of the crude reaction mixture with $PhCF_3$ (*δ* –62.0 ppm) as internal standard. The results and ^{19}F-NMR spectra are shown below.

Results:

Supplementary Table 1. ^{19}F exchange experiments examining the effect of precursor **3** to Me_4NF ratio on the formation of substitution product **1** and elimination product **1b**.

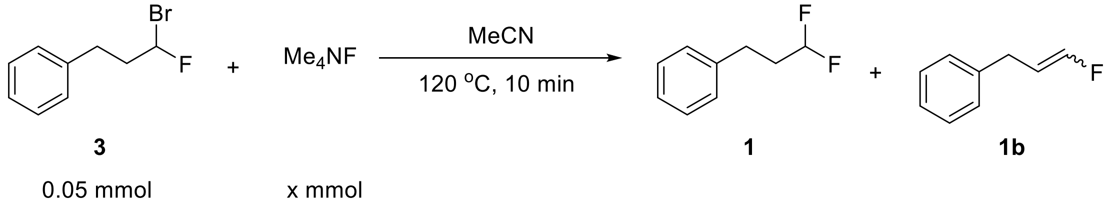

Entry	x	Ratio (**3** / Me_4NF)	Yield of **1** (%)	Yield of **1b** (%)
1	0.05	1 / 1	9	29
2	0.025	2 / 1	11	26
3	0.005	10 / 1	9	31
4	0	-	0	0

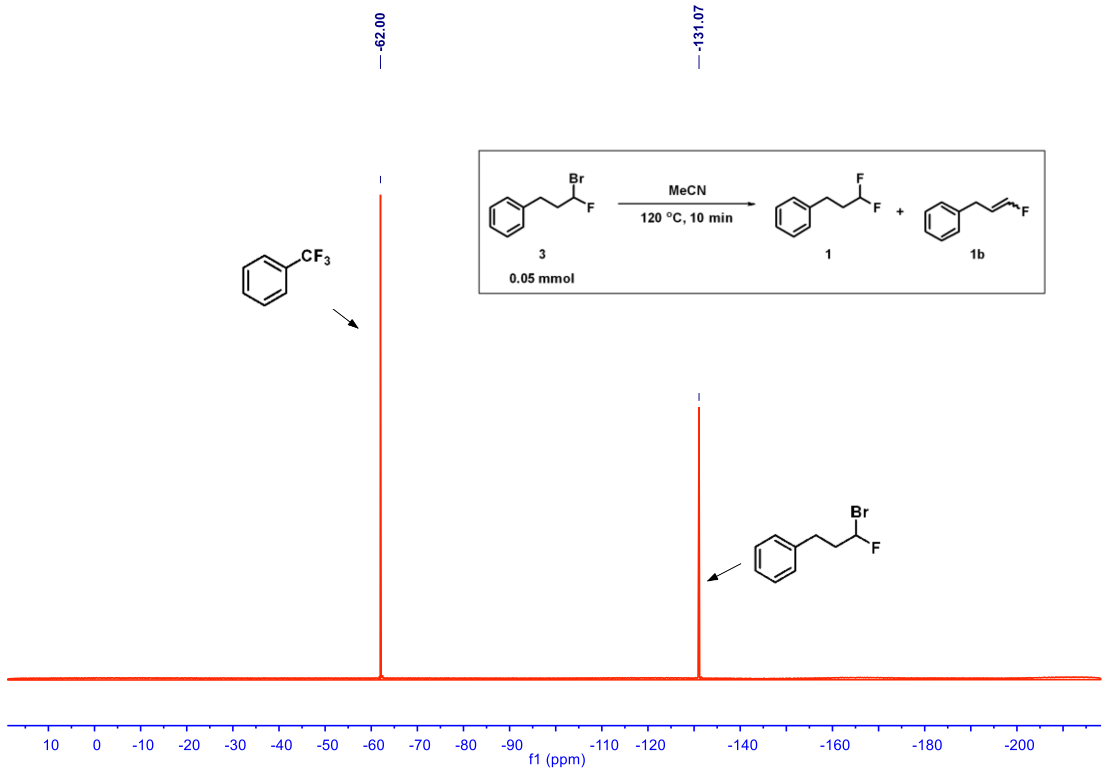

Supplementary Figure 8. ^{19}F exchange experiment of **3** in the absence of Me_4NF.

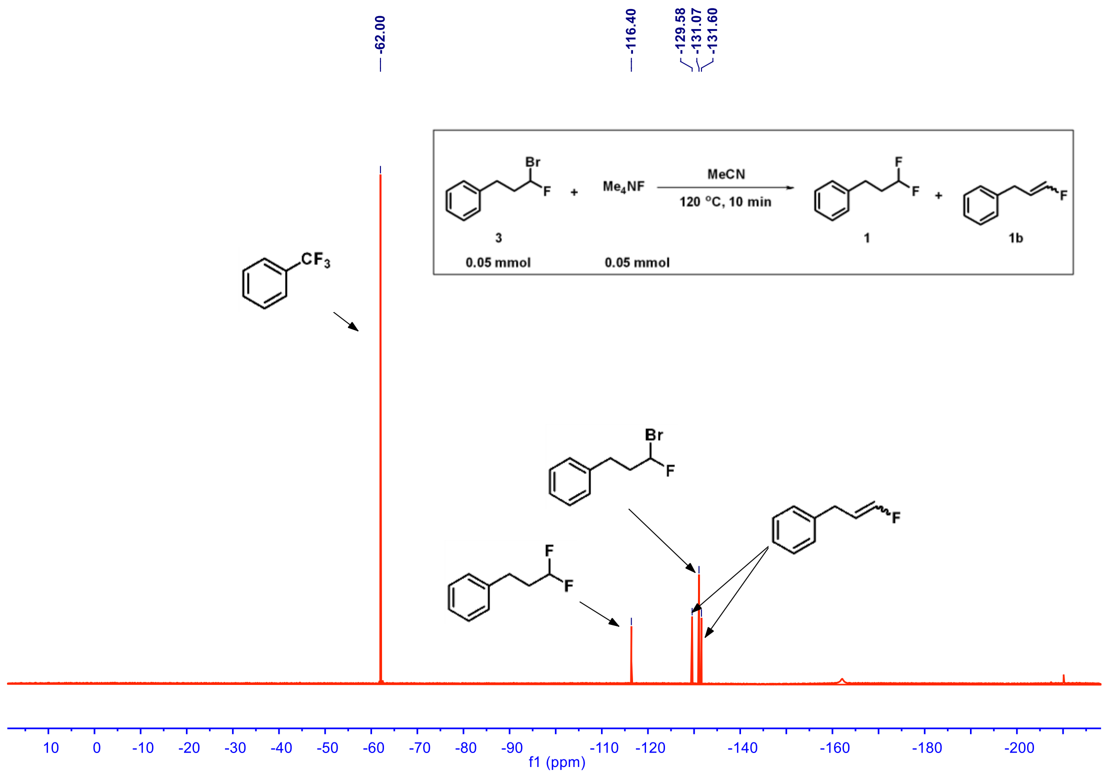

Supplementary Figure 9. ^{19}F exchange experiment of **3** with Me_4NF (0.05 mmol, 1.0 equiv.).

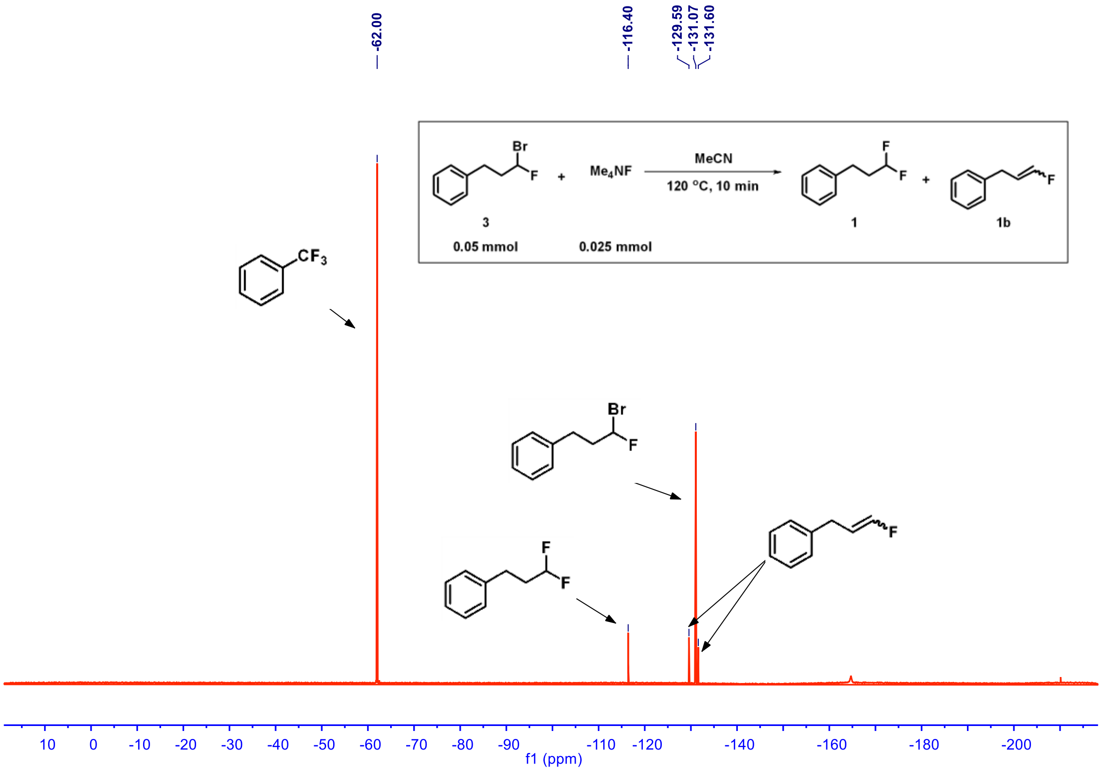

Supplementary Figure 10. ^{19}F exchange experiment of **3** with Me_4NF (0.025 mmol, 0.5 equiv.).

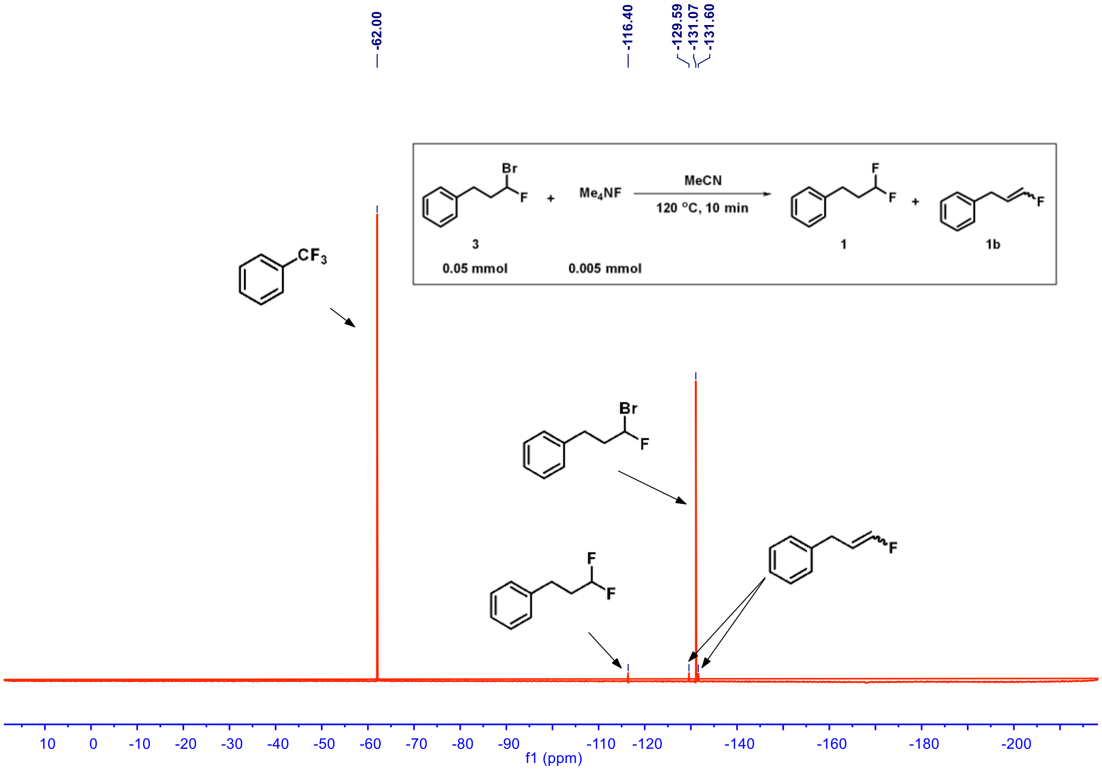

Supplementary Figure 11. ^{19}F exchange experiment of **3** with Me_4NF (0.005 mmol, 0.1 equiv.).

2.4. Synthesis of precursors and standards

2.4.1. General procedure 1 for synthesis of α-halo-α-fluoroalkanes[2]

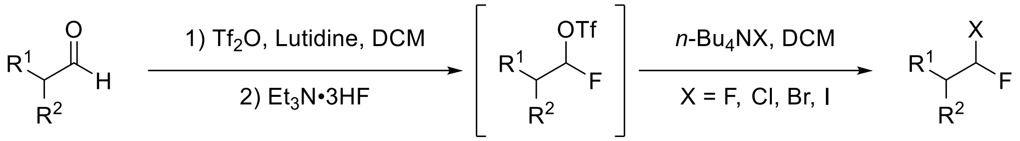

Triflic anhydride (21 mmol, 2.1 equiv.) in DCM (20 mL) was added to a round-bottomed flask containing the precursor aldehyde (10 mmol, 1 equiv.) and 2,6-lutidine (17 mmol, 1.7 equiv.) in DCM (100 mL) at –30 °C. The solution was stirring under N_2 for 1 h at –30 °C and then placed in a refrigerator for 2 days. Then, Et_3N•3HF (10 mmol, 1 equiv.) in DCM (20 mL) was added dropwise to the mixture of bis-triflate at –30 °C. The mixture was allowed to reach RT and stirred overnight. The reaction was slowly quenched by adding sat aq. $NaHCO_3$ (50 mL) at 0 °C and the organic layer was washed with 1 M aq. HCl (50 mL) and sat. aq. $NaHCO_3$ (50 mL). The organic phase was dried ($MgSO_4$), filtered, and concentrated. The product 1-fluoroalkyl triflate was used for the next step without purification.

n-Bu_4NX (4.0 mmol, 2.0 equiv., X = F, Cl, Br, or I) in DCM (2.0 mL) was added to a closed reaction vessel containing 1-fluoroalkyl triflate (2.0 mmol, 1.0 equiv.) in DCM (2.0 mL) and stirred under argon for 2 h at RT. 1 M aq. HCl (5 mL) was then added. The lower layer was washed with sat. aq. $NaHCO_3$, dried ($MgSO_4$) and concentrated in vacuo. Silica gel chromatography on the residue gave the final product.

2.4.2. General procedure 2 for the synthesis of difluoromethyl compounds

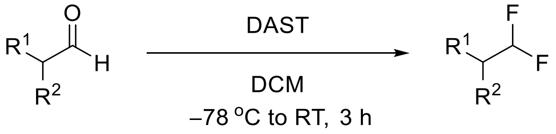

Diethylaminosulfur trifluoride (DAST; 4.0 mmol, 0.53 mL, 2 equiv.) was added to a solution of the aldehyde (2.0 mmol, 1.0 equiv.) in anhydrous DCM (5.0 mL) at –78 °C. The mixture was stirred at –78 °C for 30 min and then at RT for 2 h. Then, the mixture was poured onto sat. aq Na_2CO_3 solution. The layers were separated, and the aqueous layer was extracted three times with DCM. The combined organic phases were dried ($MgSO_4$), filtered, and evaporated. Flash chromatography of the crude mixture gave the pure difluoromethyl compound.

2.4.3. General procedure 3 for the synthesis of non-activated alkenes

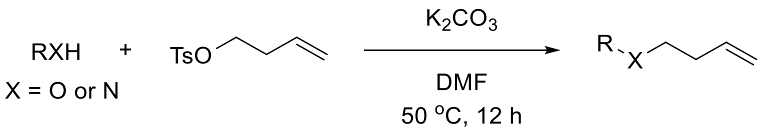

The substrate (RXH; 5.0 mmol, 1 equiv.) was added to K_2CO_3 (1.38 g, 10 mmol, 2.0 equiv.), DMF (30 mL), and but-3-en-1-yl 4-methylbenzenesulfonate (1.36 g, 6.0 mmol, 1.2 equiv.) in a dried round-bottomed flask equipped with a stirrer bar. The reaction mixture was stirred for 12 h at 50 °C and then water (50 mL) and DCM (50 mL) were added. The organic phase was washed with brine, dried (Mg_2SO_4), and concentrated in vacuo. Silica gel chromatography of the residue gave the title compound.

2.4.4. General procedure 4 for the synthesis of α-bromo-α-fluoroalkanes[3]

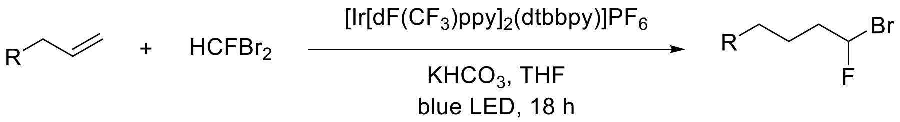

$[Ir[dF(CF_3)ppy]_2(dtbbpy)]PF_6$ (4.5 mg, 0.004 mmol) and $KHCO_3$ (160 mg, 1.6 mmol) were loaded into a 20-mL glass vial equipped with a magnetic stirrer bar, The vial was sealed and then evacuated and refilled with nitrogen for three times. Anhydrous THF (2.0 mL), alkene (0.4 mmol, 1.0 equiv), and $CHFBr_2$ (160 μL, 2.0 mmol) were added to the vial, which was then placed 5 cm away from a blue LED (450 nm). The mixture was then stirred under nitrogen while irradiated by blue LED for 18 h. The reaction mixture was then poured into water and extracted with EtOAc. The combined organic phase was separated, washed with brine, dried (Mg_2SO_4), and concentrated under vacuum. Silica gel flash column chromatography of the residue gave the title compound.

2.4.5. General procedure 5 for the synthesis of difluoromethyl compounds[4]

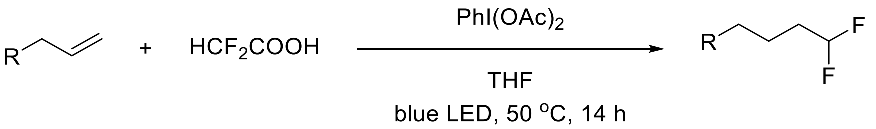

Difluoroacetic acid (113 uL, 1.80 mmol, 6.0 equiv) was added to a 20-mL glass vial equipped with a magnetic stirrer bar and containing a suspension of (diacetoxyiodo)benzene (290 mg, 0.90 mmol, 3.0 equiv) and alkene (0.30 mmol) in THF (4.5 mL). The vial was stirred while irradiated with a blue LED lamp at 50 °C for 6 h after which a second portion of (diacetoxyiodo)benzene (290 mg,

1.80 mmol, 3.0 equiv) was added. The solution was stirred under blue LED irradiation for another 8 h. The solvents were removed in vacuo. Column chromatography of the residue gave the title compound.

2.4.6. General procedure 6 for the synthesis of difluoromethyl compounds

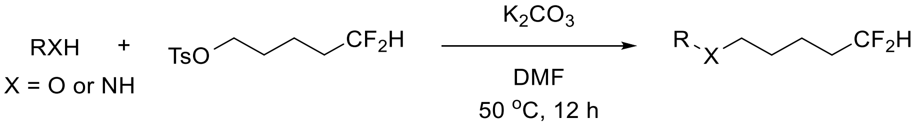

Precursor (RXH; 0.9 mmol, 3.0 equiv.), K_2CO_3 (124 mg, 0.9 mmol, 3.0 equiv.), DMF (3.0 mL) and 5,5-difluoropentyl 4-methylbenzenesulfonate (84 mg, 0.3 mmol, 1.0 equiv.) were added under argon to a 20-mL reaction vessel. The reaction vessel was capped loosely and stirred vigorously at 50 °C for 12 h. The mixture was cooled to RT, poured onto water, and extracted with DCM (2 × 10 mL). The organic phase was dried (Mg_2SO_4) and concentrated in vacuo. Silica gel flash chromatography of the residue gave the title compound.

2.4.7. General procedure 7 for the synthesis of difluoromethyl compounds[5]

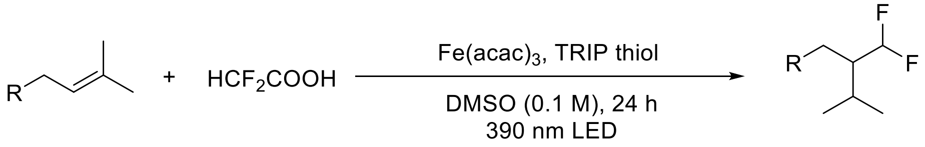

An oven-dried 20-mL glass vial was charged with a stirrer bar and $Fe(acac)_3$ (14 mg, 0.04 mmol, 0.1 equiv.). Anhydrous DMSO (4.0 mL, 0.1 M) was then added followed by alkene (0.4 mmol, 1.0 equiv.), TRIP thiol (2,4,6-triisopropylbenzenethiol; 19 mg, 0.08 mmol, 0.2 equiv.), and difluoroacetic acid (192 mg, 2.0 mmol, 5.0 equiv.) under argon. The reaction mixture was stirred and irradiated using a 390 nm LED lamp for 24 h. Then, the reaction was quenched with sat. aq. $NaHCO_3$ (5 mL) and extracted with EtOAc (2 × 10 mL). The organic phase was dried (Mg_2SO_4) and concentrated in vacuo. Silica gel flash chromatography of the residue gave the title compound.

2.4.8. Specific syntheses

(3,3-Difluoropropyl)benzene (1). General procedure 2 with 3-phenylpropanal followed by flash chromatography (*n*-hexane) gave **1** as a colorless oil (221 mg, 71% yield).

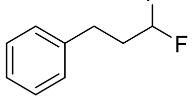

^{1}H NMR (400 MHz, $CDCl_3$) δ (ppm): 7.30 (t, J = 7.2 Hz, 2H), 7.21 (dd, J = 12.8, 7.0 Hz, 3H), 5.79 (tt, J = 56.7, 4.3 Hz, 1H), 2.90–2.58 (m, 2H), 2.41–1.95 (m, 2H); ^{19}F NMR (376 MHz, $CDCl_3$) δ (ppm): –117.11 (dt, J = 34.2, 16.7 Hz, 2F); ^{13}C NMR (101 MHz, $CDCl_3$) δ (ppm): 140.12, 128.86, 128.52, 126.61, 116.91 (t, J = 238.9 Hz), 35.88 (t, J = 21.0 Hz), 28.61 (t, J = 5.9 Hz). [Known compound [6]].

(3-Chloro-3-fluoropropyl)benzene (2). General procedure 1 with 3-phenylpropanal followed by flash chromatography (*n*-hexane) gave **2** as a colorless oil (202 mg, 58% yield).

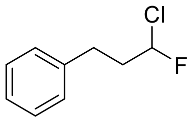

^{1}H NMR (400 MHz, $CDCl_3$) δ (ppm): 7.29 (t, J = 7.4 Hz, 2H), 7.22–7.16 (m, 3H), 6.08 (dt, J = 50.8, 5.4 Hz, 1H), 2.80 (t, J = 7.5 Hz, 2H), 2.58–2.07 (m, 2H); ^{19}F NMR (376 MHz, $CDCl_3$) δ (ppm):–122.49 to –139.50 (m, 1F); ^{13}C NMR (101 MHz, $CDCl_3$) δ (ppm): 139.79, 128.90, 128.63, 126.69, 102.27 (d, J = 241.5 Hz), 40.97 (d, J = 20.2 Hz), 30.53 (d, J = 5.0 Hz). MS (EI) *m*/*z*: 172.0 [*M*]. HRMS (EI) *m*/*z*: for $C_9H_{10}FCl$ $[M]^+$: calc'd: 172.0455; found: 172.0461.

(3-Bromo-3-fluoropropyl)benzene (3). General procedure 1 with 3-phenylpropanal followed by flash chromatography (*n*-hexane) gave **3** as a colorless oil (264 mg, 61% yield).

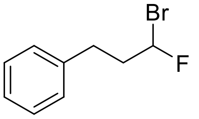

^{1}H NMR (400 MHz, $CDCl_3$) δ (ppm): 7.30 (t, J = 7.2 Hz, 2H), 7.20 (dd, J = 15.1, 7.5 Hz, 3H), 6.38 (dt, J = 50.3, 5.3 Hz, 1H), 2.82 (t, J = 7.7 Hz, 2H), 2.72–2.25 (m, 2H); ^{19}F NMR (376 MHz, $CDCl_3$) δ (ppm): –132.32 (ddd, J = 50.1, 20.3, 16.2 Hz, 1F); ^{13}C NMR (101 MHz, $CDCl_3$) δ (ppm): 139.59, 128.89, 128.63, 126.69, 94.91 (d, J = 252.2 Hz), 42.26 (d, J = 19.2 Hz), 31.41 (d, J = 4.4 Hz). [Known compound [2]].

(3-Fluoro-3-iodopropyl)benzene (4). General procedure 1 with 3-phenylpropanal followed by flash chromatography (*n*-hexane) gave **4** as a colorless oil (256 mg, 48% yield).

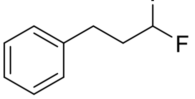

^{1}H NMR (400 MHz, $CDCl_3$) δ (ppm): 7.30 (t, J = 7.4 Hz, 2H), 7.20 (dd, J = 16.1, 7.3 Hz, 3H), 6.89–6.54 (m, 1H), 2.87–2.70 (m, 2H), 2.70–2.32 (m, 2H); ^{19}F NMR (376 MHz, $CDCl_3$) δ (ppm): –138.12 (ddd, J = 50.3, 21.8, 17.0 Hz, 1F); ^{13}C NMR (101 MHz, $CDCl_3$) δ (ppm): 139.46, 128.91, 128.69, 126.72, 74.47 (d, J = 253.9 Hz), 44.93 (d, J = 19.0 Hz), 32.99 (d, J = 3.8 Hz). [Known compound [2]].

1-Fluoro-3-phenylpropyl trifluoromethanesulfonate (5). General procedure 1 with 3-phenylpropanal followed by flash chromatography (*n*-hexane) gave **5** as a colorless oil (2.15 g, 75% yield).

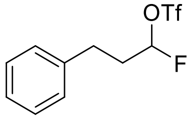

^{1}H NMR (400 MHz, $CDCl_3$) δ (ppm): 7.32 (t, J = 7.4 Hz, 2H), 7.24 (t, J = 7.6 Hz, 1H), 7.18 (d, J = 7.3 Hz, 2H), 6.11 (dt, J = 54.3, 5.1 Hz, 1H), 2.80 (t, J = 7.9 Hz, 2H), 2.51–2.10 (m, 2H); ^{19}F NMR (376 MHz, $CDCl_3$) δ (ppm): –74.96 (d, J = 8.1 Hz, 3F), –113.21 to –126.81 (m, 1F); ^{13}C NMR (101 MHz, $CDCl_3$) δ (ppm): 138.85, 129.08, 128.46, 127.03, 118.51 (q, J = 318.9 Hz), 111.99 (d, J = 245.8 Hz), 35.43 (d, J = 20.3 Hz), 28.78 (d, J = 5.3 Hz). [Known compound[2]].

1-(3-Bromo-3-fluoropropyl)-4-(*tert*-butyl)benzene (6a). General procedure 1 with 3-(4-(*tert*-butyl)phenyl)propanal followed by flash chromatography (*n*-hexane) gave **6a** as a colorless oil (187 mg, 34% yield).

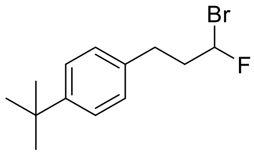

^{1}H NMR (400 MHz, $CDCl_3$) δ (ppm): 7.33 (d, J = 8.2 Hz, 2H), 7.13 (d, J = 8.1 Hz, 2H), 6.39 (dt, J = 50.3, 5.5 Hz, 1H), 2.80 (t, J = 7.7 Hz, 2H), 2.70–2.22 (m, 2H), 1.31 (s, 9H); ^{19}F NMR (376 MHz, $CDCl_3$) δ (ppm): –132.26 (ddd, J = 50.2, 20.4, 15.6 Hz, 1F); ^{13}C NMR (101 MHz, $CDCl_3$) δ (ppm): 149.58, 136.48, 128.31, 125.78, 95.07 (d, J = 252.2 Hz), 42.29 (d, J = 19.1 Hz), 34.63, 31.58, 30.87 (d, J = 4.5 Hz). MS (EI) m/z: 272.0 $[M]^+$. HRMS (EI) m/z for $C_{13}H_{18}FBr$ $[M]^+$: calc'd: 272.0576; found: 272.0571.

1-(*tert*-Butyl)-4-(3,3-difluoropropyl)benzene (6). General procedure 2 with 3-(4-(*tert*-butyl)phenyl)propanal followed by flash chromatography (*n*-hexane) gave **6** as a colorless oil (268 mg, 63% yield).

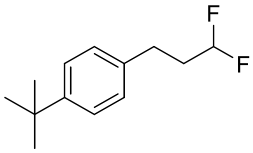

^{1}H NMR (400 MHz, $CDCl_3$) δ (ppm): 7.32 (d, J = 7.9 Hz, 2H), 7.12 (d, J = 7.9 Hz, 2H), 5.78 (tt, J = 56.8, 4.4 Hz, 1H), 2.97–2.52 (m, 2H), 2.41–1.95 (m, 2H), 1.31 (s, 9H); ^{19}F NMR (376 MHz, $CDCl_3$) δ (ppm): –117.01 (dt, J = 34.2, 16.6 Hz, 2F); ^{13}C NMR (101 MHz, $CDCl_3$) δ (ppm): 149.48, 137.03, 128.20, 125.75, 117.01 (t, J = 238.9 Hz), 35.87 (t, J = 21.0 Hz), 34.61, 31.58, 28.06 (t, J = 6.0 Hz). [Known compound[7]].

1-(3-Bromo-3-fluoropropyl)-4-methoxybenzene (7a). General procedure 1 with 3-(4-methoxyphenyl)propanal followed by flash chromatography (ethyl acetate/*n*-hexane, 1: 20) gave **7a** as a colorless oil (125 mg, 25% yield).

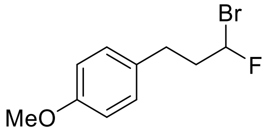

^{1}H NMR (400 MHz, $CDCl_3$) δ (ppm): 7.14 (d, J = 7.3 Hz, 2H), 6.87 (d, J = 7.1 Hz, 2H), 6.41 (dt, J = 50.3, 5.2 Hz, 1H), 3.81 (s, 3H), 2.80 (t, J = 7.6 Hz, 2H), 2.67–2.28 (m, 2H); ^{19}F NMR (376 MHz, $CDCl_3$) δ (ppm): –132.31 (ddd, J = 50.4, 20.4, 16.2 Hz, 1F); ^{13}C NMR (101 MHz, $CDCl_3$) δ (ppm): 158.43, 131.53, 129.55, 114.27, 95.01 (d, J = 252.2 Hz), 55.44, 42.49 (d, J = 19.0 Hz), 30.52 (d, J = 4.5 Hz). MS (EI) m/z: 246.0 $[M]^+$. HRMS (EI) m/z for $C_{10}H_{12}FOBr$ $[M]^+$: calc'd: 246.0056; found: 246.0046.

1-(3,3-Difluoropropyl)-4-methoxybenzene (7). General procedure 2 with 3-(4-methoxyphenyl)propanal followed by flash chromatography (ethyl acetate/*n*-hexane, 1: 20) gave **7** as a colorless oil (192 mg, 52% yield).

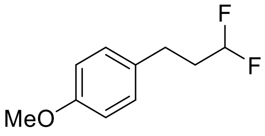

^{1}H NMR (400 MHz, $CDCl_3$) δ (ppm): 7.14 (d, J = 7.7 Hz, 2H), 6.88 (d, J = 7.5 Hz, 2H), 5.81 (tt, J = 56.7, 4.3 Hz, 1H), 3.82 (s, 3H), 2.97–2.57 (m, 2H), 2.34–1.94 (m, 2H); ^{19}F NMR (376 MHz, $CDCl_3$) δ (ppm): –117.08 (dt, J = 56.3, 16.9 Hz, 2F); ^{13}C NMR (101 MHz, $CDCl_3$) δ (ppm): 158.41, 132.12, 129.46, 117.01 (t, J = 238.9 Hz), 114.26, 55.43, 36.12 (t, J = 20.9 Hz), 27.75 (t, J = 6.0 Hz). [Known compound[8]].

1-(3-Bromo-3-fluoropropyl)-4-chlorobenzene (8a). General procedure 1 with 3-(4-chlorophenyl)propanal followed by flash chromatography (*n*-hexane) gave **8a** as a colorless oil (200 mg, 40% yield).

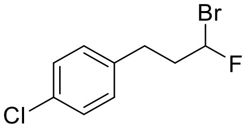

^{1}H NMR (400 MHz, $CDCl_3$) δ (ppm): 7.29 (d, J = 8.2 Hz, 2H), 7.14 (d, J = 8.1 Hz, 2H), 6.41 (dt, J = 50.2, 5.4 Hz, 1H), 2.82 (t, J = 7.7 Hz, 2H), 2.70–2.28 (m, 2H); ^{19}F NMR (376 MHz, $CDCl_3$) δ (ppm): –132.63 (ddd, J = 50.2, 20.1, 16.7 Hz, 1F); ^{13}C NMR (101 MHz, $CDCl_3$) δ (ppm): 138.03, 132.49, 129.97, 128.99, 94.55 (d, J = 252.3 Hz), 42.04 (d, J = 19.5 Hz), 30.73 (d, J = 4.4 Hz). [Known compound[9]].

Chloro-4-(3,3-difluoropropyl)benzene (8). General procedure 2 with 3-(4-chlorophenyl)propanal followed by flash chromatography (*n*-hexane) gave **8** as a colorless oil (171 mg, 45% yield).

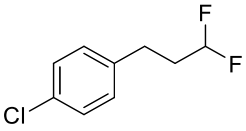

^{1}H NMR (400 MHz, $CDCl_3$) δ (ppm): 7.27 (d, J = 7.7 Hz, 2H), 7.12 (d, J = 7.7 Hz, 2H), 5.79 (tt, J = 56.6, 4.2 Hz, 1H), 2.99–2.56 (m, 2H), 2.40–1.85 (m, 2H); ^{19}F NMR (376 MHz, $CDCl_3$) δ (ppm): –117.17 (dt, J = 34.2, 16.6 Hz, 2F); ^{13}C NMR (101 MHz, $CDCl_3$) δ (ppm): 138.56, 132.42,

129.87, 128.98, 116.66 (t, J = 239.2 Hz), 35.75 (t, J = 21.2 Hz), 27.93 (t, J = 6.1 Hz). MS (EI) m/z: 190.0 $[M]^+$. HRMS (EI) m/z for $C_9H_9F_2Cl$ $[M]^+$: calc'd: 190.0361; found: 190.0352.

1-Bromo-4-(3-bromo-3-fluoropropyl)benzene (9a). General procedure 1 with 3-(4-bromophenyl)propanal followed by flash chromatography (*n*-hexane) gave **9a** as a colorless oil (195 mg, 27% yield).

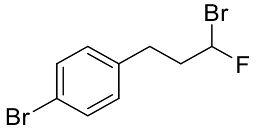

^{1}H NMR (400 MHz, $CDCl_3$) δ (ppm): 7.43 (d, J = 7.3 Hz, 2H), 7.08 (d, J = 7.6 Hz, 2H), 6.40 (dt, J = 50.2, 5.3 Hz, 1H), 2.80 (t, J = 7.6 Hz, 2H), 2.64–2.18 (m, 2H); ^{19}F NMR (376 MHz, $CDCl_3$) δ (ppm): –132.59 (ddd, J = 36.5, 19.4, 16.6 Hz, 1F); ^{13}C NMR (101 MHz, $CDCl_3$) δ (ppm): 138.53, 131.93, 130.35, 120.48, 94.52 (d, J = 252.4 Hz), 41.94 (d, J = 19.5 Hz), 30.78 (d, J = 4.4 Hz). MS (EI) m/z: 293.9 $[M]^+$. HRMS (EI) m/z for $C_9H_9FBr_2$ $[M]^+$: calc'd: 293.9055; found: 293.9042.

1-Bromo-4-(3,3-difluoropropyl)benzene (9). General procedure 2 with 3-(4-bromophenyl)propanal followed by flash chromatography (*n*-hexane) gave **9** as a colorless oil (308 mg, 66% yield).

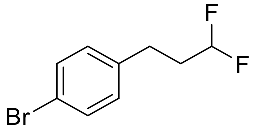

^{1}H NMR (400 MHz, $CDCl_3$) δ (ppm): 7.44 (d, J = 7.3 Hz, 2H), 7.09 (d, J = 7.7 Hz, 2H), 5.82 (tt, J = 56.6, 4.1 Hz, 1H), 2.92–2.53 (m, 2H), 2.52–1.66 (m, 2H); ^{19}F NMR (376 MHz, $CDCl_3$) δ (ppm): –117.05 (dt, J = 56.3, 17.0 Hz, 2F); ^{13}C NMR (101 MHz, $CDCl_3$) δ (ppm): 139.08, 131.88, 130.24, 120.35, 116.64 (t, J = 239.2 Hz), 35.61 (t, J = 21.2 Hz), 27.94 (t, J = 5.9 Hz). MS (EI) m/z: 234.0 $[M]^+$. HRMS (EI) m/z for $C_9H_9F_2Br$ $[M]^+$: calc'd: 233.9856; found: 233.9853.

4-(3-Bromo-3-fluoropropyl)-1,1'-biphenyl (10a). General procedure 1 with 3-([1,1'-biphenyl]-4-yl)propanal followed by flash chromatography (*n*-hexane) gave **10a** as a white solid (173 mg, 30% yield). Mp: 38–40 °C.

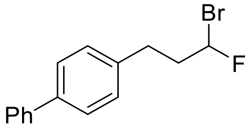

^{1}H NMR (400 MHz, $CDCl_3$) δ (ppm): 7.55 (dd, J = 14.5, 7.9 Hz, 4H), 7.43 (t, J = 7.6 Hz, 2H), 7.33 (t, J = 7.3 Hz, 1H), 7.27 (d, J = 8.0 Hz, 2H), 6.44 (dt, J = 50.2, 5.4 Hz, 1H), 2.87 (t, J = 7.6 Hz, 2H), 2.76–2.37 (m, 2H); ^{19}F NMR (376 MHz, $CDCl_3$) δ (ppm): –132.43 (ddd, J = 50.2, 20.2, 16.2 Hz, 1F); ^{13}C NMR (101 MHz, $CDCl_3$) δ (ppm): 141.00, 139.73, 138.66, 129.08, 128.99, 127.63, 127.44, 127.22, 94.89 (d, J = 252.2 Hz), 42.23 (d, J = 19.2 Hz), 31.06 (d, J = 4.4 Hz). MS (EI) m/z: 292.0 $[M]^+$. HRMS (EI) m/z for $C_{15}H_{14}FBr$ $[M]^+$: calc'd: 292.0263; found: 292.0258.

4-(3,3-Difluoropropyl)-1,1'-biphenyl (10). General procedure 2 with 3-([1,1'-biphenyl]-4-yl)propanal followed by flash chromatography (*n*-hexane) gave **10** as a white solid (300 mg, 65% yield). Mp: 45–47 °C.

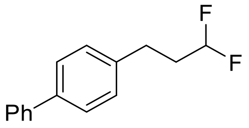

^{1}H NMR (400 MHz, $CDCl_3$) δ (ppm): 7.58 (dd, J = 15.1, 7.9 Hz, 4H), 7.45 (t, J = 7.6 Hz, 2H), 7.36 (t, J = 7.3 Hz, 1H), 7.29 (d, J = 8.0 Hz, 2H), 5.86 (tt, J = 56.7, 4.5 Hz, 1H), 3.14–2.51 (m, 2H), 2.45–1.93 (m, 2H); ^{19}F NMR (376 MHz, $CDCl_3$) δ (ppm): –117.07 (dt, J = 34.1, 17.4 Hz, 2F); ^{13}C NMR (101 MHz, $CDCl_3$) δ (ppm): 141.04, 139.63, 139.18, 128.99, 128.95, 127.59, 127.42, 127.22, 35.84 (t, J = 21.0 Hz), 28.24 (t, J = 6.0 Hz). [Known compound[7]].

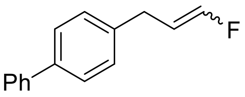

E/*Z* = 57/47

(*E*/*Z*)-4-(3-Fluoroallyl)-1,1'-biphenyl (10b). In a 4-mL glass vial equipped with a magnetic stirrer bar, 4-(3-bromo-3-fluoropropyl)-1,1'-biphenyl (0.4 mmol, 117 mg, 1.0 equiv.) was added to a solution of NMe_4F (0.8 mmol, 75 mg, 2.0 equiv.) in anhydrous CH_3CN (2.0 mL) at RT under He atmosphere. After 12 h, the reaction mixture was concentrated under reduced pressure. Sat. aq. NaCl solution was added. Then the mixture was extracted with diethyl ether. The combined organic phase was separated and washed with brine, dried (Mg_2SO_4), and concentrated under vacuum. The resulting residue was purified by silica gel flash column chromatography (*n*-hexane) to afford **10b** as a colorless oil (73 mg, *E*/*Z* = 57/47, 86% yield. ^{1}H NMR (400 MHz, $CDCl_3$) δ (ppm): 7.46 (dd, J = 17.3, 7.7 Hz, 4H), 7.34 (t, J = 7.5 Hz, 2H), 7.30–7.07 (m, 3H), 6.63–6.38 (m, 1H), 5.48 (ddt, J = 18.6, 11.1, 7.7 Hz, 0.56H), 4.90 (dtd, J = 41.8, 7.7, 4.7 Hz, 0.48H), 3.42 (d, J = 7.7 Hz, 0.91H), 3.19 (d, J = 7.7 Hz, 1.18H); ^{19}F NMR (376 MHz, $CDCl_3$) δ (ppm): –127.64 to –129.72 (m, 0.55F), –130.77 (dd, J = 85.2, 41.6 Hz, 0.47F); ^{13}C NMR (101 MHz, $CDCl_3$) δ (ppm): 151.01, 149.60, 148.47, 147.04, 128.96, 128.95, 128.91, 127.48, 127.38, 127.33, 127.23, 110.84 (d, J = 10.9 Hz), 110.02 (d, J = 5.1 Hz), 31.10 (d, J = 10.1 Hz), 28.77 (d, J = 5.9 Hz). MS (EI) *m/z*: 212.1 $[M]^+$. HRMS (EI) *m/z* for $C_{15}H_{13}F$ $[M]^+$: calc'd: 212.1001; found: 212.1007.

(*E*/*Z*)-4-(3-bromoallyl)-1,1'-biphenyl (10c)[10]**.**

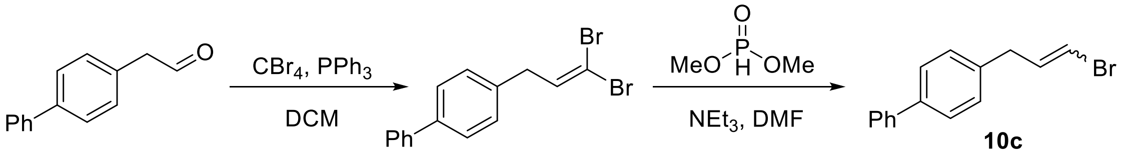

10c

E/*Z* = 8/5

To a flame-dried flask was added 2-([1,1'-biphenyl]-4-yl)acetaldehyde (589 mg, 3.0 mmol, 1.0 equiv.), CBr_4 (1.49 g, 4.5 mmol, 1.5 equiv.), and CH_2Cl_2 (10 mL). The flask was cooled to 0 °C, and then a solution of PPh_3 (2.36 g, 9.0 mmol, 3.0 equiv.) in CH_2Cl_2 (5.0 mL) was added dropwise from a syringe over 10 min. The solution was stirred at 0 °C under N_2 for 1 h. About half of the volume of CH_2Cl_2 was removed under reduced pressure. Hexane (50 mL) was added.

Triphenylphosphine oxide (TPPO) precipitated out. After filtration and evaporation of the filtrant, the residue was dissolved in hexane (50 mL) which led to further precipitation of TPPO. Filtration and evaporation of the solvent afforded crude 4-(3,3-dibromoallyl)-1,1'-biphenyl which was used directly in the next step, as follows To a solution of the crude 4-(3,3-dibromoallyl)-1,1'-biphenyl (~ 2.0 mmol, 1.0 equiv.) and NEt_3 (607 mg, 6.0 mmol, 3.0 equiv.) in DMF (3.0 mL) was added dimethyl phosphonate (660 mg, 6.0 mmol, 3.0 equiv.). The solution was stirred overnight at room temperature. Water (10 mL) was added to the mixture, which was then extracted with diethyl ether (50 mL). The combined organic phase was separated, washed with brine, dried (Mg_2SO_4), and concentrated under vacuum. The residue was purified by silica gel flash column chromatography (*n*-hexane) to afford **10c** as a white solid (293 mg, *E*/*Z* = 8/5, 36% yield). Mp: 42–44 °C.

^{1}H NMR (400 MHz, $CDCl_3$) δ (ppm): 7.69–7.55 (m, 4H), 7.51–7.47 (m, 2H), 7.45–7.27 (m, 3H), 6.56–6.30 (m, 1.33H), 6.18 (dt, *J* = 13.5, 1.4 Hz, 0.58H), 3.84–3.55 (m, 0.79H), 3.45 (d, *J* = 7.1 Hz, 1.24H); ^{13}C NMR (101 MHz, $CDCl_3$) δ (ppm): 141.08, 141.00, 139.73, 139.60, 137.95, 137.46, 136.63, 133.58, 129.12, 129.04, 128.96, 128.94, 127.54, 127.53, 127.41, 127.35, 127.21, 108.98, 106.21, 38.91, 35.83. MS (EI) *m/z*: 272.0 $[M]^+$. HRMS (EI) *m/z* for $C_{15}H_{13}Br$ $[M]^+$: calc'd: 272.0201; found: 272.0201.

(3-Bromo-3-fluoropropane-1,1-diyl)dibenzene (11a). General procedure 1 with 3,3-diphenylpropanal followed by flash chromatography (*n*-hexane) gave **11a** as a colorless oil (93 mg, 16% yield).

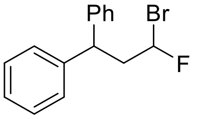

^{1}H NMR (400 MHz, $CDCl_3$) δ (ppm): 7.31 (ddd, *J* = 10.5, 5.4, 2.9 Hz, 4H), 7.26–7.15 (m, 6H), 6.14 (ddd, *J* = 50.3, 6.8, 5.4 Hz, 1H), 4.22 (t, *J* = 8.0 Hz, 1H), 3.03 (ddt, *J* = 14.3, 10.9, 7.1 Hz, 1H), 2.86 (ddd, *J* = 23.1, 14.2, 8.6, 5.4 Hz, 1H); ^{19}F NMR (376 MHz, $CDCl_3$) δ (ppm): –133.28 (ddd, *J* = 50.4, 23.3, 10.9 Hz, 1F); ^{13}C NMR (101 MHz, $CDCl_3$) δ (ppm): 142.59 (d, *J* = 30.0 Hz), 129.05 (d, *J* = 3.8 Hz), 127.91 (d, *J* = 17.9 Hz), 127.11 (d, *J* = 1.4 Hz), 94.37 (d, *J* = 251.6 Hz), 47.58 (d, *J* = 4.2 Hz), 46.56 (d, *J* = 19.3 Hz). MS (EI) *m/z*: 292.0 $[M]^+$. HRMS (EI) *m/z* for $C_{15}H_{14}FBr$ $[M]^+$: calc'd: 292.0263; found: 292.0254.

(3,3-Difluoropropane-1,1-diyl)dibenzene (11). General procedure 2 with 3,3-diphenylpropanal followed by flash chromatography (*n*-hexane) gave **11** as a colorless oil (115 mg, 50% yield).

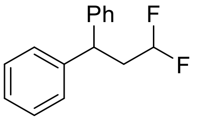

^{1}H NMR (400 MHz, $CDCl_3$) δ (ppm): 7.34–7.26 (m, 4H), 7.26–7.13 (m, 6H), 5.57 (tt, J = 56.7, 5.1 Hz, 1H), 4.17 (t, J = 8.1 Hz, 1H), 2.57 (tdd, J = 15.8, 8.2, 5.1 Hz, 1H); ^{19}F NMR (376 MHz, $CDCl_3$) δ (ppm): –116.94 (dt, J = 56.9, 15.9 Hz, 2F); ^{13}C NMR (101 MHz, $CDCl_3$) δ (ppm): 143.02, 128.94, 127.75, 126.95, 116.71 (t, J = 238.8 Hz), 45.45 (t, J = 6.0 Hz), 39.91 (t, J = 21.4 Hz). [Known compound[11]].

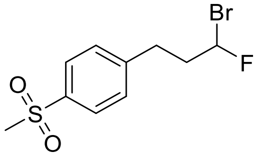

1-(3-Bromo-3-fluoropropyl)-4-(methylsulfonyl)benzene (12a). General procedure 1 with 3-(4-(methylsulfonyl)phenyl)propanal followed by flash chromatography (ethyl acetate/*n*-hexane, 1: 3) gave **12a** as a white solid (126 mg, 21% yield). Mp: 62–64 °C.

^{1}H NMR (400 MHz, $CDCl_3$) δ (ppm): 7.89 (d, J = 8.3 Hz, 2H), 7.41 (d, J = 8.2 Hz, 2H), 6.44 (dt, J = 50.0, 5.2 Hz, 1H), 3.05 (s, 3H), 3.00–2.89 (m, 2H), 2.70–2.37 (m, 2H); ^{19}F NMR (376 MHz, $CDCl_3$) δ (ppm): –127.44 to –140.13 (m, 1F); ^{13}C NMR (101 MHz, $CDCl_3$) δ (ppm): 146.24, 139.10, 129.64, 128.05, 94.09 (d, J = 252.7 Hz), 44.73, 41.58 (d, J = 19.5 Hz), 31.25 (d, J = 4.3 Hz). MS (EI) *m*/z: 294.9 $[M]^+$. HRMS (EI) *m/z* for $C_{10}H_{12}FO_2BrS$ $[M]^+$: calc'd: 293.9725; found: 293.9724.

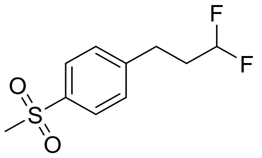

1-(3,3-Difluoropropyl)-4-(methylsulfonyl)benzene (12). General procedure 2 with 3-(4-(methylsulfonyl)phenyl)propanal followed by flash chromatography (ethyl acetate/*n*-hexane, 1: 3) gave **12** as a white solid (116 mg, 50% yield). Mp: 58–60 °C.

^{1}H NMR (400 MHz, $CDCl_3$) δ (ppm): 7.87 (d, J = 8.3 Hz, 2H), 7.40 (d, J = 8.3 Hz, 2H), 5.83 (tt, J = 56.4, 4.3 Hz, 1H), 3.03 (s, 3H), 2.88 (dd, J = 9.1, 7.0 Hz, 2H), 2.39–1.92 (m, 2H); ^{19}F NMR (376 MHz, $CDCl_3$) δ (ppm): –117.15 (dt, J = 34.3, 16.8 Hz, 2F); ^{13}C NMR (101 MHz, $CDCl_3$) δ (ppm): 146.69, 138.95, 129.49, 127.95, 116.33 (t, J = 239.5 Hz), 44.68, 35.27 (t, J = 21.4 Hz), 28.33 (t, J = 6.0 Hz). MS (EI) *m/z*: 234.0 $[M]^+$. HRMS (EI) *m/z* for $C_{10}H_{12}F_2O_2S$ $[M]^+$: calc'd: 234.0526; found: 234.0525.

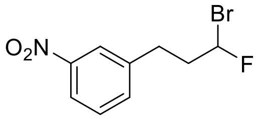

1-(3-Bromo-3-fluoropropyl)-3-nitrobenzene (13a). General procedure 1 with 3-(3-nitrophenyl)propanal followed by flash chromatography (ethyl acetate/*n*-hexane, 1: 10) gave **13a** as a colorless oil (100 mg, 19% yield).

^{1}H NMR (400 MHz, $CDCl_3$) δ (ppm): 8.10 (d, J = 7.9 Hz, 2H), 7.55 (d, J = 7.6 Hz, 1H), 7.49 (t, J = 7.6 Hz, 1H), 6.47 (dt, J = 50.0, 5.2 Hz, 1H), 3.08–2.83 (m, 2H), 2.82–2.32 (m, 2H); ^{19}F NMR

(376 MHz, $CDCl_3$) δ (ppm): –133.03 (dt, J = 50.0, 18.3 Hz, 1F); ^{13}C NMR (101 MHz, $CDCl_3$) δ (ppm): 148.69, 141.70, 134.91, 129.85, 123.47, 121.95, 94.03 (d, J = 252.6 Hz), 41.61 (d, J = 19.7 Hz), 30.96 (d, J = 4.3 Hz). MS (EI) m/z: 261.0 $[M]^+$. HRMS (EI) m/z for $C_9H_9FO_2BrN$ $[M]^+$: calc'd: 260.9800; found: 260.9808.

1-(3,3-Difluoropropyl)-3-nitrobenzene (13). General procedure 2 with 3-(3-nitrophenyl)propanal followed by flash chromatography (ethyl acetate/*n*-hexane, 1: 10) gave **13** as a colorless oil (306 mg, 76% yield).

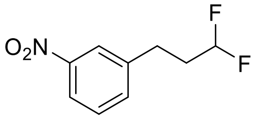

^1H NMR (400 MHz, $CDCl_3$) δ (ppm): 8.09 (d, J = 7.5 Hz, 2H), 7.55 (d, J = 7.6 Hz, 1H), 7.49 (t, J = 7.6 Hz, 1H), 5.86 (tt, J = 56.3, 4.2 Hz, 1H), 3.23–2.52 (m, 2H), 2.49–1.78 (m, 2H); ^{19}F NMR (376 MHz, $CDCl_3$) δ (ppm):–117.17 (dt, J = 34.1, 16.6 Hz, 2F); ^{13}C NMR (101 MHz, $CDCl_3$) δ (ppm): 148.72, 142.17, 134.80, 129.82, 123.38, 121.89, 116.30 (t, J = 239.6 Hz), 35.42 (t, J = 21.5 Hz), 28.10 (t, J = 5.9 Hz). MS (EI) m/z: 201.0 $[M]^+$. HRMS (EI) m/z for $C_9H_9F_2O_2N$ $[M]^+$: calc'd: 201.0601; found: 201.0605.

4-(3-Bromo-3-fluoropropyl)benzonitrile (14a). General procedure 1 with 4-(3-oxopropyl)benzonitrile followed by flash chromatography (ethyl acetate/*n*-hexane, 1: 10) gave **14a** as a colorless oil (89 mg, 18% yield).

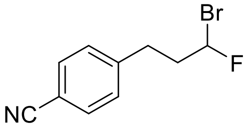

^1H NMR (400 MHz, $CDCl_3$) δ (ppm): 7.59 (d, J = 8.1 Hz, 2H), 7.31 (d, J = 8.1 Hz, 2H), 6.43 (dt, J = 50.0, 5.2 Hz, 1H), 3.12–2.74 (m, 2H), 2.74–2.30 (m, 2H); ^{19}F NMR (376 MHz, $CDCl_3$) δ (ppm): –121.23 to –143.82 (m, 1F); ^{13}C NMR (101 MHz, $CDCl_3$) δ (ppm): 145.20, 132.58, 129.38, 118.87, 110.58, 94.11 (d, J = 252.5 Hz), 41.35 (d, J = 19.5 Hz), 31.31 (d, J = 4.4 Hz). MS (EI) m/z: 241.0 $[M]^+$. HRMS (EI) m/z for $C_{10}H_9FBrN$ $[M]^+$: calc'd: 240.9902; found: 240.9897.

4-(3,3-Difluoropropyl)benzonitrile (14). General procedure 2 with 4-(3-oxopropyl)benzonitrile followed by flash chromatography (ethyl acetate/*n*-hexane, 1: 10) gave **14** as a colorless oil (69 mg, 38% yield).

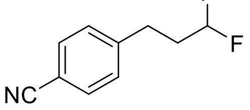

^1H NMR (400 MHz, $CDCl_3$) δ (ppm): 7.59 (d, J = 8.2 Hz, 2H), 7.31 (d, J = 8.1 Hz, 2H), 5.83 (tt, J = 56.4, 4.3 Hz, 1H), 3.13–2.60 (m, 2H), 2.63–1.80 (m, 2H); ^{19}F NMR (376 MHz, $CDCl_3$) δ (ppm): –117.15 (dt, J = 56.6, 17.2 Hz, 2F); ^{13}C NMR (101 MHz, $CDCl_3$) δ (ppm): 145.72, 132.60, 129.30, 118.95, 116.31 (t, J = 239.5 Hz), 110.57, 35.16 (t, J = 21.5 Hz), 28.49 (t, J = 6.0 Hz). [Known compound[12]].

Methyl 4-(3-bromo-3-fluoropropyl)benzoate (15a). General procedure 1 with methyl 4-(3-oxopropyl)benzoate followed by flash chromatography (ethyl acetate/*n*-hexane, 1: 10) gave **15a** as a colorless oil (179 mg, 33% yield).

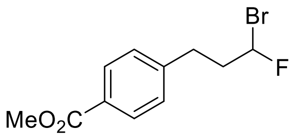

^{1}H NMR (400 MHz, $CDCl_3$) δ (ppm): 7.98 (d, J = 7.3 Hz, 2H), 7.27 (d, J = 8.0 Hz, 2H), 6.42 (dt, J = 50.1, 5.1 Hz, 1H), 3.91 (s, 3H), 2.90 (t, J = 7.7 Hz, 2H), 2.76–2.28 (m, 2H); ^{19}F NMR (376 MHz, $CDCl_3$) δ (ppm): –132.73 (ddd, J = 36.6, 19.8, 17.7 Hz, 1F); ^{13}C NMR (101 MHz, $CDCl_3$) δ (ppm): 167.00, 144.97, 130.16, 128.71, 128.62, 94.44 (d, J = 252.4 Hz), 52.21, 41.71 (d, J = 19.6 Hz), 31.32 (d, J = 4.4 Hz). MS (EI) *m/z*: 274.0 $[M]^+$. HRMS (EI) *m/z* for $C_{11}H_{12}FO_2Br$ $[M]^+$: calc'd: 274.0005; found: 273.9994.

Methyl 4-(3,3-difluoropropyl)benzoate (15). General procedure 2 with methyl 4-(3-oxopropyl)benzoate followed by flash chromatography (ethyl acetate/*n*-hexane, 1: 10) gave **15** as a white solid (206 mg, 48% yield). Mp: 50–52 °C.

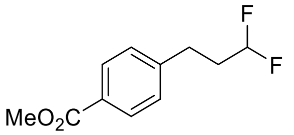

^{1}H NMR (400 MHz, $CDCl_3$) δ (ppm): 7.98 (d, J = 7.3 Hz, 2H), 7.27 (d, J = 7.6 Hz, 2H), 5.81 (tt, J = 56.6, 4.1 Hz, 1H), 3.90 (s, 3H), 2.96–2.72 (m, 2H), 2.35–1.97 (m, 2H); ^{19}F NMR (376 MHz, $CDCl_3$) δ (ppm): –117.14 (dt, J = 56.6, 17.0 Hz, 2F); ^{13}C NMR (101 MHz, $CDCl_3$) δ (ppm): 167.06, 145.50, 130.15, 128.65, 128.52, 116.57 (t, J = 239.3 Hz), 52.20, 35.41 (t, J = 21.3 Hz), 28.49 (t, J = 6.0 Hz). [Known compound[13]].

2-(5-Bromo-5-fluoropentyl)thiophene (16a). General procedure 1 with 5-(thiophen-2-yl)pentanal followed by flash chromatography (*n*-hexane) gave **16a** as a colorless oil (117 mg, 23% yield).

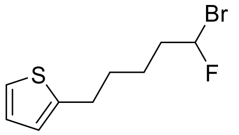

^{1}H NMR (400 MHz, $CDCl_3$) δ (ppm): 7.14 (dd, J = 5.1, 1.1 Hz, 1H), 6.94 (dd, J = 5.1, 3.4 Hz, 1H), 6.87–6.77 (m, 1H), 6.47 (dt, J = 50.4, 5.4 Hz, 1H), 2.88 (t, J = 7.5 Hz, 2H), 2.48–2.03 (m, 2H), 1.94–1.68 (m, 2H), 1.68–1.45 (m, 2H); ^{19}F NMR (376 MHz, $CDCl_3$) δ (ppm): –124.93 to –152.82 (m, 1F); ^{13}C NMR (101 MHz, $CDCl_3$) δ (ppm): 144.84, 126.94, 124.44, 123.25, 95.54 (d, J = 252.5 Hz), 40.50 (d, J = 18.8 Hz), 30.98, 29.78, 24.68 (d, J = 3.8 Hz). MS (EI) *m/z*: 249.9 $[M]^+$. HRMS (EI) *m/z* for $C_9H_{12}FSBr$ $[M]^+$: calc'd: 249.9827; found: 249.9823.

2-(5,5-Difluoropentyl)thiophene (16). General procedure 2 with 5-(thiophen-2-yl)pentanal followed by flash chromatography (*n*-hexane) gave **16** as a colorless oil (67 mg, 35% yield).

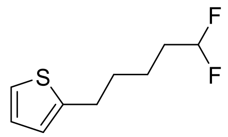

^{1}H NMR (400 MHz, $CDCl_3$) δ (ppm): 7.13 (dd, J = 5.1, 1.1 Hz, 1H), 6.93 (dd, J = 5.1, 3.4 Hz, 1H), 6.87–6.71 (m, 1H), 5.81 (tt, J = 56.9, 4.5 Hz, 1H), 2.87 (t, J = 7.5 Hz, 2H), 2.02–1.80 (m, 2H), 1.75 (dd, J = 15.3, 7.7 Hz, 2H), 1.54 (tt, J = 10.4, 6.3 Hz, 2H); ^{19}F NMR (376 MHz, $CDCl_3$) δ (ppm): –115.77 (dt, J = 57.0, 17.6 Hz, 2F); ^{13}C NMR (101 MHz, $CDCl_3$) δ (ppm): 144.93, 126.96, 124.43, 123.24, 117.45 (t, J = 238.9 Hz), 34.03 (t, J = 20.7 Hz), 31.36, 29.83, 21.77 (t, J = 5.5 Hz). [Known compound[14]].

4-(4-Bromo-4-fluorobutyl)-1,2-dimethoxybenzene (17a). General procedure 4 with 4-allyl-1,2-dimethoxybenzene followed by flash chromatography (ethyl acetate/*n*-hexane, 1: 20) gave **17a** as a colorless oil (41 mg, 35% yield).

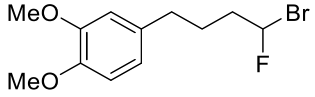

^{1}H NMR (400 MHz, $CDCl_3$) δ (ppm): 6.80 (d, J = 7.9 Hz, 1H), 6.71 (d, J = 9.1 Hz, 2H), 6.45 (dt, J = 50.4, 5.3 Hz, 1H), 3.87 (s, 3H), 3.86 (s, 3H), 2.63 (t, J = 7.6 Hz, 2H), 2.38–2 .04 (m, 2H), 1.96–1.70 (m, 2H); ^{19}F NMR (376 MHz, $CDCl_3$) δ (ppm): –121.86 to – 146.61 (m, 1F); ^{13}C NMR (101 MHz, $CDCl_3$) δ (ppm): 149.11, 147.58, 133.91, 120.39, 111.78, 111.46, 95.57 (d, J = 252.4 Hz), 56.06 (d, J = 8.0 Hz), 40.10 (d, J = 19.0 Hz), 34.54, 26.92 (d, J = 3.6 Hz). MS (EI) m/z: 290.0 $[M]^+$. HRMS (EI) m/z for $C_{12}H_{16}FO_2Br$ $[M]^+$: calc'd: 290.0318; found: 290.0326.

4-(4,4-Difluorobutyl)-1,2-dimethoxybenzene (17). General procedure 45 with 4-allyl-1,2-dimethoxybenzene followed by flash chromatography (ethyl acetate/*n*-hexane, 1: 20) gave **17** as a colorless oil (38 mg, 55% yield).

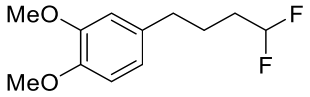

^{1}H NMR (400 MHz, $CDCl_3$) δ (ppm): 6.80 (d, J = 7.8 Hz, 1H), 6.71 (d, J = 8.3 Hz, 2H), 6.08–5.51 (m, 1H), 3.87 (s, 3H), 3.86 (s, 3H), 2.62 (t, J = 7.3 Hz, 2H), 2.18–1.63 (m, 4H); ^{19}F NMR (376 MHz, $CDCl_3$) δ (ppm): –115.73 (dt, J = 56.5, 17.1 Hz, 2F); ^{13}C NMR (101 MHz, $CDCl_3$) δ (ppm): 149.10, 147.56, 134.07, 120.40, 117.45 (t, J = 238.8 Hz), 111.81, 111.44, 56.05 (d, J = 9.5 Hz), 34.90, 33.65 (t, J = 20.9 Hz), 24.06 (t, J = 5.2 Hz). [Known compound[8]].

5-Bromo-5-fluoropentyl 3-fluorobenzoate (18a). General procedure 4 with but-3-en-1-yl 3-fluorobenzoate followed by flash chromatography (ethyl acetate/*n*-hexane, 1: 10) gave **18a** as a colorless oil (53 mg, 43% yield).

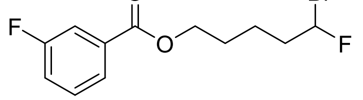

^{1}H NMR (400 MHz, $CDCl_3$) δ (ppm): 7.83 (d, *J* = 7.7 Hz, 1H), 7.71 (d, *J* = 9.3 Hz, 1H), 7.42 (td, *J* = 8.0, 5.6 Hz, 1H), 7.26 (td, *J* = 8.3, 2.6 Hz, 1H), 6.50 (dt, *J* = 50.3, 5.3 Hz, 1H), 4.35 (t, *J* = 6.4 Hz, 2H), 2.53–2.00 (m, 2H), 1.85 (dt, *J* = 14.1, 6.9 Hz, 2H), 1.78–1.54 (m, 2H); ^{19}F NMR (376 MHz, $CDCl_3$) δ –103.59 to – 120.95 (m, 1F), –122.76 to – 135.45 (m, 1F); ^{13}C NMR (101 MHz, $CDCl_3$) δ (ppm): 165.49 (d, *J* = 3.0 Hz), 162.65 (d, *J* = 247.1 Hz), 132.49 (d, *J* = 7.5 Hz), 130.15 (d, *J* = 7.8 Hz), 125.39 (d, *J* = 3.2 Hz), 120.14 (d, *J* = 21.3 Hz), 116.55 (d, *J* = 23.0 Hz), 95.16 (d, *J* = 252.4 Hz), 64.82, 40.15 (d, *J* = 19.2 Hz), 27.90, 21.82 (d, *J* = 4.2 Hz). MS (EI) *m*/*z*: 306.0 $[M]^+$. HRMS (EI) *m*/*z* for $C_{12}H_{13}F_2O_2Br$ $[M]^+$: calc'd: 306.0067; found: 306.0073.

5,5-Difluoropentyl 3-fluorobenzoate (18). General procedure 5 with but-3-en-1-yl 3-fluorobenzoate followed by flash chromatography (ethyl acetate/*n*-hexane, 1: 10) gave **18** as a colorless oil (38 mg, 51% yield).

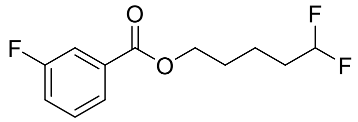

^{1}H NMR (400 MHz, $CDCl_3$) δ (ppm): 7.83 (d, *J* = 7.7 Hz, 1H), 7.71 (d, *J* = 9.3 Hz, 1H), 7.42 (td, *J* = 8.0, 5.7 Hz, 1H), 7.26 (td, *J* = 8.3, 2.5 Hz, 1H), 5.84 (tt, *J* = 56.7, 4.3 Hz, 1H), 4.35 (t, *J* = 6.4 Hz, 2H), 2.08–1.72 (m, 4H), 1.72–1.43 (m, 2H); ^{19}F NMR (376 MHz, $CDCl_3$) δ (ppm): –111.55 to – 113.31 (m, 1F), –116.06 (dt, *J* = 57.1, 17.6 Hz, 2F); ^{13}C NMR (101 MHz, $CDCl_3$) δ (ppm): 165.61 (d, *J* = 2.9 Hz), 162.75 (d, *J* = 247.1 Hz), 132.61 (d, *J* = 7.4 Hz), 130.24 (d, *J* = 7.7 Hz), 125.48 (d, *J* = 3.0 Hz), 120.22 (d, *J* = 21.3 Hz), 117.23 (t, *J* = 238.9 Hz), 116.63 (d, *J* = 23.2 Hz), 64.97, 33.84 (t, *J* = 21.0 Hz), 28.36, 18.98 (t, *J* = 5.7 Hz). MS (EI) *m*/z: 246.1 $[M]^+$. HRMS (EI) *m*/*z* for $C_{12}H_{13}F_3O_2$ $[M]^+$: calc'd: 246.0868; found: 246.0863.

5-Bromo-5-fluoropentyl 4-iodobenzoate (19a). General procedure 4 with but-3-en-1-yl 4-iodobenzoate followed by flash chromatography (ethyl acetate/*n*-hexane, 1: 10) gave **19a** as a colorless oil (96 mg, 58% yield).

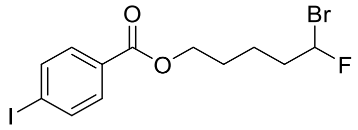

^{1}H NMR (400 MHz, $CDCl_3$) δ (ppm): 7.86–7.77 (m, 2H), 7.77–7.65 (m, 2H), 6.49 (dt, *J* = 50.3, 5.3 Hz, 1H), 4.33 (t, *J* = 6.4 Hz, 2H), 2.51–2.08 (m, 2H), 1.84 (dt, *J* = 14.2, 6.9 Hz, 2H), 1.77–1.62 (m, 2H); ^{19}F NMR (376 MHz, $CDCl_3$) δ (ppm): –119.98 to –143.82 (m, 1F); ^{13}C NMR (101 MHz, $CDCl_3$) δ (ppm): 166.24, 137.95, 131.19, 129.87, 100.98, 95.23 (d, *J* = 252.4 Hz), 64.80, 40.23 (d, *J* = 19.2 Hz), 27.99, 21.91 (d, *J* = 3.9 Hz). MS (EI) *m*/*z*: 413.9 $[M]^+$. HRMS (EI) *m*/*z* for $C_{12}H_{13}FO_2BrI$ $[M]^+$: calc'd: 413.9128; found: 413.9121.

5,5-Difluoropentyl 4-iodobenzoate (19). General procedure 5 with but-3-en-1-yl 3-fluorobenzoate followed by flash chromatography (ethyl acetate/*n*-hexane, 1: 10) gave **19** as a colorless oil (72 mg, 68% yield).

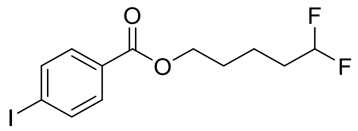

^{1}H NMR (400 MHz, $CDCl_3$) δ (ppm): 7.80 (d, J = 8.4 Hz, 2H), 7.73 (d, J = 8.4 Hz, 2H), 5.83 (tt, J = 56.7, 4.3 Hz, 1H), 4.33 (t, J = 6.4 Hz, 2H), 2.07–1.73 (m, 4H), 1.72 – 1.54 (m, 2H); ^{19}F NMR (376 MHz, $CDCl_3$) δ (ppm): –116.05 (dt, J = 34.7, 17.6 Hz, 2F); ^{13}C NMR (101 MHz, $CDCl_3$) δ (ppm): 166.26, 137.95, 131.19, 129.90, 117.21 (t, J = 238.9 Hz), 100.95, 64.86, 33.84 (t, J = 21.1 Hz), 28.37, 18.98 (t, J = 5.7 Hz). [Known compound[8]].

5-Bromo-5-fluoropentyl 4-cyanobenzoate (20a). General procedure 4 with but-3-en-1-yl 4-cyanobenzoate followed by flash chromatography (ethyl acetate/*n*-hexane, 1: 10) gave **20a** as a colorless oil (79 mg, 63% yield).

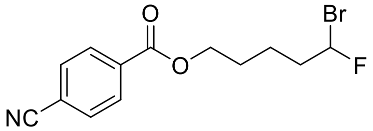

^{1}H NMR (400 MHz, $CDCl_3$) δ (ppm): 8.35–8.00 (m, 2H), 8.00–7.55 (m, 2H), 6.47 (dt, J = 50.3, 5.2 Hz, 1H), 4.36 (t, J = 6.4 Hz, 2H), 2.44–2.09 (m, 2H), 1.84 (dt, J = 14.2, 7.0 Hz, 2H), 1.78–1.62 (m, 2H); ^{19}F NMR (376 MHz, $CDCl_3$) δ (ppm): –131.23 (dt, J = 50.3, 19.1 Hz, 1F); ^{13}C NMR (101 MHz, $CDCl_3$) δ (ppm): 165.07, 134.18, 132.44, 130.25, 118.13, 116.63, 95.14 (d, J = 252.4 Hz), 65.34, 40.15 (d, J = 19.2 Hz), 27.92, 21.82 (d, J = 3.9 Hz). MS (EI) *m/z*: 313.0 $[M]^+$. HRMS (EI) *m/z* for $C_{13}H_{13}FO_2BrN$ $[M]^+$: calc'd: 313.0114; found: 313.0109.

5,5-Difluoropentyl 4-cyanobenzoate (20). General procedure 5 with but-3-en-1-yl 4-cyanobenzoate followed by flash chromatography (ethyl acetate/*n*-hexane, 1: 10) gave **20** as a white solid (42 mg, 55% yield). Mp: 81–83 °C.

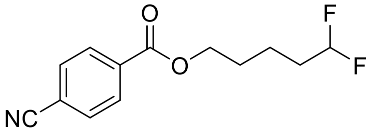

^{1}H NMR (400 MHz, $CDCl_3$) δ (ppm): 8.12 (d, J = 8.3 Hz, 2H), 7.74 (d, J = 8.3 Hz, 2H), 5.84 (tt, J = 56.7, 4.3 Hz, 1H), 4.37 (t, J = 6.4 Hz, 2H), 2.08–1.72 (m, 4H), 1.72–1.43 (m, 2H); ^{19}F NMR (376 MHz, $CDCl_3$) δ (ppm):–116.14 (dt, J = 57.0, 17.6 Hz, 2F); ^{13}C NMR (101 MHz, $CDCl_3$) δ (ppm): 165.09, 134.21, 132.43, 130.24, 118.14, 117.13 (t, J = 239.1 Hz), 116.62, 65.40, 33.78 (t, J = 21.1 Hz), 28.29, 18.89 (t, J = 5.6 Hz). MS (EI) *m/z*: 253.1 $[M]^+$. HRMS (EI) *m/z* for $C_{13}H_{13}F_2O_2N$ $[M]^+$: calc'd: 253.0914; found: 253.0910.

5-Bromo-5-fluoropentyl 4-(trifluoromethyl)benzoate (21a). General procedure 4 with but-3-en-1-yl 4-(trifluoromethyl)benzoate followed by flash chromatography (ethyl acetate/*n*-hexane, 1: 20) gave **21a** as a colorless oil (109 mg, 76% yield).

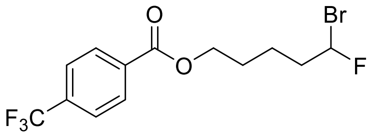

^{1}H NMR (400 MHz, $CDCl_3$) δ (ppm): 8.15 (d, J = 8.2 Hz, 2H), 7.71 (d, J = 8.3 Hz, 2H), 6.50 (dt, J = 50.3, 5.3 Hz, 1H), 4.38 (t, J = 6.4 Hz, 2H), 2.64–2.07 (m, 2H), 1.87 (dt, J = 14.2, 6.9 Hz, 2H), 1.80–1.58 (m, 2H); ^{19}F NMR (376 MHz, $CDCl_3$) δ (ppm): –63.72 (s, 3F), –131.80 (dt, J = 50.2, 18.9 Hz, 1F); ^{13}C NMR (101 MHz, $CDCl_3$) δ (ppm): 165.54, 134.70 (q, J = 32.7 Hz), 133.63, 130.17, 125.66 (q, J = 3.7 Hz), 123.83 (q, J = 272.8 Hz), 95.19 (d, J = 252.4 Hz), 65.12, 40.23 (d, J = 19.2 Hz), 28.00, 21.90 (d, J = 4.0 Hz). MS (EI) *m*/*z*: 356.0 [M]$^+$. HRMS (EI) *m*/*z* for $C_{13}H_{13}F_4O_2Br$ [M]$^+$: calc'd: 356.0035; found: 356.0034.

5,5-Difluoropentyl 4-(trifluoromethyl)benzoate (21). General procedure 5 with but-3-en-1-yl 4-(trifluoromethyl)benzoate followed by flash chromatography (ethyl acetate/*n*-hexane, 1: 20) gave **21** as a colorless oil (51 mg, 57% yield).

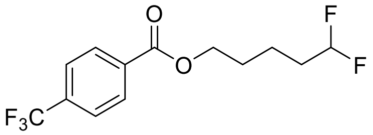

^{1}H NMR (400 MHz, $CDCl_3$) δ (ppm): 8.14 (d, J = 8.3 Hz, 2H), 7.70 (d, J = 8.3 Hz, 2H), 5.84 (tt, J = 56.7, 4.3 Hz, 1H), 4.38 (t, J = 6.4 Hz, 2H), 2.10–1.72 (m, 4H), 1.72–1.38 (m, 2H); ^{19}F NMR (376 MHz, $CDCl_3$) δ (ppm):–63.72 (s, 3F), –116.69 (dt, J = 56.4, 17.5 Hz, 2F); ^{13}C NMR (101 MHz, $CDCl_3$) δ (ppm): 165.54, 134.67 (q, J = 32.9 Hz), 133.66, 130.16, 125.64 (q, J = 3.7 Hz), 123.84 (q, J = 272.7 Hz), 117.20 (t, J = 239.1 Hz), 65.17, 33.84 (t, J = 21.0 Hz), 28.35, 18.96 (t, J = 5.6 Hz). MS (EI) *m*/*z*: 296.1 [M]$^+$. HRMS (EI) *m*/*z* for $C_{13}H_{13}F_5O_2$ [M]$^+$: calc'd: 296.0836; found: 296.0844.

5-Bromo-5-fluoropentyl benzofuran-2-carboxylate (22a). General procedure 4 with but-3-en-1-yl benzofuran-2-carboxylate followed by flash chromatography (ethyl acetate/*n*-hexane, 1: 10) gave **22a** as a colorless oil (79 mg, 60% yield).

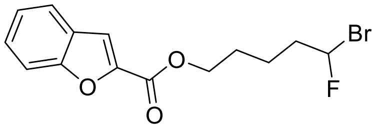

^{1}H NMR (400 MHz, $CDCl_3$) δ (ppm): 7.69 (d, J = 7.9 Hz, 1H), 7.60 (d, J = 8.4 Hz, 1H), 7.53 (s, 1H), 7.45 (t, J = 7.8 Hz, 1H), 7.31 (t, J = 7.5 Hz, 1H), 6.50 (dt, J = 50.3, 5.3 Hz, 1H), 4.41 (t, J = 6.4 Hz, 2H), 2.61–2.07 (m, 2H), 2.07–1.81 (m, 2H), 1.83–1.64 (m, 2H); ^{19}F NMR (376 MHz, $CDCl_3$) δ (ppm): –123.11 to – 145.98 (m, 1F); ^{13}C NMR (101 MHz, $CDCl_3$) δ (ppm): 159.75, 155.95, 145.60, 127.89, 127.11, 124.02, 123.03, 114.18, 112.58, 95.23 (d, J = 252.4 Hz), 65.03,

40.24 (d, J = 19.1 Hz), 27.98, 21.88 (d, J = 3.8 Hz). MS (EI) m/z: 327.9 $[M]^+$. HRMS (EI) m/z for $C_{14}H_{14}O_3FBr$ $[M]^+$: calc'd: 328.0110; found: 328.0096.

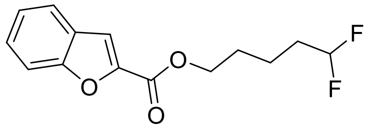

5,5-Difluoropentyl benzofuran-2-carboxylate (22). General procedure 5 with but-3-en-1-yl benzofuran-2-carboxylate followed by flash chromatography (ethyl acetate/*n*-hexane, 1: 10) gave **22** as a colorless oil (36 mg, 45% yield).

^{1}H NMR (400 MHz, $CDCl_3$) δ (ppm): 7.67 (d, J = 7.9 Hz, 1H), 7.58 (d, J = 8.4 Hz, 1H), 7.51 (s, 1H), 7.43 (t, J = 7.8 Hz, 1H), 7.29 (t, J = 7.5 Hz, 1H), 5.84 (tt, J = 56.7, 4.3 Hz, 1H), 4.38 (t, J = 6.4 Hz, 2H), 2.11–1.76 (m, 4H), 1.70–1.49 (m, 2H); ^{19}F NMR (376 MHz, $CDCl_3$) δ (ppm): –116.01 (dt, J = 57.0, 17.6 Hz, 2F); ^{13}C NMR (101 MHz, $CDCl_3$) δ (ppm): 159.69, 155.88, 145.58, 127.81, 127.06, 123.96, 122.97, 117.20 (t, J = 238.9 Hz), 114.07, 112.49, 65.00, 33.75 (t, J = 21.0 Hz), 28.28, 18.87 (t, J = 5.6 Hz). MS (EI) m/z: 268.1 $[M]^+$. HRMS (EI) m/z for $C_{14}H_{14}O_3F_2$ $[M]^+$: calc'd: 268.0911; found: 268.0904.

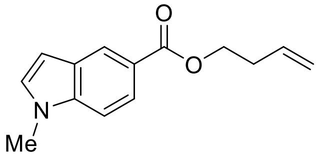

But-3-en-1-yl 1-methyl-1*H*-indole-5-carboxylate (23b). General procedure 3 with 1-methyl-1*H*-indole-5-carboxylic acid followed by flash chromatography (ethyl acetate/*n*-hexane, 1: 10) gave **23b** as a colorless oil (863 mg, 75% yield).

^{1}H NMR (400 MHz, $CDCl_3$) δ (ppm): 8.41 (s, 1H), 7.94 (d, J = 8.7 Hz, 1H), 7.31 (d, J = 8.7 Hz, 1H), 7.10 (d, J = 2.9 Hz, 1H), 6.59 (d, J = 2.8 Hz, 1H), 5.93 (ddt, J = 17.0, 10.2, 6.7 Hz, 1H), 5.17 (dd, J = 31.2, 13.7 Hz, 2H), 4.40 (t, J = 6.7 Hz, 2H), 3.80 (s, 3H), 2.57 (q, J = 6.7 Hz, 2H); ^{13}C NMR (101 MHz, $CDCl_3$) δ (ppm): 167.88, 139.26, 134.53, 130.38, 128.12, 124.03, 123.08, 121.67, 117.32, 108.97, 102.77, 63.80, 33.50, 33.15. MS (ESI) m/z: 230.1 $[M+H]^+$. HRMS (ESI) m/z for $C_{14}H_{16}NO_2$ $[M+H]^+$: calc'd: 230.1181; found: 230.1178.

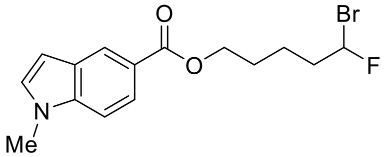

5-Bromo-5-fluoropentyl 1-methyl-1*H*-indole-5-carboxylate (23a). General procedure 4 with but-3-en-1-yl 1-methyl-1*H*-indole-5-carboxylate followed by flash chromatography (ethyl acetate/*n*-hexane, 1: 10) gave **23a** as a colorless oil (39 mg, 28% yield).

^{1}H NMR (400 MHz, $CDCl_3$) δ (ppm): 8.39 (s, 1H), 7.93 (d, J = 8.7 Hz, 1H), 7.33 (d, J = 8.7 Hz, 1H), 7.11 (d, J = 2.7 Hz, 1H), 6.73–6.31 (m, 2H), 4.36 (t, J = 6.3 Hz, 2H), 3.82 (s, 3H), 2.70–2.11 (m, 2H), 2.11–1.81 (m, 2H), 1.74 (dt, J = 13.4, 6.6 Hz, 2H); ^{19}F NMR (376 MHz, $CDCl_3$) δ (ppm):

–117.81 to –146.61 (m, 1F); ^{13}C NMR (101 MHz, $CDCl_3$) δ (ppm): 167.92, 139.33, 130.47, 128.16, 124.07, 123.09, 121.57, 109.06, 102.83, 95.43 (d, J = 252.4 Hz), 64.13, 40.38 (d, J = 19.1 Hz), 33.23, 28.18, 22.08 (d, J = 4.2 Hz). MS (ESI) m/z: 342.0 $[M+H]^+$. HRMS (ESI) m/z for $C_{15}H_{18}NO_2F^{79}Br$ $[M+H]^+$: calc'd: 342.0505; found: 342.0505.

5,5-Difluoropentyl 1-methyl-1*H*-indole-5-carboxylate (23). General procedure 6 with but-3-en-1-yl 1-methyl-1*H*-indole-5-carboxylate followed by flash chromatography (ethyl acetate/*n*-hexane, 1: 10) gave **23** as a colorless oil (62 mg, 74% yield).

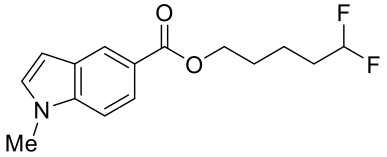

^{1}H NMR (400 MHz, $CDCl_3$) δ (ppm): 8.40 (s, 1H), 7.93 (d, J = 8.7 Hz, 1H), 7.33 (d, J = 8.7 Hz, 1H), 7.11 (d, J = 2.6 Hz, 1H), 6.60 (d, J = 2.7 Hz, 1H), 5.85 (tt, J = 56.8, 4.2 Hz, 1H), 4.36 (t, J = 6.3 Hz, 2H), 3.81 (s, 3H), 2.11–1.78 (m, 4H), 1.78–1.53 (m, 2H); ^{19}F NMR (376 MHz, $CDCl_3$) δ (ppm): –115.89 (dt, J = 56.6, 17.5 Hz, 2F); ^{13}C NMR (101 MHz, $CDCl_3$) δ (ppm): 167.91, 139.30, 130.46, 128.15, 124.02, 123.06, 121.58, 117.37 (t, J = 238.9 Hz), 109.04, 102.80, 64.16, 33.92 (t, J = 20.9 Hz), 33.18, 28.54, 19.13 (t, J = 5.6 Hz). MS (ESI) m/z: 282.1 $[M+H]^+$. HRMS (ESI) m/z for $C_{15}H_{18}NO_2F_2$ $[M+H]^+$: calc'd: 282.1306; found: 282.1309.

((3-Bromo-3-fluoropropoxy)methyl)benzene (24a). General procedure 1 followed by flash chromatography (ethyl acetate/*n*-hexane, 1: 20) gave **24a** as a colorless oil (187 mg, 38% yield).

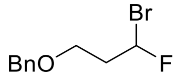

^{1}H NMR (400 MHz, $CDCl_3$) δ (ppm): 7.54–7.29 (m, 5H), 6.66 (dt, J = 50.4, 5.8 Hz, 1H), 4.53 (s, 2H), 3.84–3.37 (m, 2H), 2.90–2.14 (m, 2H); ^{19}F NMR (376 MHz, $CDCl_3$) δ (ppm):–134.14 (ddd, J = 50.4, 21.7, 12.7 Hz, 1F); ^{13}C NMR (101 MHz, $CDCl_3$) δ (ppm): 137.93, 128.60, 127.95, 127.79, 93.52 (d, J = 250.9 Hz), 73.40, 65.79 (d, J = 5.6 Hz), 41.22 (d, J = 19.5 Hz). MS (EI) m/z: 246.0 $[M]^+$. HRMS (EI) m/z for $C_{10}H_{12}OFBr$ $[M]^+$: calc'd: 246.0056; found: 246.0051.

((3,3-Difluoropropoxy)methyl)benzene (24). General procedure 2 followed by flash chromatography (ethyl acetate/*n*-hexane, 1: 20) gave **24** as a colorless oil (202 mg, 54% yield).

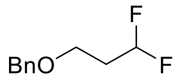

^{1}H NMR (400 MHz, $CDCl_3$) δ (ppm): 7.86–7.05 (m, 5H), 6.02 (tt, J = 56.9, 4.8 Hz, 1H), 4.53 (s, 2H), 3.64 (t, J = 6.1 Hz, 2H), 2.50–1.85 (m, 2H); ^{19}F NMR (376 MHz, $CDCl_3$) δ (ppm): –117.80 (dt, J = 56.3, 16.6 Hz, 2F); ^{13}C NMR (101 MHz, $CDCl_3$) δ (ppm): 138.08, 128.67, 127.99, 127.82, 116.14 (t, J = 238.3 Hz), 73.44, 64.21 (t, J = 6.9 Hz), 34.99 (t, J = 21.4 Hz). [Known compound[15]].

1-((5-Bromo-5-fluoropentyl)oxy)naphthalene (25a). General procedure 4 with 1-(but-3-en-1-yloxy)naphthalene followed by flash chromatography (ethyl acetate/*n*-hexane, 1: 20) gave **25a** as a colorless oil (31 mg, 25% yield).

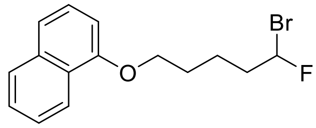

^{1}H NMR (400 MHz, $CDCl_3$) δ (ppm): 8.28 (dd, J = 6.0, 3.4 Hz, 1H), 7.82 (dd, J = 6.0, 3.2 Hz, 1H), 7.60–7.29 (m, 4H), 6.81 (d, J = 7.5 Hz, 1H), 6.53 (dt, J = 50.3, 5.3 Hz, 1H), 4.17 (t, J = 6.1 Hz, 2H), 2.53–2.15 (m, 2H), 2.14–1.92 (m, 2H), 1.91–1.73 (m, 2H); ^{19}F NMR (376 MHz, $CDCl_3$) δ (ppm): –125.90 to –136.71 (m, 1F); ^{13}C NMR (101 MHz, $CDCl_3$) δ (ppm): 154.73, 134.64, 127.61, 126.54, 125.98, 125.78, 125.33, 122.07, 120.39, 104.68, 95.45 (d, J = 252.4 Hz), 67.57, 40.43 (d, J = 19.0 Hz), 28.53, 22.25 (d, J = 3.9 Hz). MS (EI) *m*/*z*: 310.0 [M]$^+$. HRMS (EI) *m*/*z* for $C_{15}H_{16}OFBr$ [M]$^+$: calc'd: 310.0369; found: 310.0362.

1-((5,5-Difluoropentyl)oxy)naphthalene (25). General procedure 6 with 1-(but-3-en-1-yloxy)naphthalene followed by flash chromatography (ethyl acetate/*n*-hexane, 1: 20) gave **25** as a colorless oil (51 mg, 68% yield).

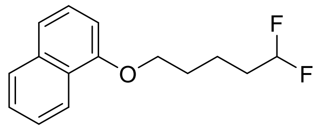

^{1}H NMR (400 MHz, $CDCl_3$) δ (ppm): 8.29 (dd, J = 7.7, 4.9 Hz, 1H), 8.05–7.72 (m, 1H), 7.59–7.33 (m, 4H), 6.81 (d, J = 7.5 Hz, 1H), 5.88 (tt, J = 56.8, 4.4 Hz, 1H), 4.17 (t, J = 6.1 Hz, 2H), 2.19–1.88 (m, 4H), 1.87–1.67 (m, 2H); ^{19}F NMR (376 MHz, $CDCl_3$) δ (ppm): –115.80 (dt, J = 35.2, 17.4 Hz, 2F); ^{13}C NMR (101 MHz, $CDCl_3$) δ (ppm): 154.75, 134.65, 127.61, 126.53, 125.99, 125.79, 125.31, 122.07, 120.36, 117.39 (t, J = 238.9 Hz), 104.66, 67.58, 33.96 (t, J = 20.9 Hz), 28.89, 19.30 (t, J = 5.6 Hz). MS (EI) *m*/*z*: 250.1 [M]$^+$. HRMS (EI) *m*/z for $C_{15}H_{16}OF_2$ [M]$^+$: calc'd: 250.1169; found: 250.1171.

2-(5-Bromo-5-fluoropentyl)isoindoline-1,3-dione (26a). General procedure 4 with 2-(but-3-en-1-yl)isoindoline-1,3-dione followed by flash chromatography (ethyl acetate/*n*-hexane, 1: 10) gave **26a** as a colorless oil (101 mg, 80% yield).

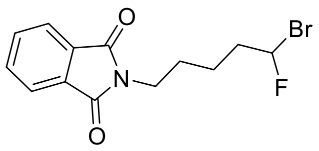

^{1}H NMR (400 MHz, $CDCl_3$) δ (ppm): 7.83 (dd, J = 8.3, 4.2 Hz, 2H), 7.72 (dd, J = 8.0, 4.0 Hz, 2H), 6.44 (dt, J = 50.3, 5.3 Hz, 1H), 3.70 (t, J = 7.1 Hz, 2H), 2.51–2.05 (m, 2H), 1.85–1.67 (m, 2H), 1.64–1.47 (m, 2H); ^{19}F NMR (376 MHz, $CDCl_3$) δ (ppm): –130.97 (ddd, J = 50.4, 19.9, 18.2 Hz, 1F); ^{13}C NMR (101 MHz, $CDCl_3$) δ (ppm): 168.56, 134.19, 132.25, 123.46, 95.22 (d, J = 252.4

Hz), 40.15 (d, *J* = 19.1 Hz), 37.64, 27.85, 22.53 (d, *J* = 3.9 Hz). MS (EI) *m*/*z*: 313.0 [*M*]$^+$. HRMS (EI) *m*/*z* for $C_{13}H_{13}FO_2BrN$ [*M*]$^+$: calc'd: 313.0114; found: 313.0118.

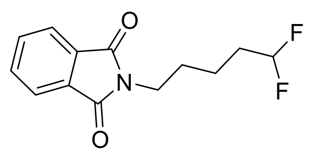

2-(5,5-Difluoropentyl)isoindoline-1,3-dione (26). General procedure 6 with isoindoline-1,3-dione followed by flash chromatography (ethyl acetate/*n*-hexane, 1: 10) gave **26** as a white solid (46 mg, 61% yield). Mp: 74–76 °C.

^{1}H NMR (400 MHz, $CDCl_3$) δ (ppm): 7.83 (dd, *J* = 8.0, 3.7 Hz, 2H), 7.72 (dd, *J* = 7.7, 4.2 Hz, 2H), 5.79 (tt, *J* = 56.8, 4.2 Hz, 1H), 3.70 (t, *J* = 7.1 Hz, 2H), 2.06–1.79 (m, 2H), 1.80–1.67 (m, 2H), 1.59–1.39 (m, 2H); ^{19}F NMR (376 MHz, $CDCl_3$) δ (ppm): –115.97 (dt, *J* = 56.5, 17.6 Hz, 2F); ^{13}C NMR (101 MHz, $CDCl_3$) δ (ppm): 168.58, 134.18, 132.25, 123.45, 117.22 (t, *J* = 239.1 Hz), 37.68, 33.73 (t, *J* = 21.1 Hz), 28.23, 19.63 (t, *J* = 5.6 Hz). [Known compound[16]].

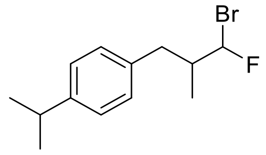

1-(3-Bromo-3-fluoro-2-methylpropyl)-4-isopropylbenzene (27a). General procedure 1 with 3-(4-isopropylphenyl)-2-methylpropanal followed by flash chromatography (*n*-hexane) gave **27a** as a colorless oil (132 mg, d.r. = 1/1, 24% yield).

^{1}H NMR (400 MHz, $CDCl_3$) δ (ppm): 7.21 (d, *J* = 7.9 Hz, 2H), 7.14 (dd, *J* = 7.7, 3.9 Hz, 2H), 6.40 (ddd, *J* = 49.4, 33.5, 3.0 Hz, 1H), 2.99–2.73 (m, 2H), 2.71–2.48 (m, 1H), 2.48–2.20 (m, 1H), 1.29 (d, *J* = 6.9 Hz, 6H), 1.12 (d, *J* = 6.6 Hz, 3H); ^{19}F NMR (376 MHz, $CDCl_3$) δ (ppm): –134.62 to –140.53 (m, 1F); ^{13}C NMR (101 MHz, $CDCl_3$) δ (ppm): 147.32, 147.24, 136.19, 135.91, 129.29, 129.17, 126.82, 126.80, 101.46 (d, *J* = 254.6 Hz), 99.82 (d, *J* = 256.0 Hz), 44.29 (d, *J* = 18.1 Hz), 43.29 (d, *J* = 17.0 Hz), 38.27 (d, *J* = 16.2 Hz), 38.23 (d, *J* = 11.5 Hz), 33.93, 24.23, 14.76 (d, *J* = 2.1 Hz), 14.44 (d, *J* = 5.1 Hz). MS (EI) *m*/*z*: 272.0 [*M*]$^+$. HRMS (EI) *m*/*z* for $C_{13}H_{18}FBr$ [*M*]$^+$: calc'd: 272.0576; found: 272.0566.

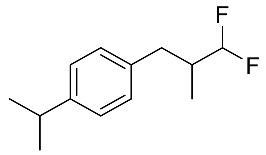

1-(3,3-Difluoro-2-methylpropyl)-4-isopropylbenzene (27). General procedure 2 with 3-(4-isopropylphenyl)-2-methylpropanal followed by flash chromatography (*n*-hexane) gave **27** as a colorless oil (234 mg, 55% yield).

^{1}H NMR (400 MHz, $CDCl_3$) δ (ppm): 7.18 (d, *J* = 7.3 Hz, 2H), 7.11 (d, *J* = 7.4 Hz, 2H), 5.66 (td, *J* = 56.8, 2.8 Hz, 1H), 2.89 (ddd, *J* = 19.7, 13.6, 6.3 Hz, 2H), 2.48 (dd, *J* = 13.5, 9.1 Hz, 1H), 2.31–2.05 (m, 1H), 1.26 (d, *J* = 6.8 Hz, 6H), 1.00 (d, *J* = 6.8 Hz, 3H); ^{19}F NMR (376 MHz, $CDCl_3$) δ

(ppm):–125.04 (ddd, *J* = 276.8, 75.1, 56.8, 15.1 Hz, 1F); ^{13}C NMR (101 MHz, $CDCl_3$) δ (ppm): 147.17, 136.22, 129.27, 126.75, 118.84 (t, *J* = 242.0 Hz), 39.49 (t, *J* = 19.5 Hz), 36.08 (dd, *J* = 5.9, 3.8 Hz), 33.93, 24.23, 12.12 (t, *J* = 4.9 Hz). MS (EI) *m/z*: 212.1 [*M*]$^+$. HRMS (EI) *m/z* for $C_{13}H_{18}F_2$ [*M*]$^+$: calc'd: 212.1377; found: 212.1379.

5-(3-Bromo-3-fluoro-2-methylpropyl)benzo[*d*][1,3]dioxole (28a). General procedure 1 with 3-(benzo[*d*][1,3]dioxol-5-yl)-2-methylpropanal followed by flash chromatography (ethyl acetate/*n*-hexane, 1: 20) gave **28a** as a colorless oil (156 mg, d.r. = 5/9, 28% yield).

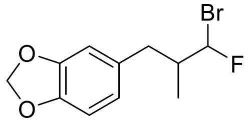

^{1}H NMR (400 MHz, $CDCl_3$) δ (ppm): 6.75 (d, *J* = 7.9 Hz, 1H), 6.72–6.59 (m, 2H), 6.47–6.25 (m, 1H), 5.94 (s, 2H), 2.89–2.70 (m, 1H), 2.51–2.45 (m, 1H), 2.34–2.23 (m, 1H), 1.07 (d, *J* = 6.7 Hz, 3H); ^{19}F NMR (376 MHz, $CDCl_3$) δ (ppm): –137.75 (ddd, *J* = 69.6, 49.6, 15.8 Hz, 1F); ^{13}C NMR (101 MHz, $CDCl_3$) δ (ppm): 147.98, 146.41, 146.36, 132.63, 132.32, 122.31, 122.19, 109.61, 109.49, 108.51, 108.49, 101.14, 103.02–98.13 (m), 44.38 (d, *J* = 17.9 Hz), 43.42 (d, *J* = 17.2 Hz), 38.40 (d, *J* = 6.4 Hz), 38.27 (d, *J* = 1.5 Hz), 14.67 (d, *J* = 2.1 Hz), 14.35 (d, *J* = 5.1 Hz). MS (EI) *m/z*: 274.0 [*M*]$^+$. HRMS (EI) *m/z* for $C_{11}H_{12}FO_2Br$ [*M*]$^+$: calc'd: 274.0005; found: 273.9999.

5-(3,3-Difluoro-2-methylpropyl)benzo[*d*][1,3]dioxole (28). General procedure 2 with 3-(benzo[*d*][1,3]dioxol-5-yl)-2-methylpropanal followed by flash chromatography (ethyl acetate/*n*-hexane, 1: 20) gave **28** as a colorless oil (201 mg, 47% yield).

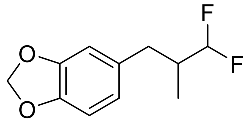

^{1}H NMR (400 MHz, $CDCl_3$) δ (ppm): 6.75 (d, *J* = 7.8 Hz, 1H), 6.72–6.55 (m, 2H), 5.94 (s, 2H), 5.64 (td, *J* = 56.8, 2.9 Hz, 1H), 2.81 (dd, *J* = 13.7, 5.7 Hz, 1H), 2.42 (dd, *J* = 13.7, 9.1 Hz, 1H), 2.24–1.98 (m, 1H), 0.97 (d, *J* = 6.9 Hz, 3H); ^{19}F NMR (376 MHz, $CDCl_3$) δ (ppm): –124.99 (ddd, *J* = 277.1, 74.4, 56.6, 15.1 Hz, 2F); ^{13}C NMR (101 MHz, $CDCl_3$) δ (ppm): 147.97, 146.33, 132.67, 122.27, 118.76 (t, *J* = 242.1 Hz), 109.60, 108.46, 101.14, 39.62 (t, *J* = 19.5 Hz), 36.19 (dd, *J* = 5.9, 3.8 Hz), 12.07 (t, *J* = 4.8 Hz). MS (EI) *m/z*: 214.1 [*M*]$^+$. HRMS (EI) *m/z* for $C_{11}H_{12}F_2O_2$ [*M*]$^+$: calc'd: 214.0805; found: 214.0799.

2-(Bromofluoromethyl)-2,3-dihydro-1*H*-indene (29a). General procedure 1 with 2,3-dihydro-1*H*-indene-2-carbaldehyde followed by flash chromatography (*n*-hexane) gave **29a** as a colorless oil (167 mg, 36% yield).

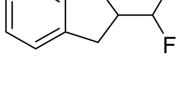

^{1}H NMR (400 MHz, $CDCl_3$) *δ* (ppm): 7.66–6.79 (m, 4H), 6.44 (dd, *J* = 50.2, 5.5 Hz, 1H), 3.29–3.06 (m, 3H), 3.06–2.78 (m, 2H); ^{19}F NMR (376 MHz, $CDCl_3$) *δ* (ppm): –132.96 (dd, *J* = 50.3, 13.4 Hz, 1F); ^{13}C NMR (101 MHz, $CDCl_3$) *δ* (ppm): 141.31 (d, *J* = 12.9 Hz), 126.94 (d, *J* = 2.2 Hz), 124.74 (d, *J* = 9.6 Hz), 98.38 (d, *J* = 254.1 Hz), 48.39 (d, *J* = 18.3 Hz), 35.55 (dd, *J* = 48.8, 3.9 Hz). MS (EI) *m/z*: 228.0 $[M]^+$. HRMS (EI) *m/z* for $C_{10}H_{10}FBr$ $[M]^+$: calc'd: 227.9950; found: 227.9945.

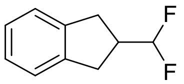

2-(Difluoromethyl)-2,3-dihydro-1*H*-indene (29). General procedure 2 with 2,3-dihydro-1*H*-indene-2-carbaldehyde followed by flash chromatography (*n*-hexane) gave **29** as a colorless oil (205 mg, 61% yield).

^{1}H NMR (400 MHz, $CDCl_3$) *δ* (ppm): 7.19 (dt, *J* = 20.8, 3.9 Hz, 4H), 5.77 (td, *J* = 56.8, 4.9 Hz, 1H), 3.11 (dd, *J* = 16.0, 8.6 Hz, 2H), 3.04–2.79 (m, 3H); ^{19}F NMR (376 MHz, $CDCl_3$) *δ* (ppm): –119.79 (dd, *J* = 56.4, 13.3 Hz, 2F); ^{13}C NMR (101 MHz, $CDCl_3$) δ (ppm): 141.48, 126.94, 124.76, 118.62 (t, *J* = 240.7 Hz), 42.81 (t, *J* = 20.3 Hz), 32.98 (t, *J* = 5.0 Hz). [Known compound[11]].

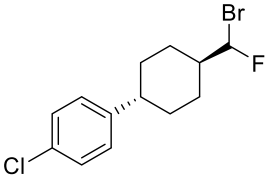

1-((1*S*,4*R*)-4-((*S*)-Bromofluoromethyl)cyclohexyl)-4-chlorobenzene (30a). General procedure 1 with (1*r*,4*r*)-4-(4-chlorophenyl)cyclohexane-1-carbaldehyde followed by flash chromatography (*n*-hexane) gave **30a** as a colorless oil (181 mg, 30% yield).

^{1}H NMR (400 MHz, $CDCl_3$) *δ* (ppm): 7.30 (d, *J* = 7.7 Hz, 2H), 7.16 (d, *J* = 7.7 Hz, 2H), 6.39 (dd, *J* = 50.0, 4.1 Hz, 1H), 2.52 (t, *J* = 11.6 Hz, 1H), 2.40–1.84 (m, 5H), 1.77–1.24 (m, 4H); ^{19}F NMR (376 MHz, $CDCl_3$) *δ* (ppm): –135.92 (dd, *J* = 49.9, 14.2 Hz, 1F); ^{13}C NMR (101 MHz, $CDCl_3$) *δ* (ppm): 145.19, 131.91, 128.69, 128.30, 100.26 (d, *J* = 255.1 Hz), 46.04 (d, *J* = 17.5 Hz), 43.47, 33.11 (d, *J* = 5.5 Hz), 28.11 (dd, *J* = 54.5, 3.6 Hz). MS (EI) *m/z*: 304.0 $[M]^+$. HRMS (EI) *m/z* for $C_{13}H_{15}FClBr$ $[M]^+$: calc'd: 304.0030; found: 304.0039.

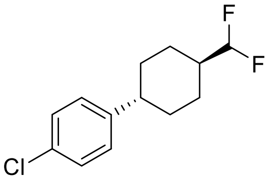

1-Chloro-4-((1*R*,4*R*)-4-(difluoromethyl)cyclohexyl)benzene (30). General procedure 2 with (1*R*,4*R*)-4-(4-chlorophenyl)cyclohexane-1-carbaldehyde followed by flash chromatography (*n*-hexane) gave **30** as a white solid (256 mg, 52% yield). Mp: 40–42 °C.

^{1}H NMR (400 MHz, $CDCl_3$) *δ* (ppm): 7.29 (d, *J* = 7.5 Hz, 2H), 7.16 (d, *J* = 7.6 Hz, 2H), 5.63 (td, *J* = 56.9, 4.2 Hz, 1H), 2.50 (t, *J* = 11.8 Hz, 1H), 2.00 (d, *J* = 10.7 Hz, 4H), 1.94–1.68 (m, 1H), 1.55–1.08 (m, 4H); ^{19}F NMR (376 MHz, $CDCl_3$) *δ* (ppm): –122.98 (dd, *J* = 57.1, 13.6 Hz, 2F); ^{13}C

NMR (101 MHz, $CDCl_3$) δ (ppm): 145.39, 131.91, 128.70, 128.30, 119.20 (t, J = 241.6 Hz), 43.49, 41.37 (t, J = 19.5 Hz), 32.97, 25.65 (t, J = 4.7 Hz). MS (EI) m/z: 244.1 $[M]^+$. HRMS (EI) m/z for $C_{13}H_{15}F_2Cl$ $[M]^+$: calc'd: 244.0830; found: 244.0836.

2-(Bromofluoromethyl)-3-methylbutyl benzoate (31a). General procedure 4 with 3-methylbut-2-en-1-yl benzoate followed by flash chromatography (ethyl acetate/*n*-hexane, 1: 20) gave **31a** as a colorless oil (56 mg, d.r. = 1:1, 46% yield).

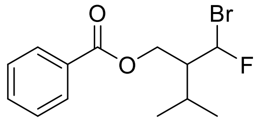

^1H NMR (400 MHz, $CDCl_3$) δ (ppm): 8.04 (d, J = 8.1 Hz, 2H), 7.58 (t, J = 7.4 Hz, 1H), 7.45 (t, J = 7.7 Hz, 2H), 6.74 (ddd, J = 49.2, 21.2, 3.8 Hz, 1H), 4.82–4.40 (m, 2H), 2.46–1.99 (m, 2H), 1.27–0.91 (m, 6H); ^{19}F NMR (376 MHz, $CDCl_3$) δ (ppm): –138.04 (ddd, J = 70.1, 49.2, 18.2 Hz, 1F); ^{13}C NMR (101 MHz, $CDCl_3$) δ (ppm): 166.47, 133.36, 130.09, 129.84, 128.67, 96.86 (dd, J = 256.0, 22.2 Hz), 62.57 (d, J = 3.5 Hz), 62.22 (d, J = 5.8 Hz), 52.18 (d, J = 16.3 Hz), 51.59 (d, J = 16.1 Hz), 28.17, 27.65 (d, J = 3.7 Hz), 21.33, 21.02, 19.98, 19.51. [Known compound[3]].

2-(Difluoromethyl)-3-methylbutyl benzoate (31). General procedure 7 with 3-methylbut-2-en-1-yl benzoate followed by flash chromatography (ethyl acetate/*n*-hexane, 1: 20) gave **31** as a colorless oil (23 mg, 24% yield).

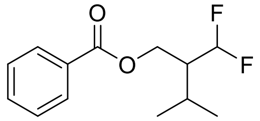

^1H NMR (400 MHz, $CDCl_3$) δ 8.17–7.95 (m, 2H), 7.57 (t, J = 7.4 Hz, 1H), 7.45 (t, J = 7.7 Hz, 2H), 6.01 (td, J = 55.9, 3.6 Hz, 1H), 4.68–4.26 (m, 2H), 2.41–1.94 (m, 2H), 1.09 (dd, J = 13.7, 6.5 Hz, 6H); ^{19}F NMR (376 MHz, $CDCl_3$) δ –117.81 to – 128.06 (m, 2F); ^{13}C NMR (101 MHz, $CDCl_3$) δ 166.55, 133.34, 130.14, 129.82, 128.66, 117.44 (t, J = 243.0 Hz), 60.93 (t, J = 5.6 Hz), 47.66 (t, J = 18.1 Hz), 26.88–24.05, 20.83 (s), 19.98. [Known compound[16]].

2-Bromo-9-(4-methylpent-3-en-1-yl)-9*H*-carbazole (32b). To a dried round-bottomed flask equipped with a stirrer bar were added 2-bromo-9*H*-carbazole (1.23 g, 5.0 mmol, 1.0 equiv.), K_2CO_3 (1.38 g, 10 mmol, 2.0 equiv.) and DMF (30 mL). 5-Bromo-2-methylpent-2-ene (1.63 g, 10 mmol, 2.0 equiv.) was then added. The reaction mixture was stirred for 12 h at 50 °C and then water (50 mL) and DCM (50 mL) were added. The organic phase was washed with brine, dried (Mg_2SO_4), and concentrated in vacuo. The residue was purified with silica gel chromatography (*n*-hexane) to give **32b** as a white solid (1.21 g, 74% yield). Mp: 56–58 °C.

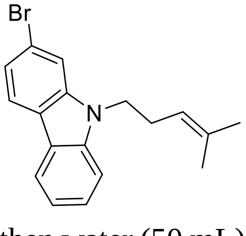

^{1}H NMR (400 MHz, $CDCl_3$) δ (ppm): 8.08 (d, J = 7.8 Hz, 1H), 7.96 (d, J = 8.2 Hz, 1H), 7.59 (d, J = 1.4 Hz, 1H), 7.52 (t, J = 7.6 Hz, 1H), 7.43 (d, J = 8.2 Hz, 1H), 7.36 (dd, J = 8.2, 1.5 Hz, 1H), 7.28 (dd, J = 9.2, 5.6 Hz, 1H), 5.23 (t, J = 7.4 Hz, 1H), 4.26 (t, J = 7.4 Hz, 2H), 2.55 (q, J = 7.3 Hz, 2H), 1.67 (s, 3H), 1.47 (s, 3H); ^{13}C NMR (101 MHz, $CDCl_3$) δ 141.38, 140.68, 135.33, 126.19, 122.52, 122.09, 121.98, 121.61, 120.50, 120.26, 119.51, 119.33, 112.07, 109.10, 43.25, 27.76, 25.87, 17.80. MS (ESI) *m/z*: 328.1 [*M*+H]$^+$. HRMS (ESI) *m/z* for $C_{18}H_{19}N^{79}Br$ [*M*+H]$^+$: calc'd: 328.0701; found: 328.0696.

2-Bromo-9-(3-(bromofluoromethyl)-4-methylpentyl)-9*H*-carbazole (32a). General procedure 4 with 2-bromo-9-(4-methylpent-3-en-1-yl)-9*H*-carbazole followed by flash chromatography (*n*-hexane) gave **32a** as a colorless oil (76 mg, d.r. = 1/1, 43% yield).

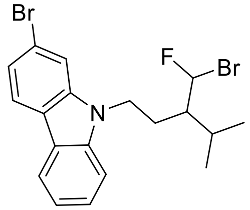

^{1}H NMR (400 MHz, $CDCl_3$) δ (ppm): 8.04 (d, J = 7.8 Hz, 1H), 7.91 (d, J = 8.2 Hz, 1H), 7.55 (s, 1H), 7.49 (t, J = 7.6 Hz, 1H), 7.41 (d, J = 8.2 Hz, 1H), 7.32 (d, J = 8.2 Hz, 1H), 7.24 (t, J = 7.4 Hz, 1H), 6.62 (ddd, J = 49.3, 11.0, 4.0 Hz, 1H), 4.46–4.22 (m, 2H), 2.48–1.67 (m, 4H), 1.10–0.62 (m, 6H); ^{19}F NMR (376 MHz, $CDCl_3$) δ (ppm): –134.56 (ddd, J = 73.9, 49.8, 17.2 Hz, 1F); ^{13}C NMR (101 MHz, $CDCl_3$) δ (ppm): 141.10 (d, J = 3.0 Hz), 140.43 (d, J = 1.8 Hz), 126.46, 122.62 (d, J = 1.5 Hz), 122.35 (d, J = 2.4 Hz), 122.10 (d, J = 1.2 Hz), 121.77, 120.64, 119.74 (d, J = 2.1 Hz), 119.58, 111.87, 108.97 (d, J = 4.0 Hz), 99.67 (dd, J = 254.9, 211.5 Hz), 51.35 (d, J = 16.5 Hz), 50.00 (d, J = 14.7 Hz), 43.47–41.78 (m), 29.88 (d, J = 6.7 Hz), 27.23, 25.87 (d, J = 2.6 Hz), 20.45 (d, J = 3.8 Hz), 19.47, 19.01. MS (ESI) *m/z*: 440.0 [*M*+H]$^+$. HRMS (ESI) *m/z* for $C_{19}H_{21}NF^{79}Br_2$ [*M*+H]$^+$: calc'd: 440.0025; found: 440.0022.

2-Bromo-9-(3-(difluoromethyl)-4-methylpentyl)-9*H*-carbazole (32). General procedure 7 with 2-bromo-9-(4-methylpent-3-en-1-yl)-9*H*-carbazole followed by flash chromatography (*n*-hexane) gave **32** as a colorless oil (63 mg, 43% yield).

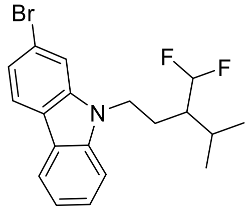

^{1}H NMR (400 MHz, $CDCl_3$) δ (ppm): 7.97 (d, J = 7.8 Hz, 1H), 7.84 (d, J = 8.3 Hz, 1H), 7.47–7.37 (m, 2H), 7.31 (d, J = 8.2 Hz, 1H), 7.25 (dd, J = 8.2, 1.5 Hz, 1H), 7.17 (t, J = 7.4 Hz, 1H), 5.81 (td, J = 56.4, 3.9 Hz, 1H), 4.58–3.91 (m, 2H), 2.07–1.60 (m, 4H), 0.82 (dd, J = 6.8, 3.8 Hz, 6H); ^{19}F NMR (376 MHz, $CDCl_3$) δ (ppm): –119.32 (ddd, J = 282.8, 78.4, 56.4, 16.1 Hz, 2F); ^{13}C NMR (101 MHz, $CDCl_3$) δ (ppm): 141.12, 140.45, 126.45, 122.61, 122.34, 122.10, 121.77, 120.63, 119.73, 119.57, 119.05 (t, J = 242.5 Hz), 111.77, 108.92, 45.84 (t, J = 17.8 Hz), 42.34, 28.78–

26.93 (m), 23.78, 20.11, 19.08. MS (ESI) *m/z*: 380.1 $[M+H]^+$. HRMS (ESI) *m/z* for $C_{19}H_{21}NF_2{}^{19}Br$ $[M+H]^+$: calc'd: 380.0825; found: 380.0827.

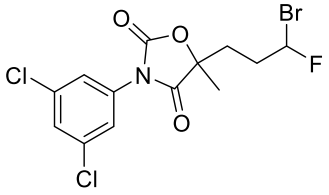

5-(3-Bromo-3-fluoropropyl)-3-(3,5-dichlorophenyl)-5-methyloxazolidine-2,4-dione (33a). General procedure 4 with vinclozolin [3-(3,5-dichlorophenyl)-5-methyl-5-vinyloxazolidine-2,4-dione] followed by flash chromatography (ethyl acetate/*n*-hexane, 1: 10) gave **33a** as a white solid (103 mg, d.r. = 1/1, 64% yield). Mp: 85–87 °C.

^{1}H NMR (400 MHz, $CDCl_3$) δ (ppm): 7.44 (dd, J = 1.7, 0.5 Hz, 2H), 7.43–7.40 (m, 1H), 6.67–6.33 (m, 1H), 2.56–2.02 (m, 4H), 1.70 (s, 3H); ^{19}F NMR (376 MHz, $CDCl_3$) δ (ppm):–125.27 to –150.93 (m, 1F); ^{13}C NMR (101 MHz, $CDCl_3$) δ (ppm): 173.20, 152.16, 135.80, 132.59, 129.33, 123.89, 93.26 (dd, J = 252.7, 4.2 Hz), 84.89, 34.10 (d, J = 20.4 Hz), 32.04 (dd, J = 7.6, 3.9 Hz), 22.49 (d, J = 3.2 Hz). [Known compound[3]].

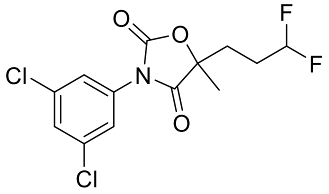

3-(3,5-Dichlorophenyl)-5-(3,3-difluoropropyl)-5-methyloxazolidine-2,4-dione (33). General procedure 5 with **Vinclozolin** followed by flash chromatography (ethyl acetate/*n*-hexane, 1: 10) gave **33** as a white solid (46 mg, 46% yield). Mp: 83–86 °C.

^{1}H NMR (400 MHz, $CDCl_3$) δ (ppm): 7.53–7.34 (m, 3H), 5.89 (tt, J = 56.2, 3.9 Hz, 1H), 2.51–1.82 (m, 4H), 1.69 (s, 3H); ^{19}F NMR (376 MHz, $CDCl_3$) δ (ppm): –116.86 (dt, J = 34.0, 16.7 Hz, 2F); ^{13}C NMR (101 MHz, $CDCl_3$) δ (ppm): 173.26, 152.22, 135.83, 132.61, 129.35, 123.91, 115.79 (t, J = 240.2 Hz), 84.99, 29.26 (t, J = 5.6 Hz), 28.21 (t, J = 22.3 Hz), 22.56. [Known compound[17]].

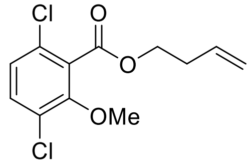

But-3-en-1-yl 3,6-dichloro-2-methoxybenzoate (34b). General procedure 3 with dicamba (3,6-dichloro-2-methoxybenzoic acid) followed by flash chromatography (ethyl acetate/*n*-hexane, 1: 10) gave **34b** as a colorless oil (602 mg, 44% yield).

^{1}H NMR (400 MHz, $CDCl_3$) δ (ppm): 7.34 (d, J = 8.6 Hz, 1H), 7.11 (d, J = 8.7 Hz, 1H), 5.85 (ddt, J = 17.0, 10.3, 6.7 Hz, 1H), 5.14 (dd, J = 23.3, 13.7 Hz, 2H), 4.43 (t, J = 6.8 Hz, 2H), 3.90 (s, 3H), 2.53 (q, J = 6.7 Hz, 2H); ^{13}C NMR (101 MHz, $CDCl_3$) δ (ppm): 164.76, 154.00, 133.75, 131.95, 130.74, 129.83, 126.93, 126.03, 117.80, 65.50, 62.47, 33.07. MS (EI) *m/z*: 274.0 $[M]^+$. HRMS (EI) *m/z* for $C_{12}H_{12}O_3Cl_2$ $[M]^+$: calc'd: 274.0164; found: 274.0158.

5-Bromo-5-fluoropentyl 3,6-dichloro-2-methoxybenzoate (34a). General procedure 4 with but-3-en-1-yl 3,6-dichloro-2-methoxybenzoate followed by flash chromatography (ethyl acetate/*n*-hexane, 1: 10) gave **34a** as a colorless oil (21 mg, 14% yield).

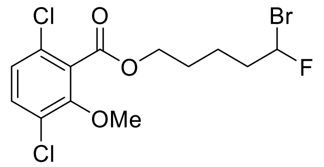

^{1}H NMR (400 MHz, $CDCl_3$) δ (ppm): 7.36 (d, J = 8.7 Hz, 1H), 7.12 (d, J = 8.6 Hz, 1H), 6.47 (dt, J = 50.3, 5.2 Hz, 1H), 4.41 (t, J = 6.3 Hz, 2H), 3.91 (s, 3H), 2.44–2.11 (m, 2H), 2.00–1.75 (m, 2H), 1.75–1.62 (m, 2H); ^{19}F NMR (376 MHz, $CDCl_3$) δ (ppm): –123.67 to –145.36 (m, 1F); ^{13}C NMR (101 MHz, $CDCl_3$) δ (ppm): 164.79, 153.98, 132.02, 130.72, 129.74, 126.96, 126.06, 95.20 (d, J = 252.5 Hz), 65.71, 62.50, 40.21 (d, J = 19.2 Hz), 27.91, 21.79 (d, J = 4.1 Hz). MS (EI) *m/z*: 385.9 $[M]^+$. HRMS (EI) *m/z* for $C_{13}H_{14}FO_3BrCl_2$ $[M]^+$: calc'd: 385.9487; found: 385.9500.

5,5-Difluoropentyl 3,6-dichloro-2-methoxybenzoate (34). General procedure 5 with but-3-en-1-yl 3,6-dichloro-2-methoxybenzoate followed by flash chromatography (ethyl acetate/*n*-hexane, 1: 10) gave **34** as a colorless oil (63 mg, 64% yield).

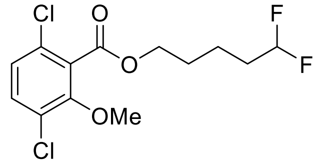

^{1}H NMR (400 MHz, $CDCl_3$) δ (ppm): 7.35 (d, J = 8.7 Hz, 1H), 7.11 (d, J = 8.7 Hz, 1H), 5.82 (tt, J = 56.7, 4.3 Hz, 1H), 4.40 (t, J = 6.4 Hz, 2H), 3.90 (s, 3H), 2.05–1.74 (m, 4H), 1.72–1.52 (m, 2H); ^{19}F NMR (376 MHz, $CDCl_3$) δ (ppm): –116.01 (dt, J = 56.9, 17.6 Hz, 2F); ^{13}C NMR (101 MHz, $CDCl_3$) δ (ppm): 164.77, 153.97, 131.99, 130.74, 129.71, 126.94, 126.05, 117.22 (t, J = 239.0 Hz), 65.72, 62.44, 33.74 (t, J = 21.0 Hz), 28.23, 18.82 (t, J = 5.6 Hz). MS (EI) *m/z*: 326.0 $[M]^+$. HRMS (EI) *m/z* for $C_{13}H_{14}F_2O_3Cl_2$ $[M]^+$: calc'd: 326.0288; found: 326.0280.

Allyl (*S*)-2-(3-benzoylphenyl)propanoate (35b). (*S*)-Ketoprofen [(*S*)-2-(3-benzoylphenyl)propanoic acid; 763 mg, 3.0 mmol, 1.0 equiv.], K_2CO_3 (828 mg, 6.0 mmol, 2.0 equiv.), DMF (20 mL) and allyl bromide (726 mg, 6.0 mmol, 2.0 equiv.) were added under argon to the reaction vessel. The reaction vessel was capped loosely and stirred vigorously at 50 °C for 12 h. The mixture was cooled to RT, poured onto water, and extracted with DCM (2 × 10 mL). The organic phase was dried (Mg_2SO_4) and concentrated in vacuo. The residue was then purified by silica gel flash chromatography (ethyl acetate/*n*-hexane, 1: 10) to give **35b** as a colorless oil (628 mg, 71% yield).

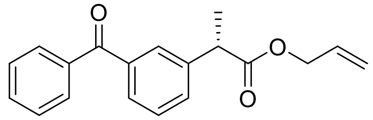

^{1}H NMR (400 MHz, $CDCl_3$) δ (ppm): 7.83–7.74 (m, 3H), 7.68 (d, J = 7.6 Hz, 1H), 7.62–7.53 (m, 2H), 7.52–7.41 (m, 3H), 5.86 (ddt, J = 16.3, 10.8, 5.6 Hz, 1H), 5.21 (t, J = 14.2 Hz, 2H), 4.94–

4.29 (m, 2H), 3.83 (q, J = 7.2 Hz, 1H), 1.55 (d, J = 7.2 Hz, 3H); ^{13}C NMR (101 MHz, $CDCl_3$) δ (ppm): 196.69, 173.91, 140.95, 138.12, 137.71, 132.71, 132.13, 131.73, 130.28, 129.47, 129.23, 128.77, 128.51, 118.42, 65.69, 45.59, 18.68. MS (ESI) m/z: 295.1 $[M+H]^+$. HRMS (ESI) m/z for $C_{19}H_{19}O_3$ $[M+H]^+$: calc'd: 295.1334; found: 295.1336.

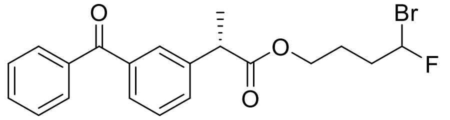

4-Bromo-4-fluorobutyl (2*S*)-2-(3-benzoylphenyl)propanoate (35a). General procedure 4 with allyl (*S*)-2-(3-benzoylphenyl)propanoate followed by flash chromatography (ethyl acetate/*n*-hexane, 1: 10) gave **35a** as a colorless oil (73 mg, d.r. = 1/1, 45% yield).

^{1}H NMR (400 MHz, $CDCl_3$) δ (ppm): 7.86–7.72 (m, 3H), 7.68 (d, J = 7.6 Hz, 1H), 7.60 (t, J = 7.3 Hz, 1H), 7.49 (tt, J = 15.6, 7.7 Hz, 4H), 6.40 (dt, J = 50.1, 5.2 Hz, 1H), 4.22–4.02 (m, 2H), 3.81 (q, J = 7.1 Hz, 1H), 2.38–1.99 (m, 2H), 1.99–1.74 (m, 2H), 1.55 (d, J = 7.2 Hz, 3H); ^{19}F NMR (376 MHz, $CDCl_3$) δ (ppm): –131.51 (ddt, J = 49.9, 28.0, 19.0 Hz, 1F); ^{13}C NMR (101 MHz, $CDCl_3$) δ (ppm): 196.62, 174.09, 140.95, 138.17, 137.67, 132.73, 131.62, 130.25, 129.36, 129.30, 128.78, 128.52, 94.75 (d, J = 252.3 Hz), 63.71, 45.57, 37.26 (d, J = 19.5 Hz), 24.43 (d, J = 5.6 Hz), 18.49. MS (ESI) m/z: 407.1 $[M+H]^+$. HRMS (ESI) m/z for $C_{20}H_{21}O_3F^{79}Br$ $[M+H]^+$: calc'd: 407.0658; found: 407.0656.

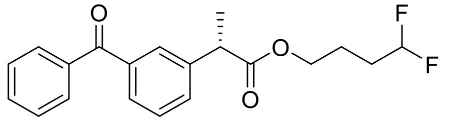

4,4-Difluorobutyl (*S*)-2-(3-benzoylphenyl)propanoate (35). General procedure 5 with allyl (*S*)-2-(3-benzoylphenyl)propanoate followed by flash chromatography (ethyl acetate/*n*-hexane, 1: 10) gave **35** as a colorless oil (52 mg, 50% yield).

^{1}H NMR (400 MHz, $CDCl_3$) δ (ppm): 7.85–7.73 (m, 1H), 7.67 (d, J = 7.6 Hz, 1H), 7.60 (t, J = 7.4 Hz, 1H), 7.57–7.41 (m, 4H), 6.13–5.39 (m, 1H), 4.12 (t, J = 5.7 Hz, 2H), 3.80 (q, J = 7.2 Hz, 1H), 2.01–1.66 (m, 4H), 1.54 (d, J = 7.2 Hz, 3H); ^{19}F NMR (376 MHz, $CDCl_3$) δ (ppm): –116.32 (dt, J = 56.6, 17.1 Hz, 2F); ^{13}C NMR (101 MHz, $CDCl_3$) δ (ppm): 196.66, 174.12, 140.97, 138.17, 137.68, 132.73, 131.62, 130.24, 129.31, 128.76, 128.52, 116.81 (t, J = 239.1 Hz), 64.02, 45.58, 30.88 (t, J = 21.4 Hz), 21.55 (t, J = 5.7 Hz), 18.52. MS (ESI) m/z: 347.1 $[M+H]^+$. HRMS (ESI) m/z for $C_{20}H_{21}O_3F_2$ $[M+H]^+$: calc'd: 347.1459; found: 347.1455.

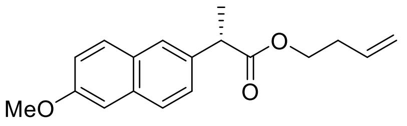

But-3-en-1-yl (*S*)-2-(6-methoxynaphthalen-2-yl)propanoate (36b). General procedure 3 with naproxen [(*S*)-2-(6-

methoxynaphthalen-2-yl)propanoic acid] followed by flash chromatography (ethyl acetate/*n*-hexane, 1: 10) gave **36b** as a white solid (1.12 g, 79% yield). Mp: 41–43 °C.

^{1}H NMR (400 MHz, $CDCl_3$) δ (ppm): 7.86–7.61 (m, 3H), 7.42 (d, *J* = 8.4 Hz, 1H), 7.24–6.99 (m, 2H), 5.71 (ddt, *J* = 17.0, 10.3, 6.8 Hz, 1H), 5.01 (dd, *J* = 13.5, 9.7 Hz, 2H), 4.14 (t, *J* = 6.7 Hz, 2H), 3.95–3.81 (m, 4H), 2.34 (q, *J* = 6.7 Hz, 2H), 1.59 (d, *J* = 7.1 Hz, 3H); ^{13}C NMR (101 MHz, $CDCl_3$) *δ* (ppm): 174.79, 157.79, 135.87, 134.05, 133.86, 129.45, 129.10, 127.25, 126.48, 126.14, 119.12, 117.38, 105.74, 63.92, 55.47, 45.66, 33.23, 18.69. MS (ESI) *m/z*: 285.1 $[M+H]^+$. HRMS (ESI) *m/z* for $C_{18}H_{21}O_3$ $[M+H]^+$: calc'd: 285.1491; found: 285.1492.

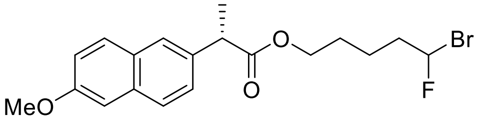

5-Bromo-5-fluoropentyl (2*S*)-2-(6-methoxynaphthalen-2-yl)propanoate (36a). General procedure 4 with but-3-en-1-yl (*S*)-2-(6-methoxynaphthalen-2-yl)propanoate followed by flash chromatography (ethyl acetate/*n*-hexane, 1: 10) gave **36a** as a colorless oil (62 mg, d.r. = 1/1, 39% yield).

^{1}H NMR (400 MHz, $CDCl_3$) *δ* (ppm): 7.81–7.51 (m, 3H), 7.40 (d, *J* = 8.4 Hz, 1H), 7.21–7.04 (m, 2H), 6.25 (dq, *J* = 50.3, 5.1 Hz, 1H), 4.26–4.03 (m, 2H), 3.98–3.77 (m, 4H), 2.23–1.91 (m, 2H), 1.73–1.53 (m, 5H), 1.44 (dd, *J* = 14.8, 7.5 Hz, 2H); ^{19}F NMR (376 MHz, $CDCl_3$) *δ* (ppm): –124.02 to –141.66 (m, 1F); ^{13}C NMR (101 MHz, $CDCl_3$) *δ* (ppm): 174.83, 157.87, 133.89, 129.44, 129.11, 127.36, 126.39, 126.13, 119.24, 105.77, 95.25 (d, *J* = 252.4 Hz), 64.28, 55.52, 45.69, 40.13 (d, *J* = 21.8 Hz), 27.80, 21.71 (d, *J* = 4.0 Hz), 18.52. MS (ESI) *m/z*: 397.1 $[M+H]^+$. HRMS (ESI) *m/z* for $C_{19}H_{23}O_3F^{79}Br$ $[M+H]^+$: calc'd: 397.0815; found: 397.0811.

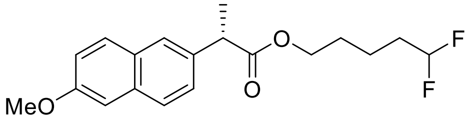

5,5-Difluoropentyl (*S*)-2-(6-methoxynaphthalen-2-yl)propanoate (36). General procedure 6 with naproxen followed by flash chromatography (ethyl acetate/*n*-hexane, 1: 10) gave **36** as a colorless oil (65 mg, 64% yield).

^{1}H NMR (400 MHz, $CDCl_3$) *δ* (ppm): 7.82–7.61 (m, 3H), 7.41 (d, *J* = 8.4 Hz, 1H), 7.21–7.04 (m, 2H), 5.64 (tt, *J* = 56.8, 4.3 Hz, 1H), 4.26–4.02 (m, 2H), 4.01–3.71 (m, 4H), 2.05–1.50 (m, 7H), 1.48–1.25 (m, 2H); ^{19}F NMR (376 MHz, $CDCl_3$) *δ* (ppm): –115.99 (dt, *J* = 57.0, 17.5 Hz, 2F); ^{13}C NMR (101 MHz, $CDCl_3$) *δ* (ppm): 174.83, 157.84, 135.86, 133.87, 129.41, 129.09, 127.32, 126.36, 126.10, 119.20, 117.20 (t, *J* = 238.9 Hz), 105.74, 64.32, 55.47, 45.65, 33.67 (t, *J* = 20.9

Hz), 28.13, 18.76 (t, J = 5.6 Hz), 18.55. MS (ESI) m/z: 337.2 $[M+H]^+$. HRMS (ESI) m/z for $C_{19}H_{23}O_3F_2$ $[M+H]^+$: calc'd: 337.1615; found: 337.1611.

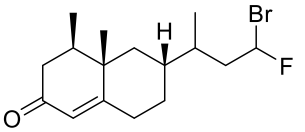

(4*R*,4a*S*,6*R*)-6-(4-Bromo-4-fluorobutan-2-yl)-4,4a-dimethyl-4,4a,5,6,7,8 hexahydronaphthalen-2(3*H*)-one (37a). General procedure 4 with nootkatone [(4*R*,4a*S*,6*R*)-4,4a-dimethyl-6-(prop-1-en-2-yl)-4,4a,5,6,7,8-hexahydronaphthalen-2(3*H*)-one] followed by flash chromatography (ethyl acetate/*n*-hexane, 1: 10) gave **37a** as a colorless oil (25 mg, d.r. = 1/1, 19% yield).

^{1}H NMR (400 MHz, $CDCl_3$) δ (ppm): 6.73–6.19 (m, 1H), 5.73 (s, 1H), 2.54–2.42 (m, 1H), 2.41–2.20 (m, 4H), 2.17–1.56 (m, 7H), 1.26–1.09 (m, 1H), 1.08–0.85 (m, 9H); ^{19}F NMR (376 MHz, $CDCl_3$) δ (ppm):–123.67 to –137.34 (m, 1F); ^{13}C NMR (101 MHz, $CDCl_3$) δ (ppm): 199.62, 170.52, 124.81, 124.79, 95.92 (dd, J = 251.8, 13.4 Hz), 94.30 (d, J = 252.8 Hz), 46.12-44.40 (m), 42.60 (d, J = 7.6 Hz), 42.19, 41.11, 40.96, 40.68, 39.38 (d, J = 11.3 Hz), 37.75 – 37.56 (m), 37.44, 35.10 (d, J = 4.1 Hz), 34.57, 33.07 (d, J = 11.2 Hz), 29.96 (d, J = 13.3 Hz), 28.44 (d, J = 12.6 Hz), 17.09, 15.79 (dd, J = 40.9, 30.5 Hz), 15.11, 15.10. MS (ESI) m/z: 331.1 $[M+H]^+$. HRMS (ESI) m/z for $C_{16}H_{25}OF^{79}Br$ $[M+H]^+$: calc'd: 331.1073; found: 331.1071.

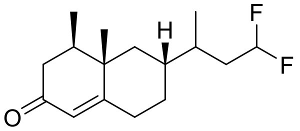

(4*R*,4a*S*,6*R*)-6-(4,4-Difluorobutan-2-yl)-4,4a-dimethyl-4,4a,5,6,7,8-hexahydronaphthalen-2(3*H*)-one (37). General procedure 5 with nootkatone followed by flash chromatography (ethyl acetate/*n*-hexane, 1: 10) gave **37** as a colorless oil (39 mg, d.r. = 1/1, 48% yield).

^{1}H NMR (400 MHz, $CDCl_3$) δ (ppm): 6.20–5.31 (m, 2H), 2.53–2.39 (m, 1H), 2.39–2.16 (m, 3H), 2.07–1.48 (m, 8H), 1.27–1.12 (m, 1H), 1.12–1.01 (m, 3H), 1.01–0.88 (m, 6H); ^{19}F NMR (376 MHz, $CDCl_3$) δ (ppm): –102.06 to –126.18 (m, 2F); ^{13}C NMR (101 MHz, $CDCl_3$) δ (ppm): 199.73, 170.68, 124.83 (d, J = 2.6 Hz), 117.38 (td, J = 238.9, 4.5 Hz), 42.42 (d, J = 37.5 Hz), 41.88–40.41, 39.40 (d, J = 10.2 Hz), 38.51 (dt, J = 47.2, 20.0 Hz), 37.80, 33.12 (d, J = 10.2 Hz), 32.37, 29.96, 28.51, 17.12, 16.37 (d, J = 36.6 Hz), 15.12 (d, J = 1.3 Hz). MS (ESI) m/z: 271.2 $[M+H]^+$. HRMS (ESI) m/z for $C_{16}H_{25}OF_2$ $[M+H]^+$: calc'd: 271.1873; found: 271.1873.

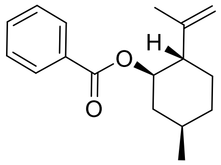

(1*R*,2*S*,5*R*)-5-methyl-2-(prop-1-en-2-yl)cyclohexyl benzoate (38b). To a round-bottomed flask equipped with a magnetic stirrer bar was added isopulegol [(1*R*,2*S*,5*R*)-5-methyl-2-(prop-1-en-2-yl)cyclohexan-1-ol; 771 mg,

5.0 mmol, 1.0 equiv.], DMAP (916 mg, 7.5 mmol, 1.5 equiv.) and DCM (20 mL) under argon. Benzoyl chloride (843 mg, 6.0 mmol, 1.2 equiv.) in DCM (3 mL) was then added dropwise at 0 °C. The reaction mixture was then stirred at RT for 12 h, poured onto water, and extracted with DCM (2 × 15 mL). The organic phase was dried (Mg_2SO_4) and concentrated in vacuo. The residue was purified by silica gel flash chromatography (ethyl acetate/*n*-hexane, 1: 20) to give **38b** as a colorless oil (1.02 g, 79% yield).

^{1}H NMR (400 MHz, $CDCl_3$) δ (ppm): 7.99 (d, *J* = 8.0 Hz, 2H), 7.52 (t, *J* = 7.3 Hz, 1H), 7.40 (t, *J* = 7.7 Hz, 2H), 5.02 (td, *J* = 10.9, 4.4 Hz, 1H), 4.75 (d, *J* = 30.9 Hz, 2H), 2.29 (td, *J* = 11.8, 3.5 Hz, 1H), 2.16 (d, *J* = 12.1 Hz, 1H), 1.83–1.58 (m, 6H), 1.54–1.38 (m, 1H), 1.14 (q, *J* = 11.8 Hz, 1H), 1.07–0.98 (m, 1H), 0.95 (d, *J* = 6.5 Hz, 3H); ^{13}C NMR (101 MHz, $CDCl_3$) δ (ppm): 166.21, 146.35, 132.81, 131.08, 129.74, 128.42, 112.14, 74.50, 51.10, 40.69, 34.37, 31.63, 30.68, 22.25, 19.69. MS (EI) *m/z*: 258.1 $[M]^+$. HRMS (EI) *m/z* for $C_{17}H_{22}O_2$ $[M]^+$: calc'd: 258.1620; found: 258.1619.

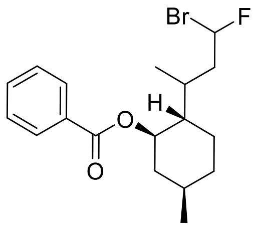

(1*R*,2*S*,5*R*)-2-(4-bromo-4-fluorobutan-2-yl)-5-methylcyclohexyl benzoate (38a). General procedure 4 with (1*R*,2*S*,5*R*)-5-methyl-2-(prop-1-en-2-yl)cyclohexyl benzoate followed by flash chromatography (ethyl acetate/*n*-hexane, 1: 20) gave **38a** as a colorless oil (69 mg, d.r. = 1/1, 47% yield).

^{1}H NMR (400 MHz, $CDCl_3$) δ (ppm): 8.04 (t, *J* = 7.7 Hz, 2H), 7.56 (t, *J* = 7.4 Hz, 1H), 7.44 (t, *J* = 7.7 Hz, 2H), 6.68–6.24 (m, 1H), 5.18–4.70 (m, 1H), 2.70–1.86 (m, 4H), 1.83–1.38 (m, 4H), 1.33–1.03 (m, 2H), 1.00–0.59 (m, 7H); ^{19}F NMR (376 MHz, $CDCl_3$) δ (ppm): -122.49 – -139.50 (m, 1F); ^{13}C NMR (101 MHz, $CDCl_3$) δ (ppm): 166.32–166.12 (m), 133.11–133.09 (m), 130.81–130.75 (m), 129.83–129.76 (m), 128.60, 97.55–93.52 (m), 47.11, 46.43–45.94 (m), 45.75–45.69 (m), 44.73–43.70 (m), 41.05–40.99 (m), 34.51–34.29 (m), 31.57, 30.95–28.99 (m), 26.39–23.77 (m), 22.19–22.15 (m), 17.47–13.96. MS (ESI) *m/z*: 393.1 $[M+Na]^+$. HRMS (ESI) *m/z* for $C_{18}H_{24}O_2F^{79}BrNa$ $[M+H]^+$: calc'd: 393.0841; found: 393.0847.

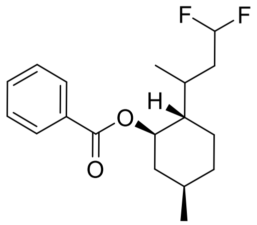

(1*R*,2*S*,5*R*)-2-(4,4-Difluorobutan-2-yl)-5-methylcyclohexyl benzoate (38). General procedure 5 with (1*R*,2*S*,5*R*)-5-methyl-2-(prop-1-en-2-yl)cyclohexyl benzoate followed by flash chromatography (ethyl acetate/*n*-hexane, 1: 20) gave **38** as a colorless oil (39 mg, d.r. = 1/1, 42% yield).

^{1}H NMR (400 MHz, $CDCl_3$) δ (ppm): 8.09–7.97 (m, 2H), 7.56 (t, J = 7.4 Hz, 1H), 7.45 (t, J = 7.6 Hz, 2H), 5.80 (tdt, J = 56.9, 34.5, 4.7 Hz, 1H), 4.95 (td, J = 10.8, 4.3 Hz, 1H), 2.24–1.48 (m, 8H), 1.32–1.06 (m, 2H), 1.02–0.87 (m, 7H); ^{19}F NMR (376 MHz, $CDCl_3$) δ (ppm): –100.17 to –136.43 (m, 2F); ^{13}C NMR (101 MHz, $CDCl_3$) δ (ppm): 166.21 (d, J = 11.0 Hz), 133.08, 130.75 (d, J = 3.9 Hz), 129.74 (d, J = 2.1 Hz), 128.59, 117.04 (dt, J = 238.5, 119.3 Hz), 74.11, 47.46, 46.09, 41.00 (d, J = 5.9 Hz), 39.45 (t, J = 20.0 Hz), 37.15 (t, J = 19.9 Hz), 34.35 (d, J = 14.6 Hz), 31.57, 27.76, 26.57, 25.76, 23.76, 22.14 (d, J = 2.6 Hz), 17.82, 14.43. MS (CI) *m/z*: 311.1 $[M+H]^+$. HRMS (CI) *m/z* for $C_{18}H_{25}O_2F_2$ $[M+H]^+$: calc'd: 311.1823; found: 311.1823.

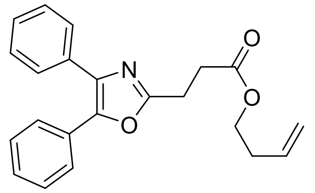

But-3-en-1-yl 3-(4,5-diphenyloxazol-2-yl)propanoate (39b). General procedure 3 with oxaprozin [3-(4,5-diphenyloxazol-2-yl)propanoic acid] followed by flash chromatography (ethyl acetate/*n*-hexane, 1: 10) gave **39b** as a white solid (1.23 g, 71% yield). Mp: 51–53 °C.

^{1}H NMR (400 MHz, $CDCl_3$) δ (ppm): 7.63 (dt, J = 3.6, 2.0 Hz, 2H), 7.57 (dt, J = 8.4, 2.1 Hz, 2H), 7.40–7.27 (m, 6H), 5.78 (ddt, J = 17.0, 10.2, 6.7 Hz, 1H), 5.22–4.94 (m, 2H), 4.19 (t, J = 6.7 Hz, 2H), 3.19 (t, J = 7.5 Hz, 2H), 2.92 (t, J = 7.6 Hz, 2H), 2.40 (q, J = 6.7 Hz, 2H); ^{13}C NMR (101 MHz, $CDCl_3$) δ (ppm): 172.15, 161.93, 145.60, 135.33, 134.08, 132.66, 129.19, 128.82, 128.73, 128.63, 128.24, 128.08, 126.66, 117.48, 64.01, 33.23, 31.34, 23.75. MS (ESI) *m/z*: 348.2 $[M+H]^+$. HRMS (ESI) *m/*z for $C_{22}H_{22}NO_3$ $[M+H]^+$: calc'd: 348.1600; found: 348.1596.

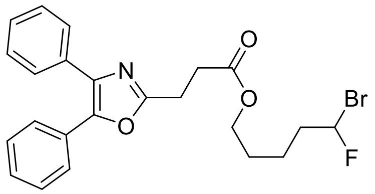

5-Bromo-5-fluoropentyl 3-(4,5-diphenyloxazol-2-yl)propanoate (39a). General procedure 4 with but-3-en-1-yl 3-(4,5-diphenyloxazol-2-yl)propanoate followed by flash chromatography (ethyl acetate/*n*-hexane, 1: 10) gave **39a** as a colorless oil (56 mg, 30% yield).

^{1}H NMR (400 MHz, $CDCl_3$) δ (ppm): 7.68–7.61 (m, 2H), 7.61–7.54 (m, 2H), 7.44–7.29 (m, 6H), 6.39 (dt, J = 50.3, 5.3 Hz, 1H), 4.15 (t, J = 6.3 Hz, 2H), 3.19 (t, J = 7.4 Hz, 2H), 2.92 (t, J = 7.5 Hz, 2H), 2.43–2.00 (m, 2H), 1.81–1.64 (m, 2H), 1.65–1.40 (m, 2H); ^{19}F NMR (376 MHz, $CDCl_3$) δ (ppm): –119.98 to –149.68 (m, 1F); ^{13}C NMR (101 MHz, $CDCl_3$) δ (ppm): 172.17, 161.89, 145.61, 135.32, 132.63, 129.16, 128.84, 128.75, 128.67, 128.27, 128.06, 126.66, 95.25 (d, J = 252.4 Hz), 64.38, 40.21 (d, J = 19.1 Hz), 31.31, 27.91, 23.73, 21.81 (d, J = 3.8 Hz). MS (ESI) *m/z*: 460.1 $[M+H]^+$. HRMS (ESI) *m/z* for $C_{23}H_{24}NO_3F^{79}Br$ $[M+H]^+$: calc'd: 460.0924; found: 460.0929.

5,5-Difluoropentyl 3-(4,5-diphenyloxazol-2-yl)propanoate (39). General procedure 5 with but-3-en-1-yl 3-(4,5-diphenyloxazol-2-yl)propanoate followed by flash chromatography (ethyl acetate/*n*-hexane, 1: 10) gave **39** as a colorless oil (53 mg, 44% yield).

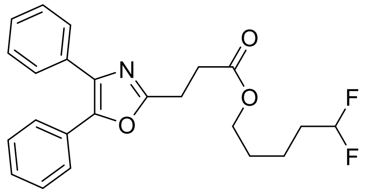

^{1}H NMR (400 MHz, $CDCl_3$) δ 7.63 (dd, J = 8.0, 1.3 Hz, 2H), 7.57 (dd, J = 8.0, 1.4 Hz, 2H), 7.45–7.28 (m, 6H), 5.75 (tt, J = 56.7, 4.4 Hz, 1H), 4.15 (t, J = 6.4 Hz, 2H), 3.19 (t, J = 7.4 Hz, 2H), 2.92 (t, J = 7.5 Hz, 2H), 1.90–1.61 (m, 4H), 1.61–1.42 (m, 2H); ^{19}F NMR (376 MHz, $CDCl_3$) δ (ppm): –115.96 (dt, J = 34.6, 17.6 Hz, 2F); ^{13}C NMR (101 MHz, $CDCl_3$) δ (ppm): 172.19, 161.90, 145.61, 135.32, 132.64, 129.17, 128.84, 128.74, 128.67, 128.26, 128.06, 126.66, 117.22 (t, J = 238.9 Hz), 64.43, 33.77 (t, J = 20.9 Hz), 31.30, 28.27, 23.73, 18.89 (t, J = 5.7 Hz). MS (ESI) *m/z*: 400.2 [M+H]$^+$. HRMS (ESI) *m/z* for $C_{23}H_{24}NO_3F_2$ [M+H]$^+$: calc'd: 400.1724; found: 400.1727.

But-3-en-1-yl 3-(5-(2-fluorophenyl)-1,2,4-oxadiazol-3-yl)benzoate (40b). General procedure 3 with ataluren [3-(5-(2-fluorophenyl)-1,2,4-oxadiazol-3-yl)benzoic acid] followed by flash chromatography (ethyl acetate/*n*-hexane, 1: 10) gave **40b** as a white solid (1.28 g, 66% yield). Mp: 62–64 °C.

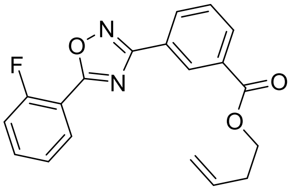

^{1}H NMR (400 MHz, $CDCl_3$) δ (ppm): 8.84 (t, J = 1.5 Hz, 1H), 8.37 (dt, J = 7.8, 1.4 Hz, 1H), 8.32–8.10 (m, 2H), 7.76–7.52 (m, 2H), 7.46–7.14 (m, 2H), 5.90 (ddt, J = 17.0, 10.2, 6.8 Hz, 1H), 5.36–5.05 (m, 2H), 4.43 (t, J = 6.8 Hz, 2H), 2.84–2.36 (m, 2H); ^{19}F NMR (376 MHz, $CDCl_3$) δ –94.59 to – 122.76 (m, 1F); ^{13}C NMR (101 MHz, $CDCl_3$) δ (ppm): 173.24 (d, J = 4.4 Hz), 168.29, 166.01, 161.01 (d, J = 260.6 Hz), 134.93 (d, J = 8.7 Hz), 134.09, 132.41, 131.94, 131.43, 131.17, 129.24, 128.91, 127.42, 124.94 (d, J = 3.7 Hz), 117.72, 117.39 (d, J = 20.8 Hz), 112.92 (d, J = 11.6 Hz), 64.52, 33.38. MS (ESI) *m/z*: 339.1 [M+H]$^+$. HRMS (ESI) *m/z* for $C_{19}H_{16}N_2O_3F$ [M+H]$^+$: calc'd: 339.1145; found: 339.1141.

5-Bromo-5-fluoropentyl 3-(5-(2-fluorophenyl)-1,2,4-oxadiazol-3-yl)benzoate (40a). General procedure 4 with but-3-en-1-yl 3-(5-(2-fluorophenyl)-1,2,4-oxadiazol-3-yl)benzoate followed by flash chromatography (ethyl acetate/*n*-hexane, 1: 10) gave **40a** as a colorless oil (86 mg, 48% yield).

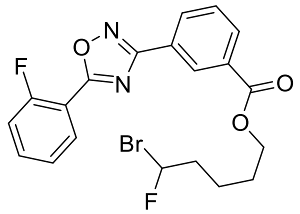

^{1}H NMR (400 MHz, $CDCl_3$) δ (ppm): 8.83 (t, J = 1.5 Hz, 1H), 8.48–8.33 (m, 1H), 8.33–8.15 (m, 2H), 7.76–7.51 (m, 2H), 7.51–7.16 (m, 2H), 6.52 (dt, J = 50.3, 5.3 Hz, 1H), 4.41 (t, J = 6.5 Hz, 2H), 2.57–2.16 (m, 2H), 1.98–1.82 (m, 2H), 1.82–1.67 (m, 2H); ^{19}F NMR (376 MHz, $CDCl_3$) δ (ppm): –102.06 to –118.16 (m, 1F), –118.16 to –138.59 (m, 1F); ^{13}C NMR (101 MHz, $CDCl_3$) δ (ppm): 173.24, 168.24, 166.01, 160.99 (d, J = 260.9 Hz), 134.95 (d, J = 8.5 Hz), 132.40, 132.01, 131.30, 131.18, 129.29, 128.87, 127.46, 124.95 (d, J = 3.7 Hz), 117.39 (d, J = 21.0 Hz), 112.88 (d, J = 11.5 Hz), 95.30 (d, J = 252.4 Hz), 64.90, 40.28 (d, J = 19.1 Hz), 28.05, 21.95 (d, J = 3.8 Hz) ppm. MS (ESI) *m/z*: 451.0 [M+H]$^+$. HRMS (ESI) *m/z* for $C_{20}H_{18}N_2O_3F_2{}^{79}Br$ [M+H]$^+$: calc'd: 451.0469; found: 451.0474.

5,5-Difluoropentyl 3-(5-(2-fluorophenyl)-1,2,4-oxadiazol-3-yl)benzoate (40). General procedure 5 with but-3-en-1-yl 3-(5-(2-fluorophenyl)-1,2,4-oxadiazol-3-yl)benzoate followed by flash chromatography (ethyl acetate/*n*-hexane, 1: 10) gave **40** as a colorless oil (52 mg, 44% yield).

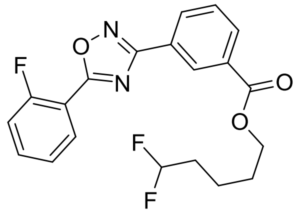

^{1}H NMR (400 MHz, $CDCl_3$) δ (ppm): 8.83 (s, 1H), 8.38 (d, J = 7.8 Hz, 1H), 8.31–8.12 (m, 2H), 7.73–7.52 (m, 2H), 7.48–7.11 (m, 2H), 5.86 (tt, J = 56.7, 4.3 Hz, 1H), 4.40 (t, J = 6.5 Hz, 2H), 2.21–1.80 (m, 4H), 1.80–1.48 (m, 2H); ^{19}F NMR (376 MHz, $CDCl_3$) δ (ppm): –104.84 to –111.33 (m, 1F), -115.99 (dt, J = 56.9, 17.5 Hz, 2F); ^{13}C NMR (101 MHz, $CDCl_3$) δ (ppm): 173.26, 168.27, 166.05, 161.02 (d, J = 260.8 Hz), 134.97 (d, J = 8.7 Hz), 132.41, 132.02, 131.33, 131.19, 129.31, 128.88, 127.47, 124.96 (d, J = 3.8 Hz), 117.36 (t, J = 239.0 Hz), 117.29 (d, J = 1.7 Hz), 112.91 (d, J = 11.2 Hz), 64.98, 33.90 (t, J = 21.0 Hz), 28.44, 19.05 (t, J = 5.7 Hz). MS (ESI) *m/z*: 391.1 [M+H]$^+$. HRMS (ESI) *m/z* for $C_{20}H_{18}N_2O_3F_3$ [M+H]$^+$: calc'd: 391.1270; found: 391.1277.

But-3-en-1-yl 2-(1,8-diethyl-1,3,4,9-tetrahydropyrano[3,4-*b*]indol-1-yl)acetate (41b). General procedure 3 with etodolac [2-(1,8-diethyl-1,3,4,9-tetrahydropyrano[3,4-*b*]indol-1-yl)acetic acid] followed by flash chromatography (ethyl acetate/*n*-hexane, 1: 10) gave **41b** as a colorless oil (1.32 g, 77% yield).

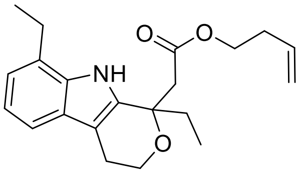

^{1}H NMR (400 MHz, $CDCl_3$) δ (ppm): 9.11 (s, 1H), 7.38 (d, J = 7.7 Hz, 1H), 7.16–6.95 (m, 2H), 5.78 (ddt, J = 17.0, 10.2, 6.7 Hz, 1H), 5.11 (ddd, J = 11.6, 6.6, 1.4 Hz, 2H), 4.34–3.82 (m, 4H), 3.09–2.64 (m, 6H), 2.41 (q, J = 6.7 Hz, 2H), 2.09 (dtd, J = 32.6, 14.5, 7.3 Hz, 2H), 1.40 (t, J = 7.6 Hz, 3H), 0.86 (t, J = 7.4 Hz, 3H); ^{13}C NMR (101 MHz, $CDCl_3$) δ (ppm): 173.00, 136.25, 134.66,

133.84, 126.80, 126.38, 120.56, 119.77, 117.70, 116.13, 108.53, 74.79, 64.18, 60.81, 43.19, 33.15, 30.83, 24.41, 22.61, 13.97, 7.79. MS (ESI) *m/z*: 342.2 [M+H]$^+$. HRMS (ESI) *m/z* for $C_{21}H_{28}NO_3$ [M+H]$^+$: calc'd: 342.2069; found: 342.2068.

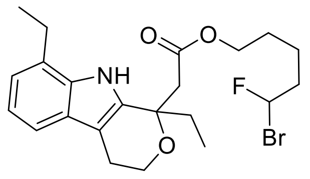

5-Bromo-5-fluoropentyl 2-(1,8-diethyl-1,3,4,9-tetrahydropyrano[3,4-*b*]indol-1-yl)acetate (41a). General procedure 4 with but-3-en-1-yl 2-(1,8-diethyl-1,3,4,9-tetrahydropyrano[3,4-*b*]indol-1-yl)acetate followed by flash chromatography (ethyl acetate/*n*-hexane, 1: 10) gave **41a** as a colorless oil (69 mg, d.r. = 1/1, 38% yield).

^{1}H NMR (400 MHz, $CDCl_3$) δ (ppm): 9.01 (s, 1H), 7.38 (d, J = 7.6 Hz, 1H), 7.16–6.95 (m, 2H), 6.42 (dtd, J = 50.3, 5.3, 2.2 Hz, 1H), 4.31–3.71 (m, 4H), 3.34–2.54 (m, 6H), 2.40–1.89 (m, 4H), 1.87–1.50 (m, 4H), 1.39 (t, J = 7.6 Hz, 3H), 0.86 (t, J = 7.4 Hz, 3H); ^{19}F NMR (376 MHz, $CDCl_3$) δ (ppm): –131.09 (ddd, J = 50.3, 37.4, 18.3 Hz, 1F); ^{13}C NMR (101 MHz, $CDCl_3$) δ (ppm): 172.94, 136.09, 134.66, 126.77, 126.38, 120.61, 119.83, 116.17, 108.66, 95.17 (d, J = 253.5 Hz), 74.83, 64.56, 60.84, 43.25, 40.18 (d, J = 18.9 Hz), 30.97, 27.81, 24.40, 22.59, 21.79 (d, J = 4.0 Hz), 13.98, 7.82. MS (ESI) *m/z*: 454.1 [M+H]$^+$. HRMS (ESI) *m/z* for $C_{22}H_{30}NO_3F^{79}Br$ [M+H]$^+$: calc'd: 454.1393; found: 454.1391.

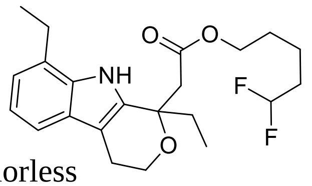

5,5-Difluoropentyl 2-(1,8-diethyl-1,3,4,9-tetrahydropyrano[3,4-*b*]indol-1-yl)acetate (41). General procedure 6 with but-3-en-1-yl 2-(1,8-diethyl-1,3,4,9-tetrahydropyrano[3,4-*b*]indol-1-yl)acetate followed by flash chromatography (ethyl acetate/*n*-hexane, 1: 10) gave **41** as a colorless oil (72 mg, 61% yield).

^{1}H NMR (400 MHz, $CDCl_3$) δ (ppm): 9.05 (s, 1H), 7.40 (d, J = 7.6 Hz, 1H), 7.19–6.89 (m, 2H), 5.79 (tt, J = 56.7, 4.3 Hz, 1H), 4.35–3.82 (m, 4H), 3.24–2.63 (m, 6H), 2.30–1.97 (m, 2H), 1.93–1.77 (m, 2H), 1.75–1.65 (m, 2H), 1.57–1.47 (m, 2H), 1.41 (t, J = 7.6 Hz, 3H), 0.88 (t, J = 7.3 Hz, 3H); ^{19}F NMR (376 MHz, $CDCl_3$) δ (ppm): –116.01 (dt, J = 56.9, 17.6 Hz, 2F); ^{13}C NMR (101 MHz, $CDCl_3$) δ (ppm): 172.95, 136.09, 134.65, 126.76, 126.37, 120.59, 119.80, 117.16 (t, J = 239.0 Hz), 116.14, 108.62, 74.82, 64.59, 60.80, 43.21, 33.74 (t, J = 21.0 Hz), 30.92, 28.14, 24.38, 22.57, 18.84 (t, J = 5.7 Hz), 13.95, 7.78. MS (ESI) *m/z*: 394.2 [M+H]$^+$. HRMS (ESI) *m/z* for $C_{22}H_{30}NO_3F_2$ [M+H]$^+$: calc'd: 394.2194; found: 394.2201.

But-3-en-1-yl 2-(3-cyano-4-isobutoxyphenyl)-4-methylthiazole-5-carboxylate (42b). General procedure 3 with febuxostat [2-(3-cyano-4-isobutoxyphenyl)-4-methylthiazole-5-carboxylic acid] followed by flash chromatography (ethyl acetate/*n*-hexane, 1: 10) gave **42b** as a white solid (1.52 g, 82% yield). Mp: 104–106 °C.

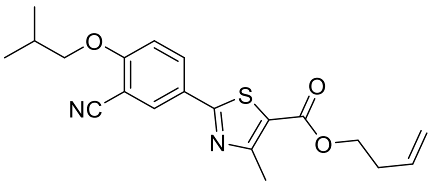

^{1}H NMR (400 MHz, $CDCl_3$) δ (ppm): 8.13 (d, J = 1.8 Hz, 1H), 8.05 (dd, J = 8.8, 1.8 Hz, 1H), 6.98 (d, J = 8.9 Hz, 1H), 5.83 (ddt, J = 17.0, 10.2, 6.7 Hz, 1H), 5.13 (dd, J = 20.4, 13.7 Hz, 2H), 4.33 (t, J = 6.6 Hz, 2H), 3.87 (d, J = 6.5 Hz, 2H), 2.72 (s, 3H), 2.49 (q, J = 6.5 Hz, 2H), 2.18 (dp, J = 13.2, 6.6 Hz, 1H), 1.07 (d, J = 6.7 Hz, 6H); ^{13}C NMR (101 MHz, $CDCl_3$) δ (ppm): 167.34, 162.60, 162.07, 161.28, 133.87, 132.67, 132.14, 126.10, 121.95, 117.77, 115.51, 112.75, 103.05, 75.82, 64.47, 33.24, 28.29, 19.18, 17.64. MS (ESI) *m/z*: 371.1 $[M+H]^+$. HRMS (ESI) *m/z* for $C_{20}H_{23}N_2O_3{}^{32}S$ $[M+H]^+$: calc'd: 371.1429; found: 371.1423.

5-Bromo-5-fluoropentyl 2-(3-cyano-4-isobutoxyphenyl)-4-methylthiazole-5-carboxylate (42a). General procedure 4 with but-3-en-1-yl 2-(3-cyano-4-isobutoxyphenyl)-4-methylthiazole-5-carboxylate followed by flash chromatography (ethyl acetate/*n*-hexane, 1: 10) gave **42a** as a white solid (92 mg, 48% yield). Mp: 99–101 °C.

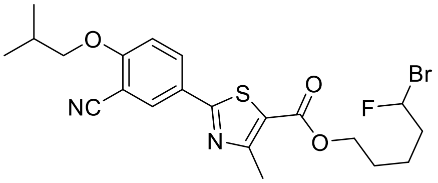

^{1}H NMR (400 MHz, $CDCl_3$) δ (ppm): 8.17 (d, J = 1.8 Hz, 1H), 8.08 (dd, J = 8.8, 1.8 Hz, 1H), 7.00 (d, J = 8.9 Hz, 1H), 6.50 (dt, J = 50.3, 5.2 Hz, 1H), 4.32 (t, J = 6.3 Hz, 2H), 3.89 (d, J = 6.5 Hz, 2H), 2.76 (s, 3H), 2.44–2.08 (m, 3H), 1.95–1.74 (m, 2H), 1.74–1.63 (m, 2H), 1.08 (d, J = 6.7 Hz, 6H); ^{19}F NMR (376 MHz, $CDCl_3$) δ (ppm): –122.76 to –143.54 (m, 1F); ^{13}C NMR (101 MHz, $CDCl_3$) δ (ppm): 167.49, 162.72, 162.16, 161.54, 132.75, 132.29, 126.15, 121.77, 115.57, 112.82, 103.18, 96.47, 93.95, 75.90, 64.90, 40.21 (d, J = 19.3 Hz), 28.35, 27.98, 21.91 (d, J = 3.8 Hz), 19.24, 17.70. MS (ESI) *m/z*: 483.1 $[M+H]^+$. HRMS (ESI) *m/z* for $C_{21}H_{25}N_2O_3F^{32}S^{79}Br$ $[M+H]^+$: calc'd: 483.0753; found: 483.0755.

5,5-Difluoropentyl 2-(3-cyano-4-isobutoxyphenyl)-4-methylthiazole-5-carboxylate (42). General procedure 5 with but-3-en-1-yl 2-(3-cyano-4-isobutoxyphenyl)-4-

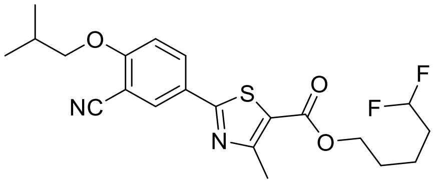

methylthiazole-5-carboxylate followed by flash chromatography (ethyl acetate/*n*-hexane, 1: 10) gave **42** as a white solid (65 mg, 51% yield). Mp: 100–102 °C.

^{1}H NMR (400 MHz, $CDCl_3$) δ (ppm): 8.16 (d, J = 2.1 Hz, 1H), 8.08 (dd, J = 8.8, 2.1 Hz, 1H), 7.00 (d, J = 8.9 Hz, 1H), 5.84 (tt, J = 56.7, 4.3 Hz, 1H), 4.31 (t, J = 6.4 Hz, 2H), 3.89 (d, J = 6.5 Hz, 2H), 2.75 (s, 3H), 2.20 (dt, J = 13.3, 6.6 Hz, 1H), 2.03–1.82 (m, 4H), 1.71–1.55 (m, 2H), 1.08 (d, J = 6.7 Hz, 6H); ^{19}F NMR (376 MHz, $CDCl_3$) δ (ppm): –116.07 (dt, J = 56.9, 17.5 Hz, 2F); ^{13}C NMR (101 MHz, $CDCl_3$) δ (ppm): 167.47, 162.71, 162.17, 161.52, 132.75, 132.28, 126.15, 121.77, 117.18 (t, J = 239.0 Hz), 115.56, 112.81, 103.18, 75.89, 64.93, 33.80 (t, J = 21.1 Hz), 28.34, 19.23, 18.95 (t, J = 5.7 Hz), 17.68. MS (ESI) *m/z*: 423.2 [M+H]$^+$. HRMS (ESI) *m/z* for $C_{21}H_{25}N_2O_3F_2{}^{32}S$ [M+H]$^+$: calc'd: 423.1554; found: 423.1557.

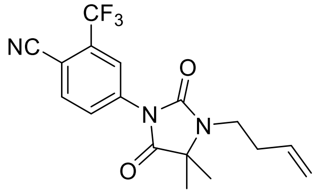

4-(3-(But-3-en-1-yl)-4,4-dimethyl-2,5-dioxoimidazolidin-1-yl)-2-(trifluoromethyl)benzonitrile (43b). General procedure 3 with RU 56279 [4-(4,4-dimethyl-2,5-dioxoimidazolidin-1-yl)-2-(trifluoromethyl)benzonitrile] on a 3.0 mmol scale followed by flash chromatography (ethyl acetate/*n*-hexane, 1: 3) gave **43b** as a white solid (637 mg, 60% yield). Mp: 96–98 °C.

^{1}H NMR (400 MHz, $CDCl_3$) δ (ppm): 8.15 (s, 1H), 8.00 (dd, J = 8.5, 1.3 Hz, 1H), 7.90 (d, J = 8.4 Hz, 1H), 5.80 (ddt, J = 17.1, 10.2, 6.9 Hz, 1H), 5.12 (t, J = 12.8 Hz, 2H), 3.96–2.87 (m, 2H), 2.47 (dd, J = 14.8, 7.2 Hz, 2H), 1.53 (s, 6H); ^{19}F NMR (376 MHz, $CDCl_3$) δ (ppm): –63.72 (s, 3F); ^{13}C NMR (101 MHz, $CDCl_3$) δ (ppm): 174.77, 152.93, 136.68, 135.42, 134.41, 133.71 (q, J = 33.3 Hz), 128.05, 123.12 (q, J = 4.9 Hz), 122.15 (q, J = 274.1 Hz), 117.91, 115.22, 108.31, 62.01, 40.02, 33.81, 23.70. MS (ESI) *m/z*: 352.1 [M+H]$^+$. HRMS (ESI) *m/z* for $C_{17}H_{17}N_3O_2F_3$ [M+H]$^+$: calc'd: 352.1273; found: 352.1271.

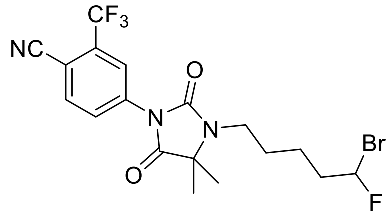

4-(3-(5-Bromo-5-fluoropentyl)-4,4-dimethyl-2,5-dioxoimidazolidin-1-yl)-2-(trifluoromethyl)benzonitrile (43a). General procedure 4 with 4-(3-(but-3-en-1-yl)-4,4-dimethyl-2,5-dioxoimidazolidin-1-yl)-2-(trifluoromethyl)benzonitrile followed by flash chromatography (ethyl acetate/*n*-hexane, 1: 3) gave **43a** as a colorless oil (153 mg, 82% yield).

^{1}H NMR (400 MHz, $CDCl_3$) δ (ppm): 8.14 (s, 1H), 8.00 (dd, J = 8.5, 1.4 Hz, 1H), 7.90 (d, J = 8.4 Hz, 1H), 6.48 (dt, J = 50.2, 5.1 Hz, 1H), 3.57–3.13 (m, 2H), 2.56–2.06 (m, 2H), 1.87–1.73 (m, 2H), 1.68–1.57 (m, 2H), 1.53 (s, 6H); ^{19}F NMR (376 MHz, $CDCl_3$) δ (ppm): –63.72 (s, 3F), -132.94 (dt, J = 50.3, 19.1 Hz, 1F); ^{13}C NMR (101 MHz, $CDCl_3$) δ (ppm): 174.68, 153.02, 136.62, 135.45, 133.72 (q, J = 33.1 Hz), 128.06, 123.13 (q, J = 4.9 Hz), 122.14 (q, J = 274.2 Hz), 115.20, 108.38, 95.10 (d, J = 252.4 Hz), 62.05, 40.16, 39.98 (d, J = 19.1 Hz), 28.75, 23.66, 22.60 (d, J = 3.8 Hz). MS (ESI) *m/z*: 481.1 $[M+NH_4]^+$. HRMS (ESI) *m/z* for $C_{18}H_{22}N_4O_2F_4{}^{79}Br$ $[M+NH_4]^+$: calc'd: 481.0862; found: 481.0861.

4-(3-(5,5-Difluoropentyl)-4,4-dimethyl-2,5-dioxoimidazolidin-1-yl)-2-(trifluoromethyl)benzonitrile (43). General procedure 6 with 4-(3-(but-3-en-1-yl)-4,4-dimethyl-2,5-dioxoimidazolidin-1-yl)-2-(trifluoromethyl)benzonitrile followed by flash chromatography (ethyl acetate/*n*-hexane, 1: 3) gave **43** as a colorless oil (54 mg, 45% yield).

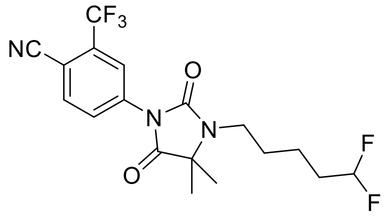

^{1}H NMR (400 MHz, $CDCl_3$) δ (ppm): 8.14 (s, 1H), 8.00 (d, J = 8.5 Hz, 1H), 7.90 (d, J = 8.4 Hz, 1H), 5.83 (tt, J = 56.7, 4.2 Hz, 1H), 3.46–3.24 (m, 2H), 2.04–1.71 (m, 4H), 1.63–1.42 (m, 8H); ^{19}F NMR (376 MHz, $CDCl_3$) δ (ppm): –63.72 (s, 3F), –117.85 (dt, J = 56.6, 17.7 Hz, 2F); ^{13}C NMR (101 MHz, $CDCl_3$) δ (ppm): 174.71, 153.03, 136.64, 135.45, 133.72 (q, J = 33.4 Hz), 128.06, 123.14 (q, J = 4.7 Hz), 122.15 (q, J = 274.1 Hz), 117.07 (t, J = 239.0 Hz), 115.21, 108.35, 62.05, 40.19, 33.64 (t, J = 21.0 Hz), 29.12, 23.64, 19.67 (t, J = 5.5 Hz). MS (ESI) *m/z*: 426.1 $[M+Na]^+$. HRMS (ESI) *m/z* for $C_{18}H_{18}N_3O_2F_5Na$ $[M+Na]^+$: calc'd: 426.1217; found: 426.1210.

2-(1-(But-3-en-1-yl)-2,6-dioxopiperidin-3-yl)isoindoline-1,3-dione (44b). General procedure 3 with thalidomide [2-(2,6-dioxopiperidin-3-yl)isoindoline-1,3-dione] followed by flash chromatography (ethyl acetate/*n*-hexane, 1: 4) gave **44b** as a white solid (962 mg, 62% yield). Mp: 126–128 °C.

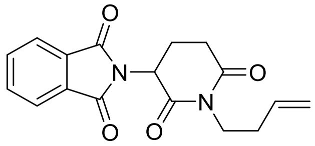

^{1}H NMR (400 MHz, $CDCl_3$) δ (ppm): 7.93–7.81 (m, 2H), 7.81–7.69 (m, 2H), 5.74 (ddt, J = 17.2, 10.1, 7.1 Hz, 1H), 5.36–4.78 (m, 4H), 4.15–3.72 (m, 2H), 3.09–2.87 (m, 1H), 2.87–2.63 (m, 2H), 2.31 (q, J = 7.2 Hz, 2H), 2.18–1.87 (m, 1H); ^{13}C NMR (101 MHz, $CDCl_3$) δ (ppm): 171.03, 168.63, 167.62, 134.93, 134.62, 132.00, 123.94, 117.40, 50.39, 39.91, 32.40, 32.25, 22.18. MS (ESI) *m/z*: 313.1 $[M+H]^+$. HRMS (ESI) *m/z* for $C_{17}H_{17}N_2O_4$ $[M+H]^+$: calc'd: 313.1188; found: 313.1185.

2-(1-(5-Bromo-5-fluoropentyl)-2,6-dioxopiperidin-3-yl)isoindoline-1,3-dione (44a). General procedure 4 with 2-(1-(but-3-en-1-yl)-2,6-dioxopiperidin-3-yl)isoindoline-1,3-dione followed by flash chromatography (ethyl acetate/*n*-hexane, 1: 4) gave **44a** as a colorless oil (78 mg, d.r. = 1/1, 46% yield).

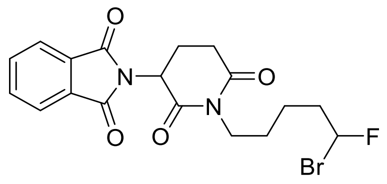

^{1}H NMR (400 MHz, $CDCl_3$) δ (ppm): 7.94–7.80 (m, 2H), 7.81–7.69 (m, 2H), 6.44 (dtd, *J* = 50.3, 5.4, 2.0 Hz, 1H), 4.98 (dd, *J* = 12.4, 5.4 Hz, 1H), 4.02–3.68 (m, 2H), 3.04–2.86 (m, 1H), 2.86–2.66 (m, 2H), 2.49–1.98 (m, 3H), 1.78–1.40 (m, 4H); ^{19}F NMR (376 MHz, $CDCl_3$) δ (ppm): –124.30 to –143.54 (m, 1F); ^{13}C NMR (101 MHz, $CDCl_3$) δ (ppm): 171.02, 168.72, 167.58, 134.64, 131.92, 123.93, 95.43 (dd, *J* = 252.3, 6.8 Hz), 50.27, 40.21, 40.18 (dd, *J* = 18.9, 3.1 Hz), 32.13, 26.94, 22.45 (d, *J* = 4.1 Hz), 22.21. MS (ESI) *m/z*: 425.1 $[M+H]^+$. HRMS (ESI) *m/z* for $C_{18}H_{19}N_2O_4F^{79}Br$ $[M+H]^+$: calc'd: 425.0512; found: 425.0517.

2-(1-(5,5-Difluoropentyl)-2,6-dioxopiperidin-3-yl)isoindoline-1,3-dione (44). General procedure 5 with 2-(1-(but-3-en-1-yl)-2,6-dioxopiperidin-3-yl)isoindoline-1,3-dione followed by flash chromatography (ethyl acetate/*n*-hexane, 1: 4) gave **44** as a colorless oil (62 mg, 57% yield).

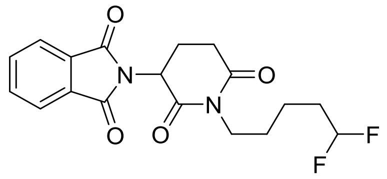

^{1}H NMR (400 MHz, $CDCl_3$) δ (ppm): 7.88 (dt, *J* = 7.0, 3.5 Hz, 2H), 7.80–7.68 (m, 2H), 5.79 (tt, *J* = 56.8, 4.5 Hz, 1H), 5.19–4.70 (m, 1H), 4.07–3.59 (m, 2H), 3.10–2.87 (m, 1H), 2.87–2.64 (m, 2H), 2.34–2.05 (m, 1H), 2.04–1.75 (m, 2H), 1.75–1.55 (m, 2H), 1.55–1.34 (m, 2H); ^{19}F NMR (376 MHz, $CDCl_3$) δ (ppm):–109.45 to –129.88 (m, 2F); ^{13}C NMR (101 MHz, $CDCl_3$) δ (ppm): 171.03, 168.72, 167.60, 134.65, 131.97, 123.96, 117.35 (t, *J* = 239.0 Hz), 50.31, 40.31, 33.79 (t, *J* = 21.0 Hz), 32.17, 27.37, 22.24, 19.62 (t, *J* = 5.7 Hz). MS (ESI) *m/z*: 365.1 $[M+H]^+$. HRMS (ESI) *m/z* for $C_{18}H_{19}N_2O_4F_2$ $[M+H]^+$: calc'd: 365.1313; found: 365.1308.

3-(But-3-en-1-yloxy)-1,5-bis(4-methoxyphenyl)-1*H*-1,2,4-triazole (45b). General procedure 3 with 1,5-bis(4-methoxyphenyl)-1*H*-1,2,4-triazol-3-ol followed by flash chromatography (ethyl acetate/*n*-hexane, 1: 4) gave **45b** as a white solid (1.08 g, 61% yield). Mp: 90–92 °C.

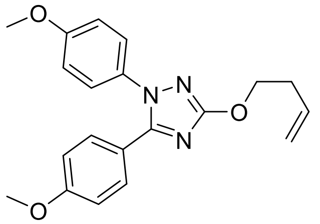

^{1}H NMR (400 MHz, $CDCl_3$) δ (ppm): 7.42 (d, *J* = 8.7 Hz, 2H), 7.27 (d, *J* = 8.8 Hz, 2H), 6.91 (d, *J* = 8.8 Hz, 2H), 6.81 (d, *J* = 8.7 Hz, 2H), 5.93 (ddt, *J* = 17.0, 10.2, 6.7

Hz, 1H), 5.14 (dd, J = 33.7, 13.7 Hz, 2H), 4.38 (t, J = 6.8 Hz, 2H), 3.84 (s, 3H), 3.79 (s, 3H), 2.59 (q, J = 6.6 Hz, 2H); ^{13}C NMR (101 MHz, $CDCl_3$) δ (ppm): 167.79, 160.93, 159.80, 153.15, 134.37, 131.64, 130.42, 127.28, 120.36, 117.32, 114.71, 114.01, 68.88, 55.72, 55.46, 33.74. MS (ESI) *m/z*: 353.2 $[M+H]^+$. HRMS (ESI) *m/z* for $C_{20}H_{22}N_3O_3$ $[M+H]^+$: calc'd: 352.1661; found: 352.1657.

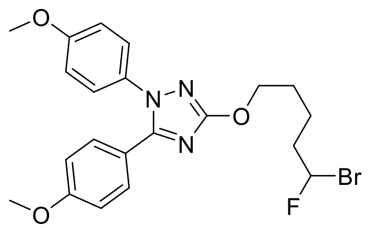

3-((5-Bromo-5-fluoropentyl)oxy)-1,5-bis(4-methoxyphenyl)-1*H*-1,2,4-triazole (45a). General procedure 4 with 3-(but-3-en-1-yloxy)-1,5-bis(4-methoxyphenyl)-1*H*-1,2,4-triazole followed by flash chromatography (ethyl acetate/*n*-hexane, 1: 4) gave **45a** as a colorless oil (72 mg, 39% yield).

^{1}H NMR (400 MHz, $CDCl_3$) δ (ppm): 7.42 (d, J = 8.7 Hz, 2H), 7.27 (d, J = 8.8 Hz, 2H), 6.92 (d, J = 8.8 Hz, 2H), 6.82 (d, J = 8.7 Hz, 2H), 6.48 (dt, J = 50.3, 5.4 Hz, 1H), 4.35 (t, J = 6.2 Hz, 2H), 3.84 (s, 3H), 3.80 (s, 3H), 2.48–2.13 (m, 2H), 2.05–1.83 (m, 2H), 1.81–1.66 (m, 2H); ^{19}F NMR (376 MHz, $CDCl_3$) δ (ppm): –130.62 (ddd, J = 50.4, 20.2, 17.8 Hz, 1F); ^{13}C NMR (101 MHz, $CDCl_3$) δ (ppm): 167.79, 160.99, 159.85, 153.23, 131.62, 130.43, 127.30, 120.31, 114.75, 114.06, 95.50 (d, J = 252.4 Hz), 69.04, 55.75, 55.48, 40.41 (d, J = 19.0 Hz), 28.43, 21.91 (d, J = 3.9 Hz). MS (ESI) *m/z*: 464.1 $[M+H]^+$. HRMS (ESI) *m/z* for $C_{21}H_{24}N_3O_3F^{79}Br$ $[M+H]^+$: calc'd: 464.0985; found: 464.0985.

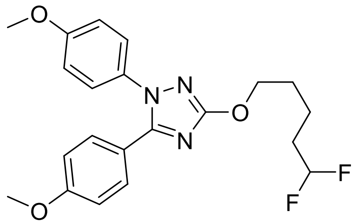

3-((5,5-Difluoropentyl)oxy)-1,5-bis(4-methoxyphenyl)-1*H*-1,2,4-triazole (45). General procedure 5 with 3-(but-3-en-1-yloxy)-1,5-bis(4-methoxyphenyl)-1*H*-1,2,4-triazole followed by flash chromatography (ethyl acetate/*n*-hexane, 1: 4) gave **45** as a colorless oil (68 mg, 56% yield).

^{1}H NMR (400 MHz, $CDCl_3$) δ (ppm): 7.42 (d, J = 8.8 Hz, 2H), 7.26 (d, J = 8.8 Hz, 2H), 6.91 (d, J = 8.8 Hz, 2H), 6.81 (d, J = 8.8 Hz, 2H), 5.82 (tt, J = 56.8, 4.4 Hz, 1H), 4.34 (t, J = 6.2 Hz, 2H), 3.84 (s, 3H), 3.79 (s, 3H), 2.07–1.81 (m, 4H), 1.81–1.53 (m, 2H); ^{19}F NMR (376 MHz, $CDCl_3$) δ (ppm): –115.78 (dt, J = 34.5, 17.5 Hz, 2F); ^{13}C NMR (101 MHz, $CDCl_3$) δ (ppm): 167.81, 160.99, 159.85, 153.22, 131.62, 130.43, 127.31, 120.32, 118.65 (d, J = 239.0 Hz), 114.75, 114.05, 69.07, 55.75, 55.48, 33.93 (t, J = 20.8 Hz), 28.78, 18.97 (t, J = 5.7 Hz). MS (ESI) *m/z*: 404.2 $[M+H]^+$. HRMS (ESI) *m/z* for $C_{21}H_{24}N_3O_3F_2$ $[M+H]^+$: calc'd: 404.1786; found: 404.1791.

3-Methylbut-2-en-1-yl 4-(*N,N*-dipropylsulfamoyl)benzoate (46b). To a dried round-bottomed flask equipped with a stirrer bar were added probenecid [4-(*N,N*-dipropylsulfamoyl)benzoic acid; 1.43 g, 5.0 mmol, 1 equiv.], K_2CO_3 (1.38 g, 10 mmol, 2.0 equiv.) and DMF (30 mL). 5-Bromo-2-methylpent-2-ene (1.49 g, 10 mmol, 2.0 equiv.) was then added. The reaction mixture was stirred for 12 h at 50 °C and then water (50 mL) and DCM (50 mL) were added. The organic phase was washed with brine, dried (Mg_2SO_4), and concentrated *in vacuo*. The residue was purified with silica gel chromatography (ethyl acetate/*n*-hexane, 1: 10) to give **46b** as a colorless oil (1.58 g, 89% yield).

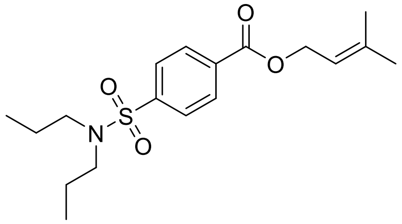

^{1}H NMR (400 MHz, $CDCl_3$) δ (ppm): 8.14 (d, J = 8.4 Hz, 12H), 7.84 (d, J = 8.4 Hz, 2H), 5.45 (t, J = 7.3 Hz, 1H), 4.83 (d, J = 7.2 Hz, 4H), 3.31–2.88 (m, 4H), 1.77 (d, J = 6.3 Hz, 6H), 1.63–1.33 (m, 4H), 0.84 (t, J = 7.4 Hz, 6H); ^{13}C NMR (101 MHz, $CDCl_3$) δ (ppm): 165.46, 144.25, 140.04, 133.99, 130.38, 127.10, 118.34, 62.63, 50.06, 25.97, 22.06, 18.29, 11.31 ppm. MS (ESI) *m/z*: 354.2 [*M*+H]$^+$. HRMS (ESI) *m/z* for $C_{18}H_{28}NO_4{}^{32}S$ [*M*+H]$^+$: calc'd: 354.1733; found: 354.1739.

2-(Bromofluoromethyl)-3-methylbutyl 4-(*N,N*-dipropylsulfamoyl)benzoate (46a). General procedure 4 with 3-methylbut-2-en-1-yl 4-(*N,N*-dipropylsulfamoyl)benzoate followed by flash chromatography (ethyl acetate/*n*-hexane, 1: 10) gave **46a** as a colorless oil (67 mg, d.r. = 1/1, 36% yield).

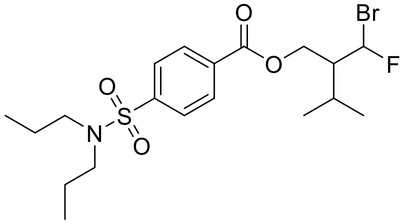

^{1}H NMR (400 MHz, $CDCl_3$) δ (ppm): 8.14 (dd, J = 8.3, 1.2 Hz, 2H), 7.87 (d, J = 8.4 Hz, 2H), 6.72 (ddd, J = 49.0, 25.6, 3.7 Hz, 1H), 4.87–4.40 (m, 2H), 3.25–2.75 (m, 4H), 2.50–2.04 (m, 2H), 1.81–1.37 (m, 4H), 1.26–1.01 (m, 6H), 0.86 (t, J = 7.4 Hz, 6H); ^{19}F NMR (376 MHz, $CDCl_3$) δ (ppm): –138.03 (ddd, J = 71.4, 49.1, 18.5 Hz, 1F); ^{13}C NMR (101 MHz, $CDCl_3$) δ (ppm): 165.14, 144.65, 133.32, 130.46 (d, J = 1.1 Hz), 127.28, 96.63 (dd, J = 256.0, 47.2 Hz), 62.98 (dd, J = 41.7, 4.4 Hz), 52.24 (d, J = 16.4 Hz), 51.38 (d, J = 15.6 Hz), 50.16, 28.20, 27.65 (d, J = 3.9 Hz), 22.16, 21.18, 20.88, 19.99, 19.63, 11.34. MS (ESI) *m*/*z*: 466.1 [*M*+H]$^+$. HRMS (ESI) *m*/*z* for $C_{19}H_{30}NO_4F^{32}S^{79}Br$ [*M*+H]$^+$: calc'd: 466.1063; found: 466.1070.

2-(Difluoromethyl)-3-methylbutyl 4-(*N*,*N*-dipropylsulfamoyl)benzoate (46). General procedure 7 with 3-methylbut-2-en-1-yl 4-(*N*,*N*-dipropylsulfamoyl)benzoate followed by flash chromatography (ethyl acetate/*n*-hexane, 1: 10) gave **46** as a colorless oil (78 mg, 48% yield).

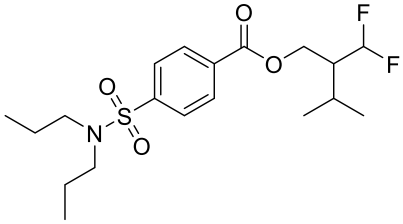

^{1}H NMR (400 MHz, $CDCl_3$) δ (ppm): 8.13 (d, J = 8.4 Hz, 2H), 7.87 (d, J = 8.4 Hz, 2H), 6.00 (td, J = 55.7, 3.3 Hz, 1H), 4.70–4.32 (m, 2H), 3.31–2.92 (m, 4H), 2.30–1.83 (m, 2H), 1.60–1.39 (m, 4H), 1.08 (dd, J = 13.8, 6.6 Hz, 6H), 1.02–0.70 (m, 6H); ^{19}F NMR (376 MHz, $CDCl_3$) δ (ppm): –121.74 (ddd, J = 286.2, 74.7, 55.8, 16.3 Hz, 2F); ^{13}C NMR (101 MHz, $CDCl_3$) δ (ppm): 165.20, 144.64, 133.37, 130.43, 127.27, 117.30 (t, J = 243.1 Hz), 61.44 (d, J = 5.6 Hz), 50.16, 47.59 (t, J = 18.2 Hz), 25.79, 22.15, 20.33 (d, J = 76.0 Hz), 11.34. MS (ESI) *m*/*z*: 406.2 $[M+H]^+$. HRMS (ESI) *m*/*z* for $C_{19}H_{30}NO_4{}^{32}SF_2$ $[M+H]^+$: calc'd: 406.1864; found: 406.1864.

1-(4-Methoxyphenyl)-3-((4-methylpent-3-en-1-yl)oxy)-5-phenyl-1*H*-1,2,4-triazole (47b). To a dried round-bottomed flask equipped with a stirrer bar were added 1-(4-methoxyphenyl)-5-phenyl-1*H*-1,2,4-triazol-3-ol (1.34 g, 5.0 mmol, 1 equiv.), K_2CO_3 (1.38 g, 10 mmol, 2.0 equiv.) and DMF (30 mL). 5-Bromo-2-methylpent-2-ene (1.63 g, 10 mmol, 2.0 equiv.) was then added. The reaction mixture was stirred for 12 h at 50 °C and then water (50 mL) and DCM (50 mL) were added. The organic phase was washed with brine, dried (Mg_2SO_4), and concentrated *in vacuo*. Silica gel chromatography (ethyl acetate/*n*-hexane, 1: 4) of the residue gave **47b** as a white solid (1.48 g, 85% yield). Mp: 104–106 °C.

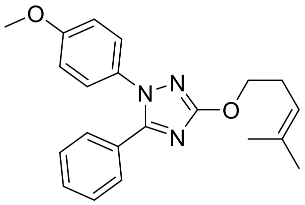

^{1}H NMR (400 MHz, $CDCl_3$) δ (ppm): 7.53–7.34 (m, 2H), 7.34–7.06 (m, 5H), 6.95–6.57 (m, 2H), 5.17 (t, J = 7.1 Hz, 1H), 4.23 (t, J = 7.1 Hz, 2H), 3.75 (s, 3H), 2.46 (q, J = 7.0 Hz, 2H), 1.61 (d, J = 19.0 Hz, 6H); ^{13}C NMR (101 MHz, $CDCl_3$) δ (ppm): 167.91, 159.72, 153.00, 134.43, 131.39, 129.93, 128.83, 128.51, 127.94, 127.07, 119.56, 114.58, 69.40, 55.62, 28.20, 25.83, 17.95. MS (ESI) *m*/*z*: 350.2 $[M+H]^+$. HRMS (ESI) *m*/*z* for $C_{21}H_{24}N_3O_2$ $[M+H]^+$: calc'd: 350.1869; found: 350.1871.

3-((3-(Bromofluoromethyl)-4-methylpentyl)oxy)-1-(4-methoxyphenyl)-5-phenyl-1*H*-1,2,4-triazole (47a). General procedure 4 with 1-(4-methoxyphenyl)-3-((4-methylpent-3-en-1-yl)oxy)-5-phenyl-1*H*-1,2,4-triazole followed by flash chromatography (ethyl acetate/*n*-hexane, 1: 4) gave **47a** as a colorless oil (84 mg, d.r. = 1/1, 45% yield).

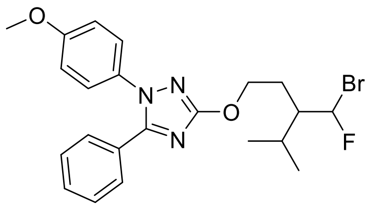

^{1}H NMR (400 MHz, $CDCl_3$) δ (ppm): 7.41 (d, *J* = 7.1 Hz, 2H), 7.35–7.11 (m, 5H), 6.83 (d, *J* = 8.9 Hz, 2H), 6.76–6.38 (m, 1H), 4.38 (dd, *J* = 11.9, 5.7 Hz, 12H), 3.76 (s, 3H), 2.32–1.82 (m, 4H), 1.16–0.71 (m, 6H); ^{19}F NMR (376 MHz, $CDCl_3$) δ (ppm): –135.13 (ddd, *J* = 73.1, 49.5, 19.1 Hz, 1F); ^{13}C NMR (101 MHz, $CDCl_3$) δ (ppm): 167.70, 159.79, 153.13, 131.32, 130.03, 128.86, 128.56, 127.85, 127.09, 114.63, 99.63 (dd, *J* = 256.2, 184.3 Hz), 68.17 (d, *J* = 9.8 Hz), 55.66, 49.47 (d, *J* = 16.2 Hz), 48.42 (d, *J* = 15.1 Hz), 29.40, 29.30 (d, *J* = 5.2 Hz), 27.22, 26.25 (d, *J* = 4.4 Hz), 20.60, 20.09 (d, *J* = 1.9 Hz), 19.57, 19.51 (d, *J* = 1.3 Hz). MS (ESI) *m*/z: 462.1 $[M+H]^+$. HRMS (ESI) *m/z* for $C_{22}H_{26}N_3O_2F^{79}Br$ $[M+H]^+$: calc'd: 462.1192; found: 462.1197.

3-((3-(Difluoromethyl)-4-methylpentyl)oxy)-1-(4-methoxyphenyl)-5-phenyl-1*H*-1,2,4-triazole (47). General procedure 7 with 1-(4-methoxyphenyl)-3-((4-methylpent-3-en-1-yl)oxy)-5-phenyl-1*H*-1,2,4-triazole followed by flash chromatography (ethyl acetate/*n*-hexane, 1: 4) gave **47** as a colorless oil (97 mg, 61% yield).

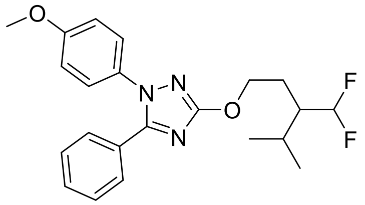

^{1}H NMR (400 MHz, $CDCl_3$) δ (ppm): 7.47–7.35 (m, 2H), 7.35–7.05 (m, 5H), 7.01–6.76 (m, 2H), 5.80 (td, *J* = 56.7, 3.0 Hz, 1H), 4.65–4.10 (m, 2H), 3.75 (s, 3H), 2.51–1.80 (m, 4H), 0.92 (dd, *J* = 6.4, 3.5 Hz, 6H); ^{19}F NMR (376 MHz, $CDCl_3$) δ (ppm): –120.43 (ddd, *J* = 281.5, 77.3, 56.1, 16.5 Hz, 2F); ^{13}C NMR (101 MHz, $CDCl_3$) δ (ppm): 167.79, 159.85, 153.18, 131.39, 130.08, 128.92, 128.62, 127.93, 127.15, 118.82 (t, *J* = 242.3 Hz), 114.69, 68.17, 55.71, 44.31 (t, *J* = 17.8 Hz), 27.32 (dd, *J* = 5.8, 3.2 Hz), 24.36 (t, *J* = 4.2 Hz), 19.97, 19.51. MS (ESI) *m/z*: 402.2 $[M+H]^+$. HRMS (ESI) *m/z* for $C_{22}H_{26}N_3O_2F_2$ $[M+H]^+$: calc'd: 402.1993; found: 402.1999.

3. Radiochemistry procedures

3.1. General HPLC methods

3.1.1. HPLC condition A

Column: Luna C18(2) (10 μm, 250 × 4.6 mm, 100 Å; Phenomenex).

The absorbance wavelength used for HPLC measurements was 254 nm.

Radioactivity was monitored using an in-line Flow-Count PMT radioactivity detector (Eckert & Ziegler).

Solvent A: aq. ammonium formate (50 mM); Solvent B: MeCN

Flow rate: 2 mL/min

0–5 min: 10 to 50% B

5–15 min: 50 to 90% B

15–20 min: 90% B

3.1.2. HPLC condition B

Column: Luna C18(2) (10 μm, 250 × 4.6 mm, 100 Å; Phenomenex).

The absorbance wavelength used for HPLC measurements was 254 nm.

Radioactivity was monitored using an in-line Flow-Count PMT radioactivity detector (Eckert & Ziegler).

Solvent A: H_2O; Solvent B: MeCN

Flow rate: 2 mL/min

0–12 min: 70% B

3.1.3. HPLC condition C

Column: Luna C18(2) (10 μm, 250 × 10.0 mm, 100 Å; Phenomenex).

The absorbance wavelength used for HPLC measurements was 254 nm.

Radioactivity was monitored using an in-line Flow-Count diode radioactivity detector (Eckert & Ziegler).

Solvent A: aq. ammonium formate (100 mM); Solvent B: MeCN

Flow rate: 5 mL/min

0–5 min: 10 to 50% B

5–40 min: 50 to 90% B

40–50 min: 90% B

3.1.4. HPLC condition D

Column: Luna C18(2) (10 μm, 250 × 4.6 mm, 100 Å; Phenomenex).

The absorbance wavelength used for HPLC measurements was 254 nm.

Radioactivity was monitored using an in-line Flow-Count PMT radioactivity detector (Eckert & Ziegler).

Solvent A: aq. ammonium formate (100 mM); Solvent B: MeOH

Flow rate: 2 mL/min

0–5 min: 10 to 30% B

5–60 min: 30 to 80% B

3.2. General procedures for radiofluorination

3.2.1. Preparation of anhydrous [^{18}F]fluoride/Et_4NHCO_3 solution

[^{18}F]Fluoride ion was produced from a cyclotron (PETtrace; GE Healthcare) according to the ^{18}O(p,n)^{18}F reaction by irradiating ^{18}O-enriched water (3 mL, 98 atom%) with a proton beam (35 MeV; 35-45 μA) for at least 45 min. [^{18}F]fluoride ion (2.96–4.44 GBq) in [^{18}O]water (200–400 μL) and a aqueous solution of Et_4NHCO_3 (50 μL, 7.5 mg) were loaded into a glassy carbon reactor of a fully automated apparatus (TRACERlab^{TM} FX2 NFX2N; GE Healthcare). MeCN (2.5 mL) was added, and the solvent was azeotropically removed at 80–100 °C under a stream of nitrogen that was vented to vacuum. This step was repeated after a second addition of MeCN (2.5 mL). The [^{18}F]fluoride/Et_4NHCO_3 reagent was cooled to 40 °C before addition of anhydrous MeCN (3.0 mL). The mixture was transferred out of the reactor under He gas pressure into a vial to yield a solution of [^{18}F]fluoride /Et_4NHCO_3 (0.74–1.85 GBq) in MeCN. An aliquot of this solution (200 μL) was then used for manual methodology experiments.

3.2.2. Radiofluorination of different precursors at set temperatures

To a 1-mL V-vial was added a solution of [^{18}F]fluoride/Et_4NHCO_3 in MeCN (200 μL), followed by a solution of precursor (10 μmol) in MeCN (300 μL). The reaction vial was maintained with good air-tightness to prevent the loss of MeCN. The reaction was conducted at a set temperature

for 10 min, then quenched with H_2O (300 µL). An aliquot of the reaction mixture was analyzed with HPLC using HPLC condition A.

Supplementary Table 2. Radiofluorination of different precursors at different temperatures.

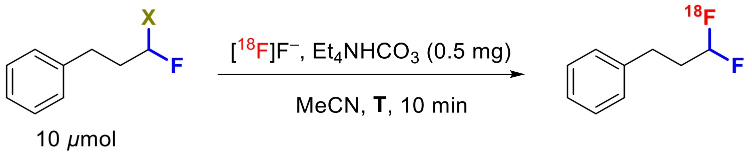

T (°C)	Yield (%) X = F	X = Cl	X = Br	X = I
40	0	trace	5 ± 1	6 ± 1
60	0	trace	19 ± 2	13 ± 1
80	0	1 ± 0	34 ± 4	18 ± 3
100	0	1 ± 0	41 ± 2	19 ± 3
120	0	7 ± 3	61 ± 8	20 ± 1
140	-	-	62 ± 1	-
160	-	-	64 ± 2	-

3.2.3. General procedure 8 for manual substrates scope evaluation

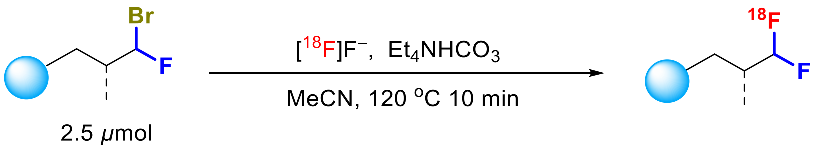

To a 1-mL glass V-vial is added a solution of azeotropically dried [^{18}F]fluoride (50–100 MBq) plus Et_4NHCO_3 (0.5 mg) in MeCN (200 μL), followed by a solution of substrate (**3**, **6a-47a**; 2.5 μmol) in MeCN (300 μL). The reaction vial is maintained with airtightness to prevent the loss of MeCN. The reaction is conducted at a selected temperature for 10 min and quenched with H_2O (300 µL). An aliquot is then analyzed using HPLC condition A. Yields is based on HPLC chromatogram peak areas. All yields are decay-corrected and expressed as mean ± SD ($n \geq 2$).

Radioactive products are collected at least once for each substrate to verify that HPLC yields match isolated yield.

3.2.4. General procedure 9 for automated radiosynthesis

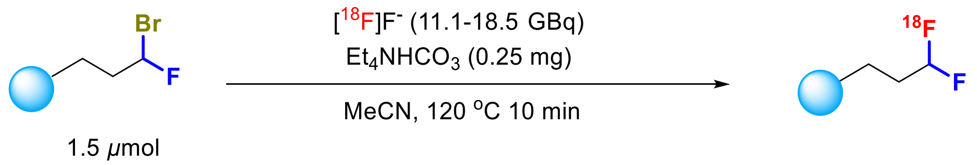

The radiosyntheses of the [18F]difluoromethylalkanes ([18F]**10**, [18F]**19**, [18F]**25**, [18F]**36** and [18F]**39)** were performed on a fully automated apparatus (TRACERlab™ FX2 N; GE Healthcare), according to the following procedure. [18F]Fluoride ion (11.1–18.5 GBq) in [18O]water (400–700 μL) and a solution of aqueous Et_4NHCO_3 (50 μL, 5 mg/mL) are loaded into a glassy carbon vial reactor of the apparatus. MeCN (2.5 mL) is added, and the solvent azeotropically removed at 80–100 °C under a stream of nitrogen gas that is vented to vacuum. This step is repeated after a second addition of MeCN (2.5 mL). The reactor is then cooled to 40 °C and a solution of precursor (**10a**, **19a**, **25a**, **36a** and **39a**; 1.5 μmol) in anhydrous MeCN (0.6 mL) is added. The reaction mixture is heated for 10 min at 120 °C. The reactor is then cooled to 40 °C. The contents are diluted with H_2O (2.5 mL), transferred into an intermediate vial, and then delivered to a 5-mL HPLC loop. Under trigger of a fluid detector, the contents are injected onto a semi-preparative HPLC column [Luna C18(2), 250 × 10 mm i.d., 10 μm; Phenomenex], and eluted with a gradient of acetonitrile and 100 mM aqueous ammonium formate at a flow rate of 5 mL/min as described (HPLC condition C). The eluate is monitored by first a UV absorbance detector (absorbance at 254 nm) and then a radioactivity detector. The desired product fraction is collected into a round-bottomed flask preloaded with water (40 mL). The contents are then passed through a Sep-Pak plus short C18 cartridge (Waters) preconditioned with ethanol (10 mL) and then water (10 mL). The Sep-Pak cartridge is washed with water (10 mL), and eluted sequentially with ethanol (1 mL) and saline (9 mL) into a final vial. An aliquot of this formulation is used for further analyses, such as determination of radiochemical purity, identity and molar activity.

3.3. Molar activity determination

3.3.1. Basic principle of molar activity determination

To determine molar activity, we first established a calibration curve using a series of known amounts of the reference compound. On this curve, the X-axis represents the amount of the reference compound (X), with values between 20 ng to 500 ng. The Y-axis denotes the UV absorbance peak area at 254 nm (Y; in arbitrary units × min), measured by HPLC. A linear regression fit provided a calibration equation (*Y*=*KX*), establishing a direct relationship between absorbance peak area and compound quantity. In the experimental analysis, we calculated the molar activity of the ^{18}F-labeled product by applying its radioactivity (GBq), molecular weight (M) and the UV peak area measured via HPLC to the following formula.

$$Carrier\ (X) = \frac{UV\ peak\ area\ (Y)}{K}\ \text{(ng)}$$

$$A_{\text{m}} = \frac{Activity}{Carrier + radioactive\ compound} \approx \frac{Activity}{Carrier} = \frac{Activity \times K \times M \times 1000}{UV\ peak\ area\ (Y)}\ \text{(GBq/}\mu\text{mol)}$$

3.3.2. Optimization for molar activity measurement

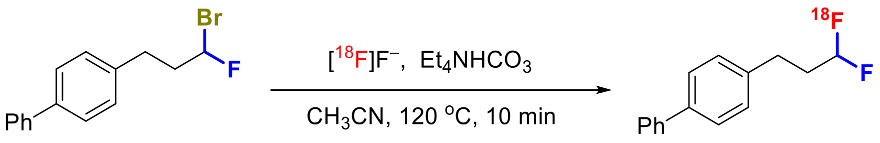

[^{18}F]**10**

The radiofluorination followed general procedure 9. ‘Semi-preparative’ purification was carried out using HPLC condition C. Analytical HPLC for [^{18}F]**10** was carried out using HPLC condition B.

quantity (ng)	20	40	60	80	100
UV peak area	55859	113647	170316	226798	284771

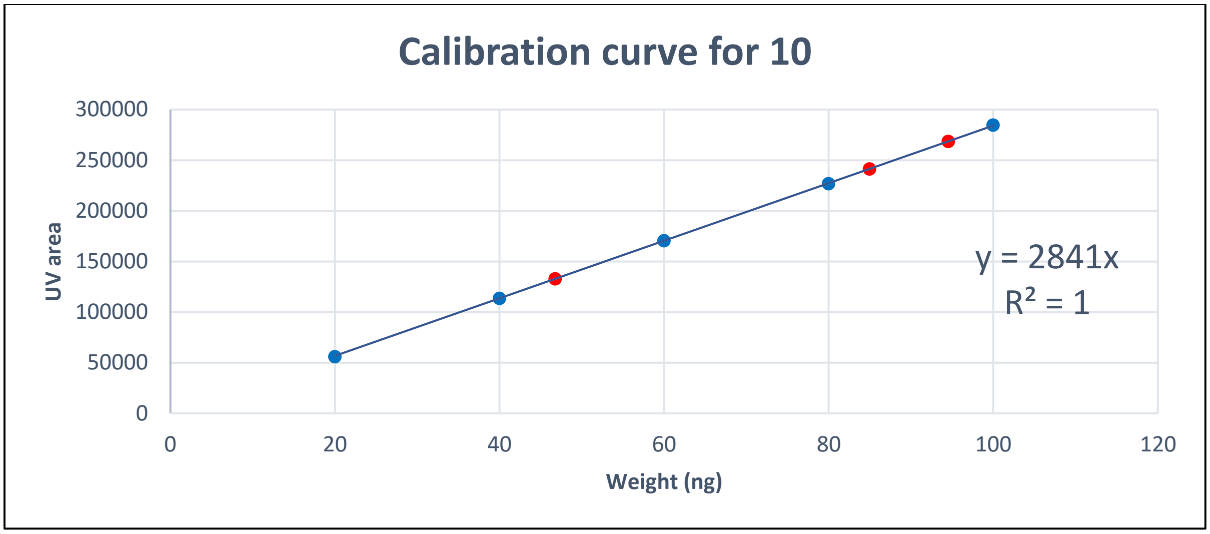

Supplementary Figure 12. Calibration curve for authentic reference **10** for determination of the molar activity of **[**[18]F]**10.** Data points for the calibration curve are in blue. Carrier amounts in [^{18}F]**10** analytes from Supplemental Table 4 are shown in red. Analytical HPLC condition B was used.

Supplementary Table 3. Optimization for radiosynthesis of [^{18}F]**10** under different conditions. AY = activity yield. n.d.c. = non-decay-corrected.

Entry	Precursor (μmol)	Et_4NHCO_3 (mg)	AY (n.d.c.) (GBq)
1	2.5	0.5	1.15
2	2.5	0.25	1.22
3	1.5	0.25	1.22

Supplementary Table 4. Molar activity (A_m) of [^{18}F]**10** under different conditions. EOS = end of radiosynthesis.

Entry	Activity (GBq)	UV peak area	Amount of carrier (ng)	M	A_m (EOS) (GBq/μmol)
1	0.01343	268551	94.53	232.27	33
2	0.01878	241448	84.99	232.27	51
3	0.01499	132808	46.75	232.27	74

3.3.3. Automated radiosyntheses

*3.3.3.1. Automated radiosynthesis of [^{18}F]**10***

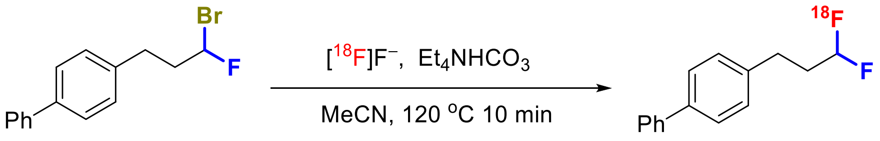

[^{18}F]**10**

The automated radiosynthesis followed general procedure 9 with 4-(3-bromo-3-fluoropropyl)-1,1'-biphenyl (1.5 μmol, 0.44 mg) and Et_4NHCO_3 (0.25 mg). The semi-preparative HPLC purification was carried out using HPLC condition C. Analytical HPLC for [^{18}F]**10** was carried out using condition B.

Quantity (ng)	20	40	60	80	100
UV peak area	55859	113647	170316	226798	284771

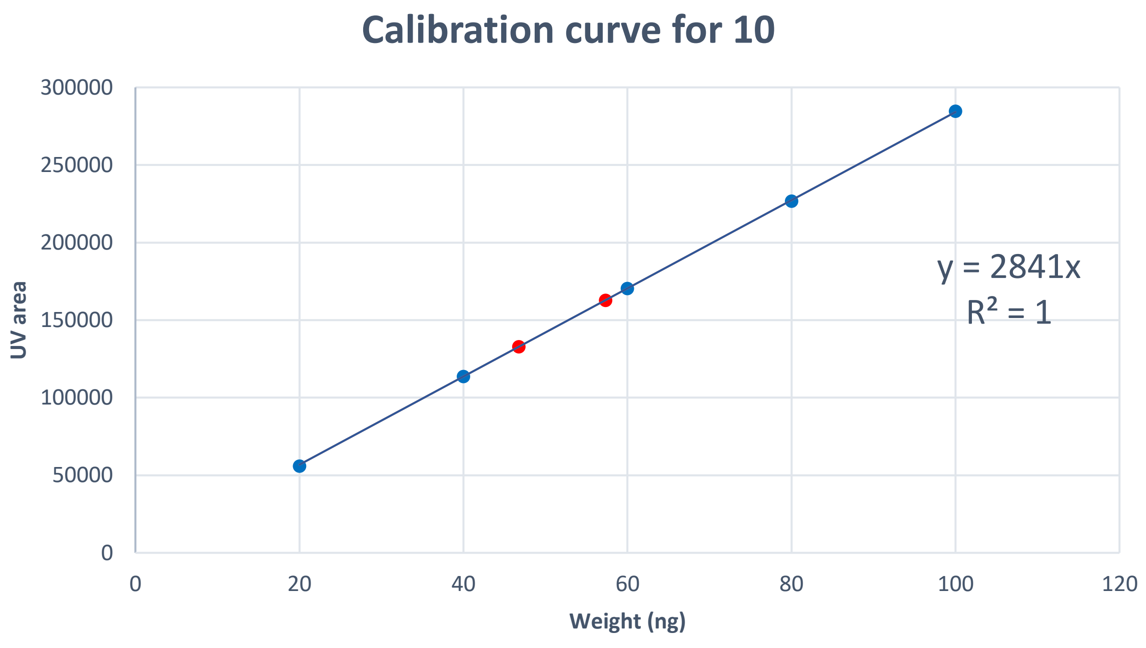

Supplementary Figure 13. Calibration curve for authentic reference **10** to determine molar activity with data for molar activity determinations added. Data points for the calibration curve are in blue. Experimental carrier amount data points from Supplementary Table 6 for two molar activity measurements are in red. Analytical HPLC condition B was used.

**Supplementary Table 5.** Radiosynthesis of [^{18}F]**10**. AY = activity yield. n.d.c. = non-decay corrected.				
Entry	Starting activity (GBq)	Radiosynthesis time (min)	Purity (%)	AY (n.d.c.) (GBq)
1	14.5	66	>99	1.22
2	17.8	62	>99	1.04
Average				1.13 ± 0.13

**Supplementary Table 6.** The molar activity determination of [^{18}F]10. EOS = end of radiosynthesis. ERP = end of radionuclide production.						
Entry	Activity (GBq)	UV peak area	Amount of carrier (ng)	M	A_m (EOS) (GBq/μmol)	A_m (ERP) (GBq/μmol)
1	0.01499	132808	46.75	232.27	74	127
2	0.01500	162840	57.32	232.27	61	106
Average					68 ± 9	117 ± 15

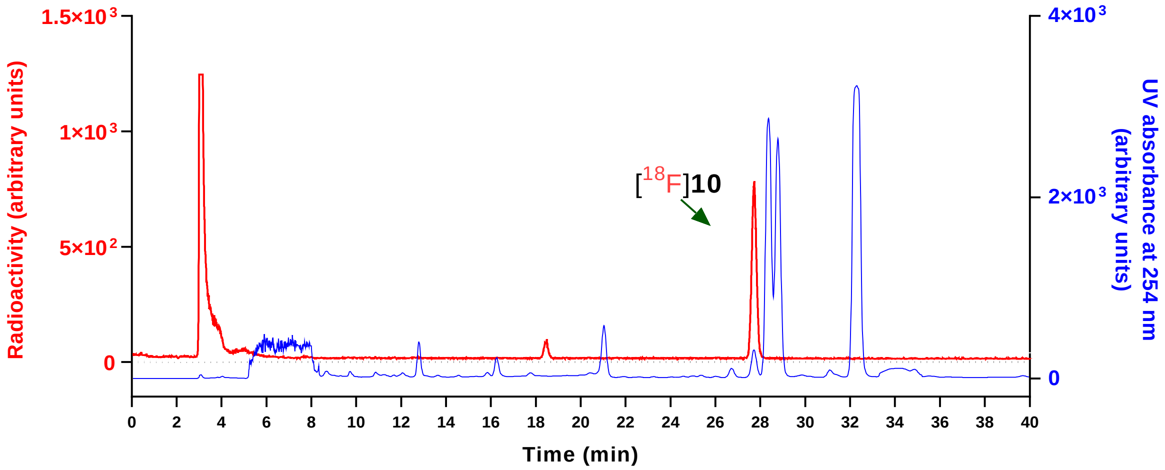

Supplementary Figure 14. Semi-preparative HPLC (condition C) chromatogram for the purification of [^{18}F]**10**.

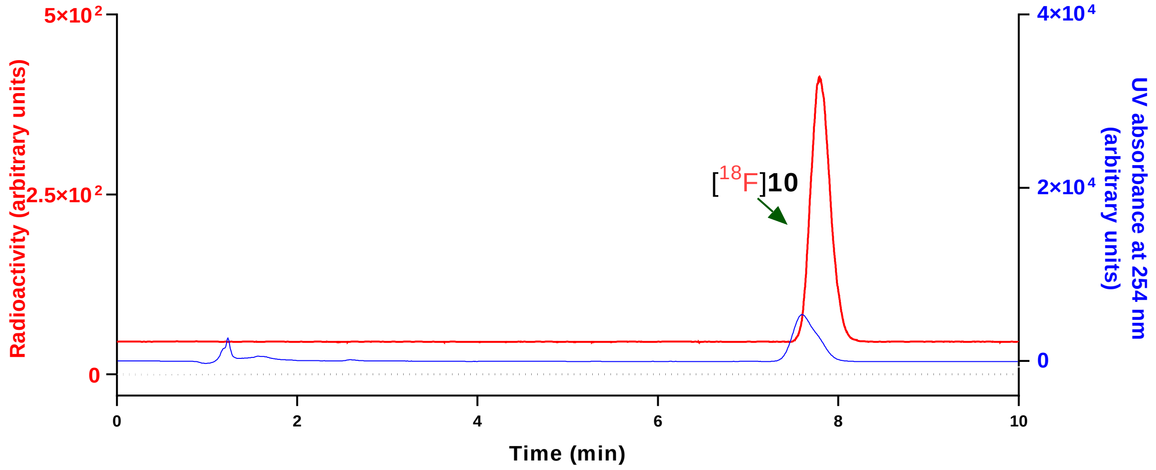

Supplementary Figure 15. Analytical HPLC (condition B) chromatogram for QC of [^{18}F]**10**.

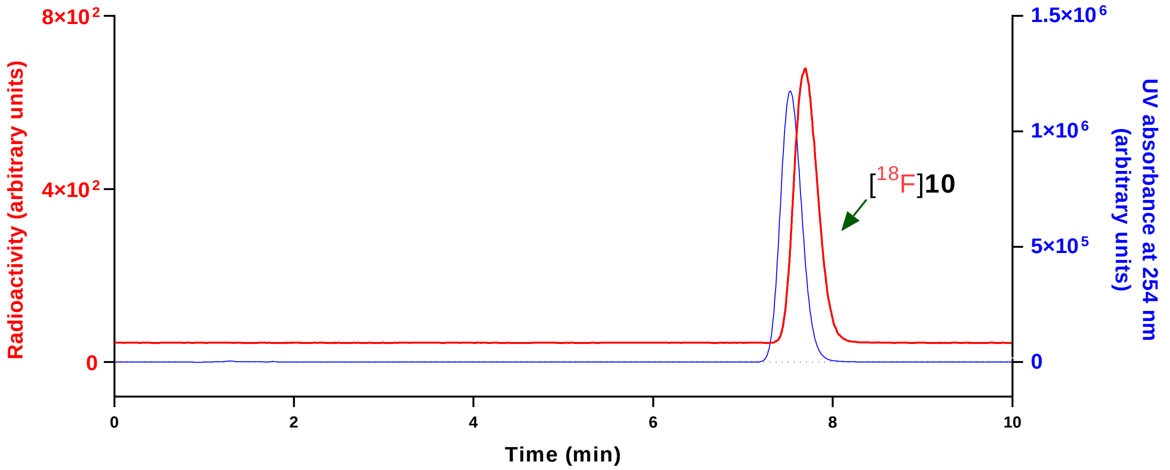

Supplementary Figure 16. Analytical HPLC (condition B) chromatogram for [^{18}F]**10** with co–injected **10.**

*3.3.3.2. Automated radiosynthesis of [^{18}F]**19***

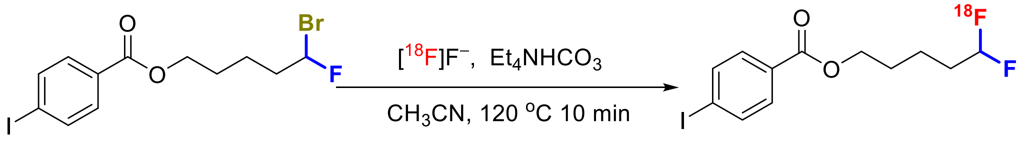

[^{18}F]**19**

The automated radiosynthesis followed general procedure 9 with 5-bromo-5-fluoropentyl 4-iodobenzoate (1.5 μmol, 0.62 mg) and Et_4NHCO_3 (0.25 mg). The semi-preparative HPLC purification was carried out using HPLC condition C. Analytic HPLC for [^{18}F]**19** was carried out using HPLC condition B.

Quantity (ng)	20	40	60	80	100	120	140
UV peak area	24915	56282	82828	112932	134452	162145	186677

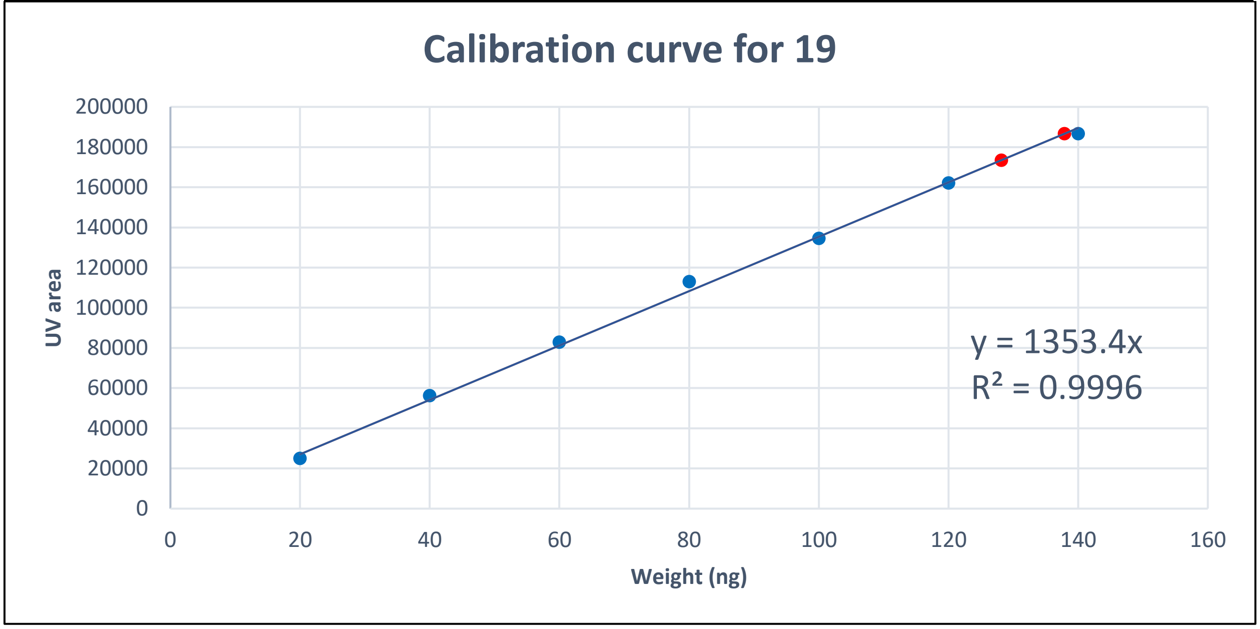

Supplementary Figure 17. Calibration curve for authentic reference **19** to determine molar activity with data for molar activity determinations added. Data points for the calibration curve are in blue. Experimental carrier amount data points from Supplementary Table 8 for molar activity measurements are shown in red. Analytical HPLC condition B was used.

**Supplementary Table 7.** Radiosynthesis of [^{18}F]19. AY = activity yield. n.d.c. = non-decay-corrected.				
Entry	Starting activity (GBq)	Radiosynthesis time (min)	Purity (%)	AY (n.d.c.) (GBq)
1	15.7	70	>99	0.74
2	15.7	67	>99	0.96
Average				0.85 ± 0.16

**Supplementary Table 8.** The molar activity determination of [^{18}F]**19**. EOS = end of radiosynthesis. ERP = end of radionuclide production.						
Entry	Activity (GBq)	UV peak area	Amount of carrier (ng)	M	A_m (EOS) (GBq/μmol)	A_m (ERP) (GBq/μmol)
1	0.02725	173479	128.18	354.14	75	134
2	0.03364	186605	137.88	354.14	86	136
Average					81 ± 8	135 ± 1

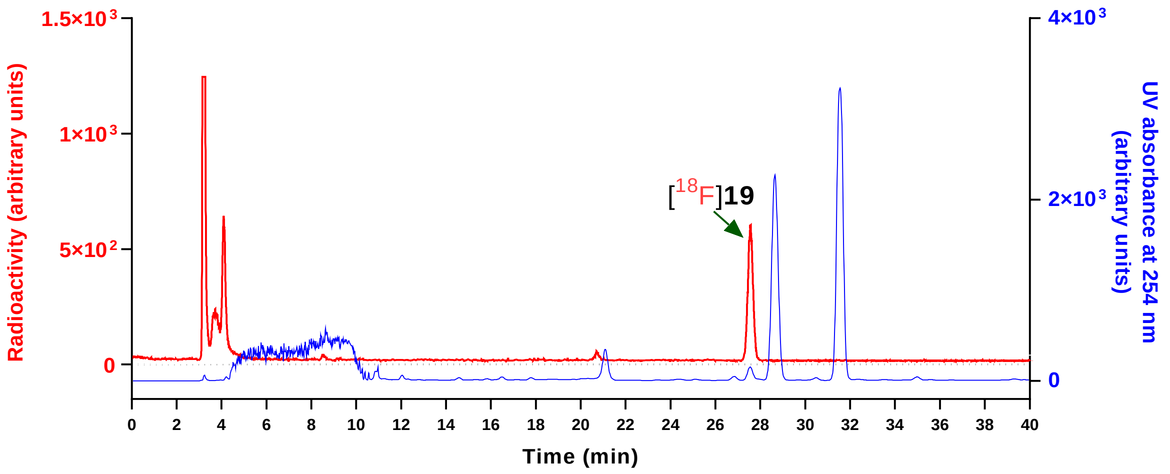

Supplementary Figure 18. Semi-preparative HPLC (condition C) chromatogram for purification of [^{18}F]**19**.

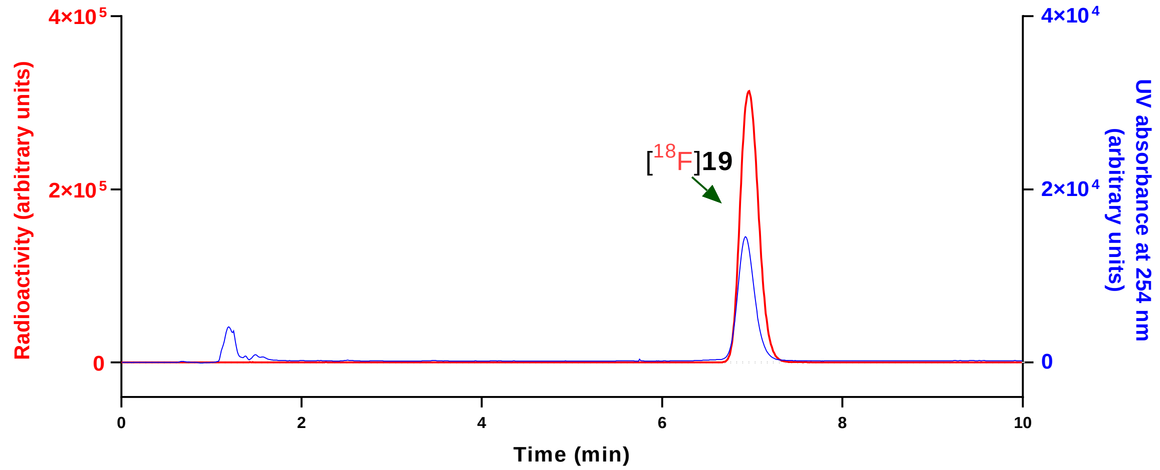

Supplementary Figure 19. Analytical HPLC chromatogram (condition B) for [^{18}F]**19.**

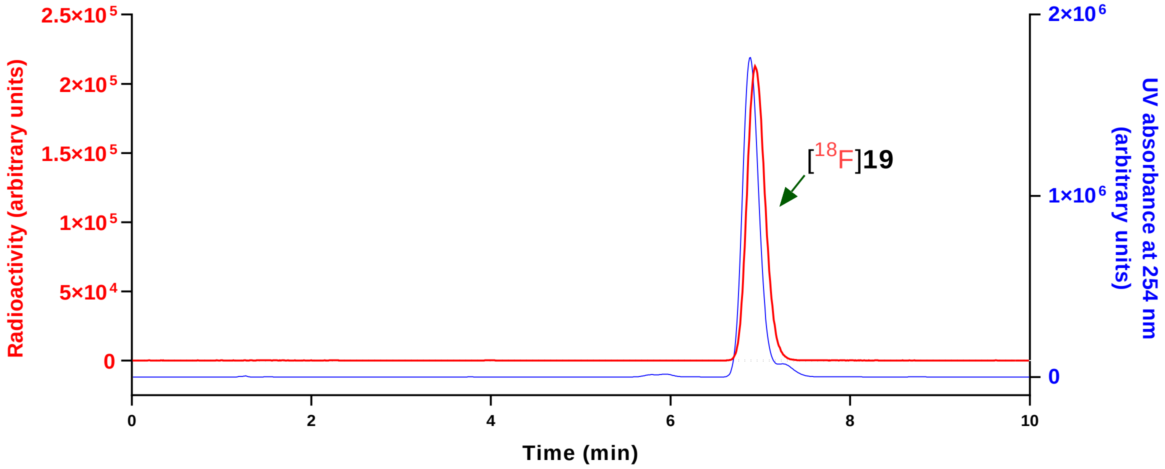

Supplementary Figure 20. Analytical HPLC (condition B) chromatogram for [^{18}F]**19** with co–injected **19**.

*3.3.3.3. Automated radiosynthesis of [18F]**25***

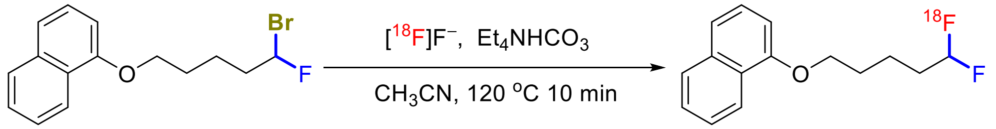

[18F]**25**

The automated radiosynthesis followed general procedure 9 with 1-((5-bromo-5-fluoropentyl)oxy)naphthalene (1.5 μmol, 0.47 mg) and Et_4NHCO_3 (0.25 mg). The semi-preparative HPLC purification was carried out using HPLC condition C. Analytical HPLC for [18F]**25** was carried out using HPLC condition B.

Quantity (ng)	40	60	80	100	120
UV peak area	5742	9050	11836	14839	17416

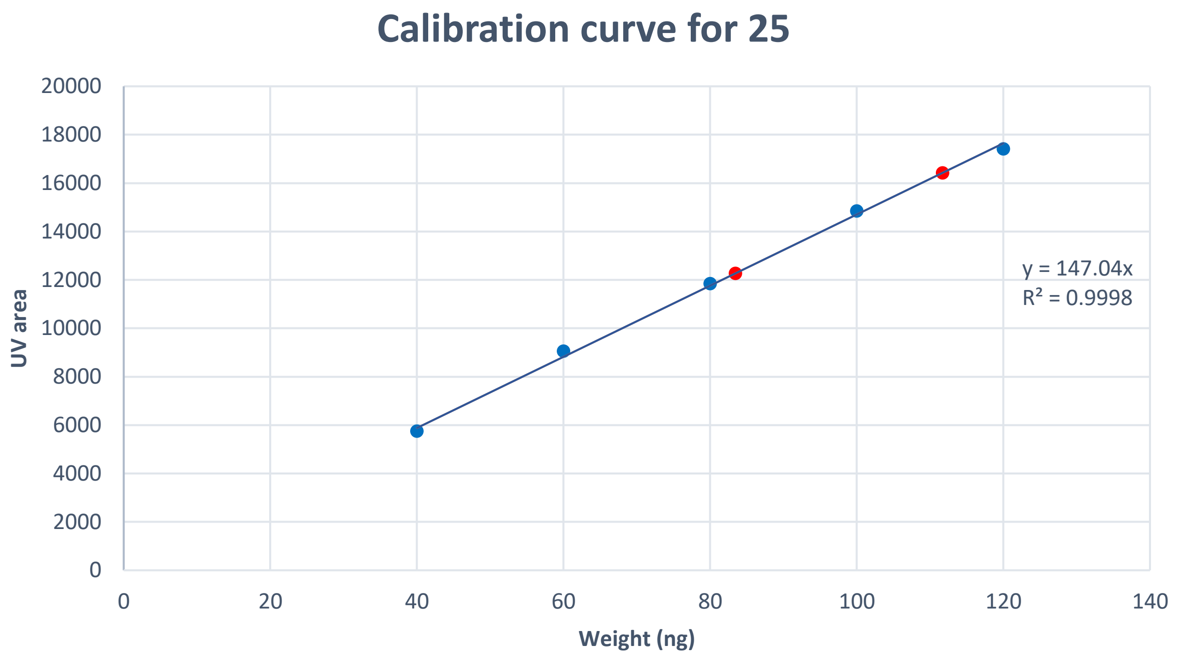

Supplementary Figure 21. Calibration curve for authentic reference **25** with added data for molar activity determinations. Data points for the calibration curve are in blue. Experimental carrier amount data points from Supplementary Table 10 for molar activity measurement are in red.

**Supplementary Table 9.** Radiosynthesis of [^{18}F]**25**. AY = activity yield. n.d.c. = non-decay-corrected.				
Entry	Starting activity (GBq)	Radiosynthesis time (min)	Purity (%)	AY (n.d.c.) (GBq)
1	17.9	68	>99	1.26
2	15.7	68	>99	0.81
Average				1.04 ± 0.32

**Supplementary Table 10.** The molar activity determination of [^{18}F]**25**. EOS = end of radiosynthesis. ERP = end of radionuclide production.						
Entry	Activity (GBq)	UV peak area	Amount of carrier (ng)	M	A_m (EOS) (GBq/μmol)	A_m (ERP) (GBq/μmol)
1	0.03297	16424	111.70	250.29	74	123
2	0.02187	12269	83.44	250.29	66	110
Average					70 ± 6	117 ± 9

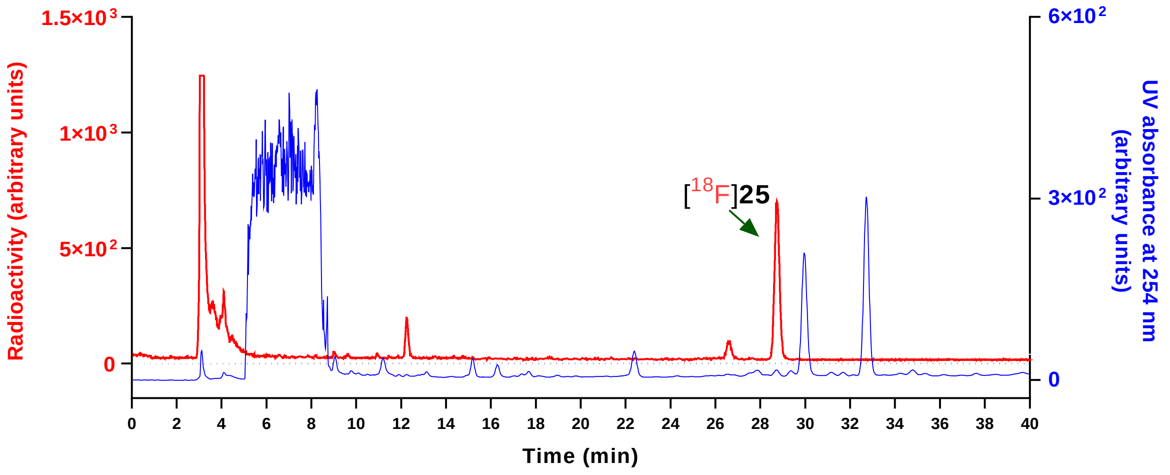

Supplementary Figure 22. Semi-preparative HPLC (condition C) chromatogram for purification of [^{18}F]**25**.

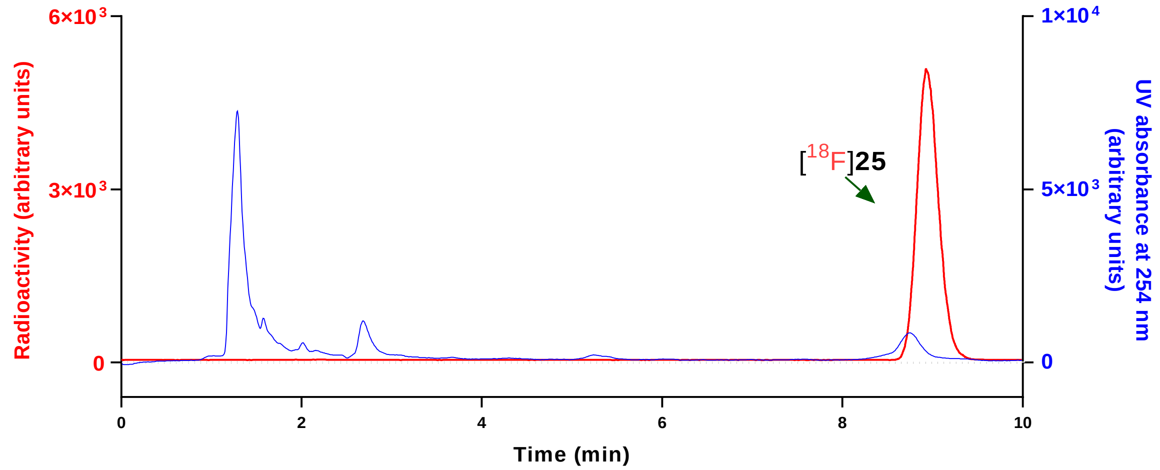

Supplementary Figure 23. Analytical HPLC (condition B) chromatogram for QC of [^{18}F]**25.**

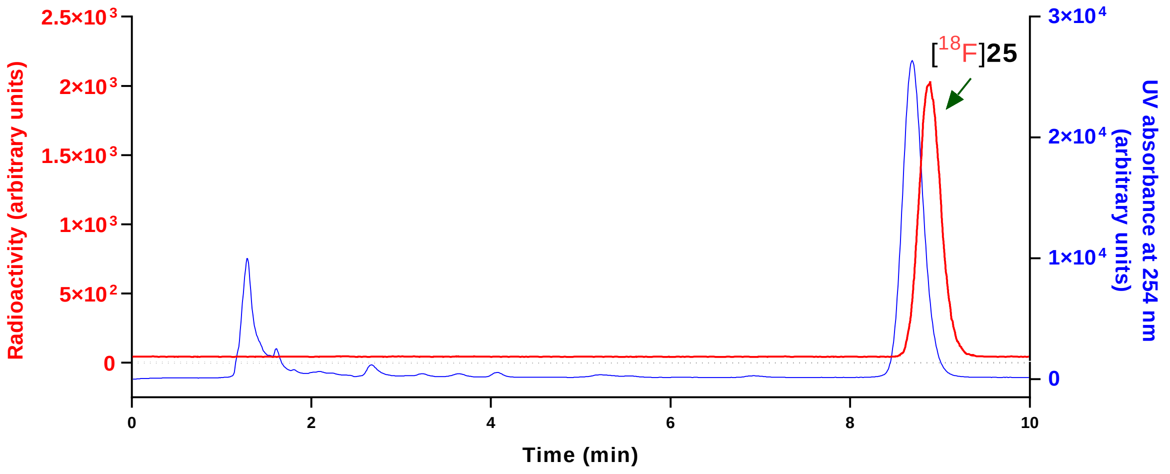

Supplementary Figure 24. Analytical HPLC (condition B) chromatogram for [^{18}F]**25** with co–injected **25**.

*3.3.3.4. Automated radiosynthesis of [^{18}F]**36***

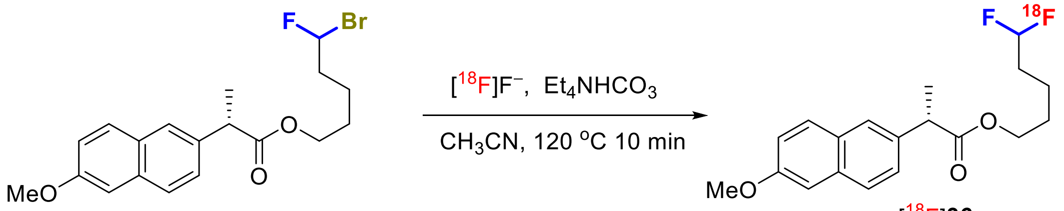

[^{18}F]**36**

The automated radiosynthesis followed General procedure 9 with 5-bromo-5-fluoropentyl (2*S*)-2-(6-methoxynaphthalen-2-yl)propanoate (1.5 μmol, 0.60 mg) and Et_4NHCO_3 (0.25 mg). The semi-preparative HPLC purification was carried out using HPLC condition C. Analytical HPLC for [^{18}F]**36** was carried out using HPLC condition B.

Quantity (ng)	20	40	60	80	100
UV peak area	6961	15390	23327	30662	39607
Quantity (ng)	120	140	160	180	200
UV peak area	47375	55296	63010	70801	79243

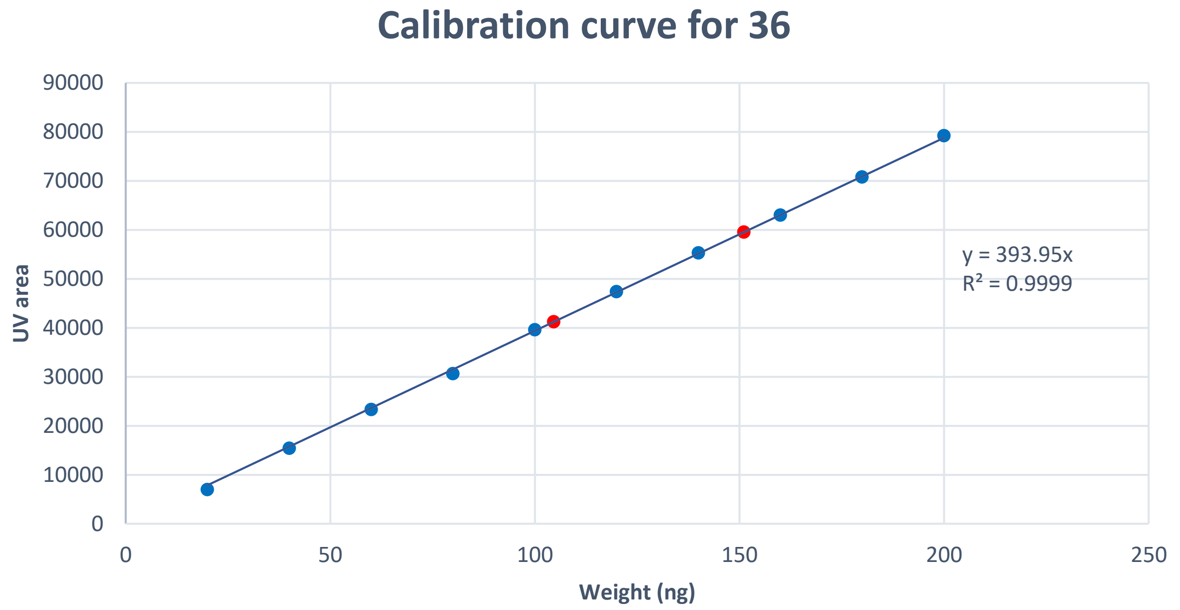

Supplementary Figure 25. Calibration curve for authentic reference **36** to determine molar activity with data for molar activity determinations added. Data points for the calibration curve are in blue. Experimental carrier amount data points from Supplementary Table 12 for molar activity measurement are in red.

Supplementary Table 11. Radiosynthesis of [^{18}F]**36**. AY = activity yield. n.d.c. = non-decay corrected.

Entry	Starting activity (GBq)	Radiosynthesis time (min)	Purity (%)	AY (n.d.c.) (GBq)
1	18.8	71	>99	1.18
2	15.7	66	>99	1.30
Average				1.24 ± 0.08

Supplementary Table 12. The molar activity determination of [^{18}F]36. EOS = end of radiosynthesis. ERP = end of radionuclide production.

Entry	Activity (GBq)	UV peak area	Amount of carrier (ng)	M	A_m (EOS) (GBq/μmol)	A_m (ERP) (GBq/μmol)
1	0.04993	59524	151.10	336.38	111	189
2	0.02537	41229	104.66	336.38	82	137
Average					97 ± 21	163 ± 37

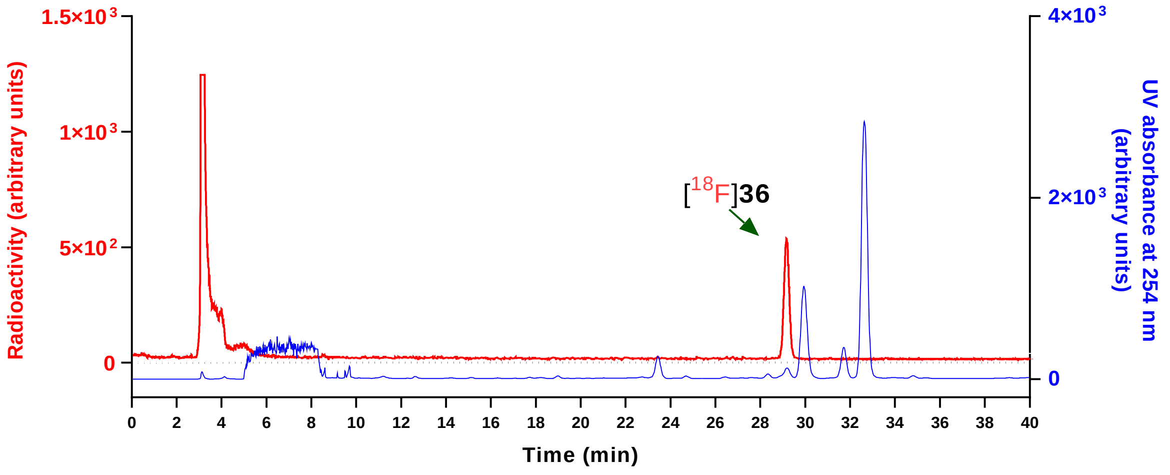

Supplementary Figure 26. Semi-preparative (condition C) HPLC chromatogram for purification of [^{18}F]**36**.

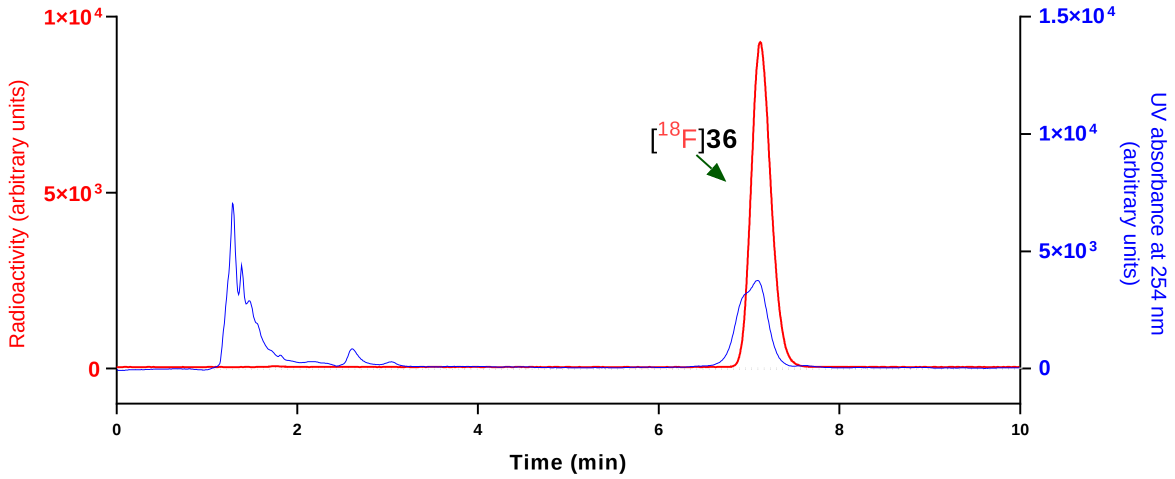

Supplementary Figure 27. Analytical HPLC (condition B) chromatogram for QC of [^{18}F]**36.**

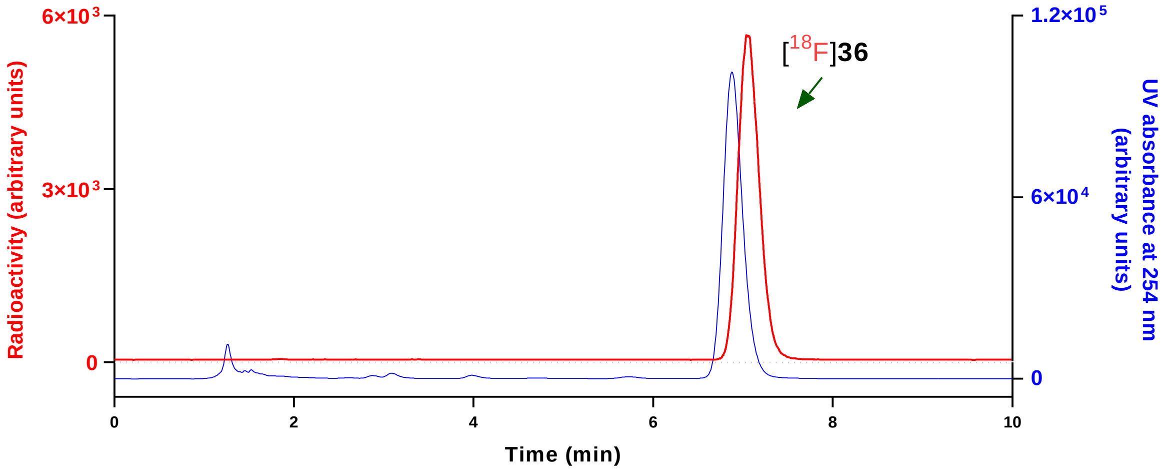

Supplementary Figure 28. Analytical HPLC (condition B) chromatogram for [^{18}F]**36** with co–injected **36.**

*3.3.3.5. Automated radiosynthesis of [^{18}F]**39***

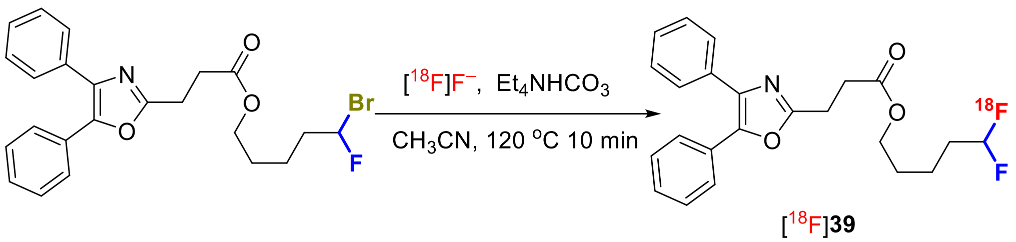

The automated radiosynthesis followed general procedure 9 with 5-bromo-5-fluoropentyl 3-(4,5-diphenyloxazol-2-yl)propanoate (1.5 μmol, 0.69 mg) and Et_4NHCO_3 (0.25 mg). The semi-preparative HPLC purification was carried out using HPLC condition C. Analytical HPLC for [^{18}F]**39** was carried out using HPLC condition B.

Quantity (ng)	20	40	60	80	100
UV peak area	10090.5	20040	29996.5	40000	50441.5
Quantity (ng)	200	300	400	500	
UV peak area	101639	157859	209989	262947	

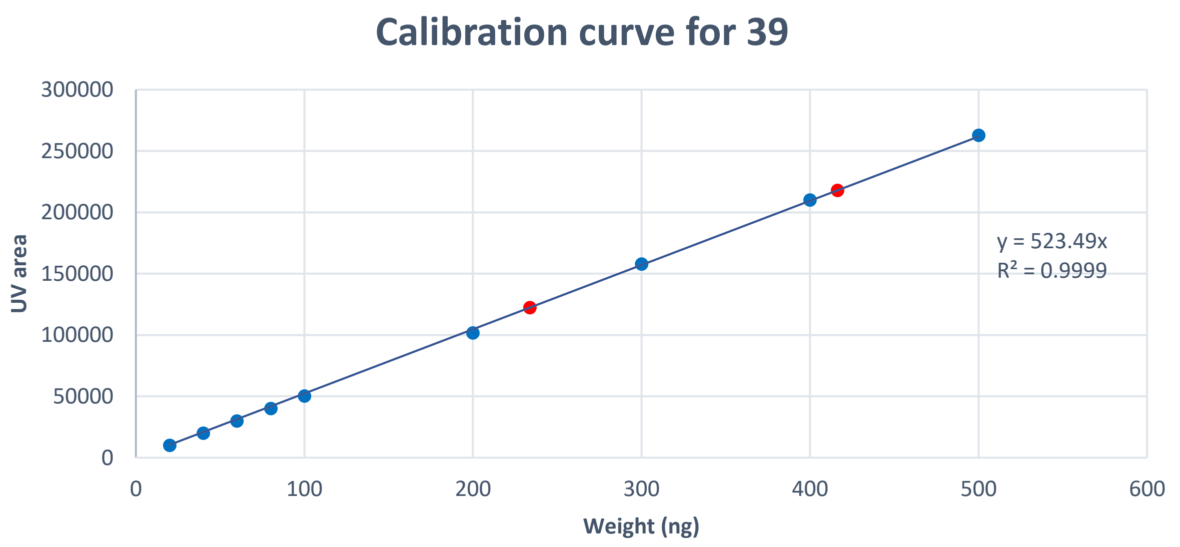

Supplementary Figure 29. Calibration curve for authentic reference **39** to determine molar activity with data for molar activity determinations added. Data points for the calibration curve are in blue. Experimental carrier amount data points from Supplemental Table 14 for A_m measurement are in red.

**Supplementary Table 13.** Radiosynthesis of [^{18}F]**39**. AY = activity yield. n.d.c. = non-decay corrected.				
Entry	Starting activity (GBq)	Radiosynthesis time (min)	Purity (%)	AY (n.d.c.) (GBq)
1	14.2	71	>99	1.07
2	14.7	68	>99	1.30
Average				1.19 ± 0.16

**Supplementary Table 14.** The molar activity determination of [^{18}F]**39**. EOS = end of radiosynthesis. ERP = end of radionuclide production.						
Entry	Activity (GBq)	UV peak area	Amount of carrier (ng)	M	A_m (EOS) (GBq/μmol)	A_m (ERP) (GBq/μmol)
1	0.03924	217938	416.32	399.44	38	62
2	0.02105	122320	233.66	399.44	36	60
Average					37 ± 1	61 ± 1

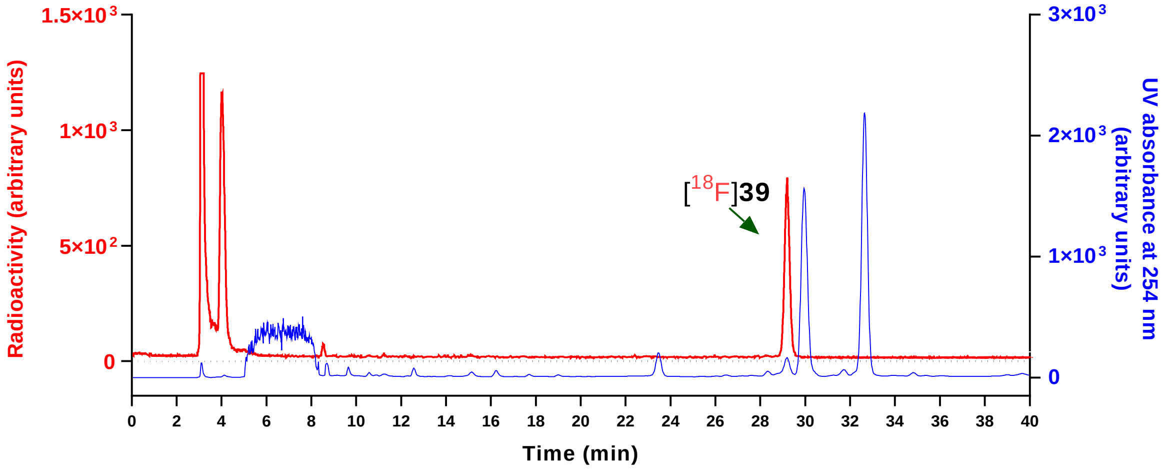

Supplementary Figure 30. Semi-preparative HPLC (condition C) chromatogram for purification of [^{18}F]**39**.

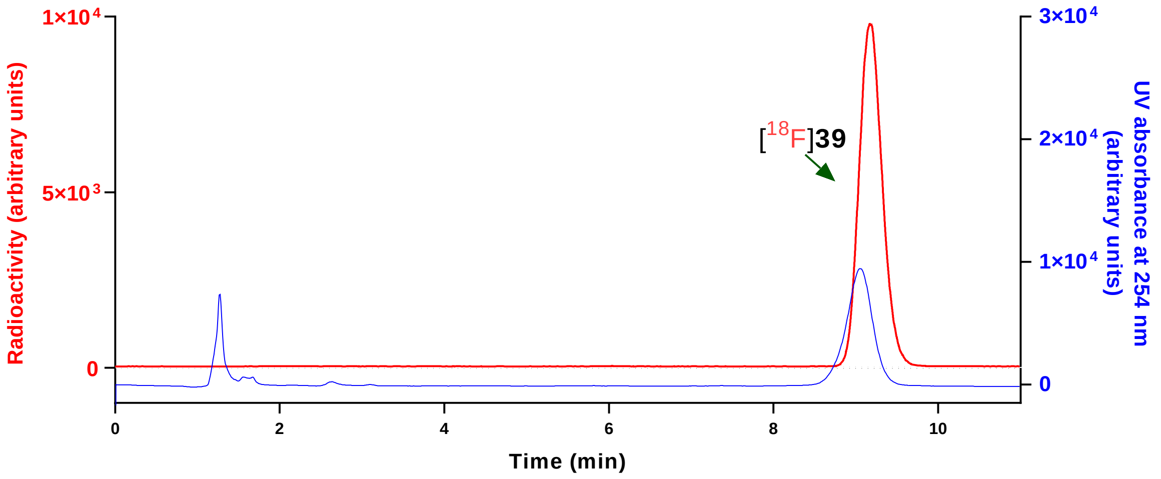

Supplementary Figure 31. Analytical HPLC (condition B) chromatogram for QC of [^{18}F]**39.**

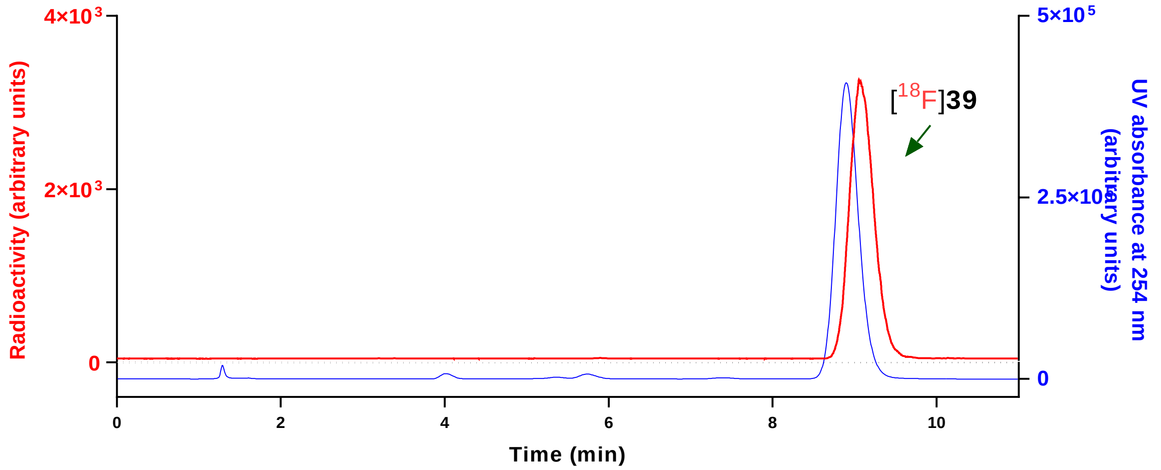

Supplementary Figure 32. Analytical HPLC (condition B) chromatogram for [^{18}F]**39** with co–injected **39**.

3.4. Mechanism study

3.4.1. Control experiment

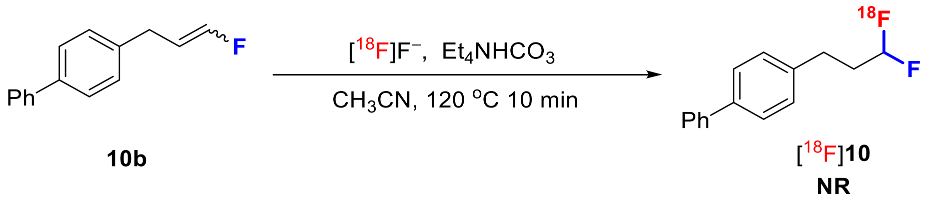

To a 1-mL glass V-vial was added a solution of [^{18}F]fluoride/Et_4NHCO_3 in MeCN (200 μL), followed by a solution of (*E*/*Z*)-4-(3-fluoroallyl)-1,1'-biphenyl (**10b**; 2.5 μmol) in MeCN (300 μL). The reaction vial was maintained with good air tightness to prevent the loss of MeCN. The reaction was conducted at 120 °C for 10 min, quenched with H_2O (300 µL). An aliquot of the reaction mixture was then analyzed using HPLC condition A. The HPLC chromatogram is shown below. No reaction (NR) was observed.

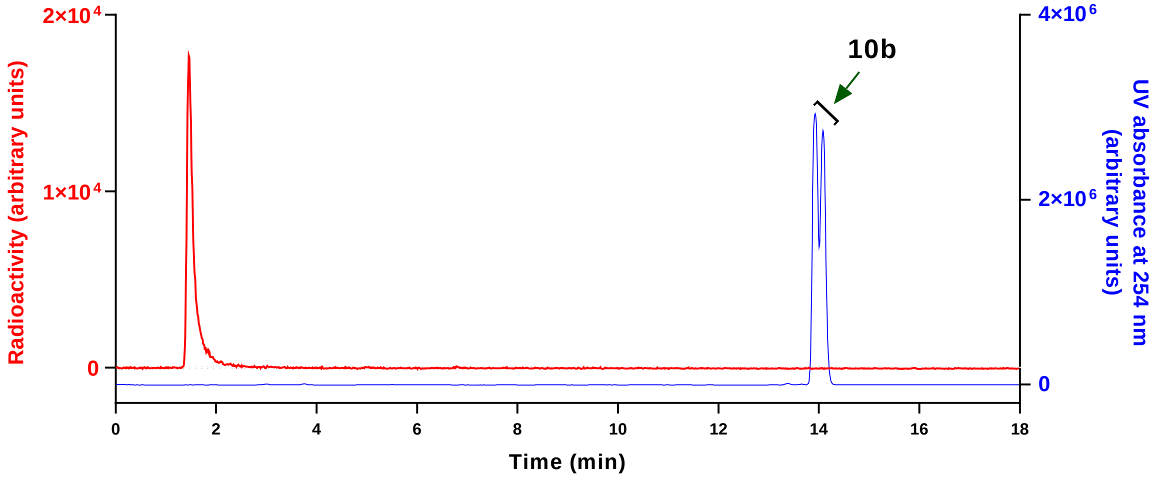

Supplementary Figure 33. Chromatogram for HPLC analysis (condition A) control radiofluorination reaction with **10b** (*E*/*Z*)-4-(3-fluoroallyl)-1,1'-biphenyl).
.

3.4.2. Investigation of the potential formation of the vinyl bromide 10c in the radiofluorination of 10a

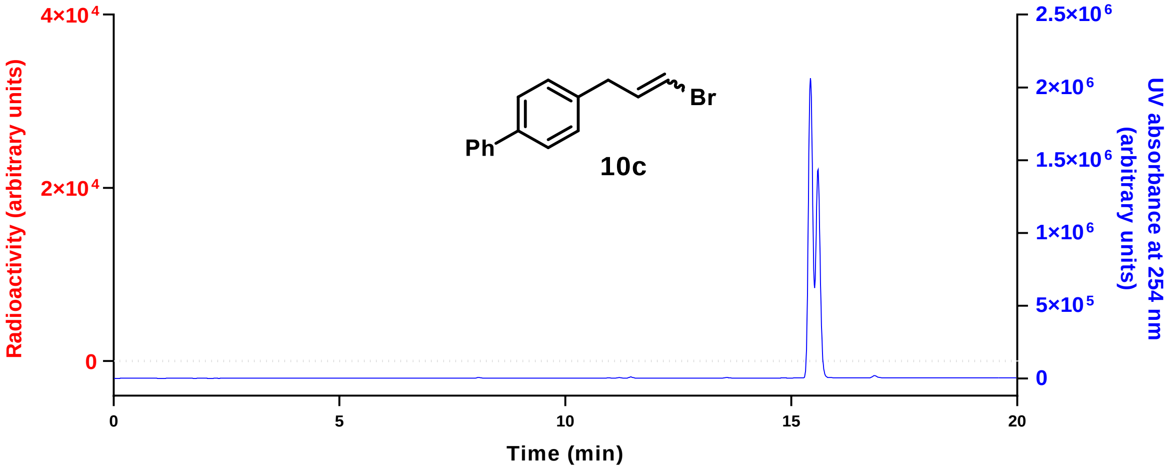

Supplementary Figure 34. Analytical HPLC chromatogram (condition A) for reference compound **10c** (*E* and *Z* isomer mixture).

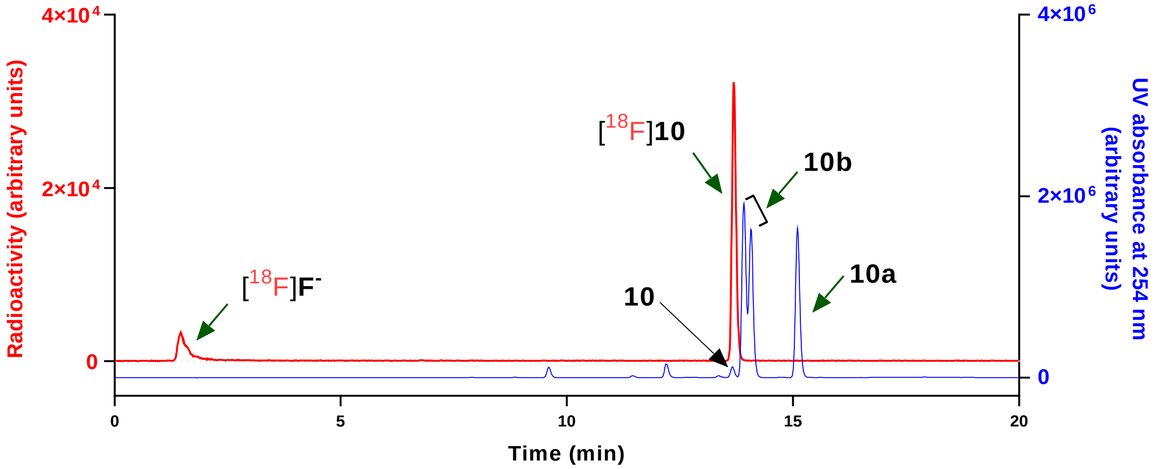

Supplementary Figure 35. Analytical HPLC chromatogram (condition A) for unpurified [^{18}F]**10** produced from **10a**.

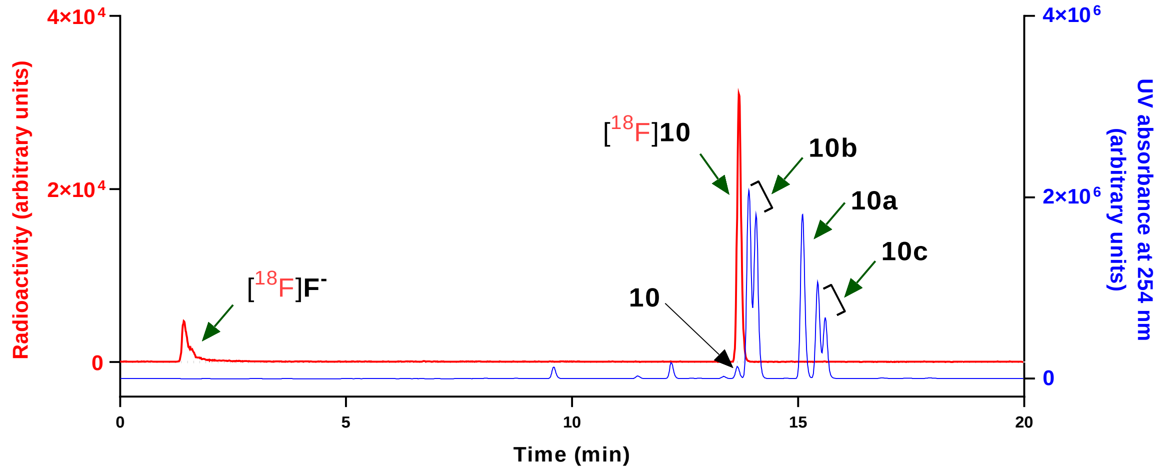

Supplementary Figure 36. Analytical HPLC chromatogram (condition A) for co-injection of reaction mixture producing [^{18}F]**10** from **10a** and reference compound **10c** (*E* and *Z* isomer mixture).

3.4.3. Proposed mechanism for the formation of α,α-difluoromethylalkane [^{18}F]10

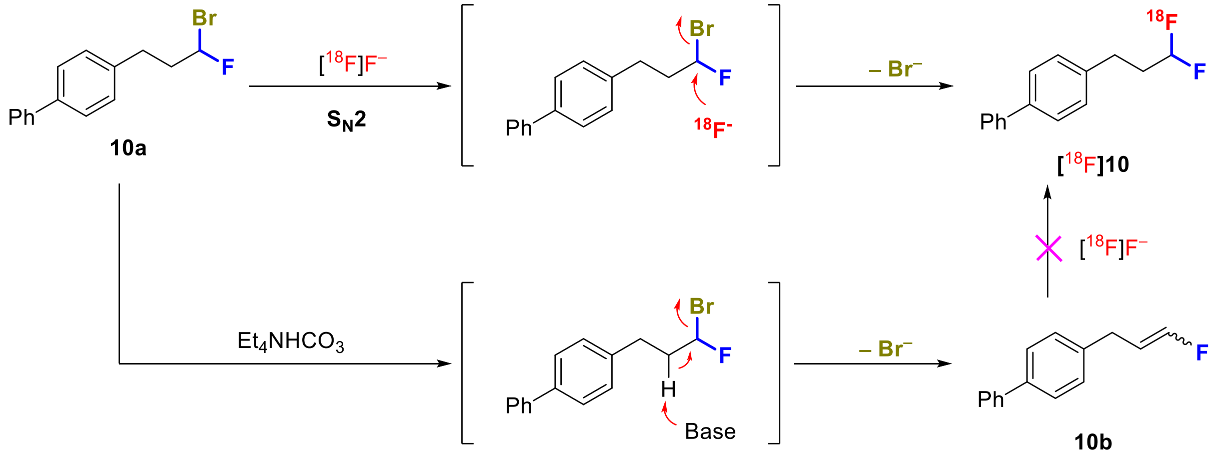

Supplementary Figure 37. Proposed mechanism for the radiofluorination α-fluoro,α-bromo alkane, exemplified with **10a**.

4. HPLC analyses of radiolabeled compounds [^{18}F]1–[^{18}F]47

HPLC analysis of [^{18}F]1

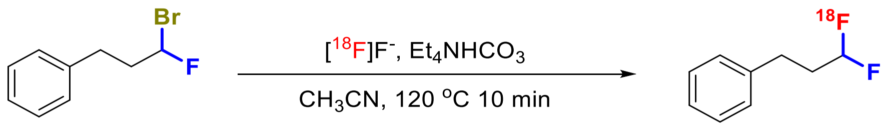

[^{18}F]**1**

Prepared following general procedure 8 and analyzed using HPLC condition A.

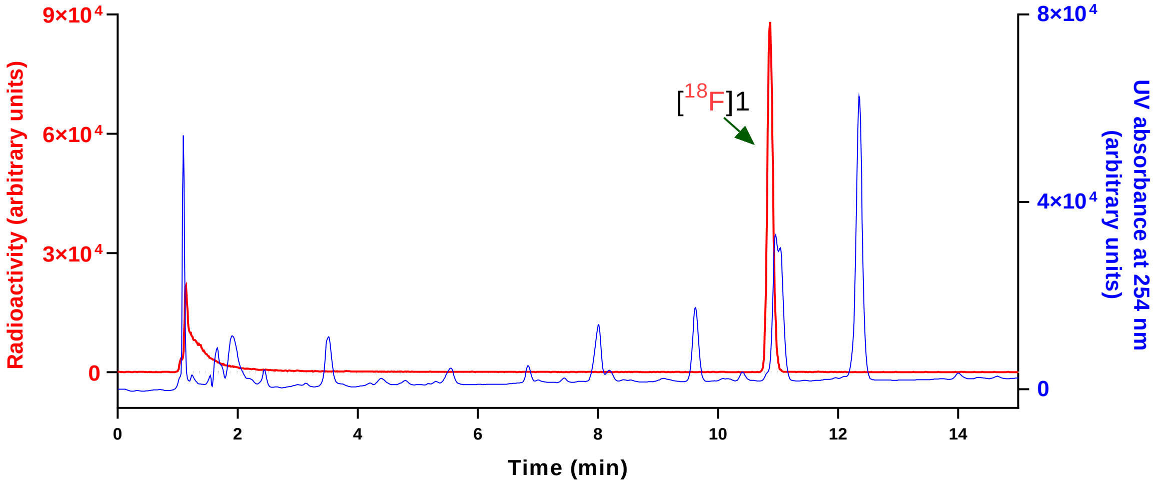

Supplementary Figure 38. Analytical HPLC (condition A)chromatogram for unpurified [^{18}F]**1**.

Supplementary Table 15. Radiosynthesis of [^{18}F]**1**.

Run	Retention time (min)	Yield (decay-corrected) (%)	Mean yield ± SD (%)	Isolated yield (%)
1	10.868	72	67 ± 5	70
2	10.868	63		
3	10.871	67		

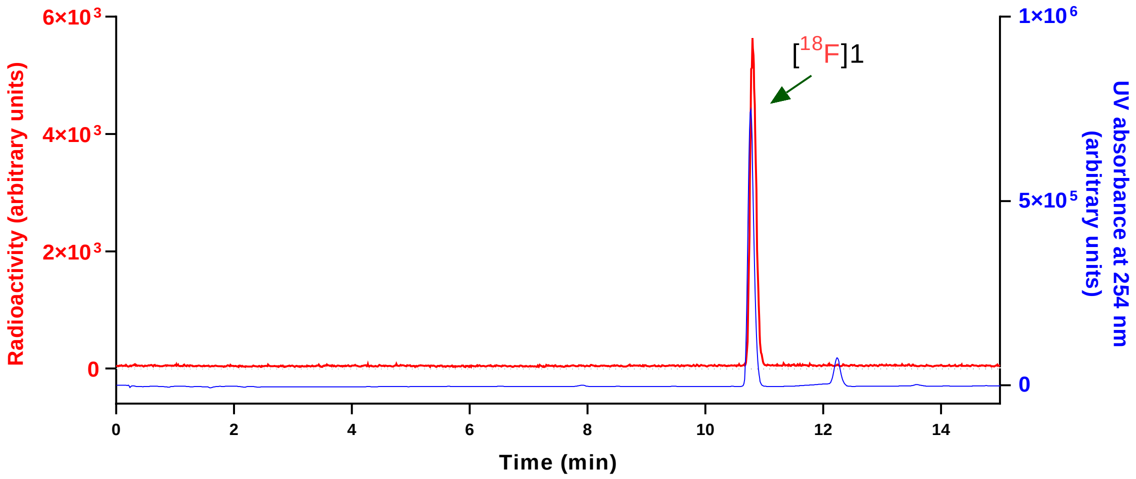

Supplementary Figure 39. Analytical HPLC (condition A) chromatogram for [^{18}F]**1** with co-injected **1**.

Separation of [^{18}F]**1** using HPLC condition D.

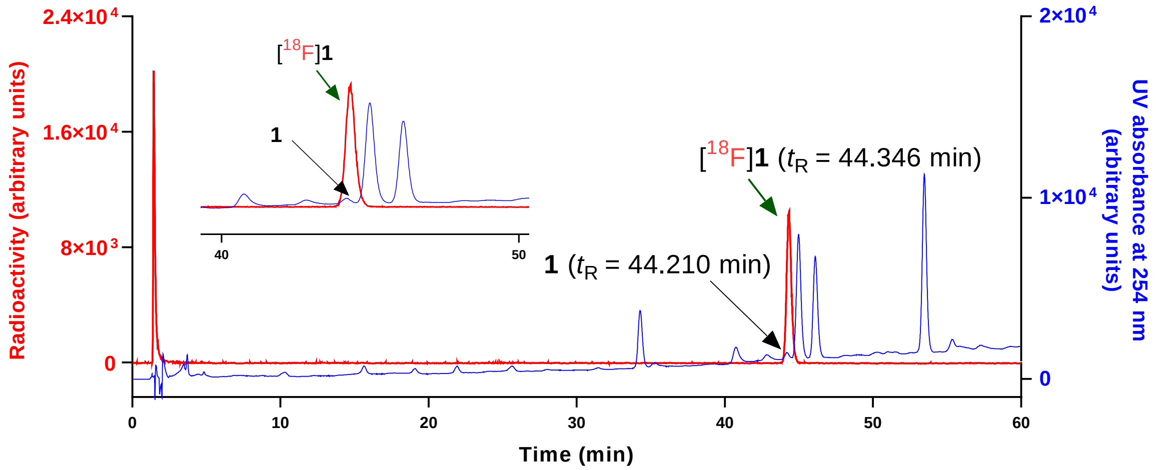

Supplementary Figure 40. Analytical HPLC (condition D) chromatogram for [^{18}F]**1** with expanded section for 40–50 min shown in the inset.

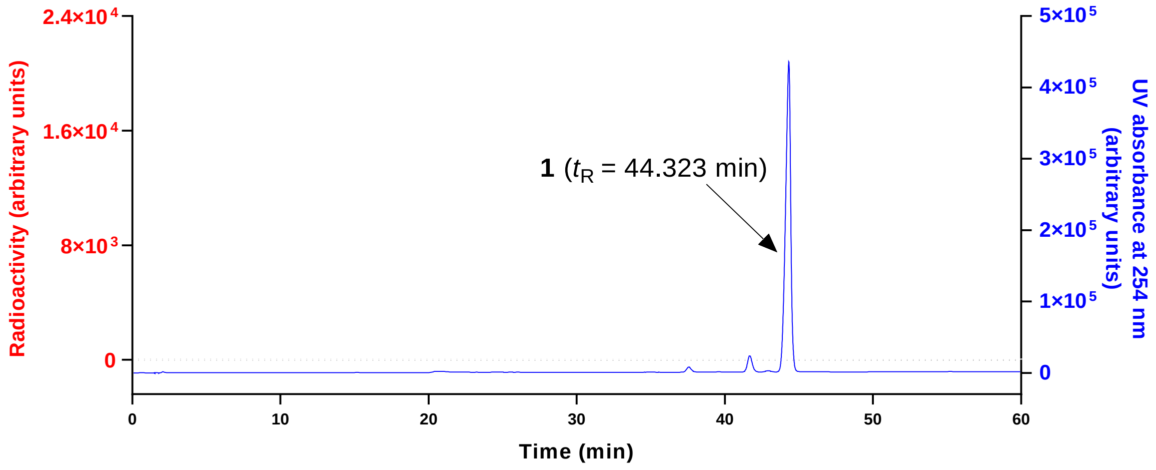

Supplementary Figure 41. Analytical HPLC (condition D) chromatogram for reference **1**.

HPLC analysis of [^{18}F]6

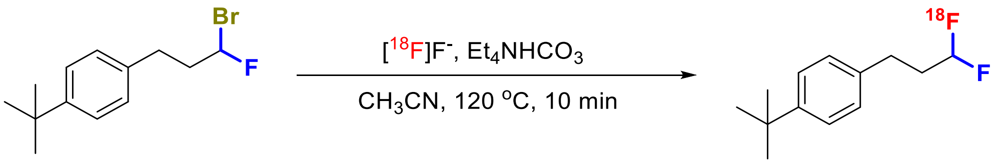

Prepared following general procedure 8 and analyzed using HPLC condition A.

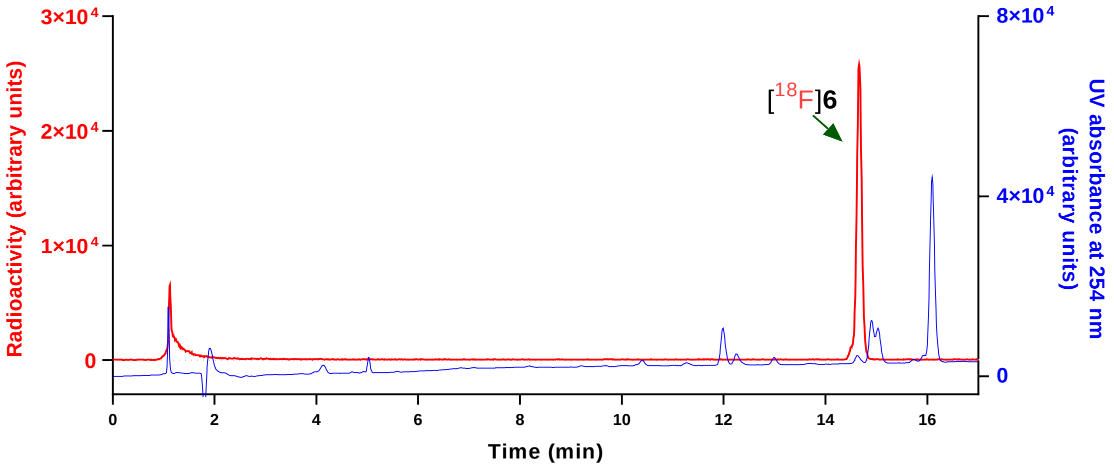

Supplementary Figure 42. Analytical HPLC chromatogram for unpurified [^{18}F]**6.**

Supplementary Table 16. Radiosynthesis of [^{18}F]**6**.

Run	Retention time (min)	Yield (decay-corrected) (%)	Mean yield ± SD (%)	Isolated yield (%)
1	14.660	73	74 ± 5	61
2	14.665	80		
3	14.656	70		

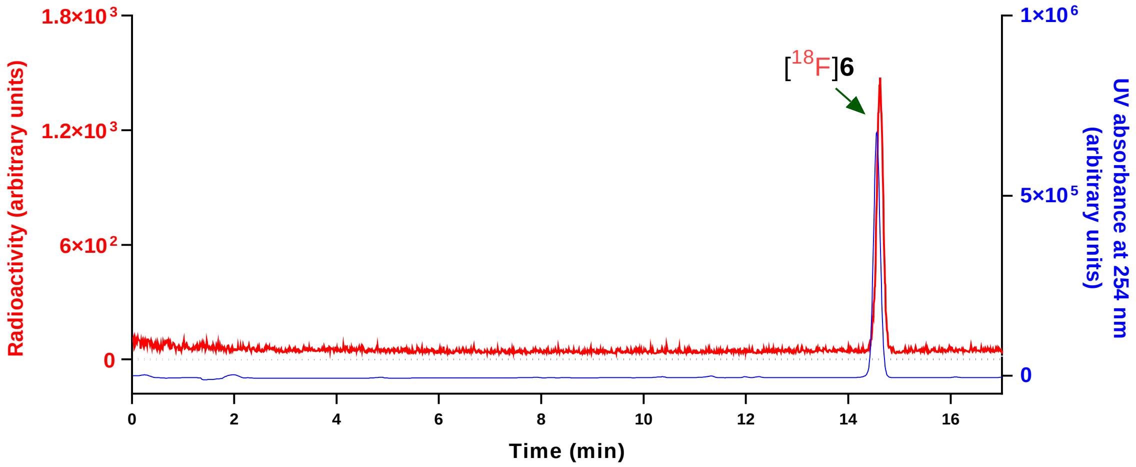

Supplementary Figure 43. Analytical HPLC chromatogram for [^{18}F]**6** with co–injected **6**.

HPLC analysis of [^{18}F]7

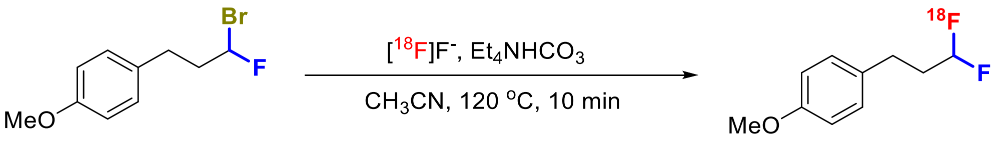

[^{18}F]7

Prepared following general procedure 8 and analyzed using HPLC condition A.

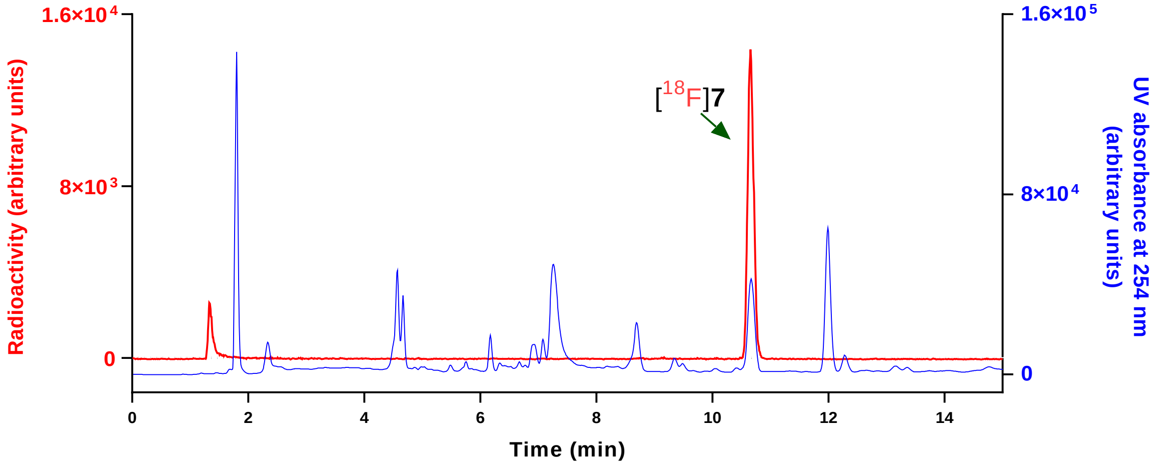

Supplementary Figure 44. Analytical HPLC chromatogram for reaction mixture of [^{18}F]**7**.

Supplementary Table 17. Radiosynthesis of [^{18}F]**7**.

Run	Retention time (min)	Yield (decay-corrected) (%)	Mean yield ± SD (%)	Isolated yield (%)
1	10.657	85	84 ± 2	83
2	10.539	84		
3	10.551	82		

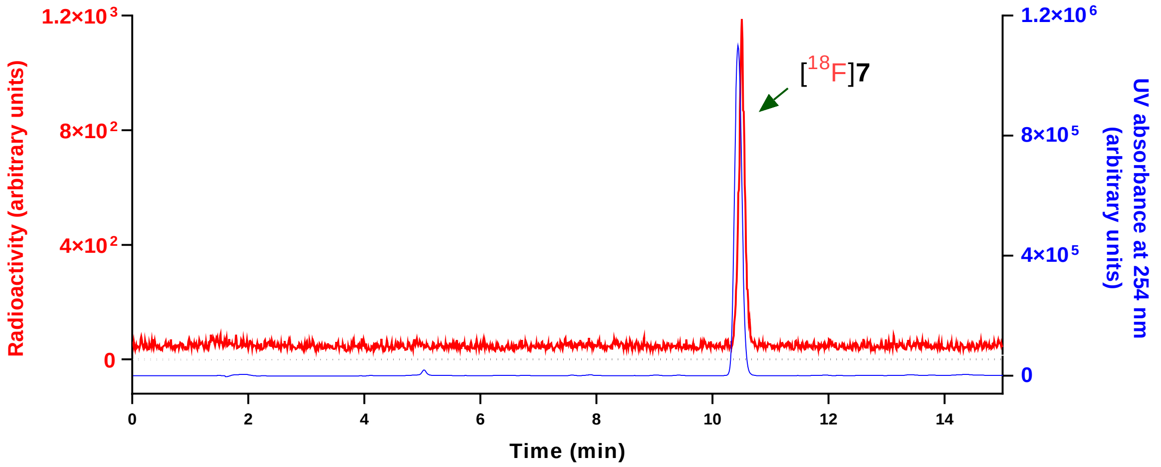

Supplementary Figure 45. Analytical HPLC chromatogram for [^{18}F]**7** with co–injected **7**.

Separation of [^{18}F]**7** using HPLC condition D.

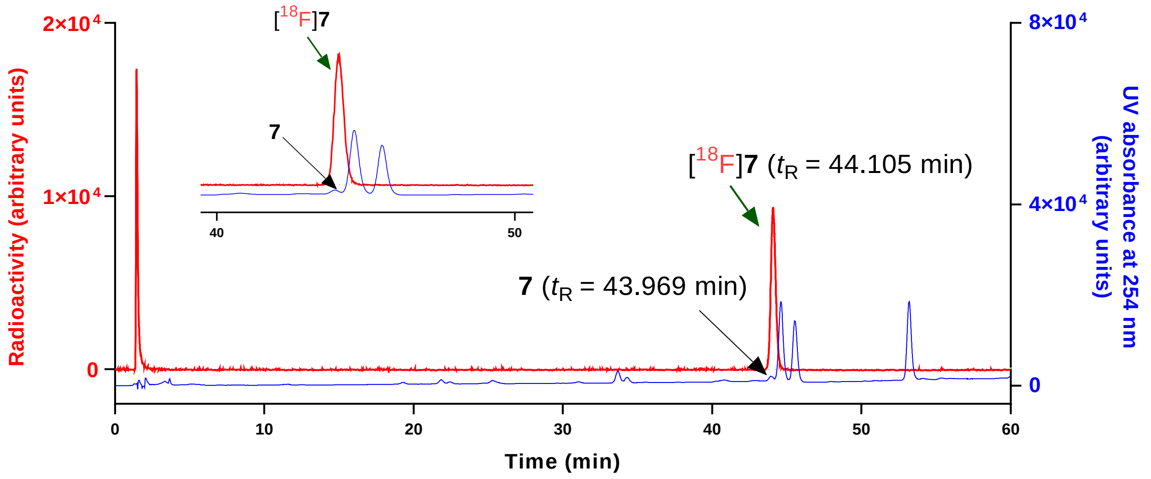

Supplementary Figure 46. Analytical HPLC (condition D) chromatogram for [^{18}F]**7** with expanded section of 40–50 min shown in the inset.

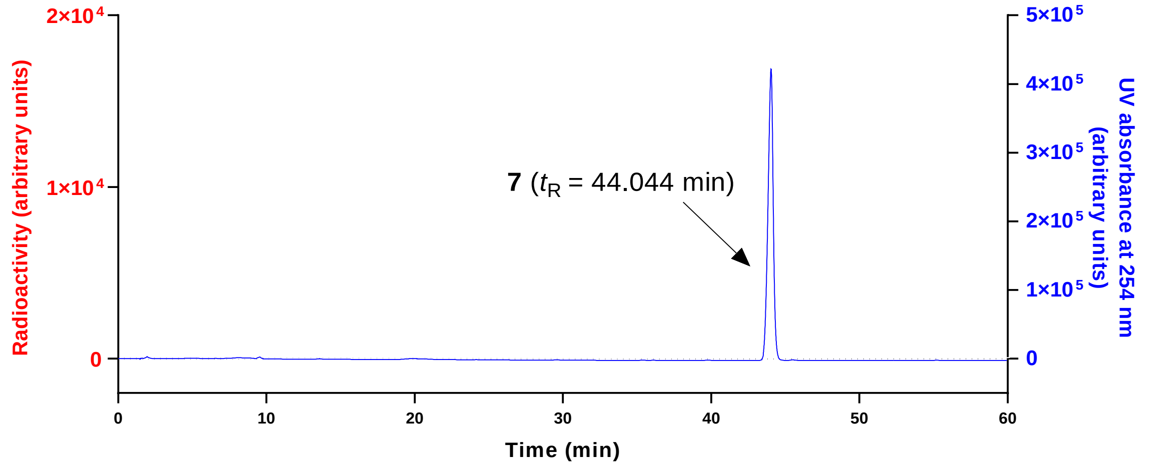

Supplementary Figure 47. Analytical HPLC (condition D) chromatogram for reference compound **7**.

HPLC analysis of [^{18}F]8

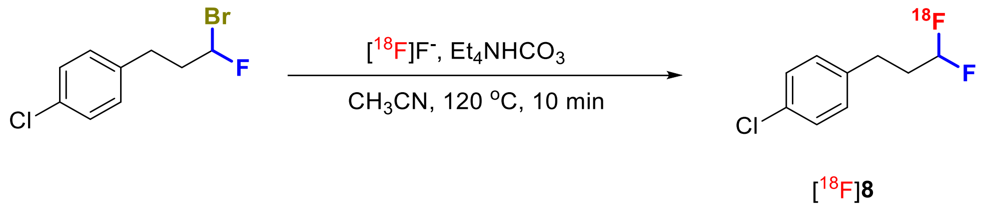

Prepared following general procedure 8 and analyzed using HPLC condition A.

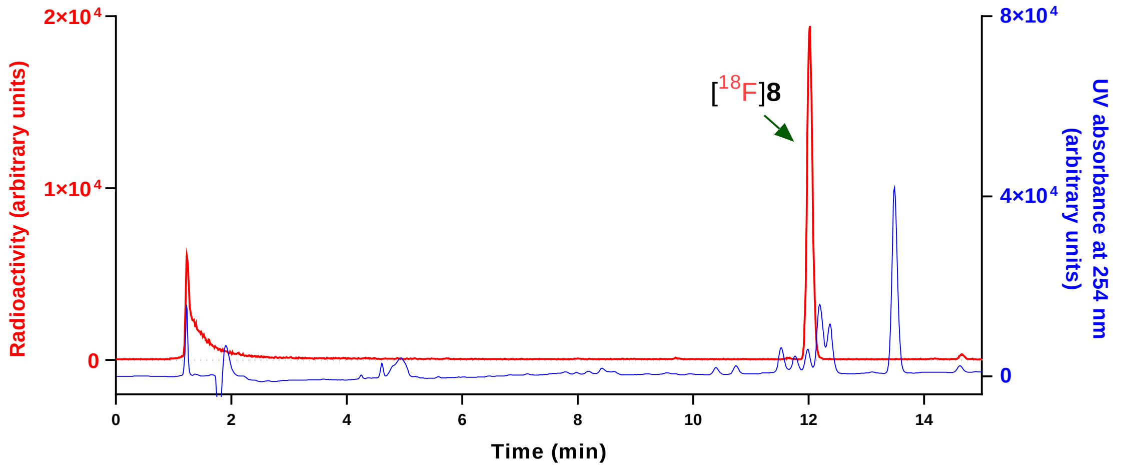

Supplementary Figure 48. Analytical HPLC chromatogram for reaction mixture of [^{18}F]**8**.

Supplementary Table 18. Radiosynthesis of [^{18}F]**8**.

Run	Retention time (min)	Yield (decay-corrected) (%)	Mean yield ± SD (%)	Isolated yield (%)
1	12.022	62	58 ± 8	43
2	12.018	63		
3	12.016	48		

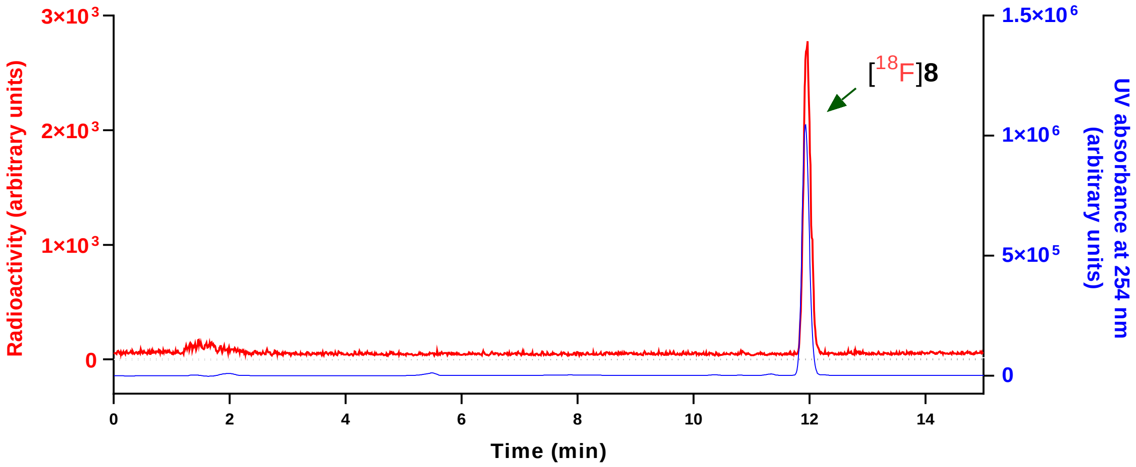

Supplementary Figure 49. Analytical HPLC chromatogram for [^{18}F]**8** with co–injected **8**.

HPLC analysis of [^{18}F]9

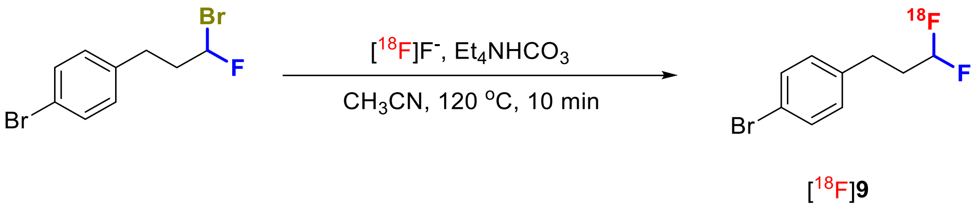

Prepared following general procedure 8 and analyzed using HPLC condition A.

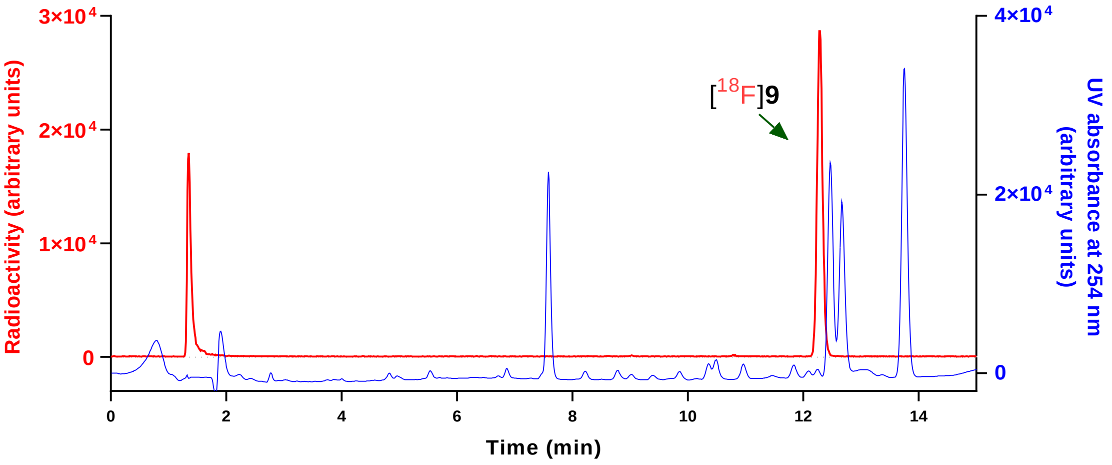

Supplementary Figure 50. Analytical HPLC chromatogram for reaction mixture of [^{18}F]**9**.

**Supplementary Table 19.** Radiosynthesis of [^{18}F]**9**.				
Run	Retention time (min)	Yield (decay-corrected) (%)	Mean yield ± SD (%)	Isolated yield (%)
1	12.382	70	72 ± 6	64
2	12.374	78		
3	12.288	67		

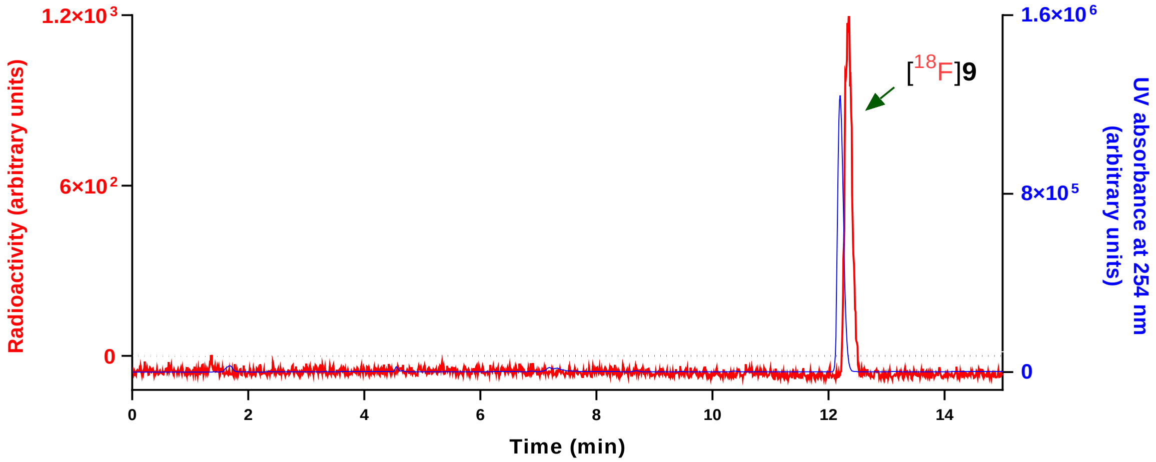

Supplementary Figure 51. Analytical HPLC chromatogram for [^{18}F]**9** with co–injected **9**.

HPLC analysis of [^{18}F]10

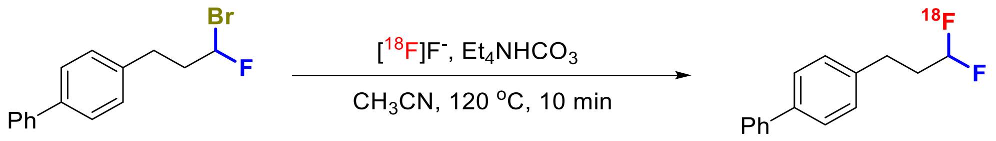

[^{18}F]**10**

Prepared following general procedure 8 and analyzed using HPLC condition A.

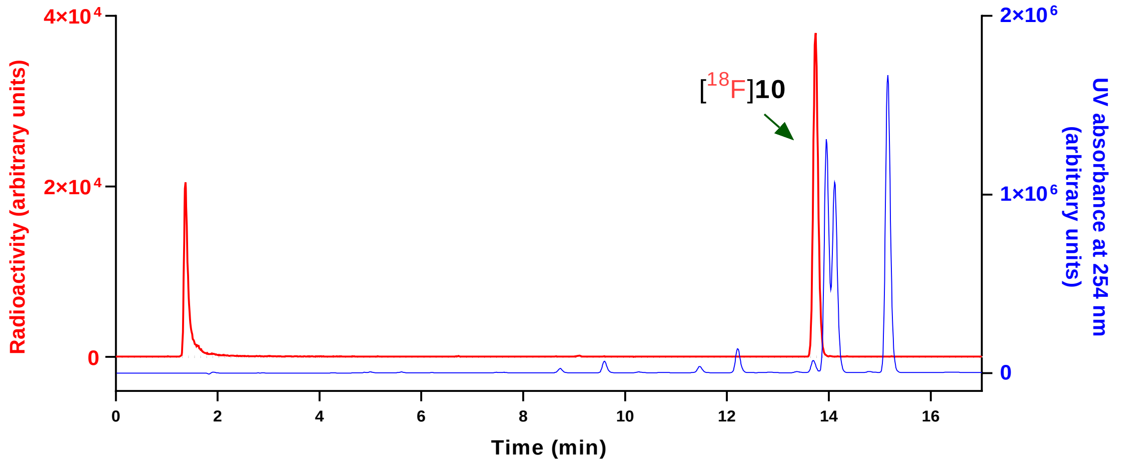

Supplementary Figure 52. Analytical HPLC chromatogram for reaction mixture of [^{18}F]**10**.

Supplementary Table 20. Radiosynthesis of [^{18}F]**10.**

Run	Retention time (min)	Yield (decay-corrected) (%)	Mean yield ± SD (%)	Isolated yield (%)
1	13.739	65	64 ± 3	59
2	13.741	61		
3	13.739	67		

HPLC analysis of [^{18}F]11

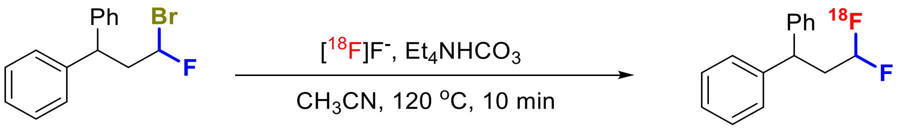

Prepared following general procedure 8 and analyzed using HPLC condition A.

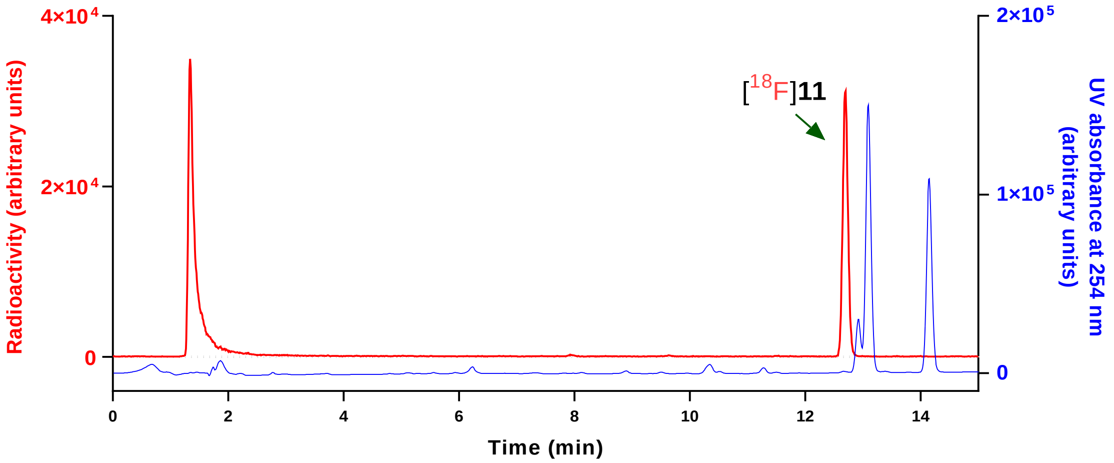

Supplementary Figure 53. Analytical HPLC chromatogram for reaction mixture of [^{18}F]**11**.

Supplementary Table 21. Radiosynthesis of [^{18}F]**11**.

Run	Retention time (min)	Yield (decay-corrected) (%)	Mean yield ± SD (%)	Isolated yield (%)
1	12.700	38	37 ± 2	36
2	12.969	35		
3	12.697	39		

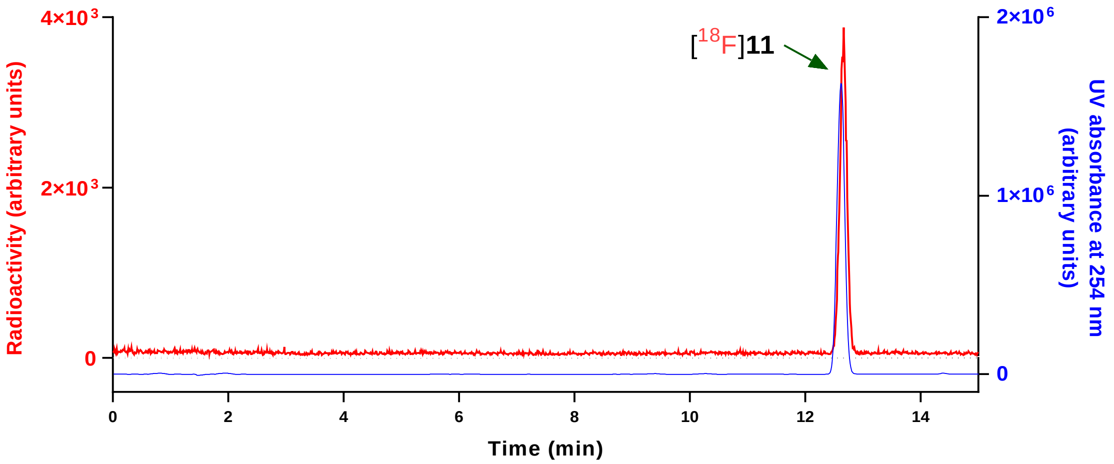

Supplementary Figure 54. Analytical HPLC chromatogram for [^{18}F]**11** with co–injected **11**.

HPLC analysis of [^{18}F]12

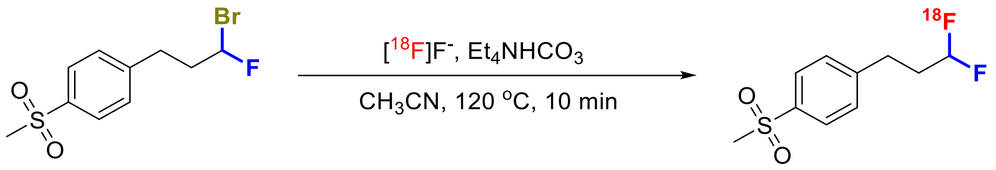

Prepared following general procedure 8 and analyzed using HPLC condition A.

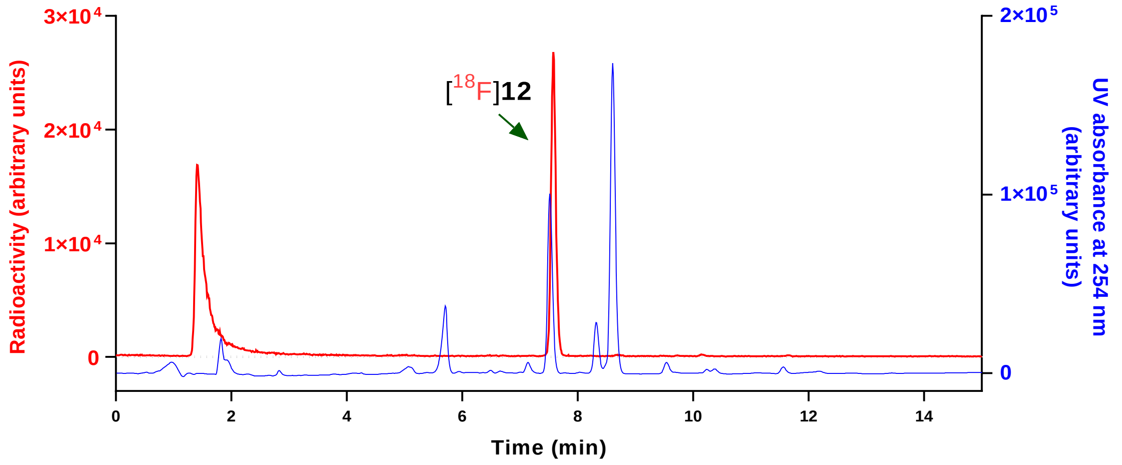

Supplementary Figure 55. Analytical HPLC chromatogram for reaction mixture of [^{18}F]**12**.

Supplementary Table 22. Radiosynthesis of [^{18}F]**12**.

Run	Retention time (min)	Yield (decay-corrected) (%)	Mean yield ± SD (%)	Isolated yield (%)
1	7.582	39	37 ± 4	27
2	7.563	33		
3	7.572	39		

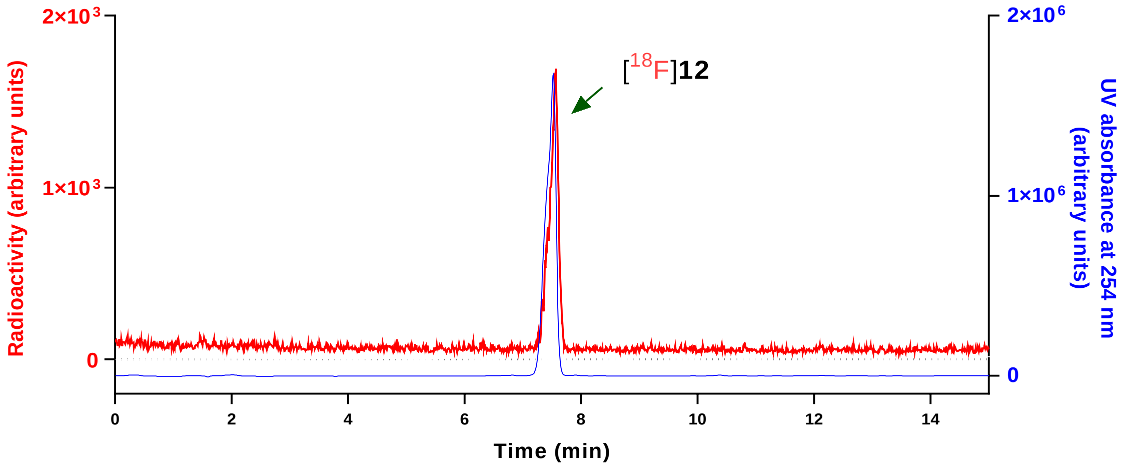

Supplementary Figure 56. Analytical HPLC chromatogram for [^{18}F]**12** with co–injected **12**.

HPLC analysis of [^{18}F]13

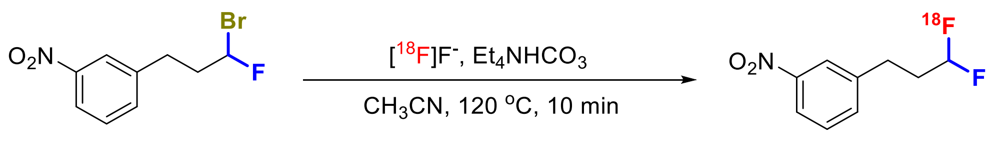

Prepared following general procedure 8 and analyzed using HPLC condition A.

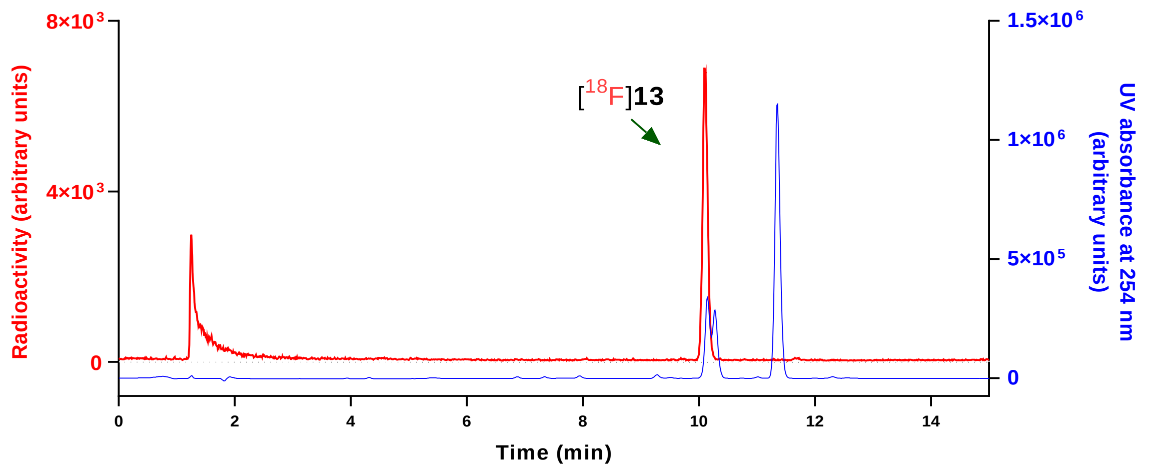

Supplementary Figure 57. Analytical HPLC chromatogram for reaction mixture of [^{18}F]**13**.

Supplementary Table 23. Radiosynthesis of [^{18}F]**13**.

Run	Retention time (min)	Yield (decay-corrected) (%)	Mean yield ± SD (%)	Isolated yield (%)
1	10.102	56	50 ± 6	44
2	10.062	48		
3	10.208	45		

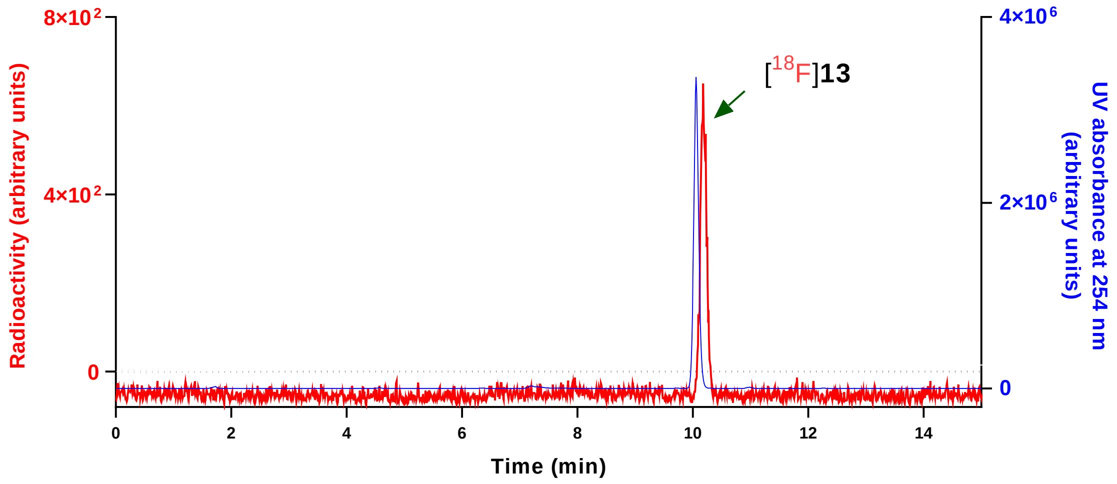

Supplementary Figure 58. Analytical HPLC chromatogram for [^{18}F]**13** with co–injected **13**.

Separation of [^{18}F]**13** using HPLC condition D.

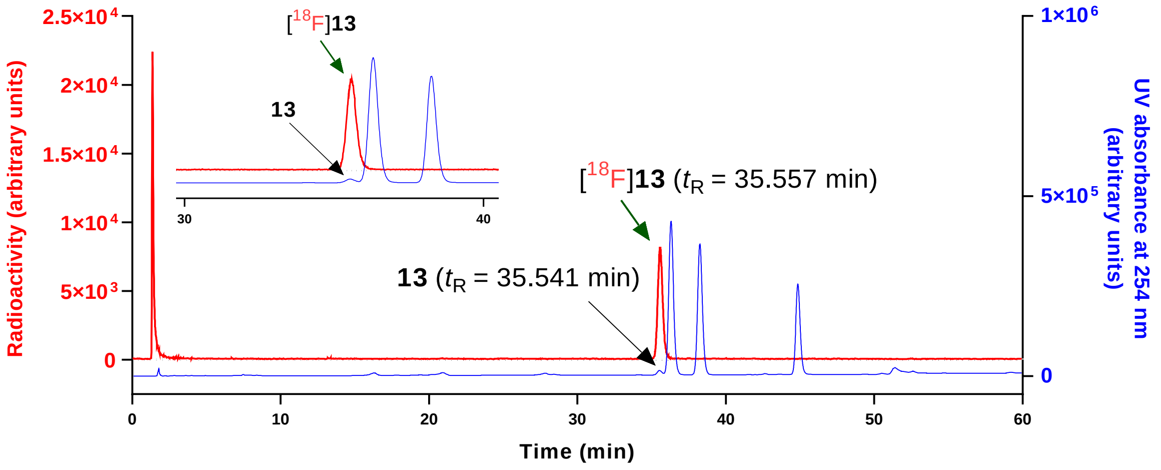

Supplementary Figure 59. Analytical HPLC (condition D) chromatogram for [^{18}F]**13** with expanded section of 30–40 min shown in the inset.

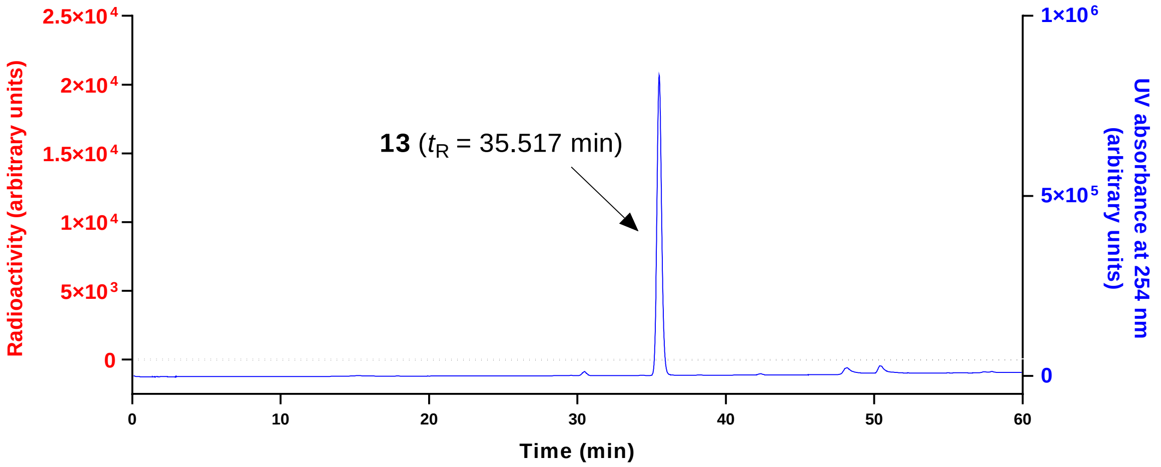

Supplementary Figure 60. Analytical HPLC (condition D) chromatogram for reference compound **13**.

HPLC analysis of [^{18}F]14

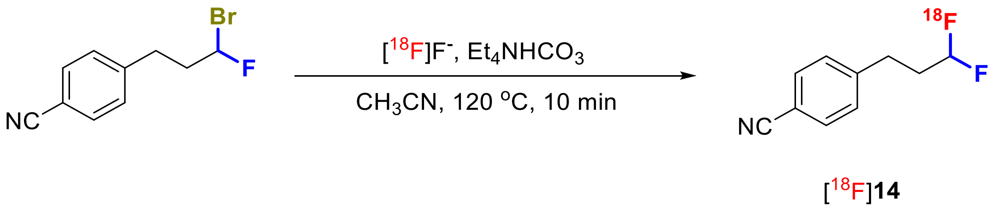

Prepared following general procedure 8 and analyzed using HPLC condition A.

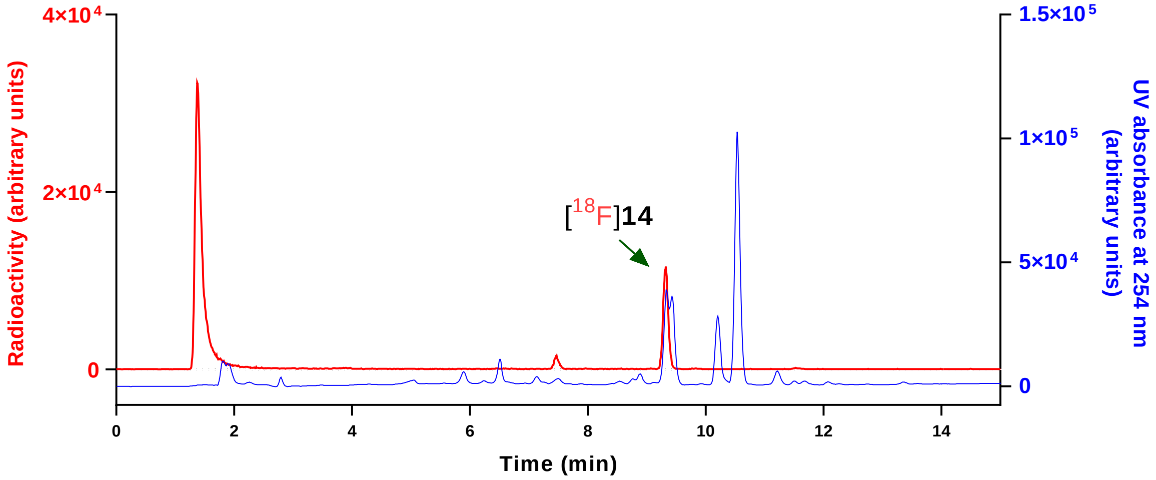

Supplementary Figure 61. Analytical HPLC chromatogram for reaction mixture of [^{18}F]**14**.

**Supplementary Table 24.** Radiosynthesis of [^{18}F]**14**.				
Run	Retention time (min)	Yield (decay-corrected) (%)	Mean yield ± SD (%)	Isolated yield (%)
1	9.322	16	17 ± 2	17
2	9.325	19		
3	9.319	16		

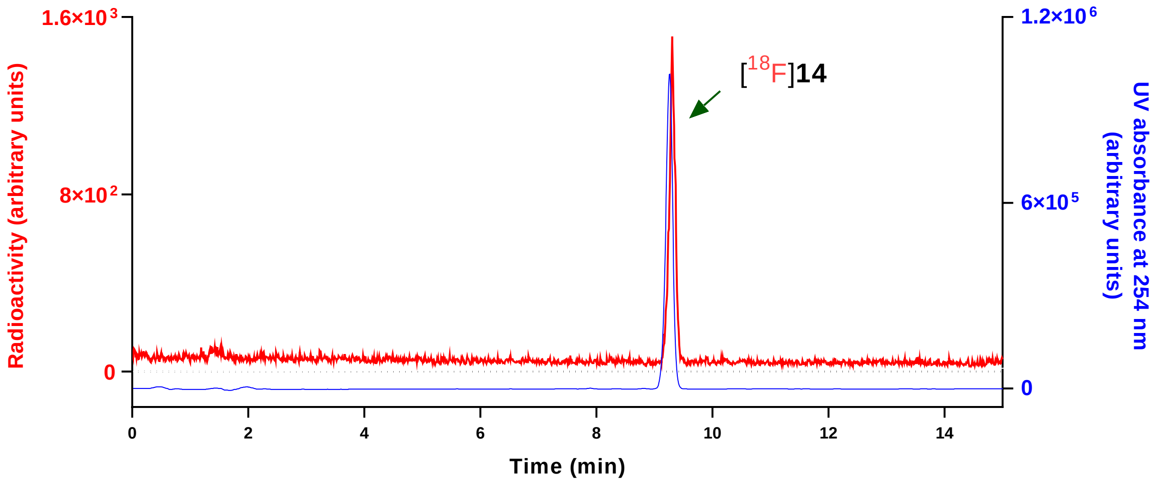

Supplementary Figure 62. Analytical HPLC chromatogram for [^{18}F]**14** with co–injected **14**.

Separation of [^{18}F]**14** using HPLC condition D.

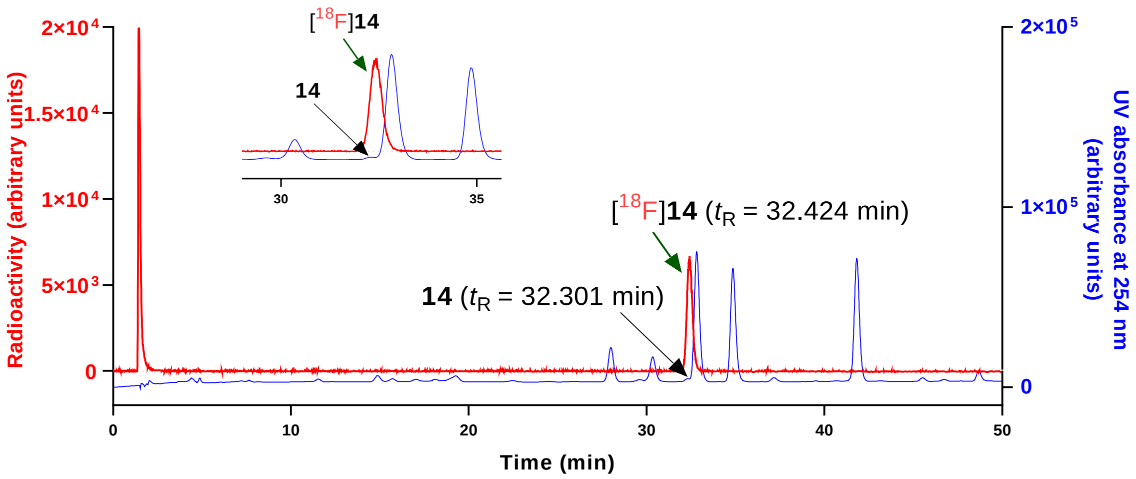

Supplementary Figure 63. Analytical HPLC (condition D) chromatogram for [^{18}F]**14** with expanded section of 30–35 min shown in the inset.

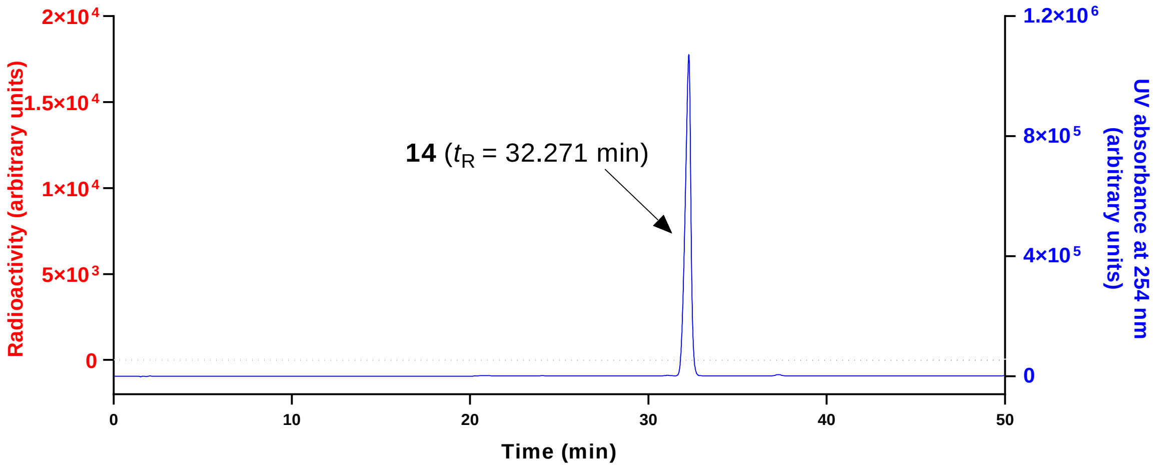

Supplementary Figure 64. Analytical HPLC (condition D) chromatogram for reference compound **14**.

HPLC analysis of [^{18}F]15

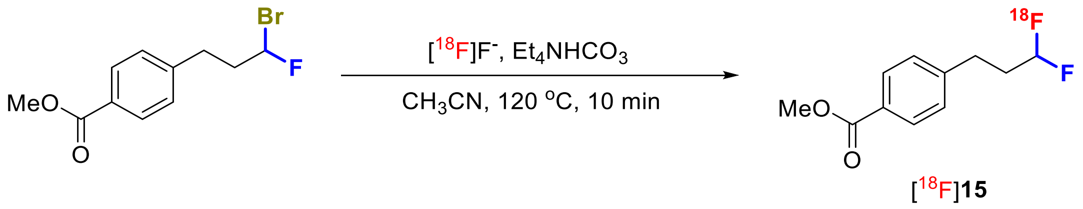

Prepared following general procedure 8 and analyzed using HPLC condition A.

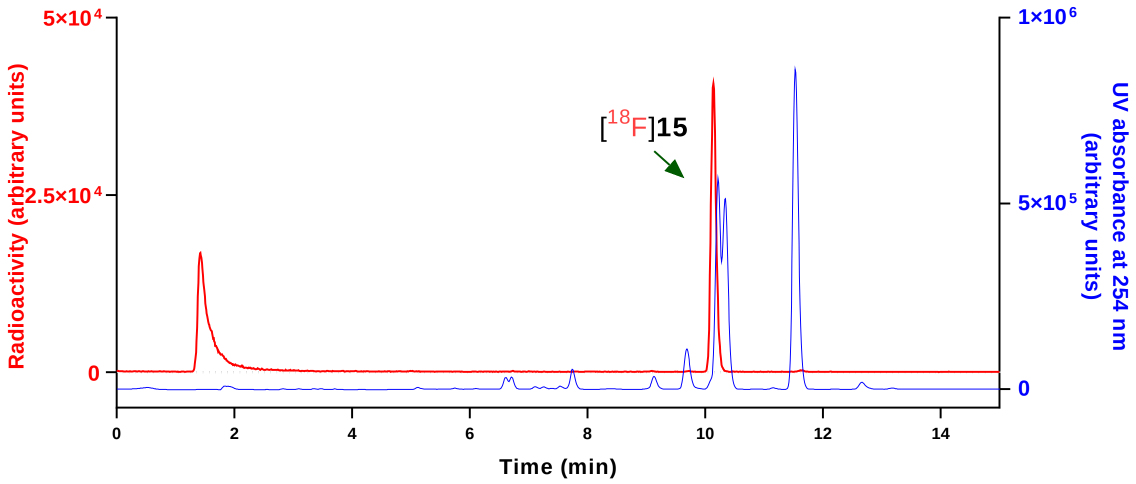

Supplementary Figure 65. Analytical HPLC chromatogram for reaction mixture of [^{18}F]**15**.

Supplementary Table 25. Radiosynthesis of [^{18}F]**15**.

Run	Retention time (min)	Yield (decay-corrected) (%)	Mean yield ± SD (%)	Isolated yield (%)
1	10.132	56	54 ± 3	49
2	10.141	51		
3	10.145	56		

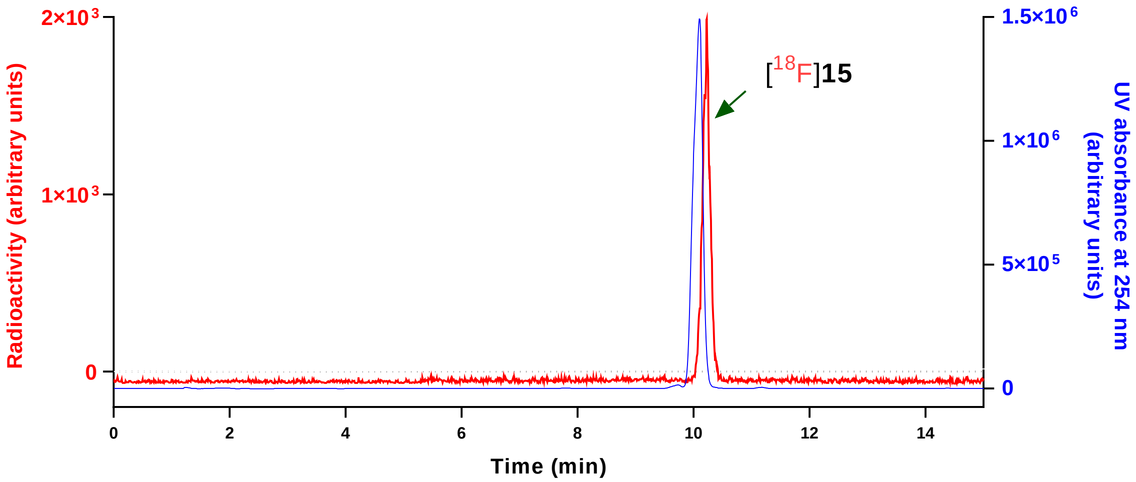

Supplementary Figure 66. Analytical HPLC chromatogram for [^{18}F]**15** with co–injected **15**.

HPLC analysis of [^{18}F]16

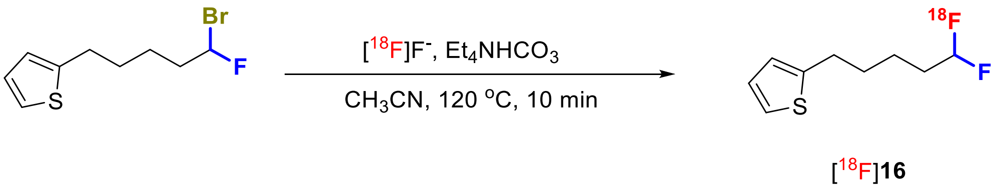

Prepared following general procedure 8 and analyzed using HPLC condition A.

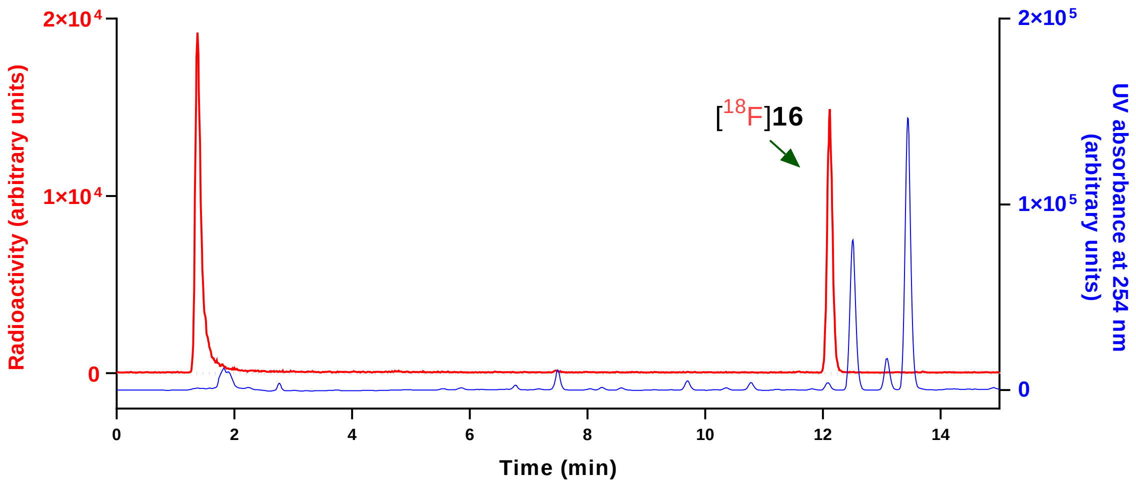

Supplementary Figure 67. Analytical HPLC chromatogram for reaction mixture of [^{18}F]**16**.

**Supplementary Table 26.** Radiosynthesis of [^{18}F]**16**.				
Run	Retention time (min)	Yield (decay-corrected) (%)	Mean yield ± SD (%)	Isolated yield (%)
1	12.313	36	36 ± 2	28
2	12.130	34		
3	12.117	38		

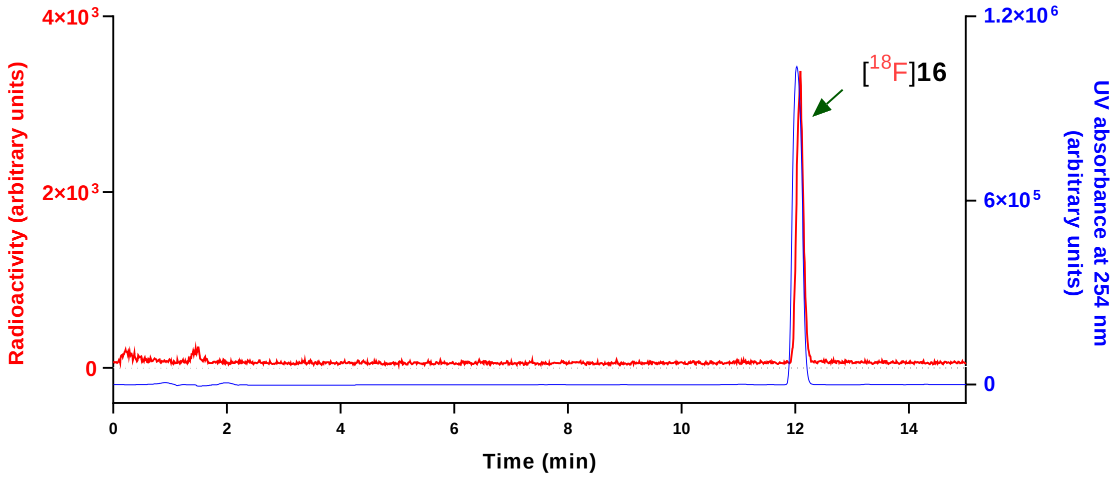

Supplementary Figure 68. Analytical HPLC chromatogram for [^{18}F]**16** with co–injected **16**.

HPLC analysis of [^{18}F]17

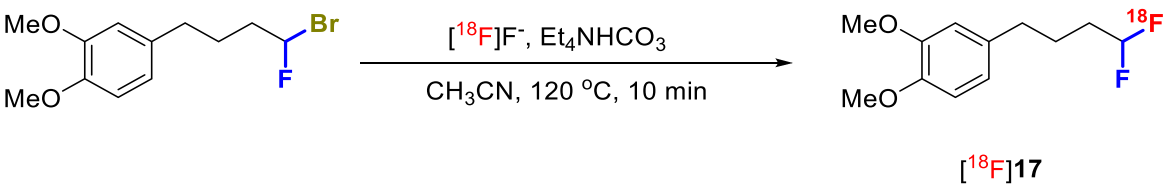

Prepared following general procedure 8 and analyzed using HPLC condition A.

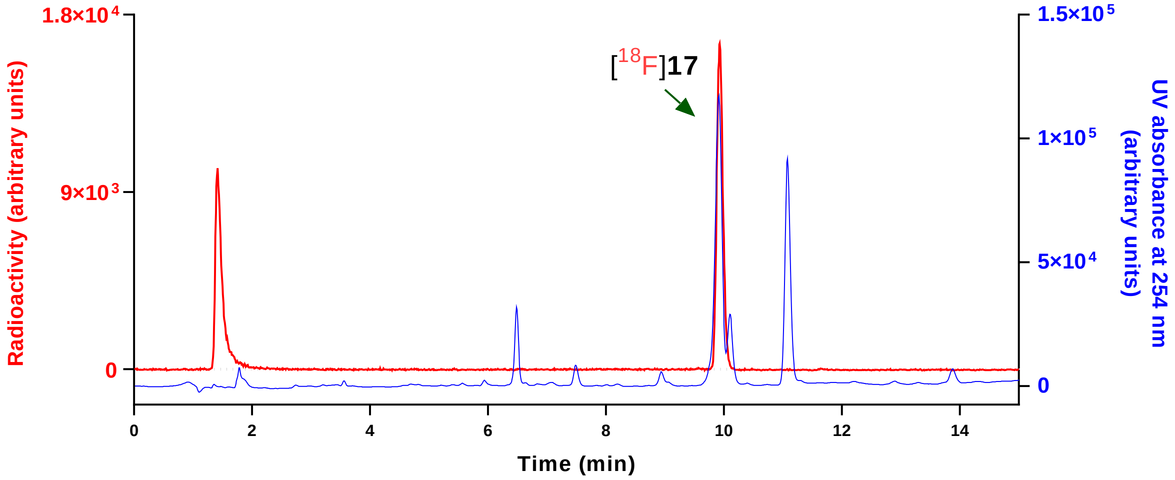

Supplementary Figure 69. Analytical HPLC chromatogram for reaction mixture of [^{18}F]**17**.

Supplementary Table 27. Radiosynthesis of [^{18}F]**17**.

Run	Retention time (min)	Yield (decay-corrected) (%)	Mean yield ± SD (%)	Isolated yield (%)
1	9.928	54	55 ± 3	52
2	9.930	58		
3	9.881	53		

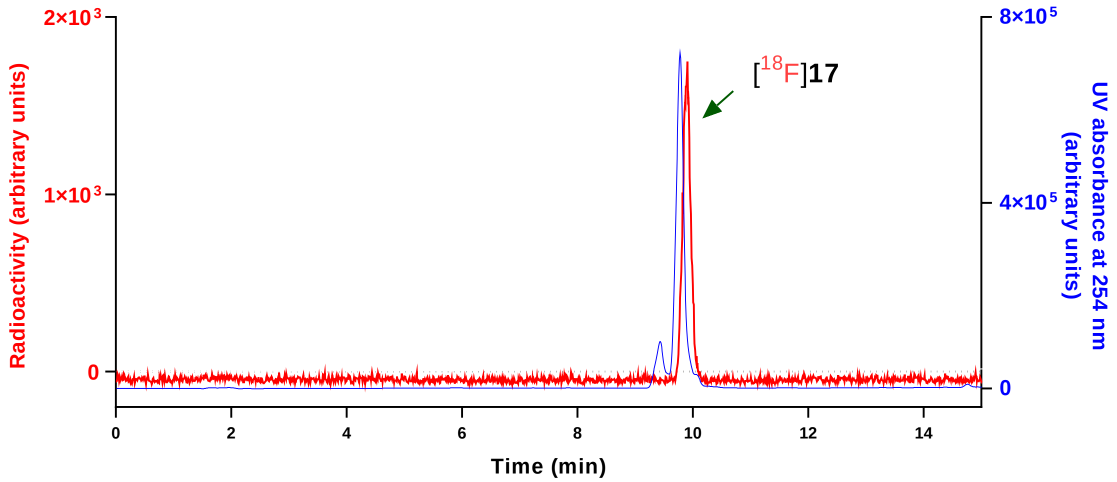

Supplementary Figure 70. Analytical HPLC chromatogram for [^{18}F]**17** with co–injected **17**.

HPLC analysis of [^{18}F]18

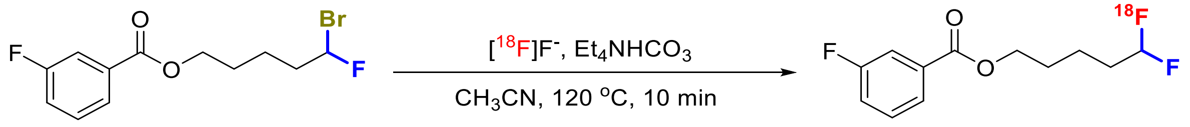

[^{18}F]**18**

Prepared following general procedure 8 and analyzed using HPLC condition A.

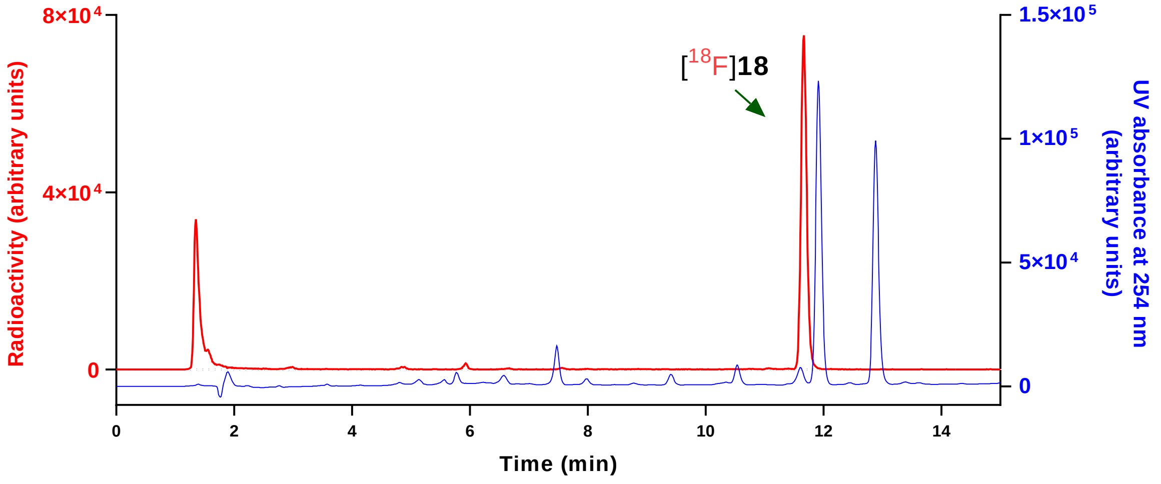

Supplementary Figure 71. Analytical HPLC chromatogram for reaction mixture of [^{18}F]**18**.

Supplementary Table 28. Radiosynthesis of [^{18}F]**18**.

Run	Retention time (min)	Yield (decay-corrected) (%)	Mean yield ± SD (%)	Isolated yield (%)
1	11.666	66	67 ± 2	61
2	11.665	67		
3	11.674	69		

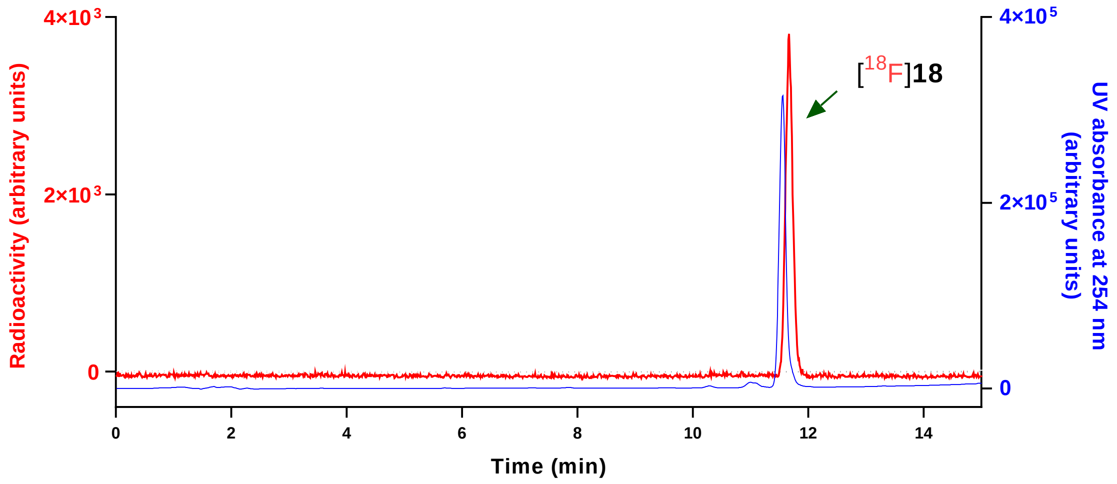

Supplementary Figure 72. Analytical HPLC chromatogram for [^{18}F]**18** with co–injected **18**.

HPLC analysis of [^{18}F]19

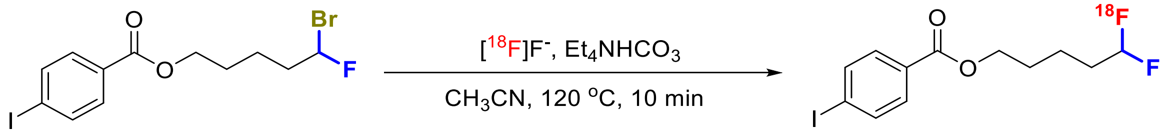

[^{18}F]**19**

Prepared following general procedure 8 and analyzed using HPLC condition A.

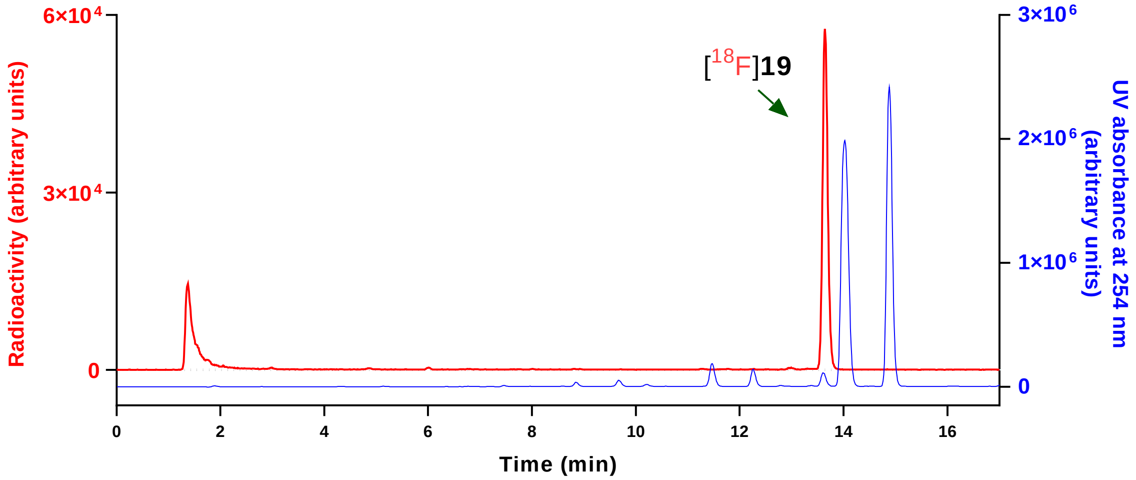

Supplementary Figure 73. Analytical HPLC chromatogram for reaction mixture of [^{18}F]**19**.

Supplementary Table 29. Radiosynthesis of [^{18}F]**19**.

Run	Retention time (min)	Yield (decay-corrected) (%)	Mean yield ± SD (%)	Isolated yield (%)
1	13.641	70	69 ± 2	63
2	13.629	67		
3	13.633	69		

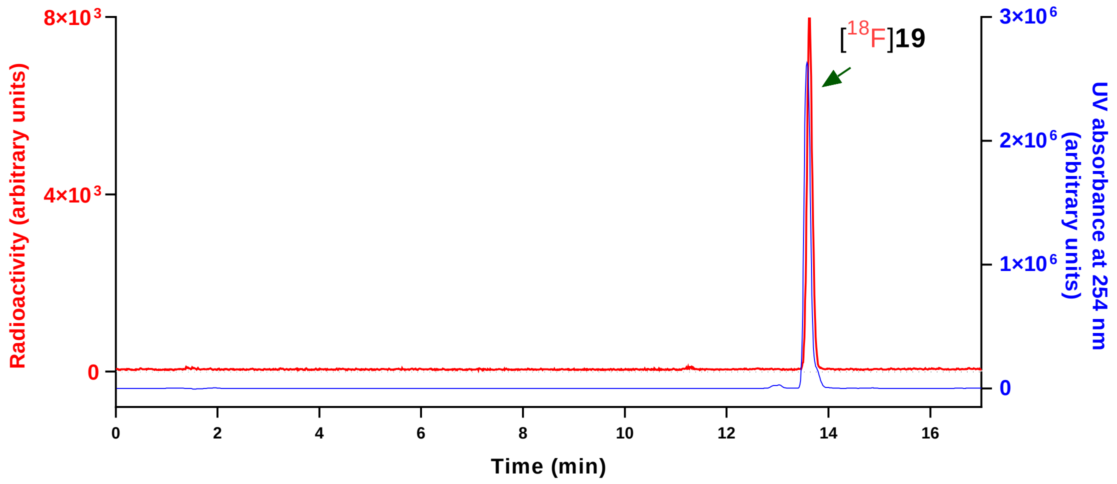

Supplementary Figure 74. Analytical HPLC chromatogram for [^{18}F]**19** with co–injected **19**.

HPLC analysis of [^{18}F]20

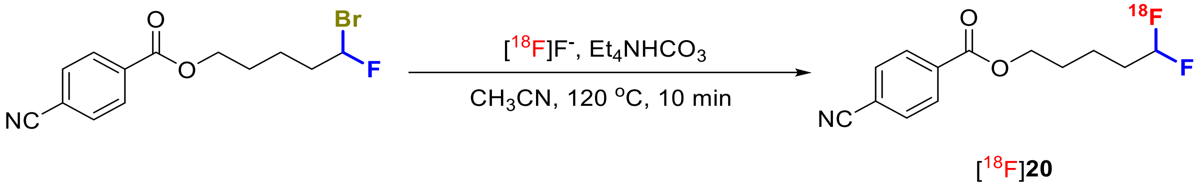

Prepared following general procedure 8 and analyzed using HPLC condition A.

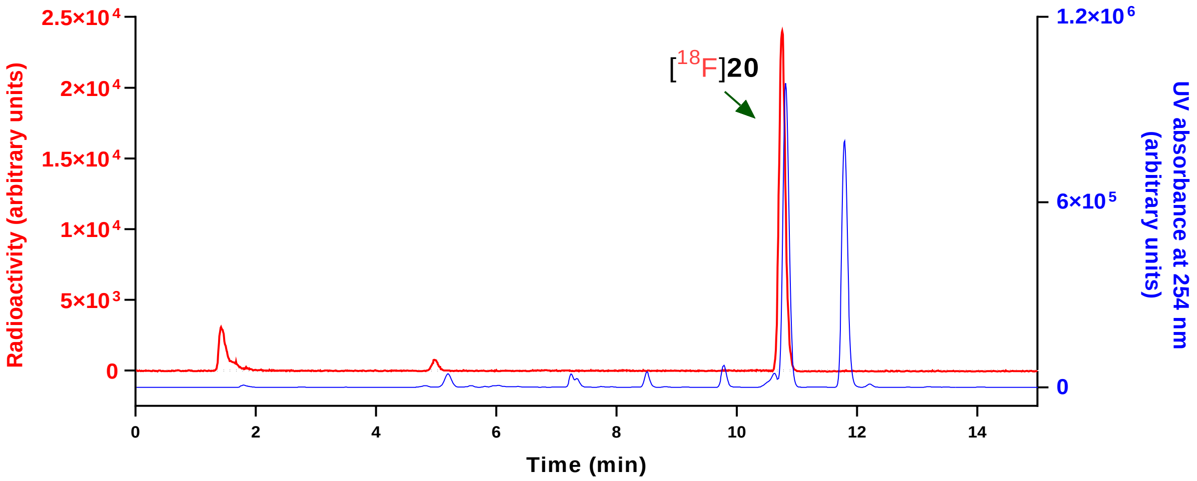

Supplementary Figure 75. Analytical HPLC chromatogram for reaction mixture of [^{18}F]**20**.

Supplementary Table 30. Radiosynthesis of [^{18}F]**20**.

Run	Retention time (min)	Yield (decay-corrected) (%)	Mean yield ± SD (%)	Isolated yield (%)
1	10.756	83	83 ± 1	75
2	10.756	82		
3	10.749	83		

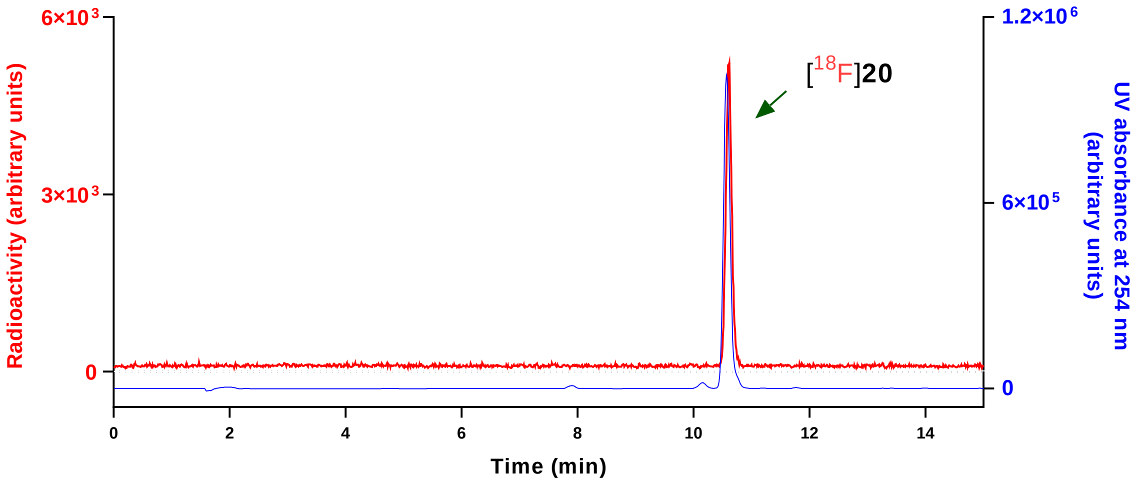

Supplementary Figure 76. Analytical HPLC chromatogram for [^{18}F]**20** with co–injected **20**.

HPLC analysis of [^{18}F]21

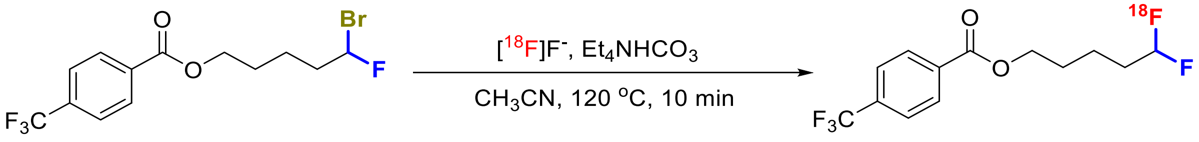

[^{18}F]**21**

Prepared following general procedure 8 and analyzed using HPLC condition A.

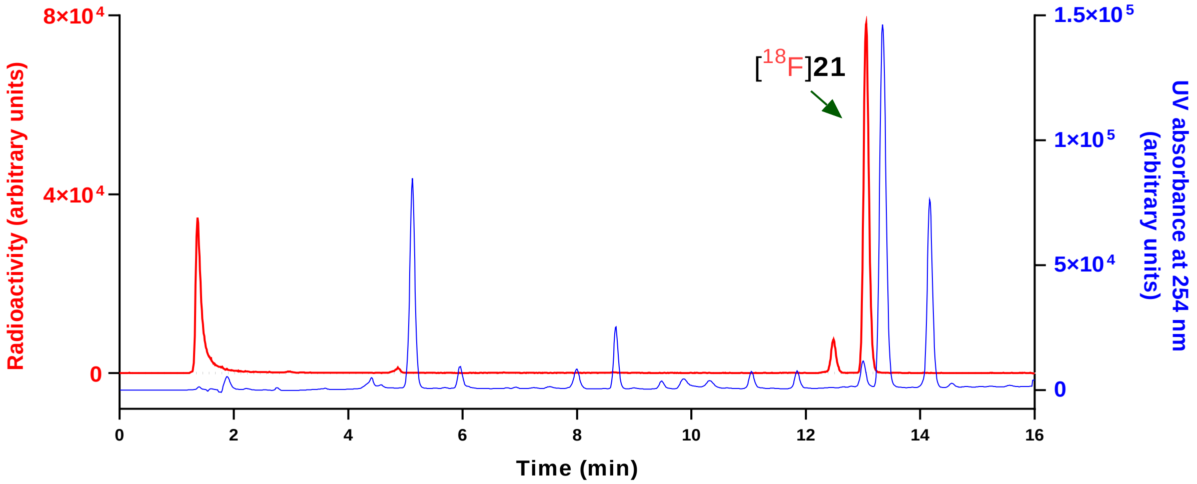

Supplementary Figure 77. Analytical HPLC chromatogram for reaction mixture of [^{18}F]**21**.

Supplementary Table 31. Radiosynthesis of [^{18}F]**21**.

Run	Retention time (min)	Yield (decay-corrected) (%)	Mean yield ± SD (%)	Isolated yield (%)
1	13.055	60	61 ± 1	57
2	13.061	62		
3	13.064	61		

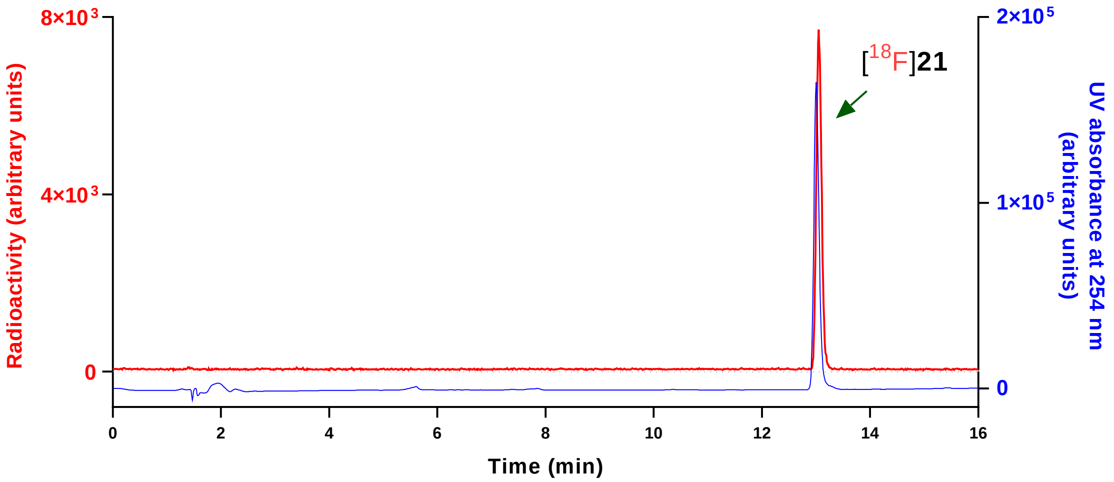

Supplementary Figure 78. Analytical HPLC chromatogram for [^{18}F]**21** with co–injected **21**.

HPLC analysis of [^{18}F]22

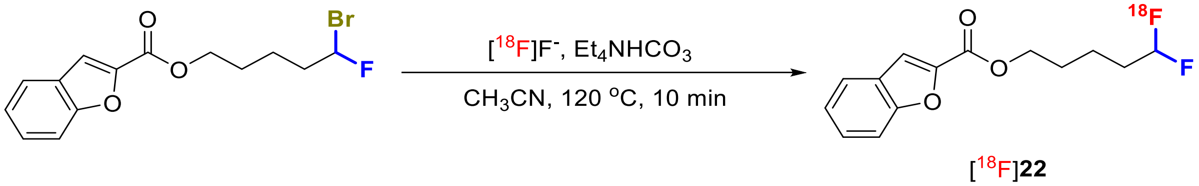

Prepared following general procedure 8 and analyzed using HPLC condition A.

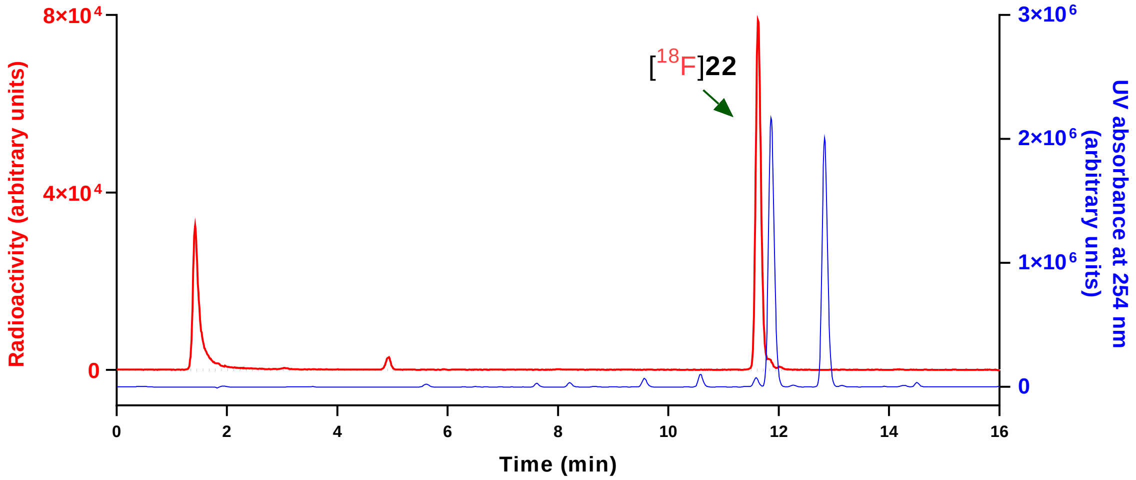

Supplementary Figure 79. Analytical HPLC chromatogram for reaction mixture of [^{18}F]**22**.

**Supplementary Table 32.** Radiosynthesis of [^{18}F]**22**.				
Run	Retention time (min)	Yield (decay-corrected) (%)	Mean yield ± SD (%)	Isolated yield (%)
1	11.636	58	60 ± 2	58
2	11.628	62		
3	11.625	60		

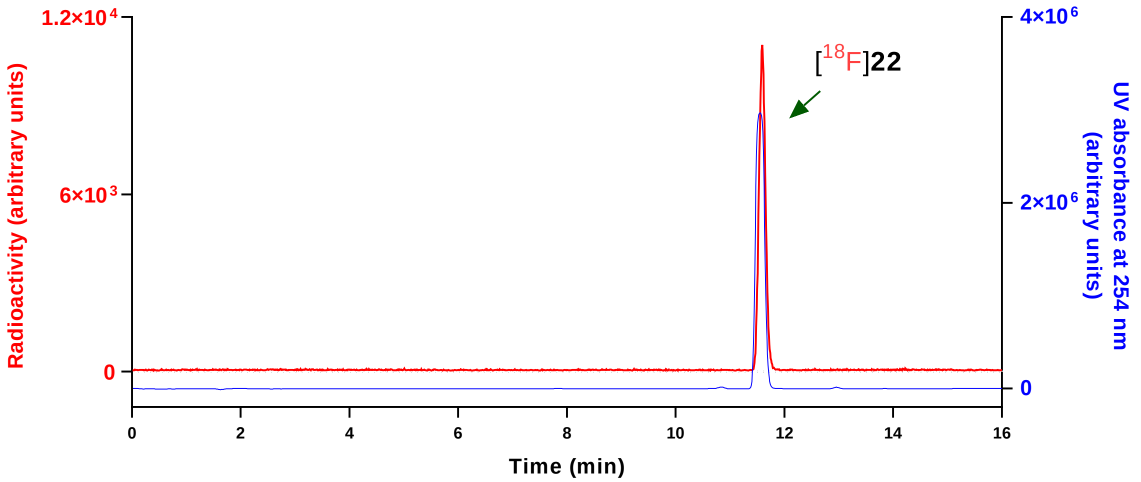

Supplementary Figure 80. Analytical HPLC chromatogram for [^{18}F]**22** with co–injected **22**.

HPLC analysis of [^{18}F]23

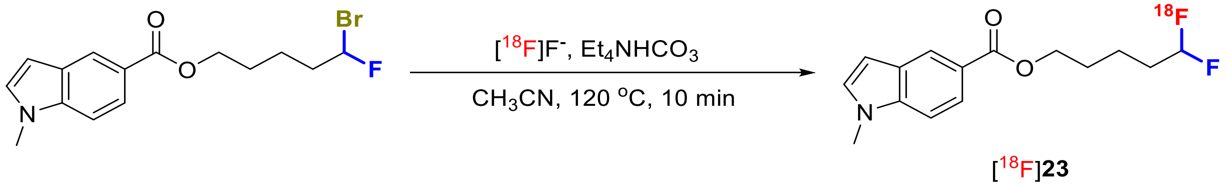

Prepared following general procedure 8 and analyzed using HPLC condition A.

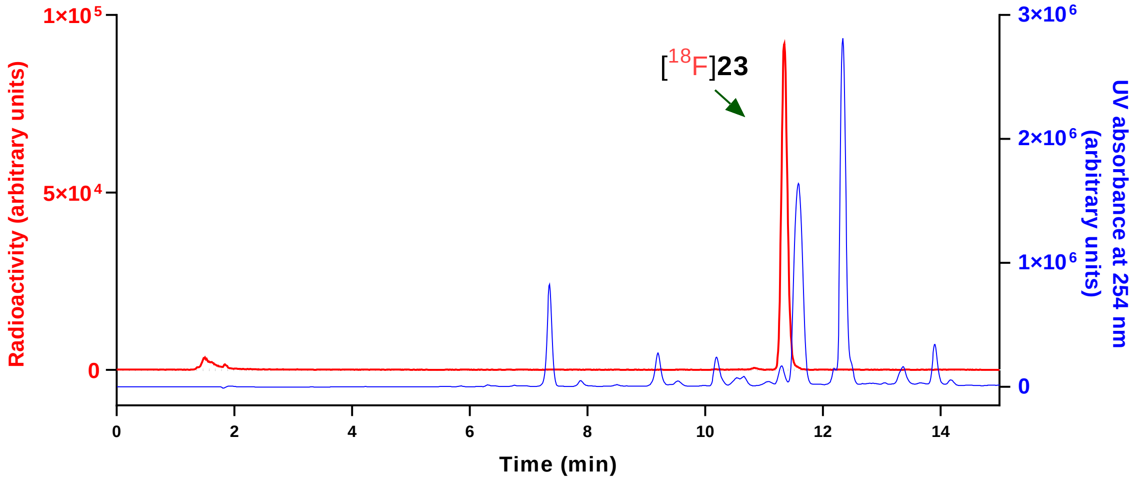

Supplementary Figure 81. Analytical HPLC chromatogram for reaction mixture of [^{18}F]**23**.

Supplementary Table 33. Radiosynthesis of [^{18}F]**23**.

Run	Retention time (min)	Yield (decay-corrected) (%)	Mean yield ± SD (%)	Isolated yield (%)
1	11.369	89	89 ± 1	84
2	11.347	90		
3	11.335	89		

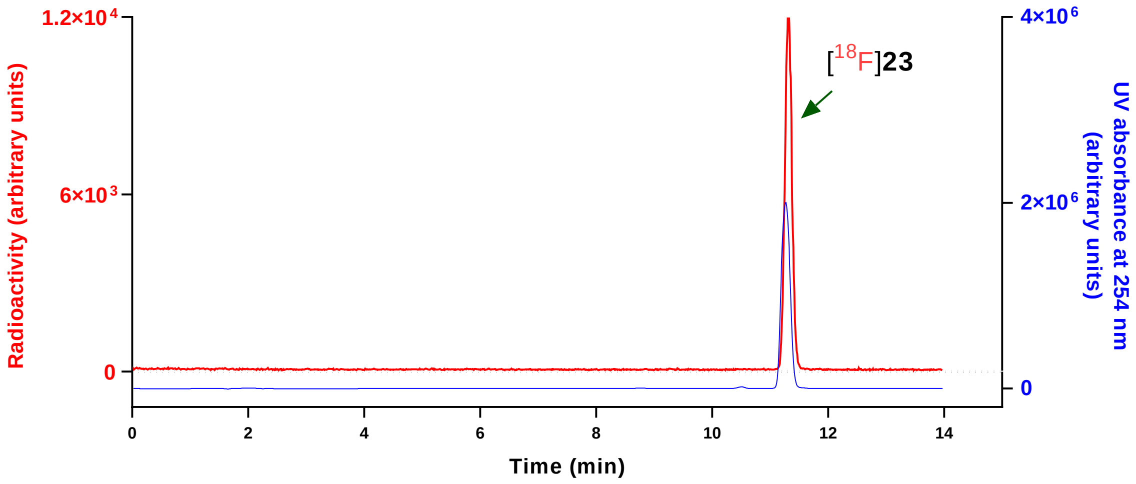

Supplementary Figure 82. Analytical HPLC chromatogram for [^{18}F]**23** with co–injected **23**.

HPLC analysis of [^{18}F]24

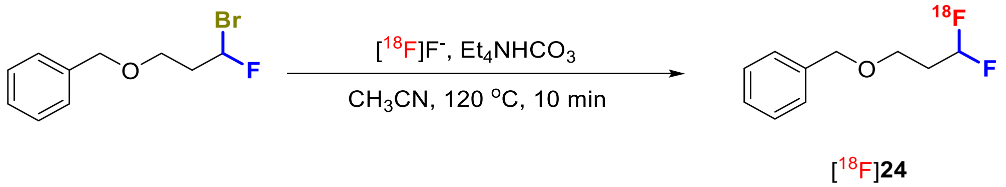

Prepared following general procedure 8 and analyzed using HPLC condition A.

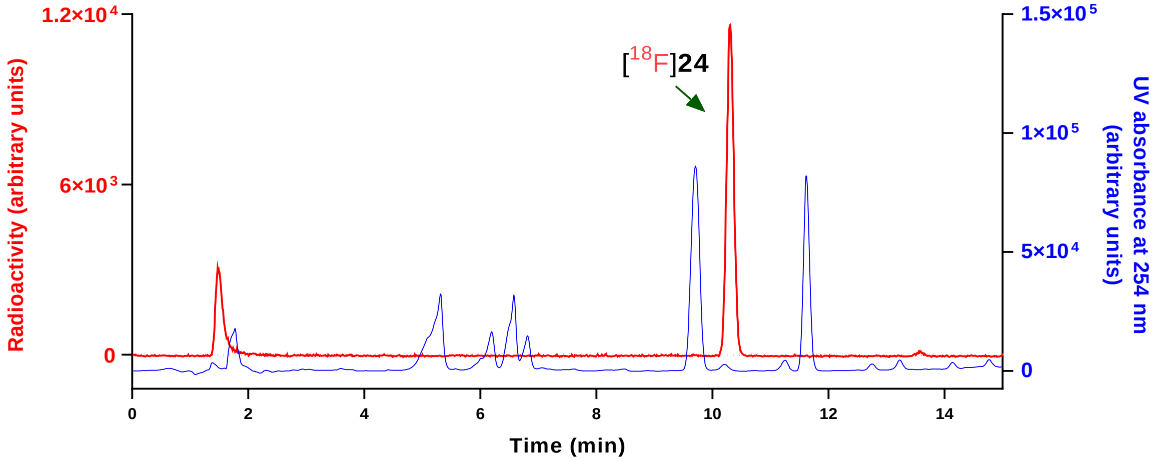

Supplementary Figure 83. Analytical HPLC chromatogram for reaction mixture of [^{18}F]**24**.

Supplementary Table 34. Radiosynthesis of [^{18}F]**24**.

Run	Retention time (min)	Yield (decay-corrected) (%)	Mean yield ± SD (%)	Isolated yield (%)
1	10.326	75	76 ± 1	67
2	10.339	76		
3	10.304	76		

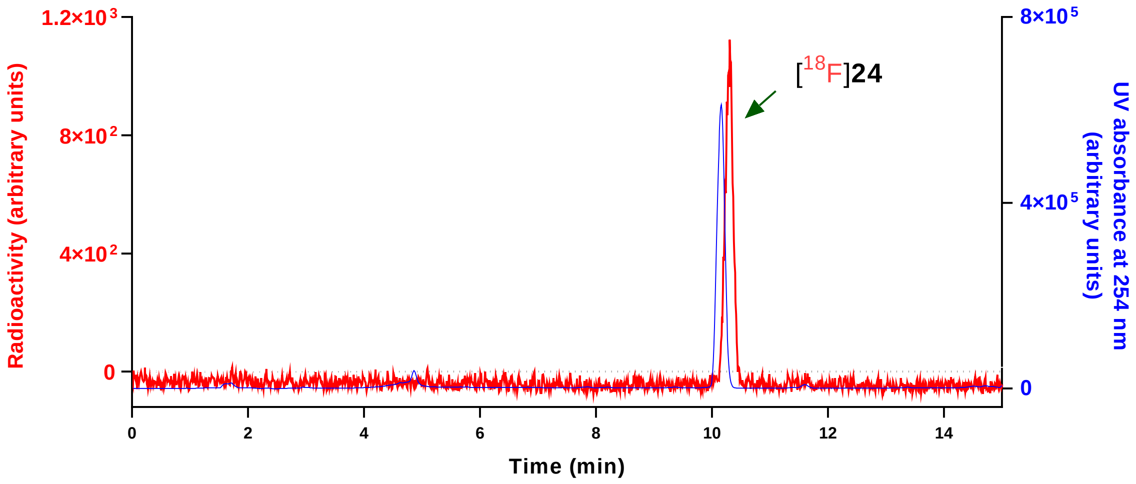

Supplementary Figure 84. Analytical HPLC chromatogram for [^{18}F]**24** with co-injected **24**.

HPLC analysis of [^{18}F]25

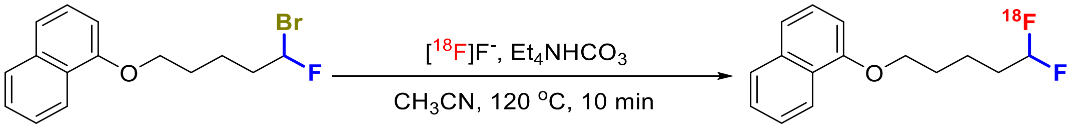

[^{18}F]**25**

Prepared following general procedure 8 and analyzed using HPLC condition A.

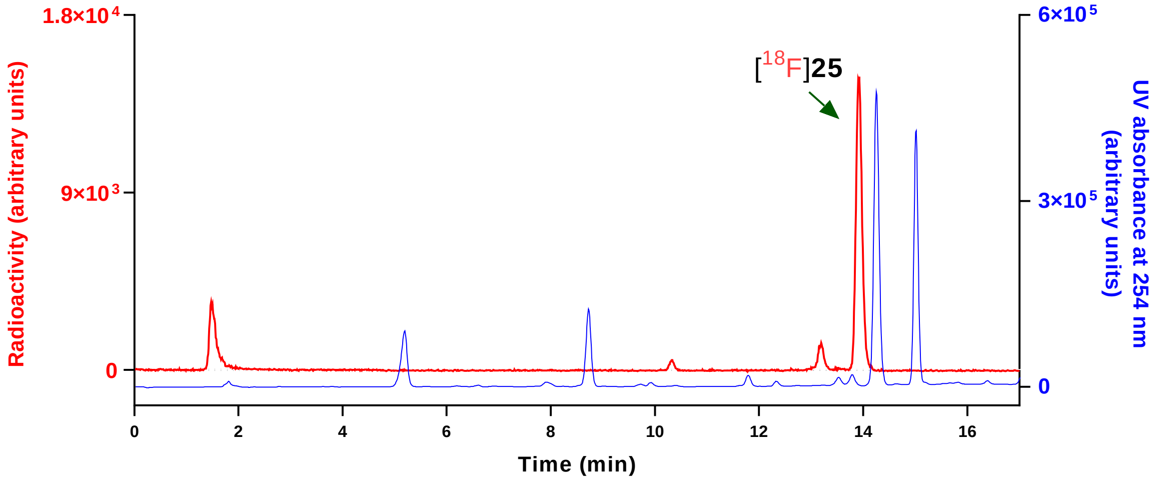

Supplementary Figure 85. Analytical HPLC chromatogram for reaction mixture of [^{18}F]**25**.

**Supplementary Table 35.** Radiosynthesis of [^{18}F]**25**.				
Run	Retention time (min)	Yield (decay-corrected) (%)	Mean yield ± SD (%)	Isolated yield (%)
1	13.911	69	72 ± 3	62
2	13.905	71		
3	13.930	75		

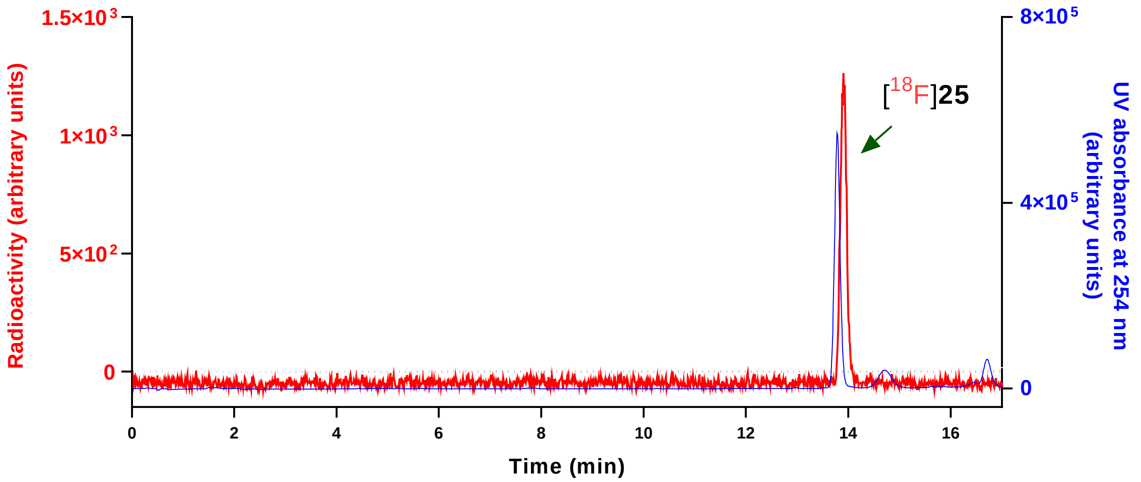

Supplementary Figure 86. Analytical HPLC chromatogram for [^{18}F]**25** with co–injected **25**.

HPLC analysis of [^{18}F]26

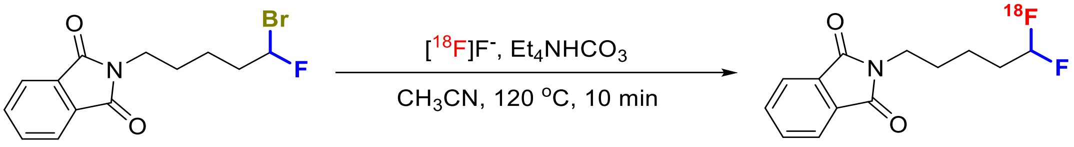

[^{18}F]**26**

Prepared following general procedure 8 and analyzed using HPLC condition A.

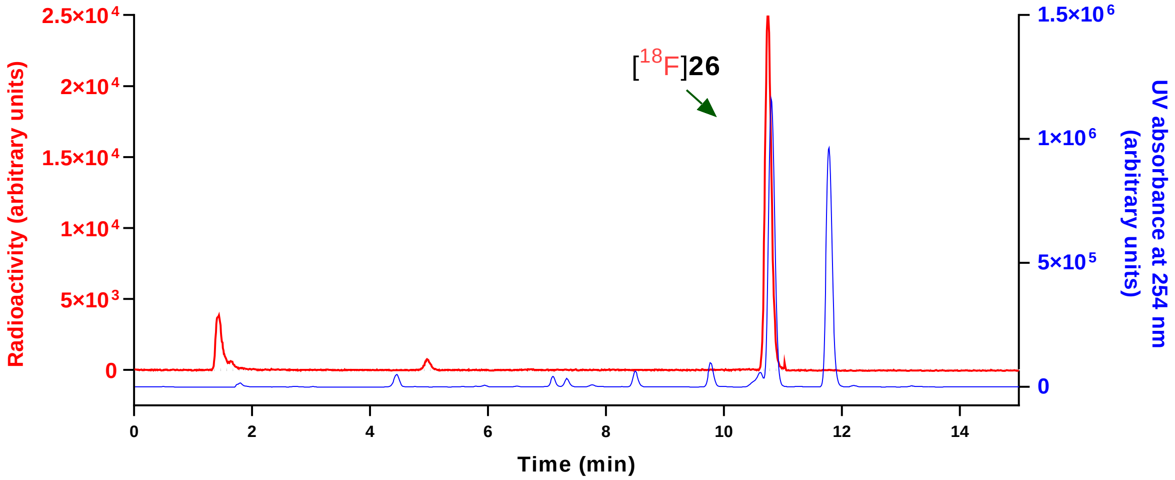

Supplementary Figure 87. Analytical HPLC chromatogram for reaction mixture of [^{18}F]**26**.

Supplementary Table 36. Radiosynthesis of [^{18}F]**26**.

Run	Retention time (min)	Yield (decay-corrected) (%)	Mean yield ± SD (%)	Isolated yield (%)
1	9.436	70	72 ± 2	65
2	9.465	73		
3	9.422	72		

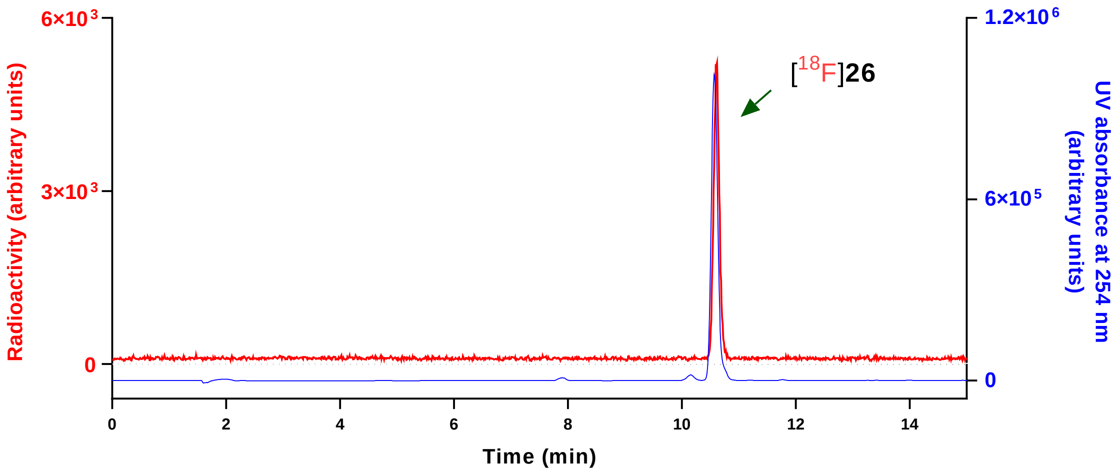

Supplementary Figure 88. Analytical HPLC chromatogram for [^{18}F]**26** with co–injected **26**.

HPLC analysis of [^{18}F]27

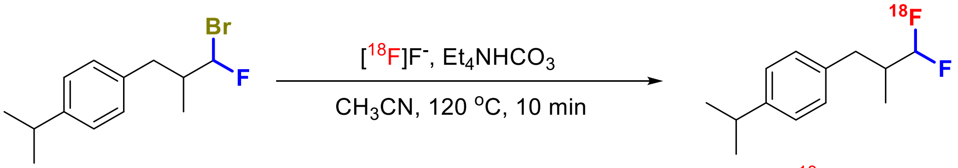

[^{18}F]**27**

Prepared following general procedure 8 and analyzed using HPLC condition A.

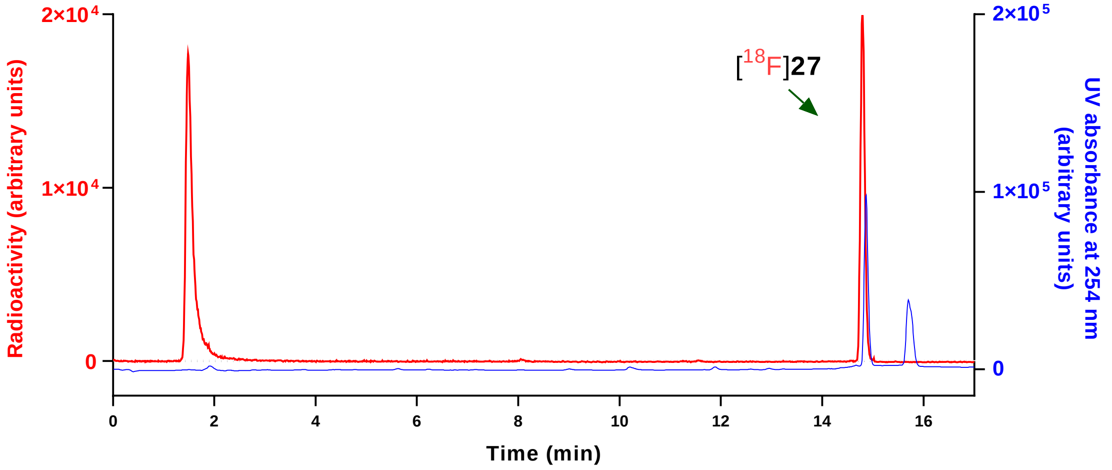

Supplementary Figure 89. Analytical HPLC chromatogram for reaction mixture of [^{18}F]**27**.

Supplementary Table 37. Radiosynthesis of [^{18}F]**27**.

Run	Retention time (min)	Yield (decay-corrected) (%)	Mean yield ± SD (%)	Isolated yield (%)
1	14.801	33	37 ± 4	38
2	14.794	40		
3	14.797	37		

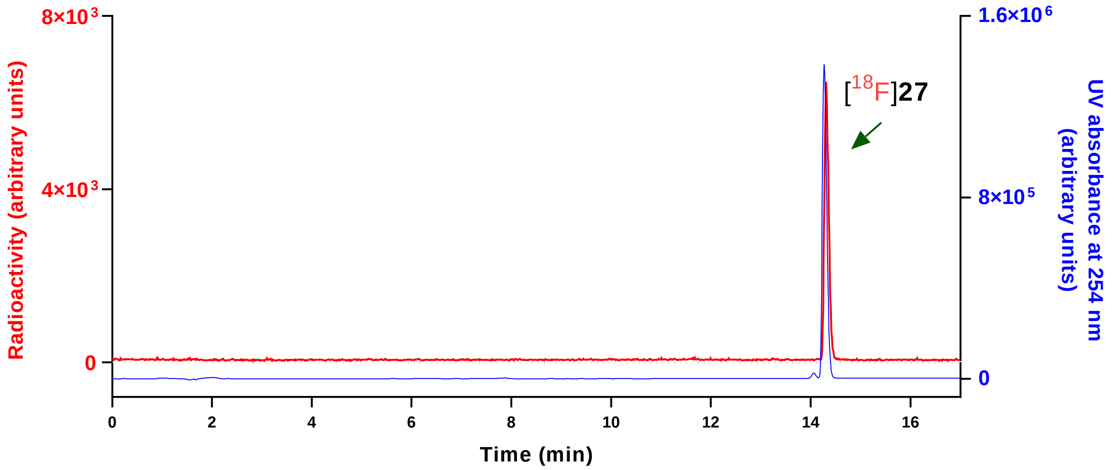

Supplementary Figure 90. Analytical HPLC chromatogram for [^{18}F]**27** with co–injected **27**.

HPLC analysis of [^{18}F]28

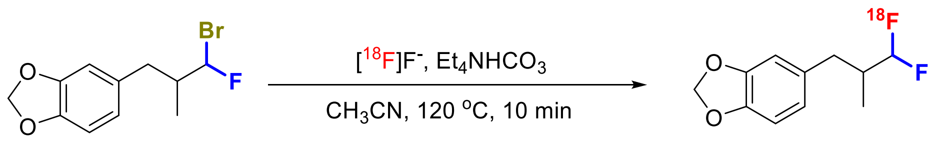

Prepared following general procedure 8 and analyzed using HPLC condition A.

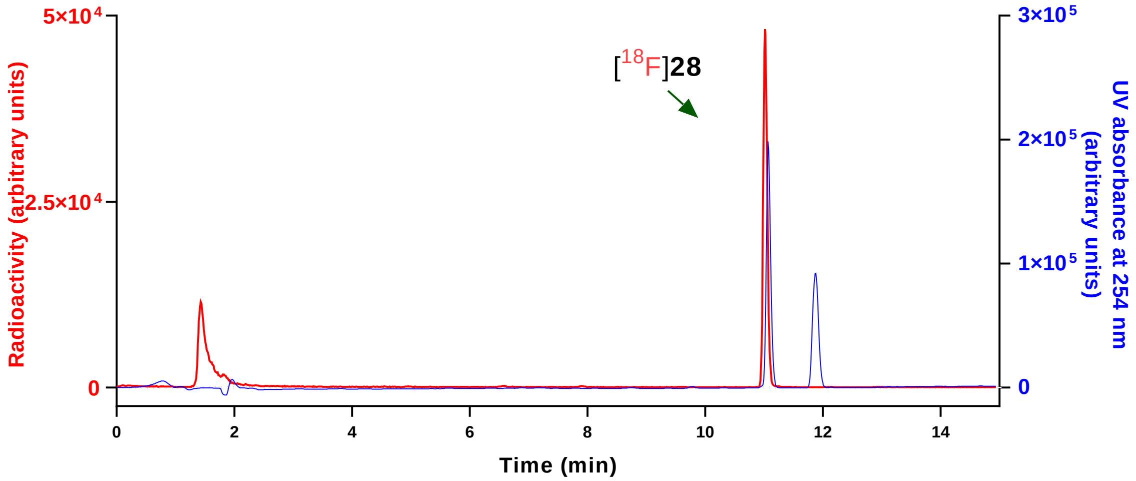

Supplementary Figure 91. Analytical HPLC chromatogram for reaction mixture of [^{18}F]**28**.

Supplementary Table 38. Radiosynthesis of [^{18}F]**28**.

Run	Retention time (min)	Yield (decay-corrected) (%)	Mean yield ± SD (%)	Isolated yield (%)
1	11.019	62	57 ± 4	54
2	10.995	55		
3	11.065	54		

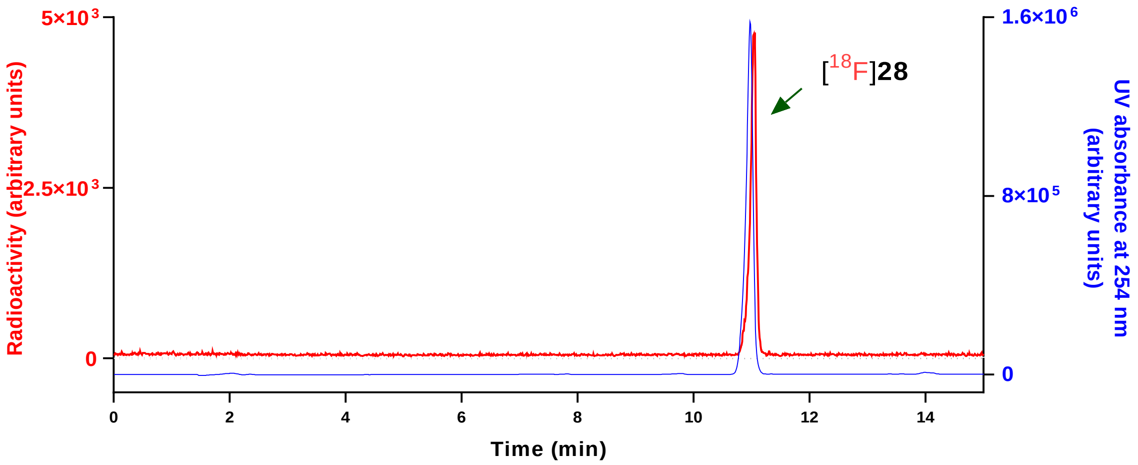

Supplementary Figure 92. Analytical HPLC chromatogram for [^{18}F]**28** with co–injected **28**.

HPLC analysis of [^{18}F]29

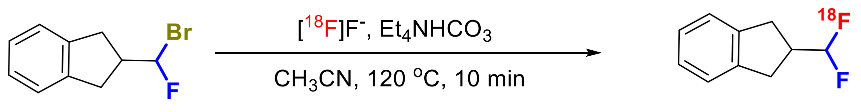

[^{18}F]**29**

Prepared following general procedure 8 and analyzed using HPLC condition A.

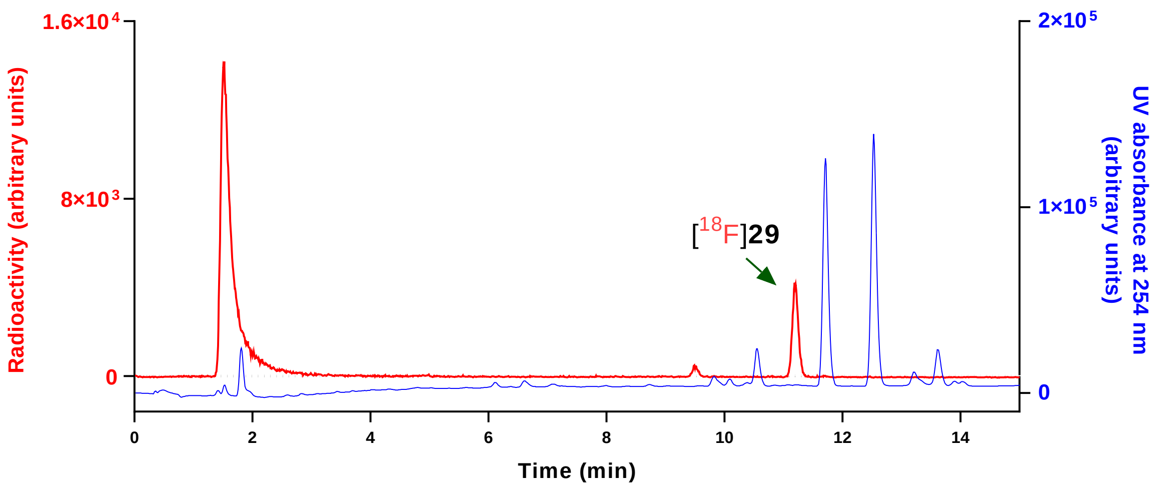

Supplementary Figure 93. Analytical HPLC chromatogram for reaction mixture of [^{18}F]**29**.

Supplementary Table 39. Radiosynthesis of [^{18}F]**29**.

Run	Retention time (min)	Yield (decay-corrected) (%)	Mean yield ± SD (%)	Isolated yield (%)
1	11.192	12	13 ± 1	11
2	11.217	14		
3	11.225	13		

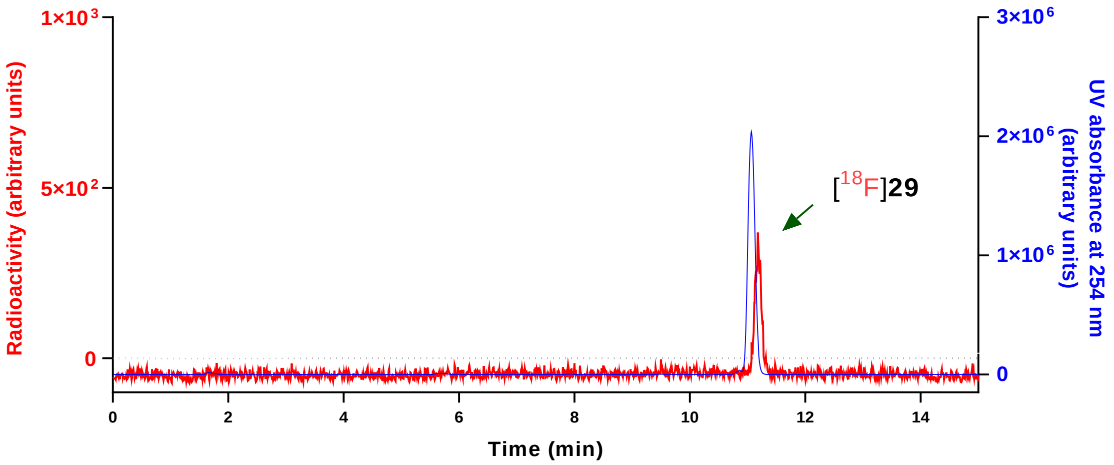

Supplementary Figure 94. Analytical HPLC chromatogram for [^{18}F]**29** with co–injected **29**.

HPLC analysis of [^{18}F]30

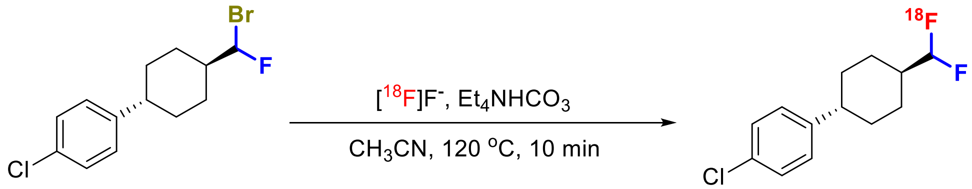

[^{18}F]**30**

Prepared following general procedure 8 and analyzed using HPLC condition A.

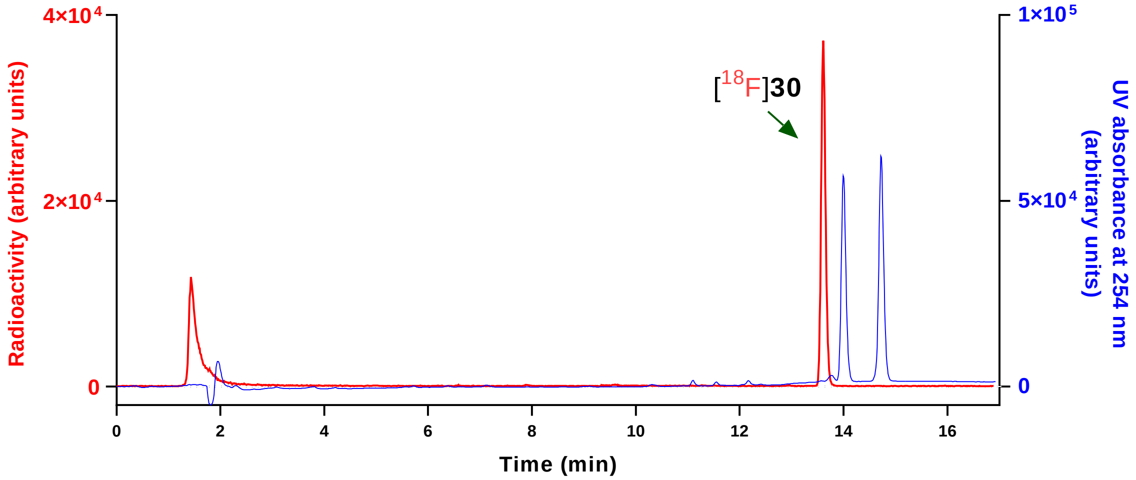

Supplementary Figure 95. Analytical HPLC chromatogram for reaction mixture of [^{18}F]**30**.

Supplementary Table 40. Radiosynthesis of [^{18}F]**30**.

Run	Retention time (min)	Yield (decay-corrected) (%)	Mean yield ± SD (%)	Isolated yield (%)
1	13.609	57	52 ± 5	47
2	13.601	51		
3	13.647	48		

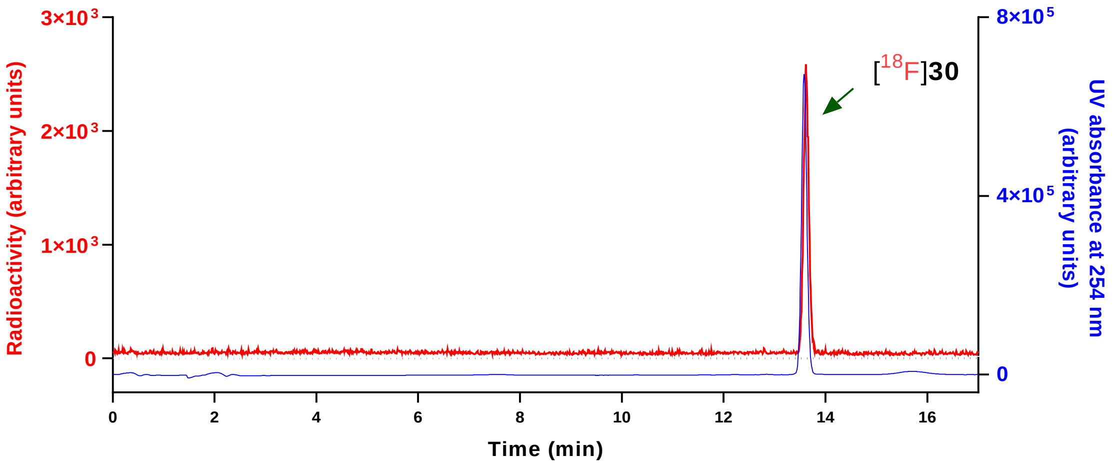

Supplementary Figure 96. Analytical HPLC chromatogram for [^{18}F]**30** with co–injected **30**.

HPLC analysis of [^{18}F]31

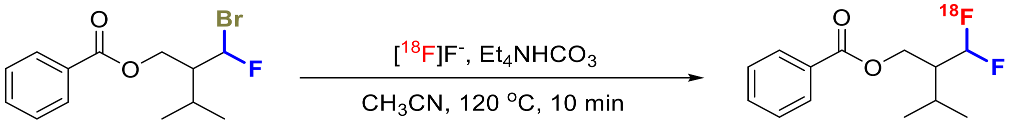

[^{18}F]**31**

Prepared following general procedure 8 and analyzed using HPLC condition A.

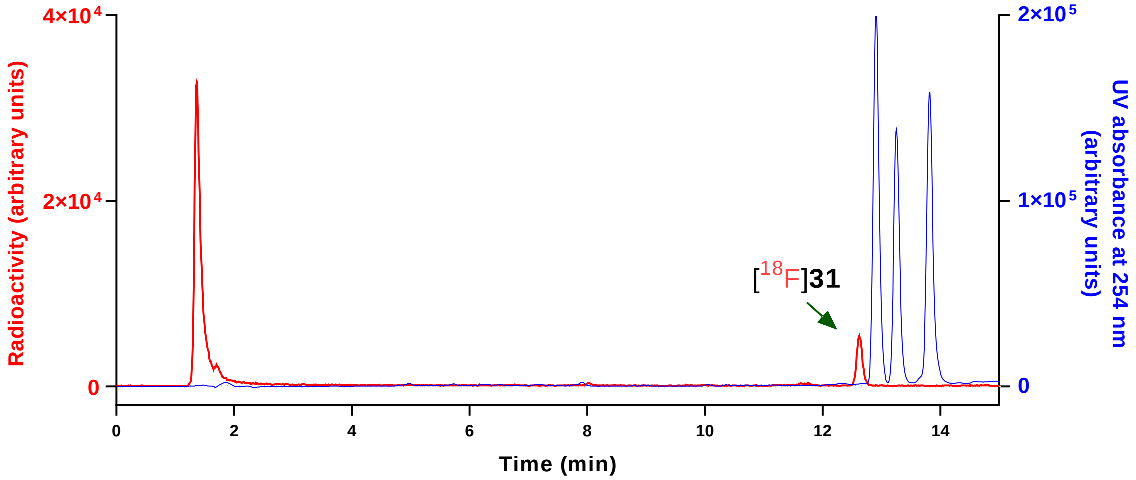

Supplementary Figure 97. Analytical HPLC chromatogram for reaction mixture of [^{18}F]**31**.

Supplementary Table 41. Radiosynthesis of [^{18}F]**31**.

Run	Retention time (min)	Yield (decay-corrected) (%)	Mean yield ± SD (%)	Isolated yield (%)
1	12.636	11	12 ± 1	10
2	12.639	11		
3	12.729	13		

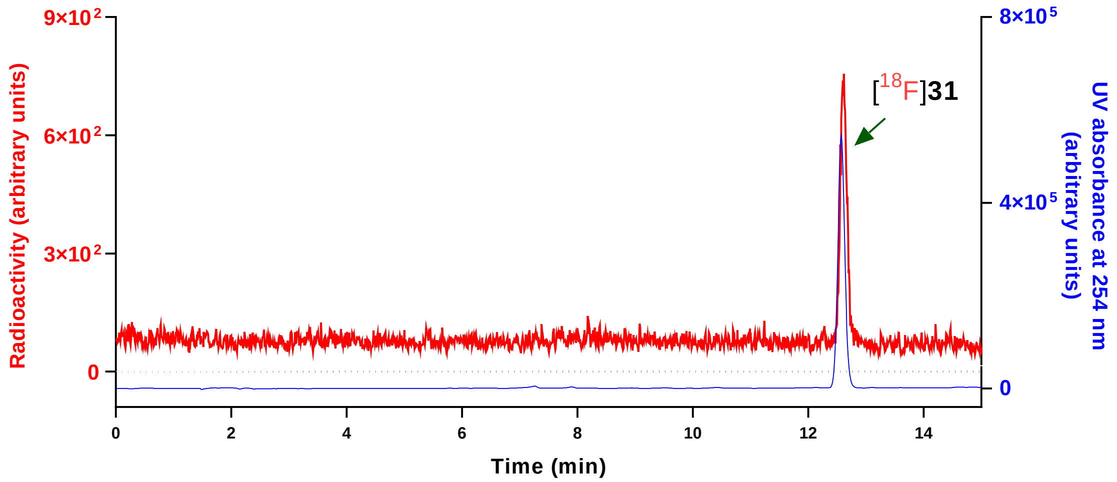

Supplementary Figure 98. Analytical HPLC chromatogram for [^{18}F]**31** with co–injected **31**.

HPLC analysis of [^{18}F]32

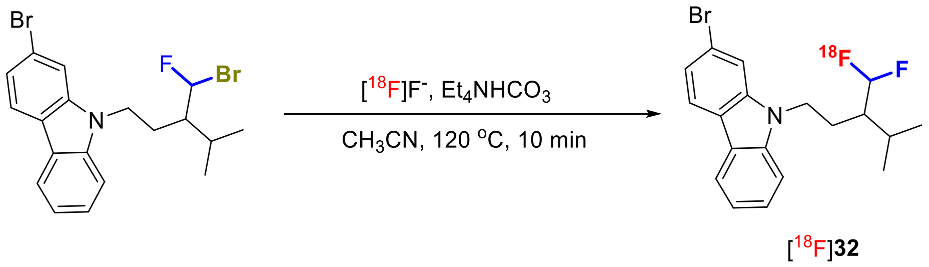

Prepared following general procedure 8 and analyzed using HPLC condition A.

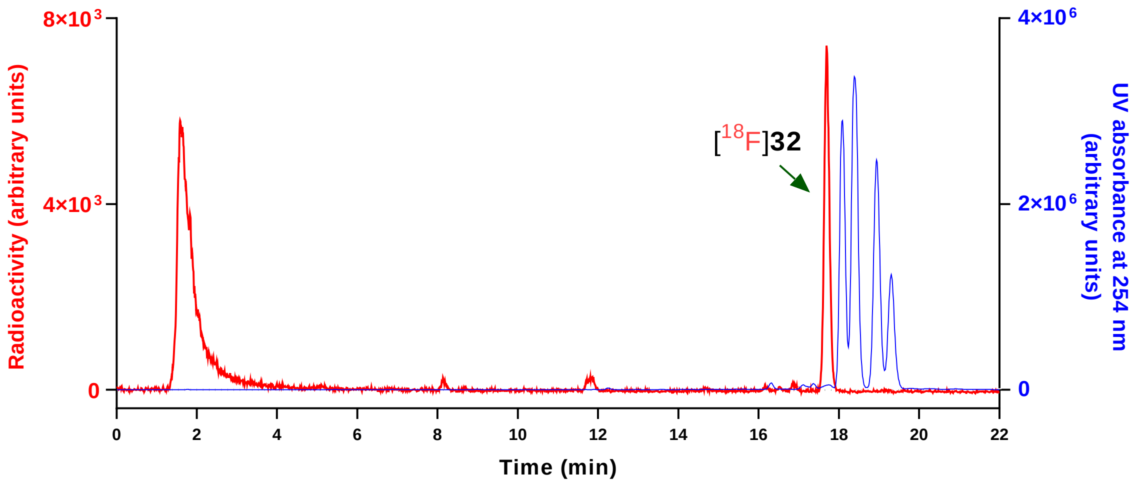

Supplementary Figure 99. Analytical HPLC chromatogram for reaction mixture of [^{18}F]**32**.

Supplementary Table 42. Radiosynthesis of [^{18}F]**32**.

Run	Retention time (min)	Yield (decay-corrected) (%)	Mean yield ± SD (%)	Isolated yield (%)
1	17.694	29	24 ± 4	18
2	17.688	22		
3	17.553	21		

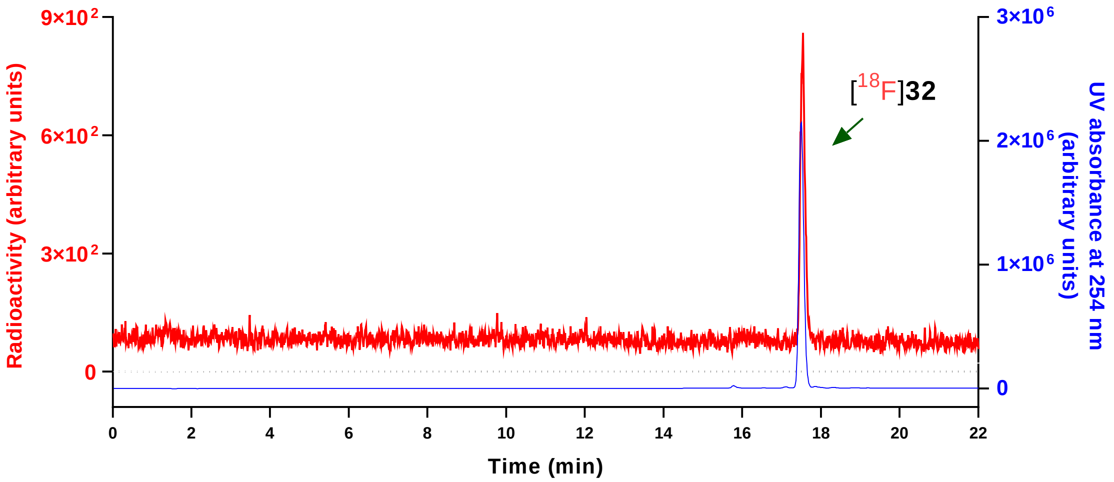

Supplementary Figure 100. Analytical HPLC chromatogram for [^{18}F]**32** with co–injected **32**.

HPLC analysis of [^{18}F]33

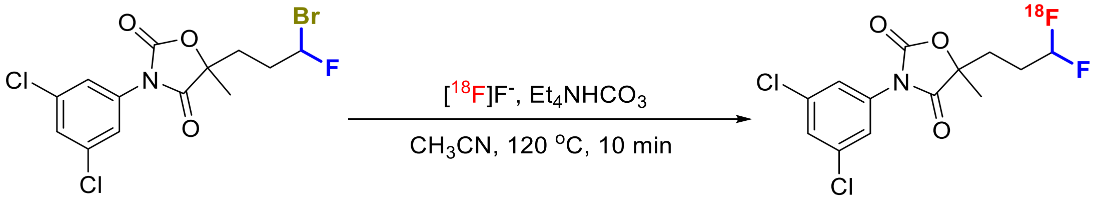

[^{18}F]**33**

Prepared following general procedure 8 and analyzed using HPLC condition A.

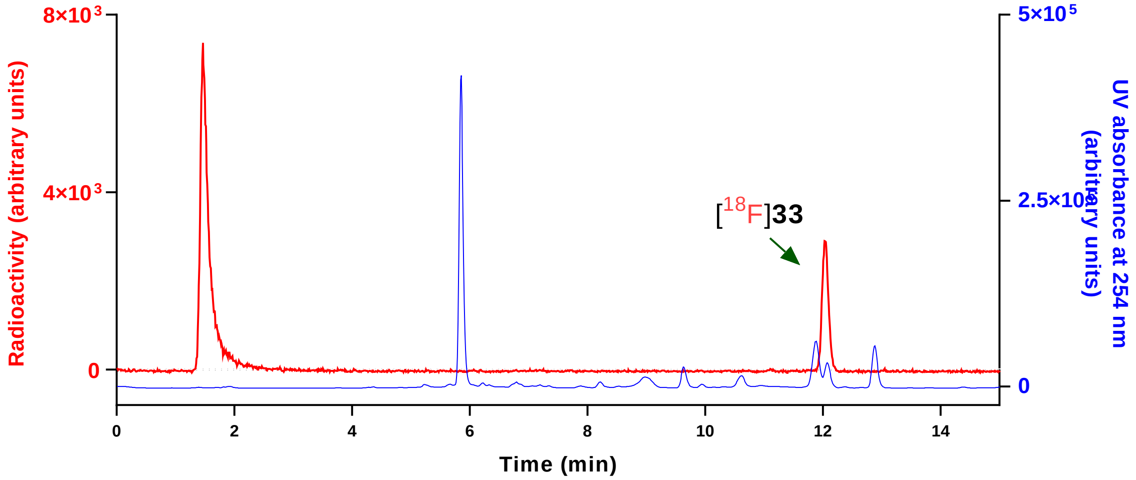

Supplementary Figure 101. Analytical HPLC chromatogram for reaction mixture of [^{18}F]**33**.

Supplementary Table 43. Radiosynthesis of [^{18}F]**33**.

Run	Retention time (min)	Yield (decay-corrected) (%)	Mean yield ± SD (%)	Isolated yield (%)
1	12.026	16	20 ± 4	19
2	12.037	22		
3	12.051	23		

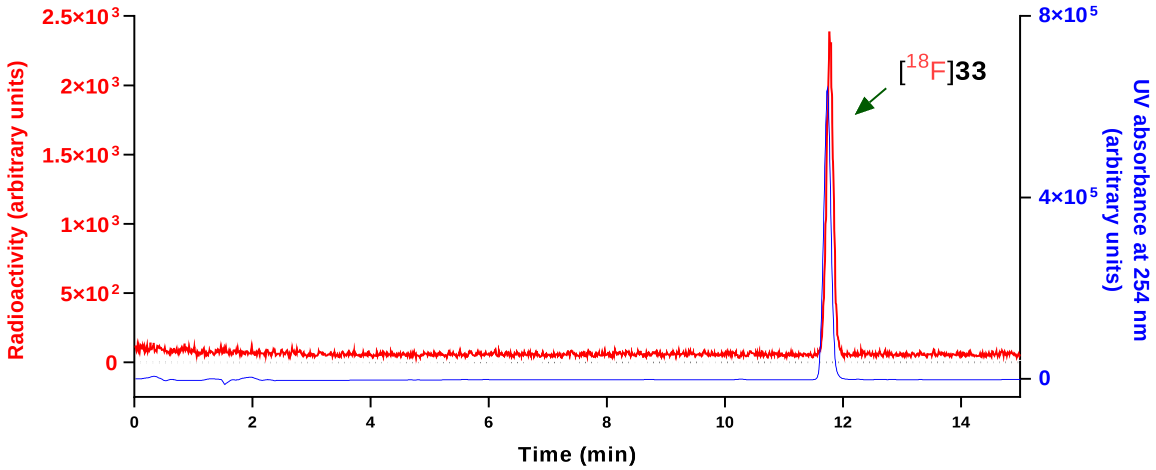

Supplementary Figure 102. Analytical HPLC chromatogram for [^{18}F]**33** with co–injected **33**.

HPLC analysis of [^{18}F]34

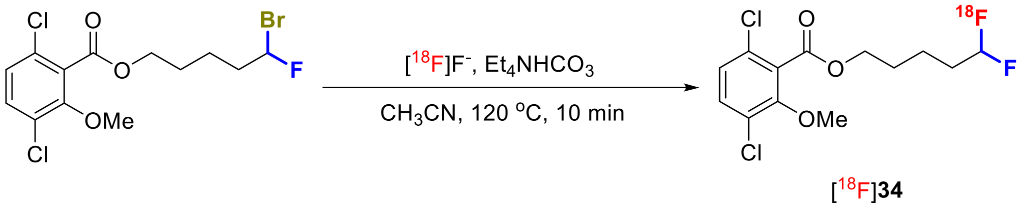

Prepared following general procedure 8 and analyzed using HPLC condition A.

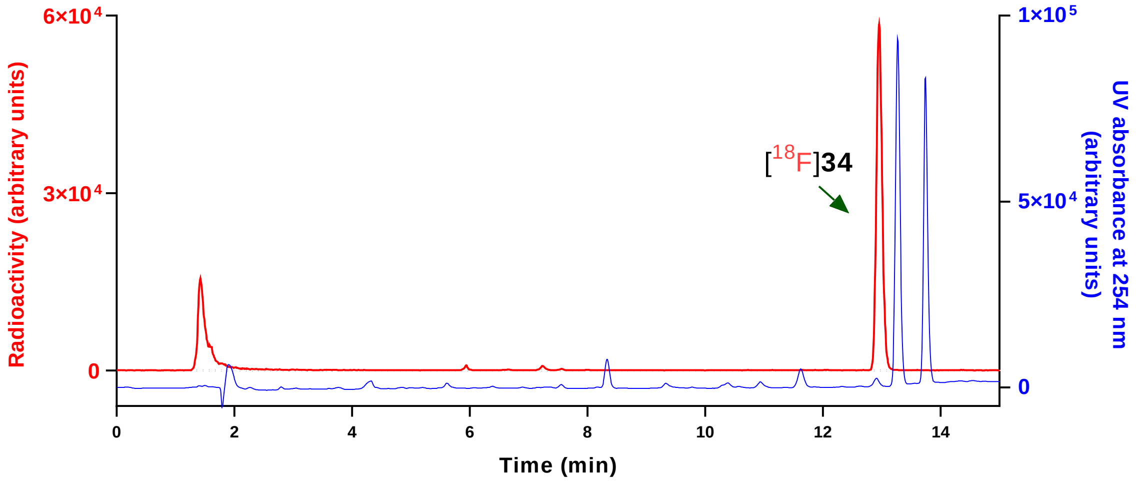

Supplementary Figure 103. Analytical HPLC chromatogram for reaction mixture of [^{18}F]**34**.

Supplementary Table 44. Radiosynthesis of [^{18}F]**34**.

Run	Retention time (min)	Yield (decay-corrected) (%)	Mean yield ± SD (%)	Isolated yield (%)
1	12.947	67	69 ± 2	62
2	12.956	69		
3	12.943	71		

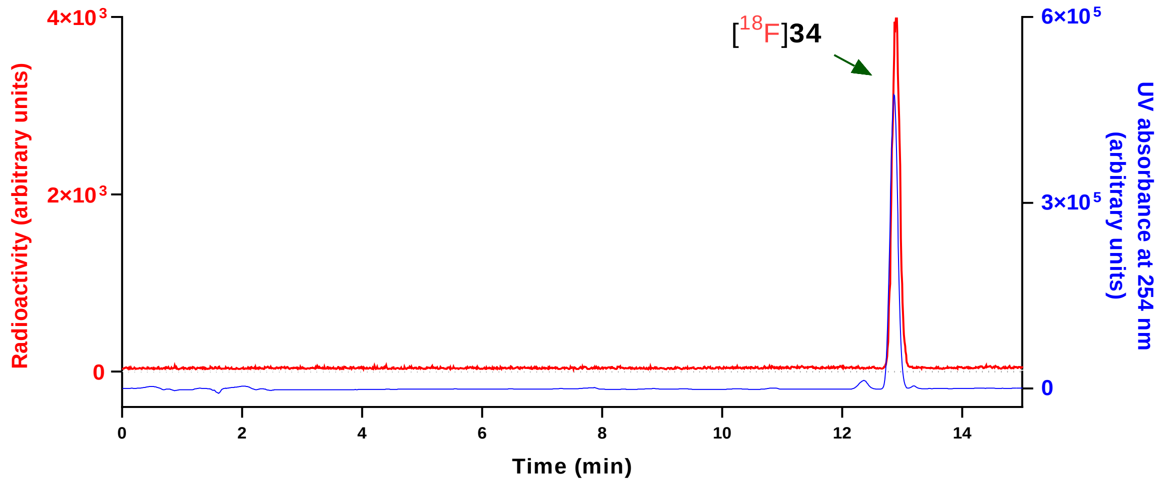

Supplementary Figure 104. Analytical HPLC chromatogram for [^{18}F]**34** with co–injected **34**.

HPLC analysis of [^{18}F]35

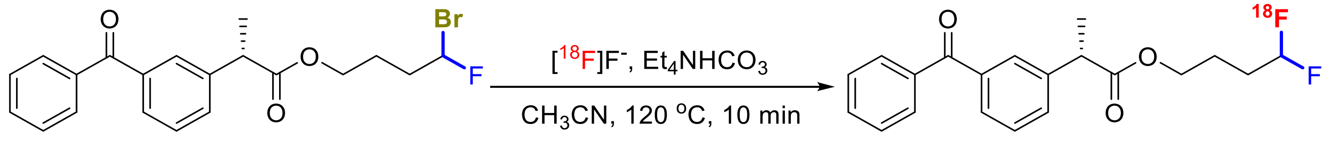

[^{18}F]**35**

Prepared following general procedure 8 and analyzed using HPLC condition A.

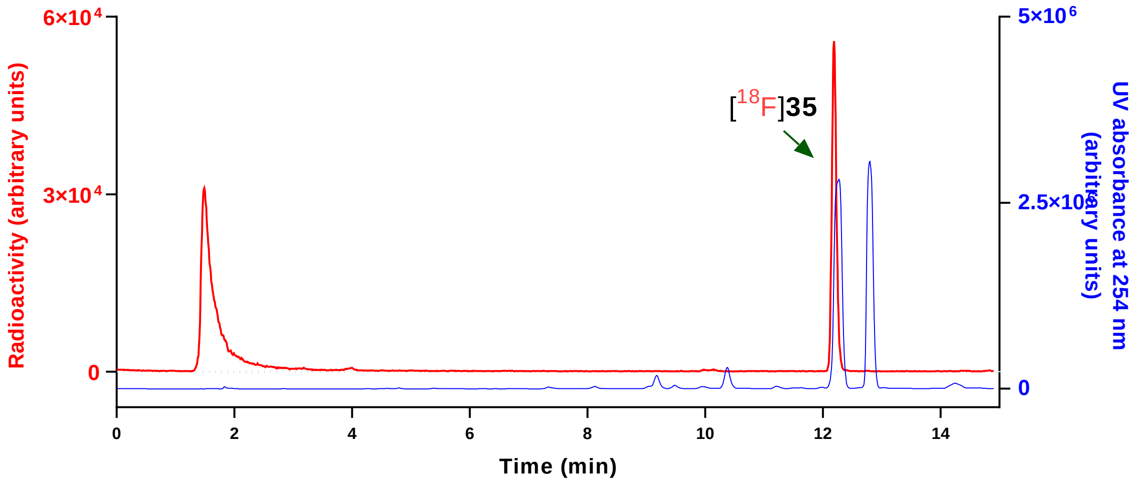

Supplementary Figure 105. Analytical HPLC chromatogram for reaction mixture of [^{18}F]**35**.

**Supplementary Table 45.** Radiosynthesis of [^{18}F]**35**.				
Run	Retention time (min)	Yield (decay-corrected) (%)	Mean yield ± SD (%)	Isolated yield (%)
1	12.191	37	38 ± 2	35
2	12.190	40		
3	12.195	38		

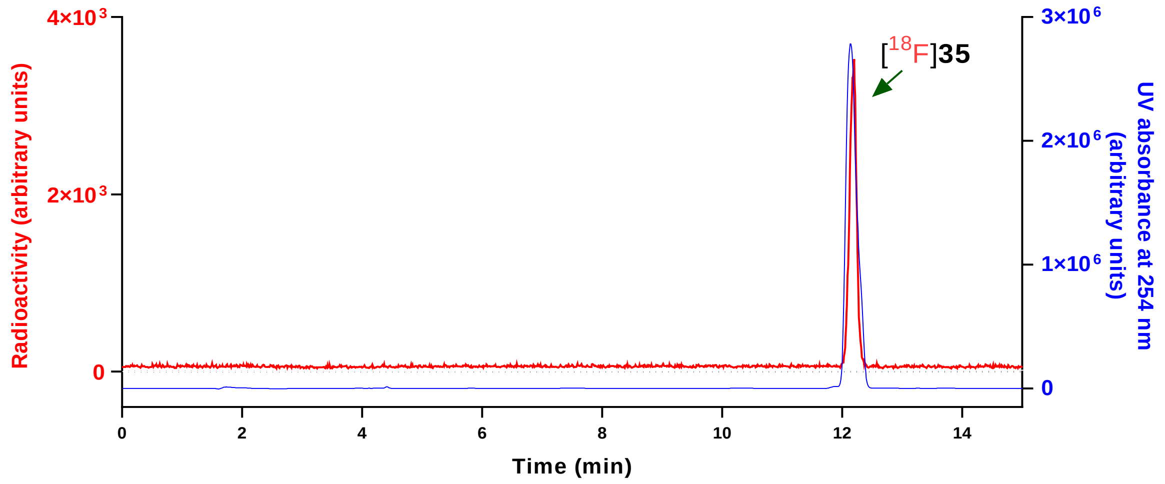

Supplementary Figure 106. Analytical HPLC chromatogram for [^{18}F]**35** with co–injected **35**.

HPLC analysis of [^{18}F]36

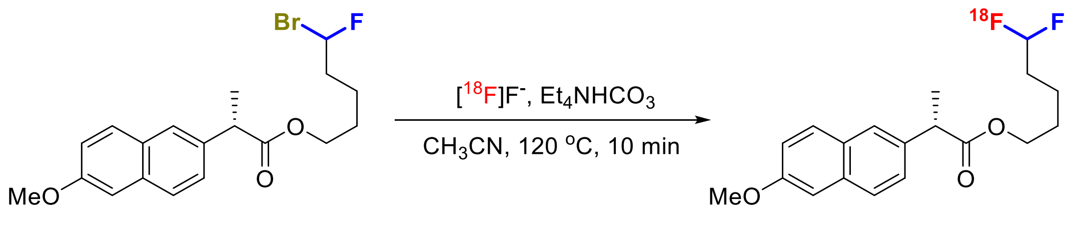

Prepared following general procedure 8 and analyzed using HPLC condition A.

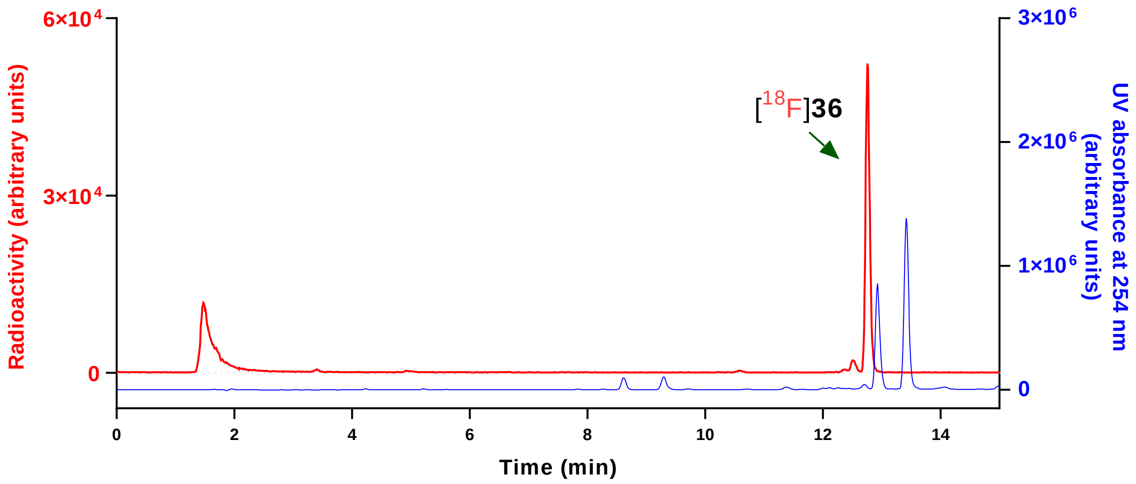

Supplementary Figure 107. Analytical HPLC chromatogram for reaction mixture of [^{18}F]**36**.

Supplementary Table 46. Radiosynthesis of [^{18}F]**36**.

Run	Retention time (min)	Yield (decay-corrected) (%)	Mean yield ± SD (%)	Isolated yield (%)
1	12.801	58	58 ± 2	52
2	12.761	56		
3	12.824	59		

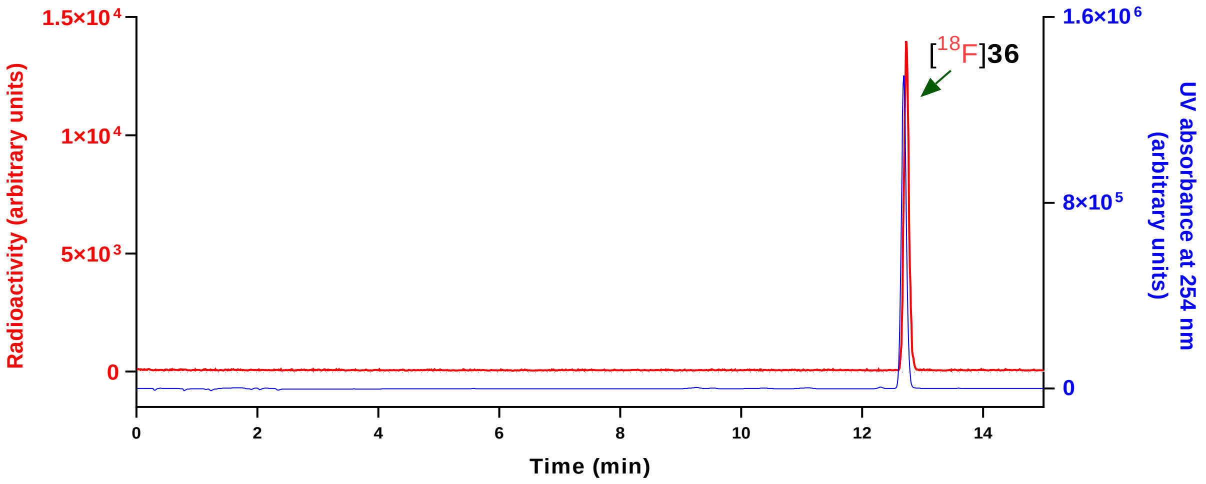

Supplementary Figure 108. Analytical HPLC chromatogram for [^{18}F]**36** with co–injected **36**.

HPLC analysis of [18F]37

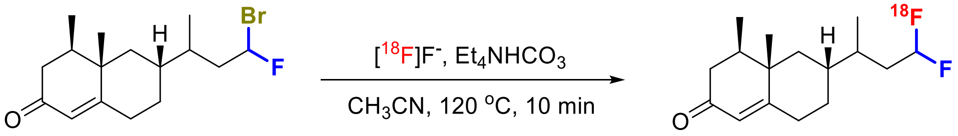

[18F]**37**

Prepared following general procedure 8 and analyzed using HPLC condition A.

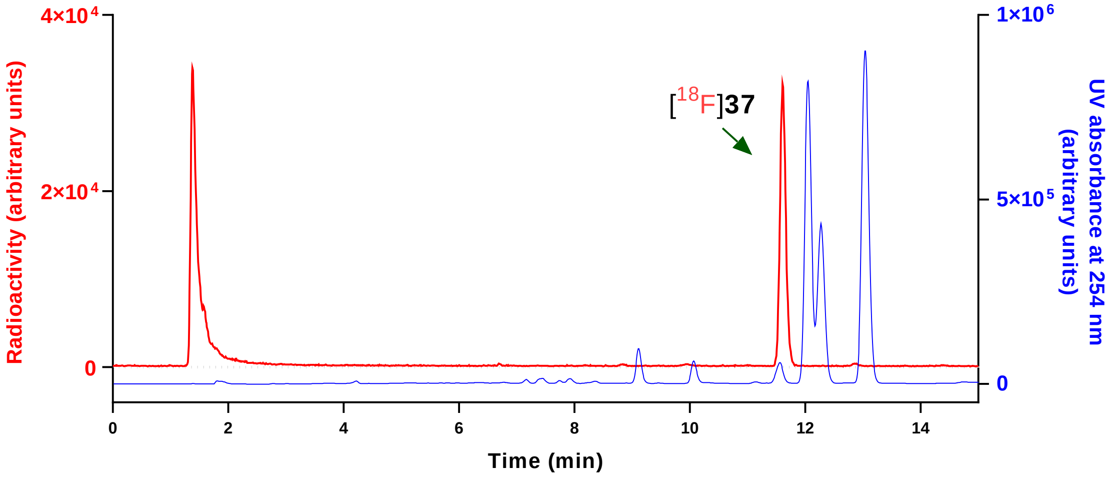

Supplementary Figure 109. Analytical HPLC chromatogram for reaction mixture of [18F]**37**.

Supplementary Table 47. Radiosynthesis of [18F]**37**.

Run	Retention time (min)	Yield (decay-corrected) (%)	Mean yield ± SD (%)	Isolated yield (%)
1	11.614	42	41 ± 1	39
2	11.610	40		
3	11.616	40		

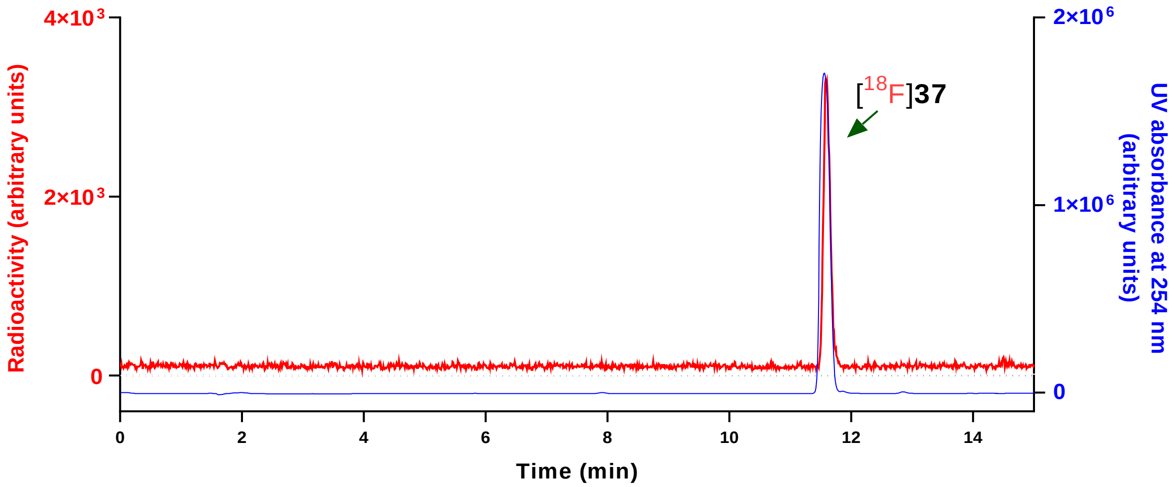

Supplementary Figure 110. Analytical HPLC chromatogram for [^{18}F]**37** with co–injected **37**.

HPLC analysis of [^{18}F]38

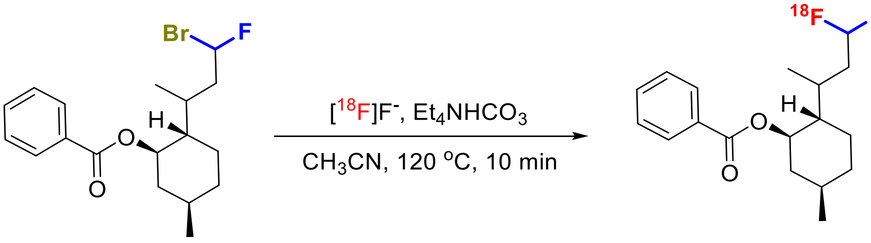

[^{18}F]**38**

Prepared following general procedure 8 and analyzed using HPLC condition A.

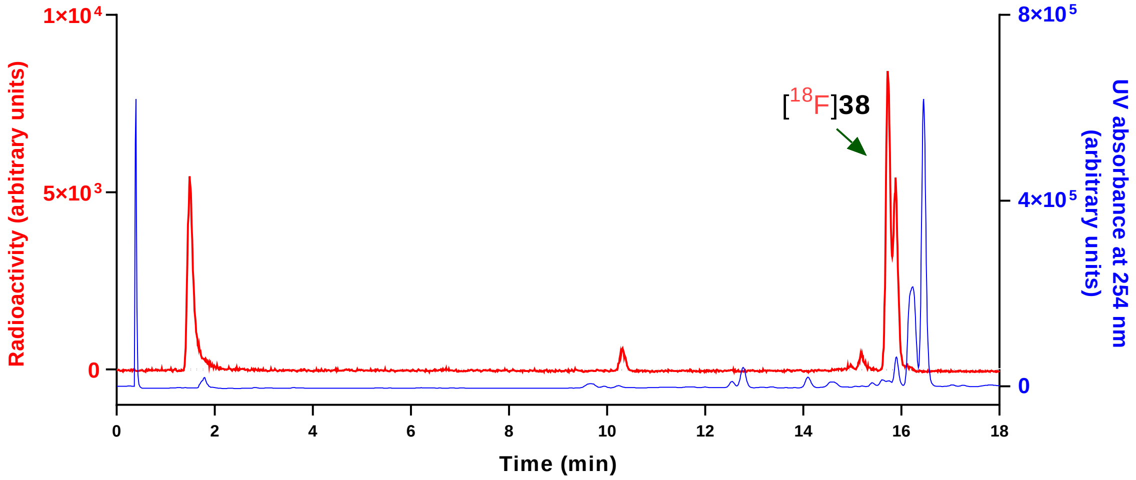

Supplementary Figure 111. Analytical HPLC chromatogram for reaction mixture of [^{18}F]**38**.

Supplementary Table 48. Radiosynthesis of [^{18}F]**38**.

Run	Retention time (min)	Yield (decay-corrected) (%)	Mean yield ± SD (%)	Isolated yield (%)
1	15.168, 15.329	64	64 ± 4	54
2	15.726, 15.885	60		
3	15.682, 15.831	68		

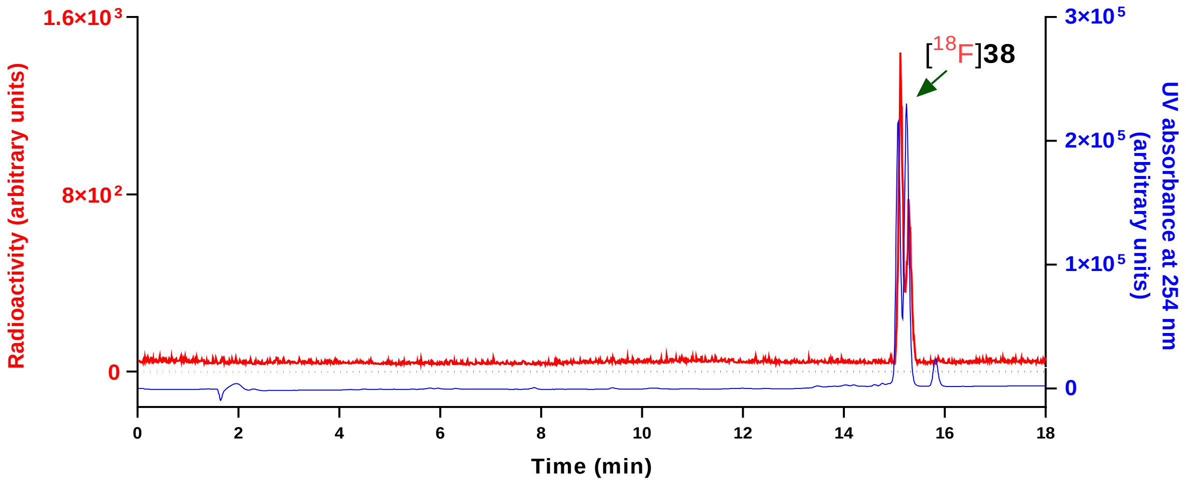

Supplementary Figure 112. Analytical HPLC chromatogram for [^{18}F]**38** with co–injected **38**.

HPLC analysis of [^{18}F]39

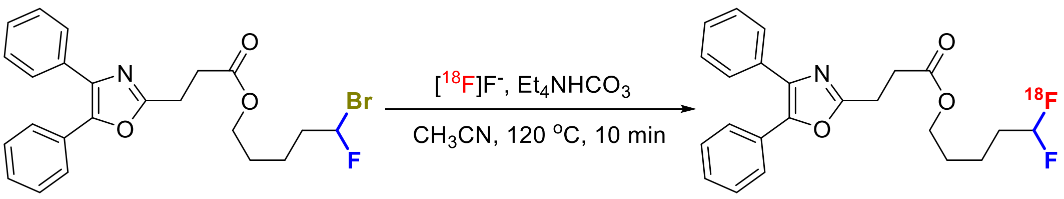

[^{18}F]39

Prepared following general procedure 8 and analyzed using HPLC condition A.

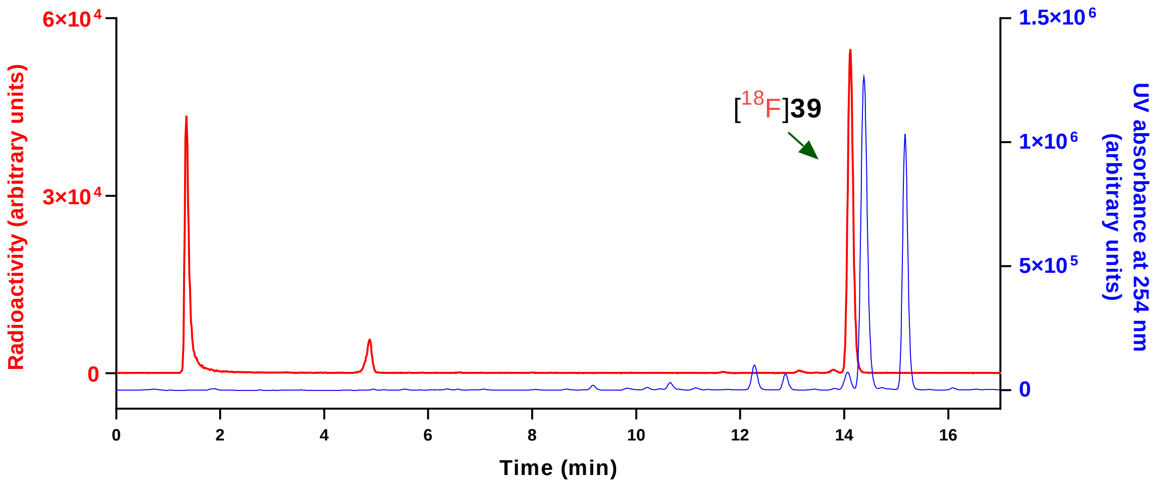

Supplementary Figure 113. Analytical HPLC chromatogram for reaction mixture of [^{18}F]**39**.

**Supplementary Table 49.** Radiosynthesis of [^{18}F]**39**.				
Run	Retention time (min)	Yield (decay-corrected) (%)	Mean yield ± SD (%)	Isolated yield (%)
1	14.101	52	54 ± 2	50
2	14.087	54		
3	14.116	55		

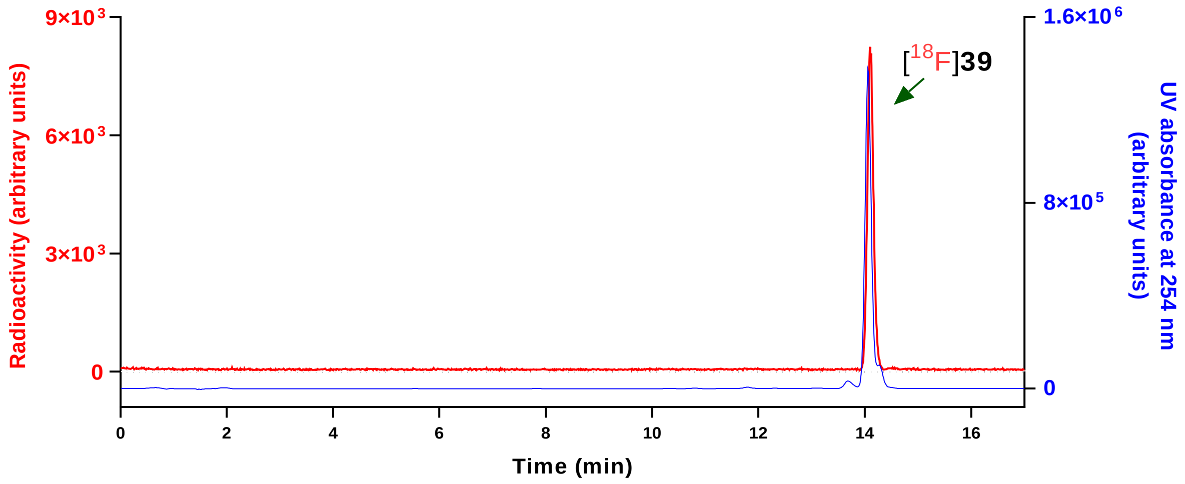

Supplementary Figure 114. Analytical HPLC chromatogram for [^{18}F]**39** with co–injected **39**.

HPLC analysis of [^{18}F]40

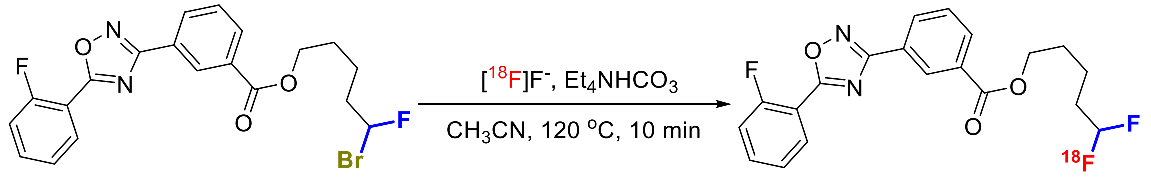

Prepared following general procedure 8 and analyzed using HPLC condition A.

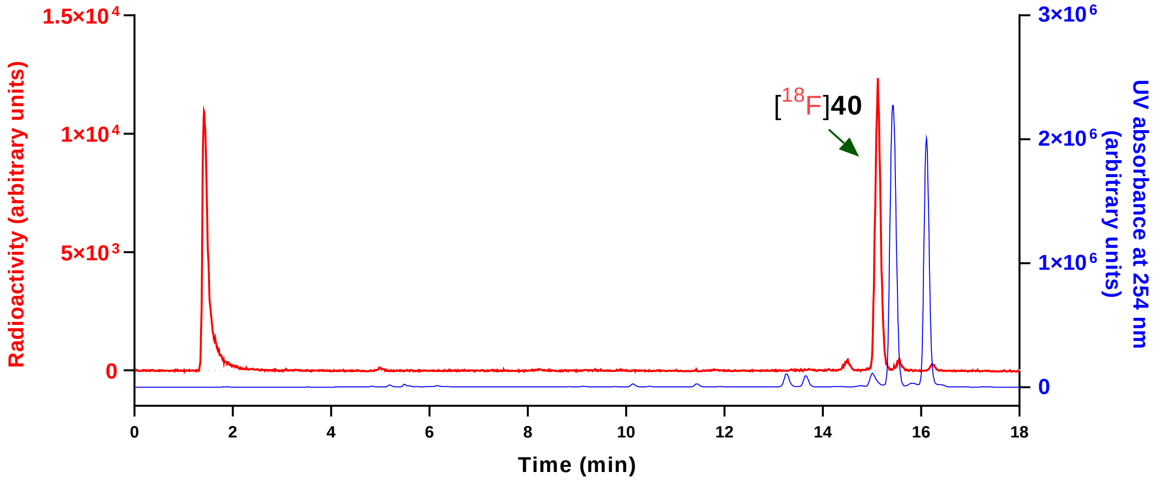

Supplementary Figure 115. Analytical HPLC chromatogram for reaction mixture of [^{18}F]**40**.

Supplementary Table 50. Radiosynthesis of [^{18}F]**40**.

Run	Retention time (min)	Yield (decay-corrected) (%)	Mean yield ± SD (%)	Isolated yield (%)
1	15.124	47	47 ± 2	35
2	15.119	48		
3	15.124	45		

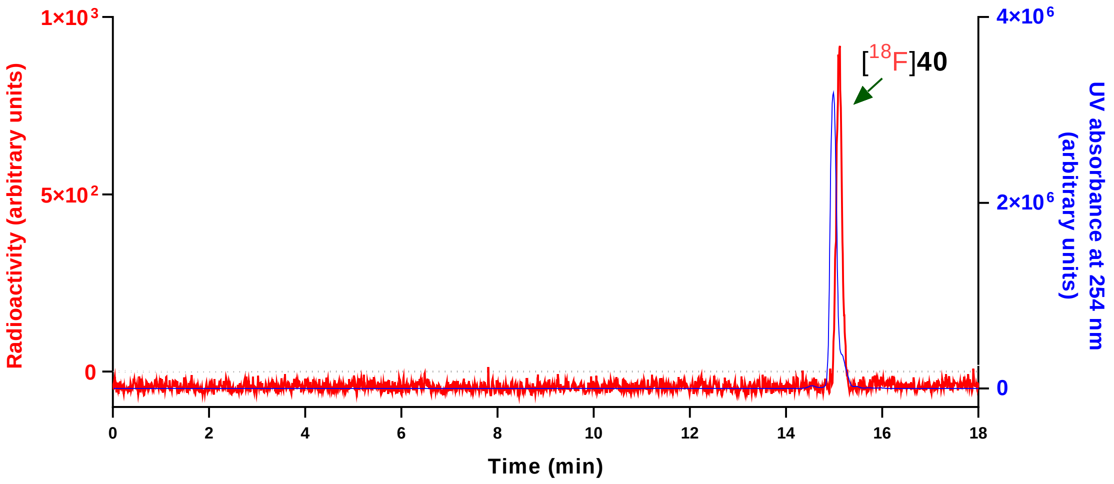

Supplementary Figure 116. Analytical HPLC chromatogram for [^{18}F]**40** with co–injected **40**.

HPLC analysis of [^{18}F]41

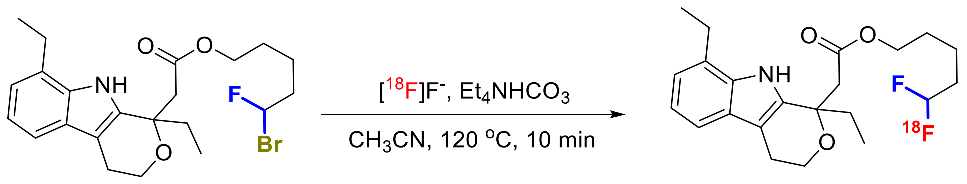

Prepared following general procedure 8 and analyzed using HPLC condition A.

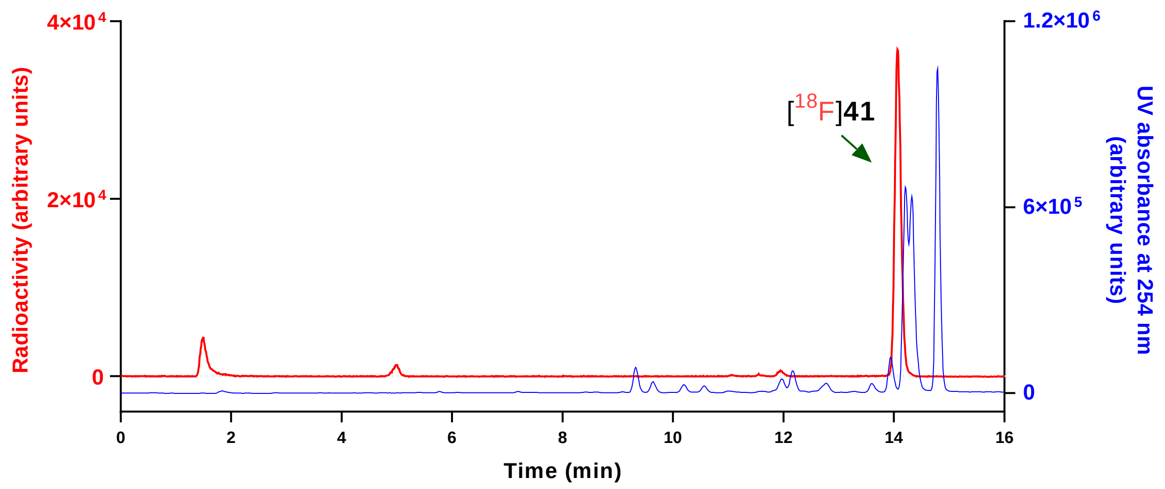

Supplementary Figure 117. Analytical HPLC chromatogram for reaction mixture of [^{18}F]**41**.

**Supplementary Table 51.** Radiosynthesis of [^{18}F]**41**.				
Run	Retention time (min)	Yield (decay-corrected) (%)	Mean yield ± SD (%)	Isolated yield (%)
1	14.068	83	84 ± 1	74
2	14.044	84		
3	14.069	84		

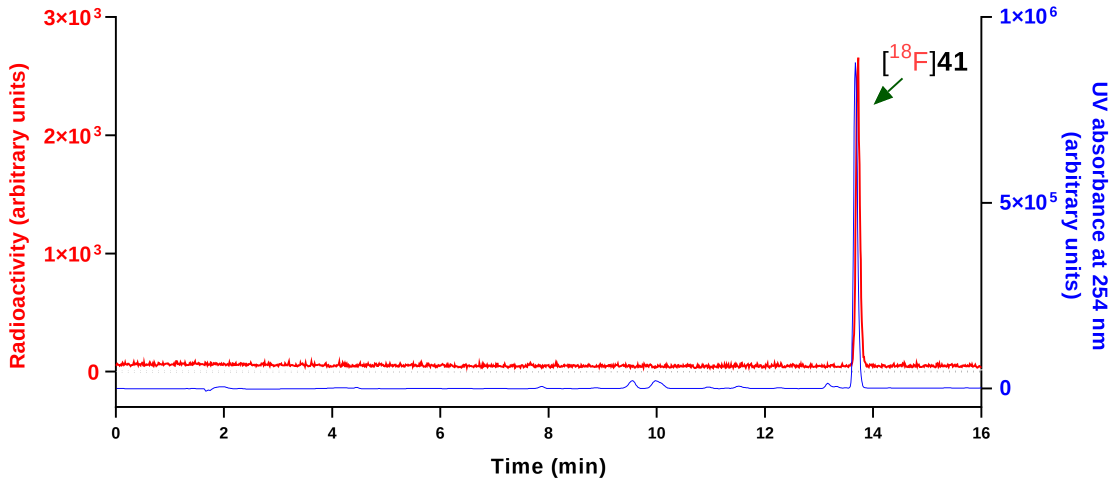

Supplementary Figure 118. Analytical HPLC chromatogram for [^{18}F]**41** with co-injected **41**.

HPLC analysis of [^{18}F]42

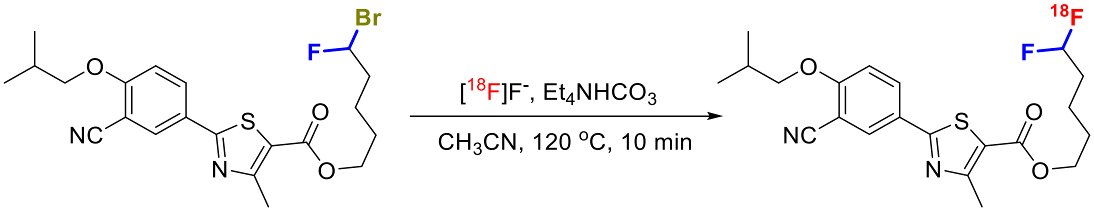

[^{18}F]**42**

Prepared following general procedure 8 and analyzed using HPLC condition A.

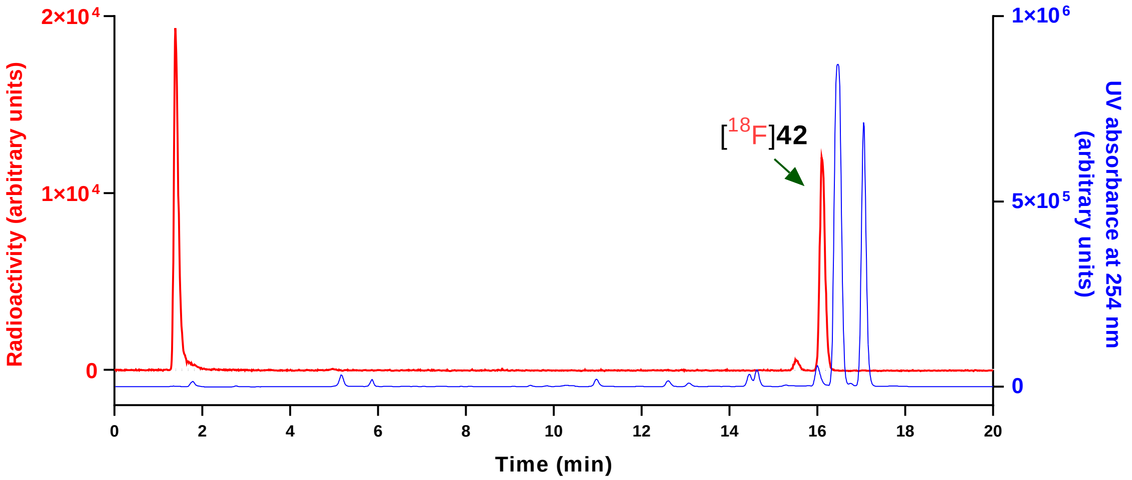

Supplementary Figure 119. Analytical HPLC chromatogram for reaction mixture of [^{18}F]**42**.

Supplementary Table 52. Radiosynthesis of [^{18}F]**42**.

Run	Retention time (min)	Yield (decay-corrected) (%)	Mean yield ± SD (%)	Isolated yield (%)
1	16.103	40	41 ± 3	34
2	16.101	39		
3	16.098	44		

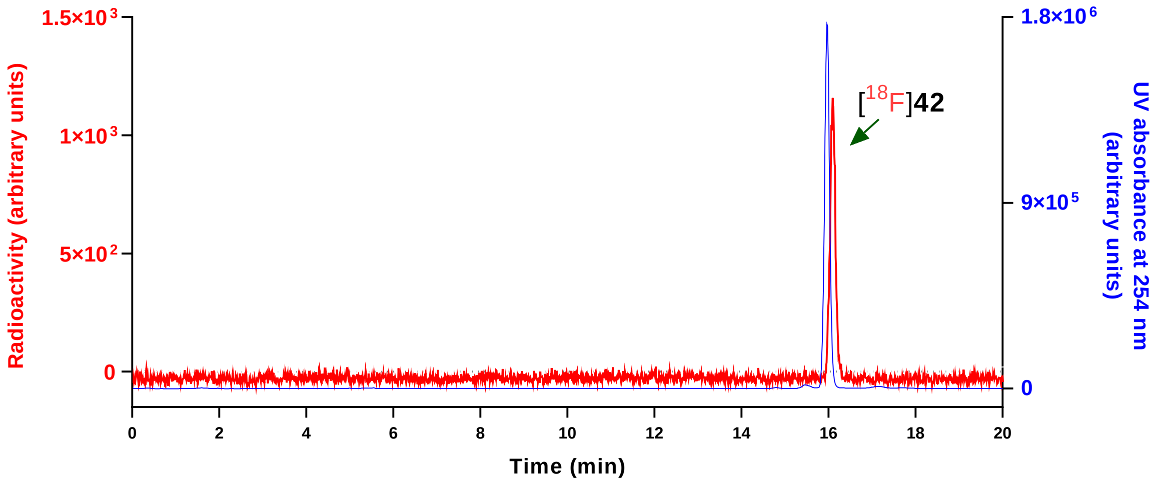

Supplementary Figure 120. Analytical HPLC chromatogram for [^{18}F]**42** with co–injected **42**.

HPLC analysis of [^{18}F]43

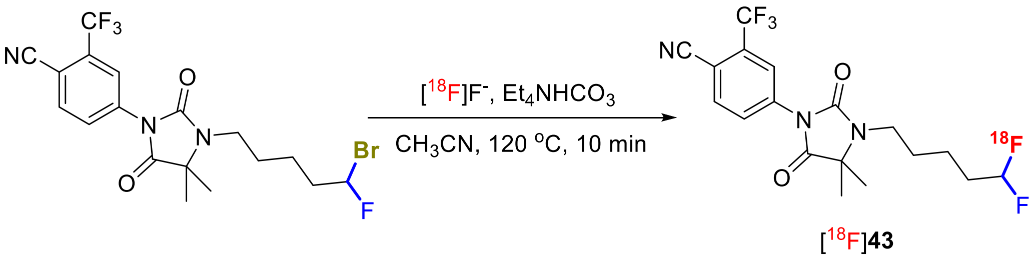

Prepared following general procedure 8 and analyzed using HPLC condition A.

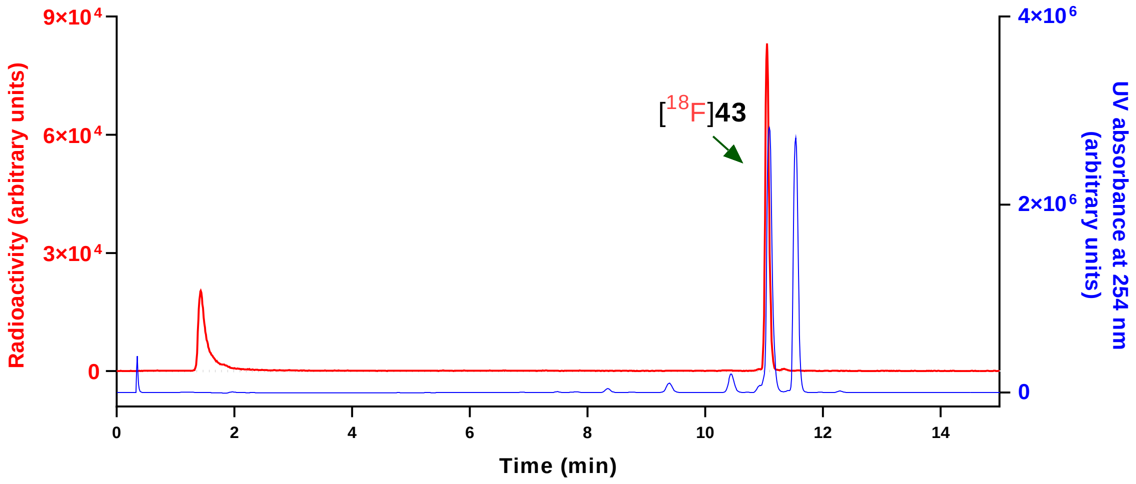

Supplementary Figure 121. Analytical HPLC chromatogram for reaction mixture of [^{18}F]**43**.

Supplementary Table 53. Radiosynthesis of [^{18}F]**43**.

Run	Retention time (min)	Yield (decay-corrected) (%)	Mean yield ± SD (%)	Isolated yield (%)
1	11.051	63	67 ± 4	61
2	11.098	70		
3	11.125	67		

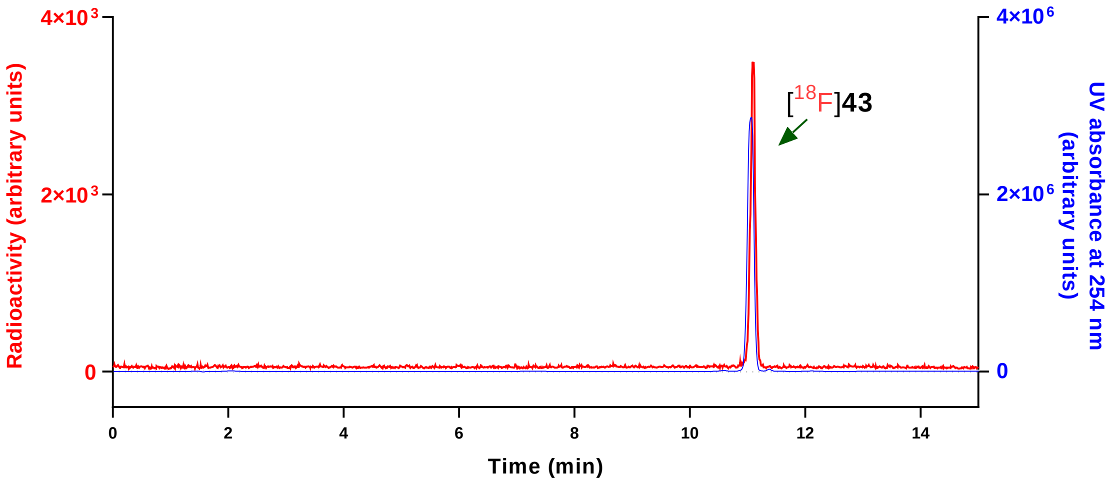

Supplementary Figure 122. Analytical HPLC chromatogram for [^{18}F]**43** with co–injected **43**.

HPLC analysis of [^{18}F]44

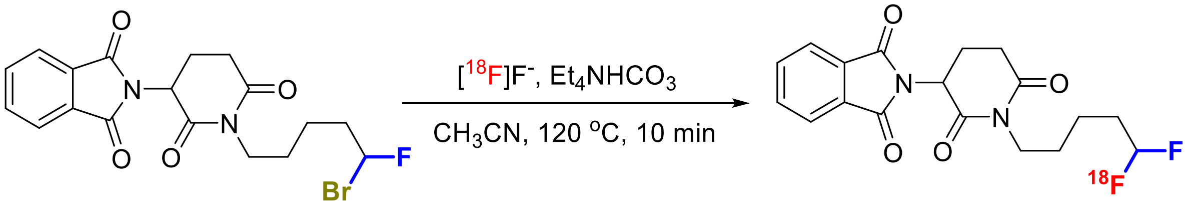

[^{18}F]**44**

Prepared following general procedure 8 and analyzed using HPLC condition A.

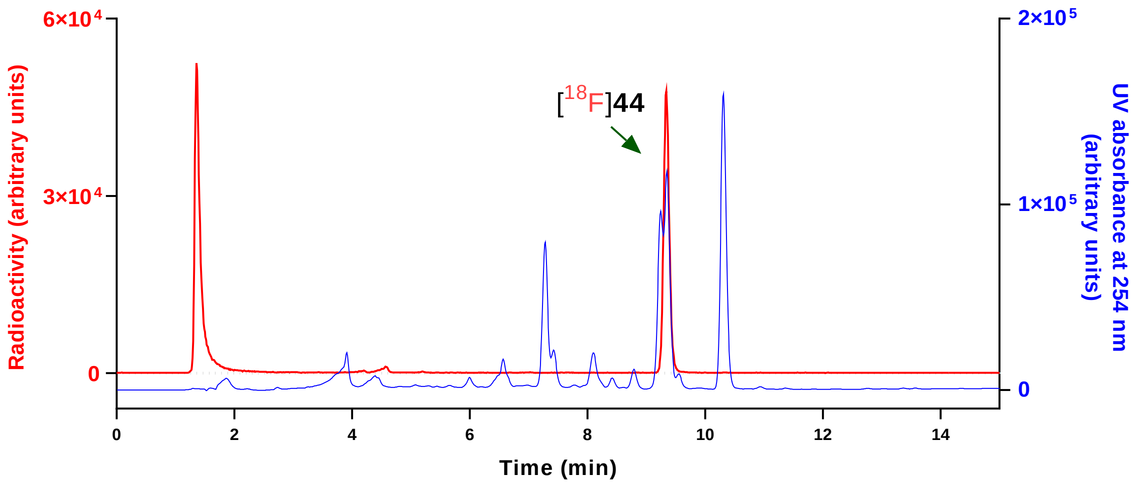

Supplementary Figure 123. Analytical HPLC chromatogram for reaction mixture of [^{18}F]**44**.

Supplementary Table 54. Radiosynthesis of [^{18}F]**44**.

Run	Retention time (min)	Yield (decay-corrected) (%)	Mean yield ± SD (%)	Isolated yield (%)
1	9.500	43	46 ± 3	44
2	9.340	45		
3	9.505	49		

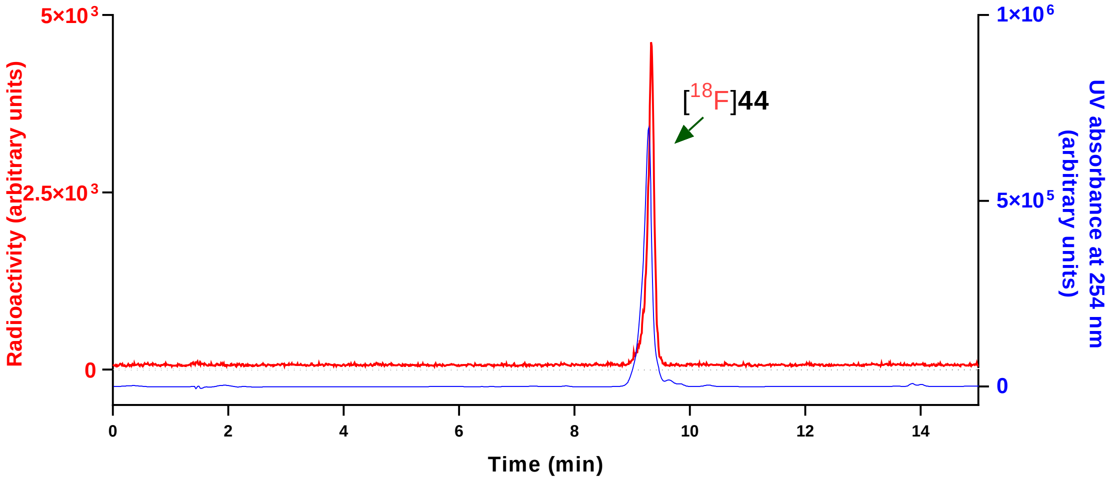

Supplementary Figure 124. Analytical HPLC chromatogram for [^{18}F]**44** with co–injected **44**.

HPLC analysis of [^{18}F]45

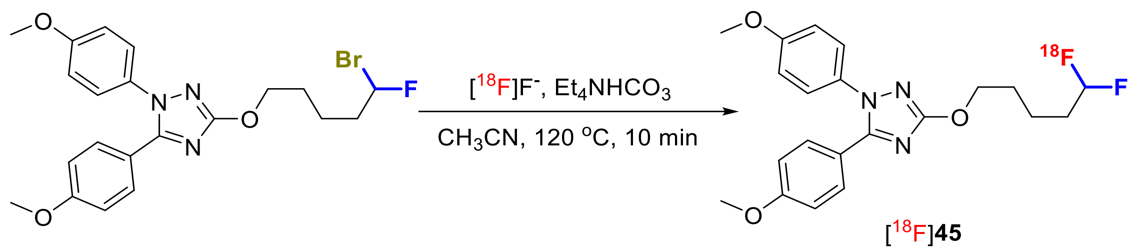

Prepared following general procedure 8 and analyzed using HPLC condition A.

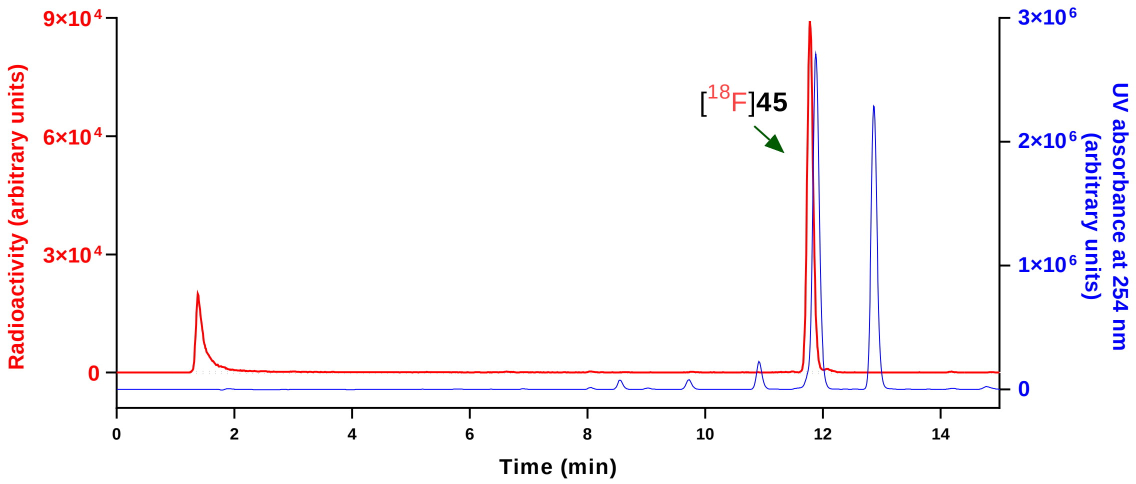

Supplementary Figure 125. Analytical HPLC chromatogram for reaction mixture of [^{18}F]**45**.

Supplementary Table 55. Radiosynthesis of [^{18}F]**45**.

Run	Retention time (min)	Yield (decay-corrected) (%)	Mean yield ± SD (%)	Isolated yield (%)
1	11.775	75	76 ± 1	68
2	11.783	76		
3	11.781	77		

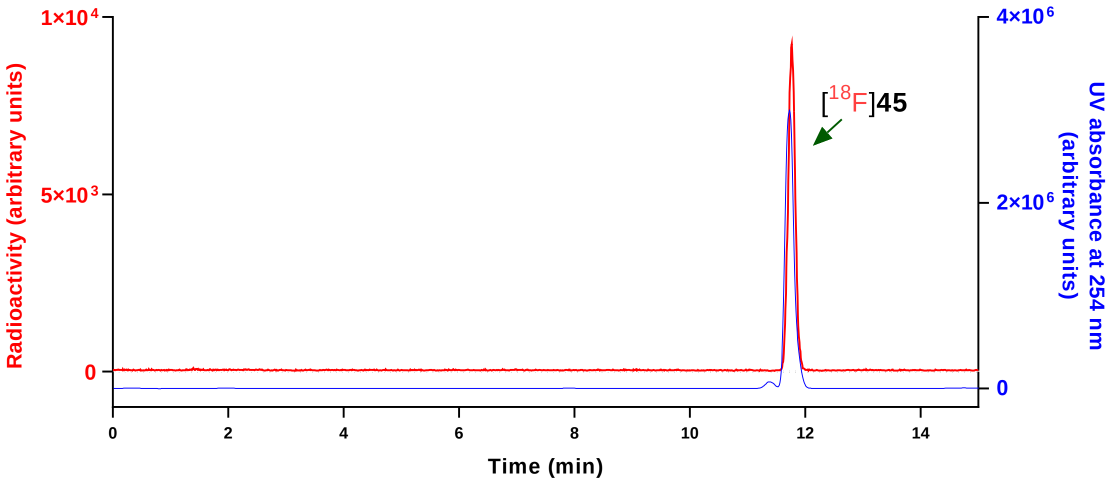

Supplementary Figure 126. Analytical HPLC chromatogram for [^{18}F]**45** with co–injected **45**.

HPLC analysis of [^{18}F]46

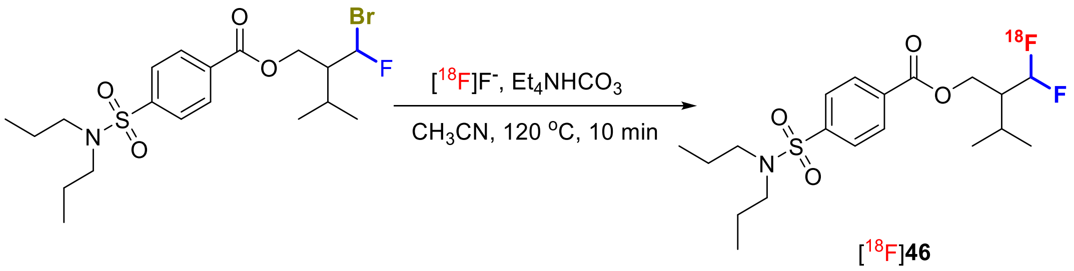

Prepared following general procedure 8 and analyzed using HPLC condition A.

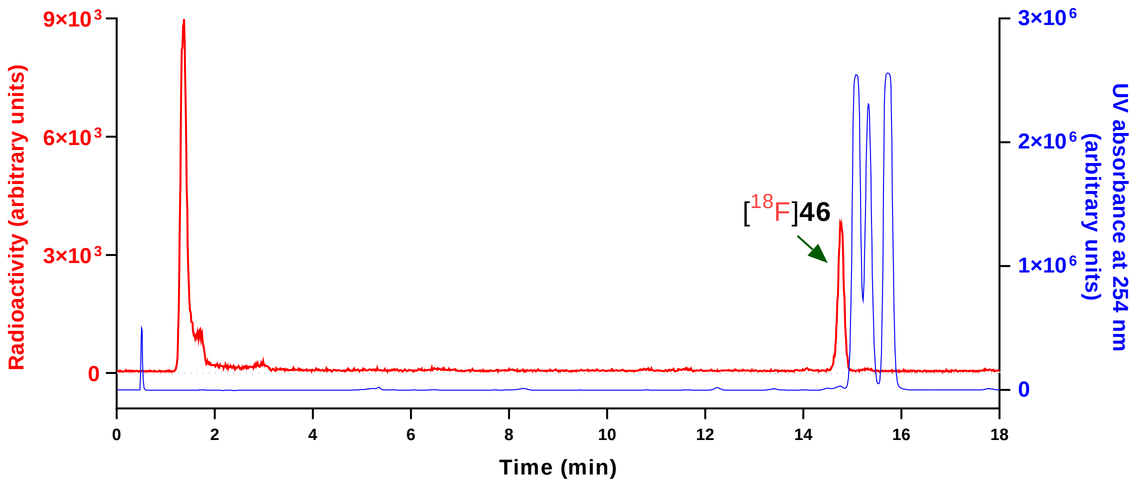

Supplementary Figure 127. Analytical HPLC chromatogram for reaction mixture of [^{18}F]**46**.

Supplementary Table 56. Radiosynthesis of [^{18}F]**46**.

Run	Retention time (min)	Yield (decay-corrected) (%)	Mean yield ± SD (%)	Isolated yield (%)
1	14.761	24	26 ± 5	23
2	14.892	31		
3	14.890	22		

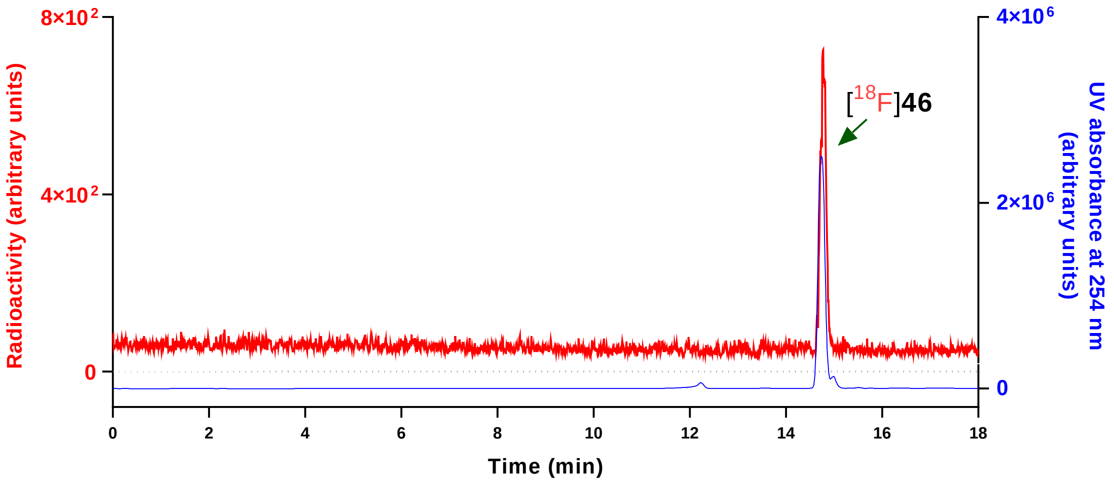

Supplementary Figure 128. Analytical HPLC chromatogram for [^{18}F]**46** with co–injected **46**.

HPLC analysis of [^{18}F]47

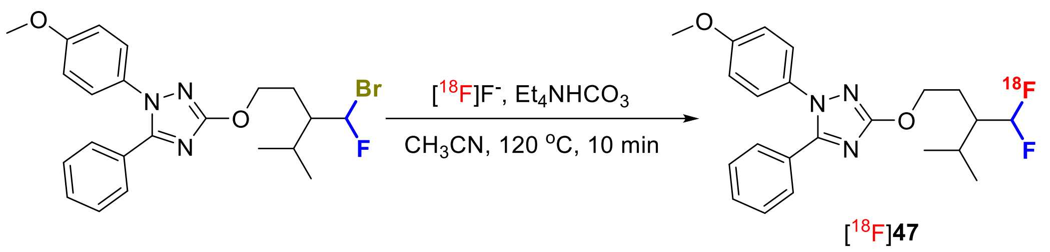

Prepared following general procedure 8 and analyzed using HPLC condition A.

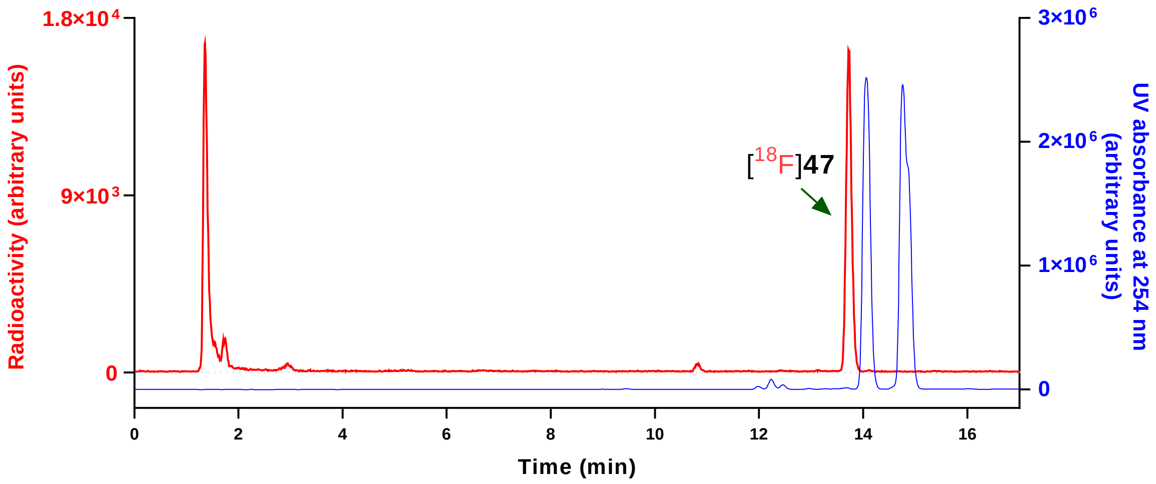

Supplementary Figure 129. Analytical HPLC chromatogram for reaction mixture of [^{18}F]**47**.

**Supplementary Table 57.** Radiosynthesis of [^{18}F]**47**				
Run	Retention time (min)	Yield (decay-corrected) (%)	Mean yield ± SD (%)	Isolated yield (%)
1	13.720	49	48 ± 3	45
2	13.736	44		
3	13.814	50		

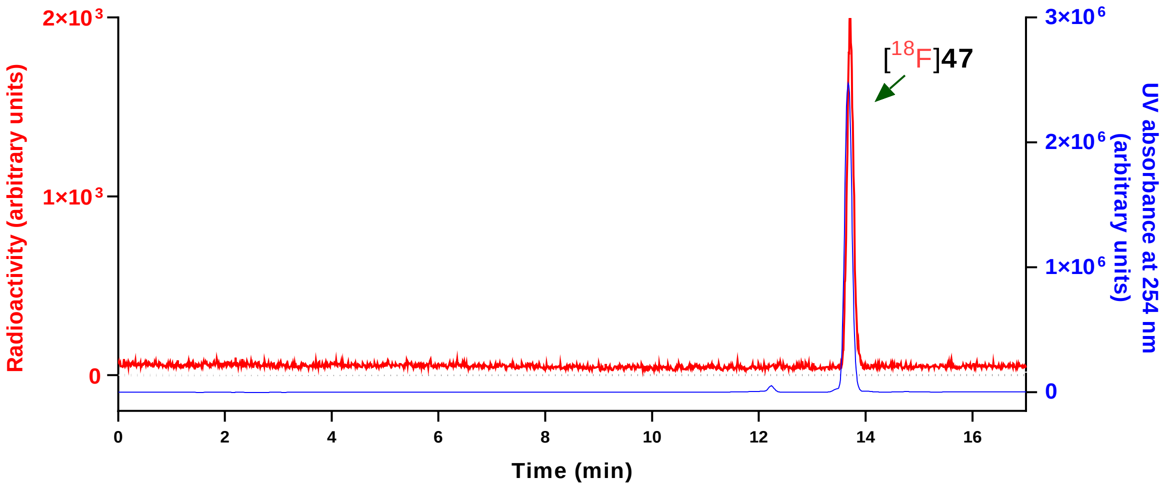

Supplementary Figure 130. Analytical HPLC chromatogram for [^{18}F]**47** with co–injected **47**.

5. NMR spectra of precursors and standards 1–47

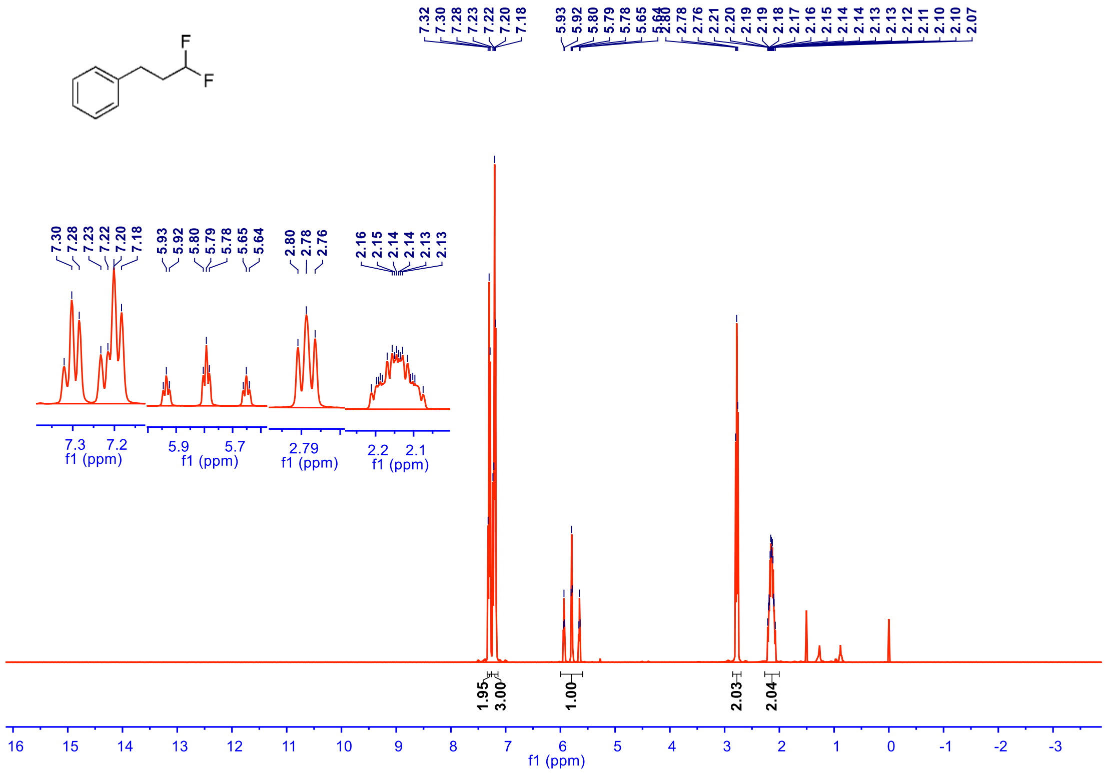

Supplementary Figure 131. ^{1}H NMR spectrum of compound **1** (400 MHz, $CDCl_3$)

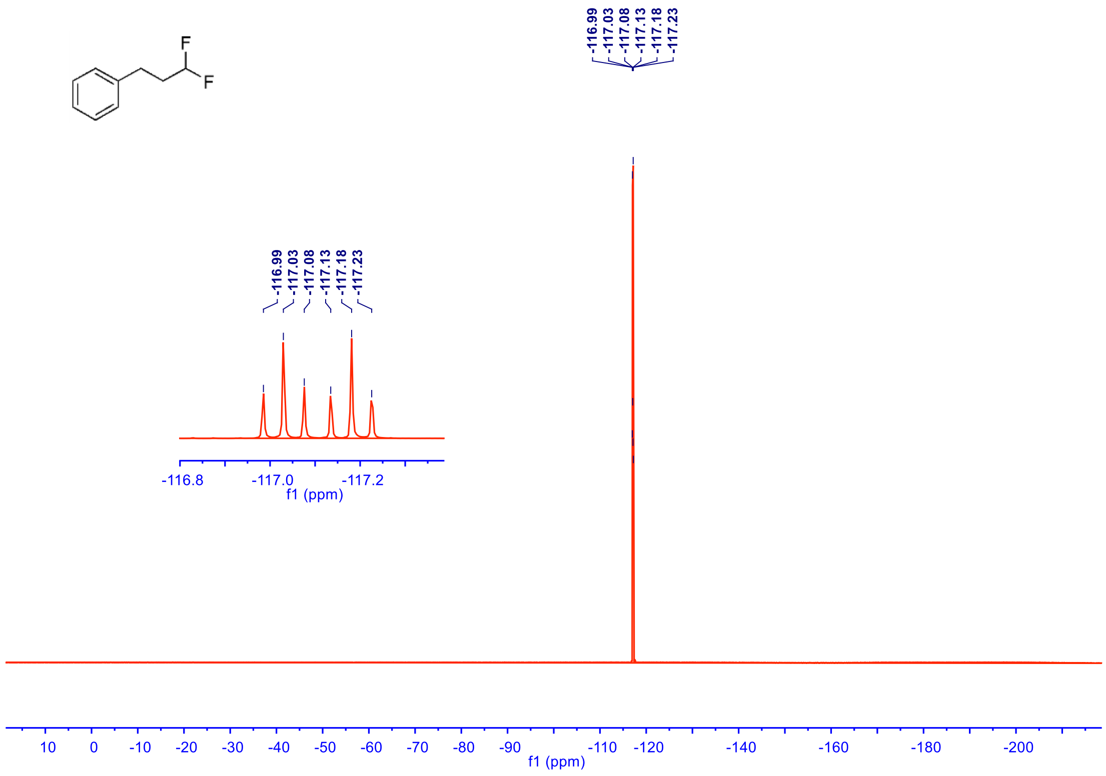

Supplementary Figure 132. ^{19}F NMR spectrum of compound **1** (376 MHz, $CDCl_3$)

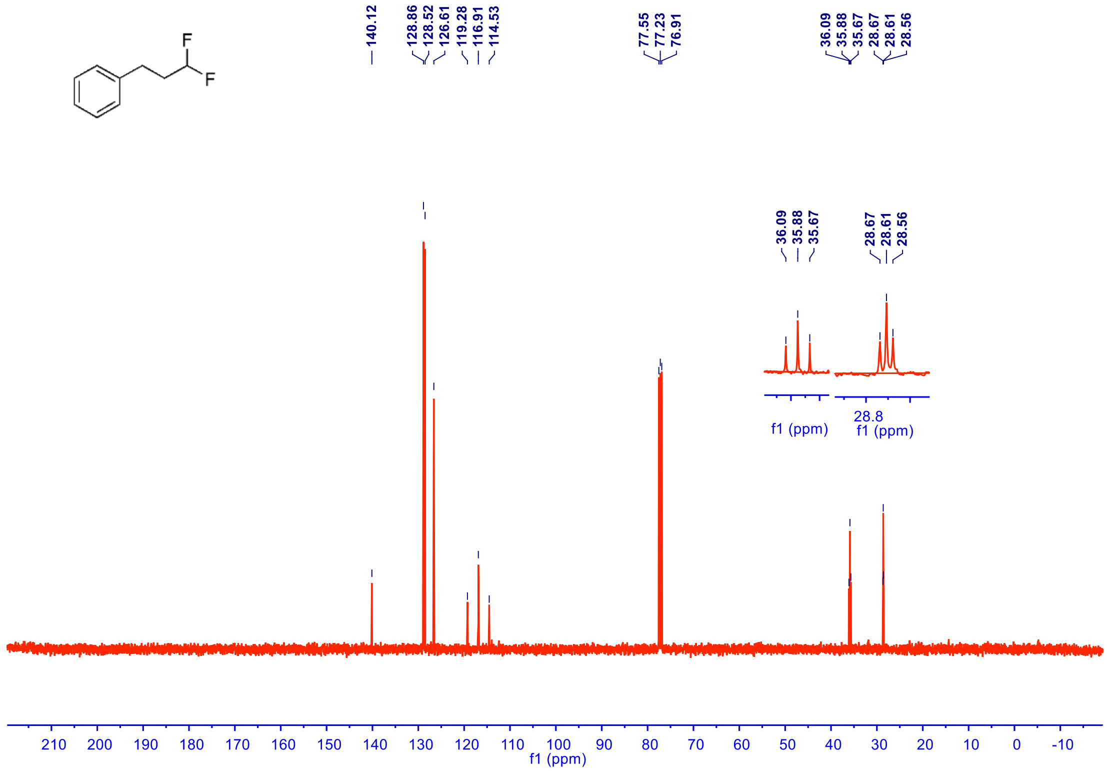

Supplementary Figure 133. ^{13}C NMR spectrum of compound **1** (101 MHz, $CDCl_3$)

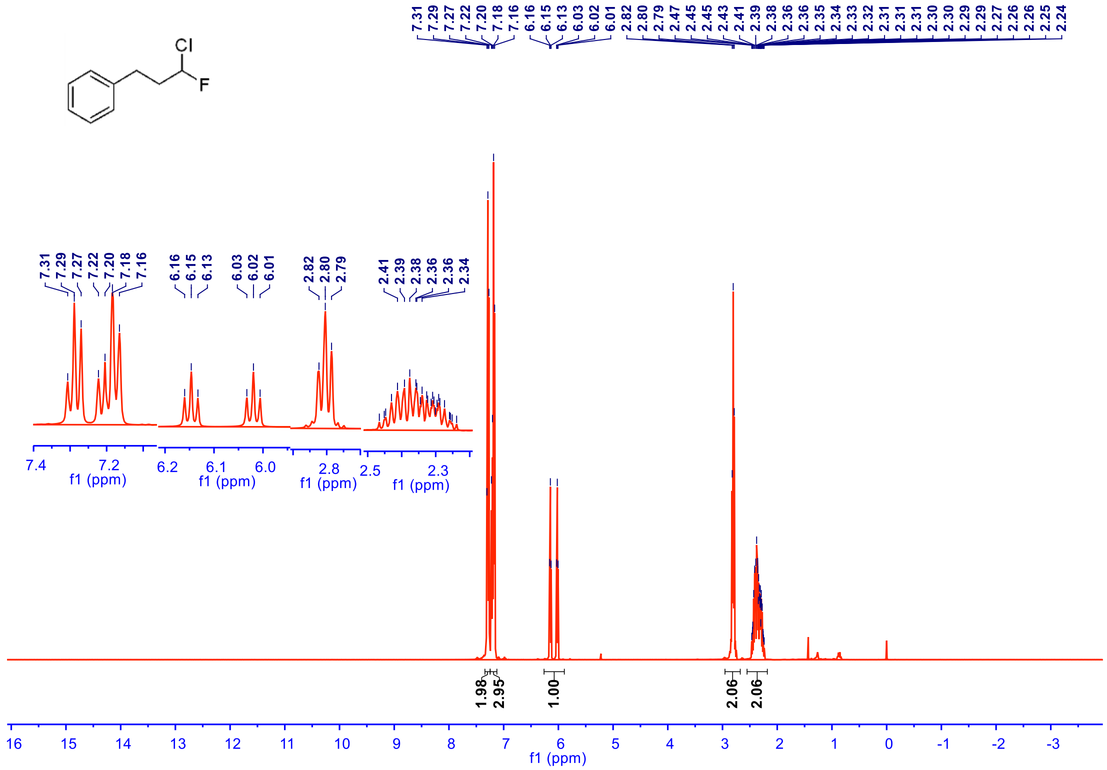

Supplementary Figure 134. ^{1}H NMR spectrum of compound **2** (400 MHz, $CDCl_3$)

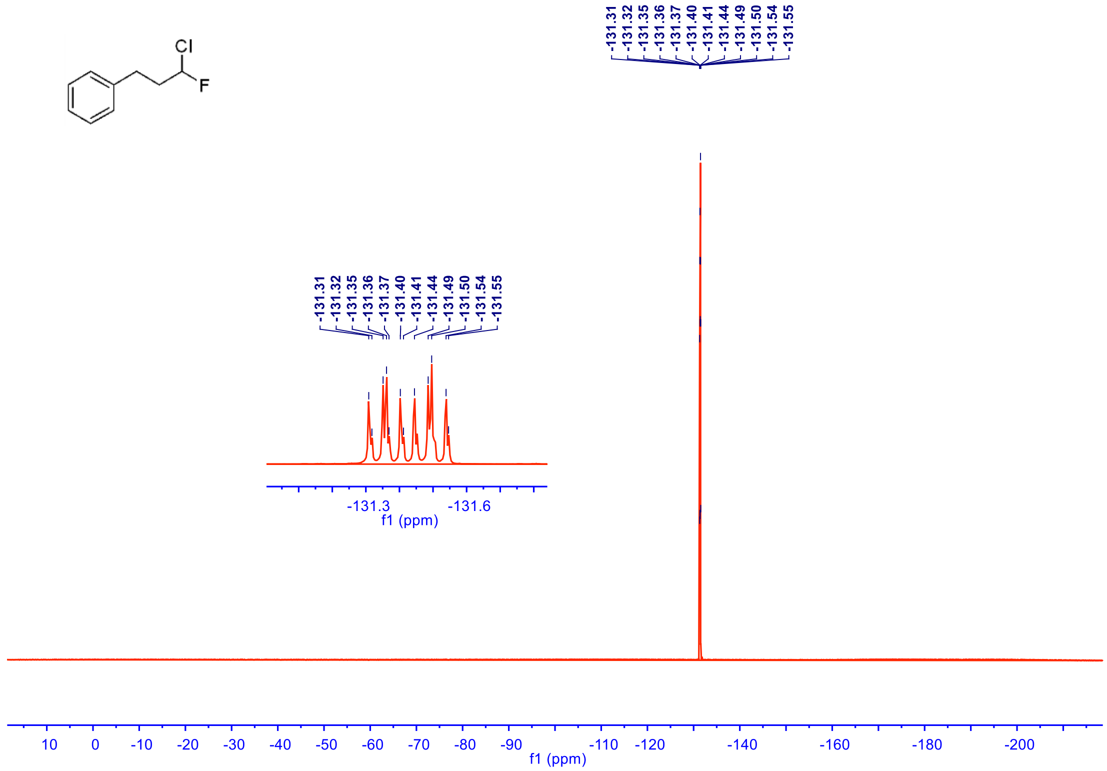

Supplementary Figure 135. ^{19}F NMR spectrum of compound **2** (376 MHz, $CDCl_3$)

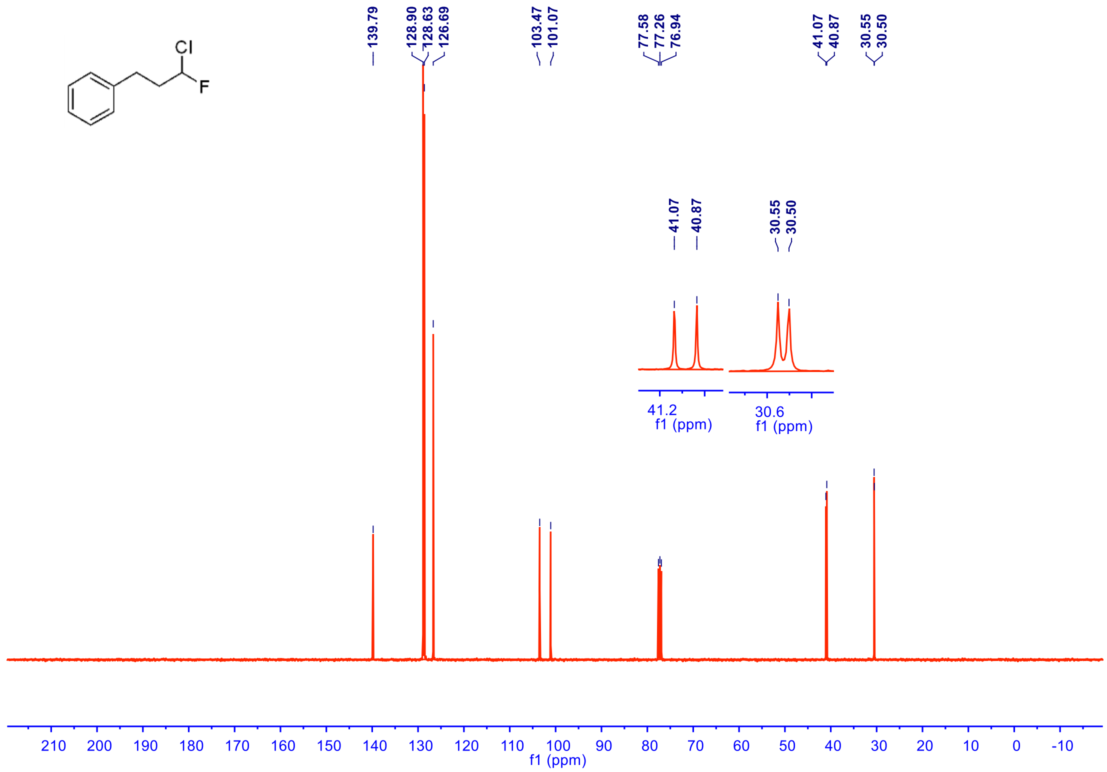

Supplementary Figure 136. ^{13}C NMR spectrum of compound **2** (101 MHz, $CDCl_3$)

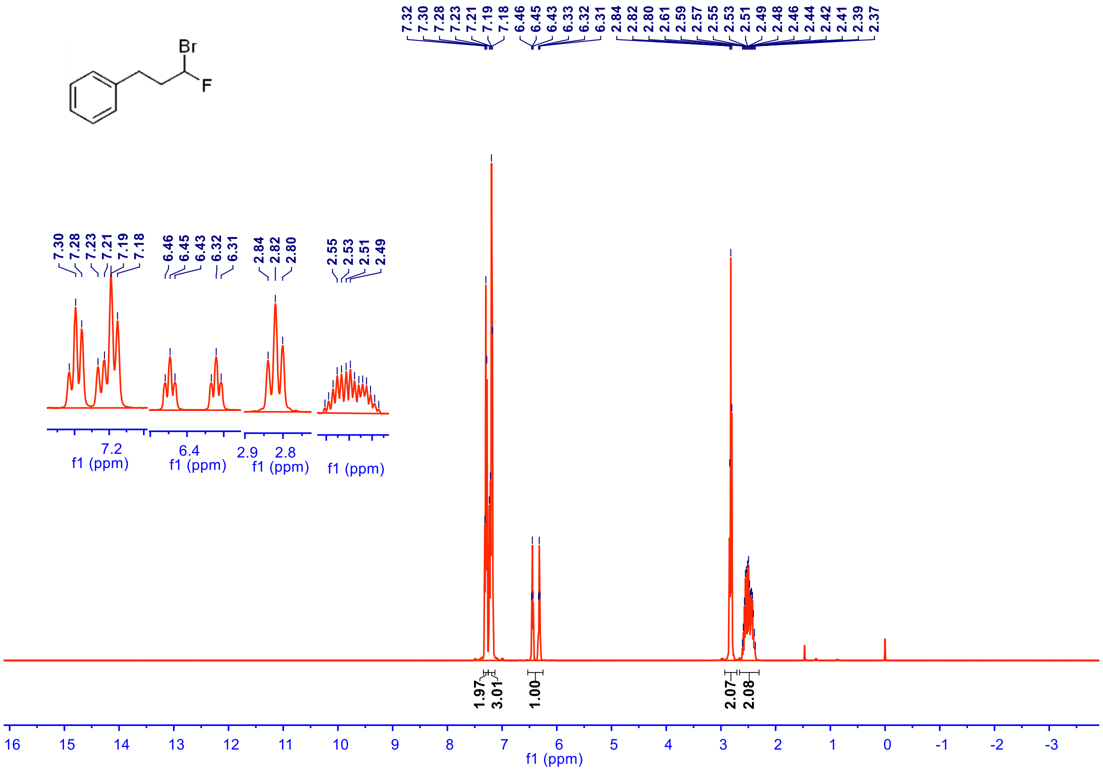

Supplementary Figure 137. ^{1}H NMR spectrum of compound **3** (400 MHz, $CDCl_3$)

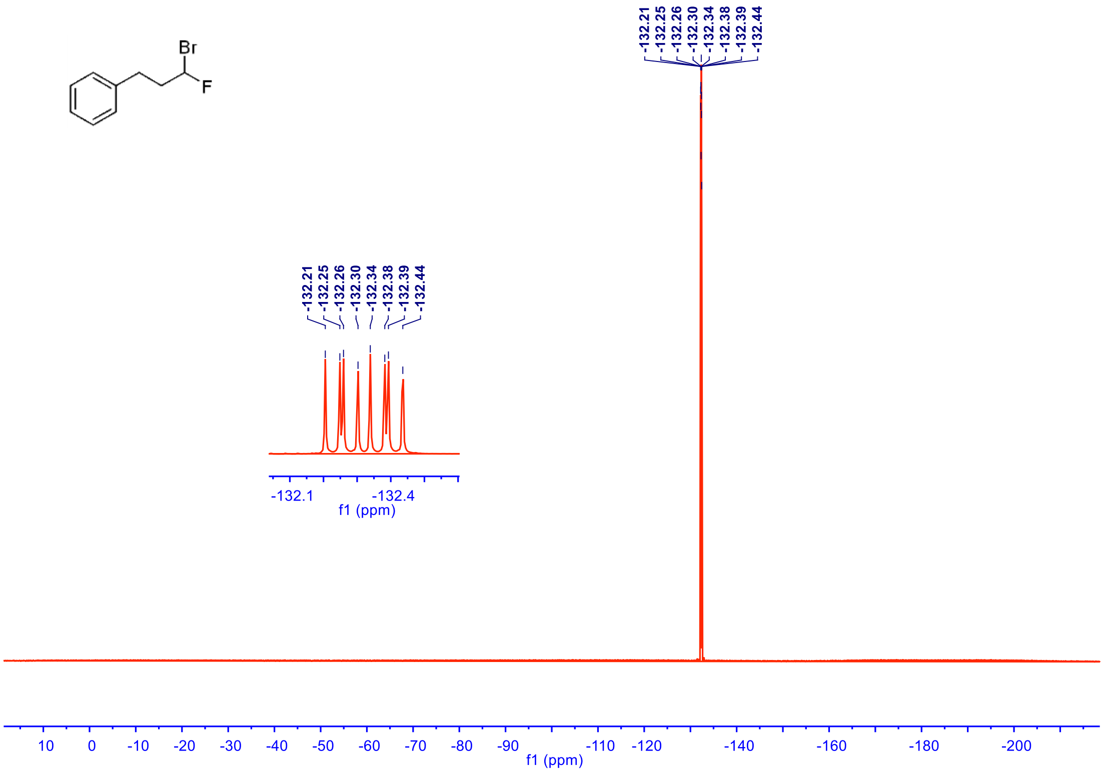

Supplementary Figure 138. ^{19}F NMR spectrum of compound **3** (376 MHz, $CDCl_3$)

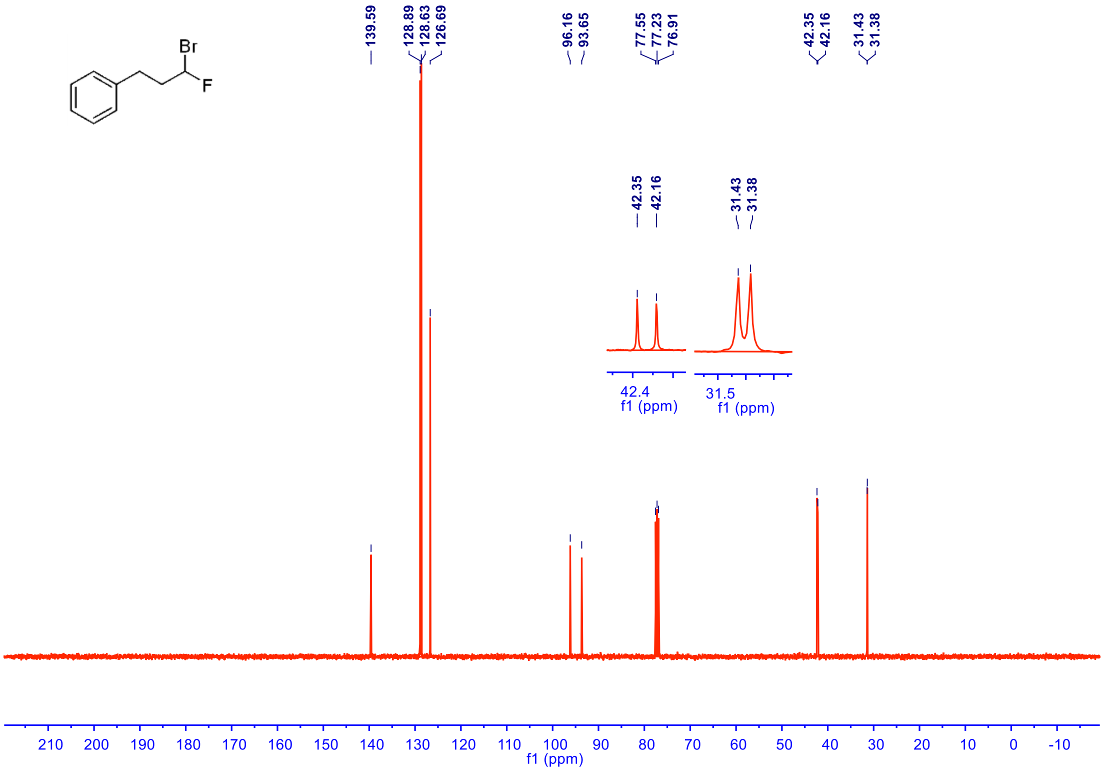

Supplementary Figure 139. ^{13}C NMR spectrum of compound **3** (101 MHz, $CDCl_3$)

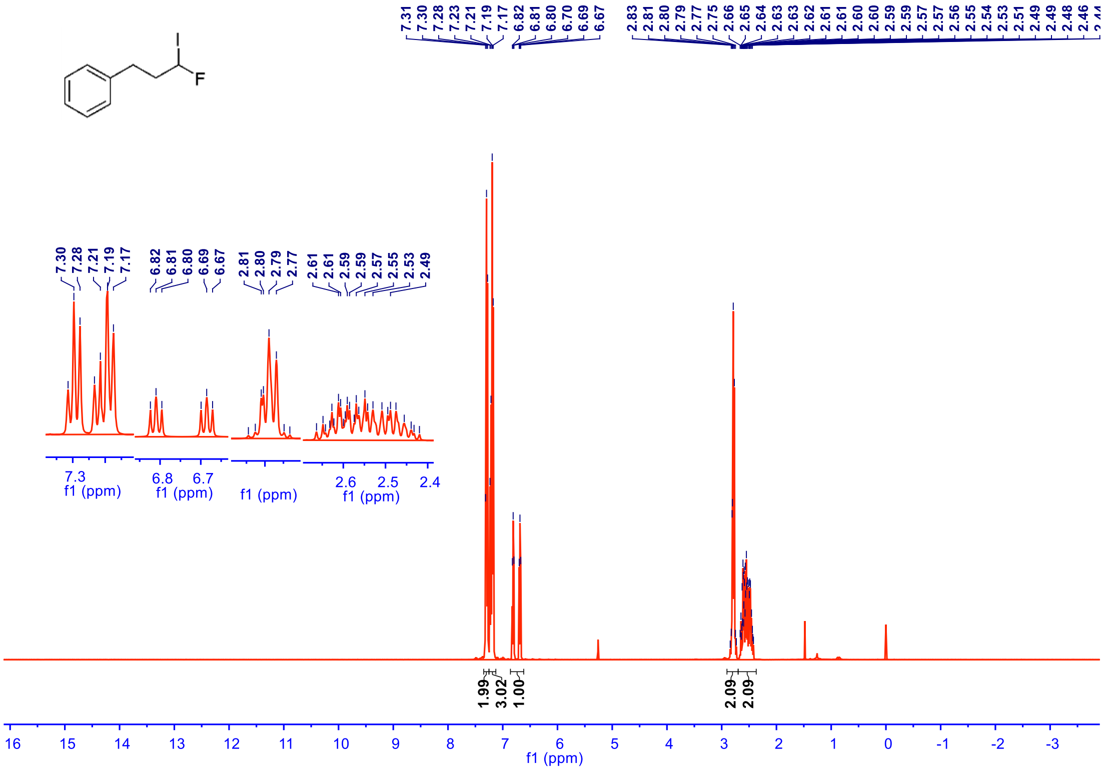

Supplementary Figure 140. ^{1}H NMR spectrum of compound **4** (400 MHz, $CDCl_3$)

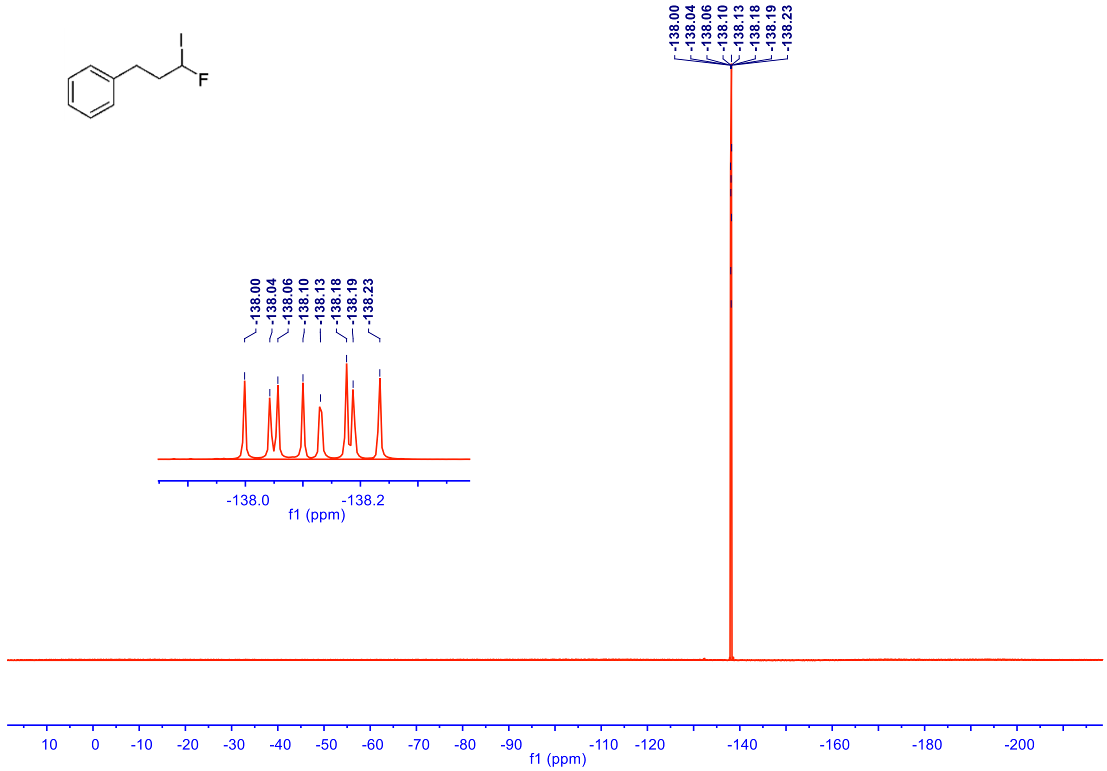

Supplementary Figure 141. ^{19}F NMR spectrum of compound **4** (376 MHz, $CDCl_3$)

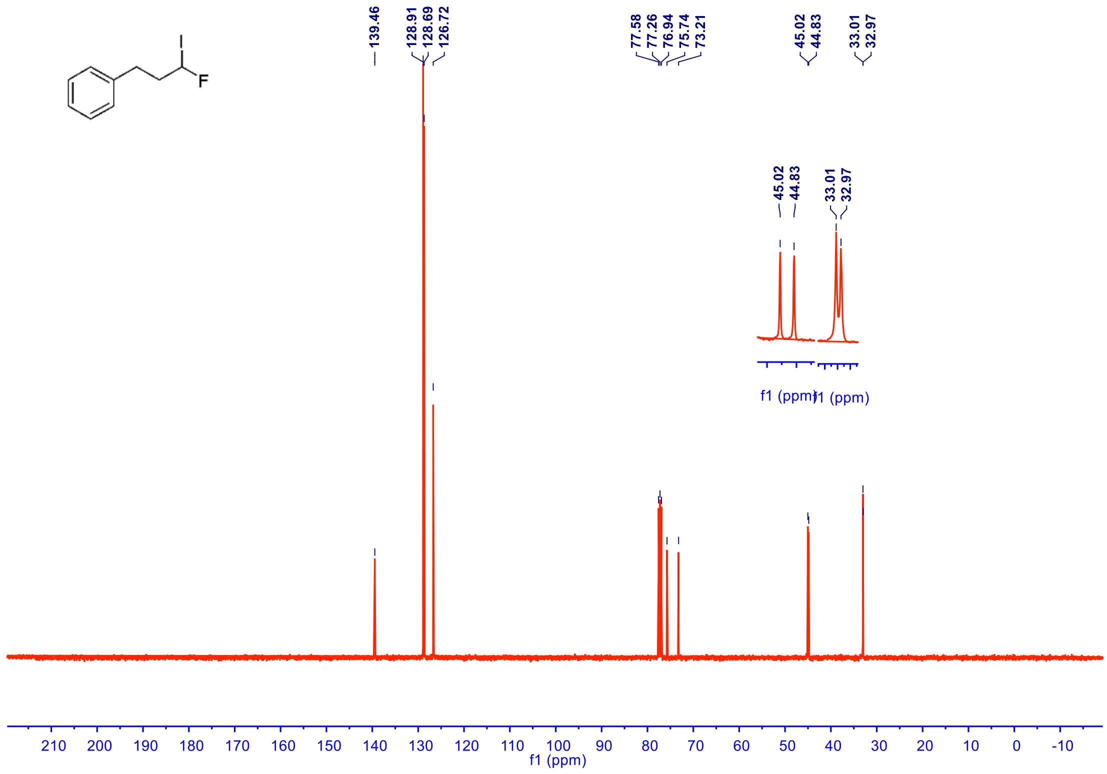

Supplementary Figure 142. ^{13}C NMR spectrum of compound **4** (101 MHz, $CDCl_3$)

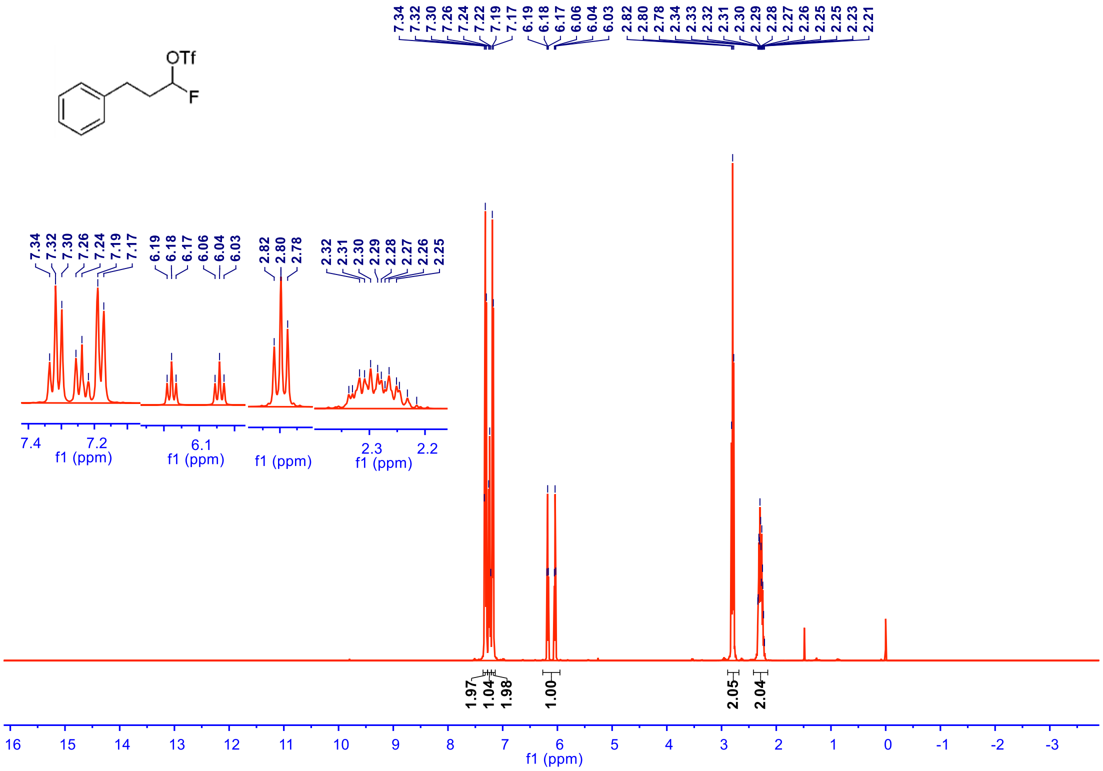

Supplementary Figure 143. ^{1}H NMR spectrum of compound **5** (400 MHz, $CDCl_3$)

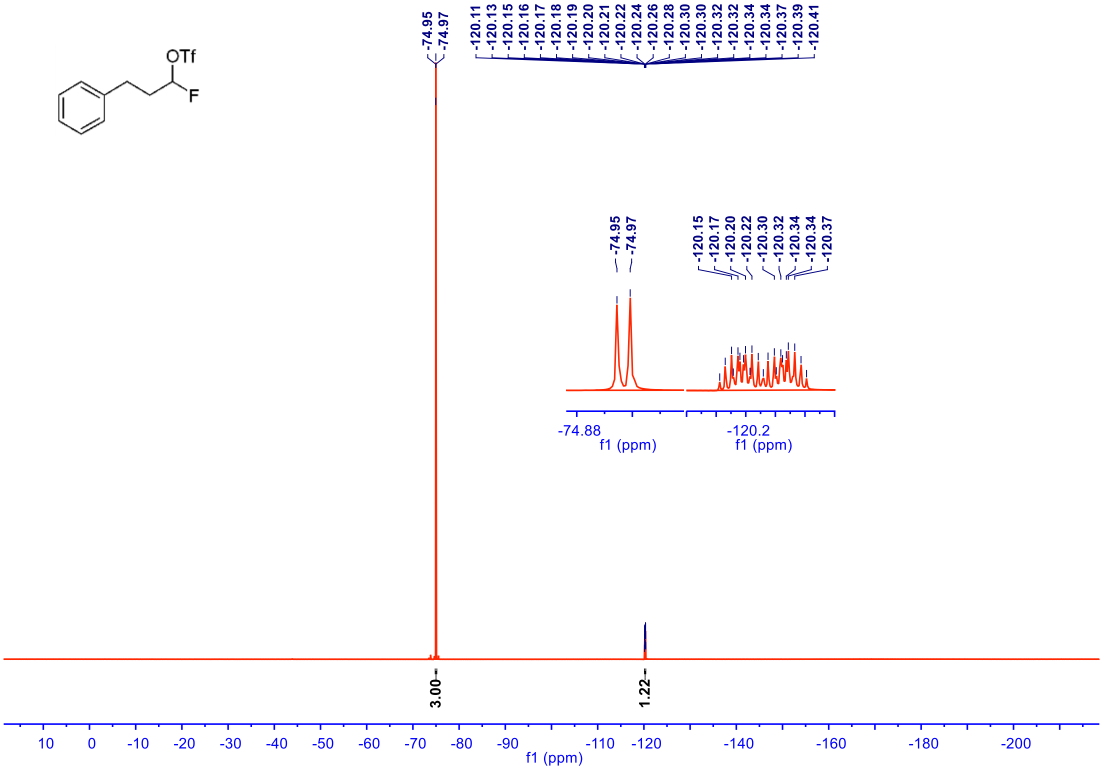

Supplementary Figure 144. ^{19}F NMR spectrum of compound **5** (376 MHz, $CDCl_3$)

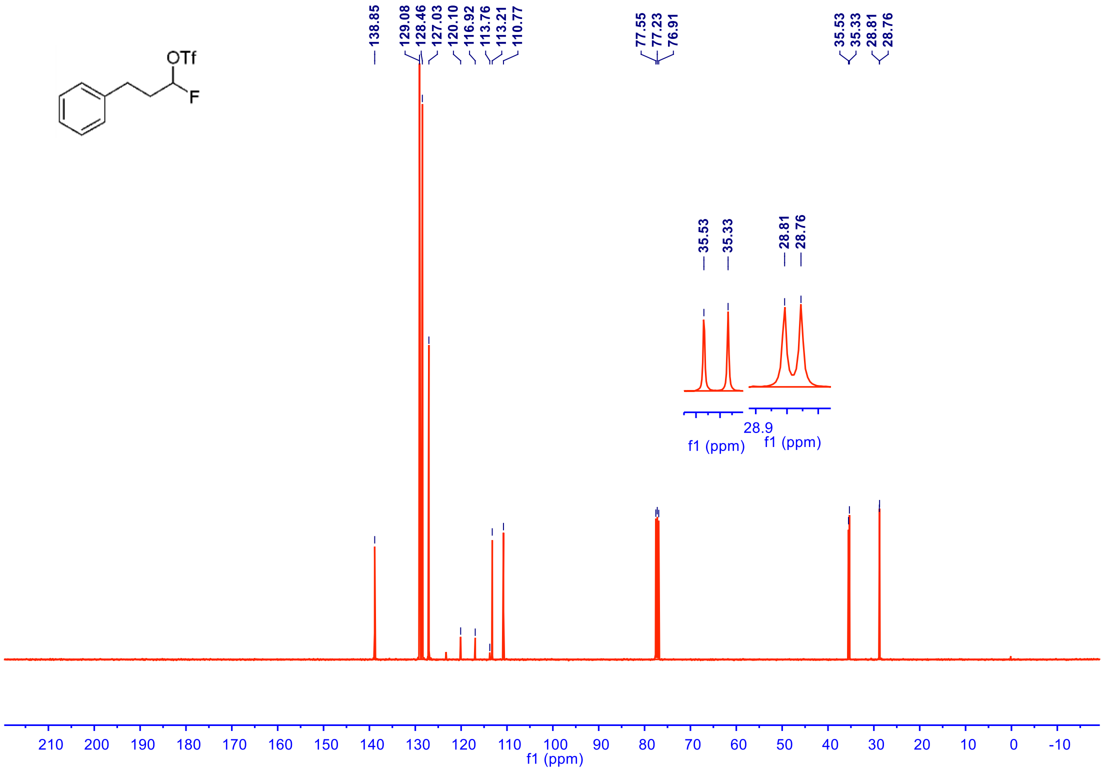

Supplementary Figure 145. ^{13}C NMR spectrum of compound **5** (101 MHz, $CDCl_3$)

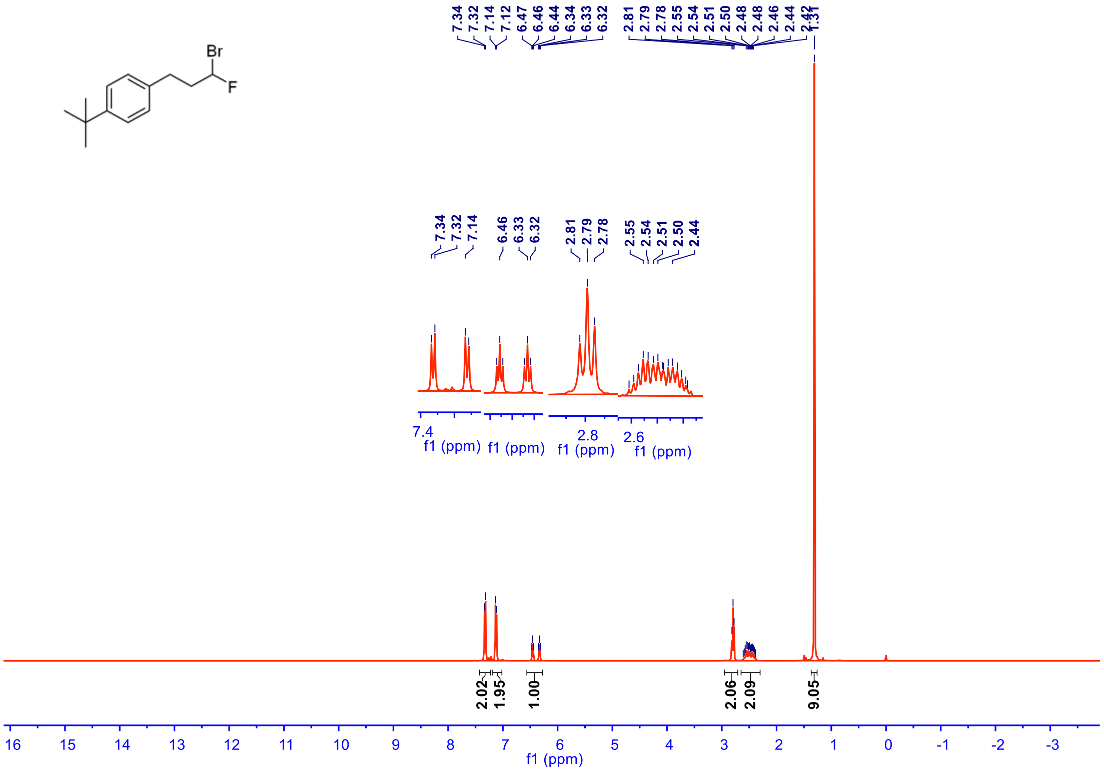

Supplementary Figure 146. ^{1}H NMR spectrum of **1-(3-bromo-3-fluoropropyl)-4-(*tert*-butyl)benzene 6a** (400 MHz, $CDCl_3$)

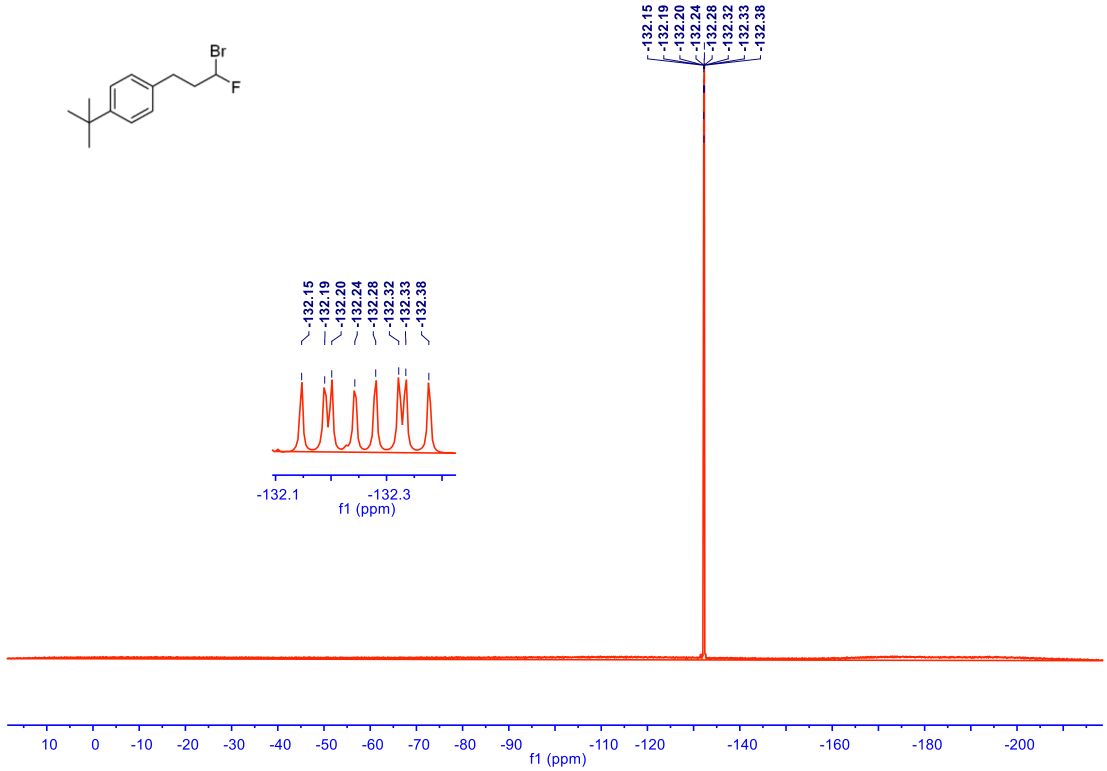

Supplementary Figure 147. ^{19}F NMR spectrum of **1-(3-bromo-3-fluoropropyl)-4-(*tert*-butyl)benzene 6a** (376 MHz, $CDCl_3$)

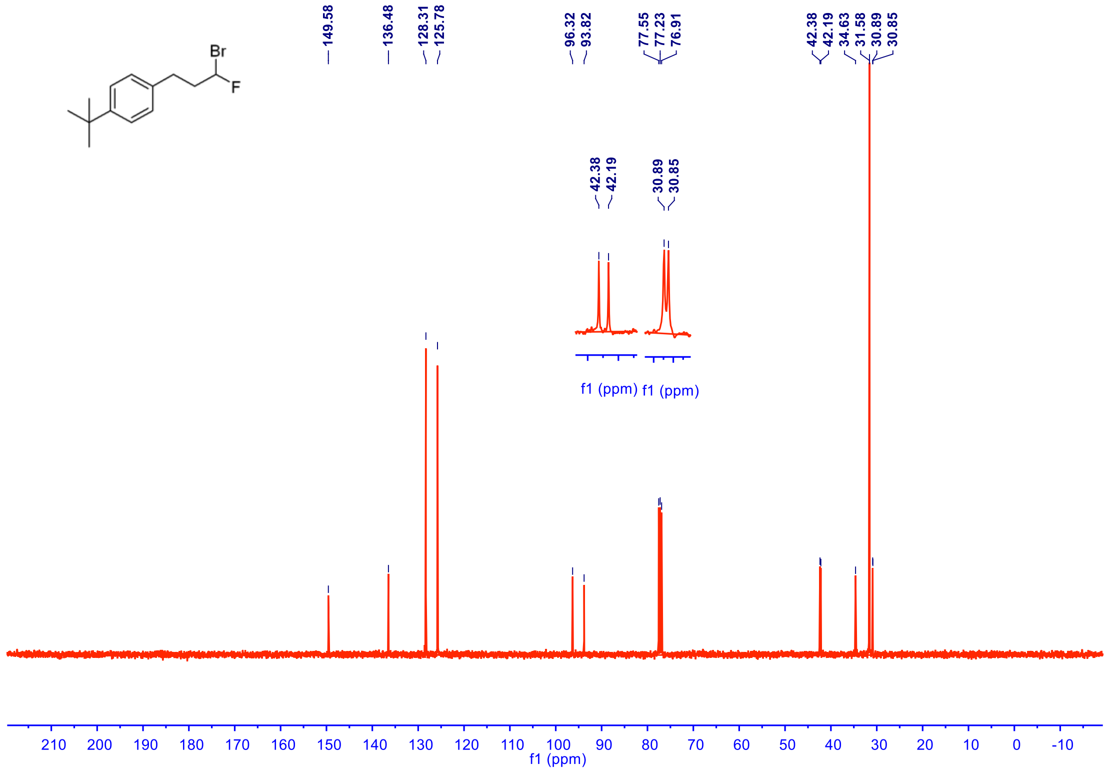

Supplementary Figure 148. ^{13}C NMR spectrum of **1-(3-bromo-3-fluoropropyl)-4-(*tert*-butyl)benzene 6a** (101 MHz, $CDCl_3$)

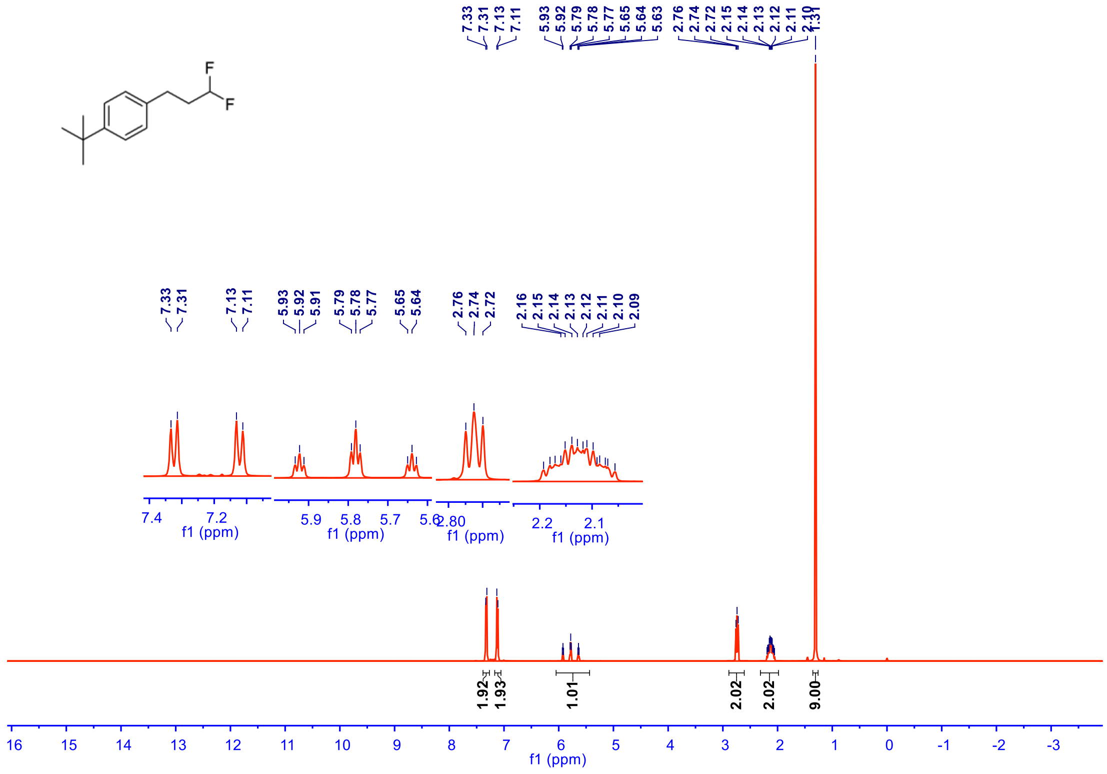

Supplementary Figure 149. ^{1}H NMR spectrum of compound **6** (400 MHz, $CDCl_3$)

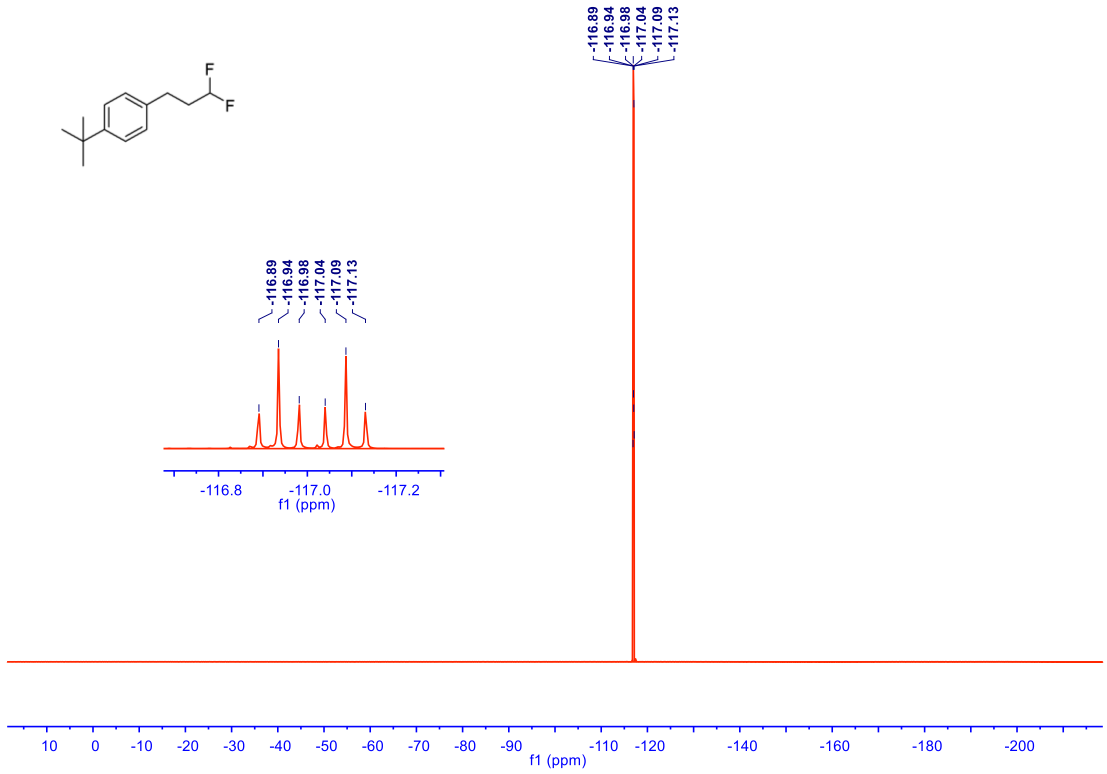

Supplementary Figure 150. ^{19}F NMR spectrum of compound **6** (376 MHz, $CDCl_3$)

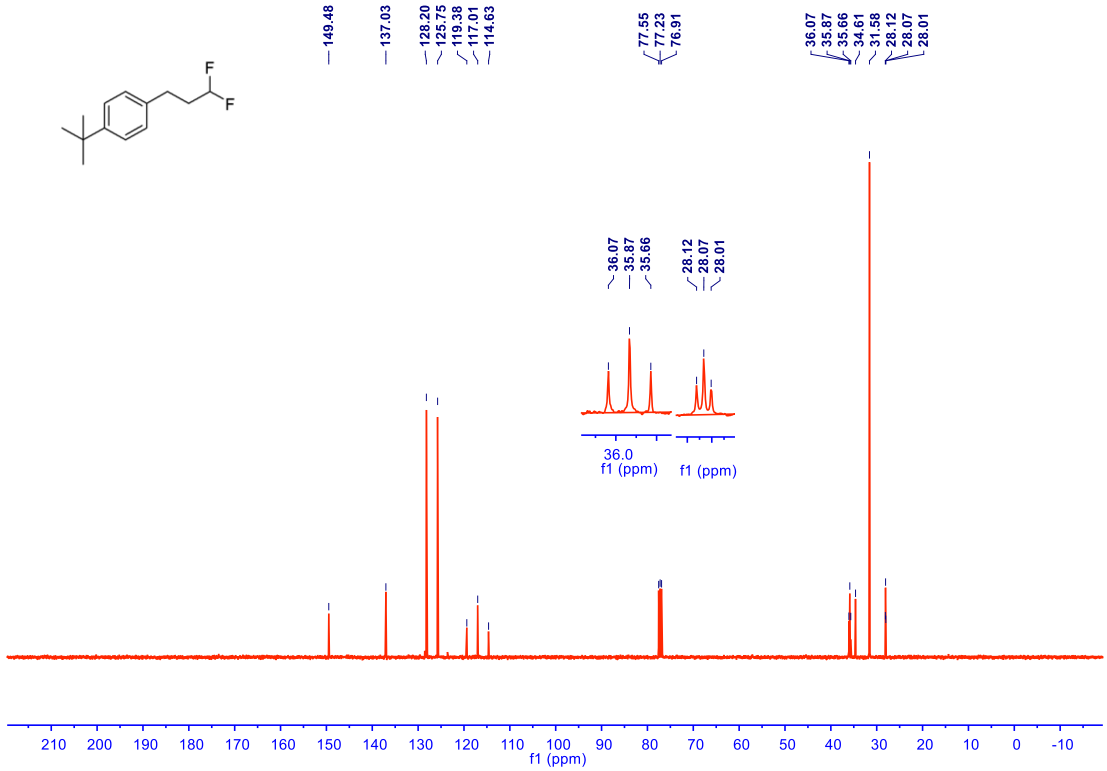

Supplementary Figure 151. ^{13}C NMR spectrum of compound **6** (101 MHz, $CDCl_3$)

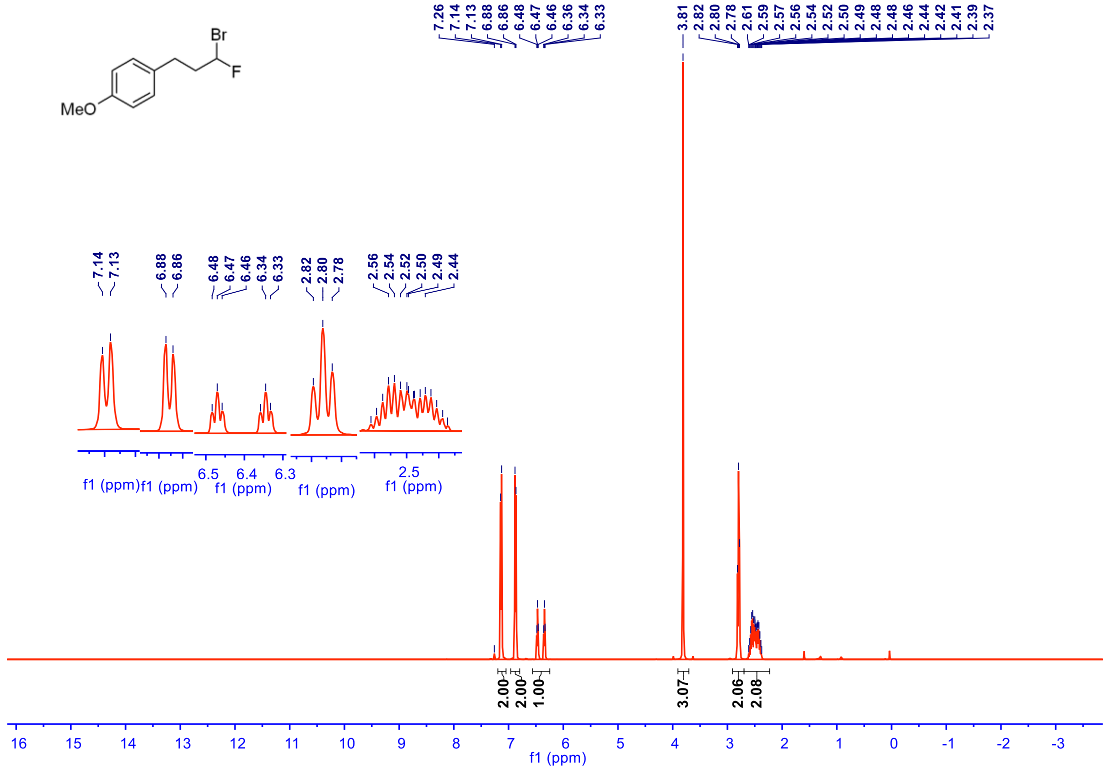

Supplementary Figure 152. ^1H NMR spectrum of **1-(3-bromo-3-fluoropropyl)-4-methoxybenzene 7a** (400 MHz, $CDCl_3$)

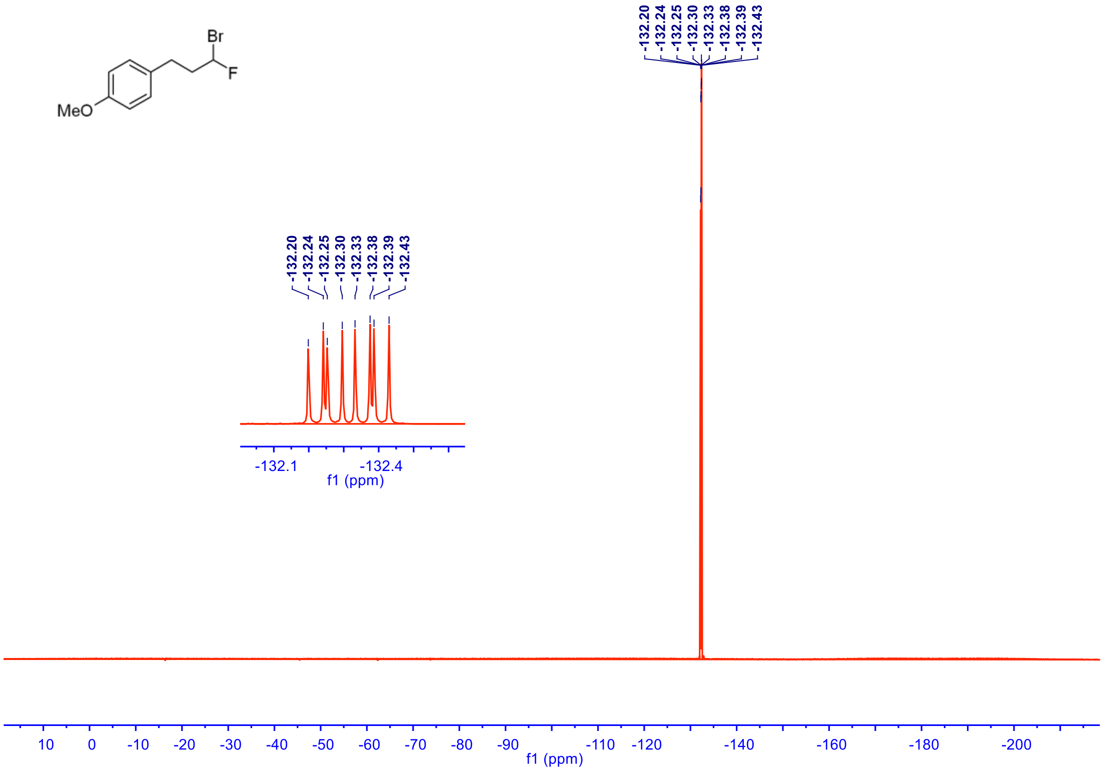

Supplementary Figure 153. ^{19}F NMR spectrum of **1-(3-bromo-3-fluoropropyl)-4-methoxybenzene 7a** (376 MHz, $CDCl_3$)

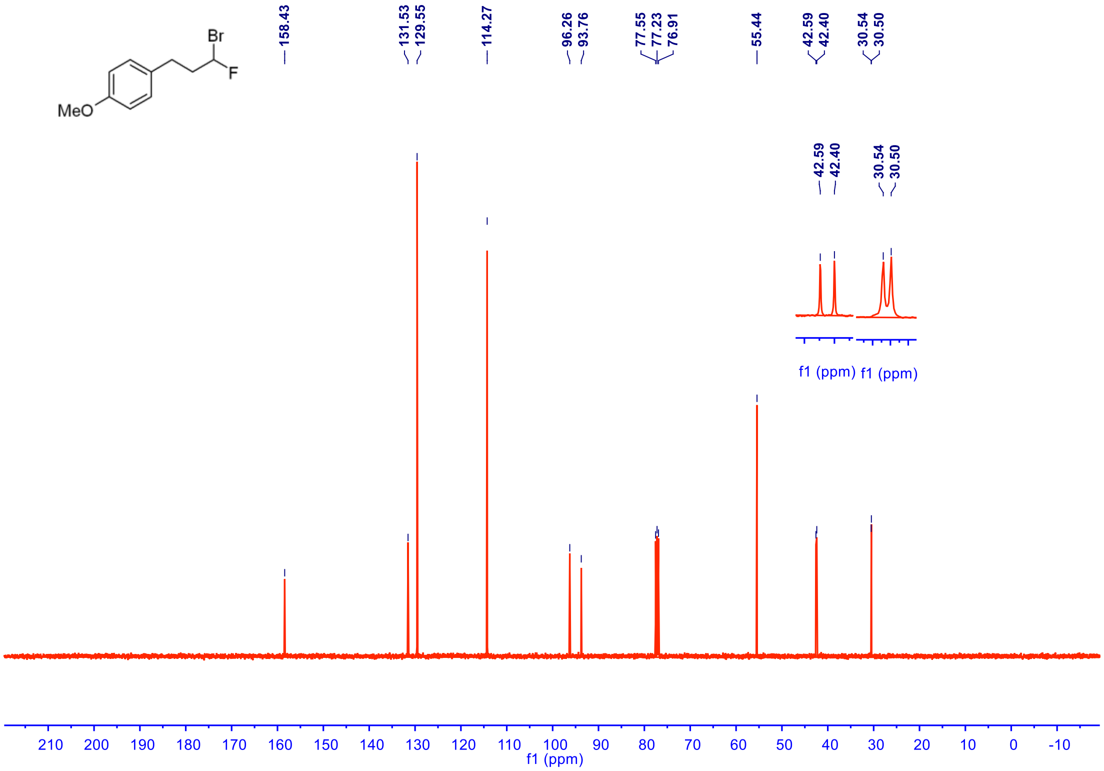

Supplementary Figure 154. ^{13}C NMR spectrum of **1-(3-bromo-3-fluoropropyl)-4-methoxybenzene 7a** (101 MHz, $CDCl_3$)

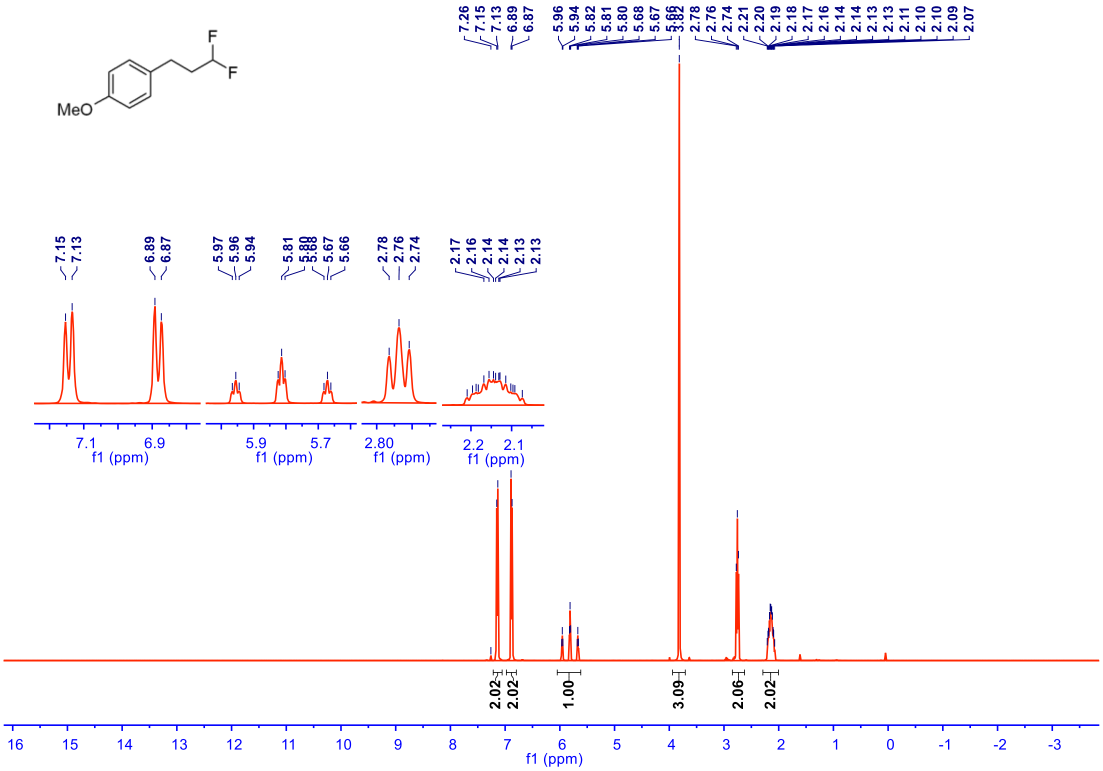

Supplementary Figure 155. ^{1}H NMR spectrum of compound **7** (400 MHz, $CDCl_3$)

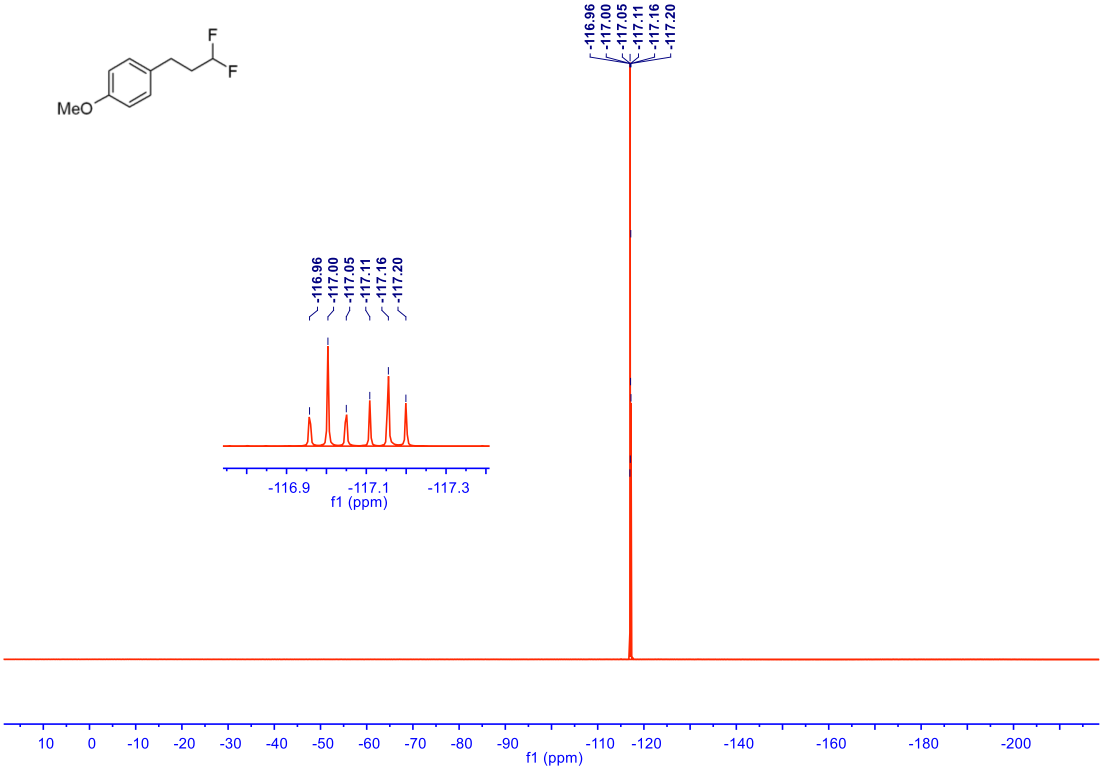

Supplementary Figure 156. ^{19}F NMR spectrum of compound **7** (376 MHz, $CDCl_3$)

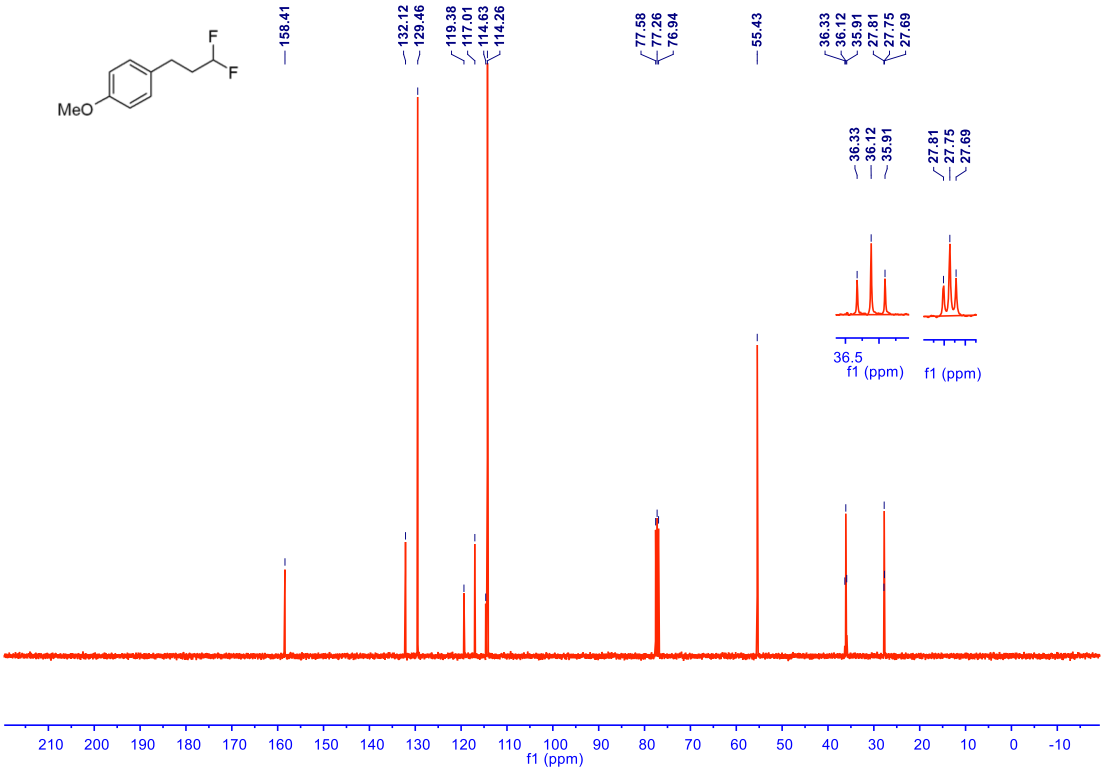

Supplementary Figure 157. ^{13}C NMR spectrum of compound **7** (101 MHz, $CDCl_3$)

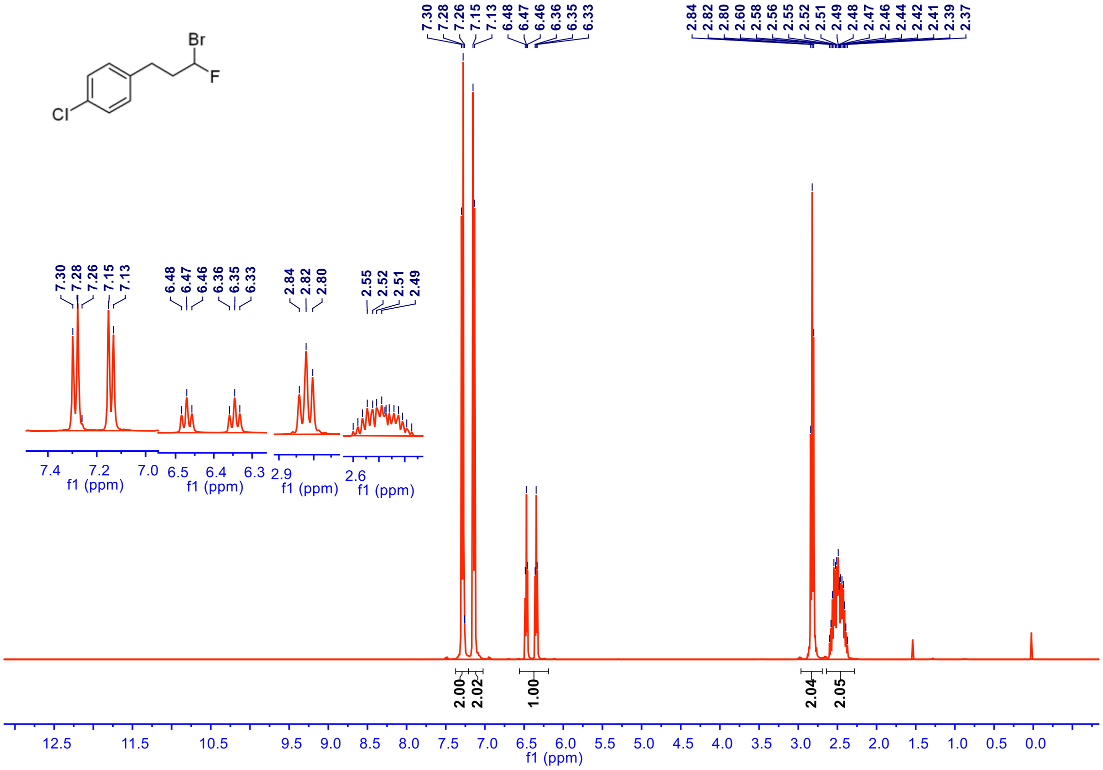

Supplementary Figure 158. ^{1}H NMR spectrum of **1-(3-bromo-3-fluoropropyl)-4-chlorobenzene 8a** (400 MHz, $CDCl_3$)

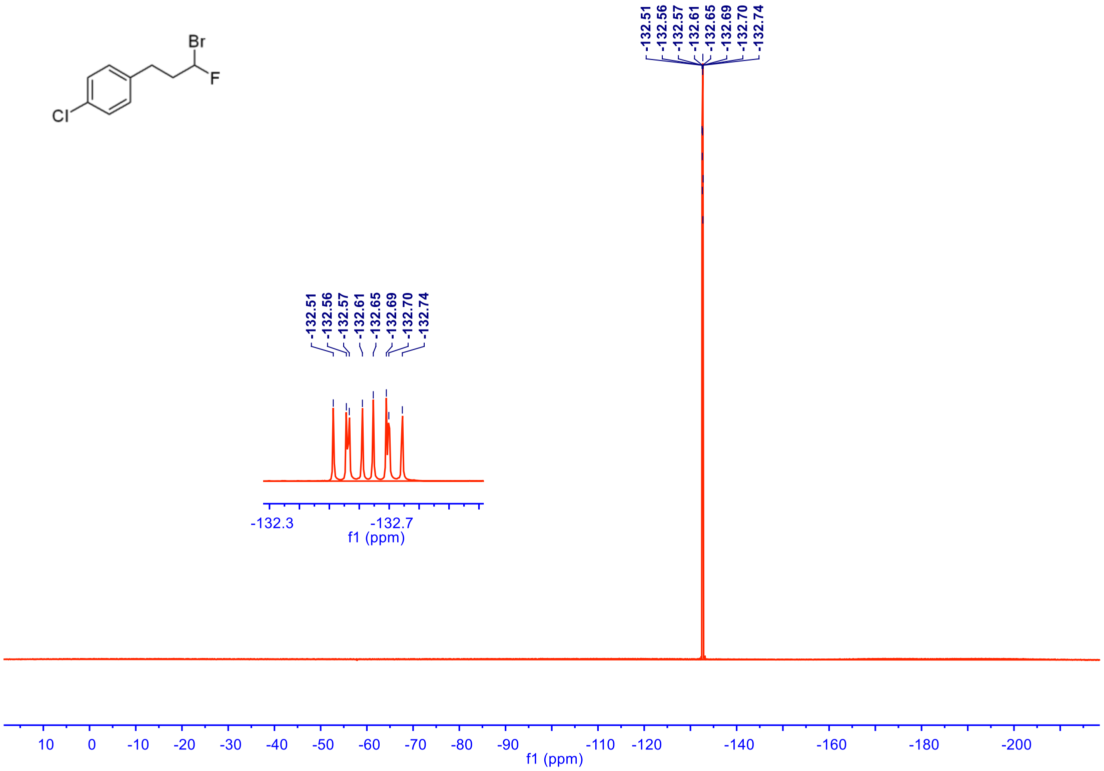

Supplementary Figure 159. ^{19}F NMR spectrum of **1-(3-bromo-3-fluoropropyl)-4-chlorobenzene 8a** (376 MHz, $CDCl_3$)

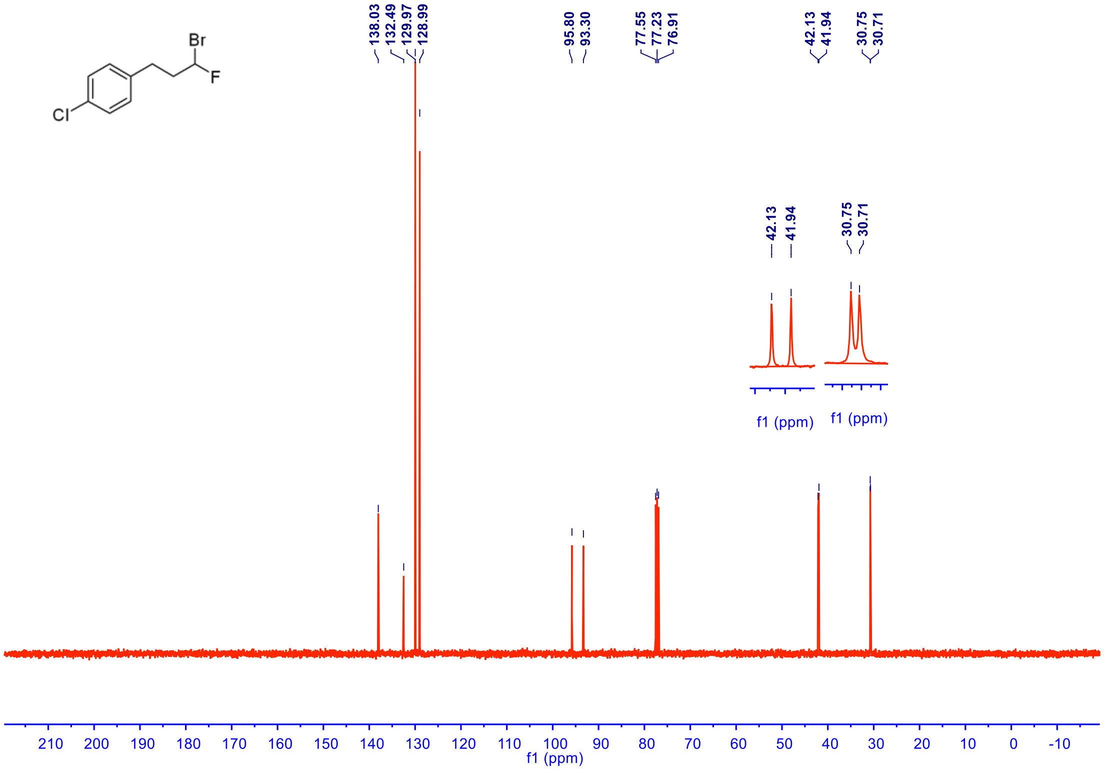

Supplementary Figure 160. ^{13}C NMR spectrum of **1-(3-bromo-3-fluoropropyl)-4-chlorobenzene 8a** (101 MHz, $CDCl_3$)

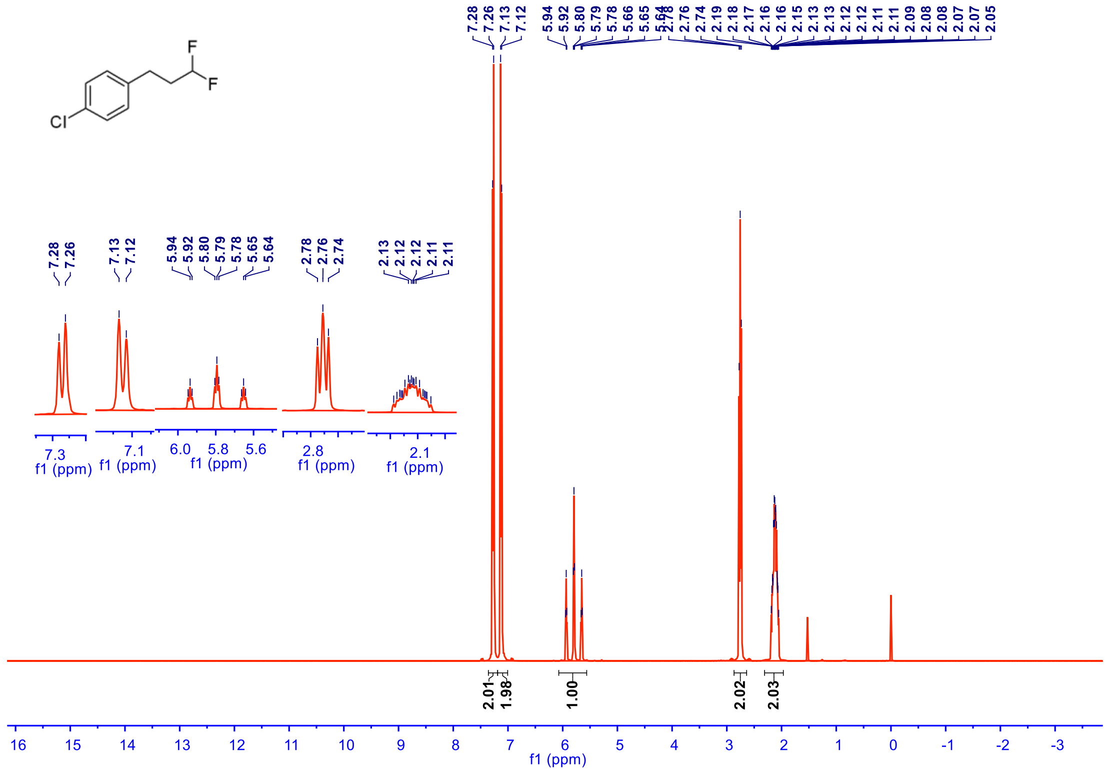

Supplementary Figure 161. ^{1}H NMR spectrum of compound **8** (400 MHz, $CDCl_3$)

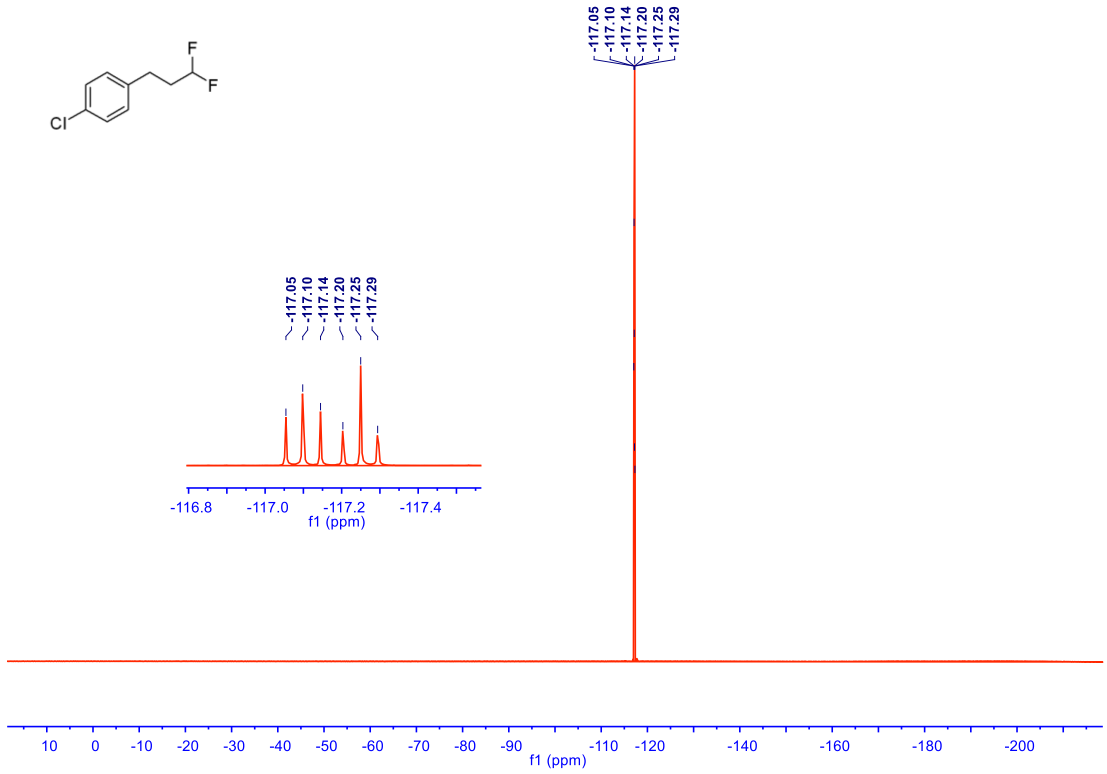

Supplementary Figure 162. ^{19}F NMR spectrum of compound **8** (376 MHz, $CDCl_3$)

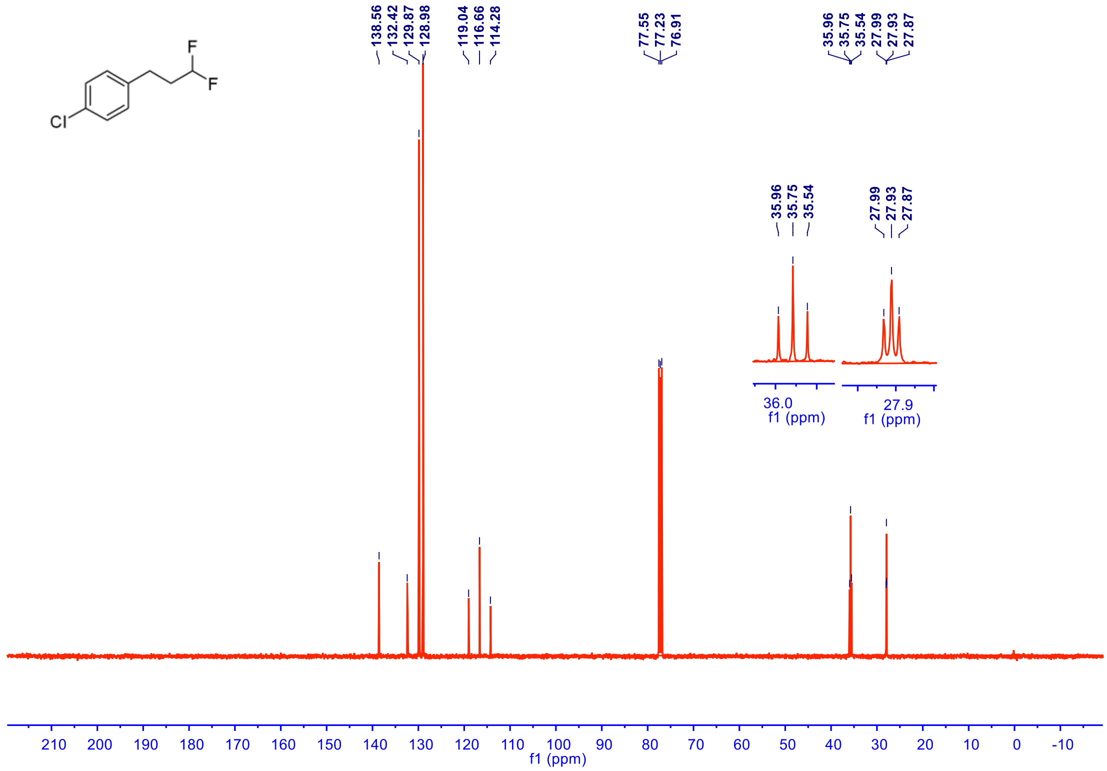

Supplementary Figure 163. ^{13}C NMR spectrum of compound **8** (101 MHz, $CDCl_3$)

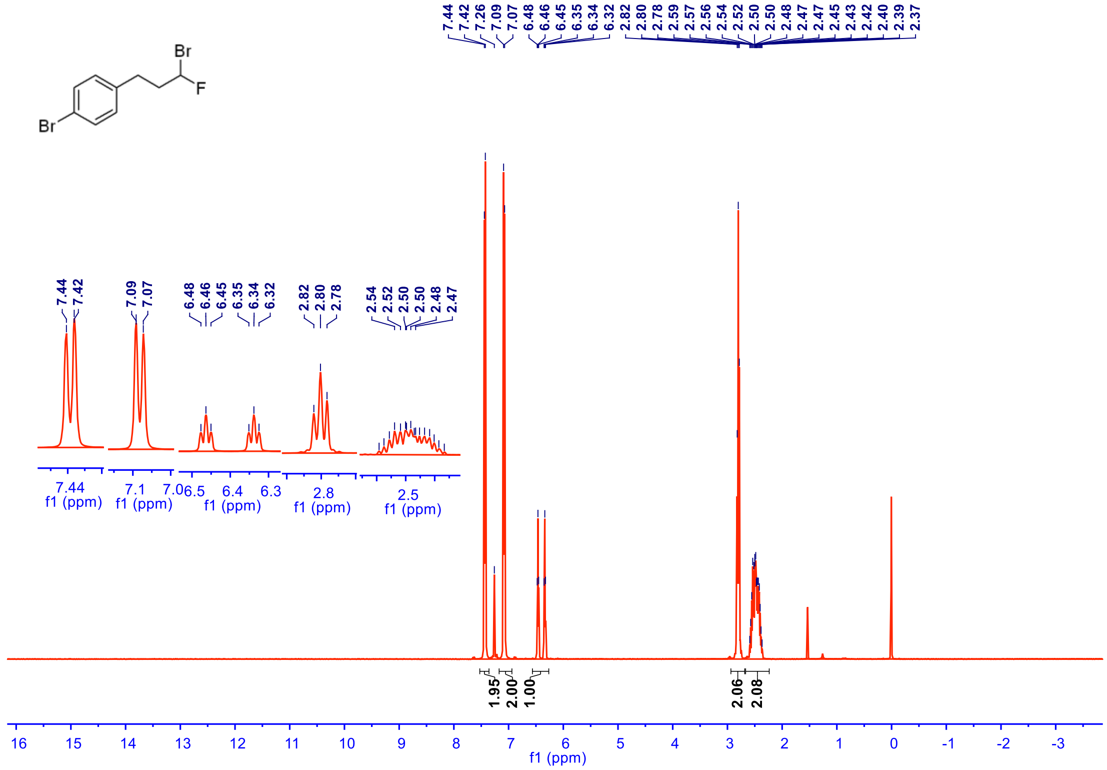

Supplementary Figure 164. ^{1}H NMR spectrum of **1-bromo-4-(3-bromo-3-fluoropropyl)benzene 9a** (400 MHz, $CDCl_3$)

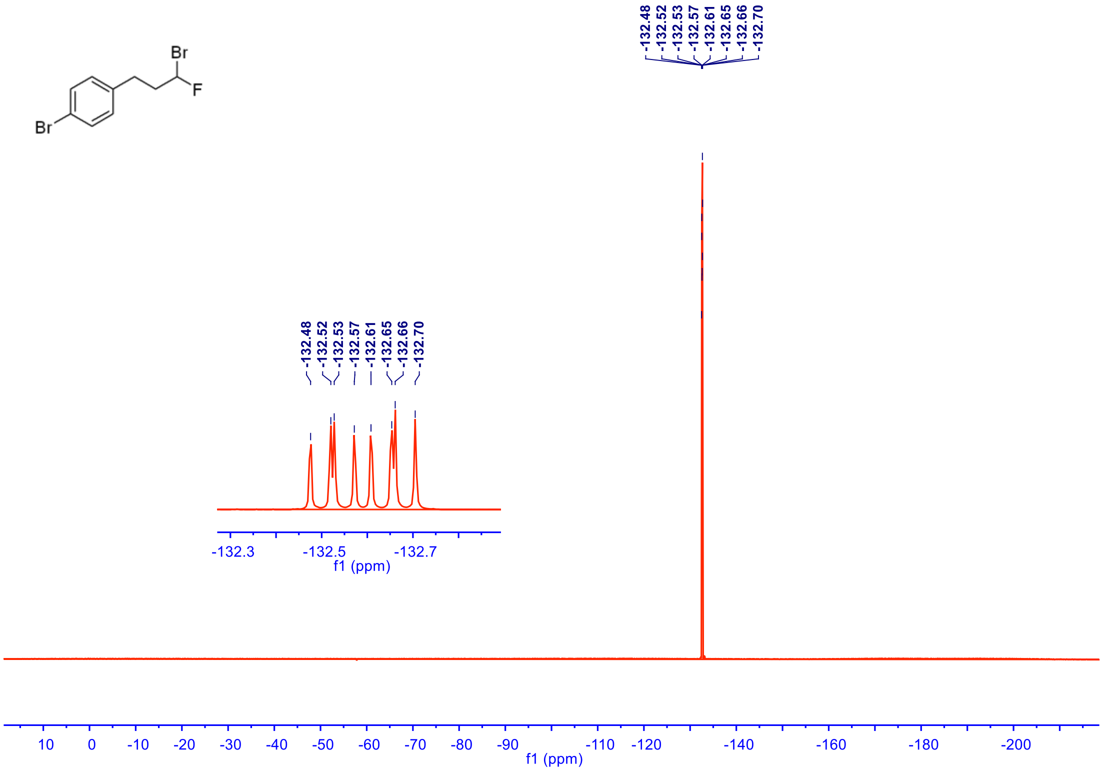

Supplementary Figure 165. ^{19}F NMR spectrum of **1-bromo-4-(3-bromo-3-fluoropropyl)benzene 9a** (376 MHz, $CDCl_3$)

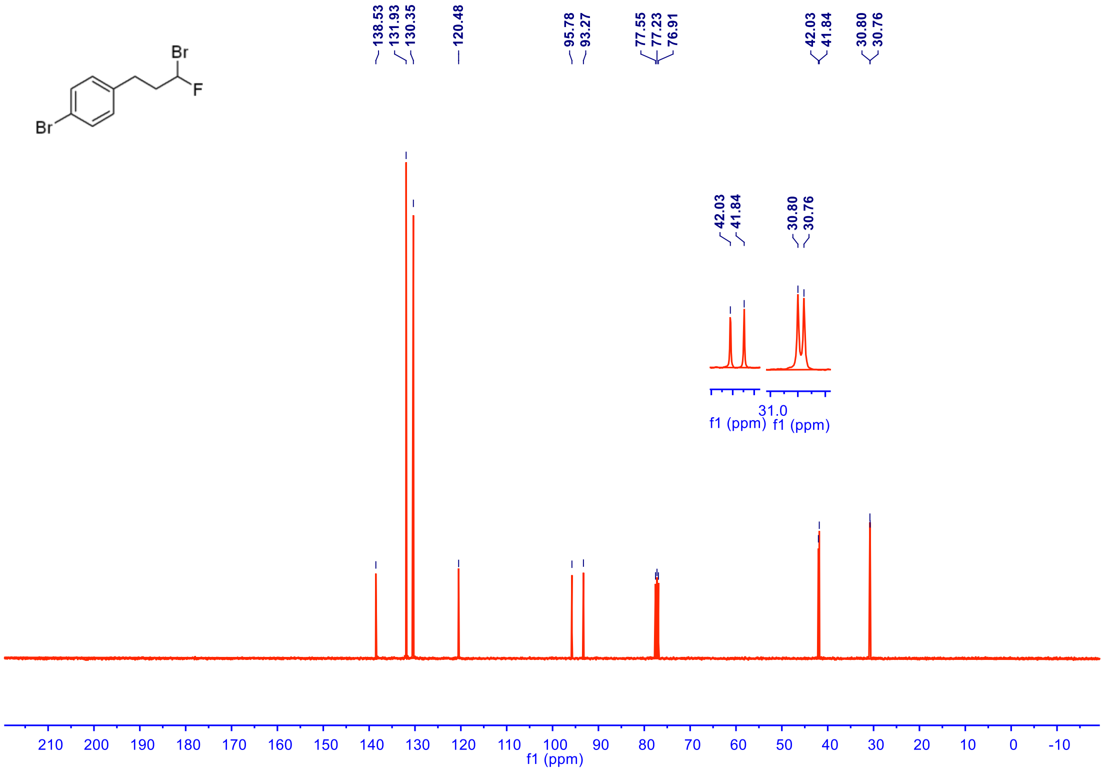

Supplementary Figure 166. ^{13}C NMR spectrum of **1-bromo-4-(3-bromo-3-fluoropropyl)benzene 9a** (101 MHz, $CDCl_3$)

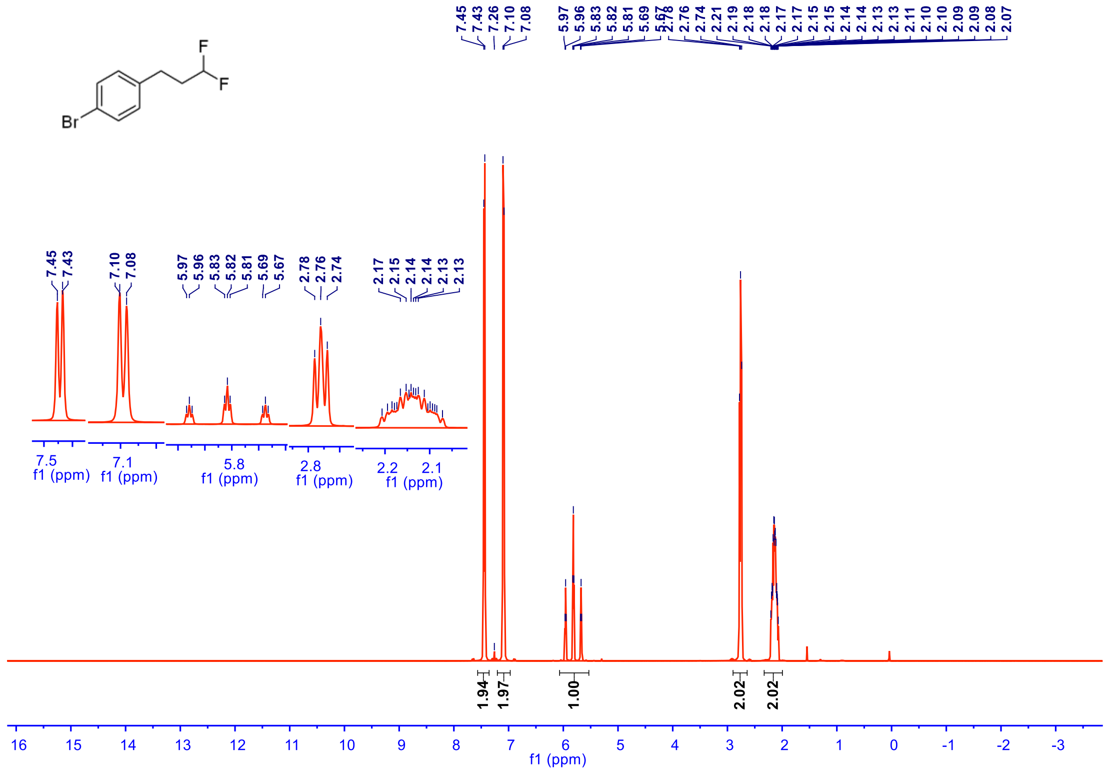

Supplementary Figure 167. ^{1}H NMR spectrum of compound **9** (400 MHz, $CDCl_3$)

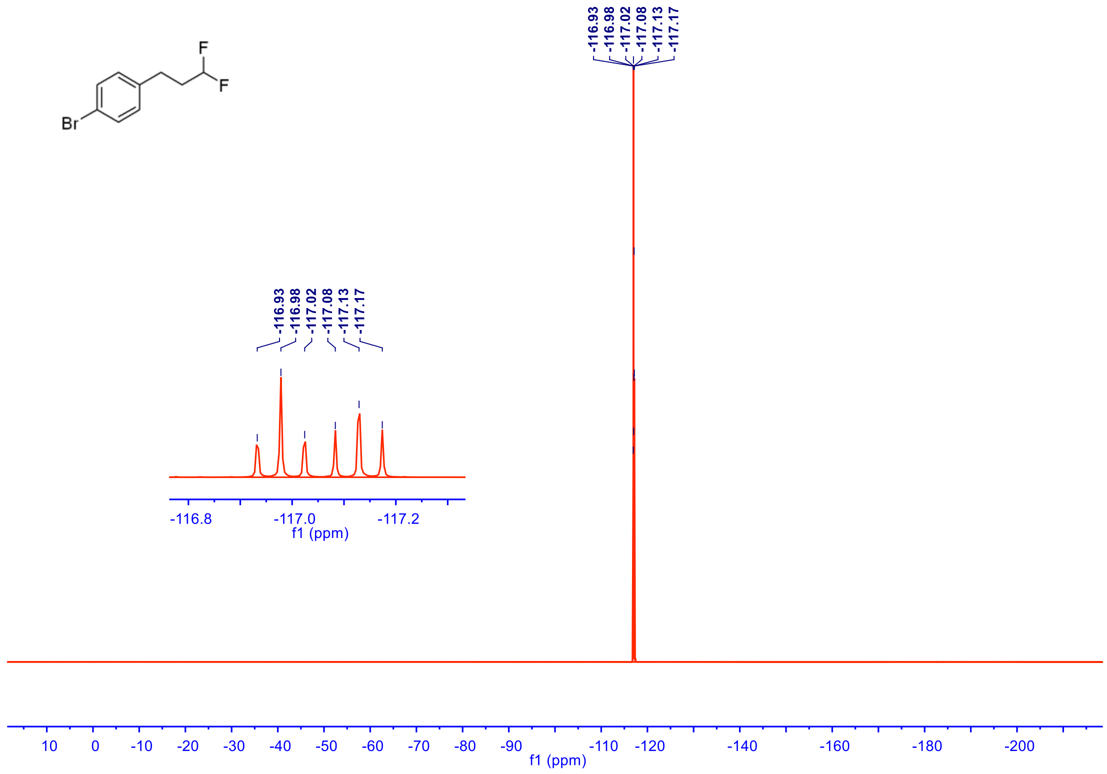

Supplementary Figure 168. ^{19}F NMR spectrum of compound **9** (376 MHz, $CDCl_3$)

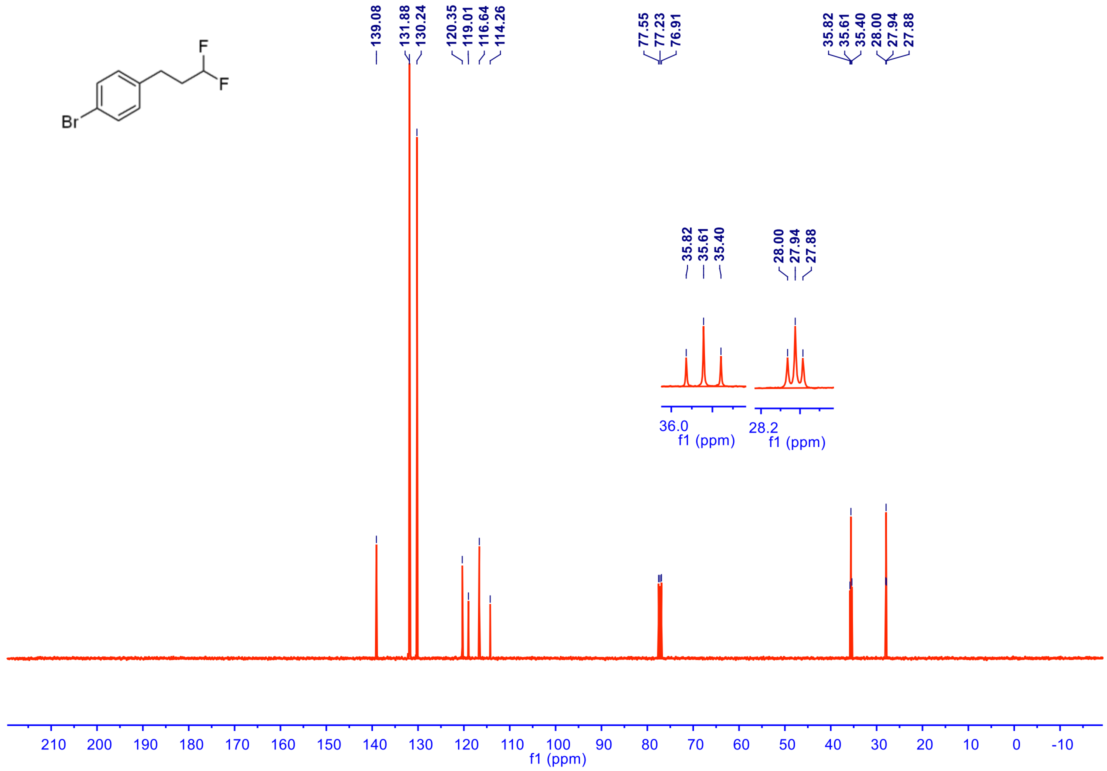

Supplementary Figure 169. ^{13}C NMR spectrum of compound **9** (101 MHz, $CDCl_3$)

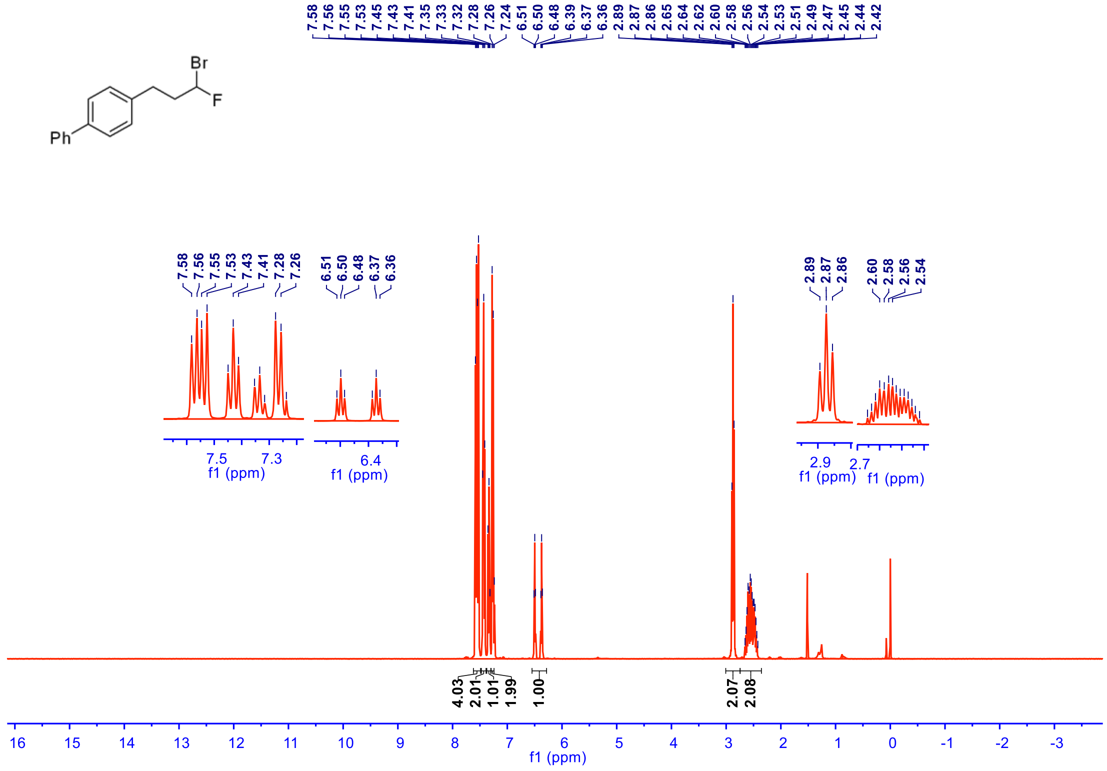

Supplementary Figure 170. ^{1}H NMR spectrum of **4-(3-bromo-3-fluoropropyl)-1,1'-biphenyl 10a** (400 MHz, $CDCl_3$)

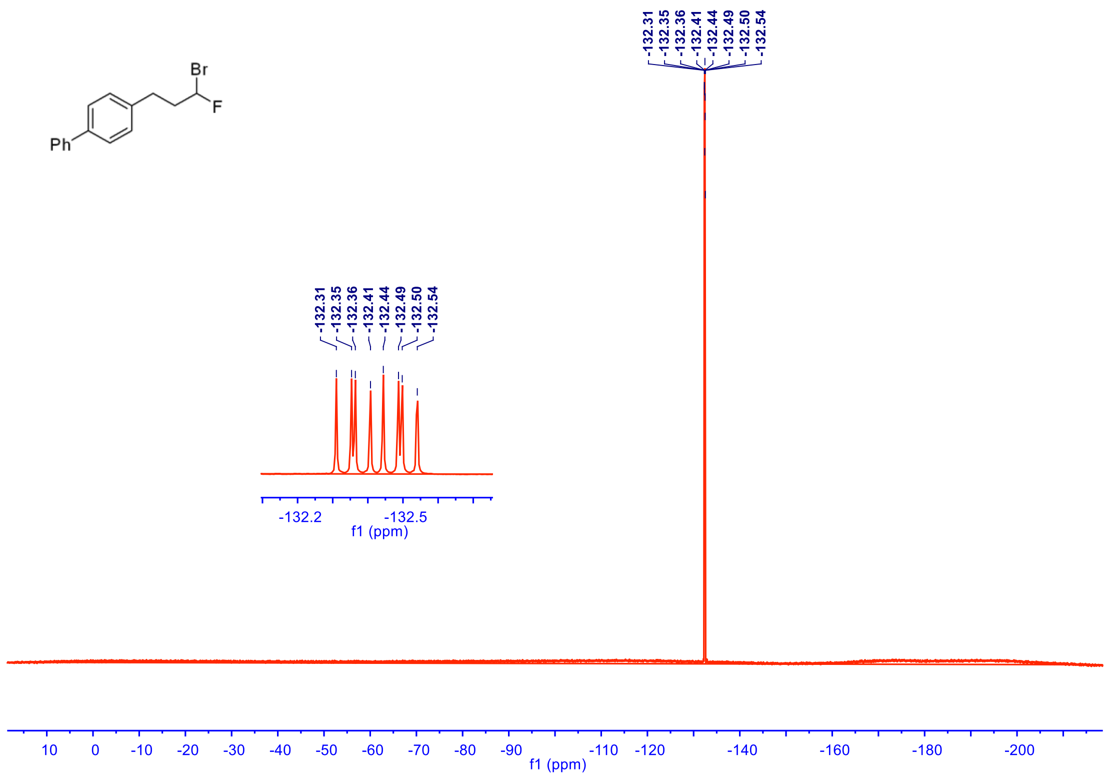

Supplementary Figure 171. ^{19}F NMR spectrum of **4-(3-bromo-3-fluoropropyl)-1,1'-biphenyl 10a** (376 MHz, $CDCl_3$)

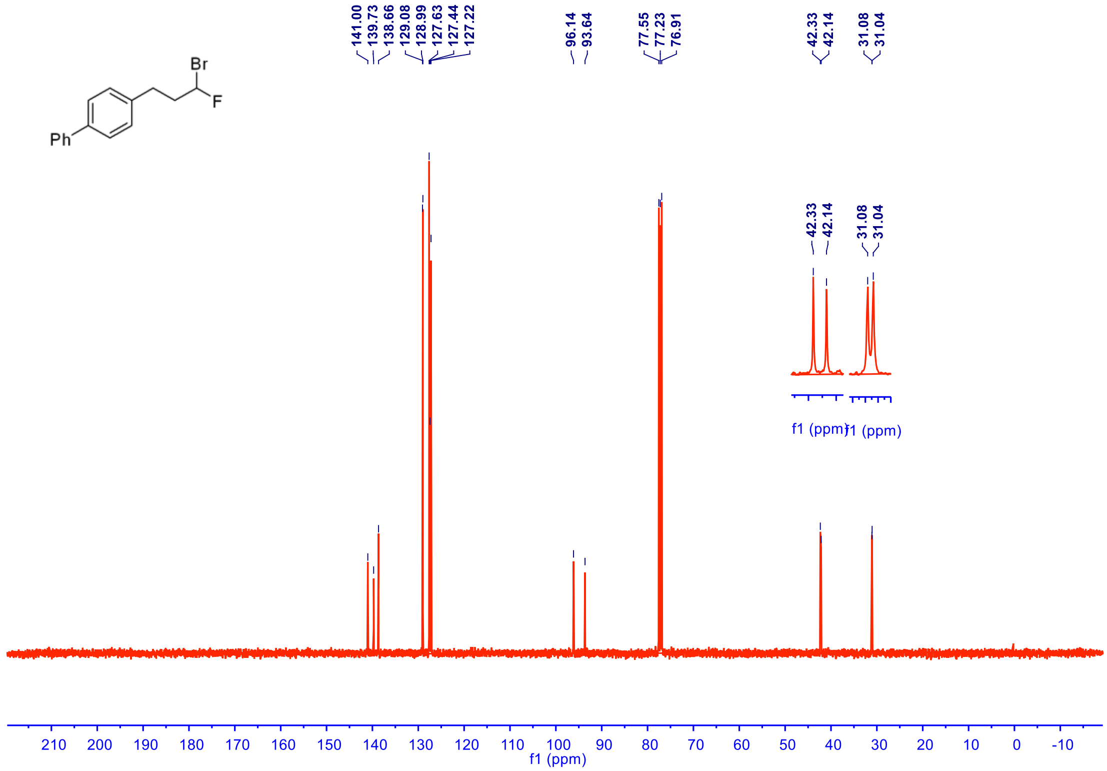

Supplementary Figure 172. ^{13}C NMR spectrum of **4-(3-bromo-3-fluoropropyl)-1,1'-biphenyl 10a** (101 MHz, $CDCl_3$)

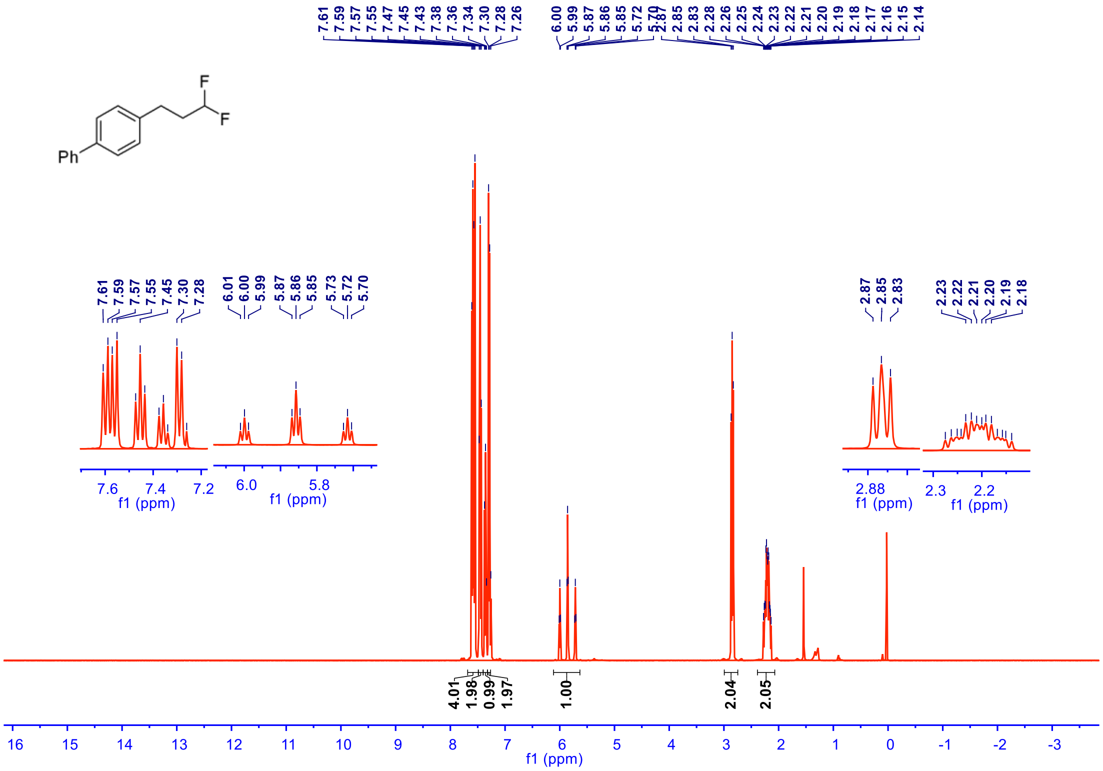

Supplementary Figure 173. ^{1}H NMR spectrum of compound **10** (400 MHz, $CDCl_3$)

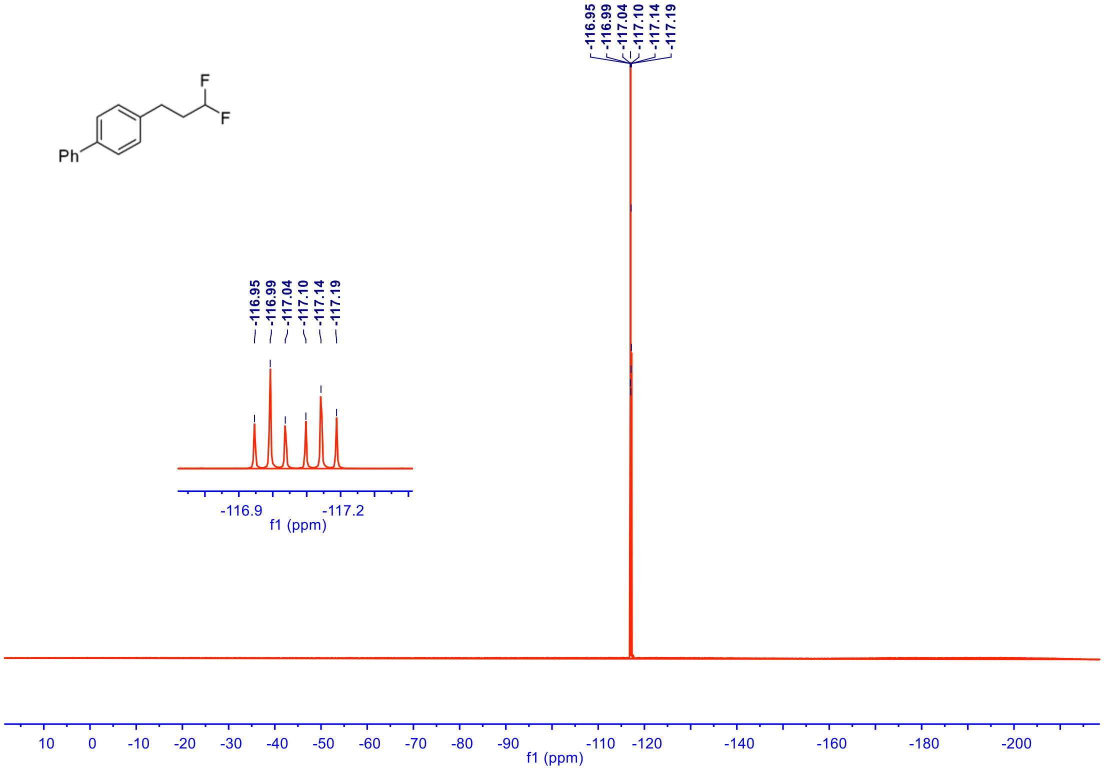

Supplementary Figure 174. ^{19}F NMR spectrum of compound **10** (376 MHz, $CDCl_3$)

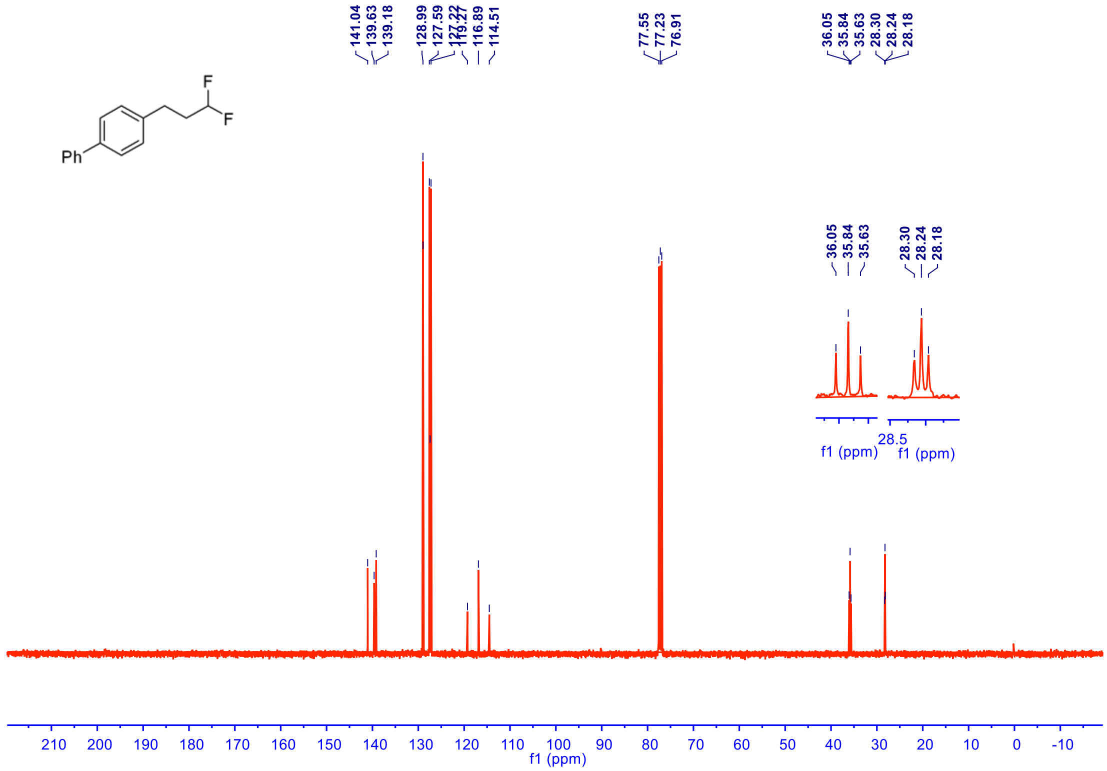

Supplementary Figure 175. ^{13}C NMR spectrum of compound **10** (101 MHz, $CDCl_3$)

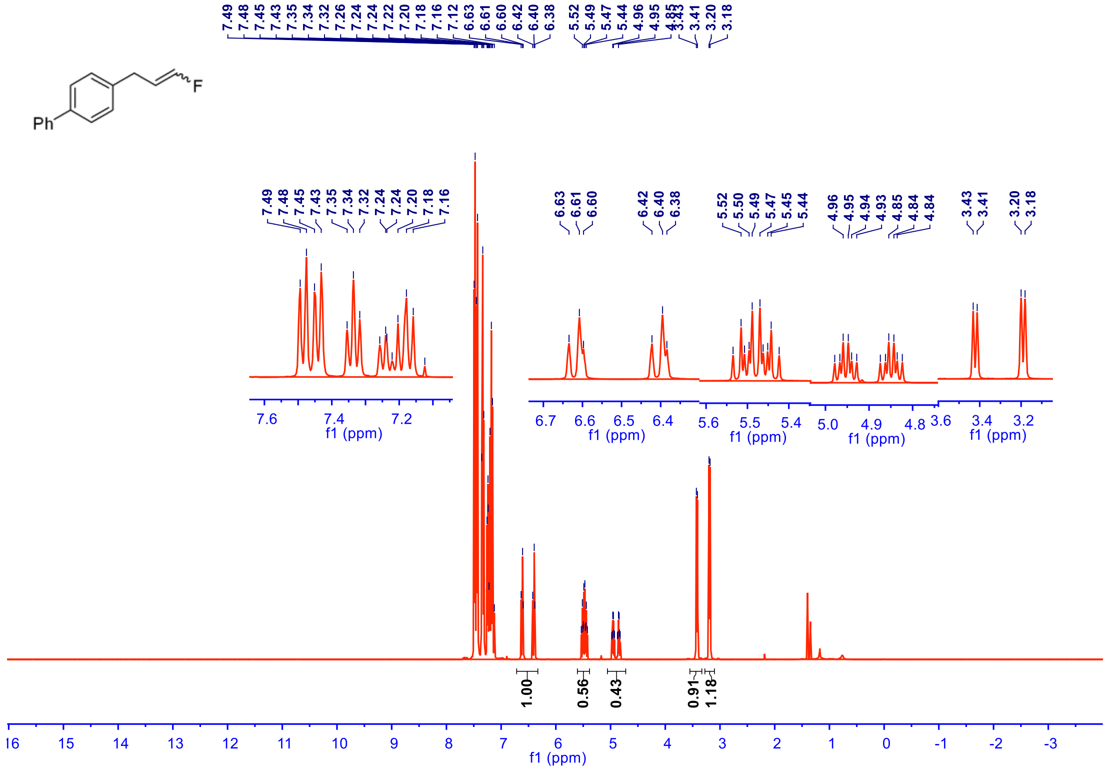

Supplementary Figure 176. ^{1}H NMR spectrum of compound **10b** (400 MHz, $CDCl_3$)

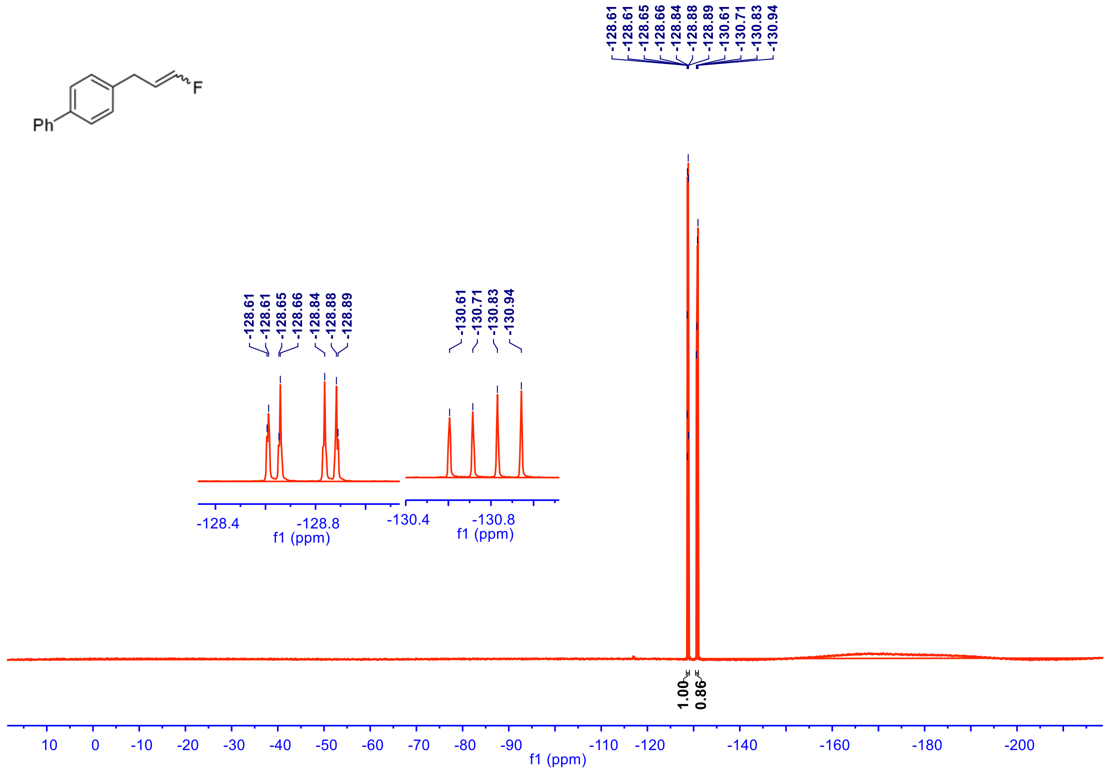

Supplementary Figure 177. ^{19}F NMR spectrum of compound **10b** (376 MHz, $CDCl_3$)

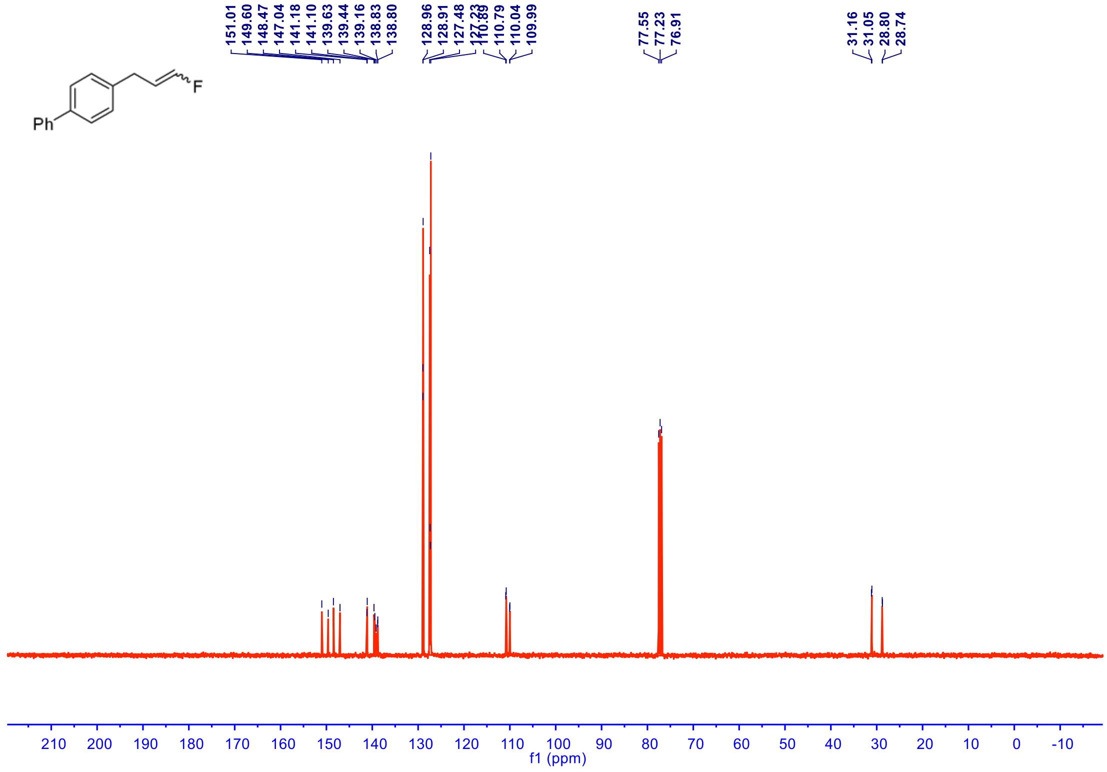

Supplementary Figure 178. ^{13}C NMR spectrum of compound **10b** (101 MHz, $CDCl_3$)

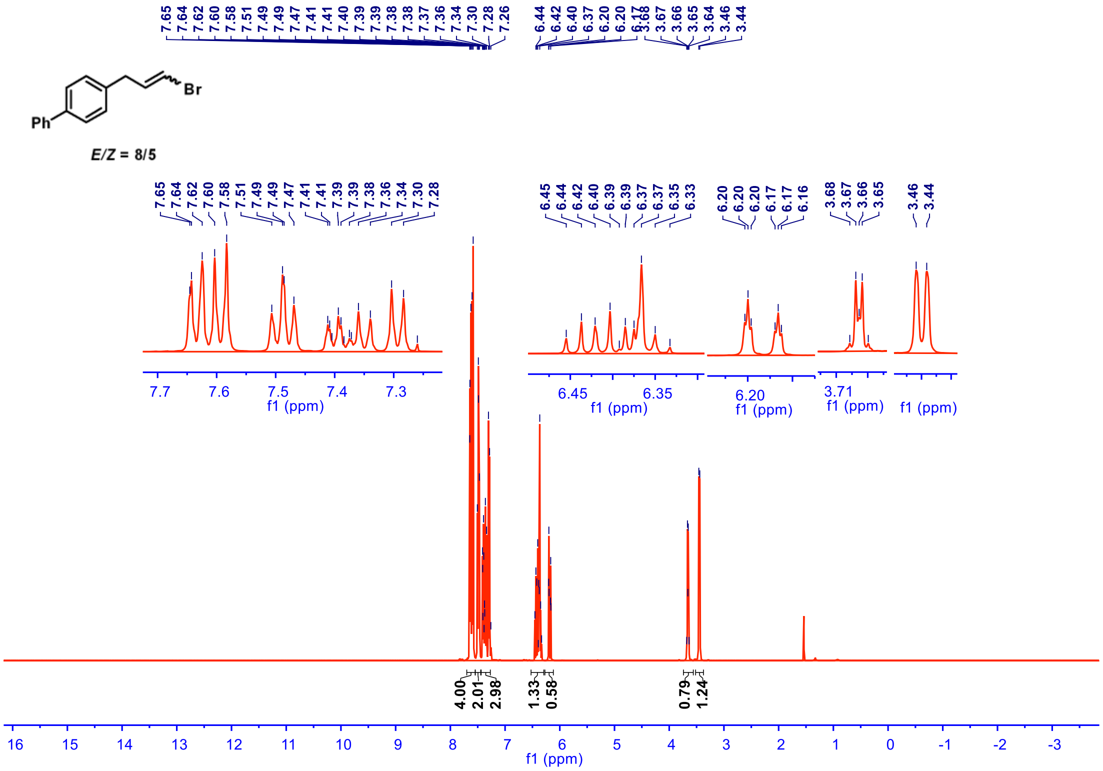

Supplementary Figure 179. ^{1}H NMR spectrum of compound **10c** (400 MHz, $CDCl_3$)

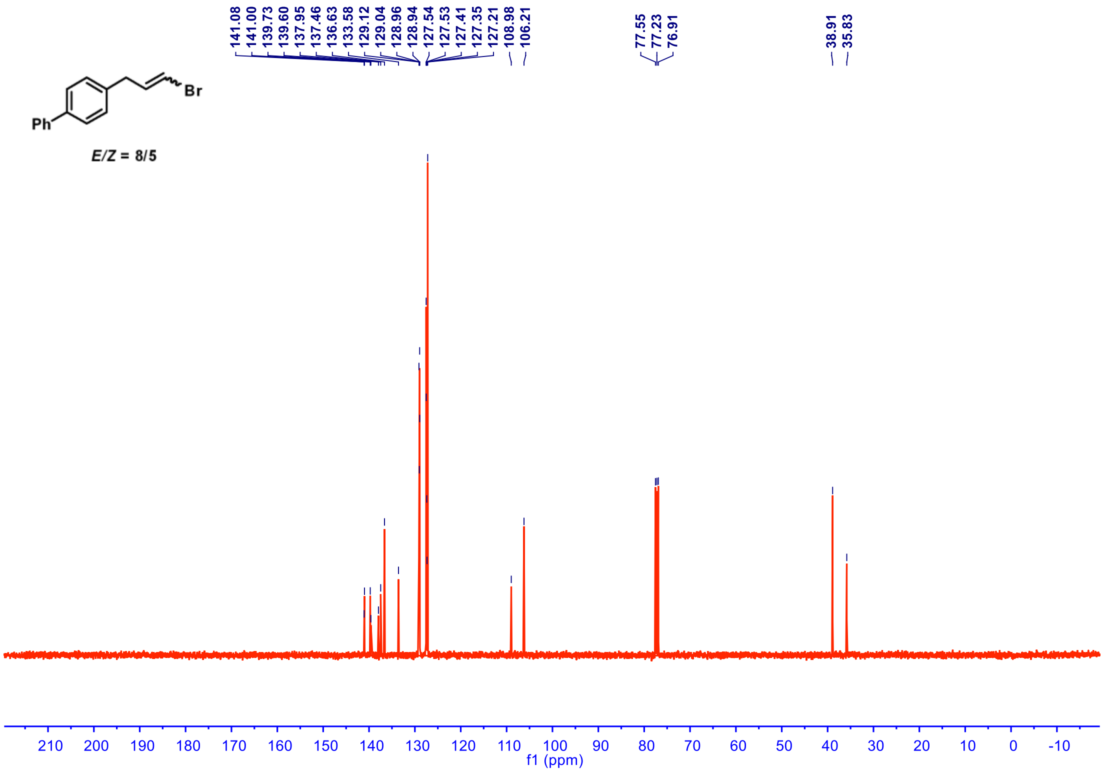

Supplementary Figure 180. ^{13}C NMR spectrum of compound **10c** (101 MHz, $CDCl_3$)

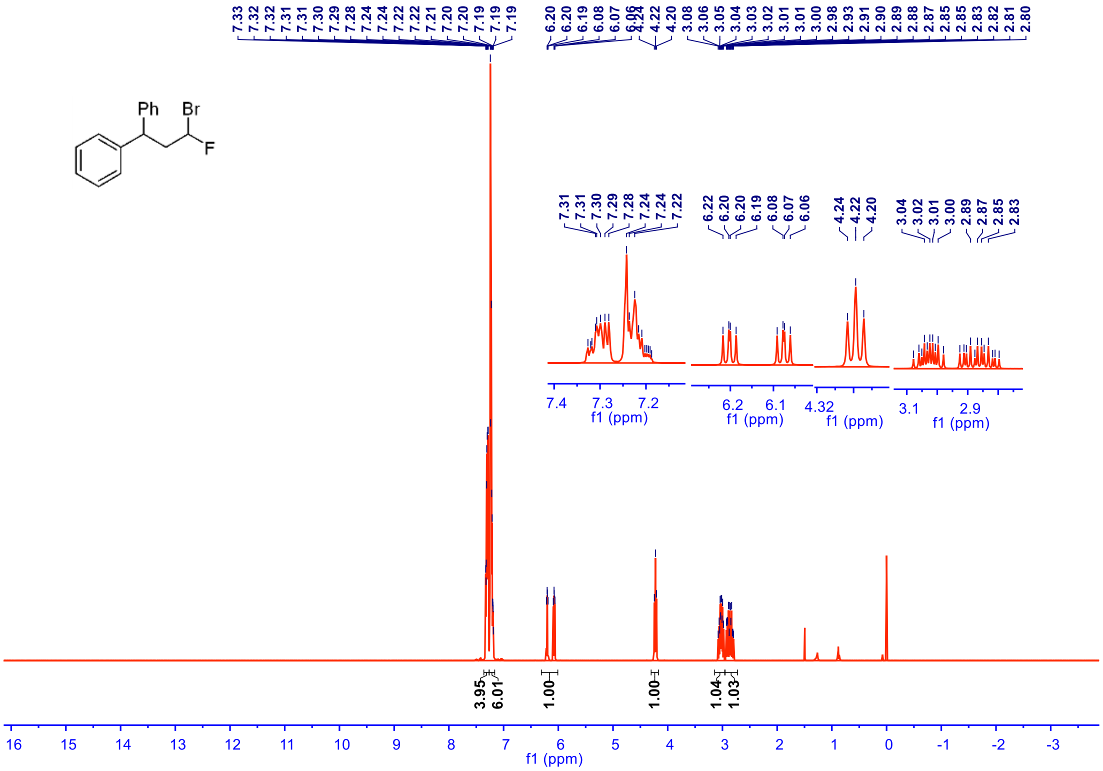

Supplementary Figure 181. ^{1}H NMR spectrum of **(3-bromo-3-fluoropropane-1,1-diyl)dibenzene 11a** (400 MHz, $CDCl_3$)

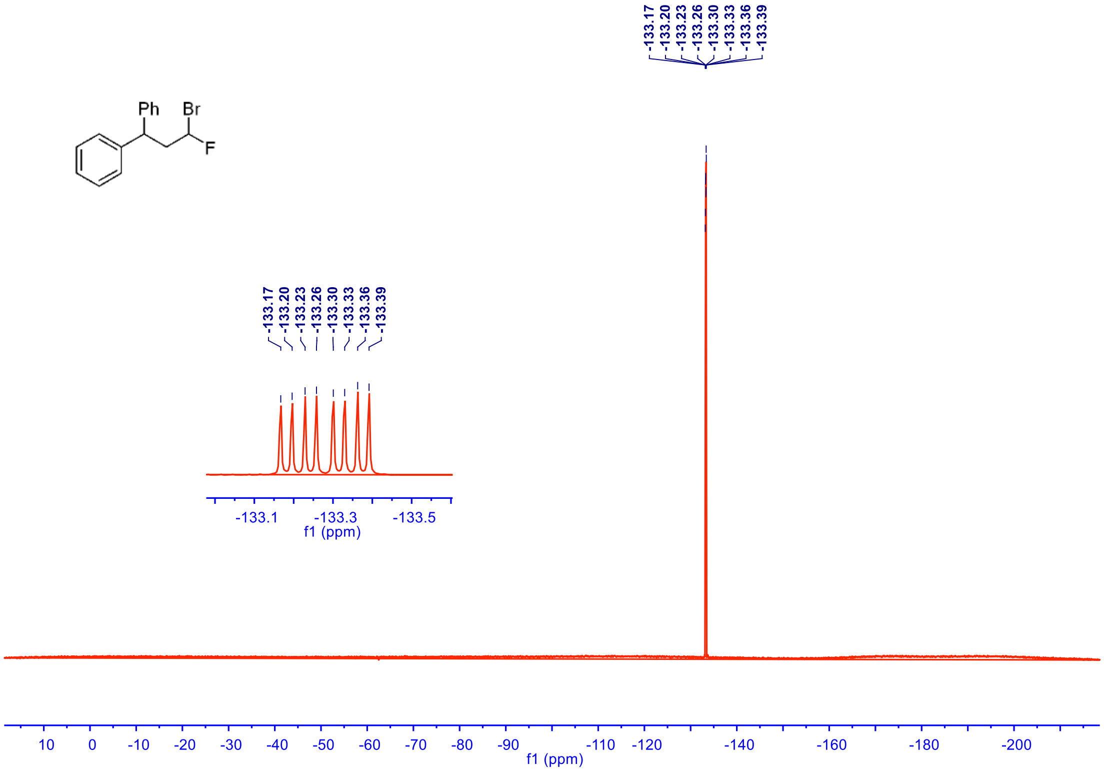

Supplementary Figure 182. ^{19}F NMR spectrum of **(3-bromo-3-fluoropropane-1,1-diyl)dibenzene 11a** (376 MHz, $CDCl_3$)

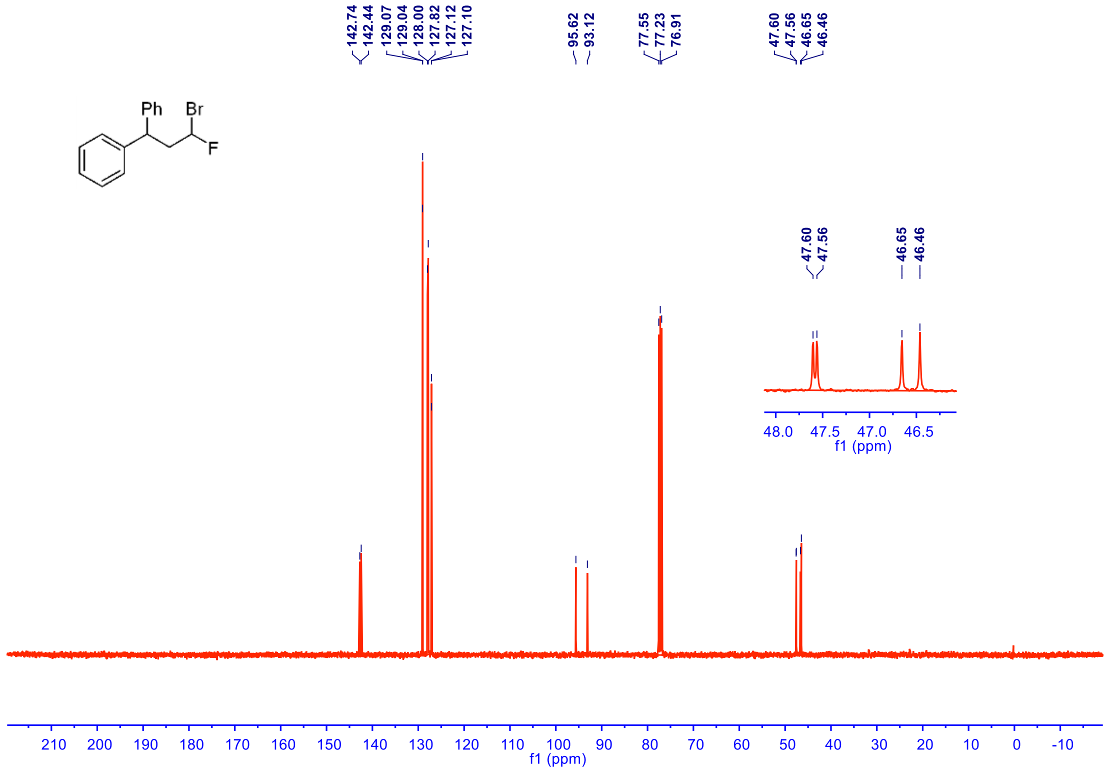

Supplementary Figure 183. ^{13}C NMR spectrum of **(3-bromo-3-fluoropropane-1,1-diyl)dibenzene 11a** (101 MHz, $CDCl_3$)

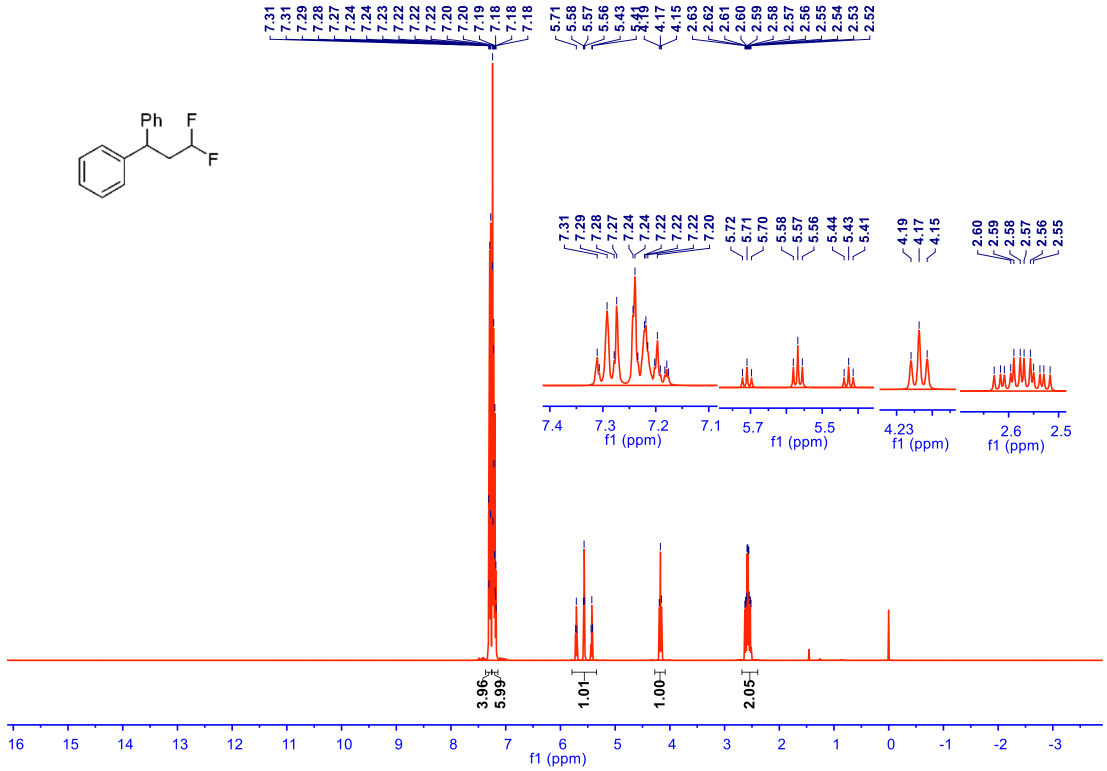

Supplementary Figure 184. ^{1}H NMR spectrum of compound **11** (400 MHz, $CDCl_3$)

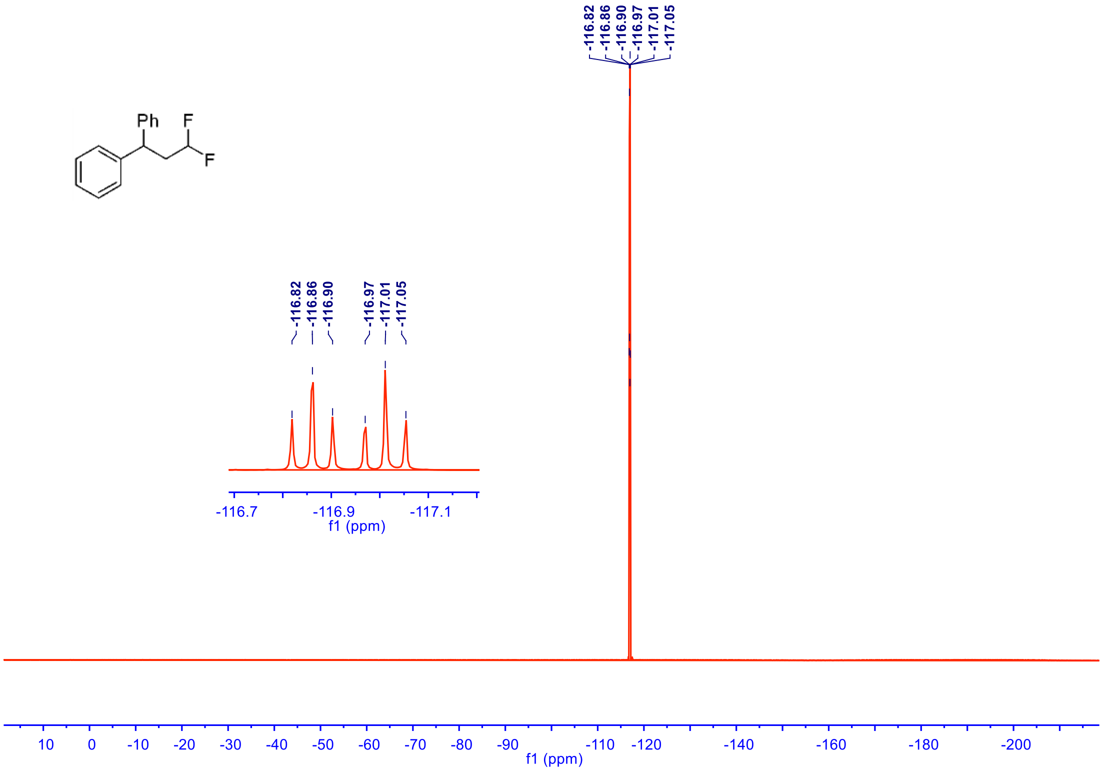

Supplementary Figure 185. ^{19}F NMR spectrum of compound **11** (376 MHz, $CDCl_3$)

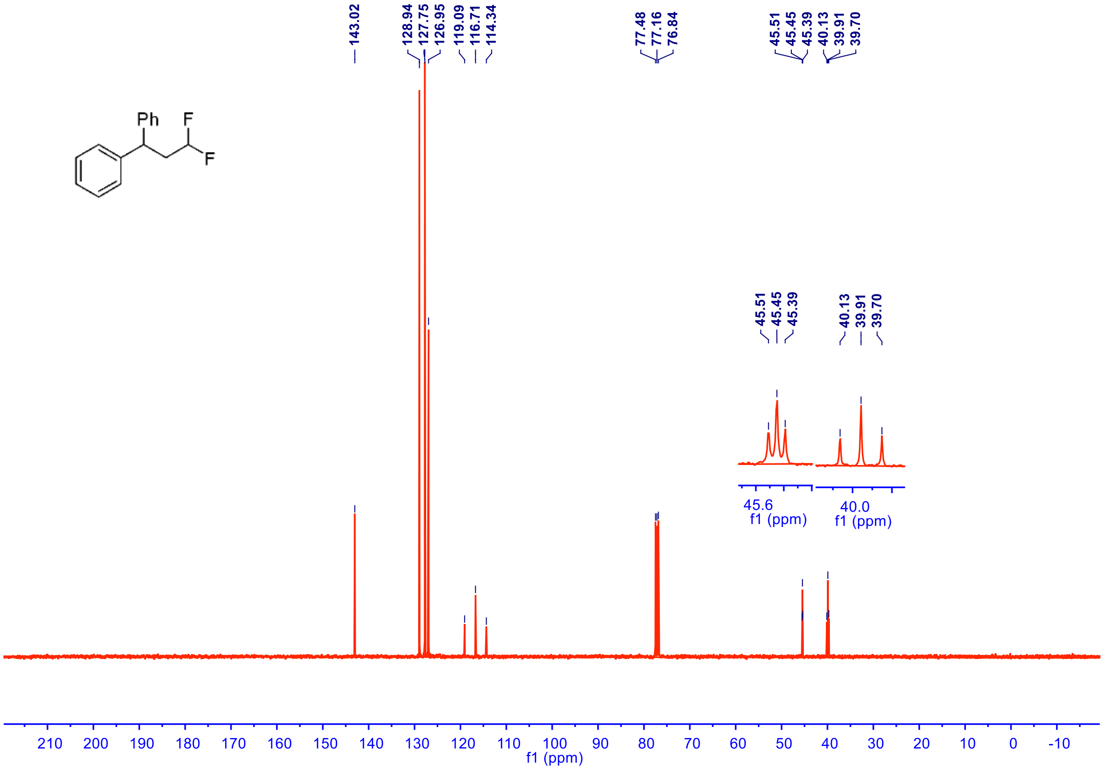

Supplementary Figure 186. ^{13}C NMR spectrum of compound **11** (101 MHz, $CDCl_3$)

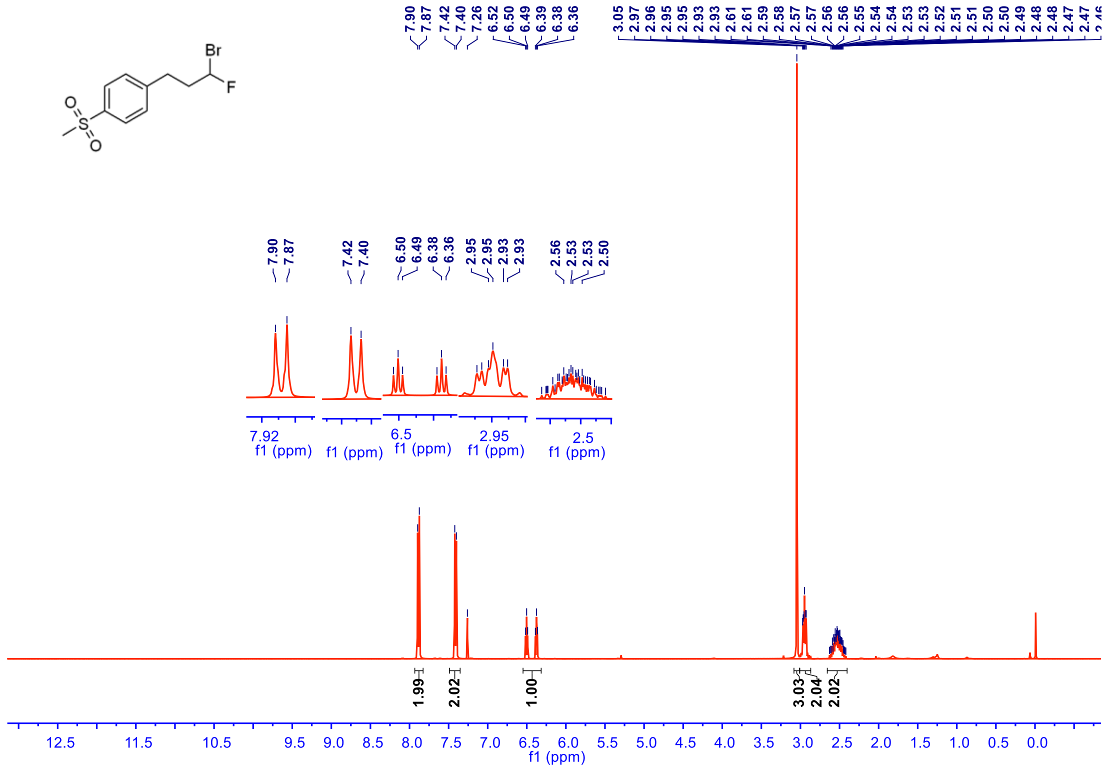

Supplementary Figure 187. ^{1}H NMR spectrum of **1-(3-bromo-3-fluoropropyl)-4-(methylsulfonyl)benzene 12a** (400 MHz, $CDCl_3$)

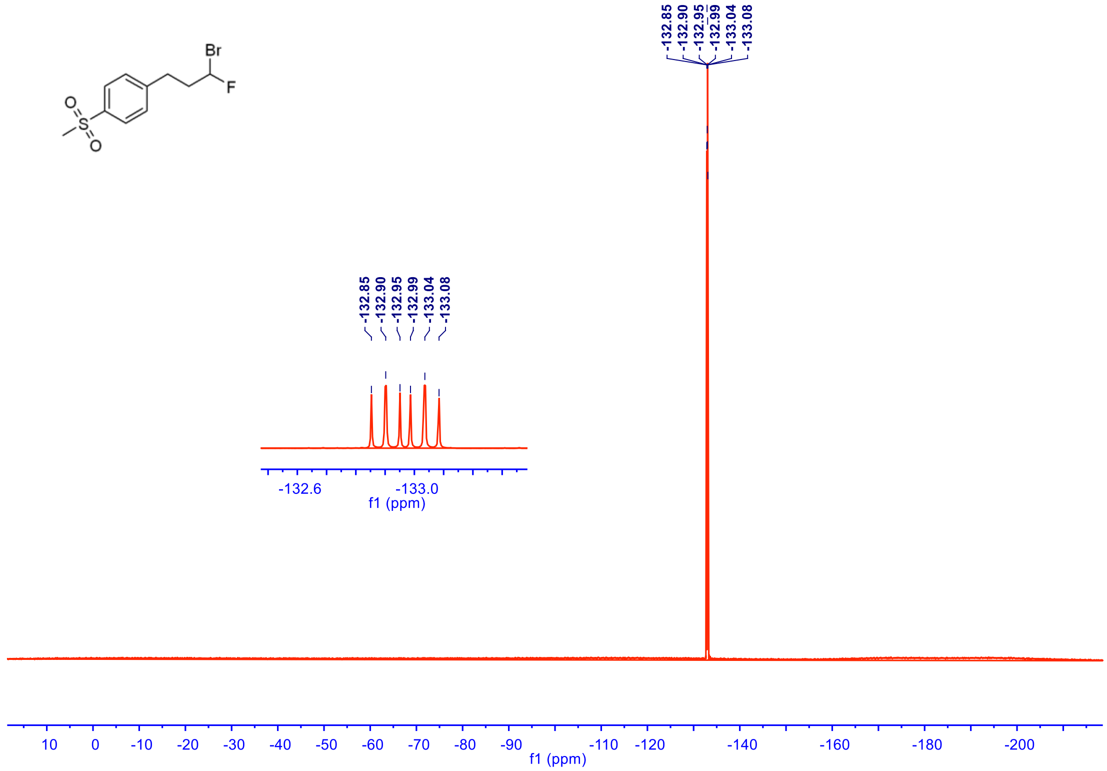

Supplementary Figure 188. ^{19}F NMR spectrum of **1-(3-bromo-3-fluoropropyl)-4-(methylsulfonyl)benzene 12a** (376 MHz, $CDCl_3$)

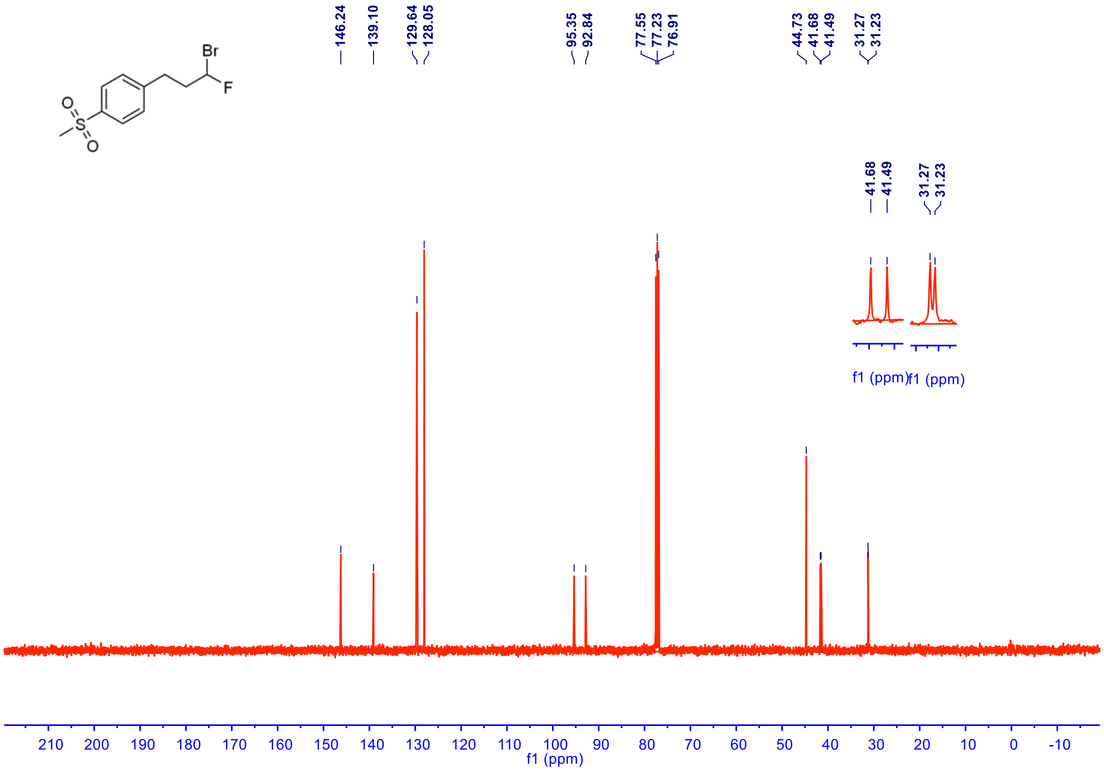

Supplementary Figure 189. ^{13}C NMR spectrum of **1-(3-bromo-3-fluoropropyl)-4-(methylsulfonyl)benzene 12a** (101 MHz, $CDCl_3$)

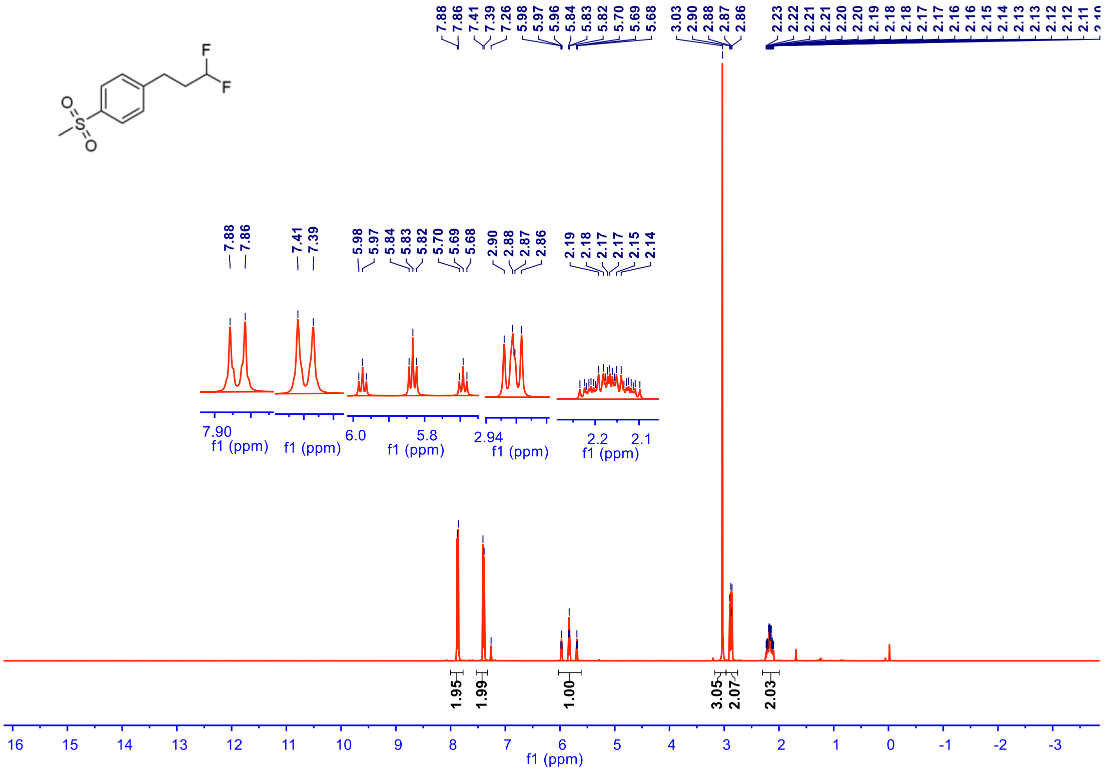

Supplementary Figure 190. ^{1}H NMR spectrum of compound **12** (400 MHz, $CDCl_3$)

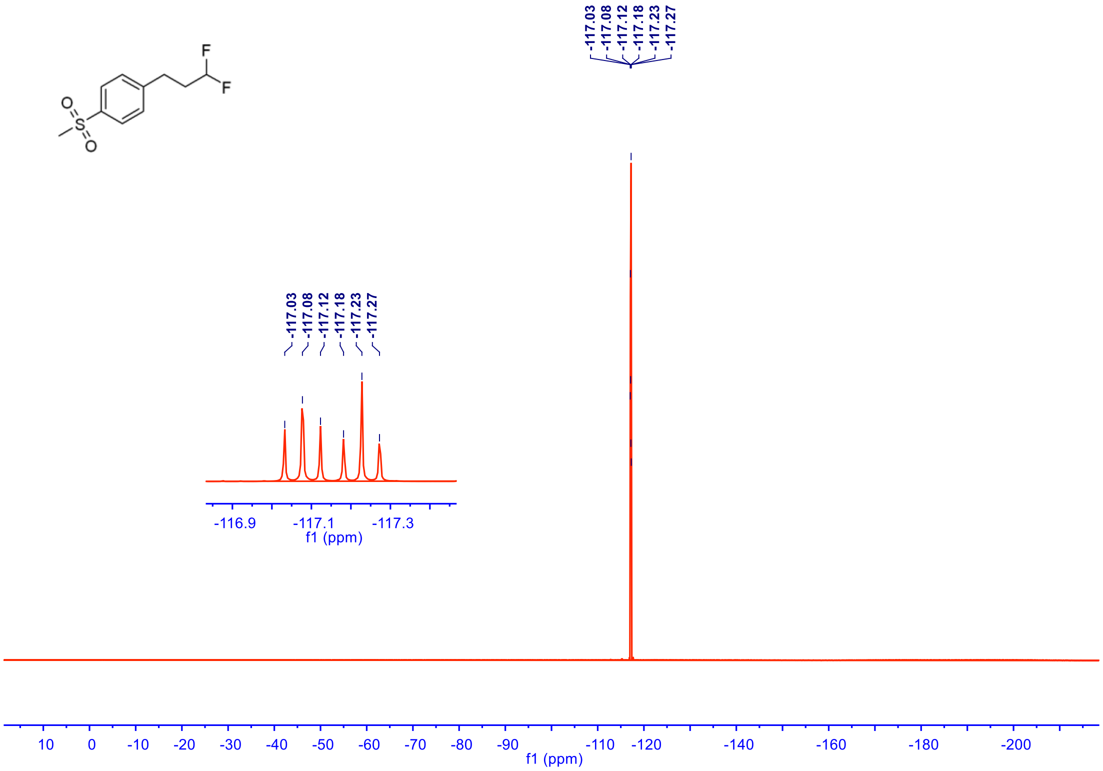

Supplementary Figure 191. ^{19}F NMR spectrum of compound **12** (376 MHz, $CDCl_3$)

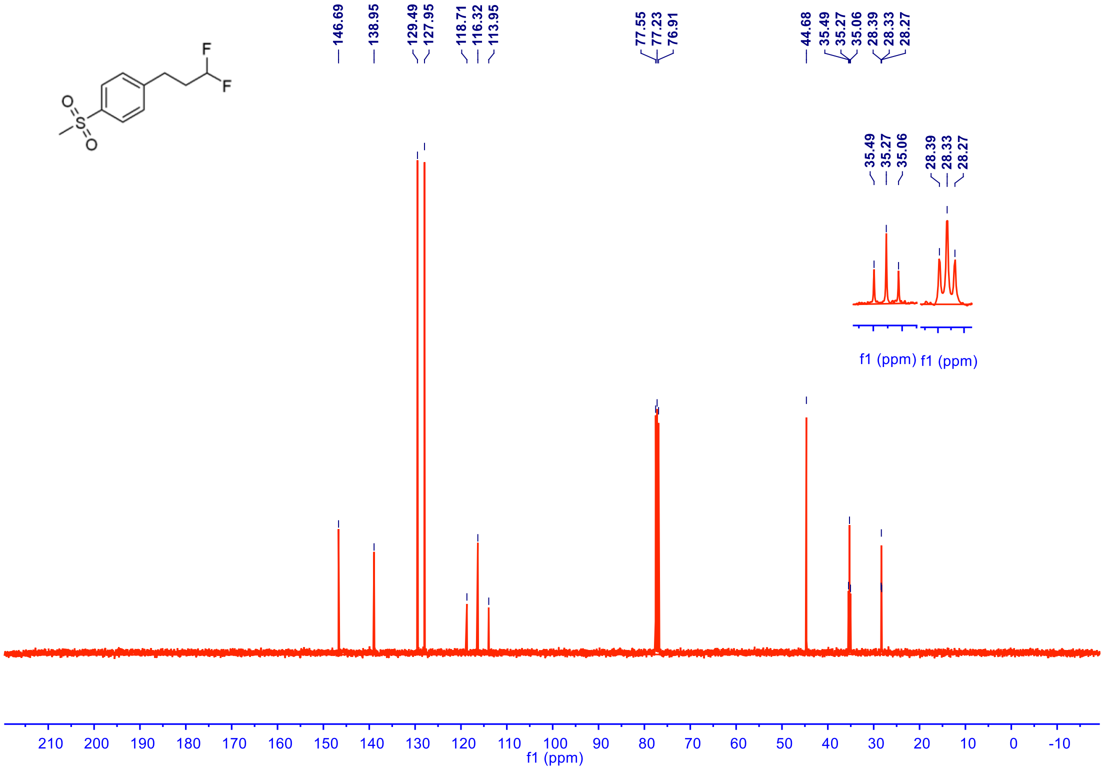

Supplementary Figure 192. ^{13}C NMR spectrum of compound **12** (101 MHz, $CDCl_3$)

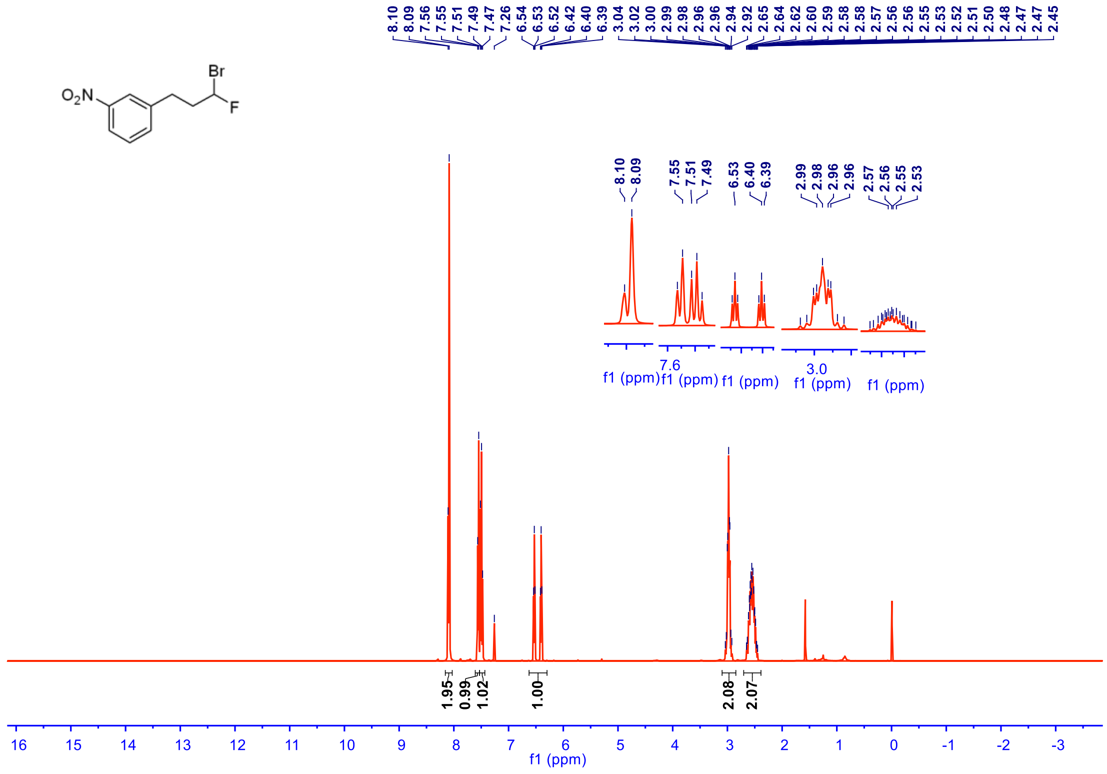

Supplementary Figure 193. ^{1}H NMR spectrum of **1-(3-bromo-3-fluoropropyl)-3-nitrobenzene 13a** (400 MHz, $CDCl_3$)

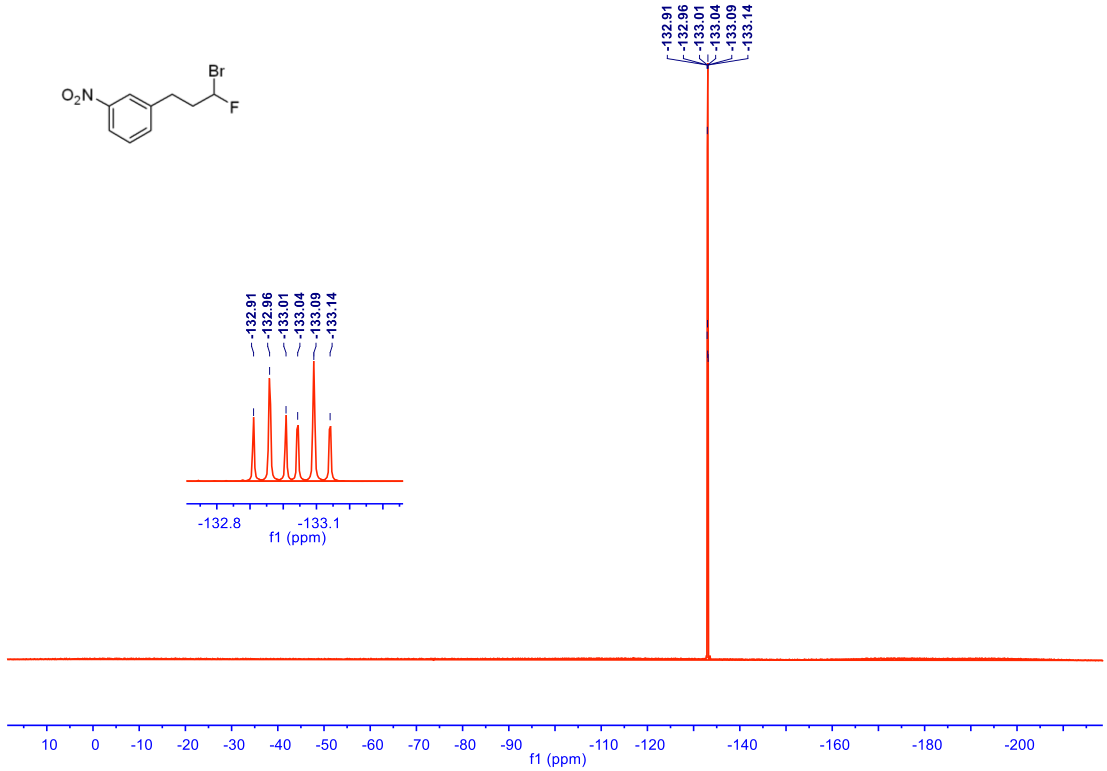

Supplementary Figure 194. ^{19}F NMR spectrum of **1-(3-bromo-3-fluoropropyl)-3-nitrobenzene 13a** (376 MHz, $CDCl_3$)

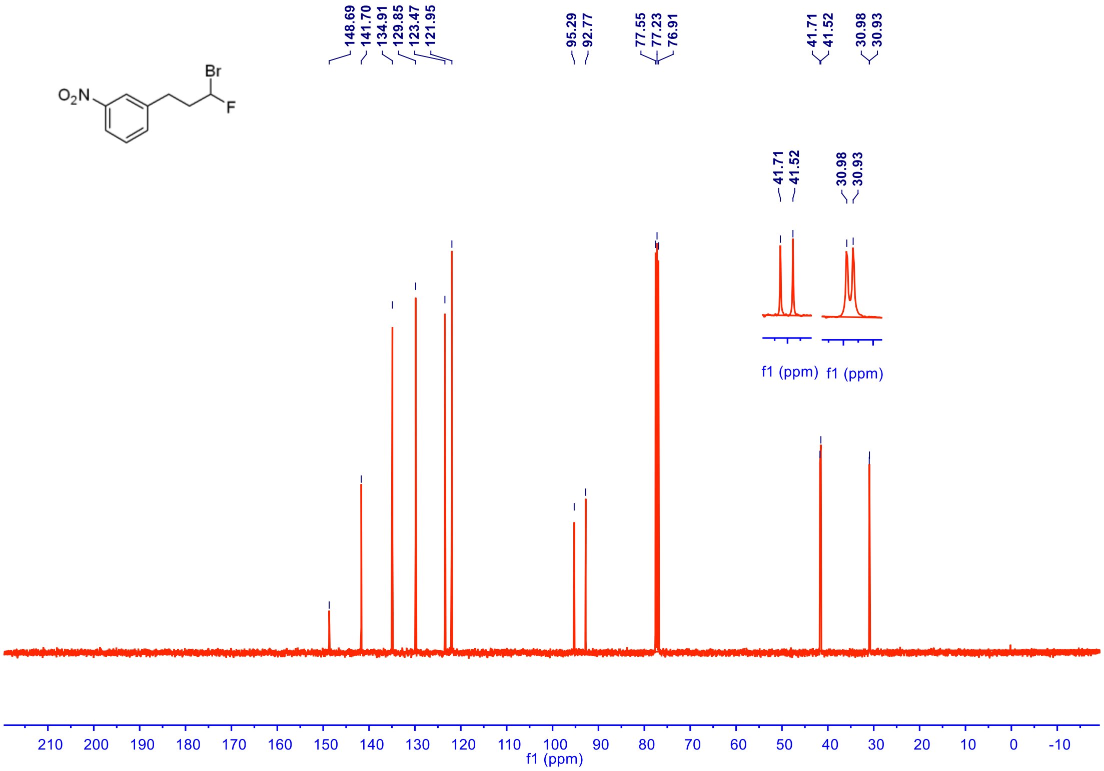

Supplementary Figure 195. ^{13}C NMR spectrum of **1-(3-bromo-3-fluoropropyl)-3-nitrobenzene 13a** (101 MHz, $CDCl_3$)

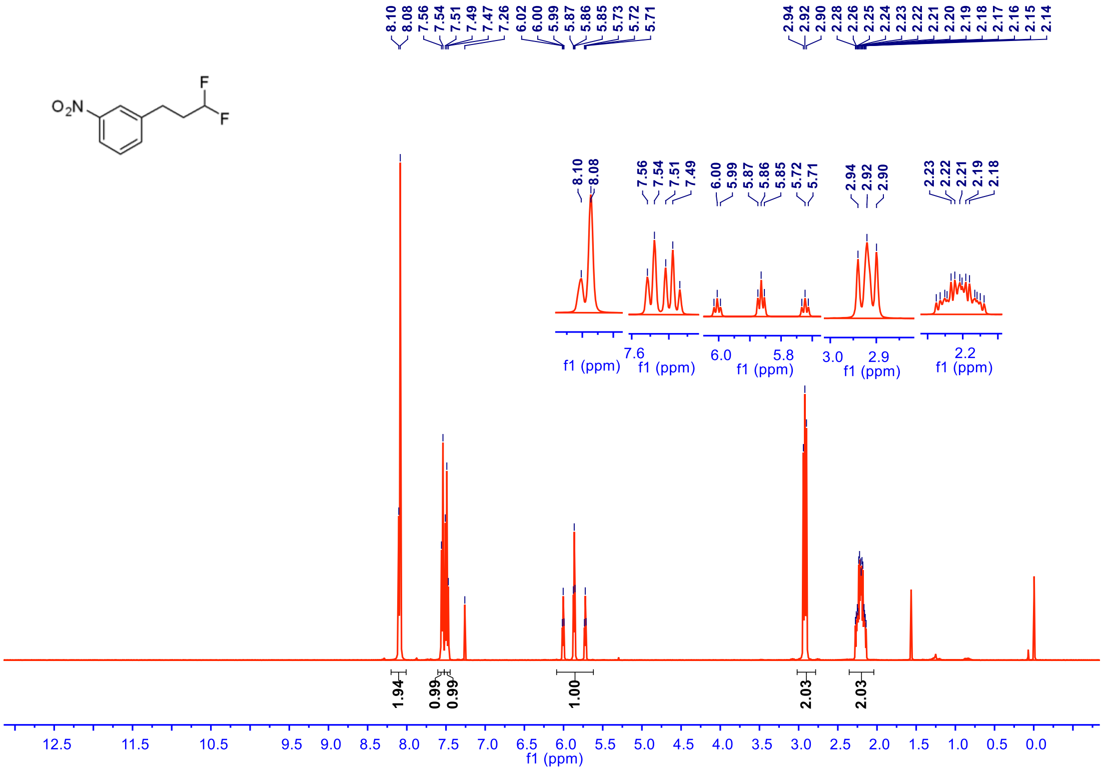

Supplementary Figure 196. ^{1}H NMR spectrum of compound **13** (400 MHz, $CDCl_3$)

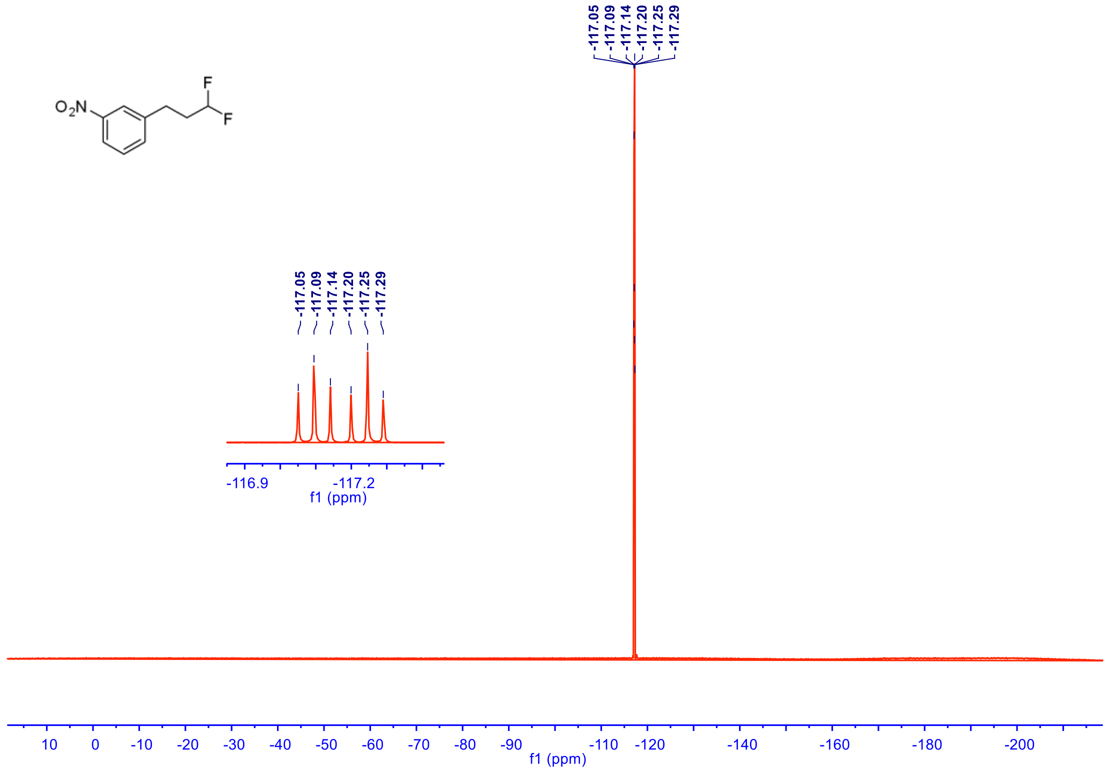

Supplementary Figure 197. ^{19}F NMR spectrum of compound **13** (376 MHz, $CDCl_3$)

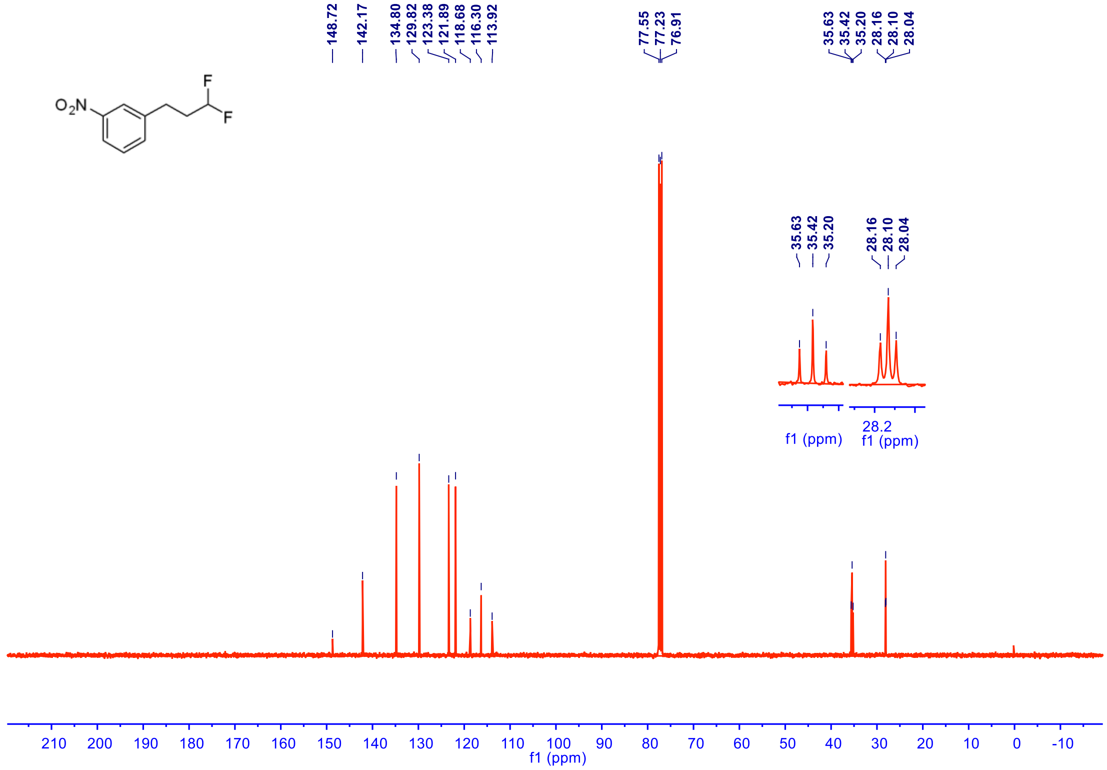

Supplementary Figure 198. ^{13}C NMR spectrum of compound **13** (101 MHz, $CDCl_3$)

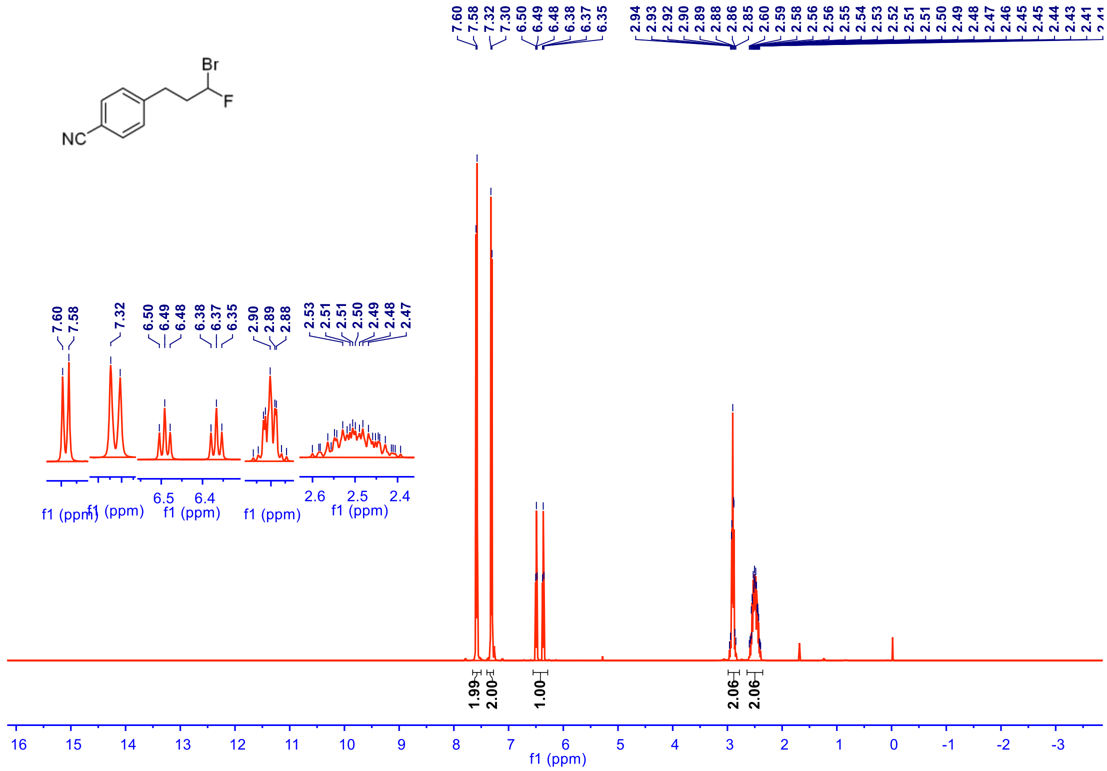

Supplementary Figure 199. ^{1}H NMR spectrum of **4-(3-bromo-3-fluoropropyl)benzonitrile 14a** (400 MHz, $CDCl_3$)

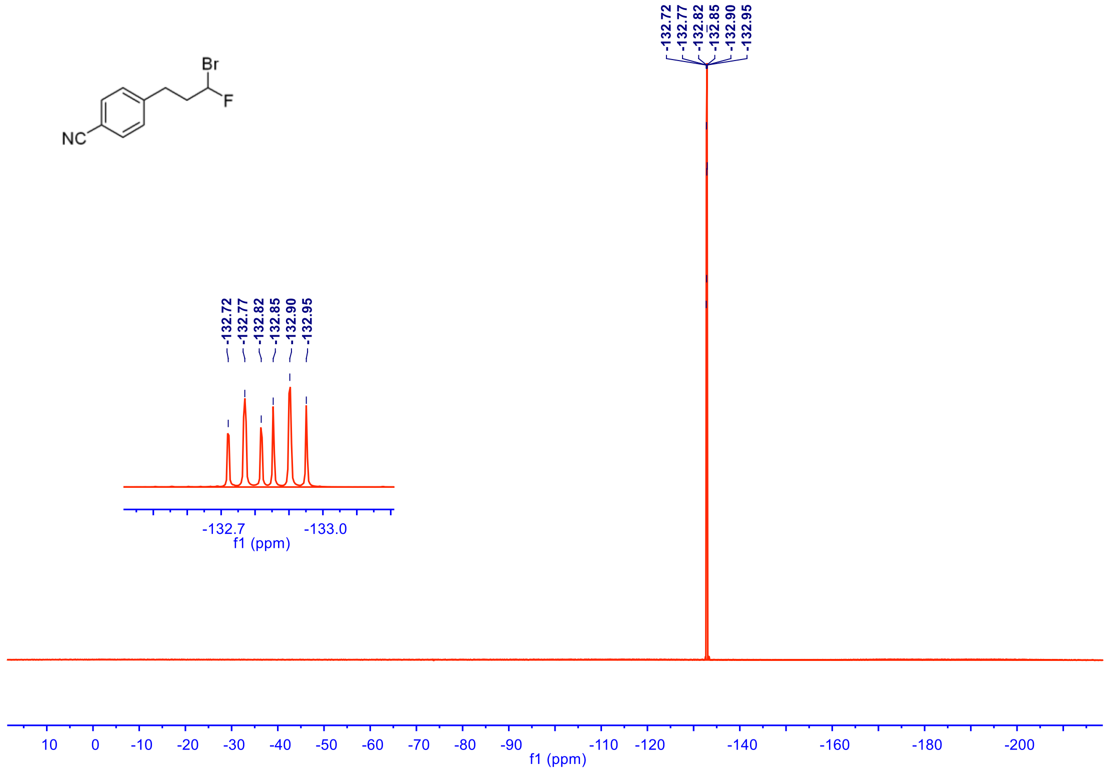

Supplementary Figure 200. ^{19}F NMR spectrum of **4-(3-bromo-3-fluoropropyl)benzonitrile 14a** (376 MHz, $CDCl_3$)

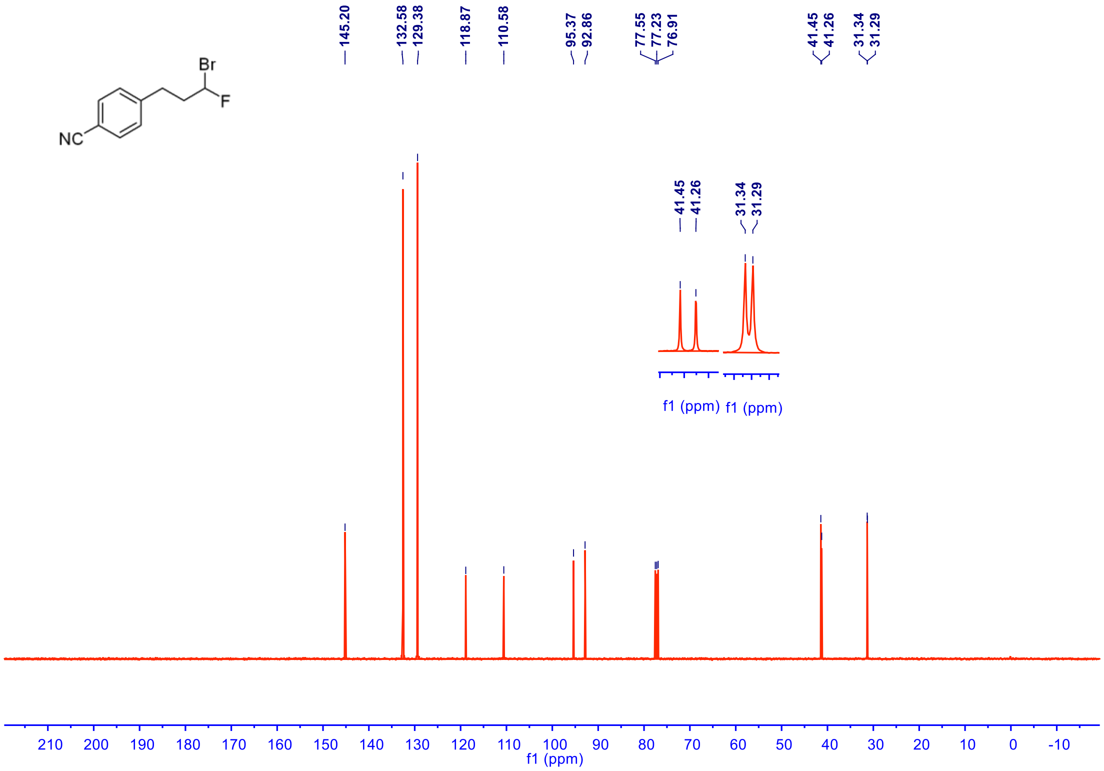

Supplementary Figure 201. ^{13}C NMR spectrum of **4-(3-bromo-3-fluoropropyl)benzonitrile 14a** (101 MHz, $CDCl_3$)

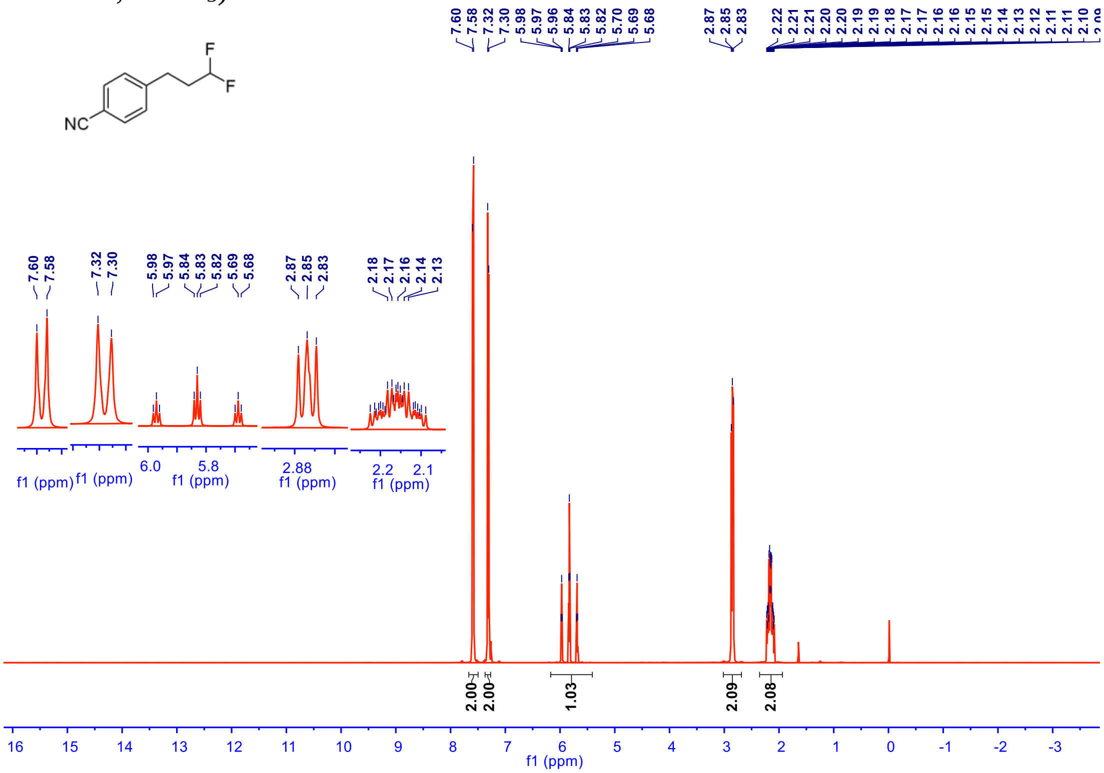

Supplementary Figure 202. ^{1}H NMR spectrum of compound **14** (400 MHz, $CDCl_3$)

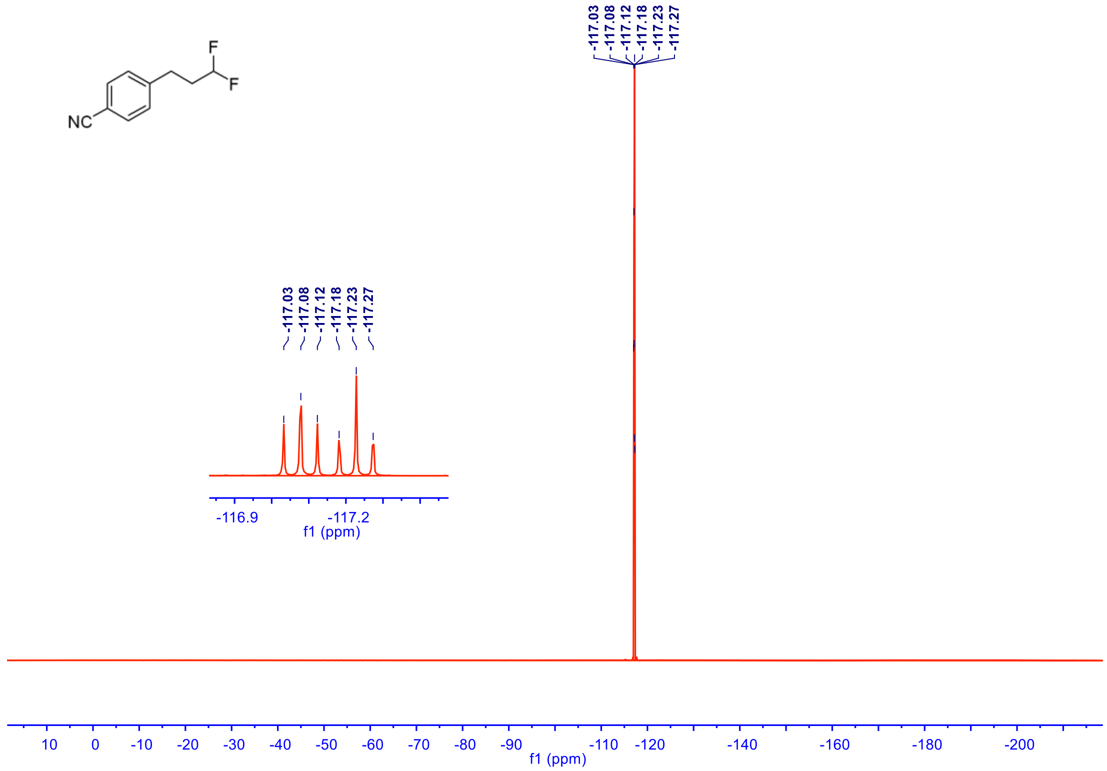

Supplementary Figure 203. ^{19}F NMR spectrum of compound **14** (376 MHz, $CDCl_3$)

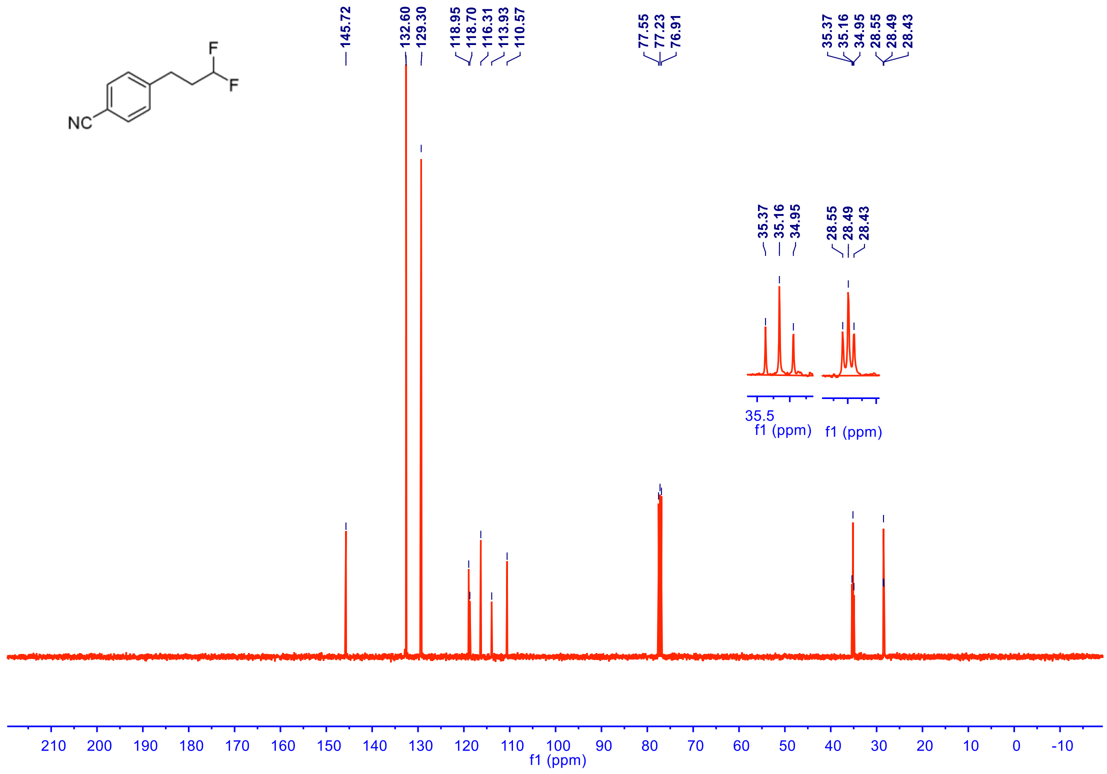

Supplementary Figure 204. ^{13}C NMR spectrum of compound **14** (101 MHz, $CDCl_3$)

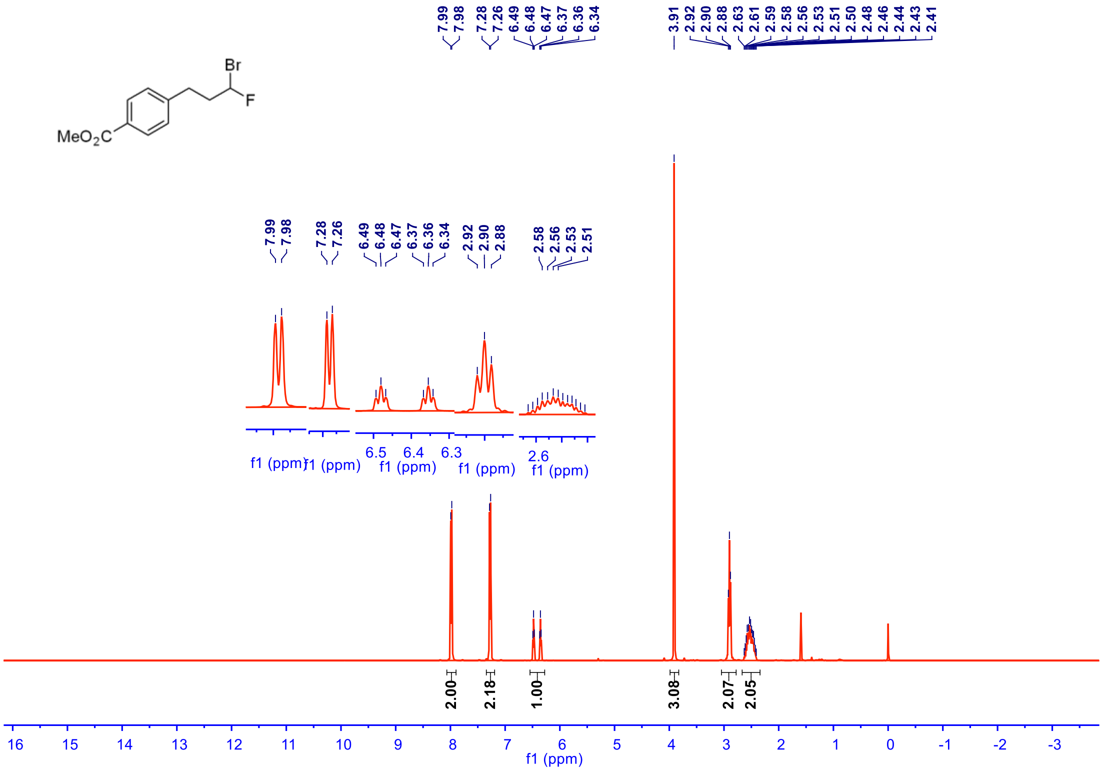

Supplementary Figure 205. ^{1}H NMR spectrum of **methyl 4-(3-bromo-3-fluoropropyl)benzoate 15a** (400 MHz, $CDCl_3$)

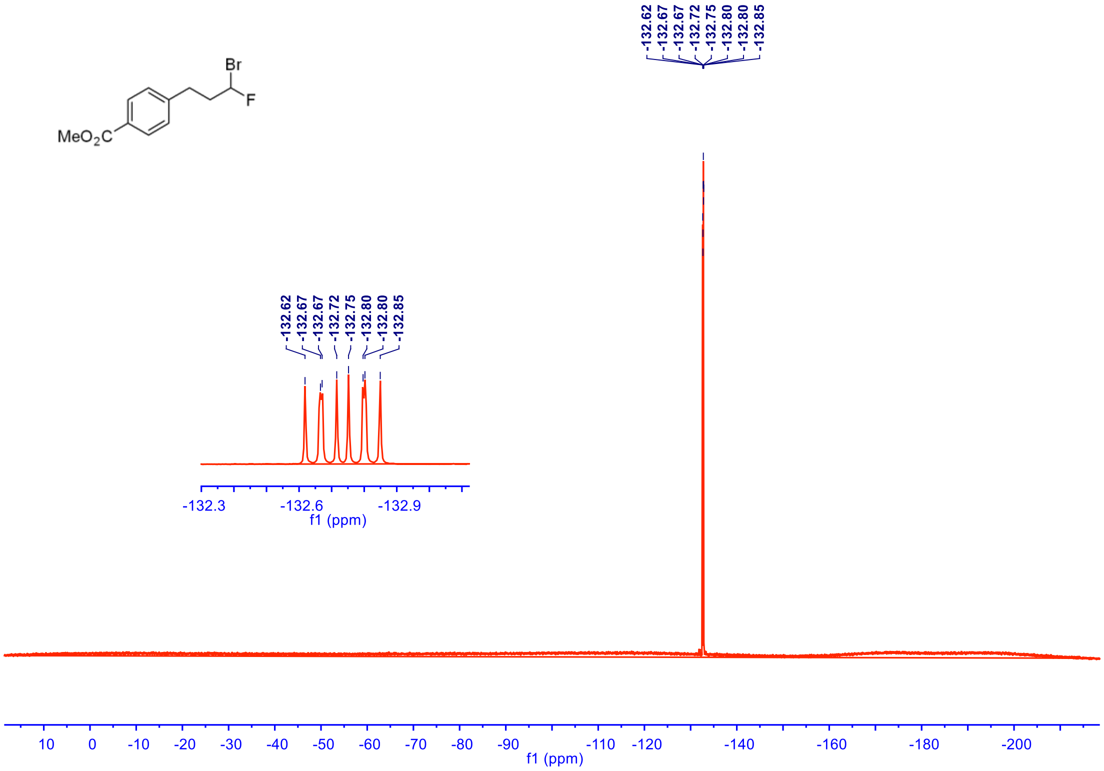

Supplementary Figure 206. ^{19}F NMR spectrum of **methyl 4-(3-bromo-3-fluoropropyl)benzoate 15a** (376 MHz, $CDCl_3$)

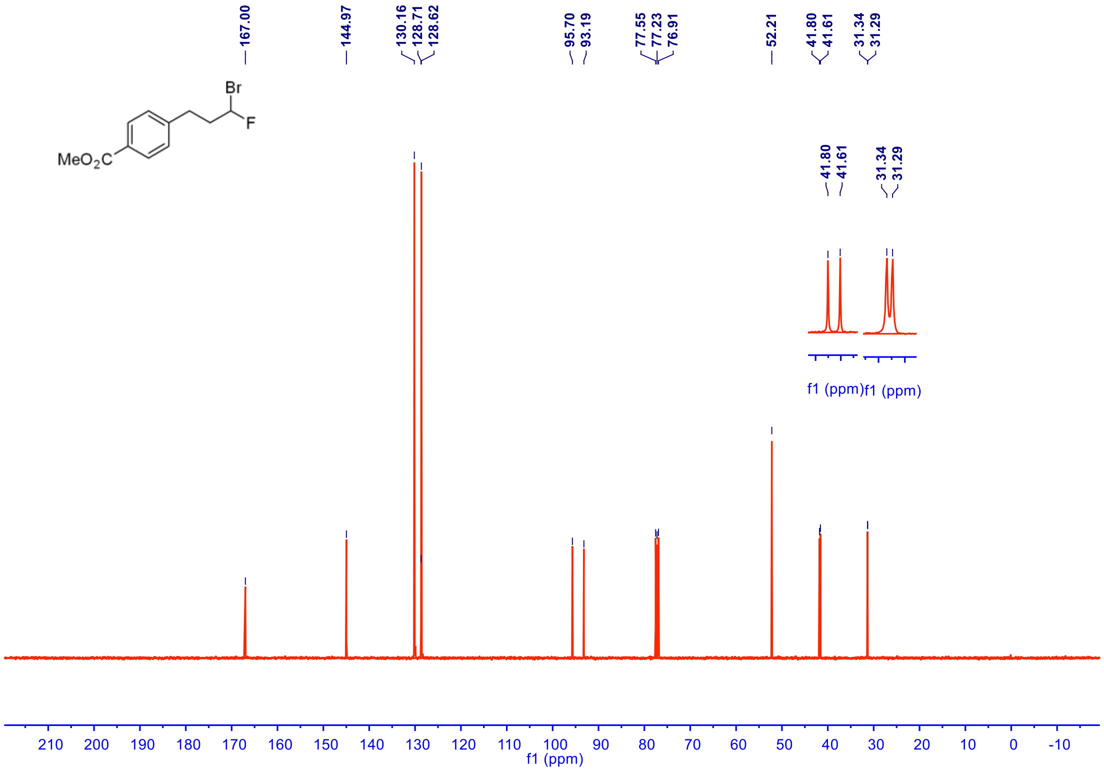

Supplementary Figure 207. ^{13}C NMR spectrum of **methyl 4-(3-bromo-3-fluoropropyl)benzoate 15a** (101 MHz, $CDCl_3$)

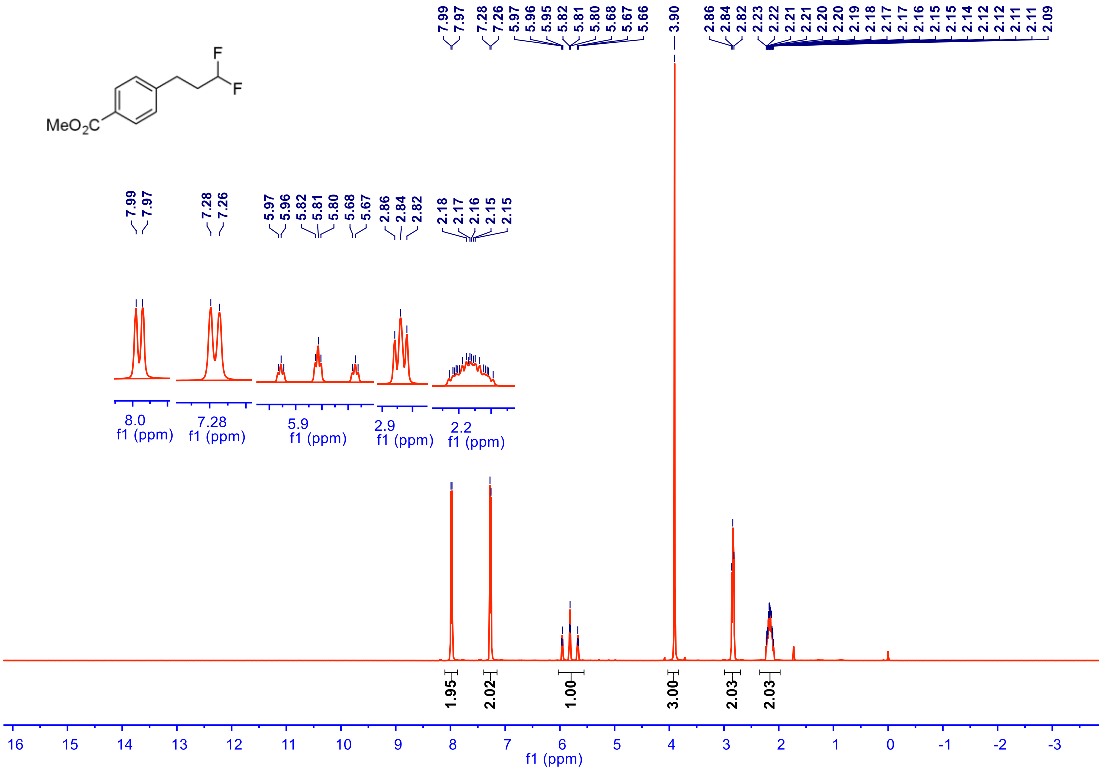

Supplementary Figure 208. ^{1}H NMR spectrum of compound **15** (400 MHz, $CDCl_3$)

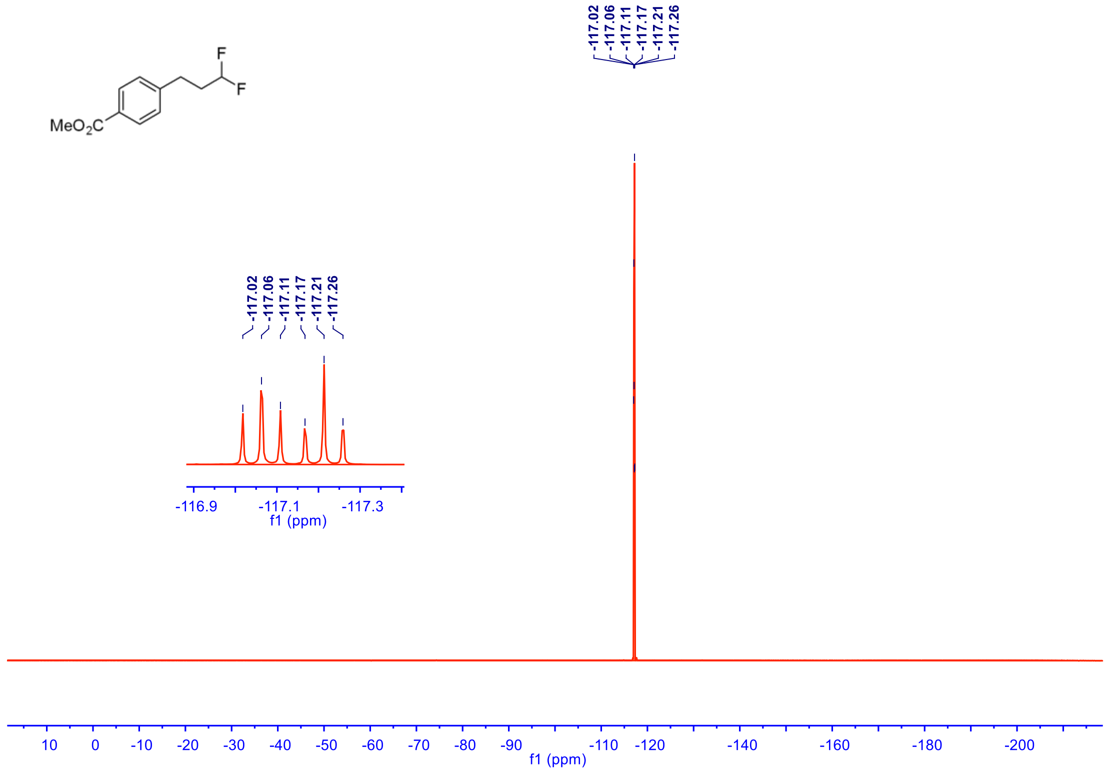

Supplementary Figure 209. ^{19}F NMR spectrum of compound **15** (376 MHz, $CDCl_3$)

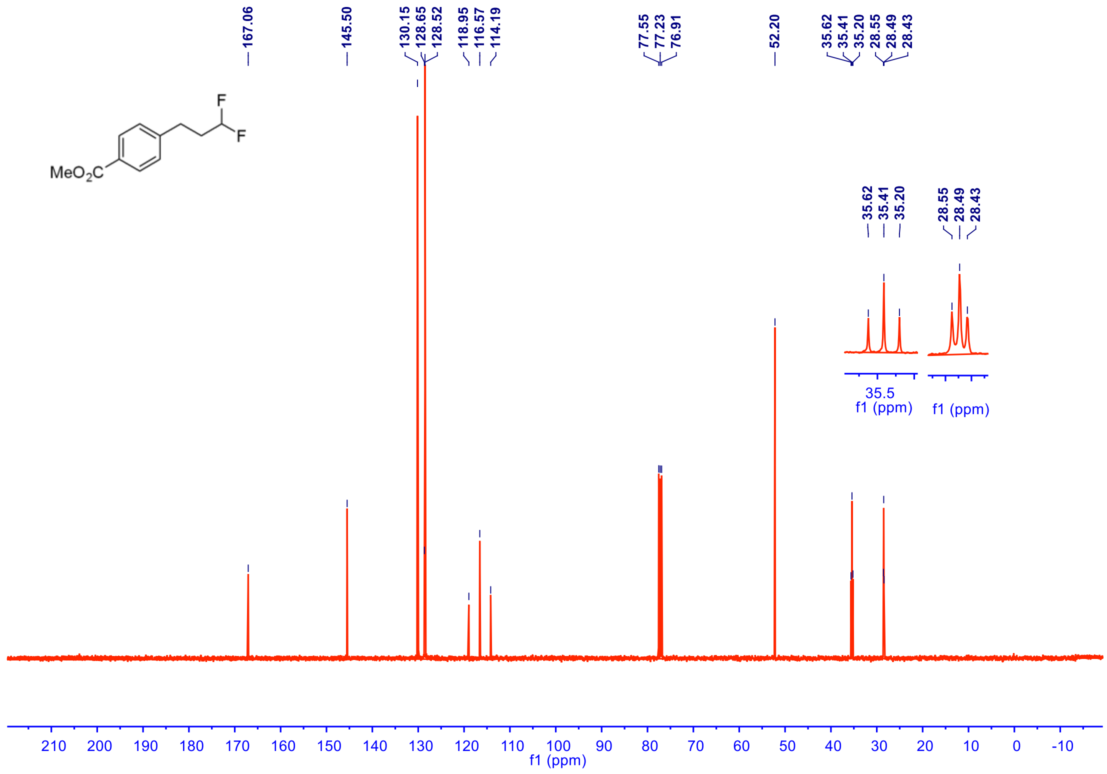

Supplementary Figure 210. ^{13}C NMR spectrum of compound **15** (101 MHz, $CDCl_3$)

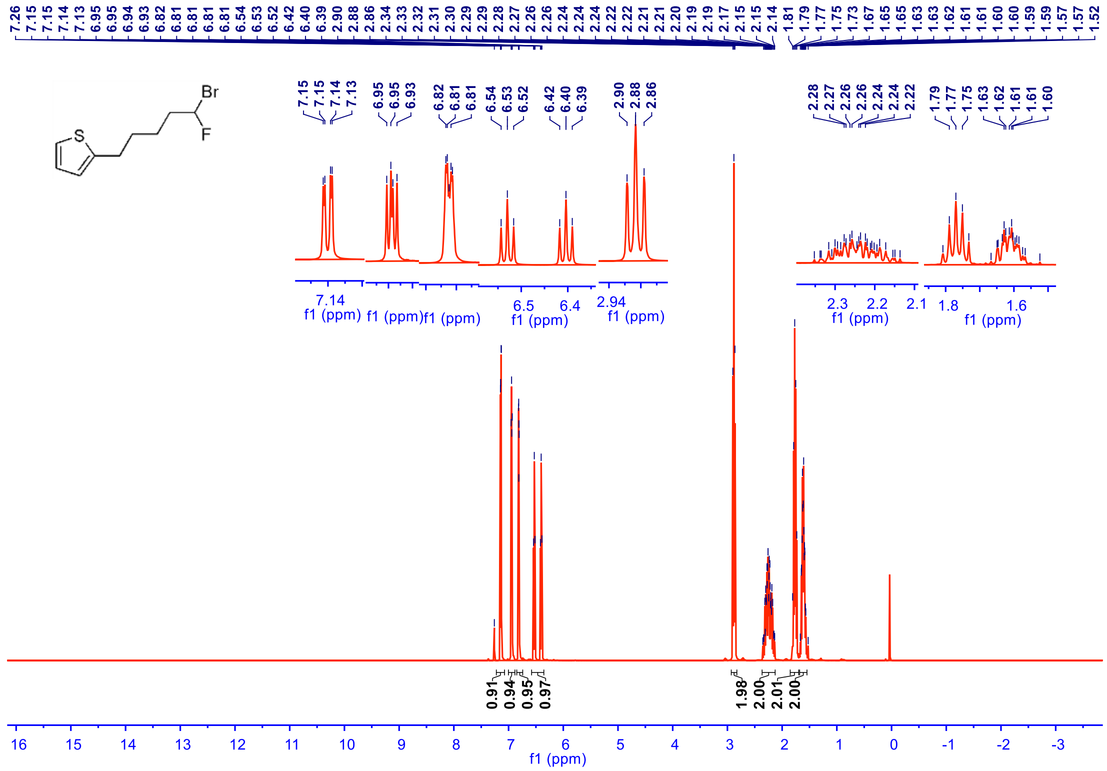

Supplementary Figure 211. ^{1}H NMR spectrum of **2-(5-bromo-5-fluoropentyl)thiophene 16a** (400 MHz, $CDCl_3$)

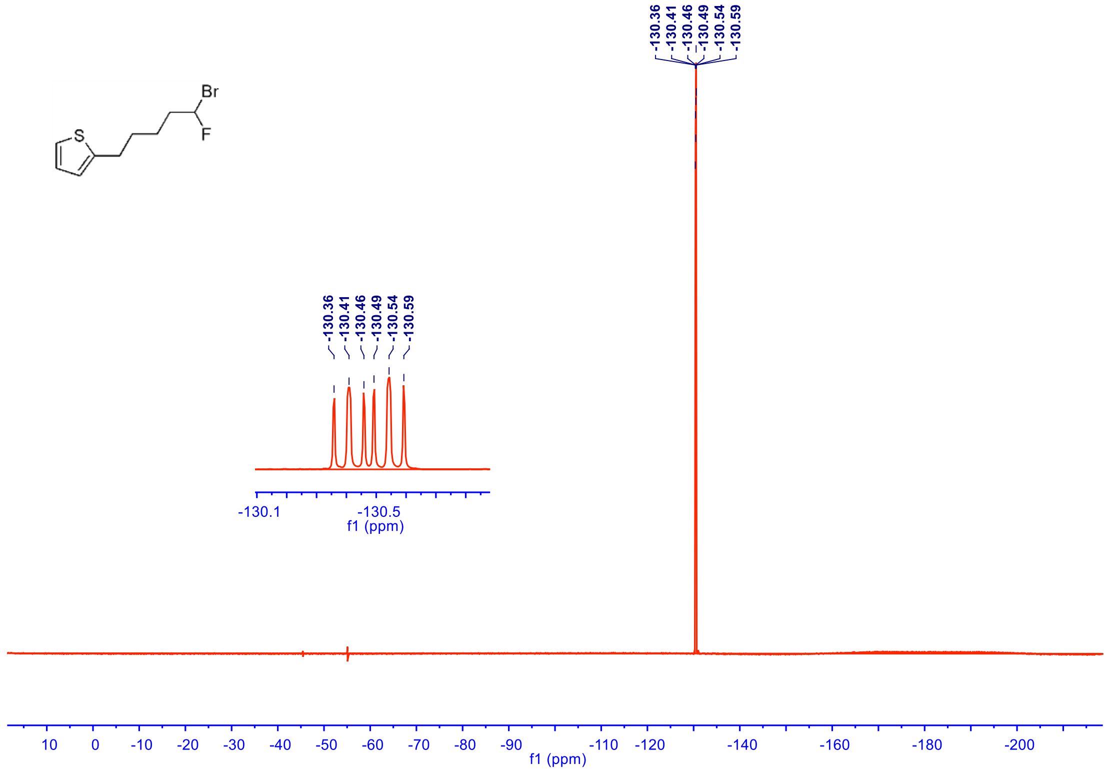

Supplementary Figure 212. ^{19}F NMR spectrum of **2-(5-bromo-5-fluoropentyl)thiophene 16a** (376 MHz, $CDCl_3$)

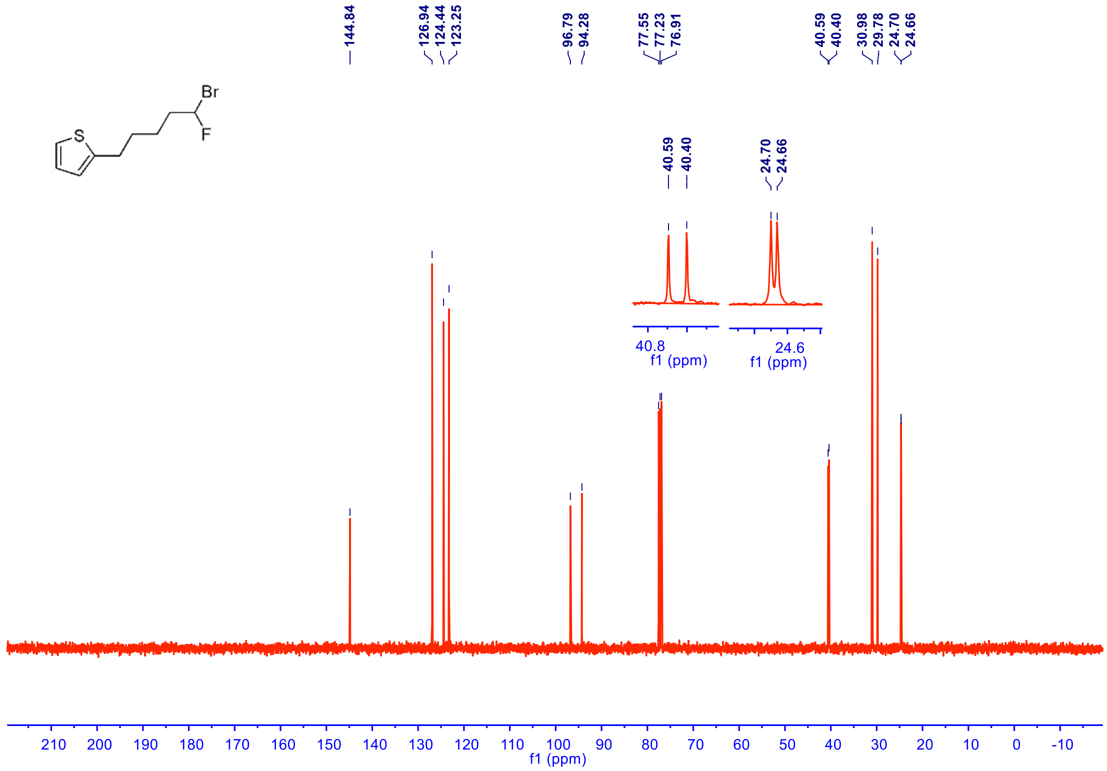

Supplementary Figure 213. ^{13}C NMR spectrum of **2-(5-bromo-5-fluoropentyl)thiophene 16a** (101 MHz, $CDCl_3$)

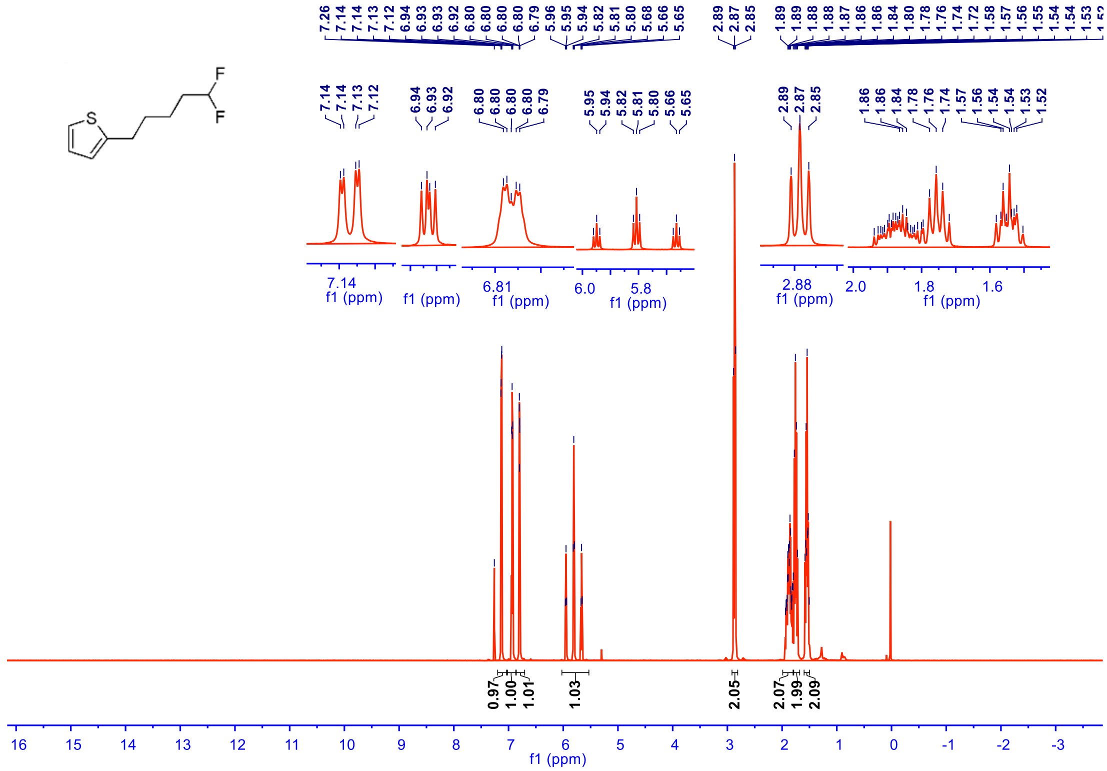

Supplementary Figure 214. ^{1}H NMR spectrum of compound **16** (400 MHz, $CDCl_3$)

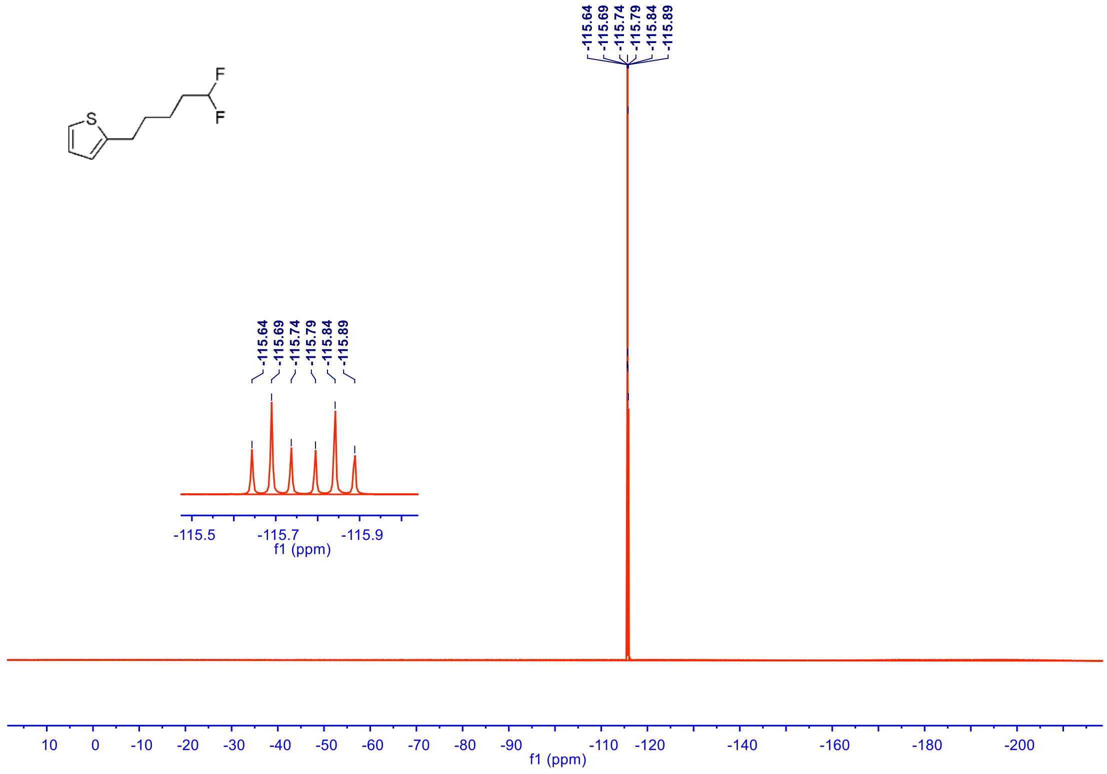

Supplementary Figure 215. ^{19}F NMR spectrum of compound **16** (376 MHz, $CDCl_3$)

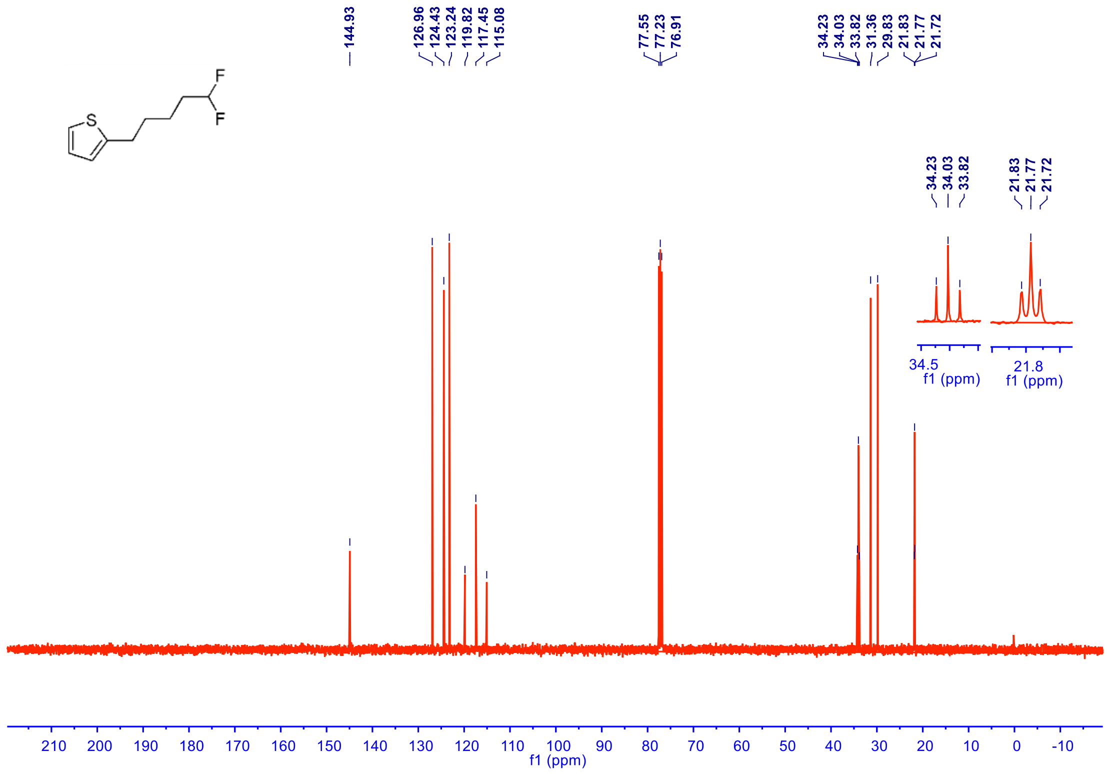

Supplementary Figure 216. ^{13}C NMR spectrum of compound **16** (101 MHz, $CDCl_3$)

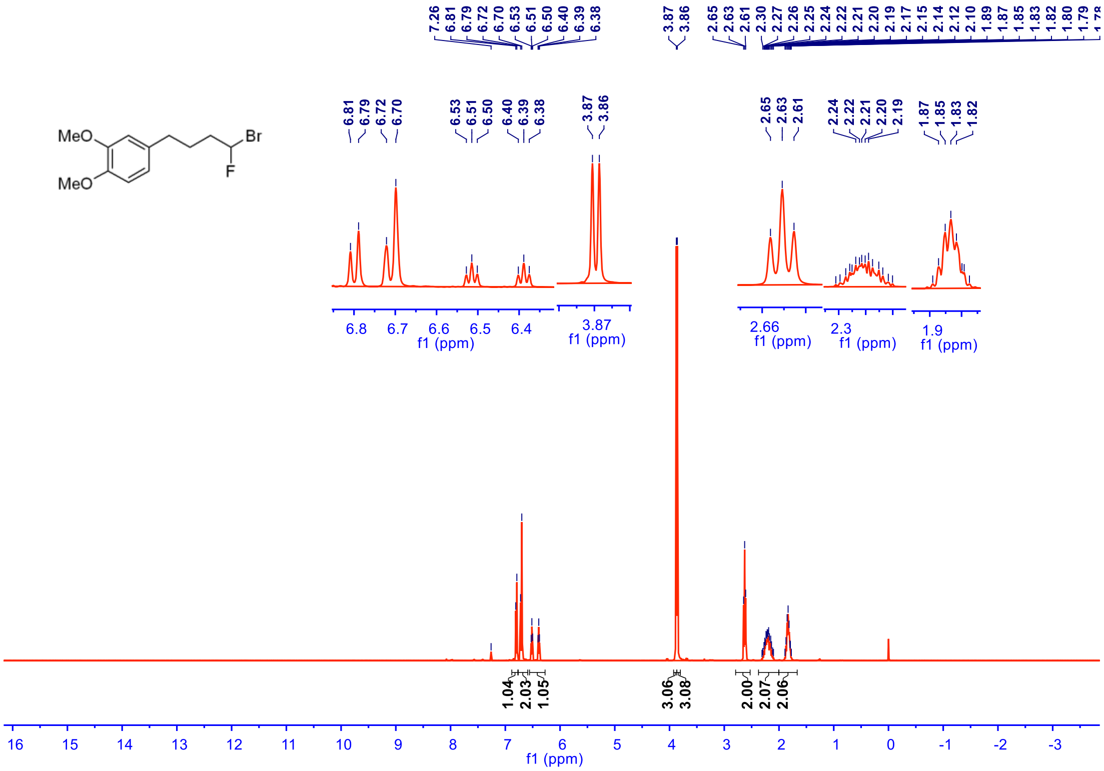

Supplementary Figure 217. ^{1}H NMR spectrum of **4-(4-bromo-4-fluorobutyl)-1,2-dimethoxybenzene 17a** (400 MHz, $CDCl_3$)

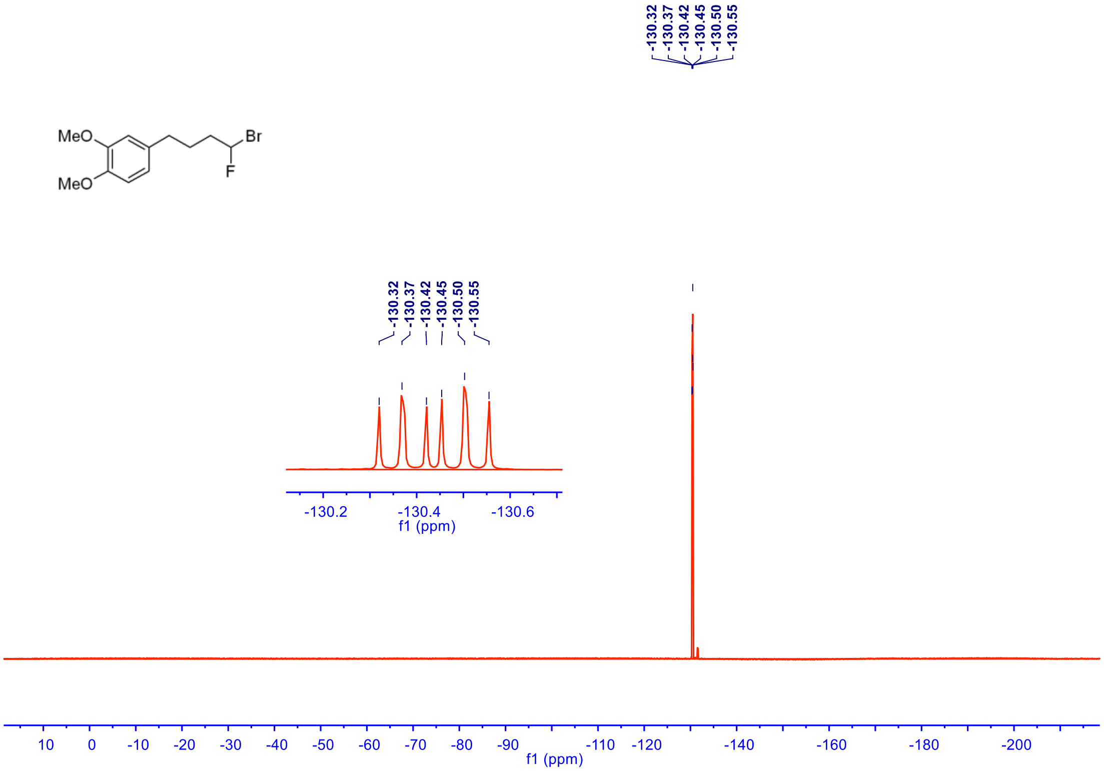

Supplementary Figure 218. ^{19}F NMR spectrum of **4-(4-bromo-4-fluorobutyl)-1,2-dimethoxybenzene 17a** (376 MHz, $CDCl_3$)

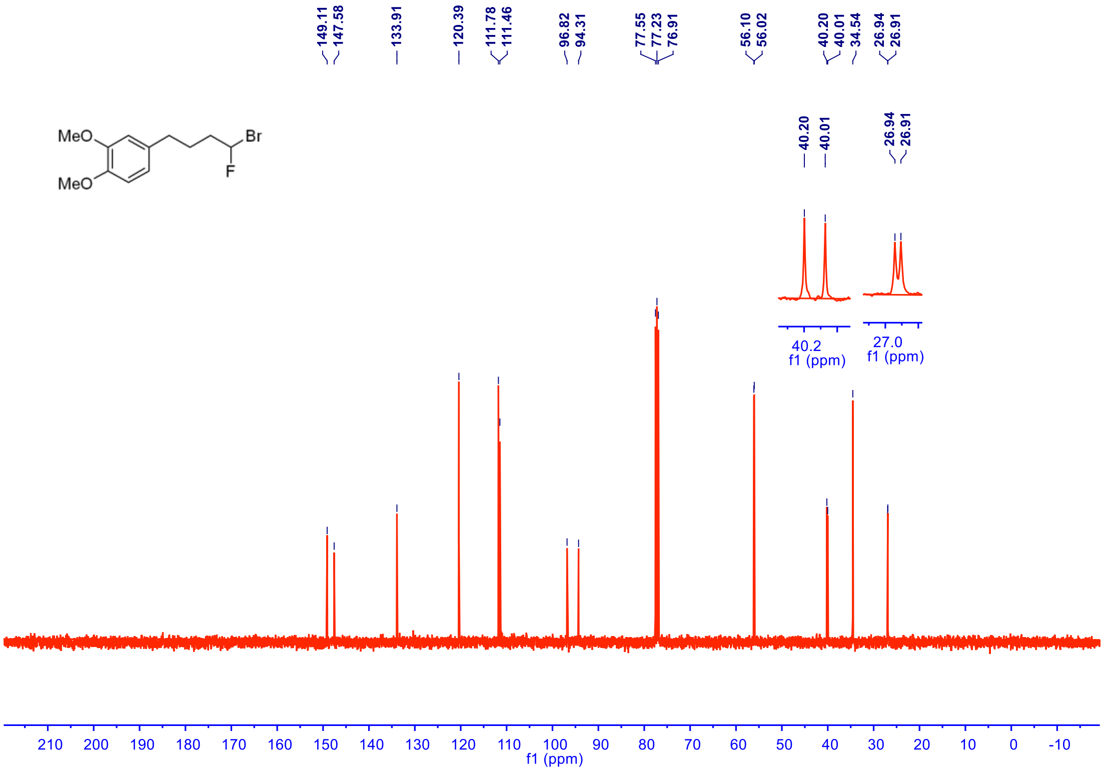

Supplementary Figure 219. ^{13}C NMR spectrum of **4-(4-bromo-4-fluorobutyl)-1,2-dimethoxybenzene 17a** (101 MHz, $CDCl_3$)

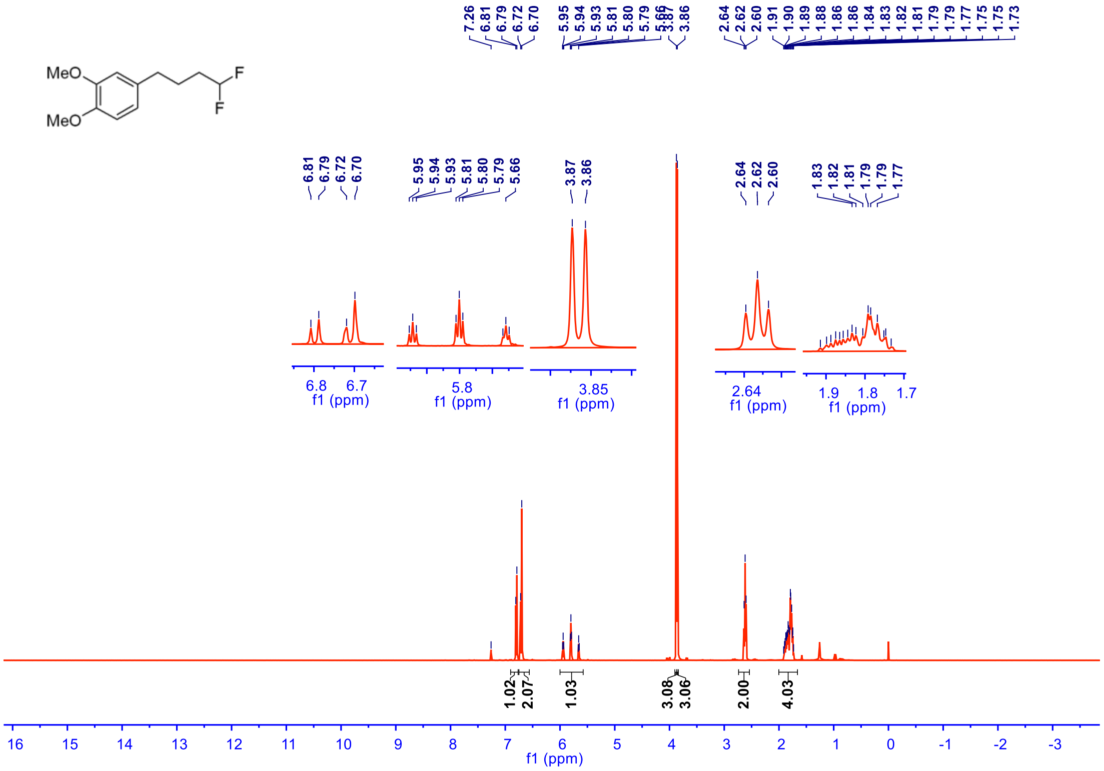

Supplementary Figure 220. ^{1}H NMR spectrum of compound **17** (400 MHz, $CDCl_3$)

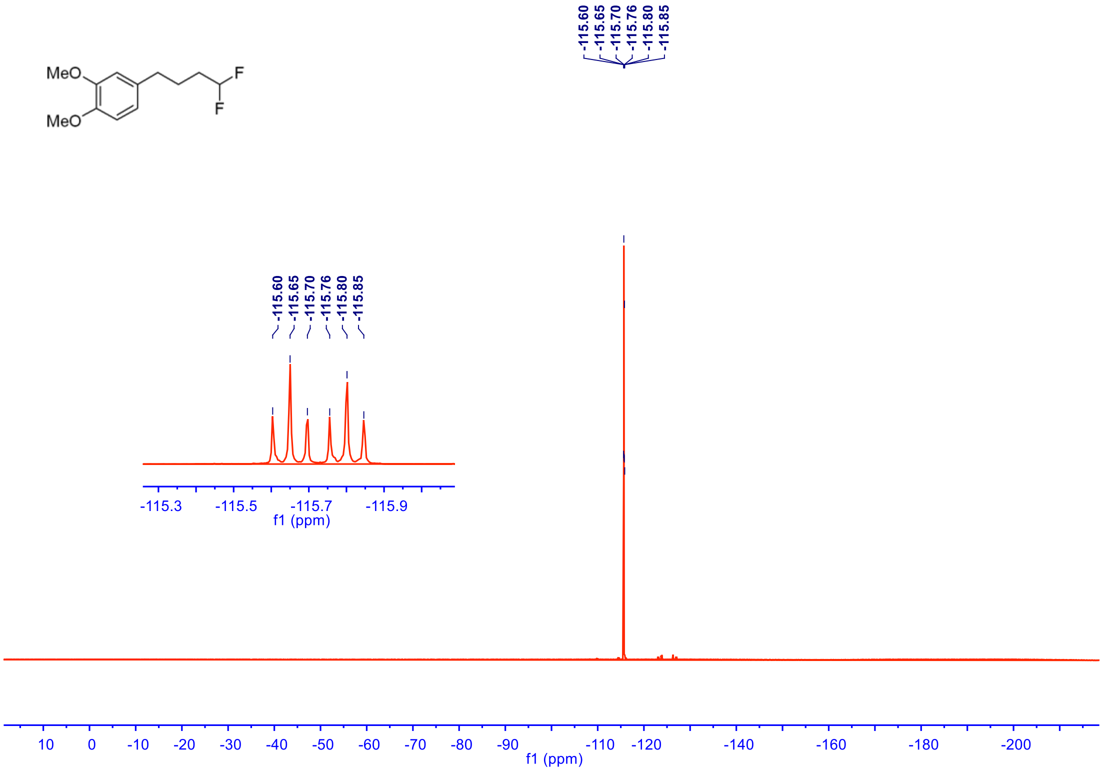

Supplementary Figure 221. ^{19}F NMR spectrum of compound **17** (376 MHz, $CDCl_3$)

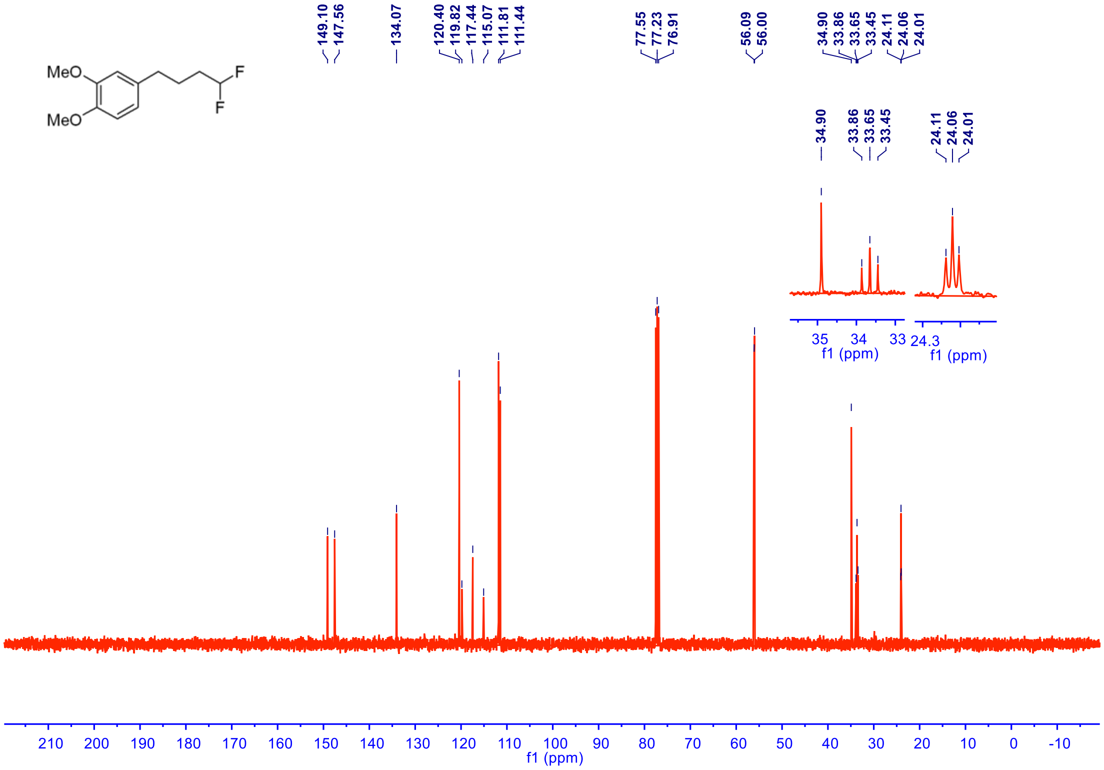

Supplementary Figure 222. ^{13}C NMR spectrum of compound **17** (101 MHz, $CDCl_3$)

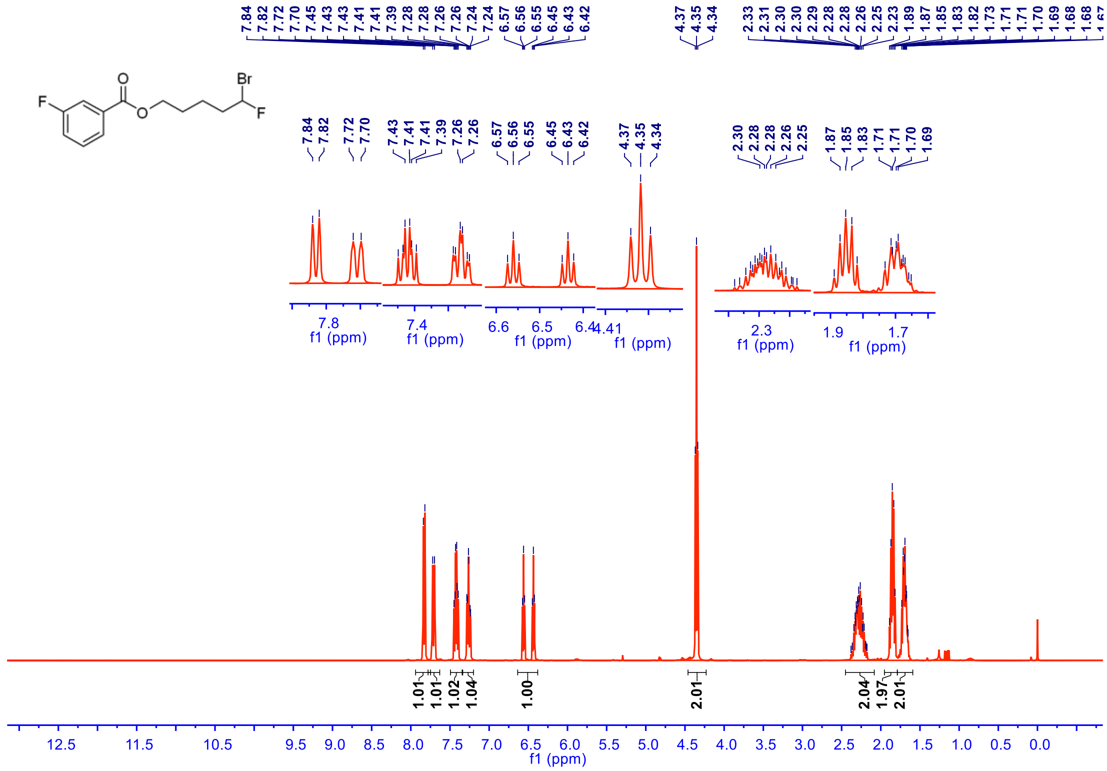

Supplementary Figure 223. ^{1}H NMR spectrum of **5-bromo-5-fluoropentyl 3-fluorobenzoate 18a** (400 MHz, $CDCl_3$)

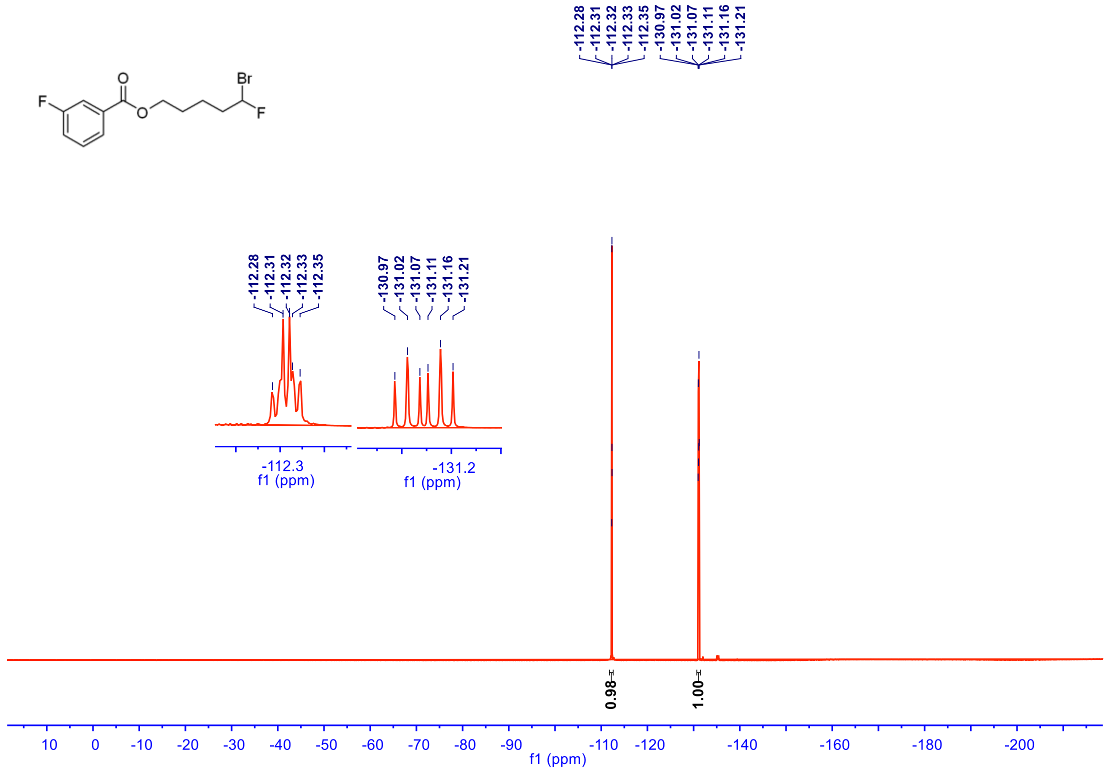

Supplementary Figure 224. ^{19}F NMR spectrum of **5-bromo-5-fluoropentyl 3-fluorobenzoate 18a** (376 MHz, $CDCl_3$)

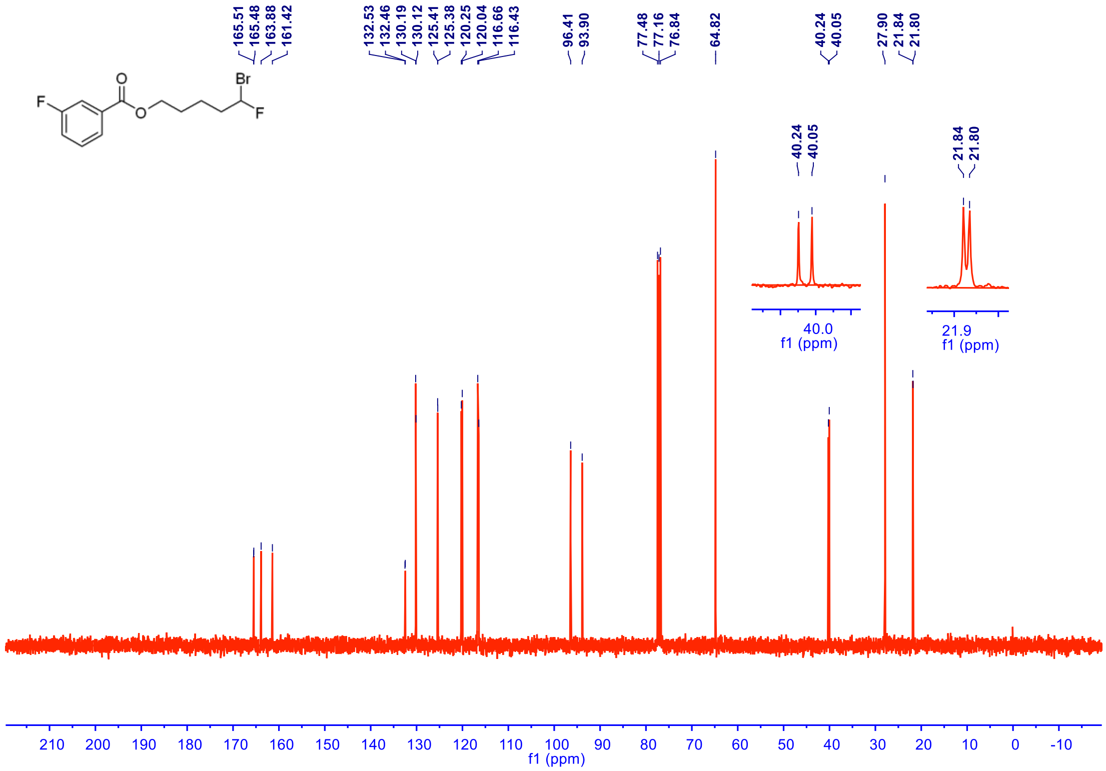

Supplementary Figure 225. ^{13}C NMR spectrum of **5-bromo-5-fluoropentyl 3-fluorobenzoate 18a** (101 MHz, $CDCl_3$)

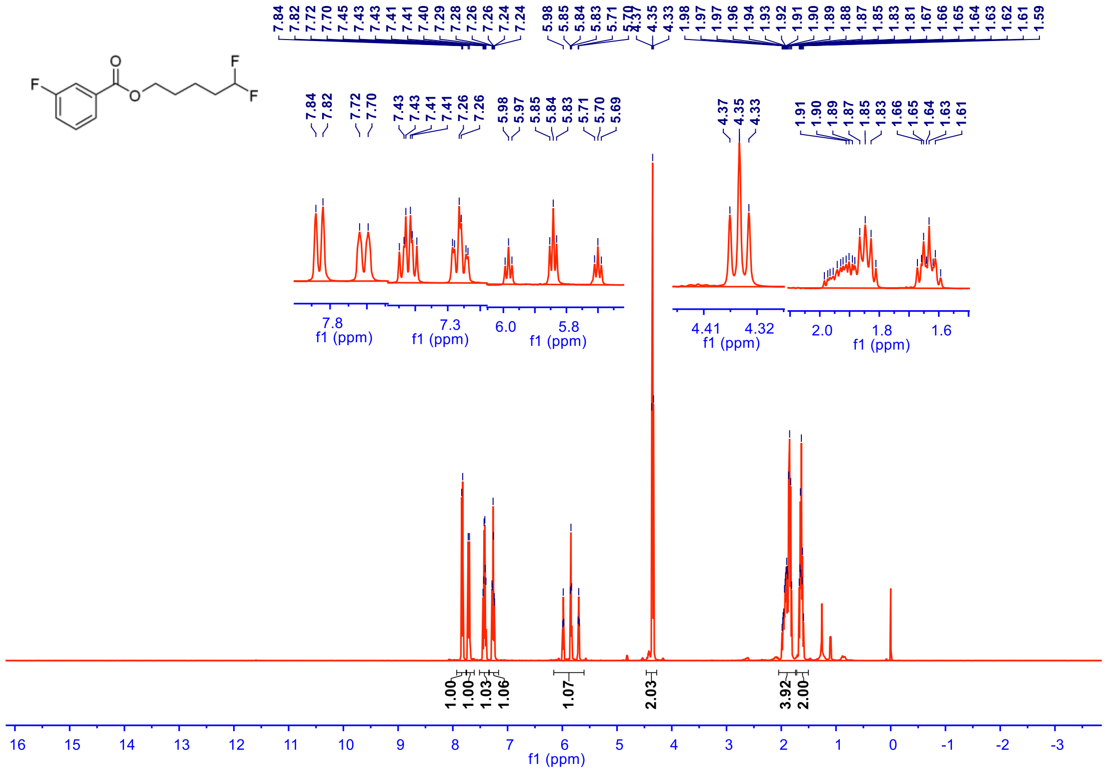

Supplementary Figure 226. ^{1}H NMR spectrum of compound **18** (400 MHz, $CDCl_3$)

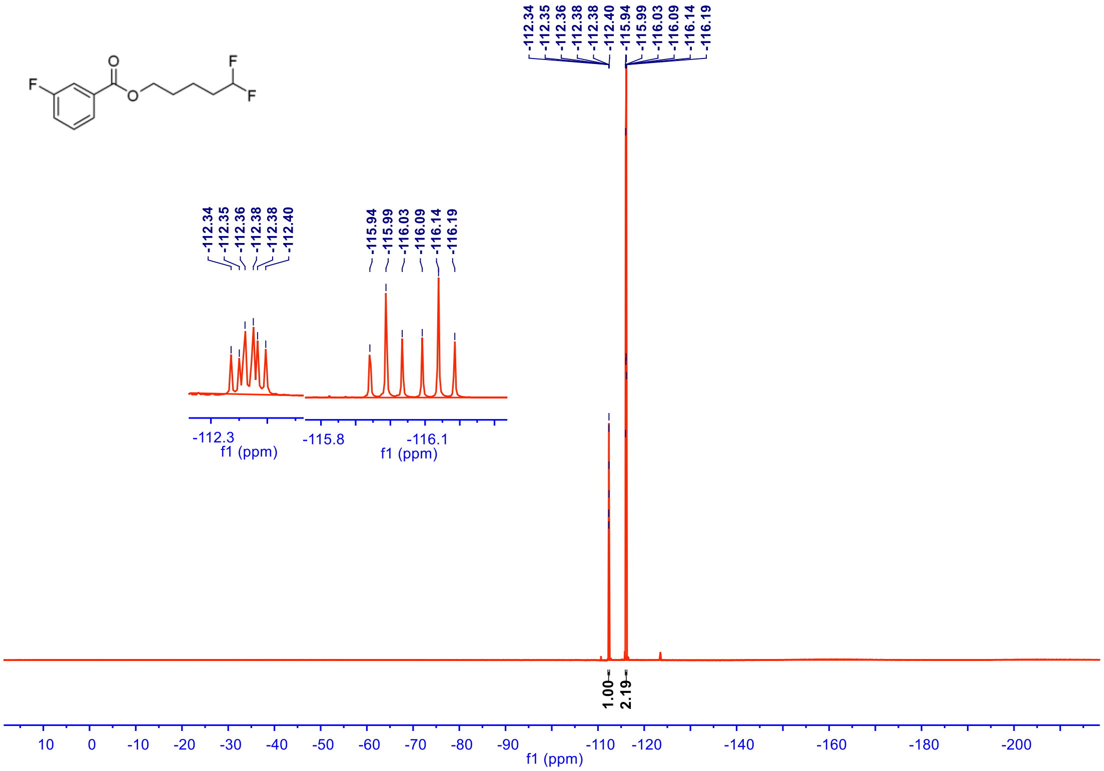

Supplementary Figure 227. ^{19}F NMR spectrum of compound **18** (376 MHz, $CDCl_3$)

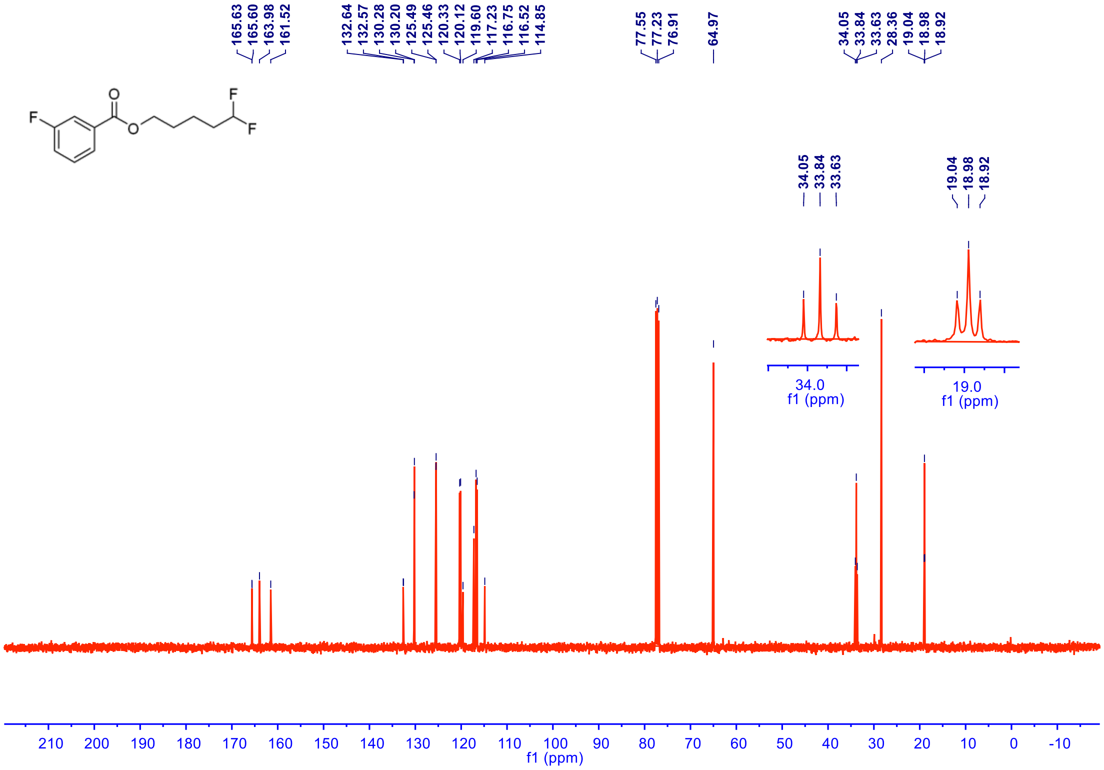

Supplementary Figure 228. ^{13}C NMR spectrum of compound **18** (101 MHz, $CDCl_3$)

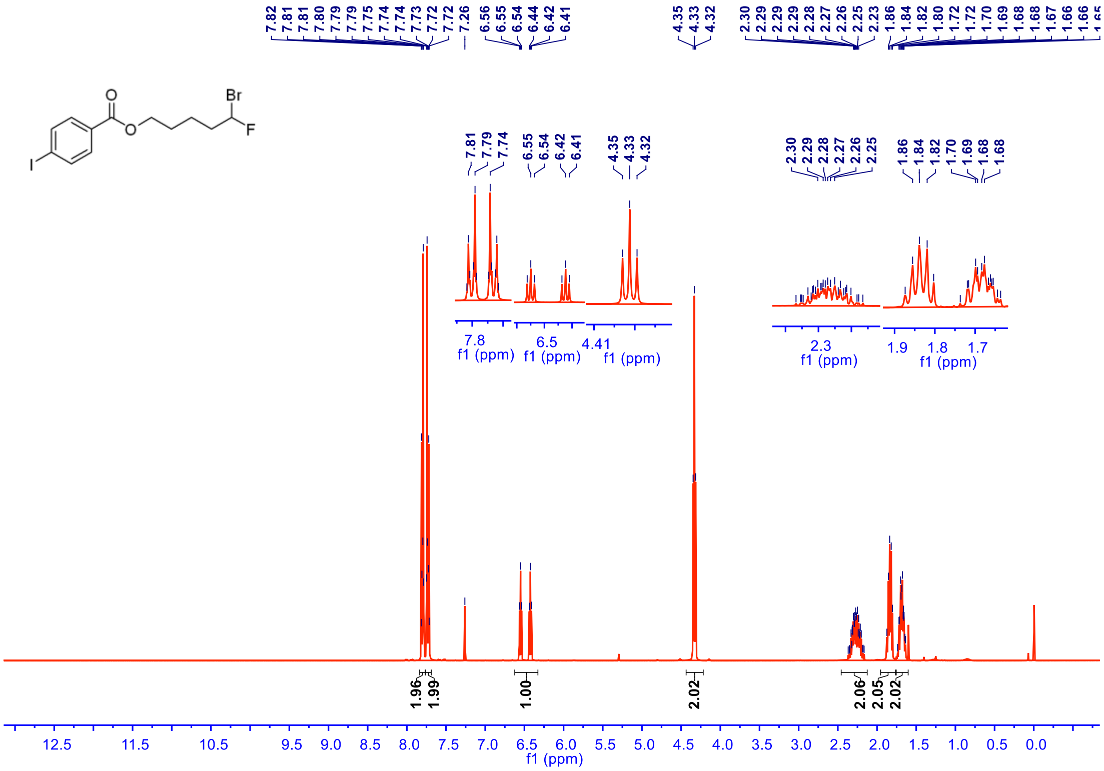

Supplementary Figure 229. ^{1}H NMR spectrum of **5-bromo-5-fluoropentyl 4-iodobenzoate 19a** (400 MHz, $CDCl_3$)

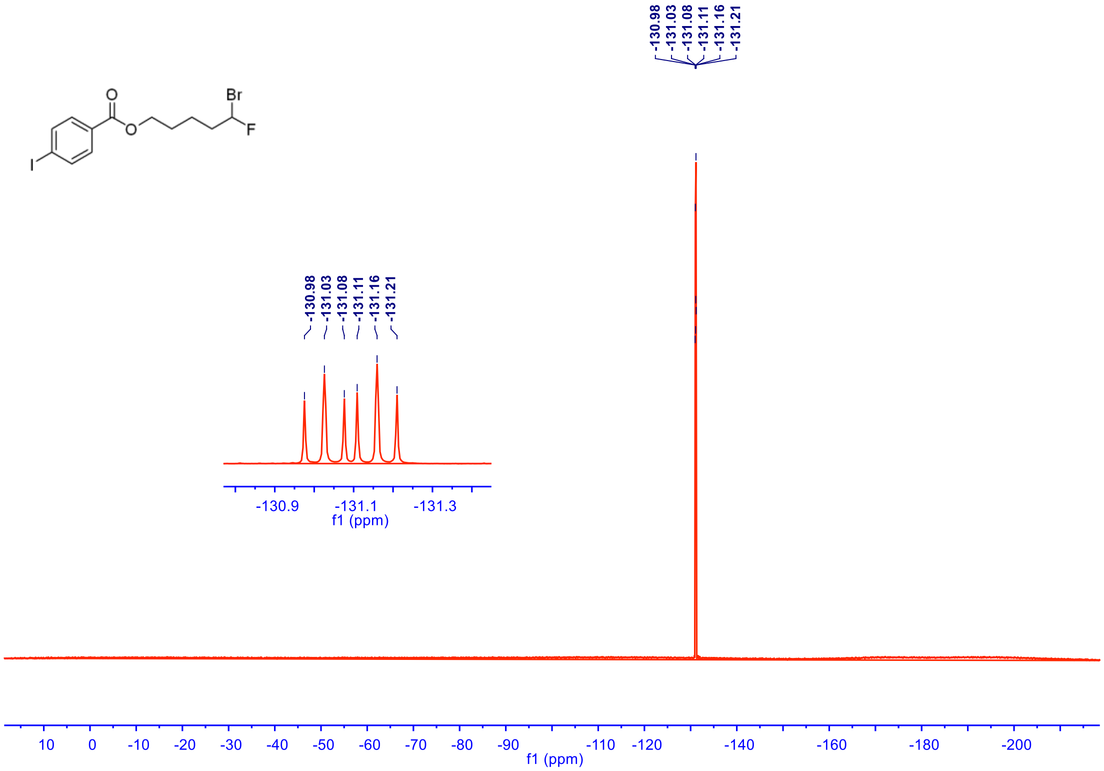

Supplementary Figure 230. ^{19}F NMR spectrum of compound **5-bromo-5-fluoropentyl 4-iodobenzoate 19a** (376 MHz, $CDCl_3$)

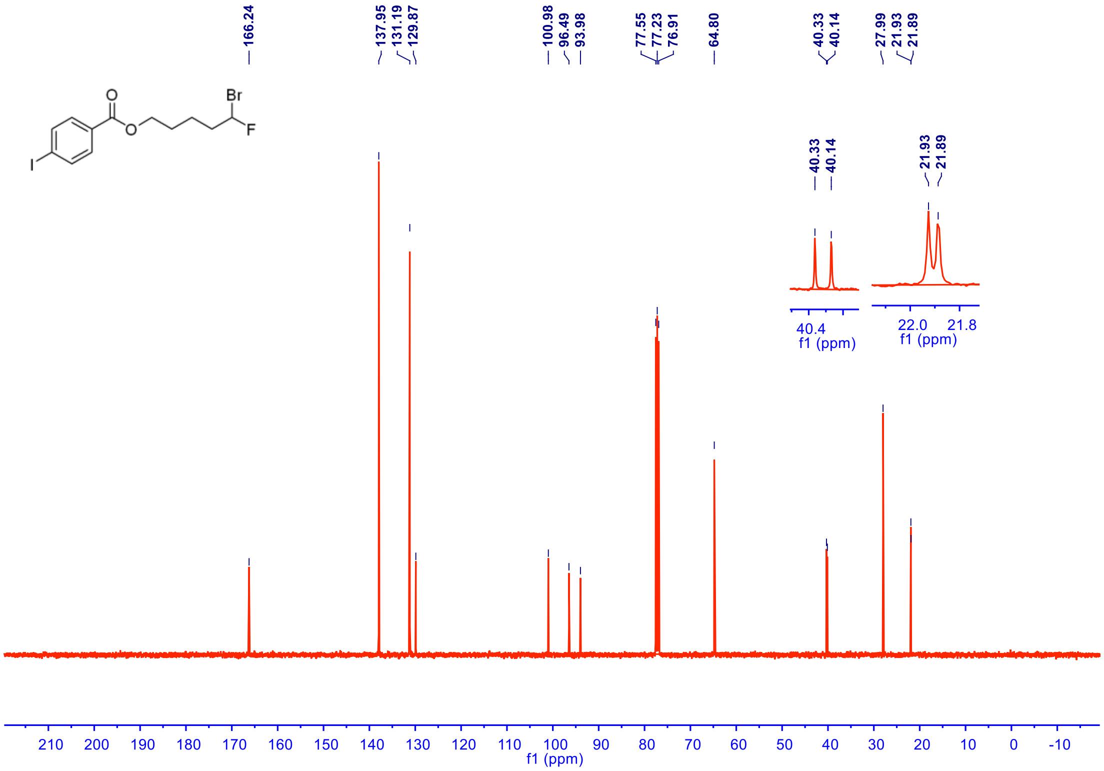

Supplementary Figure 231. ^{13}C NMR spectrum of **5-bromo-5-fluoropentyl 4-iodobenzoate 19a** (101 MHz, $CDCl_3$)

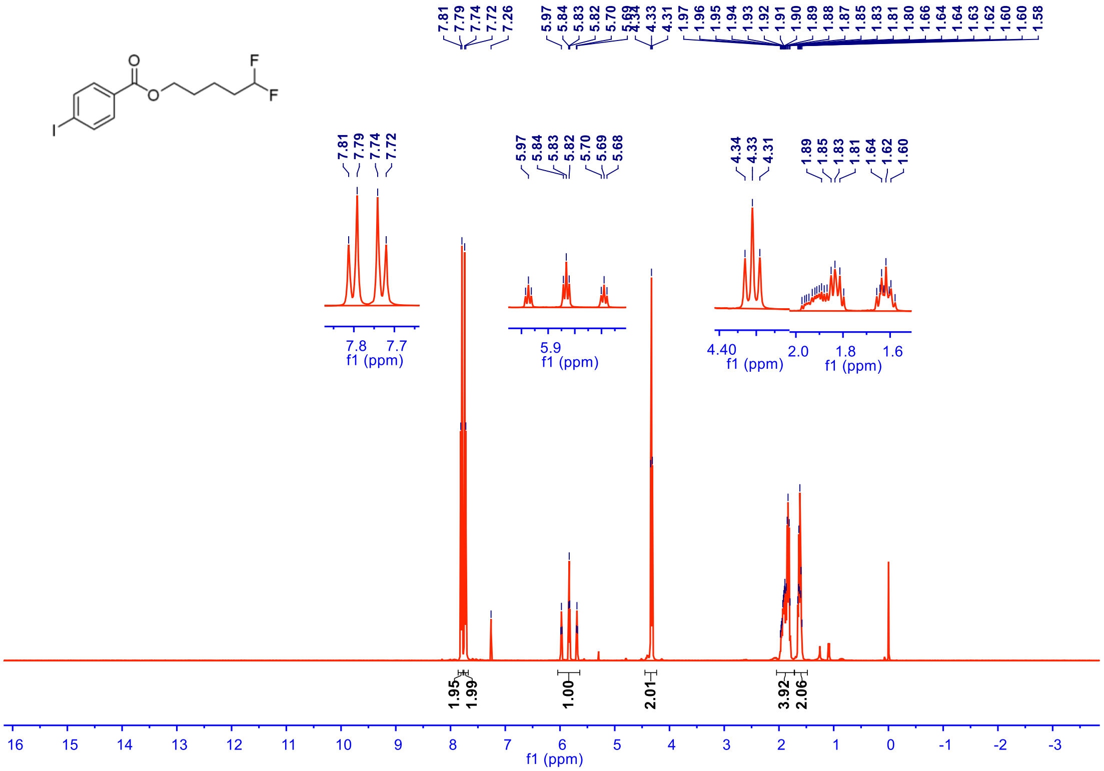

Supplementary Figure 232. ^{1}H NMR spectrum of compound **19** (400 MHz, $CDCl_3$)

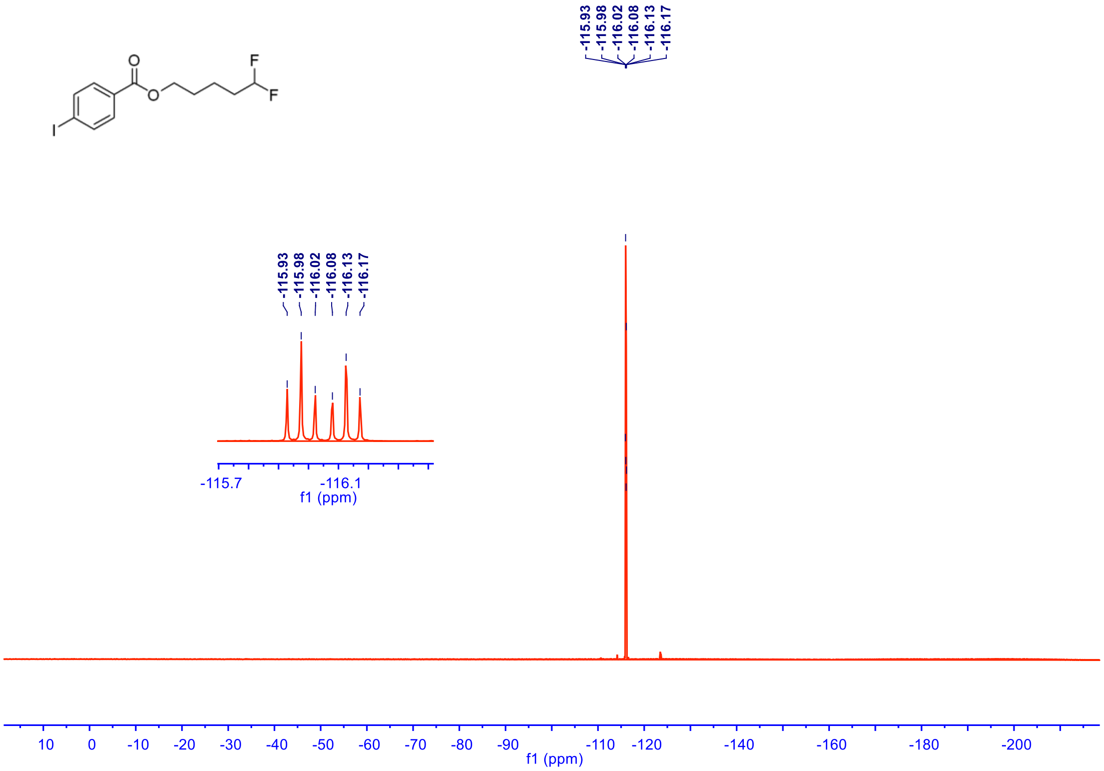

Supplementary Figure 233. ^{19}F NMR spectrum of compound **19** (376 MHz, $CDCl_3$)

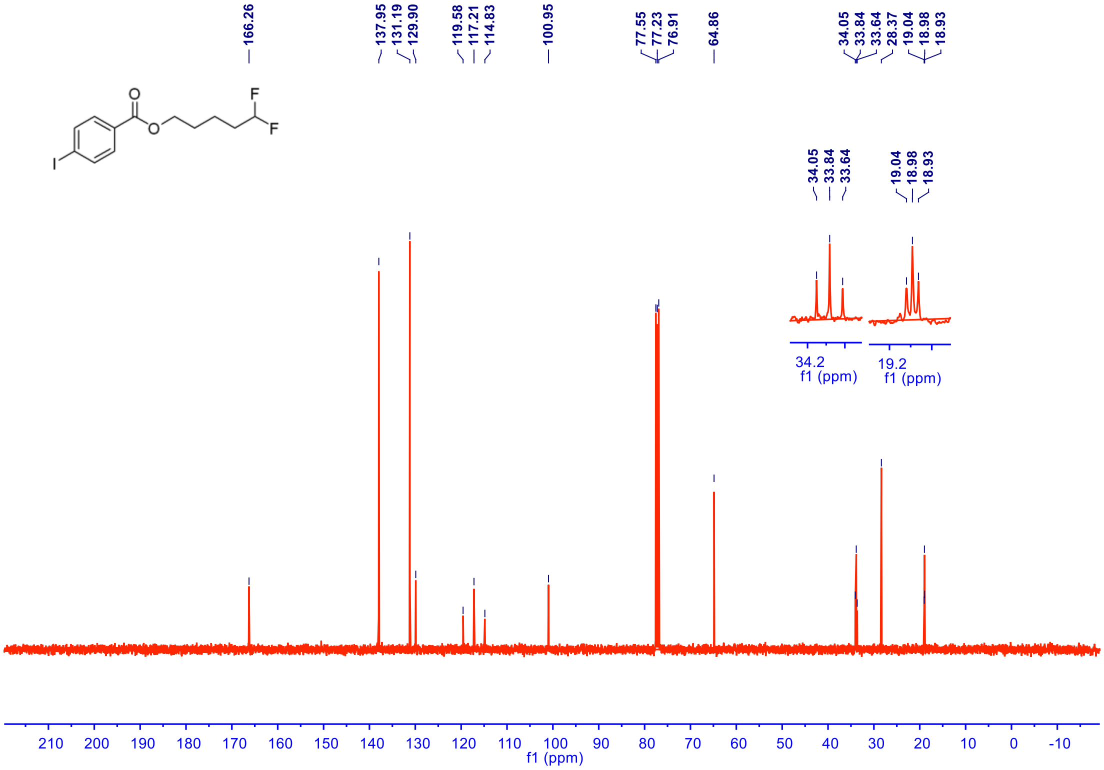

Supplementary Figure 234. ^{13}C NMR spectrum of compound **19** (101 MHz, $CDCl_3$)

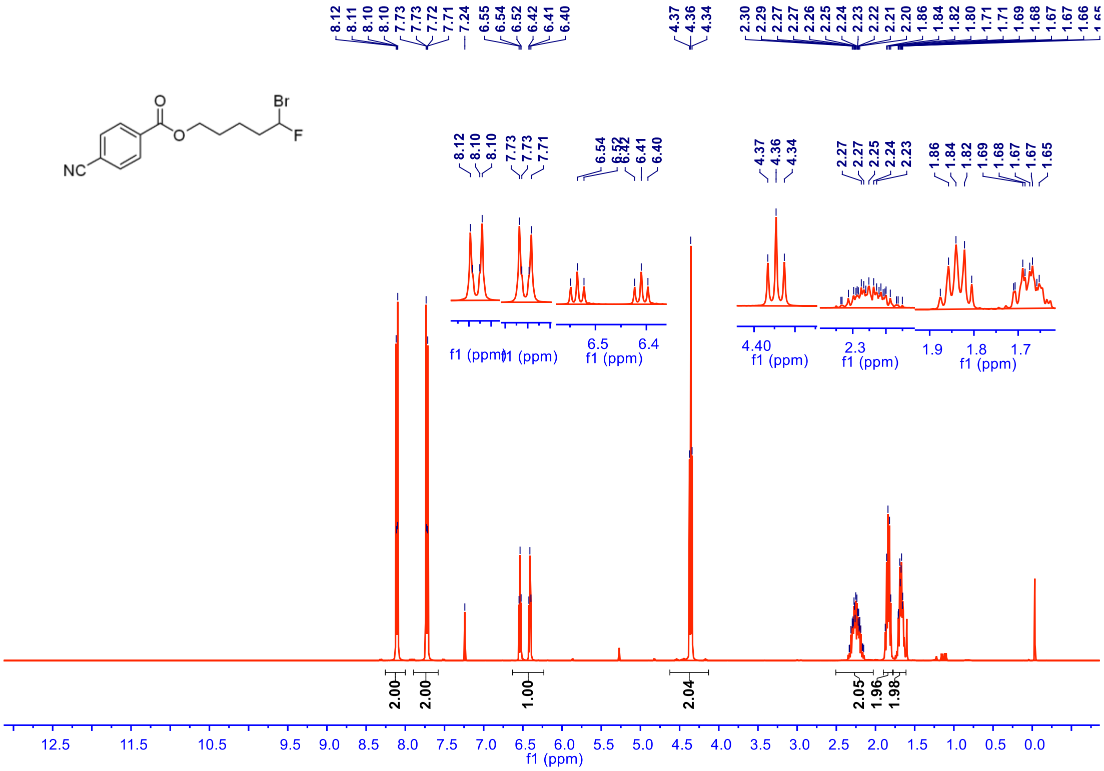

Supplementary Figure 235. ^{1}H NMR spectrum of **5-bromo-5-fluoropentyl 4-cyanobenzoate 20a** (400 MHz, $CDCl_3$)

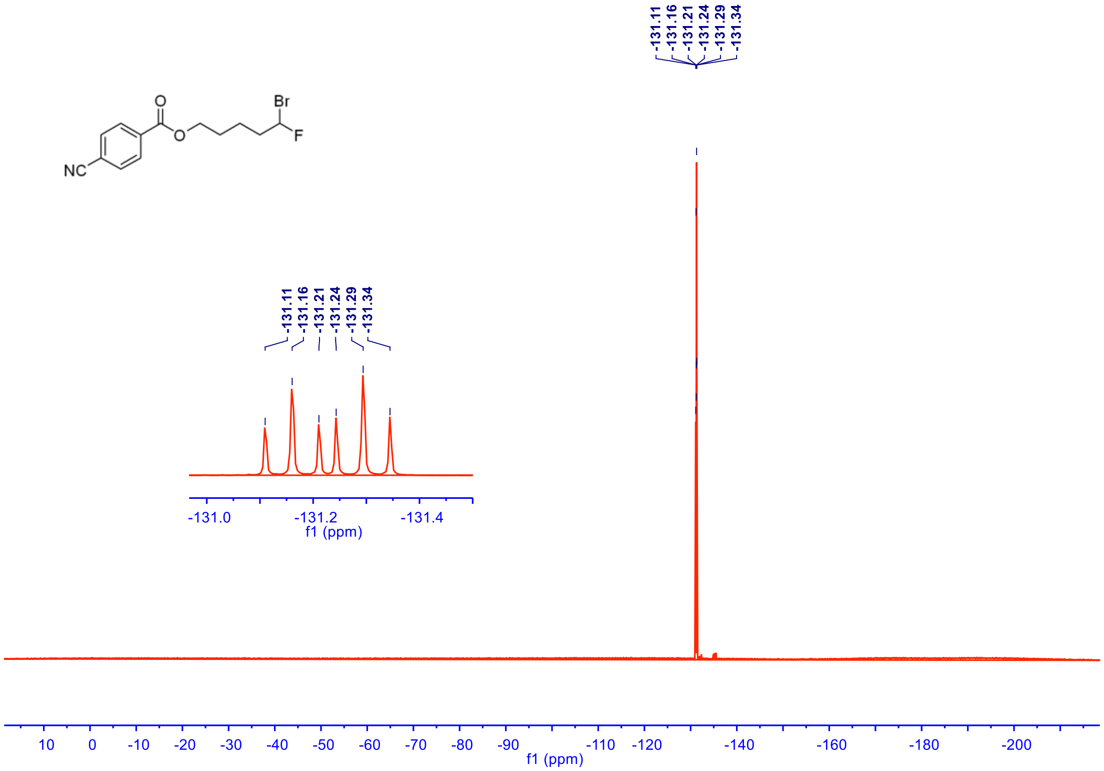

Supplementary Figure 236. ^{19}F NMR spectrum of **5-bromo-5-fluoropentyl 4-cyanobenzoate 20a** (376 MHz, $CDCl_3$)

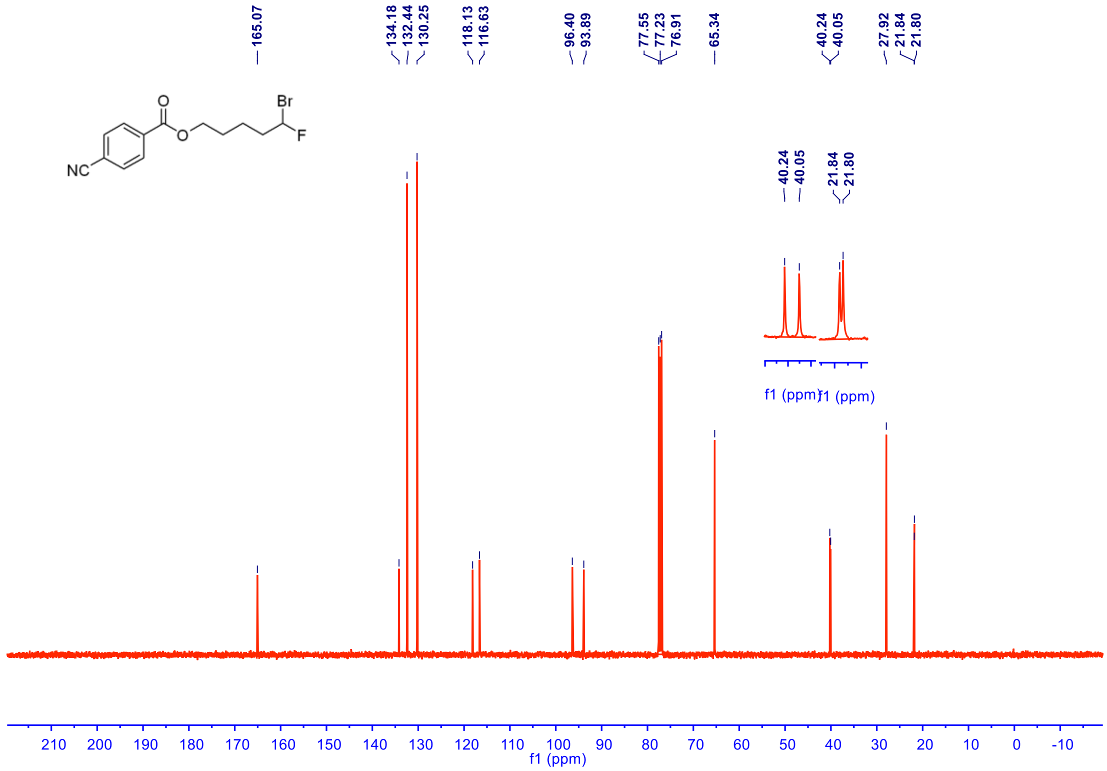

Supplementary Figure 237. ^{13}C NMR spectrum of **5-bromo-5-fluoropentyl 4-cyanobenzoate 20a** (101 MHz, $CDCl_3$)

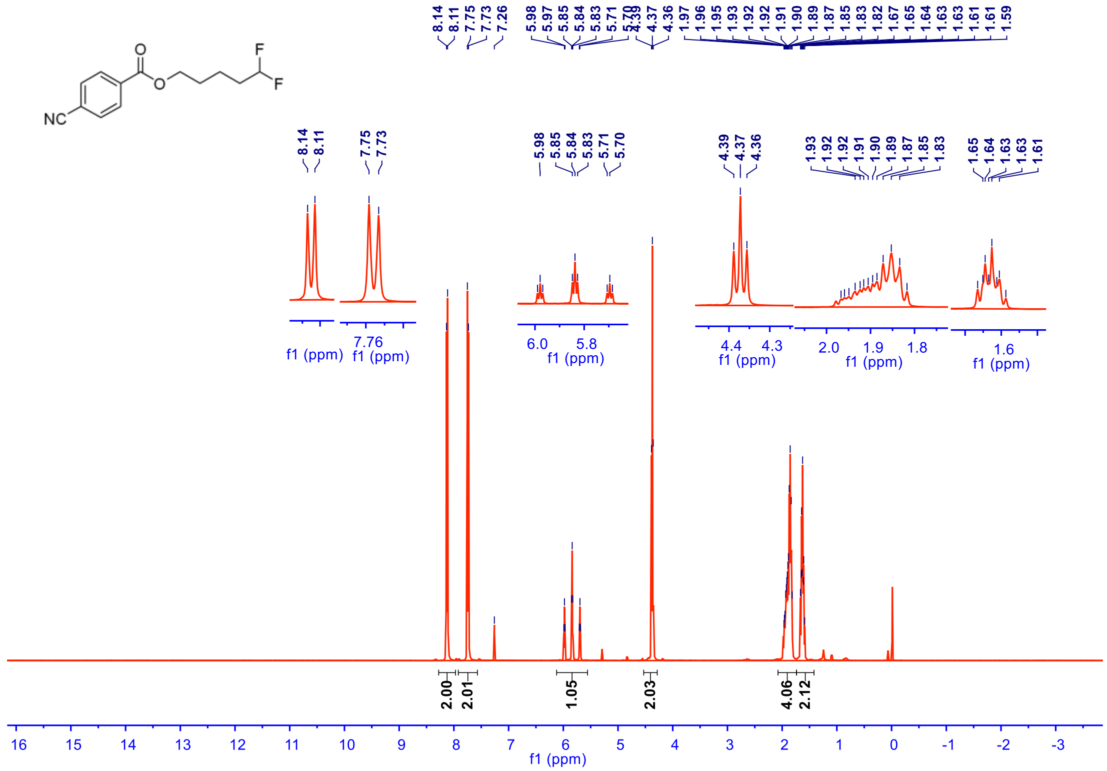

Supplementary Figure 238. ^{1}H NMR spectrum of compound **20** (400 MHz, $CDCl_3$)

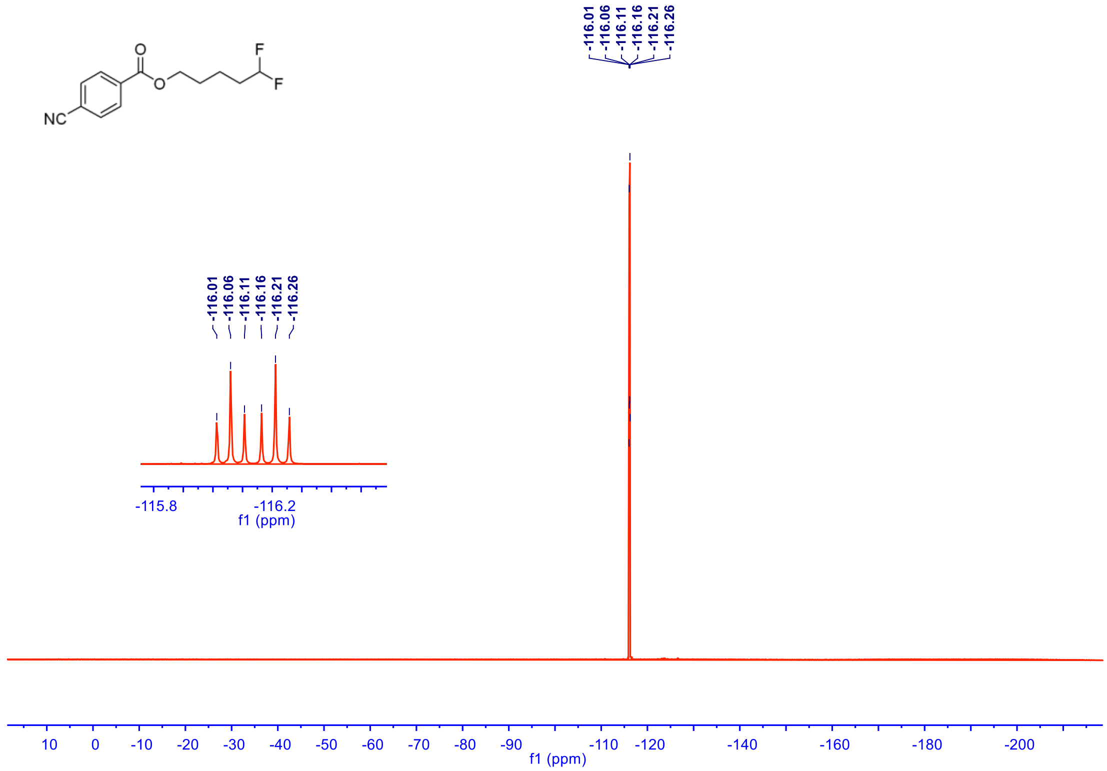

Supplementary Figure 239. ^{19}F NMR spectrum of compound **20** (376 MHz, $CDCl_3$)

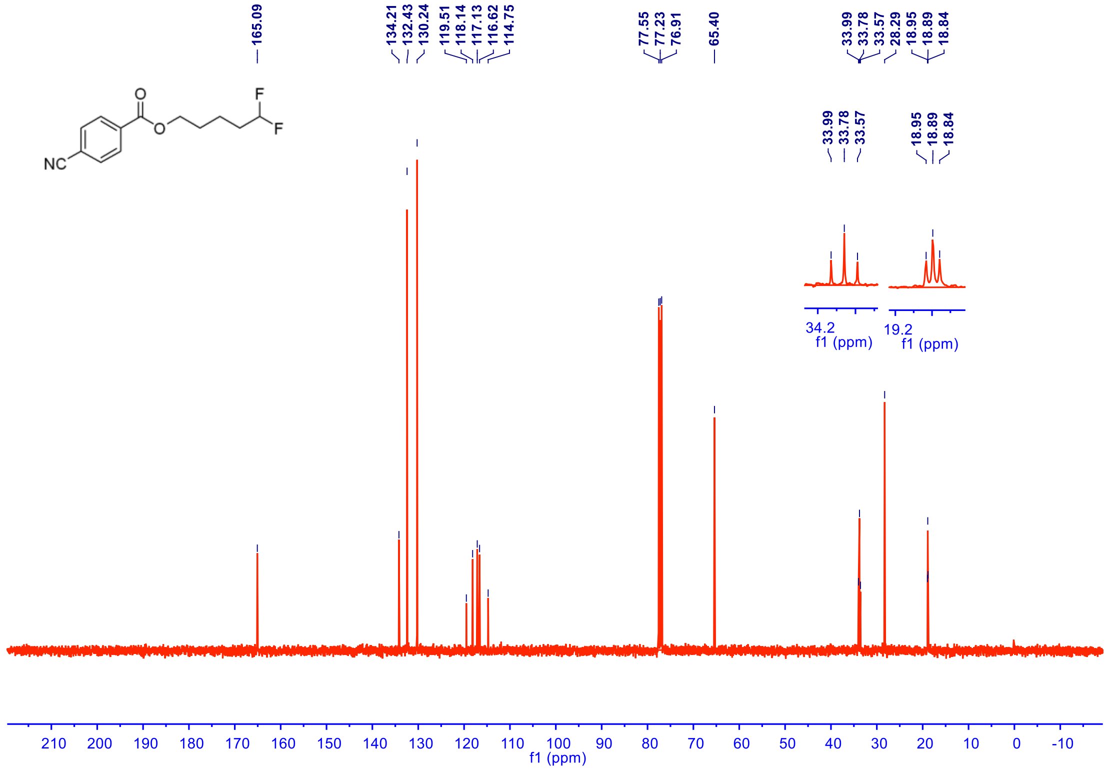

Supplementary Figure 240. ^{13}C NMR spectrum of compound **20** (101 MHz, $CDCl_3$)

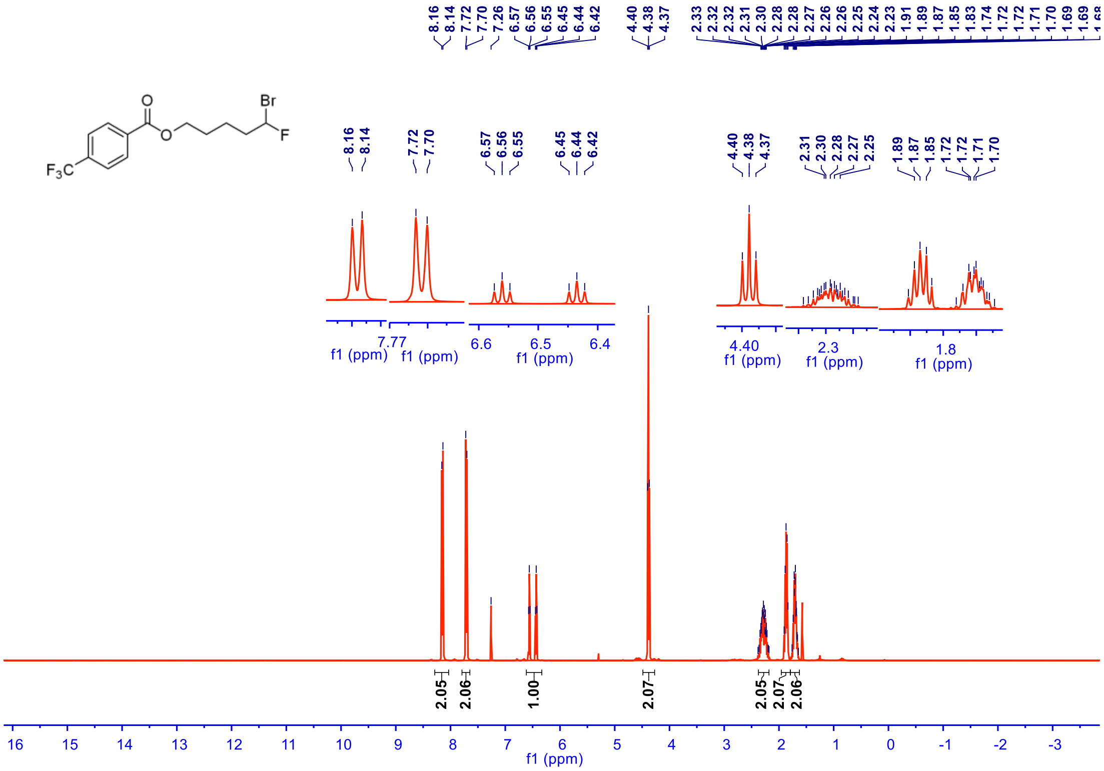

Supplementary Figure 241. ^{1}H NMR spectrum of **5-bromo-5-fluoropentyl 4-(trifluoromethyl)benzoate 21a** (400 MHz, $CDCl_3$)

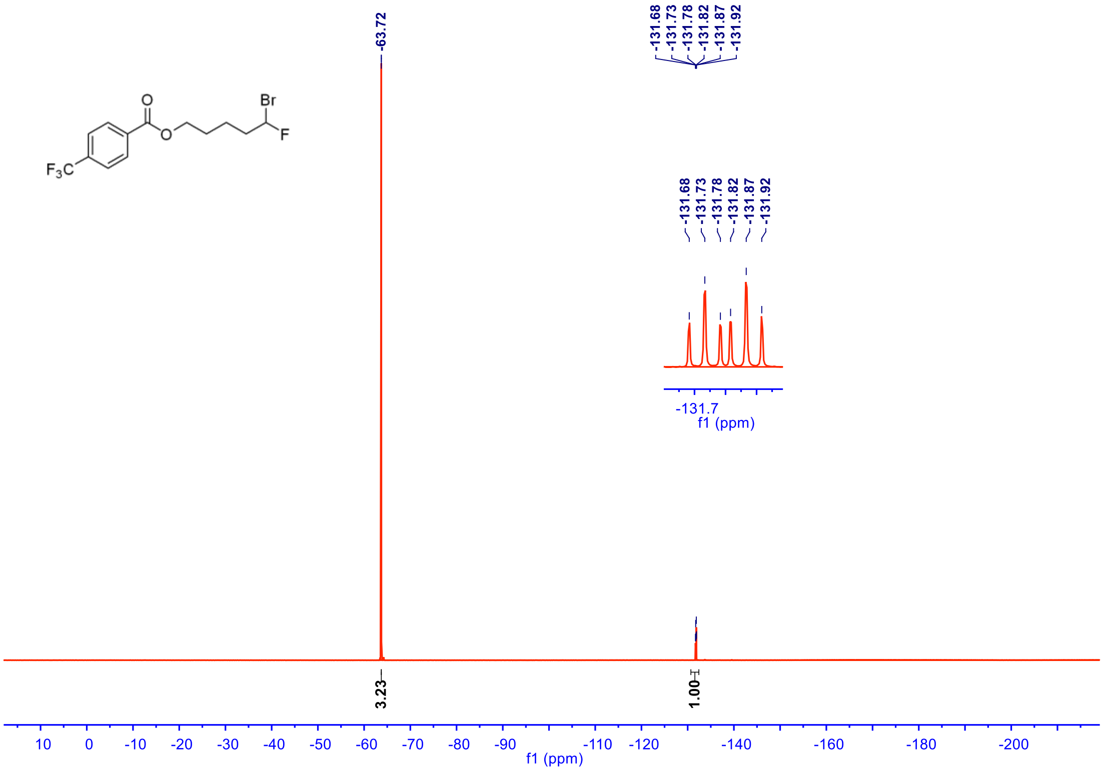

Supplementary Figure 242. ^{19}F NMR spectrum of **5-bromo-5-fluoropentyl 4-(trifluoromethyl)benzoate 21a** (376 MHz, $CDCl_3$)

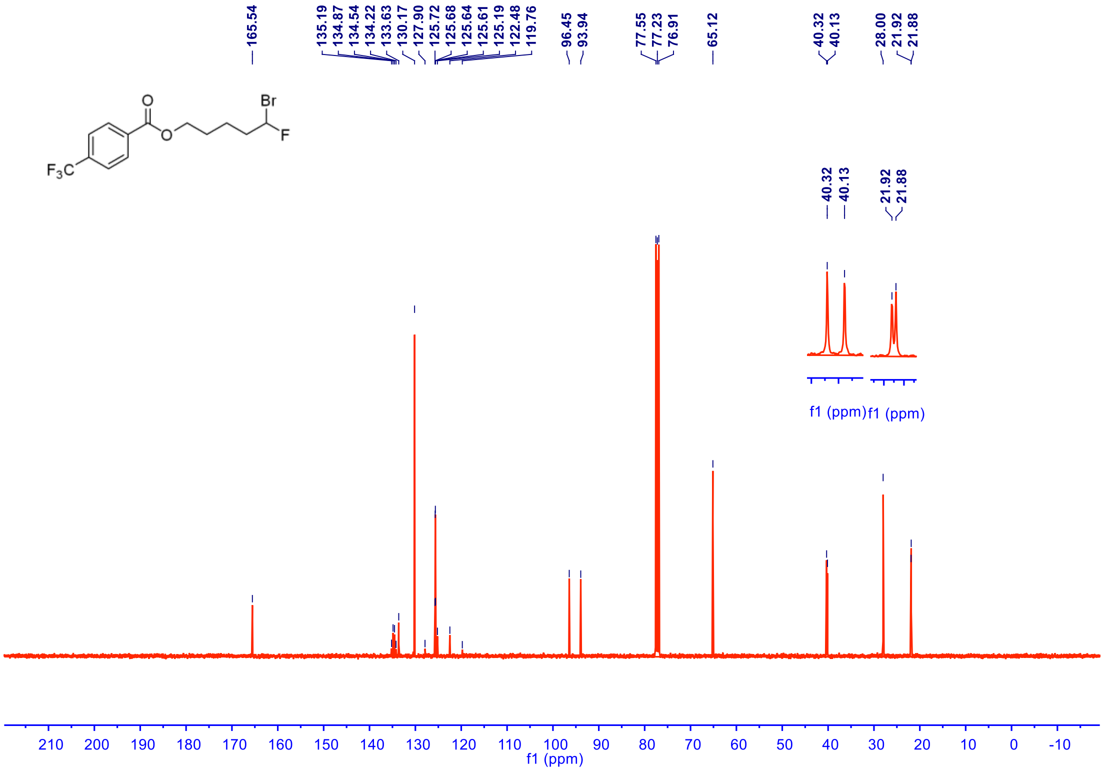

Supplementary Figure 243. ^{13}C NMR spectrum of **5-bromo-5-fluoropentyl 4-(trifluoromethyl)benzoate 21a** (101 MHz, $CDCl_3$)

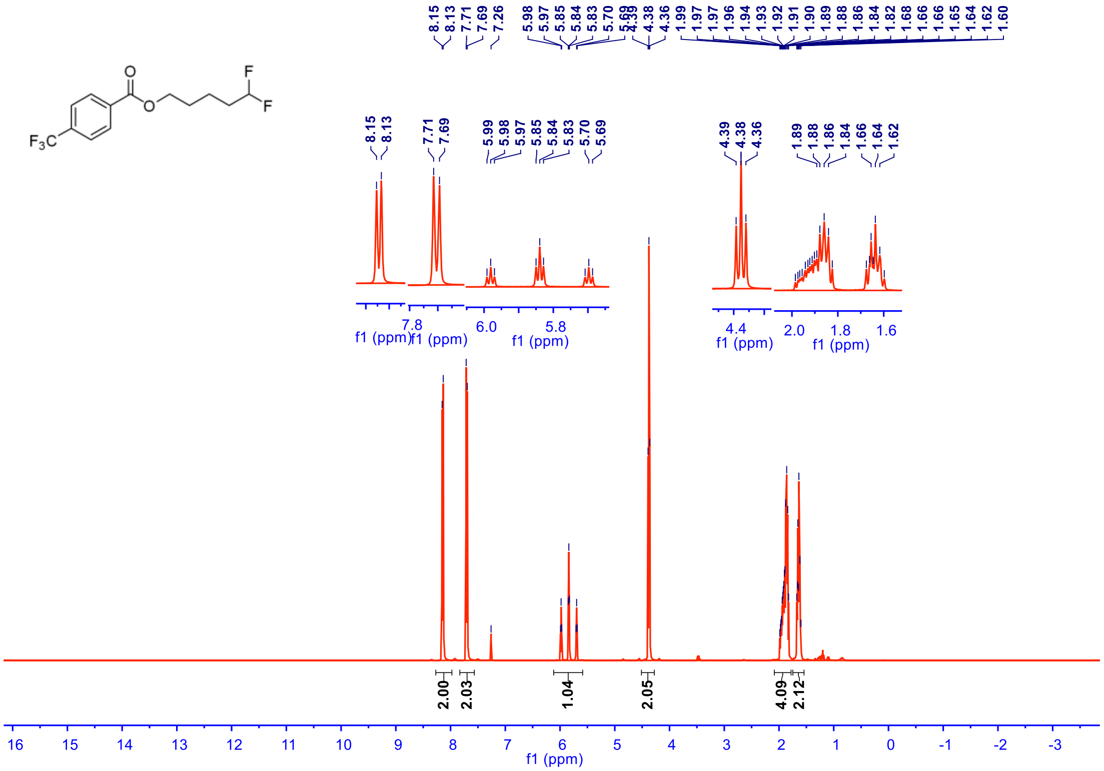

Supplementary Figure 244. ^{1}H NMR spectrum of compound **21** (400 MHz, $CDCl_3$)

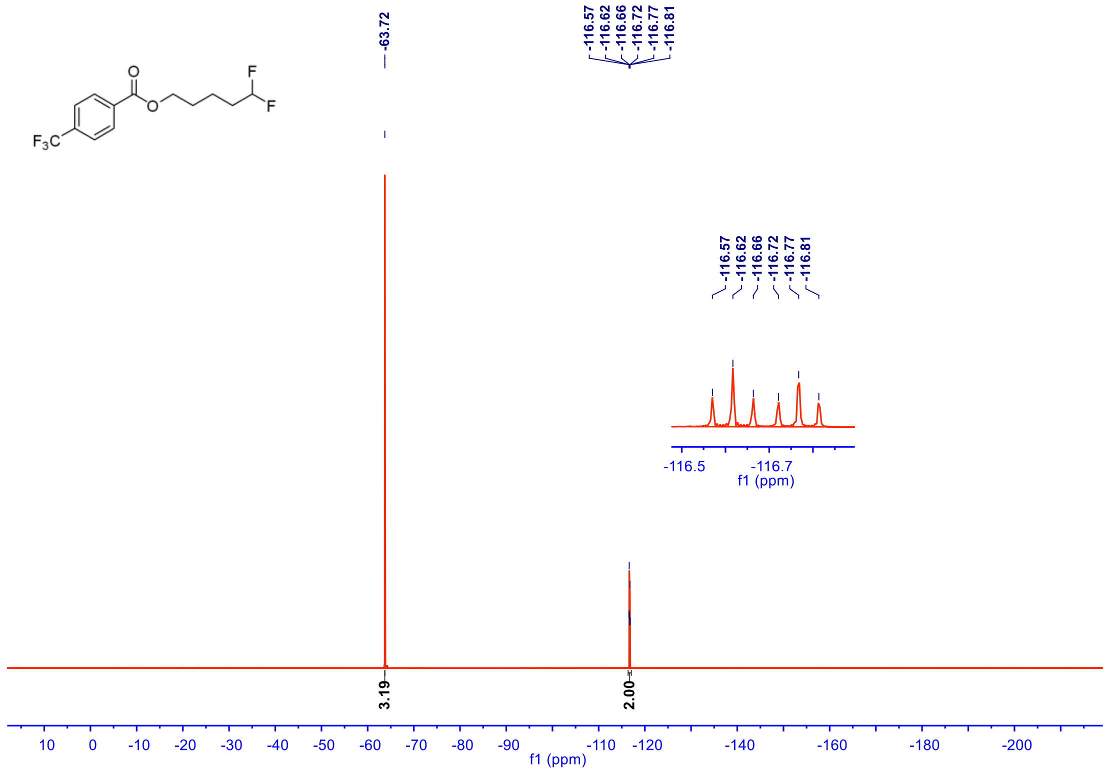

Supplementary Figure 245. ^{19}F NMR spectrum of compound **21** (376 MHz, $CDCl_3$)

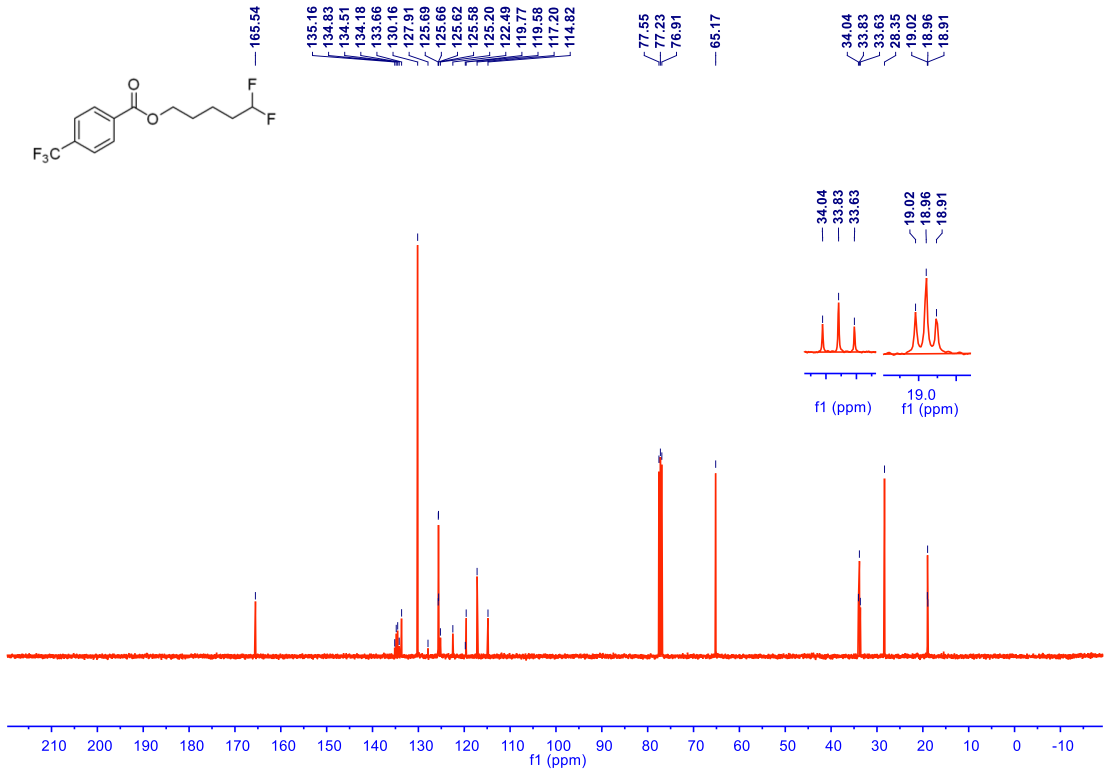

Supplementary Figure 246. ^{13}C NMR spectrum of compound **21** (101 MHz, $CDCl_3$)

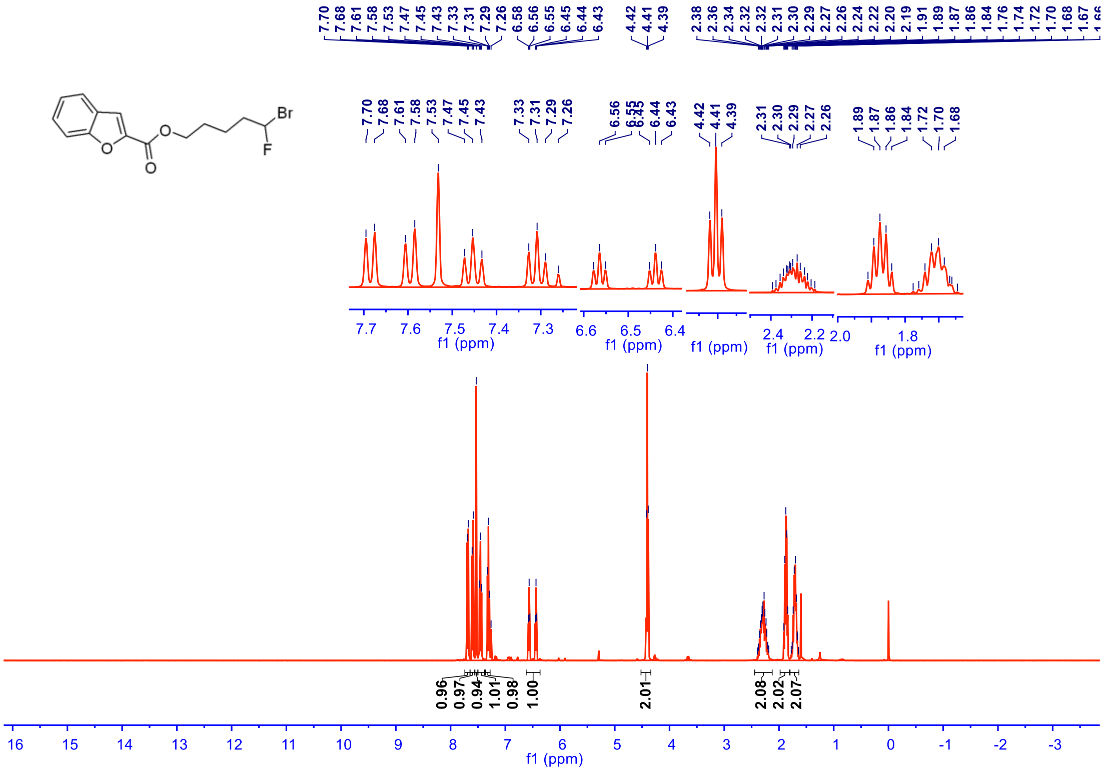

Supplementary Figure 247. ^{1}H NMR spectrum of **5-bromo-5-fluoropentyl benzofuran-2-carboxylate 22a** (400 MHz, $CDCl_3$)

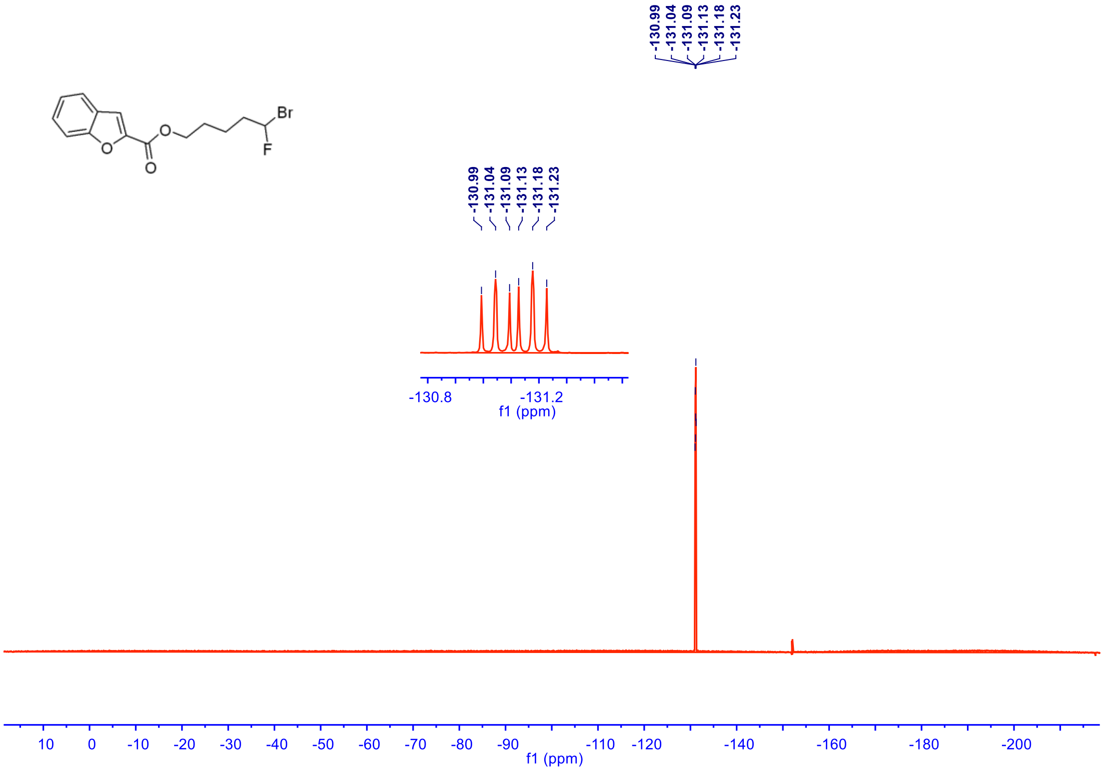

Supplementary Figure 248. ^{19}F NMR spectrum of **5-bromo-5-fluoropentyl benzofuran-2-carboxylate 22a** (376 MHz, $CDCl_3$)

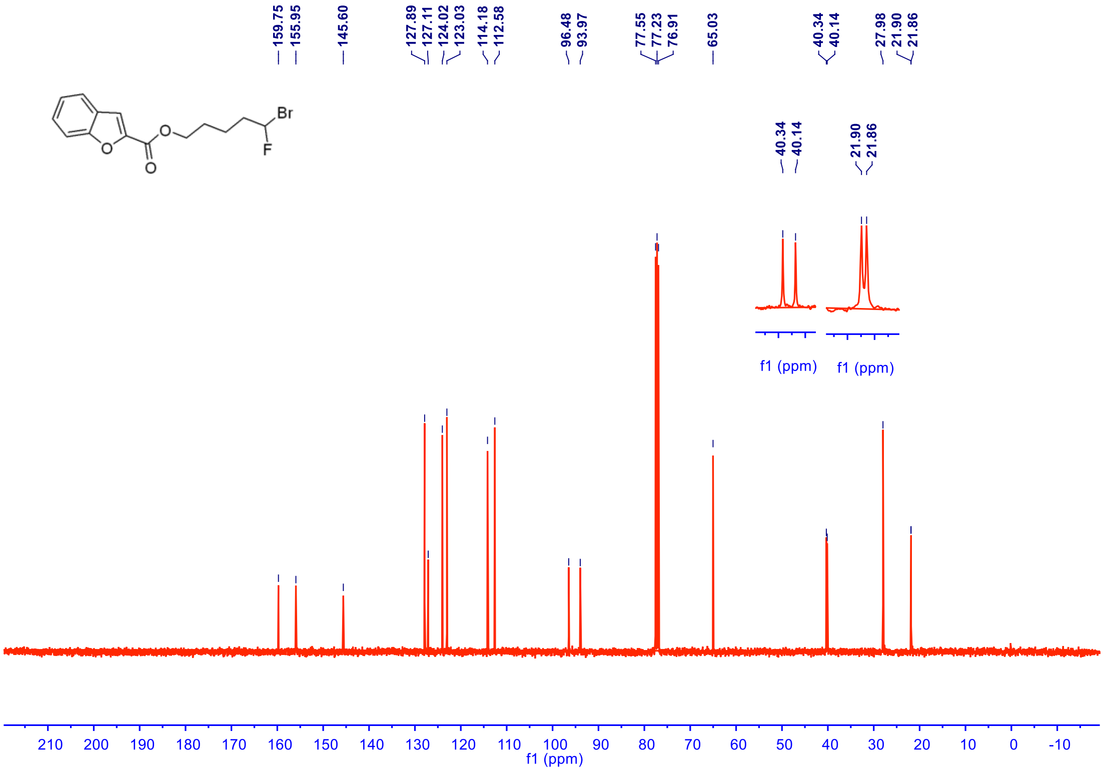

Supplementary Figure 249. ^{13}C NMR spectrum of **5-bromo-5-fluoropentyl benzofuran-2-carboxylate 22a** (101 MHz, $CDCl_3$)

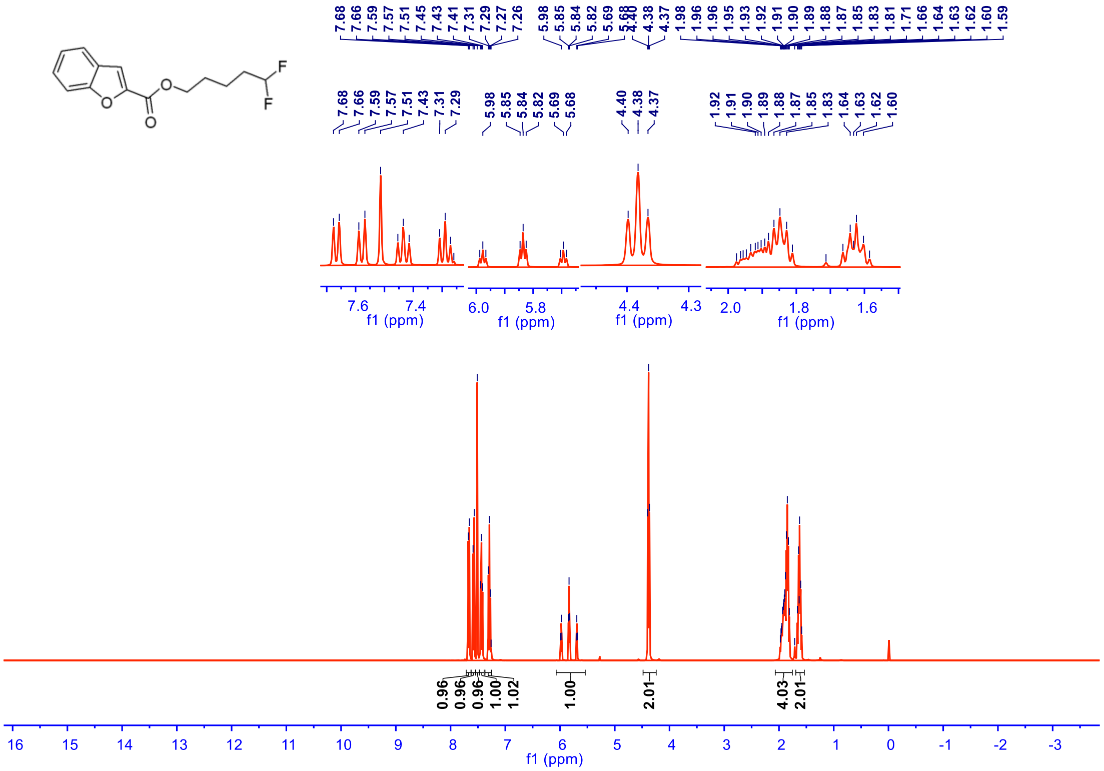

Supplementary Figure 250. ^{1}H NMR spectrum of compound **22** (400 MHz, $CDCl_3$)

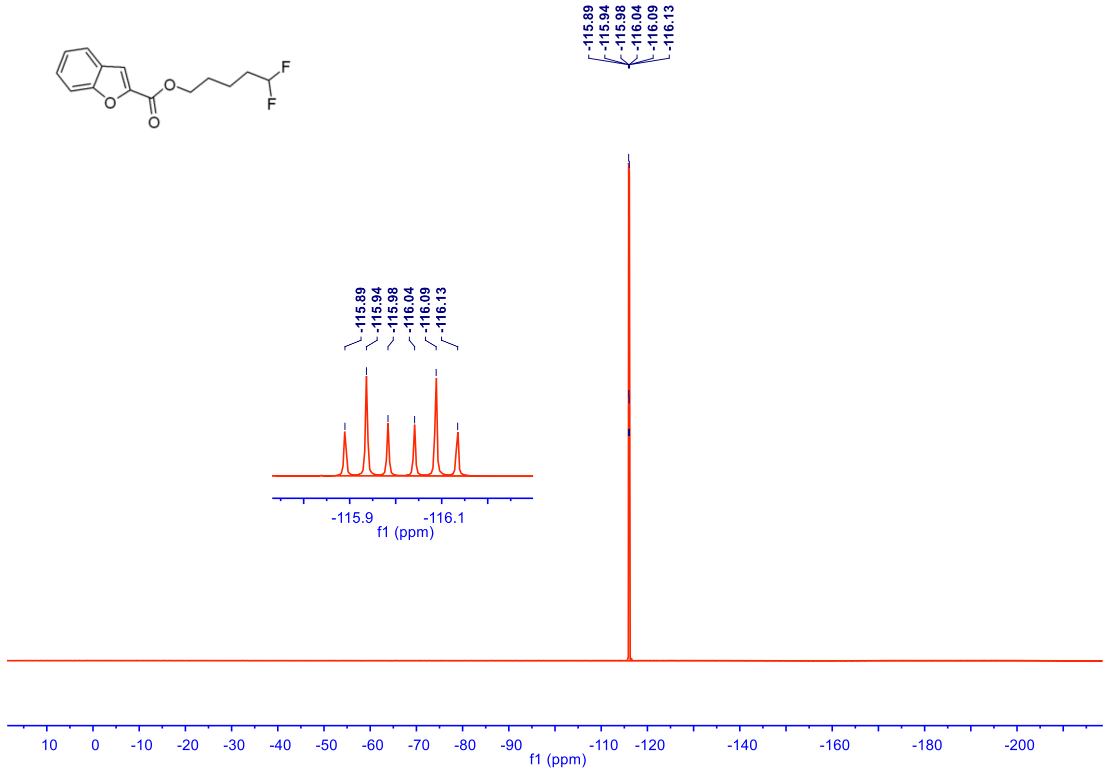

Supplementary Figure 251. ^{19}F NMR spectrum of compound **22** (376 MHz, $CDCl_3$)

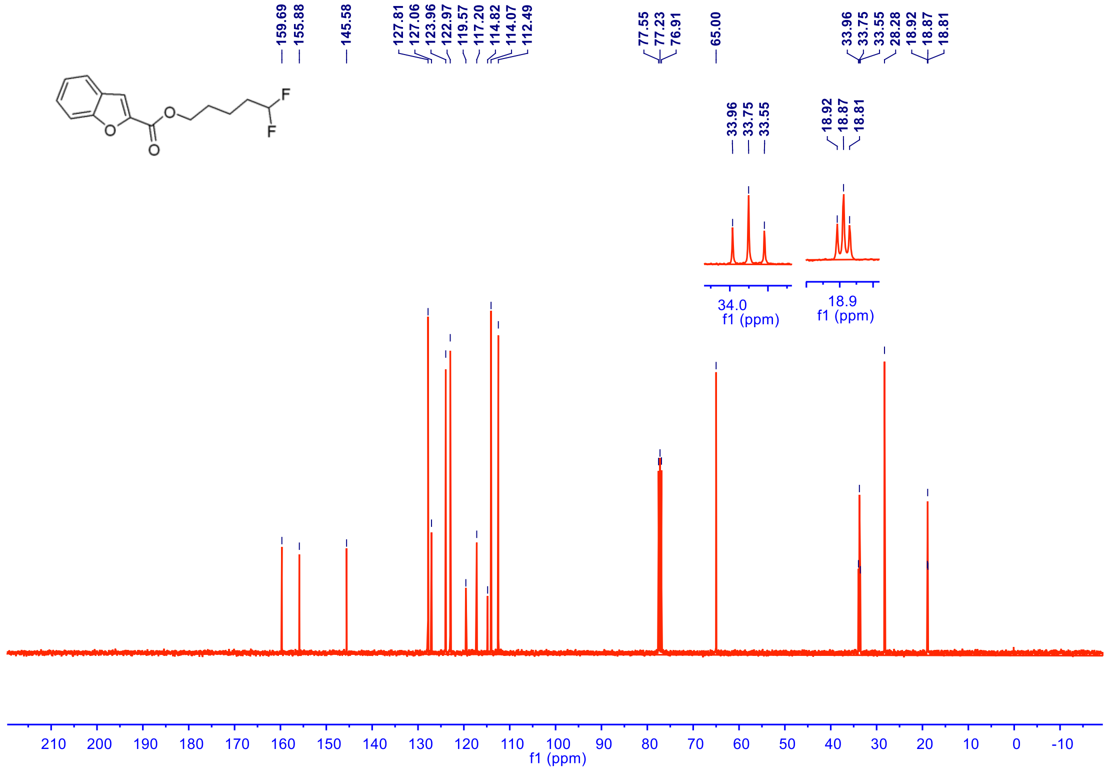

Supplementary Figure 252. ^{13}C NMR spectrum of compound **22** (101 MHz, $CDCl_3$)

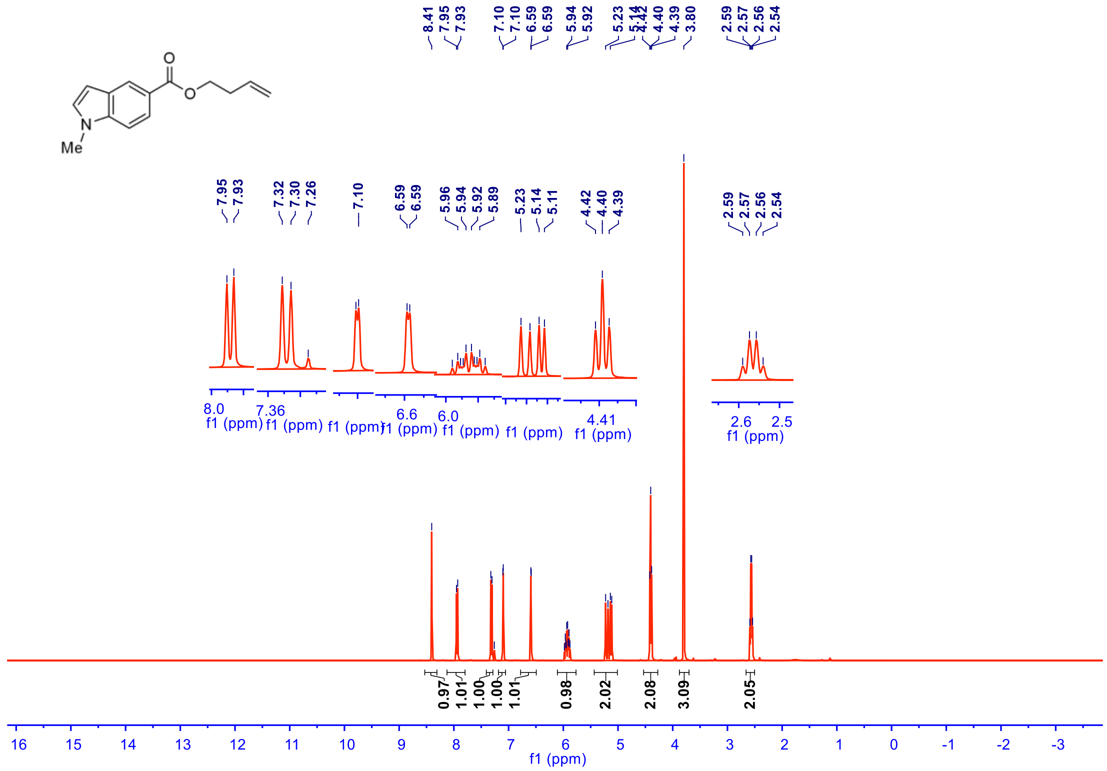

Supplementary Figure 253. ^{1}H NMR spectrum of **but-3-en-1-yl 1-methyl-1*H*-indole-5-carboxylate 23b** (400 MHz, $CDCl_3$)

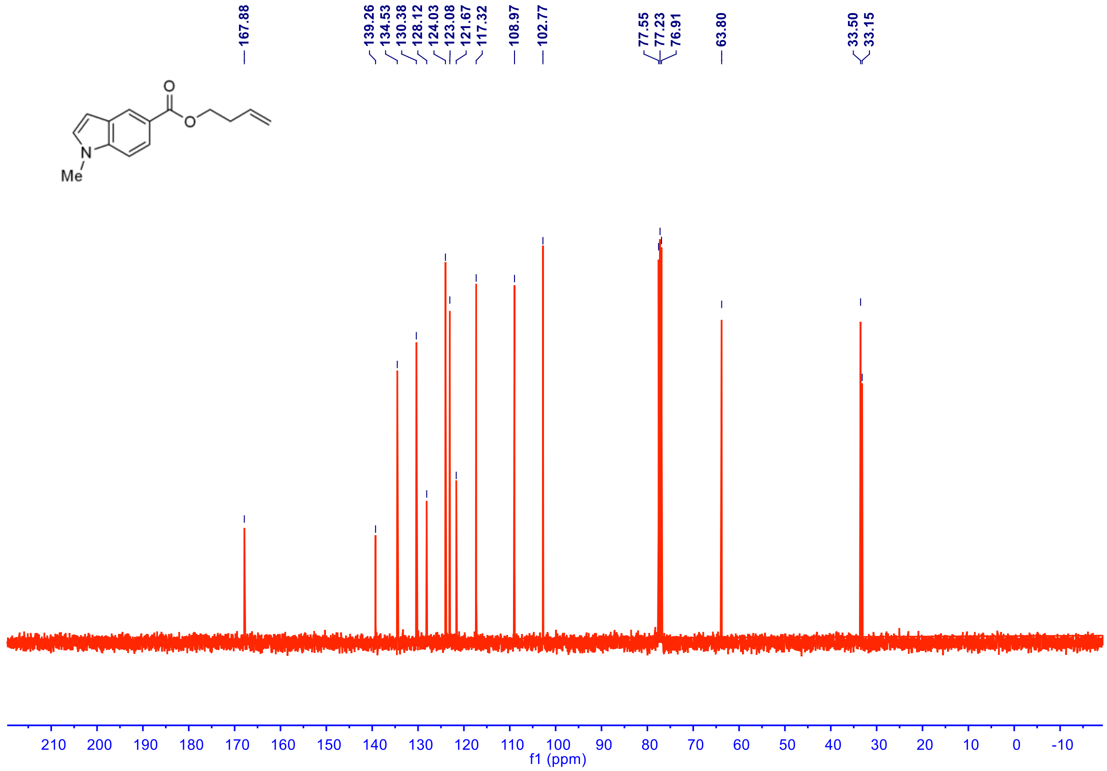

Supplementary Figure 254. ^{13}C NMR spectrum of **but-3-en-1-yl 1-methyl-1*H*-indole-5-carboxylate 23b** (101 MHz, $CDCl_3$)

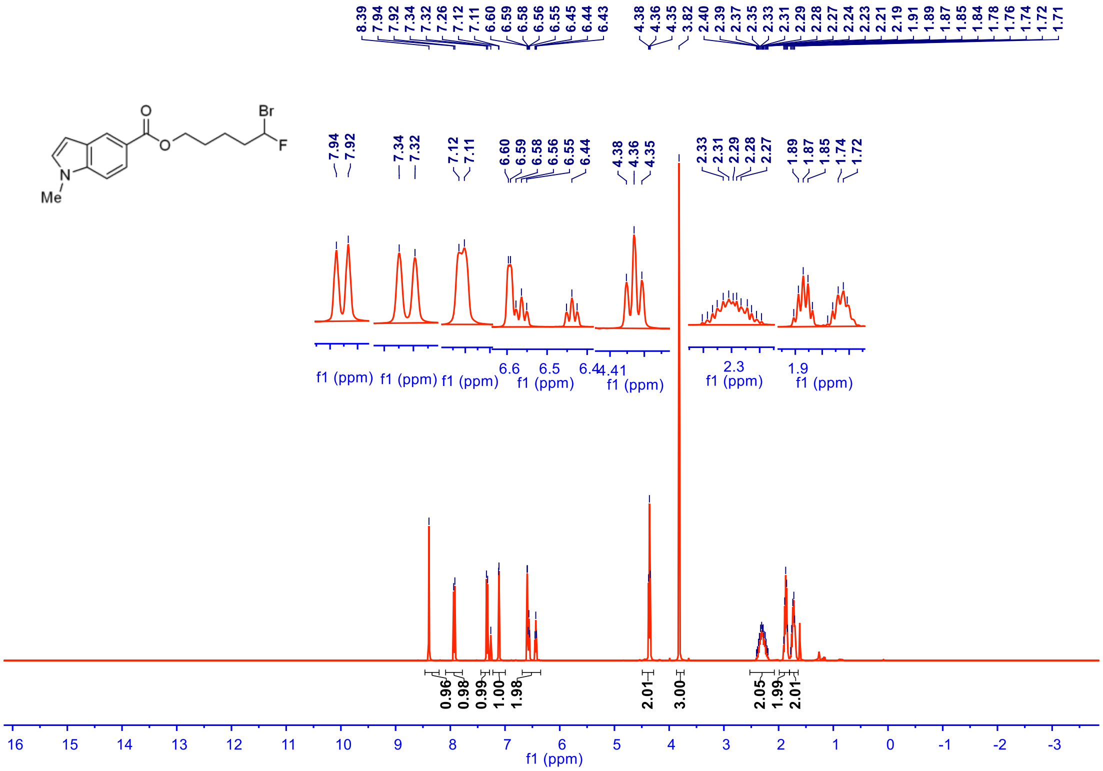

Supplementary Figure 255. ^{1}H NMR spectrum of **5-bromo-5-fluoropentyl 1-methyl-1*H*-indole-5-carboxylate 23a** (400 MHz, $CDCl_3$)

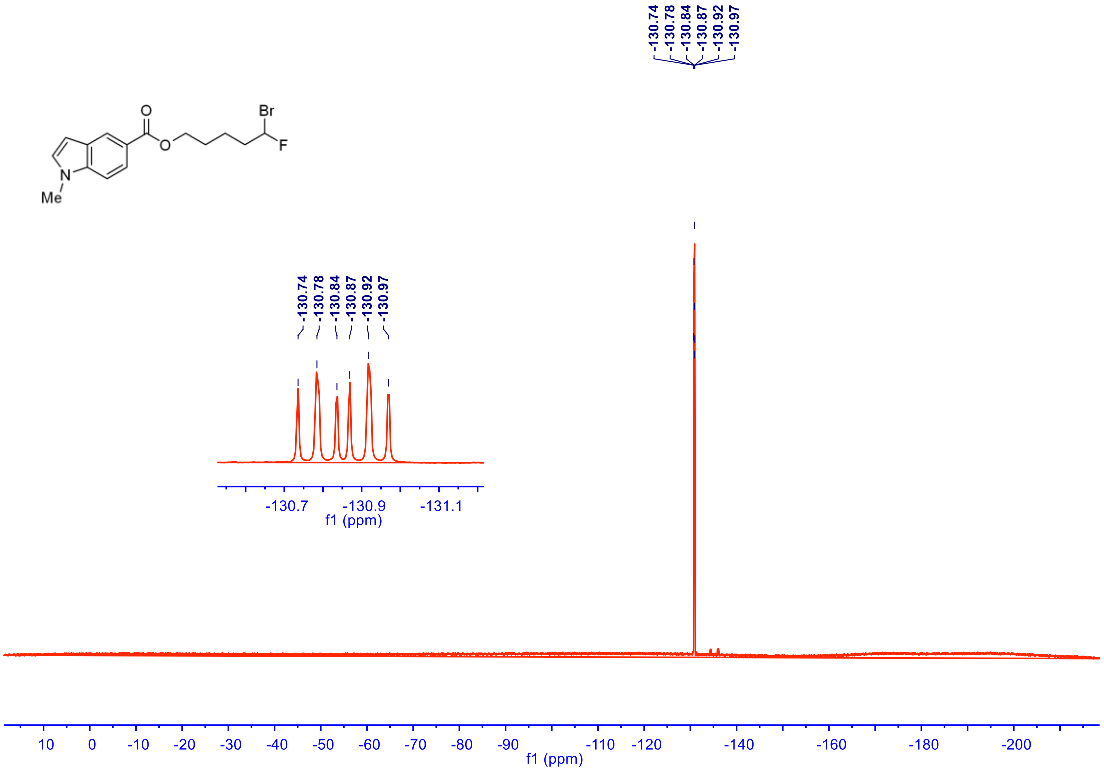

Supplementary Figure 256. ^{19}F NMR spectrum of **5-bromo-5-fluoropentyl 1-methyl-1*H*-indole-5-carboxylate 23a** (376 MHz, $CDCl_3$)

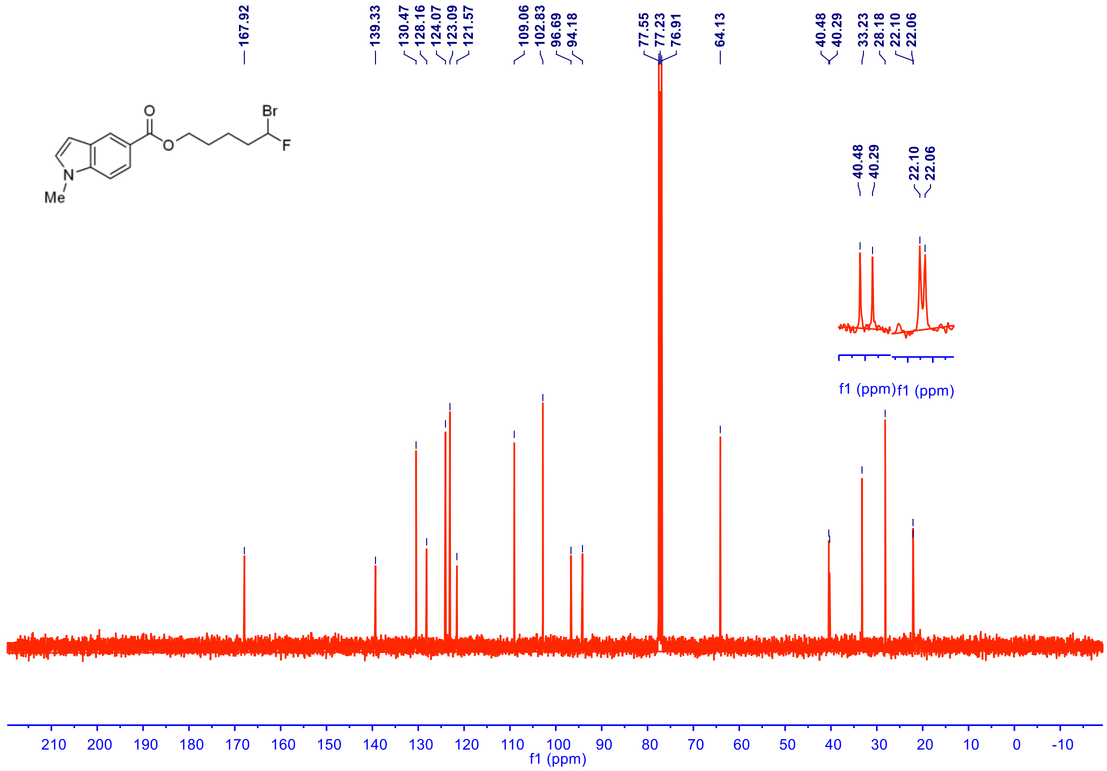

Supplementary Figure 257. ^{13}C NMR spectrum of **5-bromo-5-fluoropentyl 1-methyl-1*H*-indole-5-carboxylate 23a** (101 MHz, $CDCl_3$)

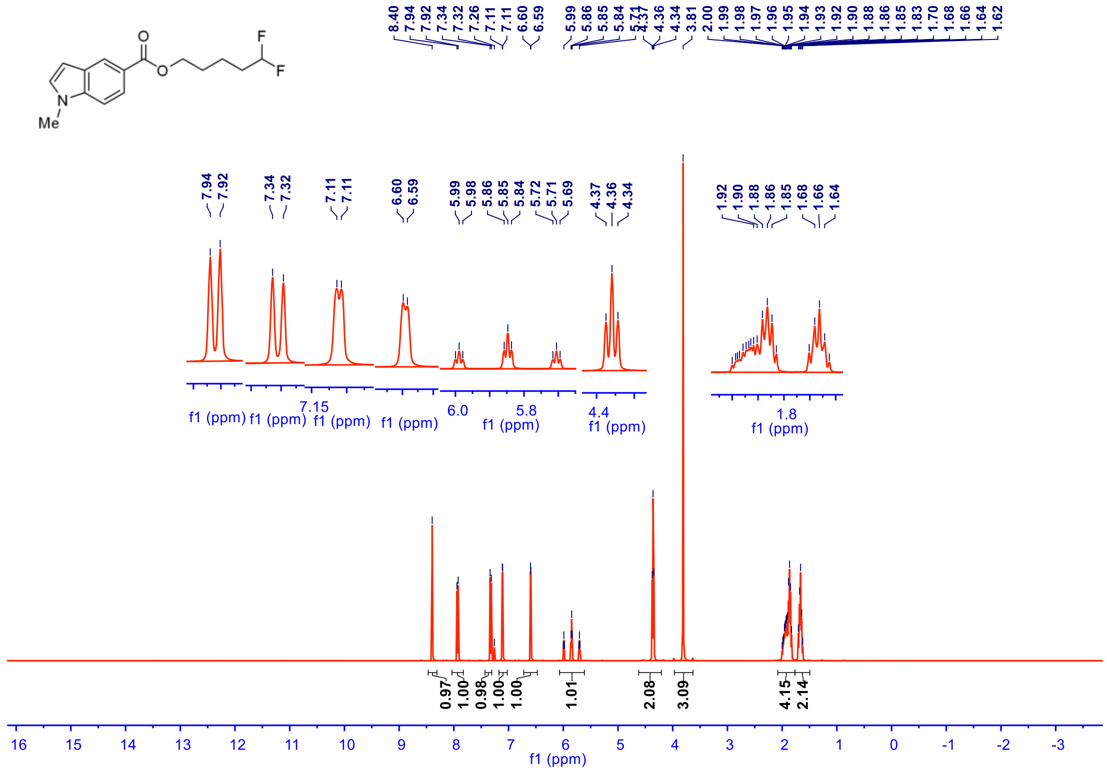

Supplementary Figure 258. ^{1}H NMR spectrum of compound **23** (400 MHz, $CDCl_3$)

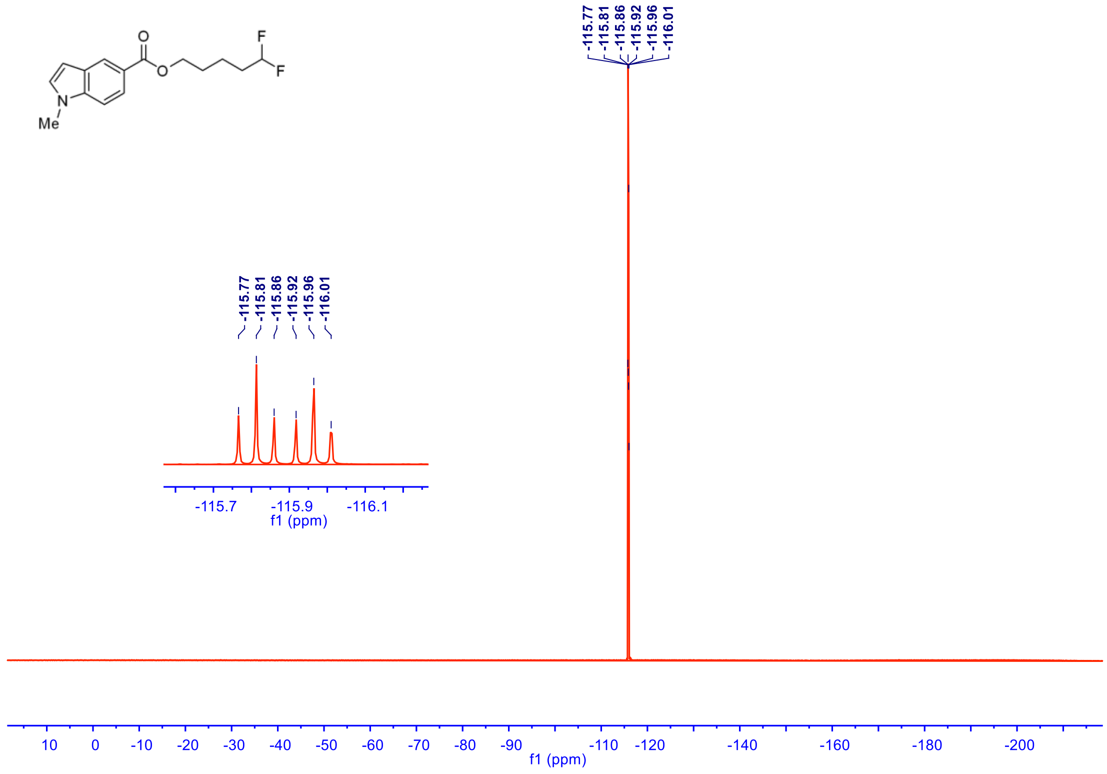

Supplementary Figure 259. ^{19}F NMR spectrum of compound **23** (376 MHz, $CDCl_3$)

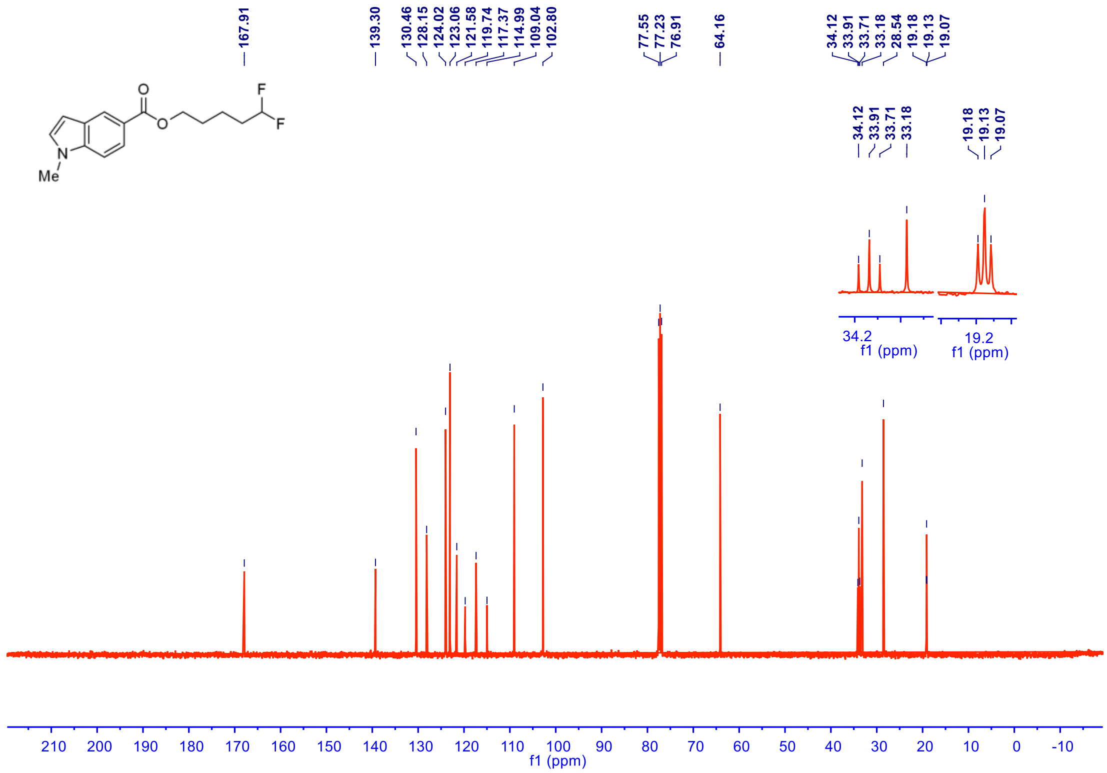

Supplementary Figure 260. ^{13}C NMR spectrum of compound **23** (101 MHz, $CDCl_3$)

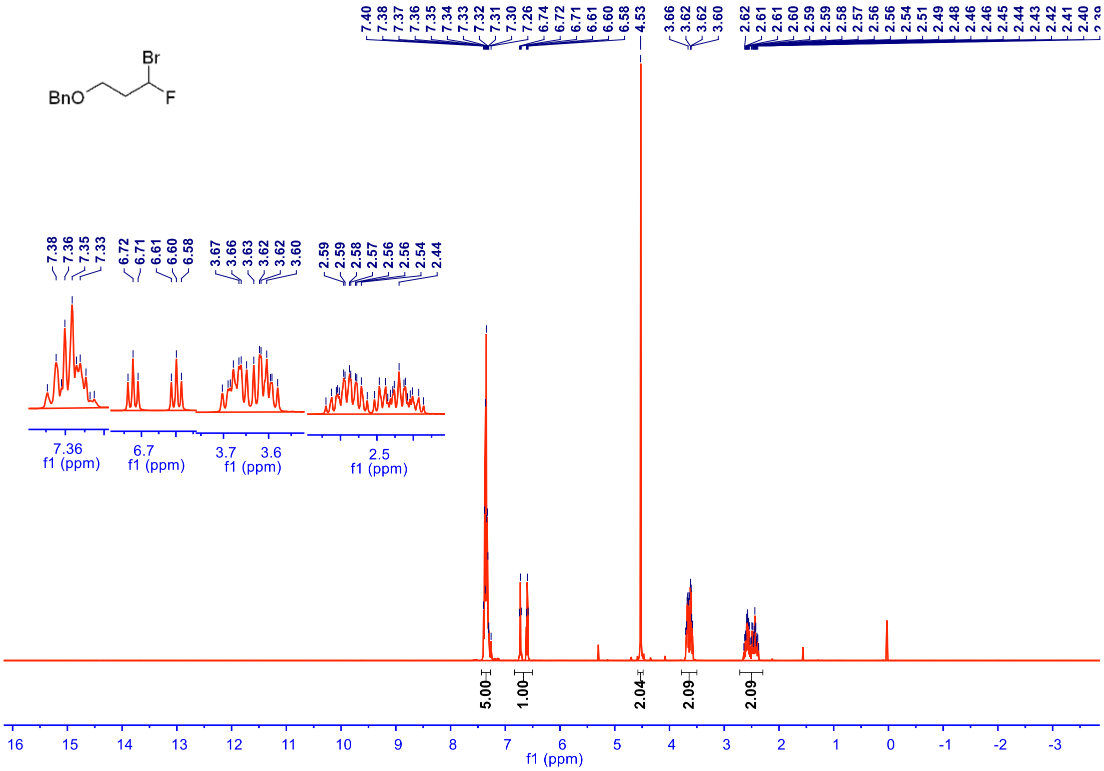

Supplementary Figure 261. ^{1}H NMR spectrum of **((3-bromo-3-fluoropropoxy)methyl)benzene 24a** (400 MHz, $CDCl_3$)

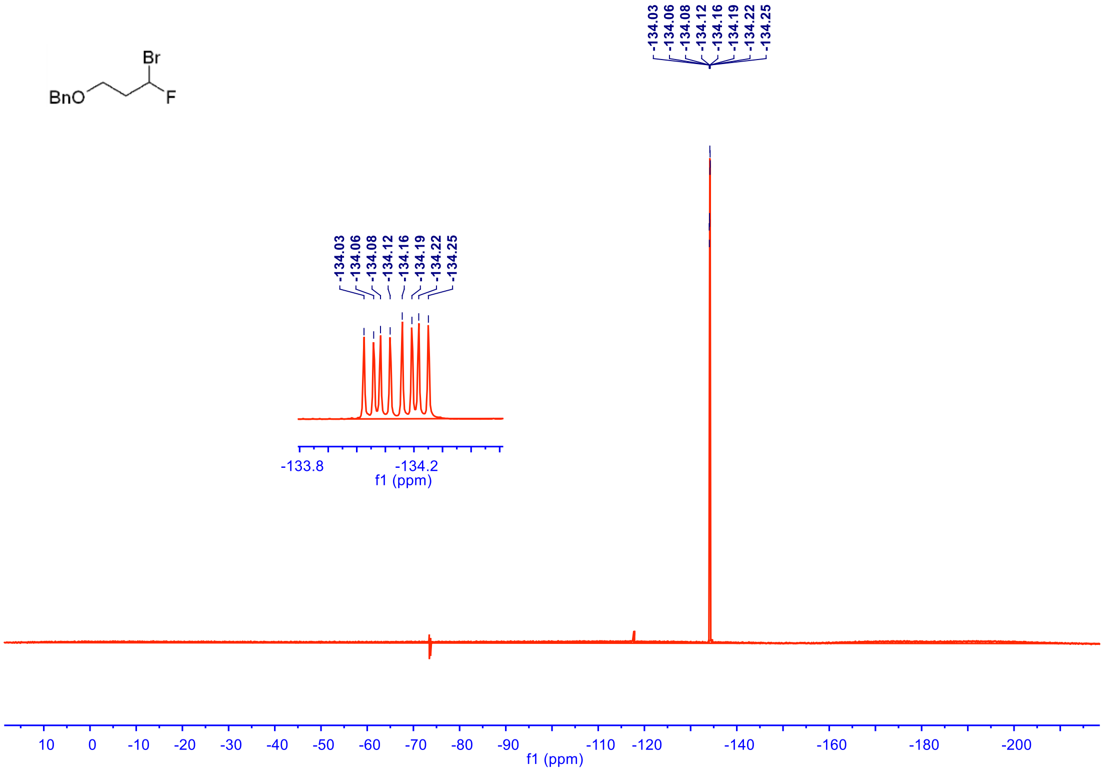

Supplementary Figure 262. ^{19}F NMR spectrum of **((3-bromo-3-fluoropropoxy)methyl)benzene 24a** (376 MHz, $CDCl_3$)

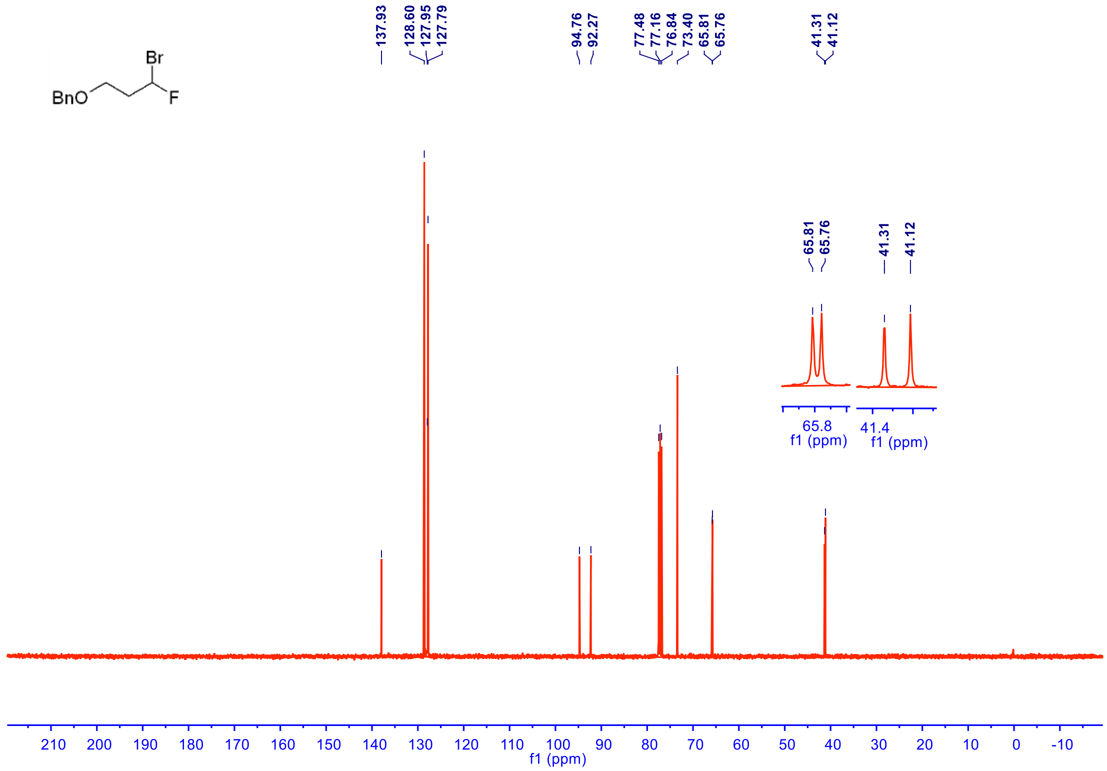

Supplementary Figure 263. ^{13}C NMR spectrum of **((3-bromo-3-fluoropropoxy)methyl)benzene 24a** (101 MHz, $CDCl_3$)

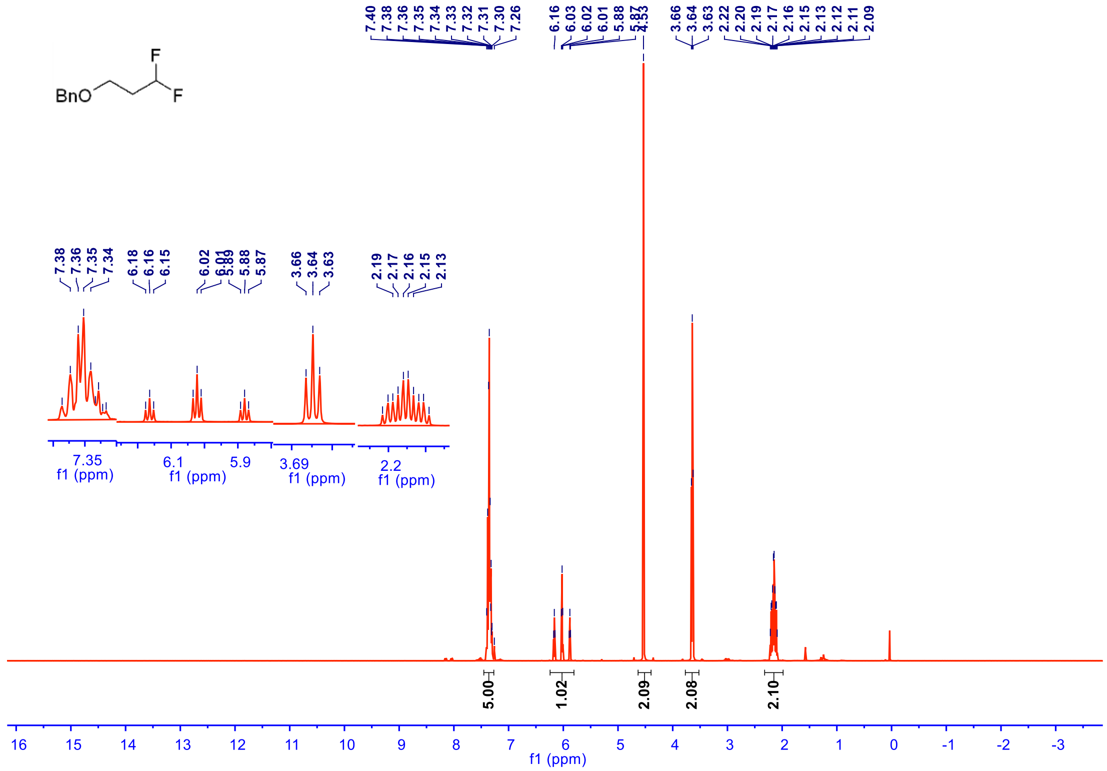

Supplementary Figure 264. ^{1}H NMR spectrum of compound **24** (400 MHz, $CDCl_3$)

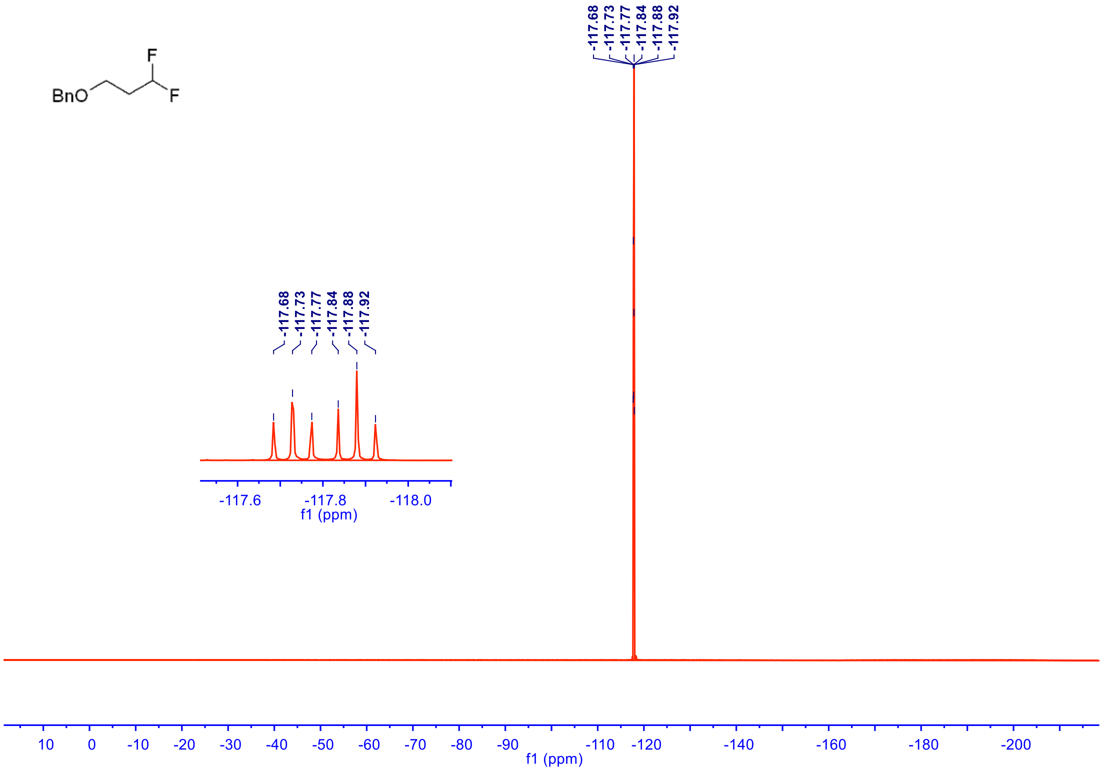

Supplementary Figure 265. ^{19}F NMR spectrum of compound **24** (376 MHz, $CDCl_3$)

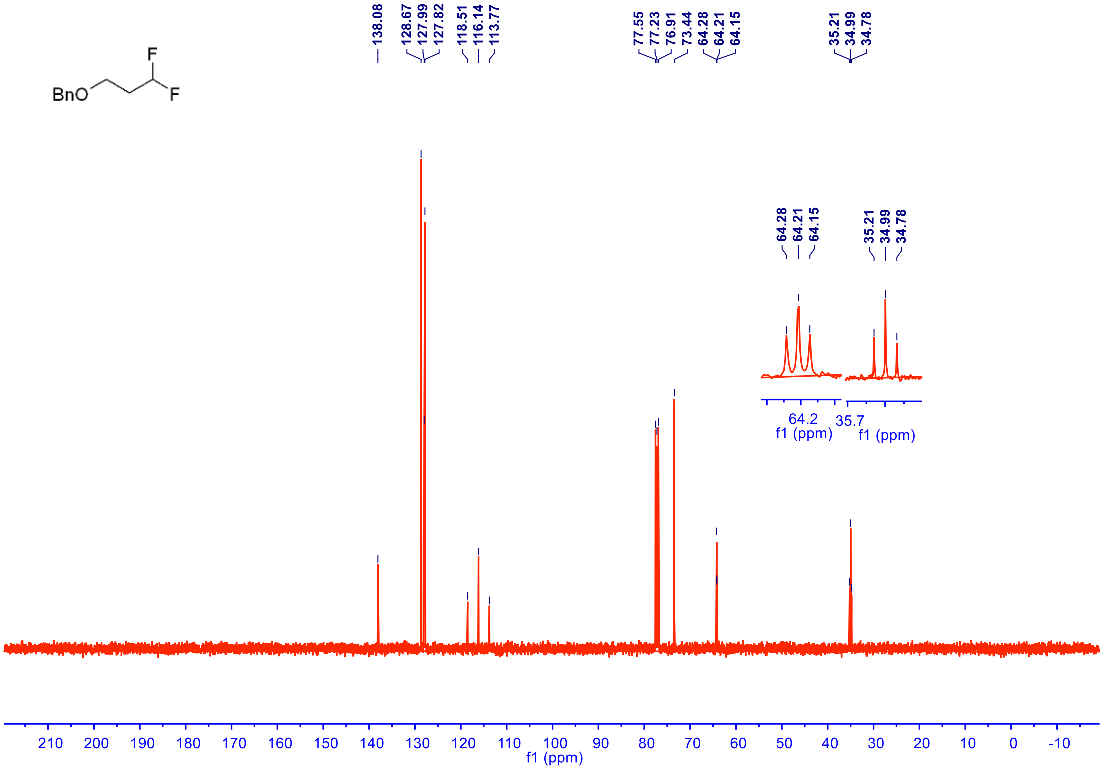

Supplementary Figure 266. ^{13}C NMR spectrum of compound **24** (101 MHz, $CDCl_3$)

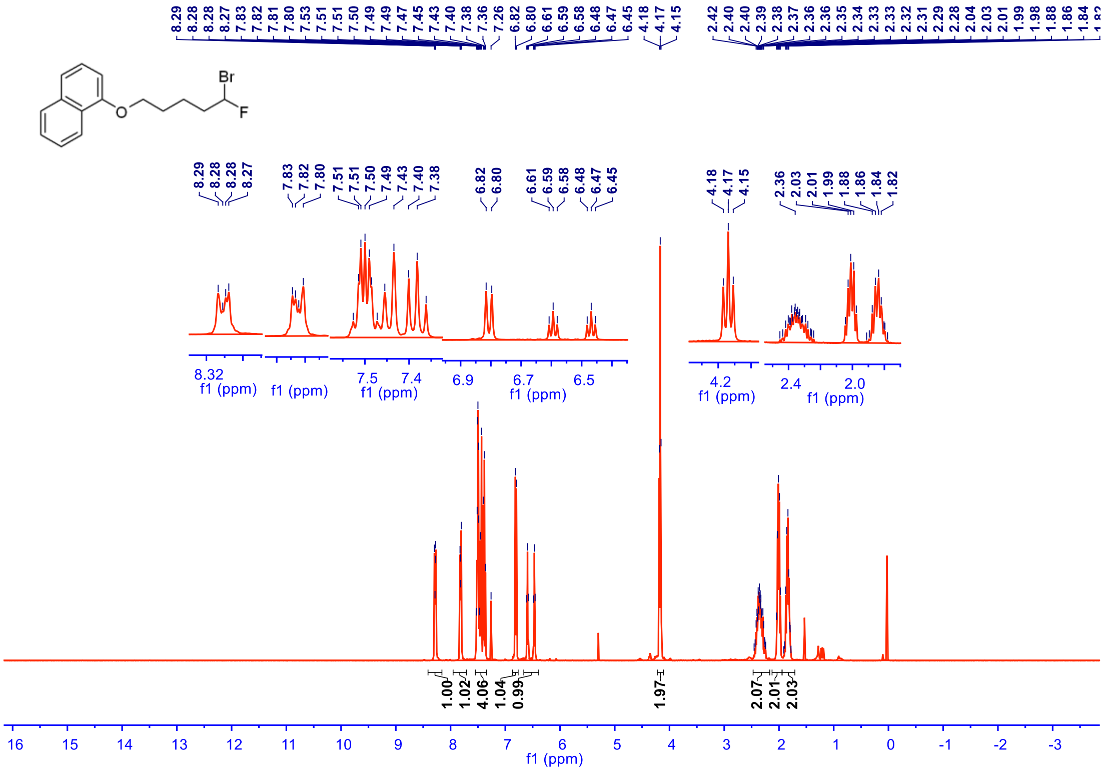

Supplementary Figure 267. ^{1}H NMR spectrum of **1-((5-bromo-5-fluoropentyl)oxy)naphthalene 25a** (400 MHz, $CDCl_3$)

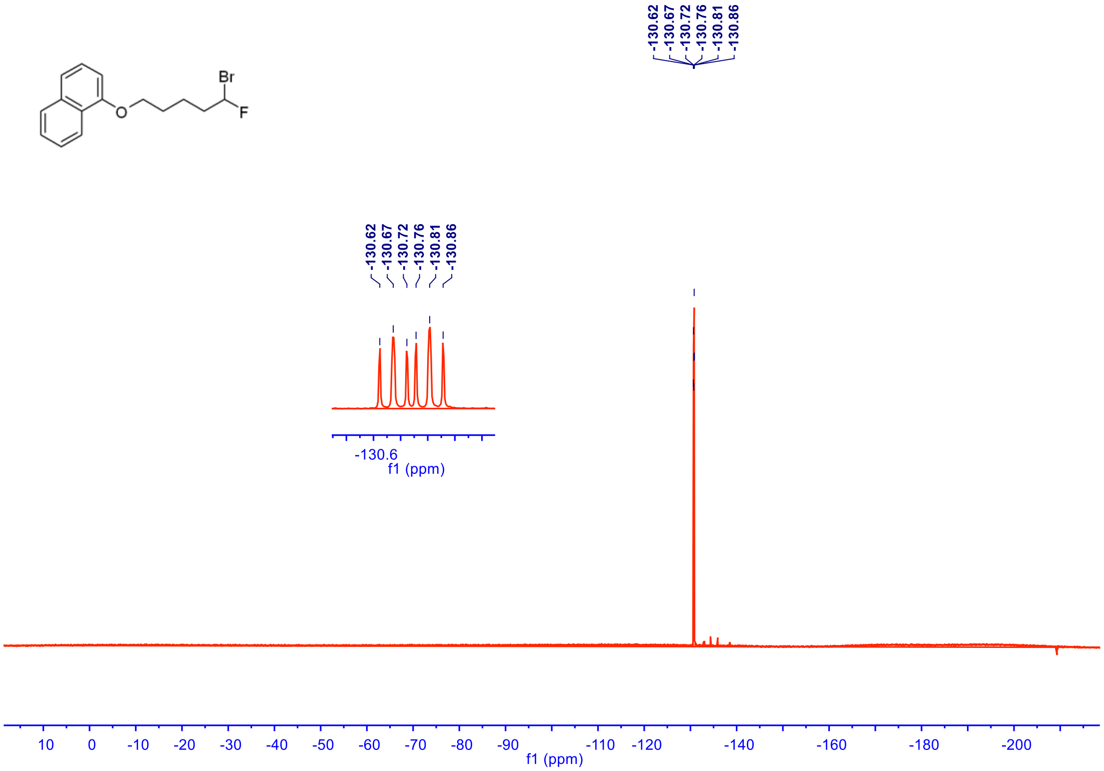

Supplementary Figure 268. ^{19}F NMR spectrum of **1-((5-bromo-5-fluoropentyl)oxy)naphthalene 25a** (376 MHz, $CDCl_3$)

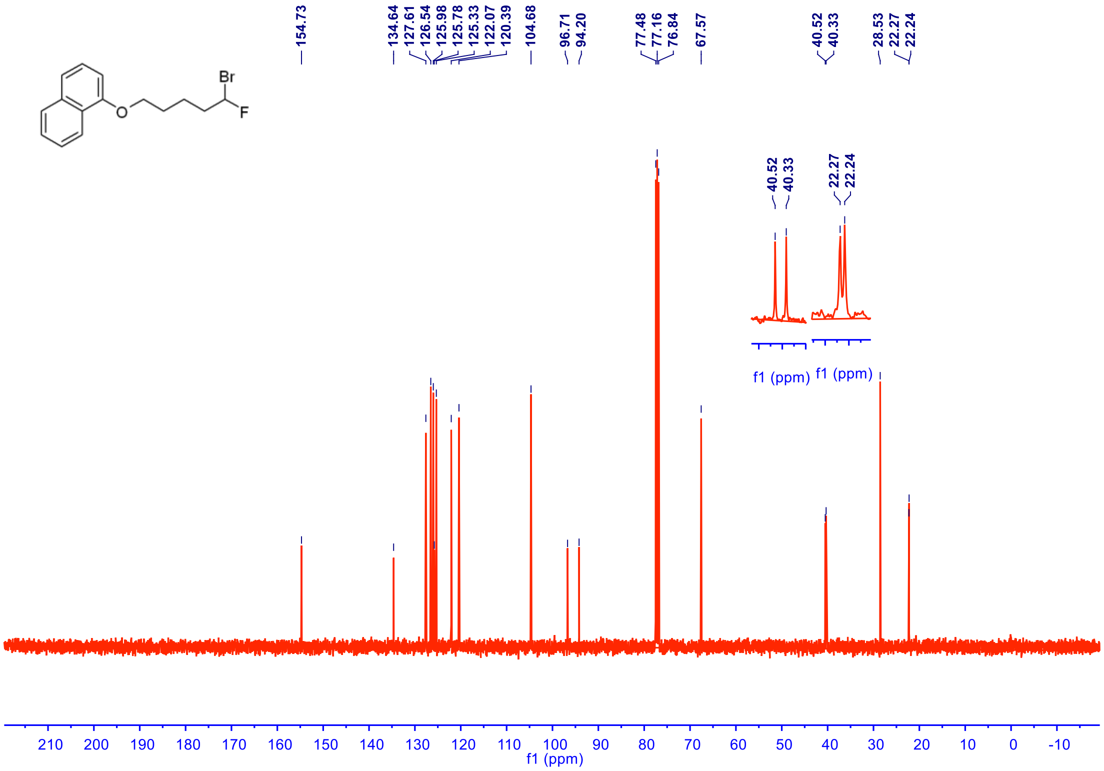

Supplementary Figure 269. ^{13}C NMR spectrum of **1-((5-bromo-5-fluoropentyl)oxy)naphthalene 25a** (101 MHz, $CDCl_3$)

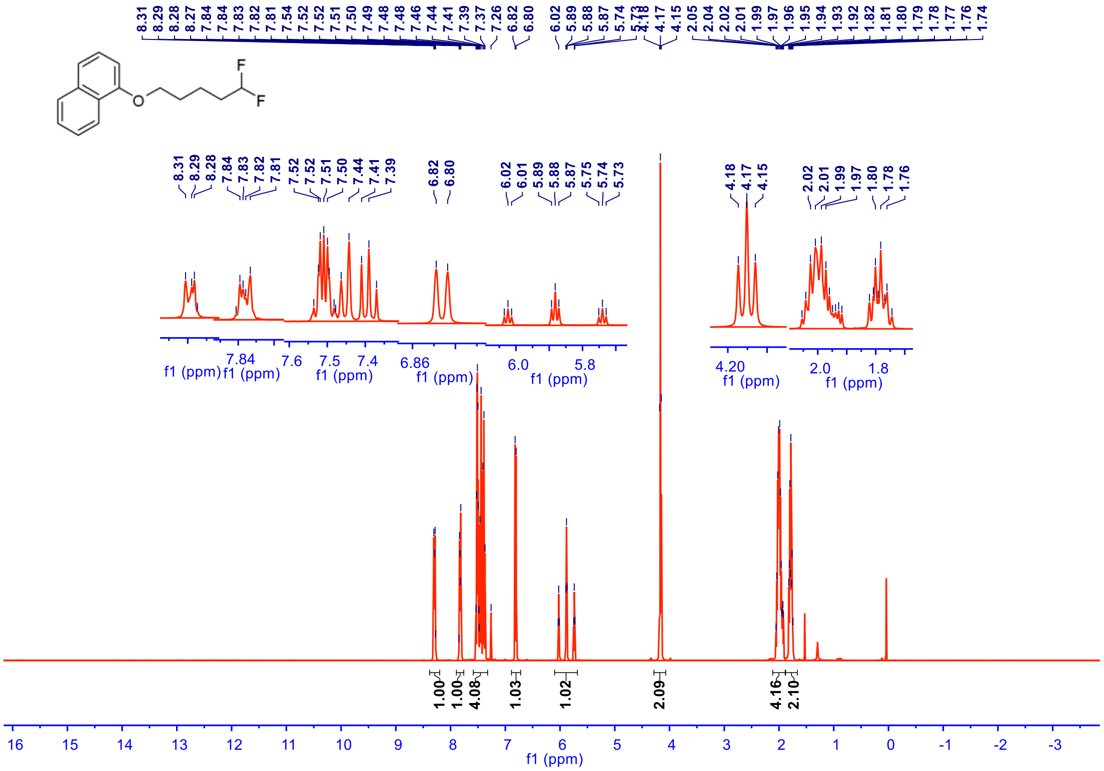

Supplementary Figure 270. ^{1}H NMR spectrum of compound **25** (400 MHz, $CDCl_3$)

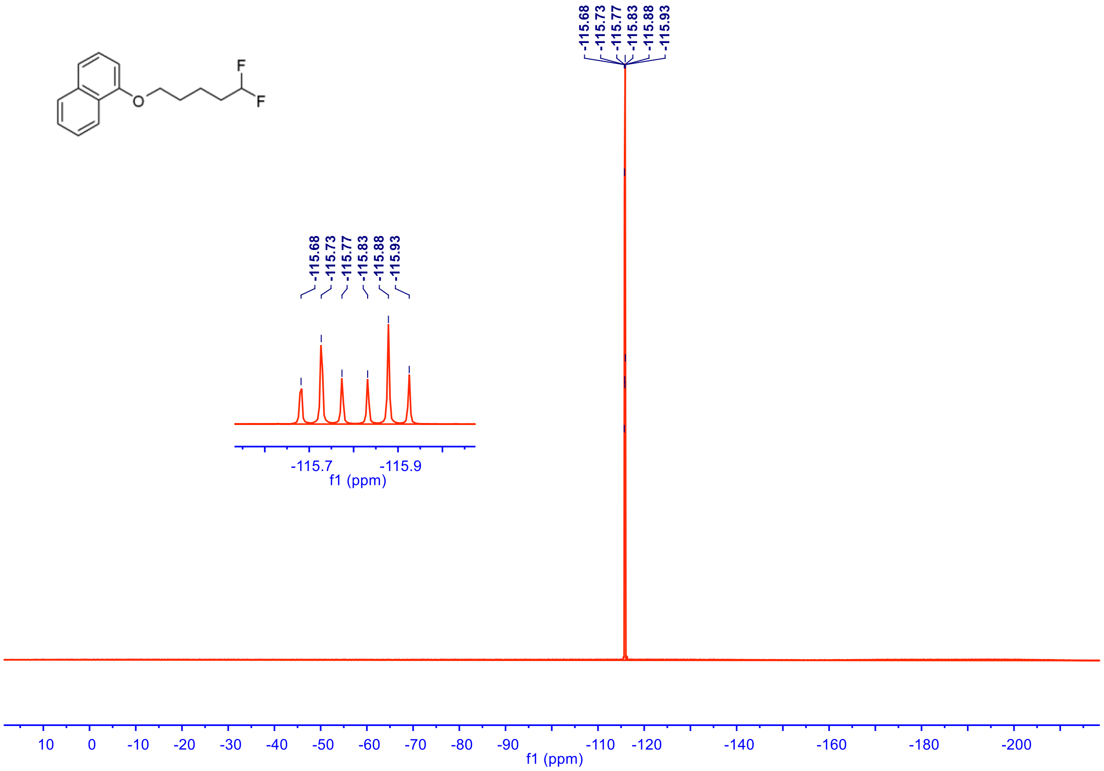

Supplementary Figure 271. ^{19}F NMR spectrum of compound **25** (376 MHz, $CDCl_3$)

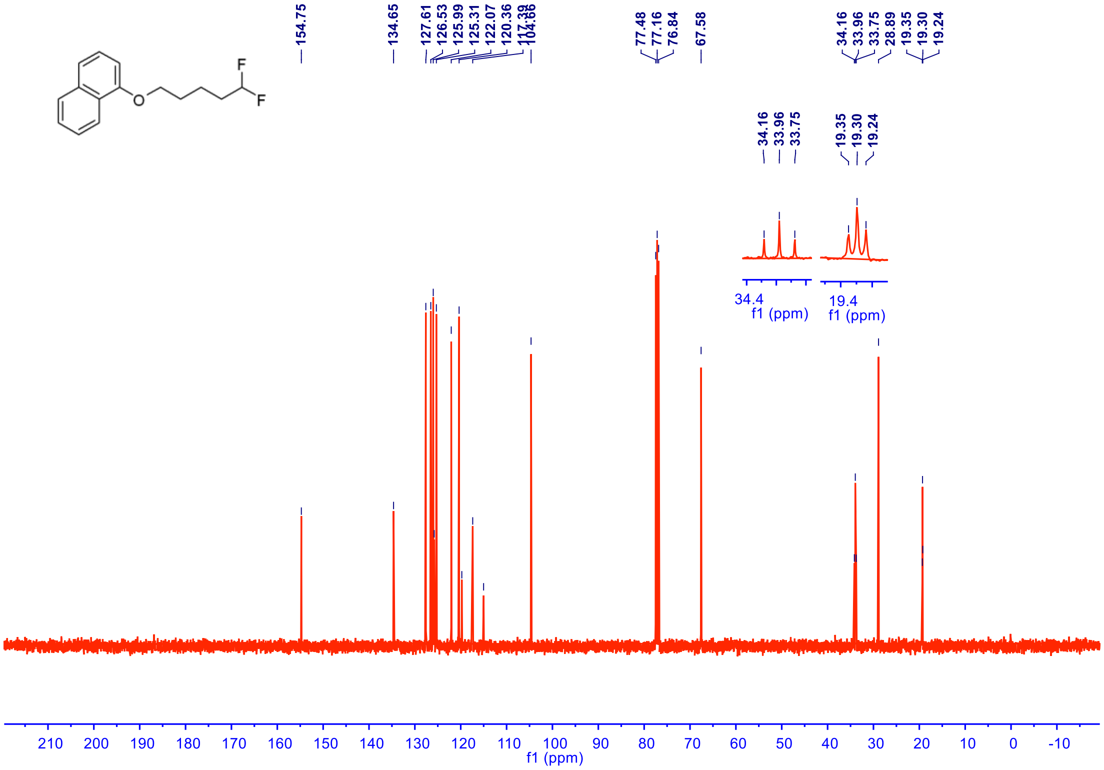

Supplementary Figure 272. ^{13}C NMR spectrum of compound **25** (101 MHz, $CDCl_3$)

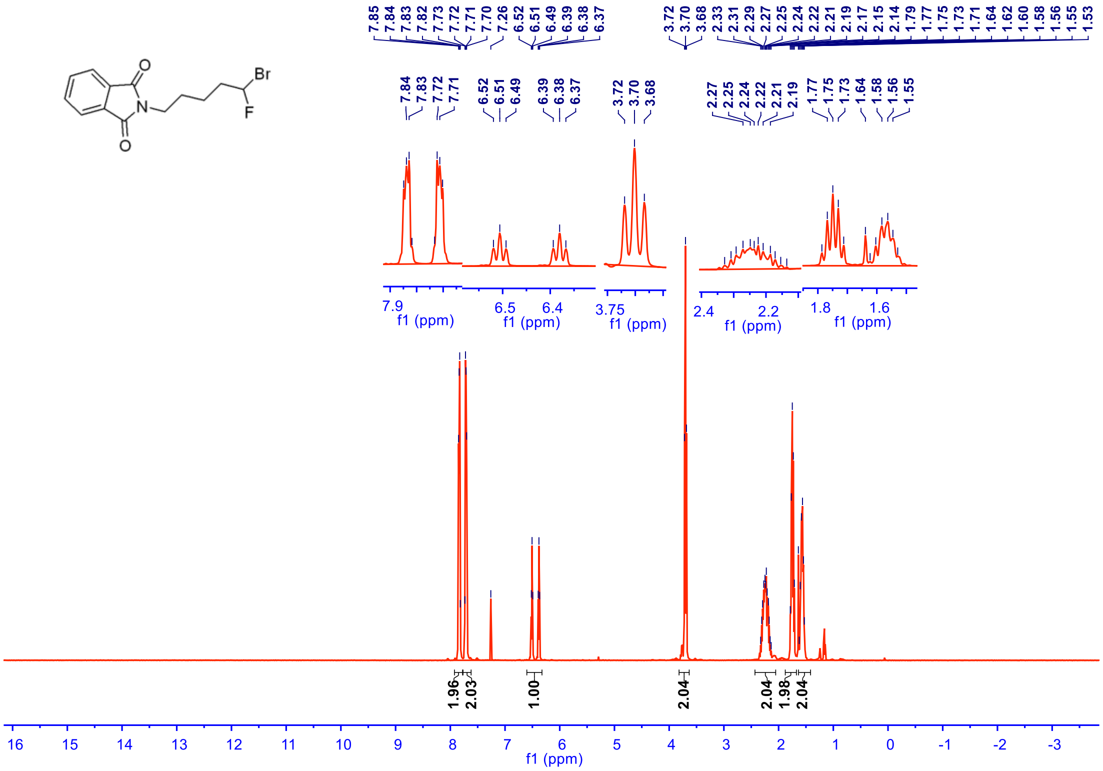

Supplementary Figure 273. ^{1}H NMR spectrum of **2-(5-bromo-5-fluoropentyl)isoindoline-1,3-dione 26a** (400 MHz, $CDCl_3$)

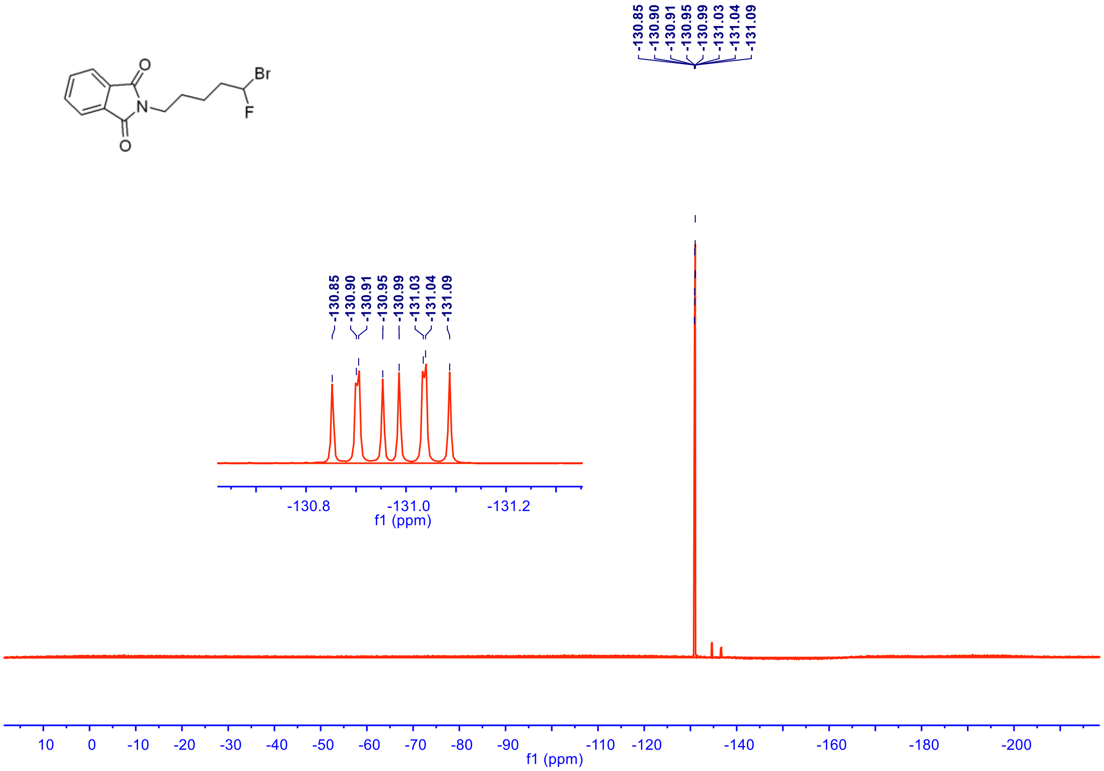

Supplementary Figure 274. ^{19}F NMR spectrum of **2-(5-bromo-5-fluoropentyl)isoindoline-1,3-dione 26a** (376 MHz, $CDCl_3$)

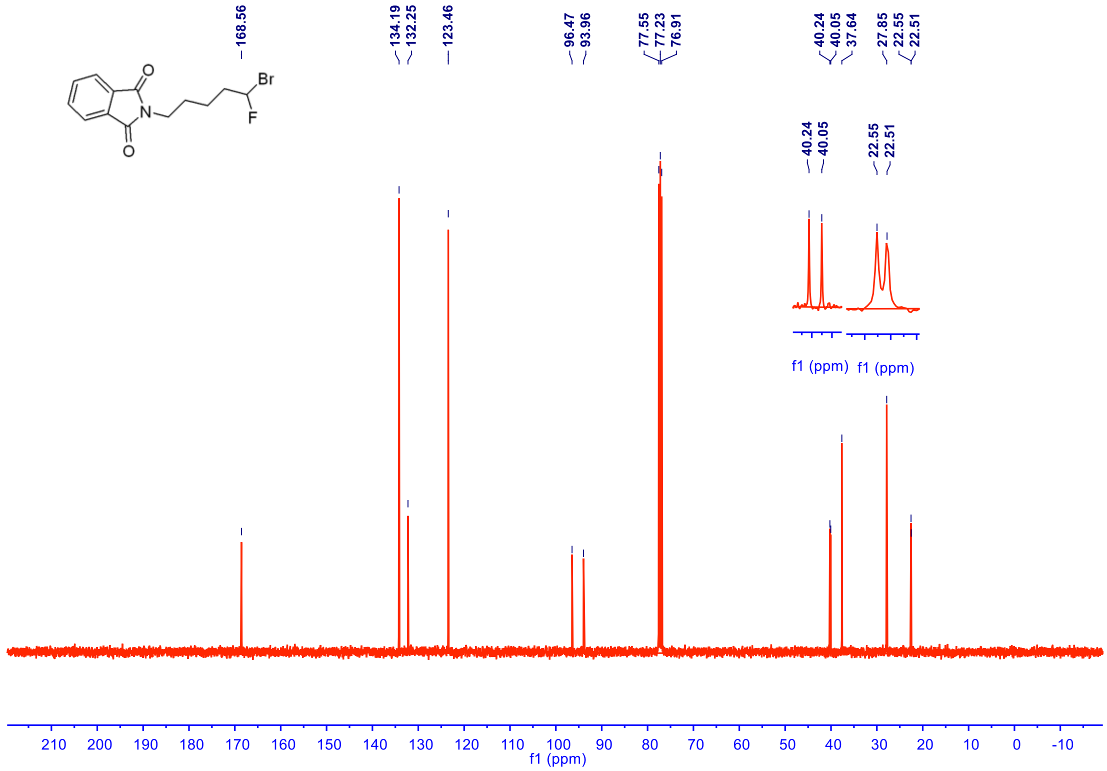

Supplementary Figure 275. ^{13}C NMR spectrum of **2-(5-bromo-5-fluoropentyl)isoindoline-1,3-dione 26a** (101 MHz, $CDCl_3$)

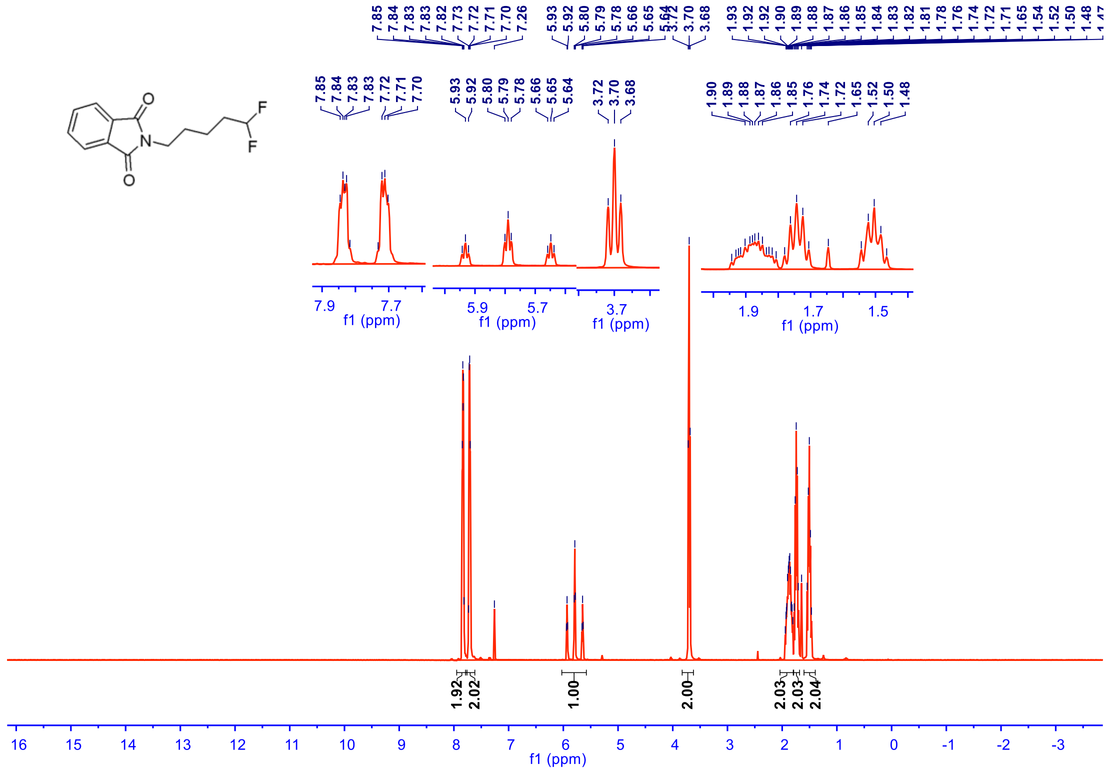

Supplementary Figure 276. ^{1}H NMR spectrum of compound **26** (400 MHz, $CDCl_3$)

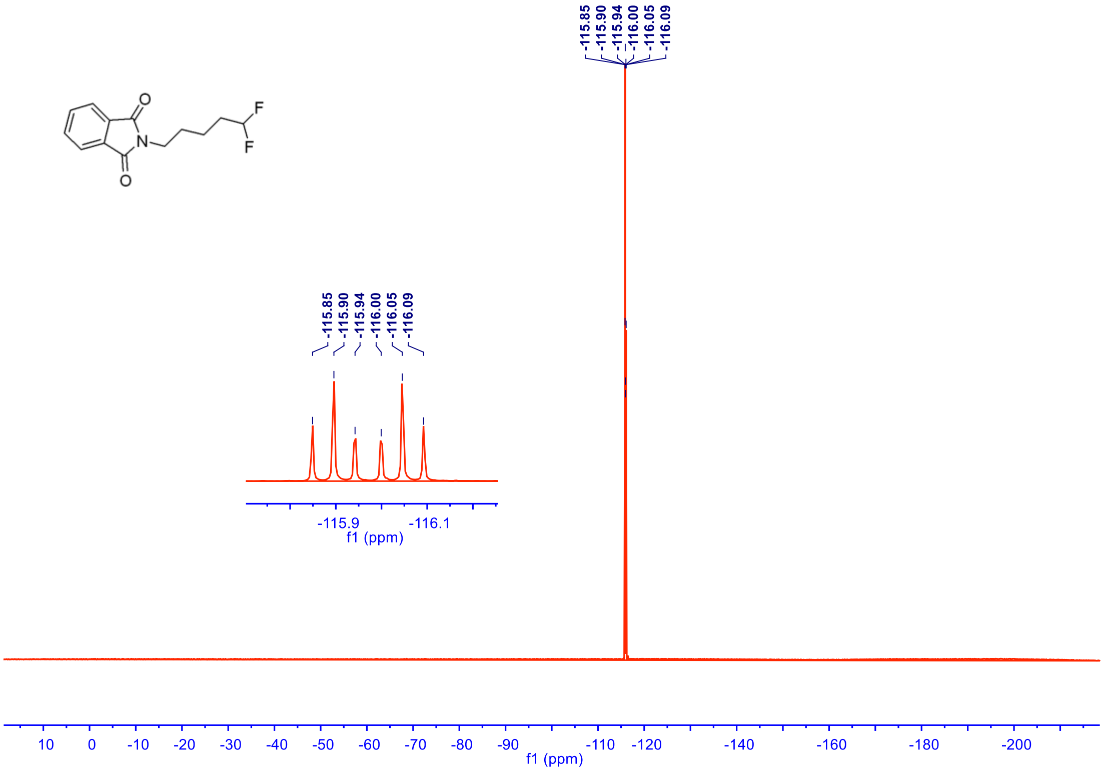

Supplementary Figure 277. ^{19}F NMR spectrum of compound **26** (376 MHz, $CDCl_3$)

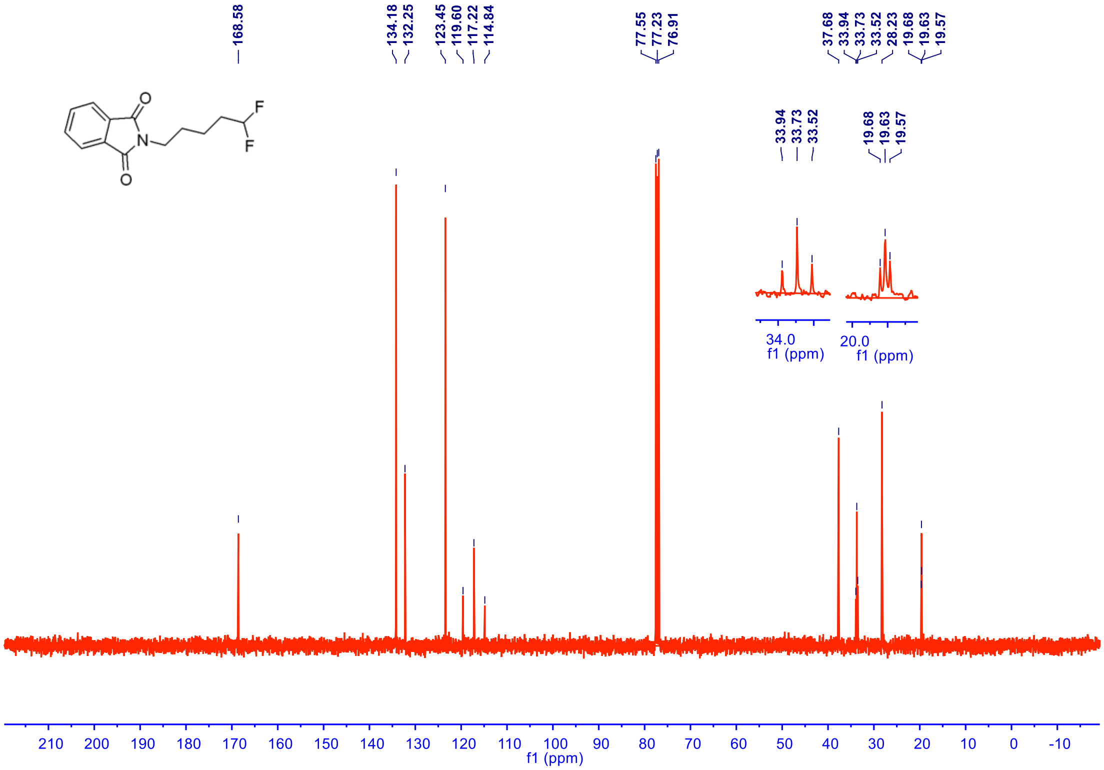

Supplementary Figure 278. ^{13}C NMR spectrum of compound **26** (101 MHz, $CDCl_3$)

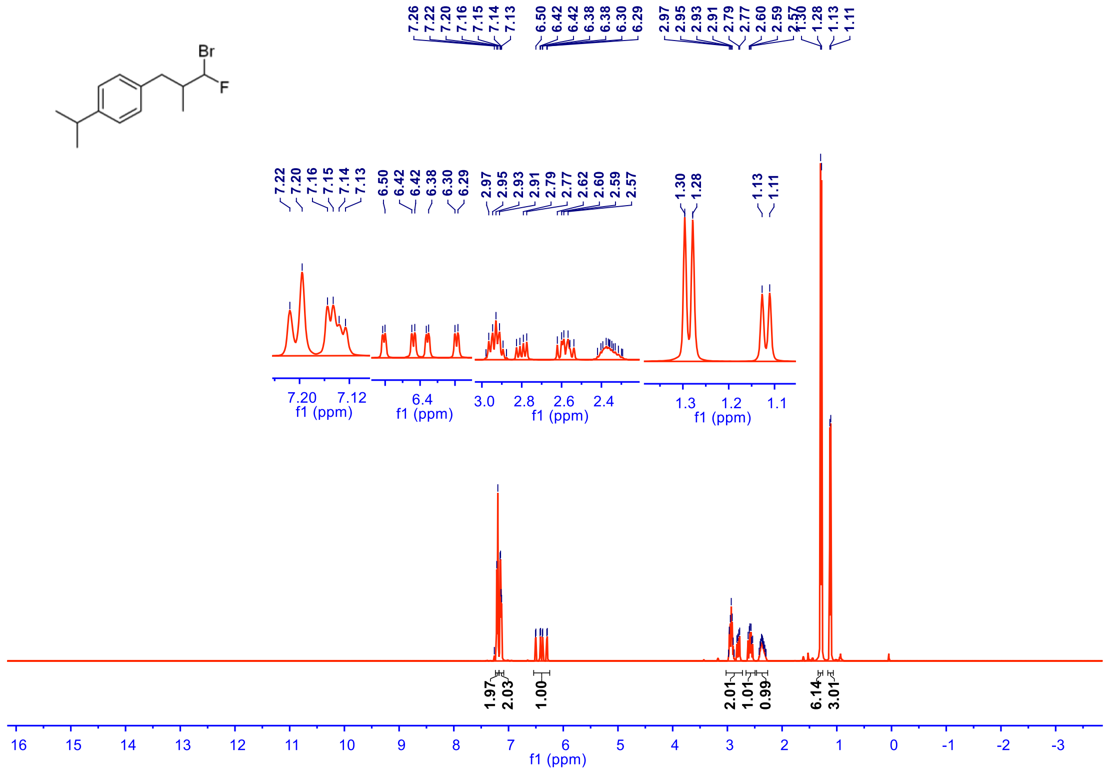

Supplementary Figure 279. ^{1}H NMR spectrum of **1-(3-bromo-3-fluoro-2-methylpropyl)-4-isopropylbenzene 27a** (400 MHz, $CDCl_3$)

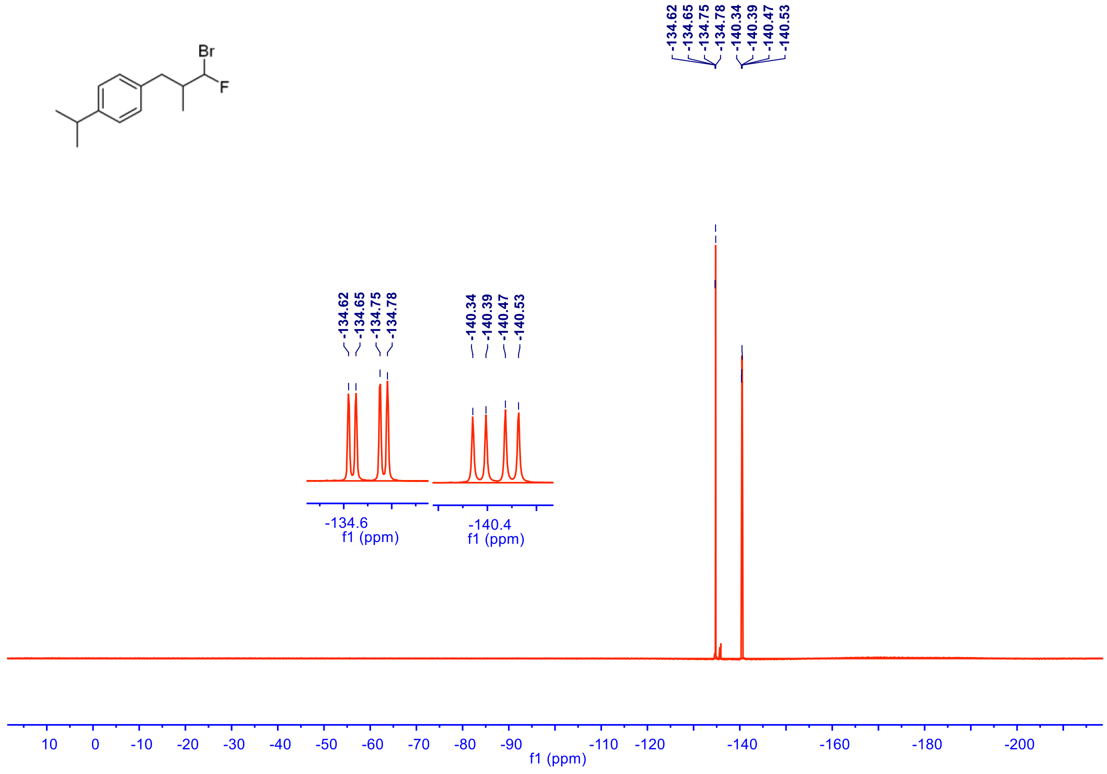

Supplementary Figure 280. ^{19}F NMR spectrum of **1-(3-bromo-3-fluoro-2-methylpropyl)-4-isopropylbenzene 27a** (376 MHz, $CDCl_3$)

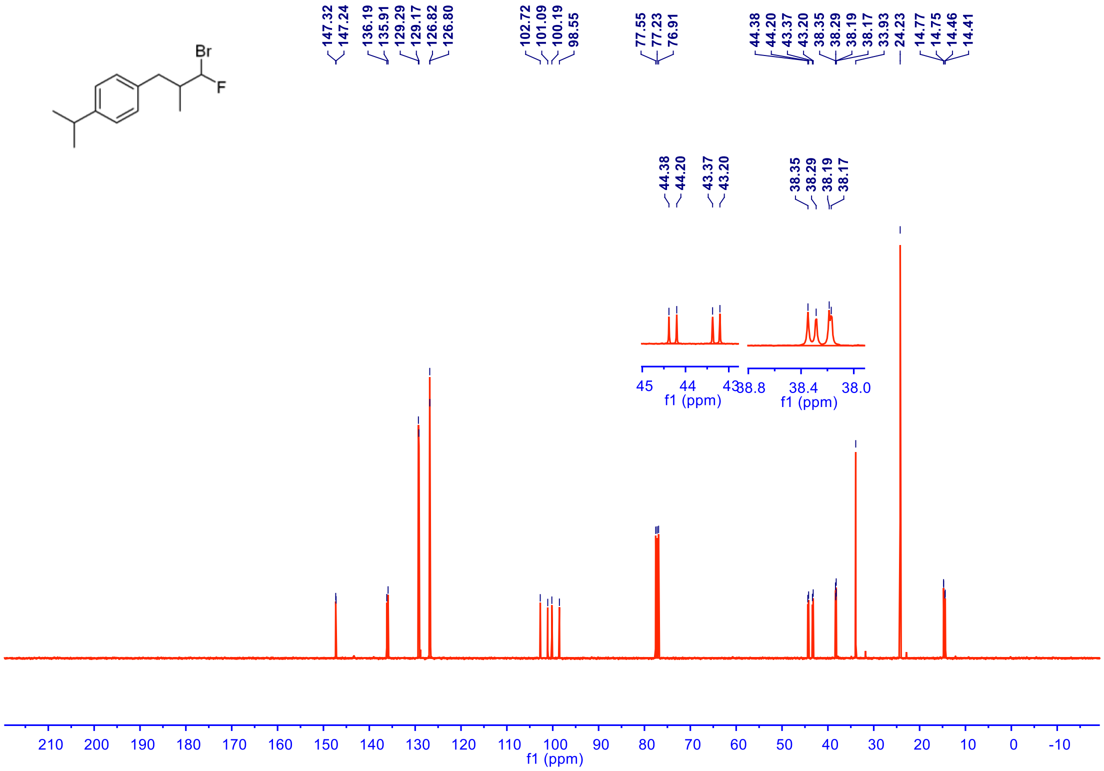

Supplementary Figure 281. ^{13}C NMR spectrum of **1-(3-bromo-3-fluoro-2-methylpropyl)-4-isopropylbenzene 27a** (101 MHz, $CDCl_3$)

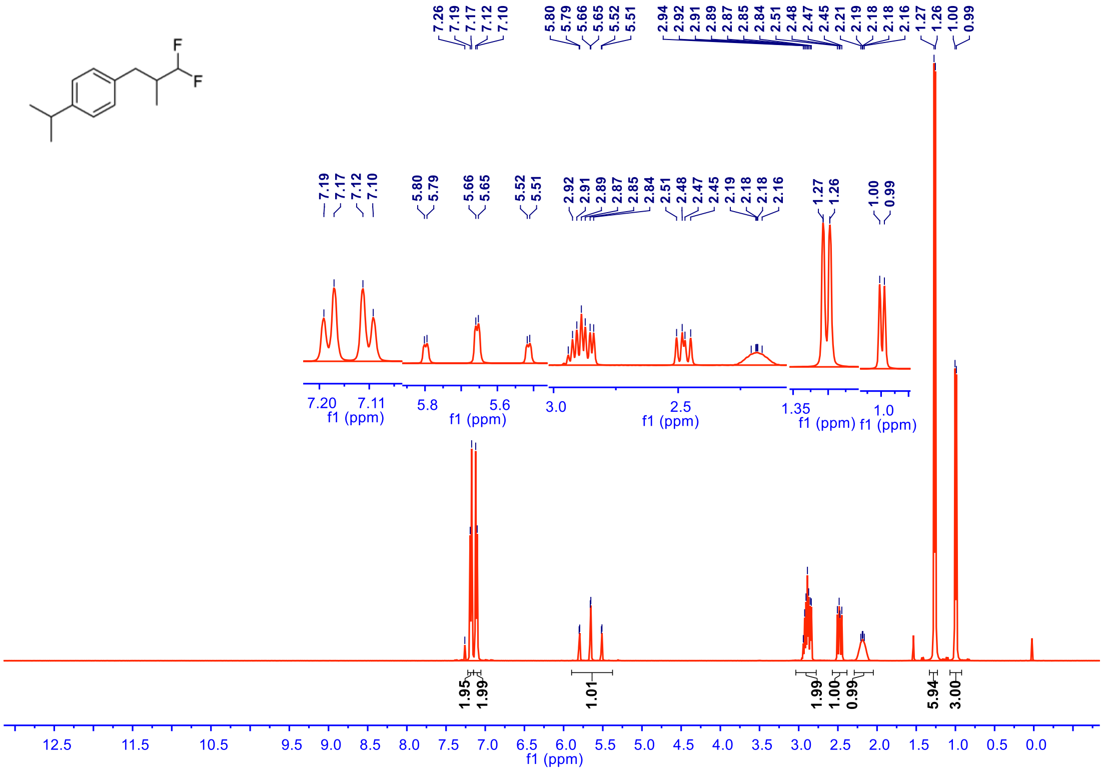

Supplementary Figure 282. ^{1}H NMR spectrum of compound **27** (400 MHz, $CDCl_3$)

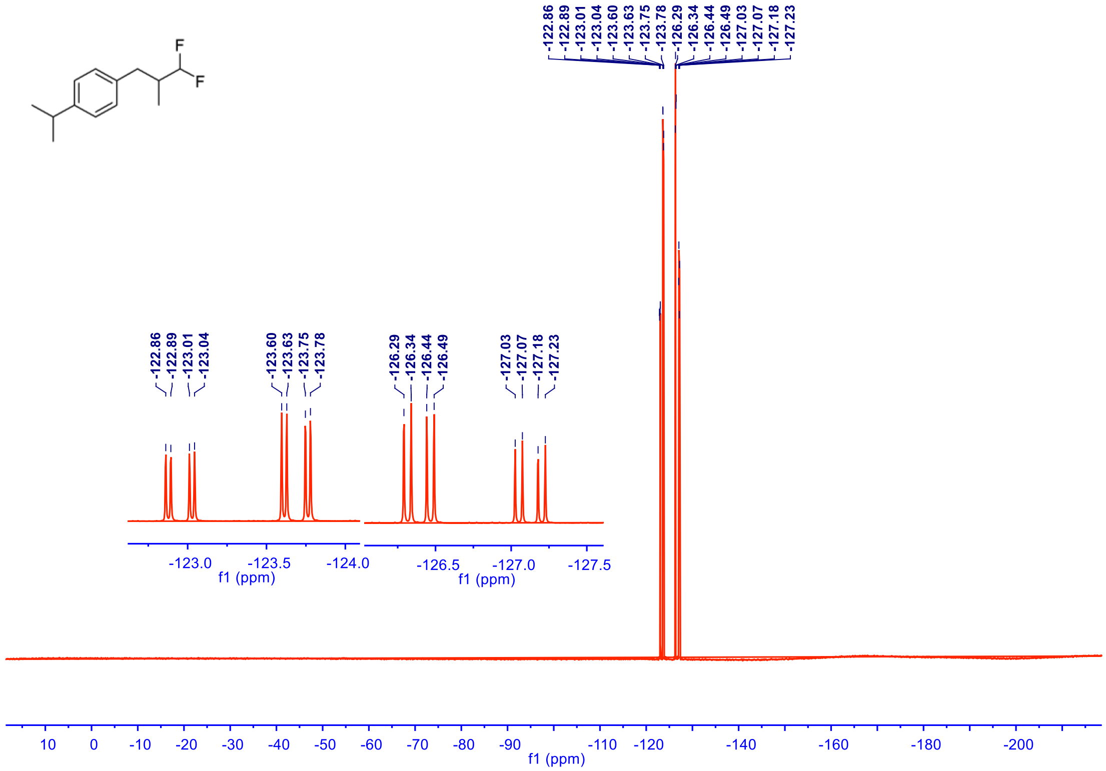

Supplementary Figure 283. ^{19}F NMR spectrum of compound **27** (376 MHz, $CDCl_3$)

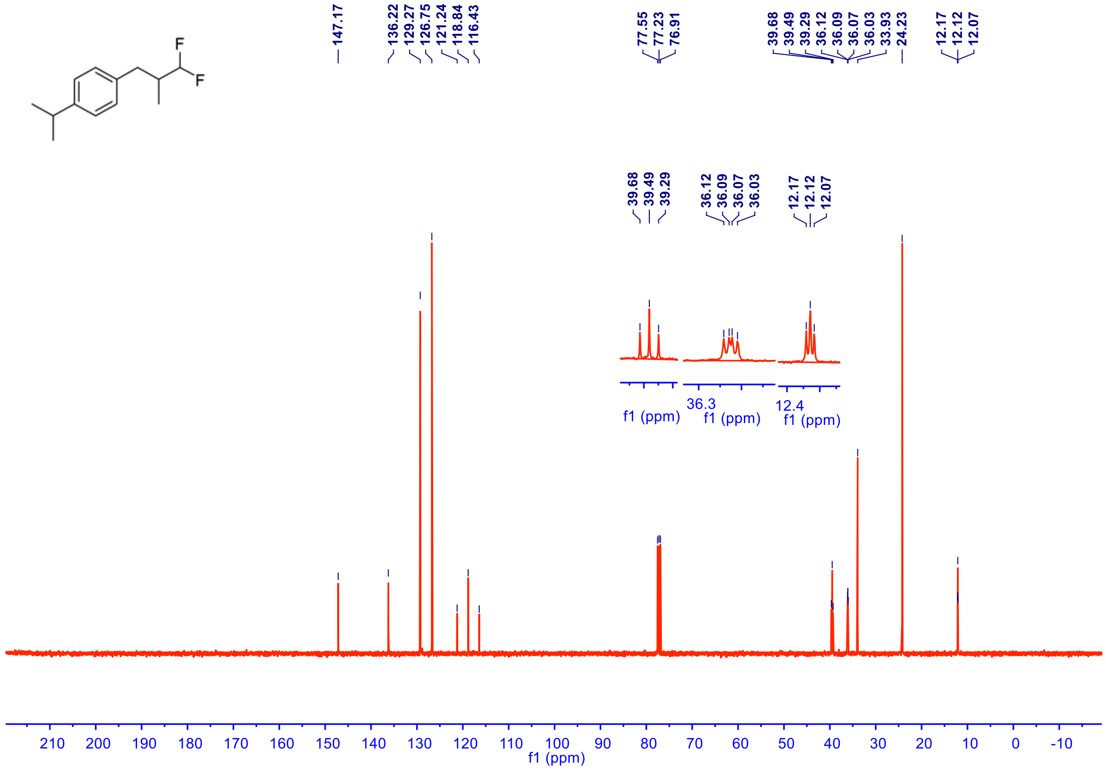

Supplementary Figure 284. ^{13}C NMR spectrum of compound **27** (101 MHz, $CDCl_3$)

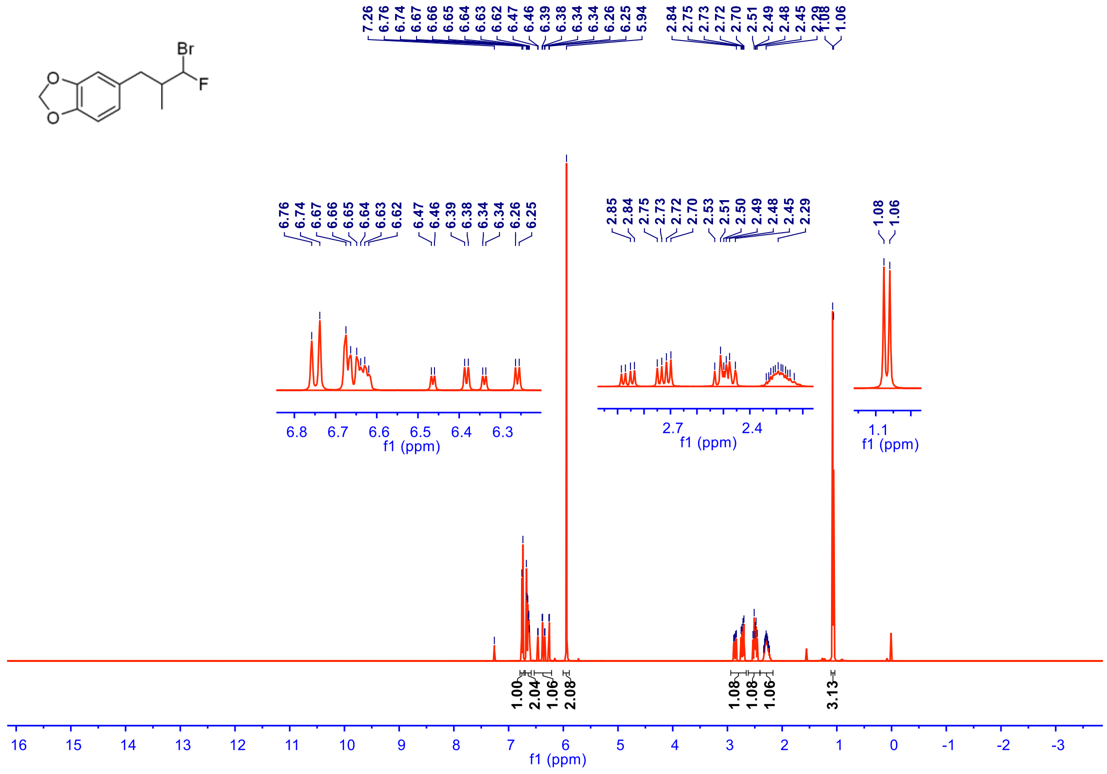

Supplementary Figure 285. ^{1}H NMR spectrum of **5-(3-bromo-3-fluoro-2-methylpropyl)benzo[*d*][1,3]dioxole 28a** (400 MHz, $CDCl_3$)

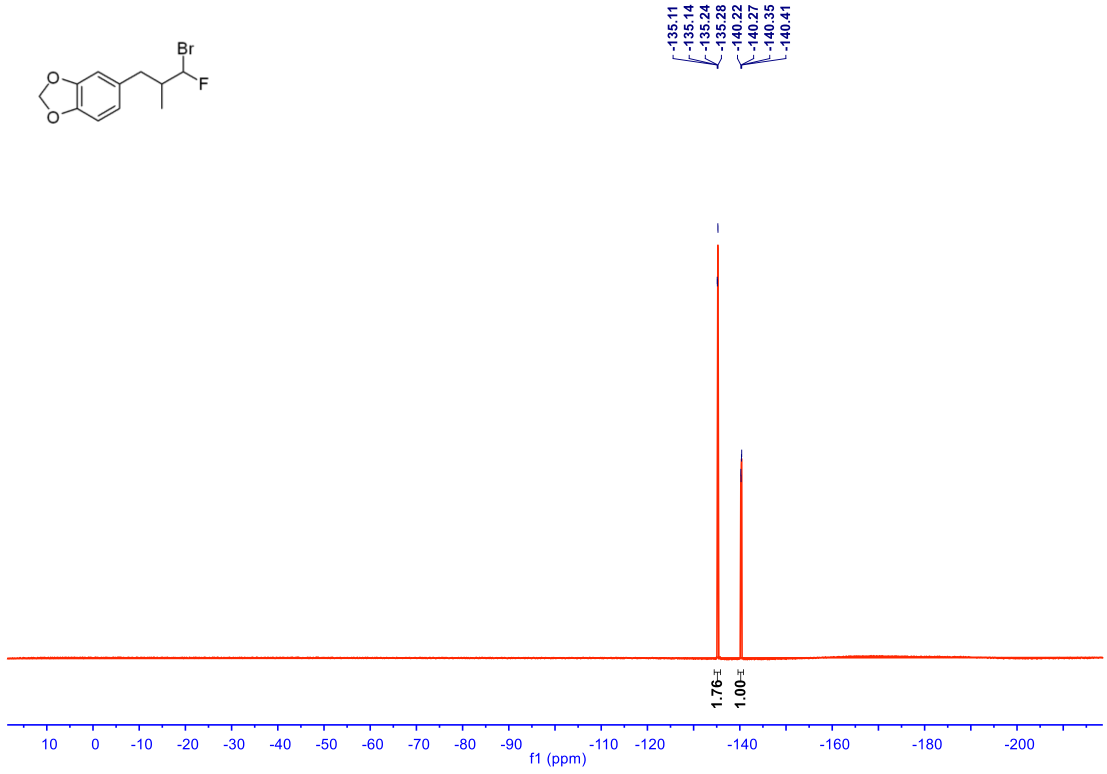

Supplementary Figure 286. ^{19}F NMR spectrum of **5-(3-bromo-3-fluoro-2-methylpropyl)benzo[*d*][1,3]dioxole 28a** (376 MHz, $CDCl_3$)

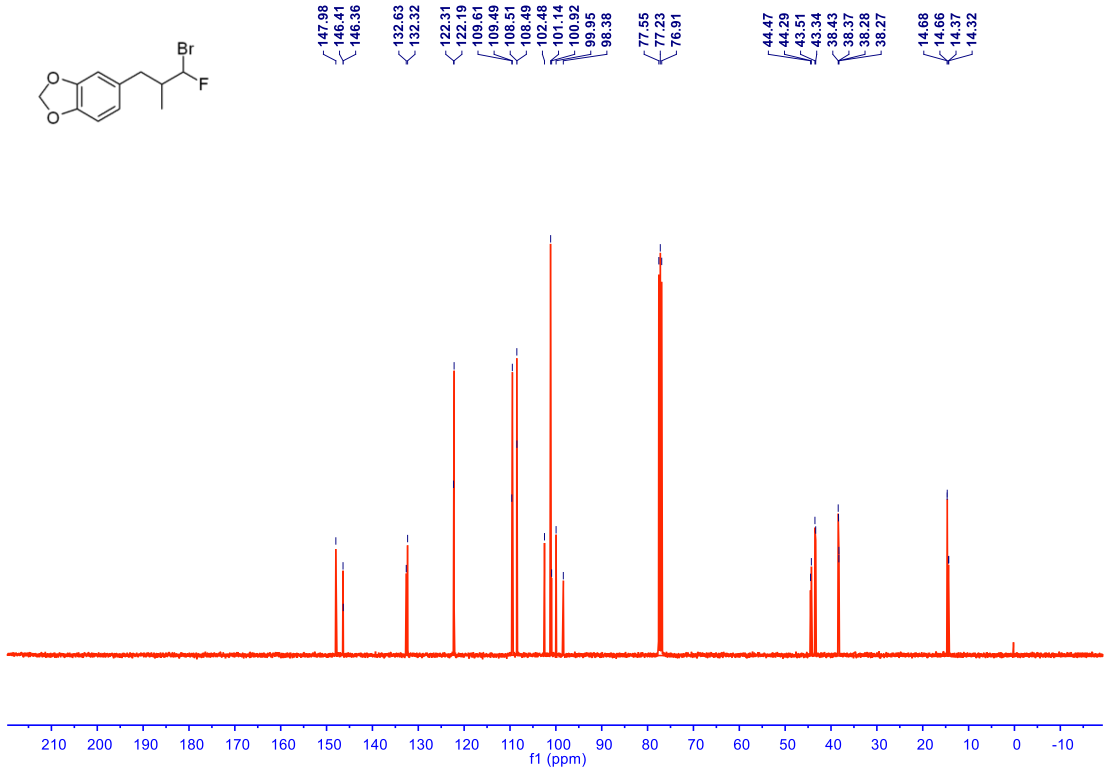

Supplementary Figure 287. ^{13}C NMR spectrum of **5-(3-bromo-3-fluoro-2-methylpropyl)benzo[*d*][1,3]dioxole 28a** (101 MHz, $CDCl_3$)

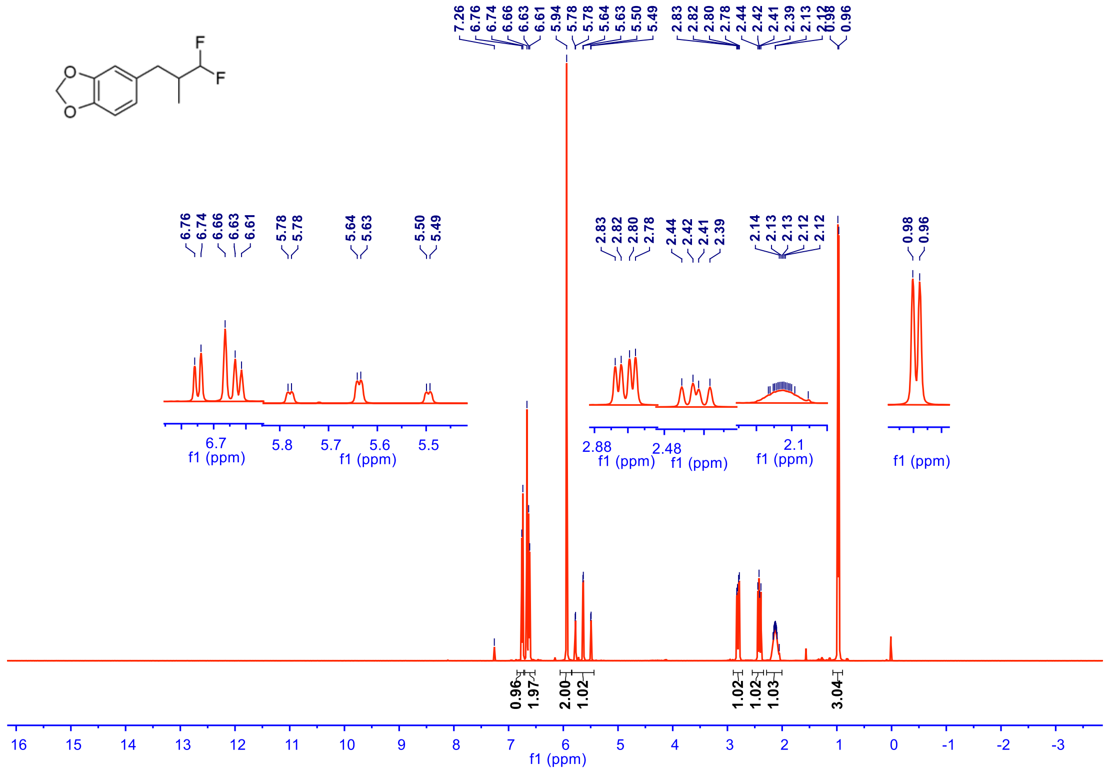

Supplementary Figure 288. ^{1}H NMR spectrum of compound **28** (400 MHz, $CDCl_3$)

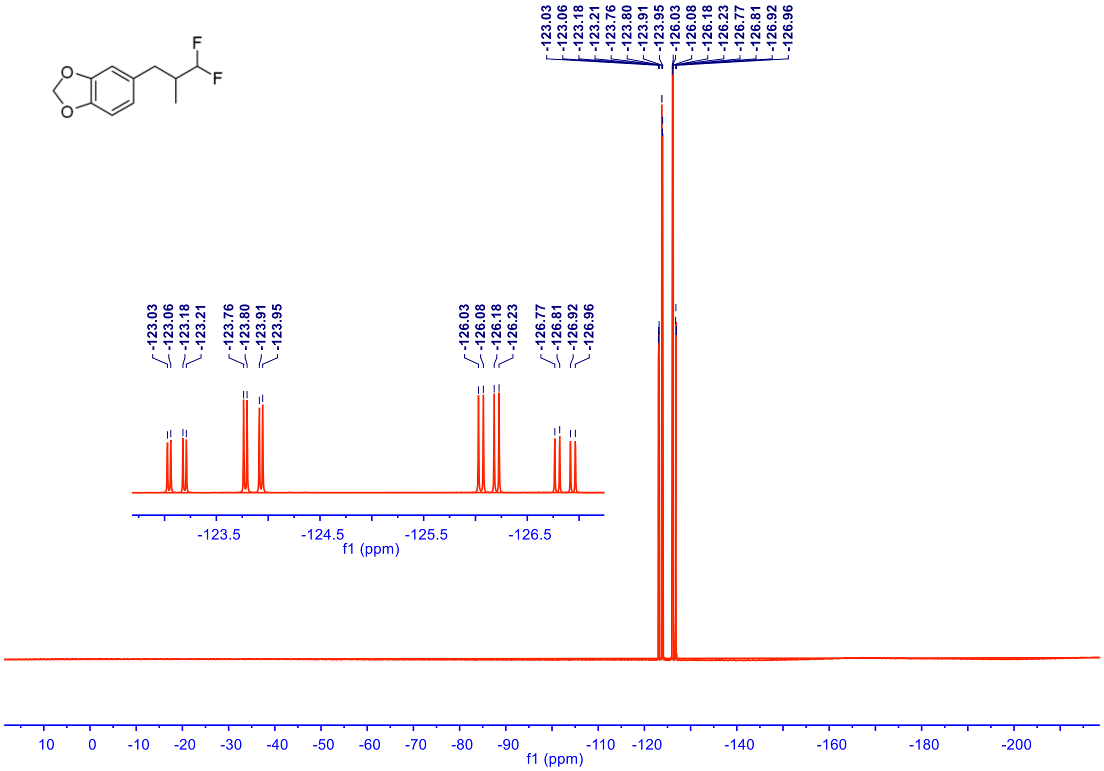

Supplementary Figure 289. ^{19}F NMR spectrum of compound **28** (376 MHz, $CDCl_3$)

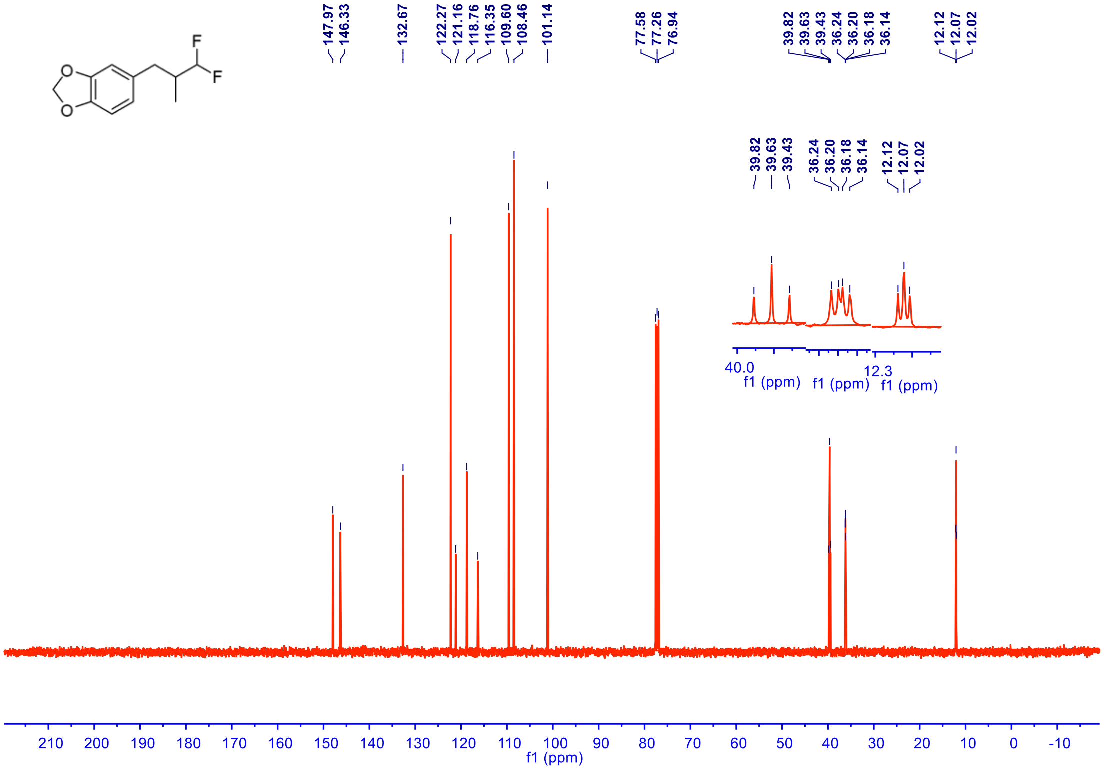

Supplementary Figure 290. ^{13}C NMR spectrum of compound **28** (101 MHz, $CDCl_3$)

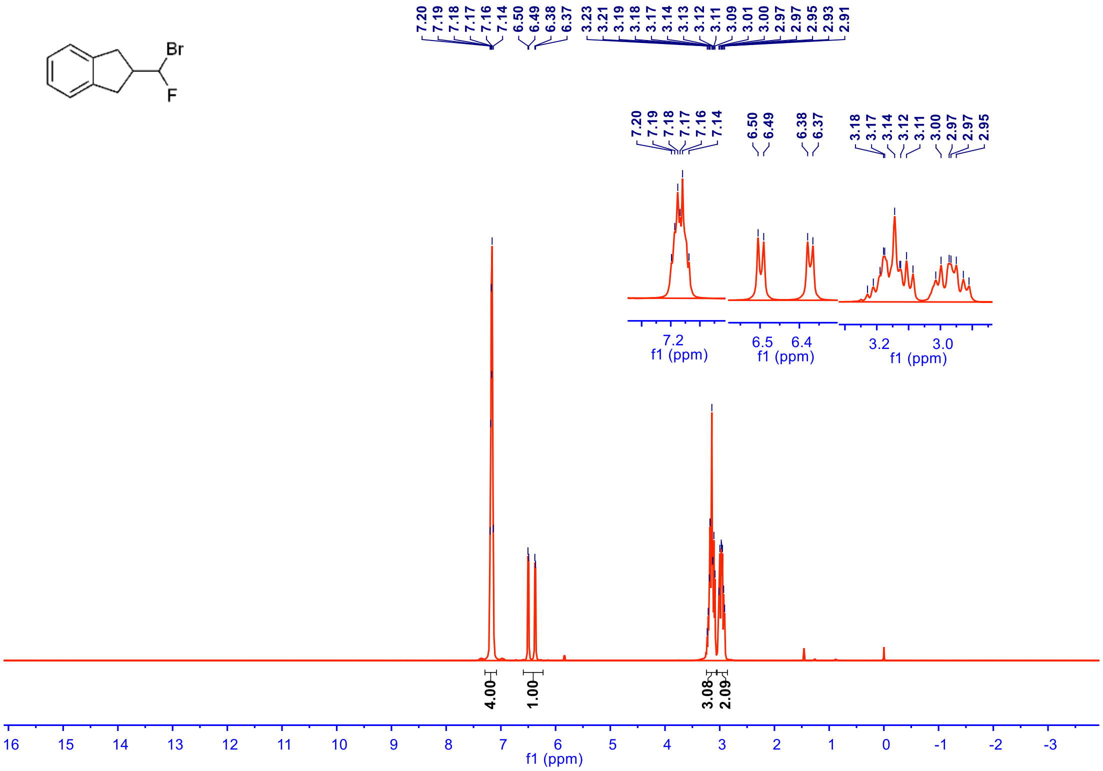

Supplementary Figure 291. ^{1}H NMR spectrum of compound **2-(bromofluoromethyl)-2,3-dihydro-1*H*-indene 29a** (400 MHz, $CDCl_3$)

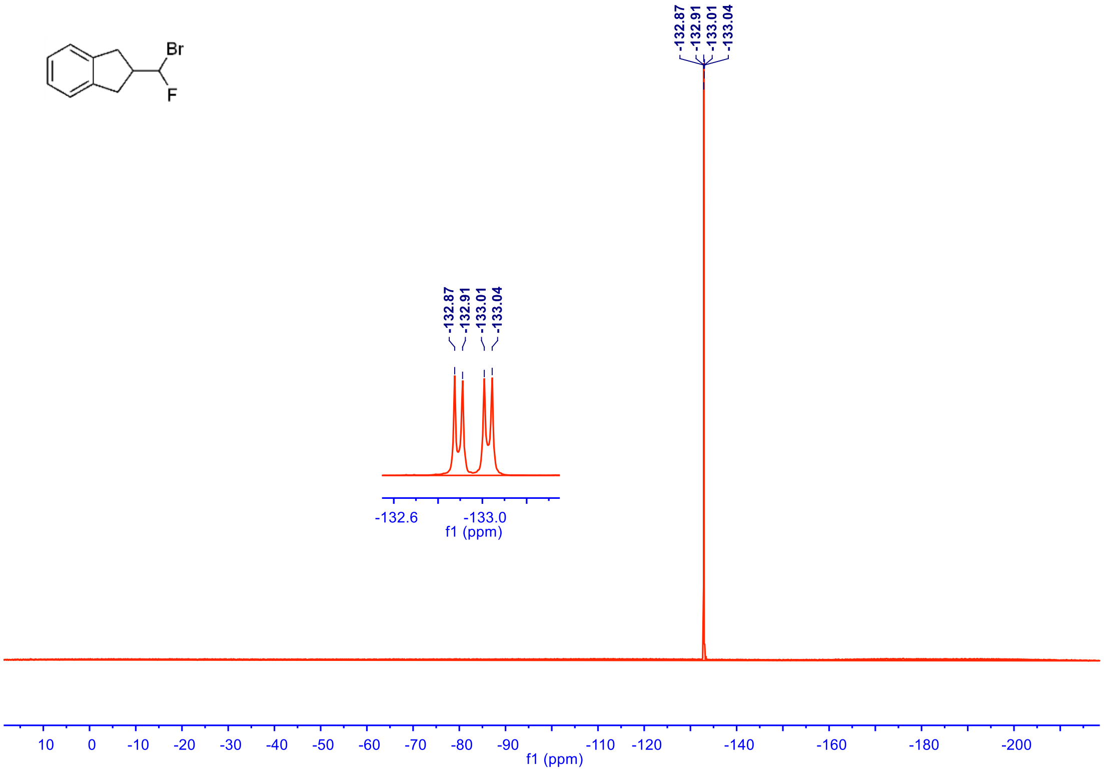

Supplementary Figure 292. ^{19}F NMR spectrum of compound **2-(bromofluoromethyl)-2,3-dihydro-1*H*-indene 29a** (376 MHz, $CDCl_3$)

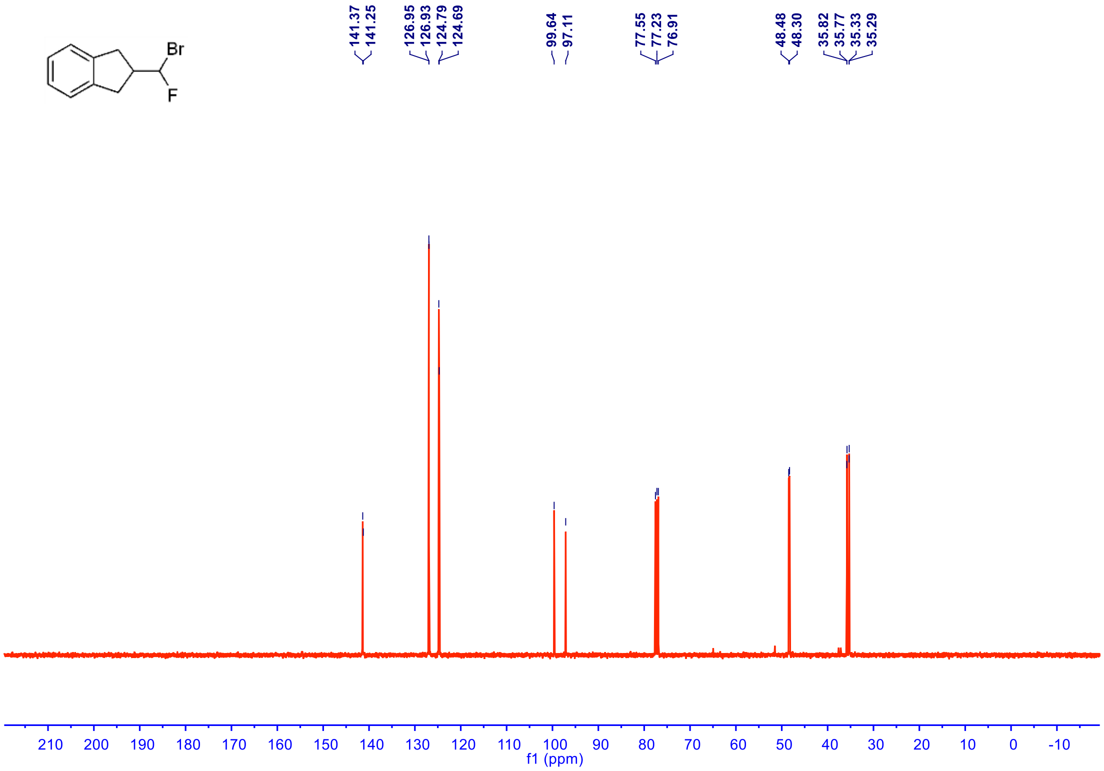

Supplementary Figure 293. ^{13}C NMR spectrum of **2-(bromofluoromethyl)-2,3-dihydro-1*H*-indene 29a** (101 MHz, $CDCl_3$)

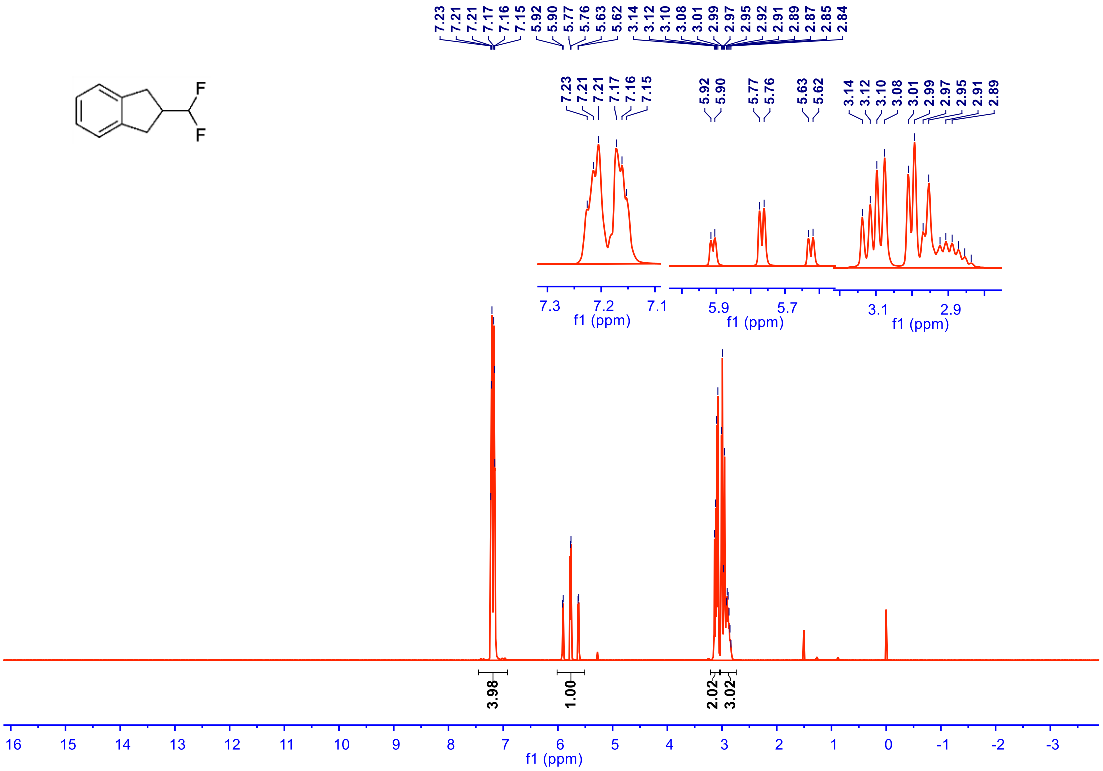

Supplementary Figure 294. ^{1}H NMR spectrum of compound **29** (400 MHz, $CDCl_3$)

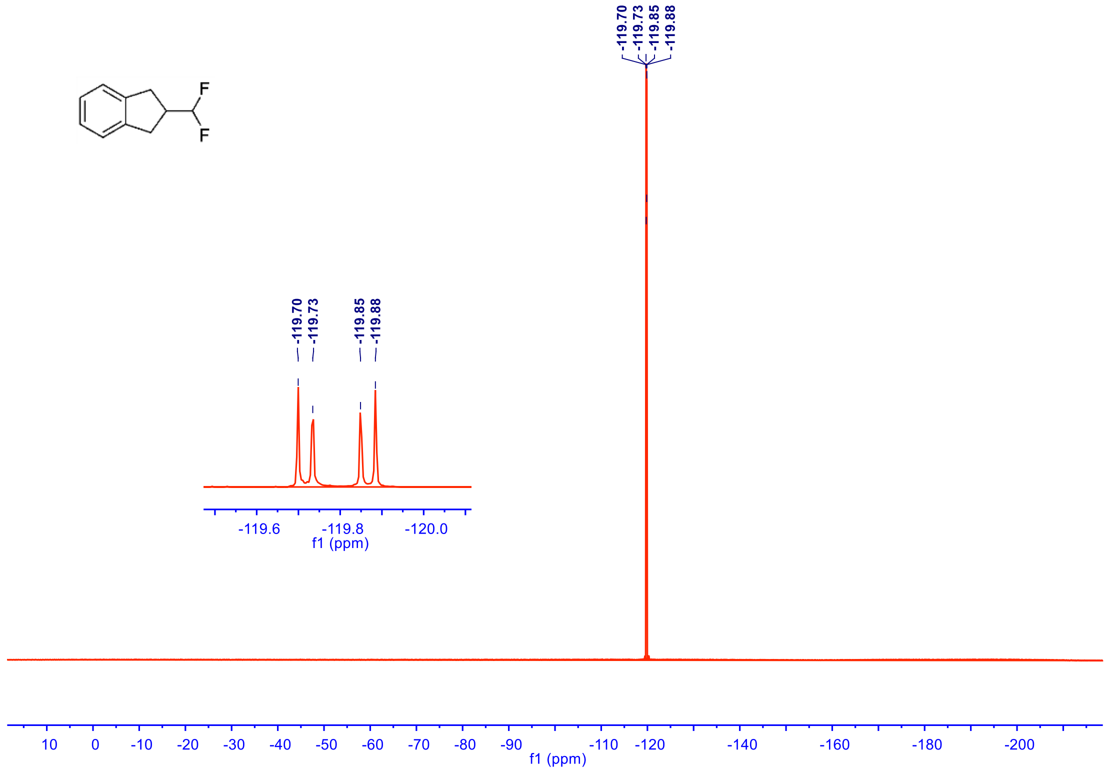

Supplementary Figure 295. ^{19}F NMR spectrum of compound **29** (376 MHz, $CDCl_3$)

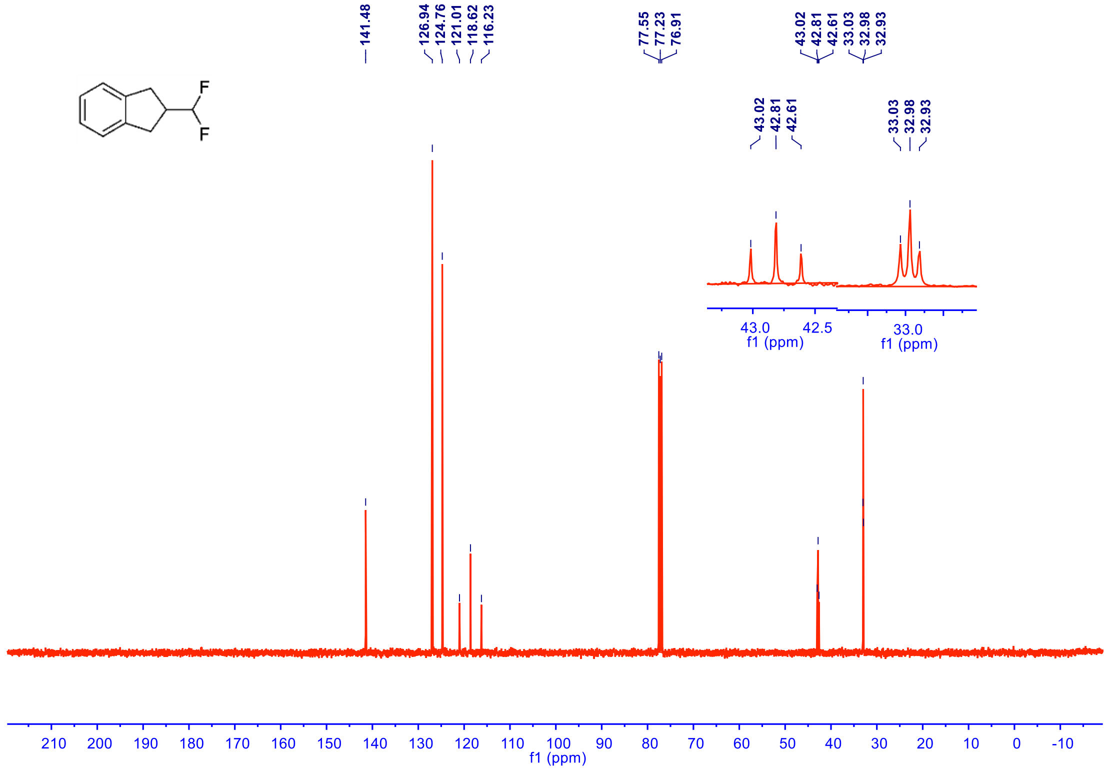

Supplementary Figure 296. ^{13}C NMR spectrum of compound **29** (101 MHz, $CDCl_3$)

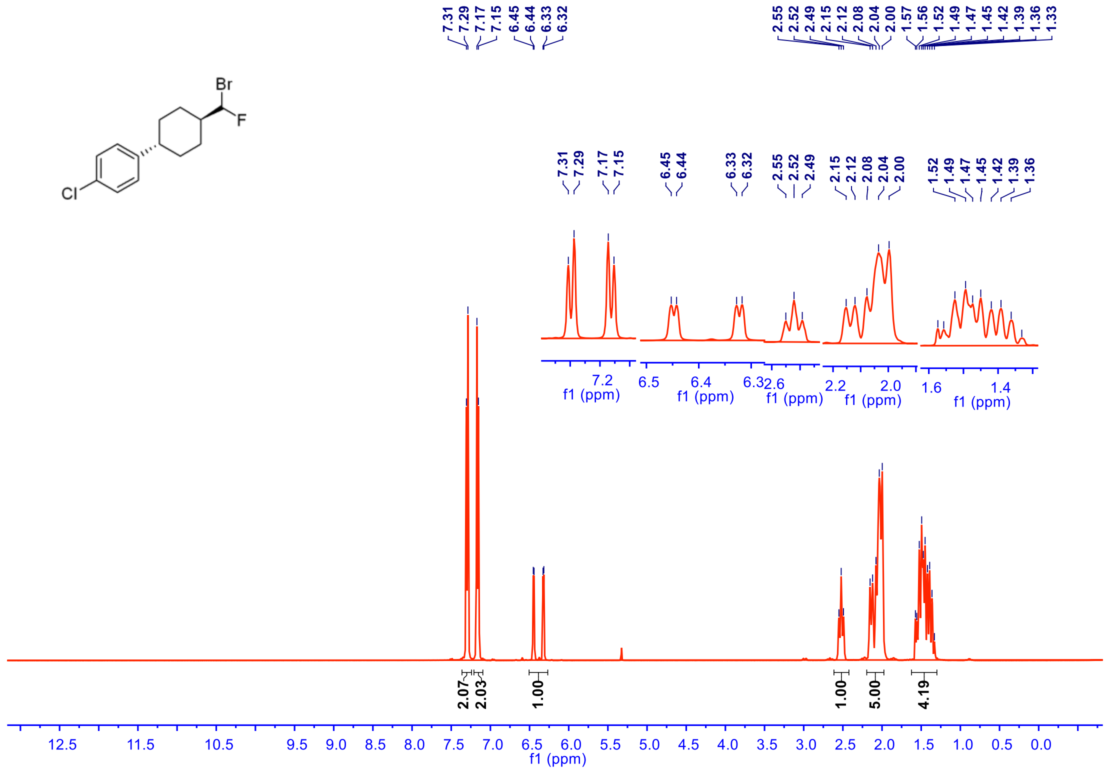

Supplementary Figure 297. ^{1}H NMR spectrum of **1-((1*S*,4*R*)-4-((*S*)-bromofluoromethyl)cyclohexyl)-4-chlorobenzene 30a** (400 MHz, $CDCl_3$)

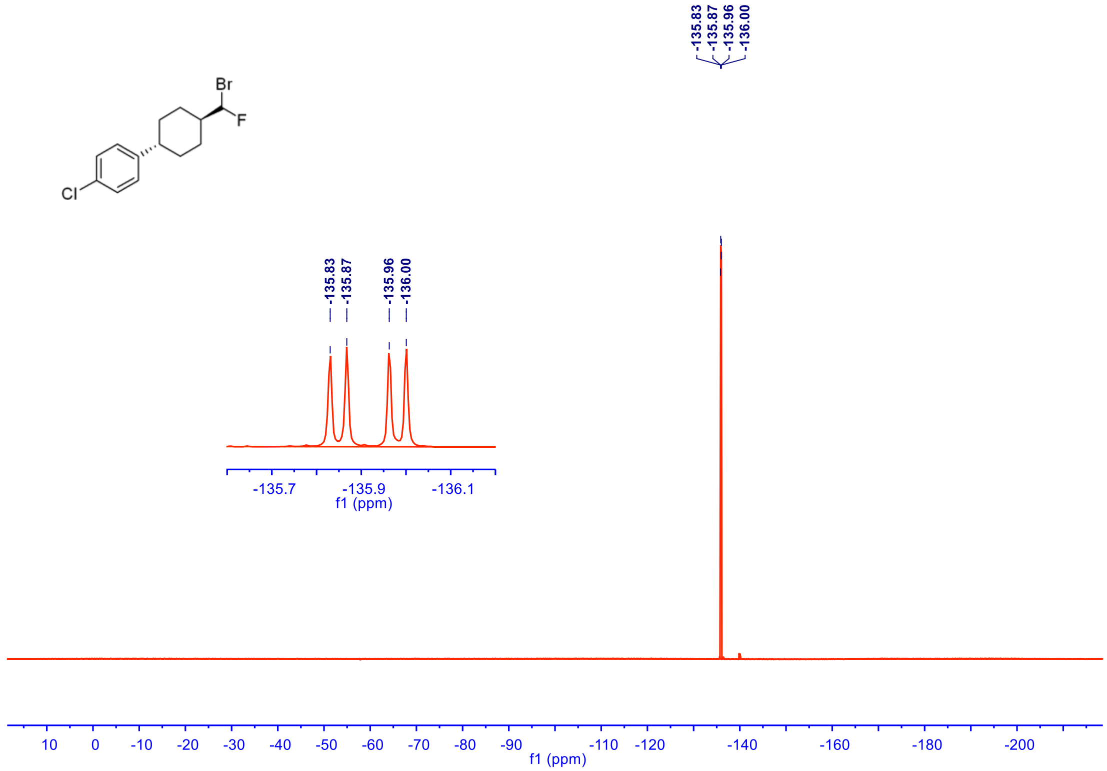

Supplementary Figure 298. ^{19}F NMR spectrum of **1-((1*S*,4*R*)-4-((*S*)-bromofluoromethyl)cyclohexyl)-4-chlorobenzene 30a** (376 MHz, $CDCl_3$)

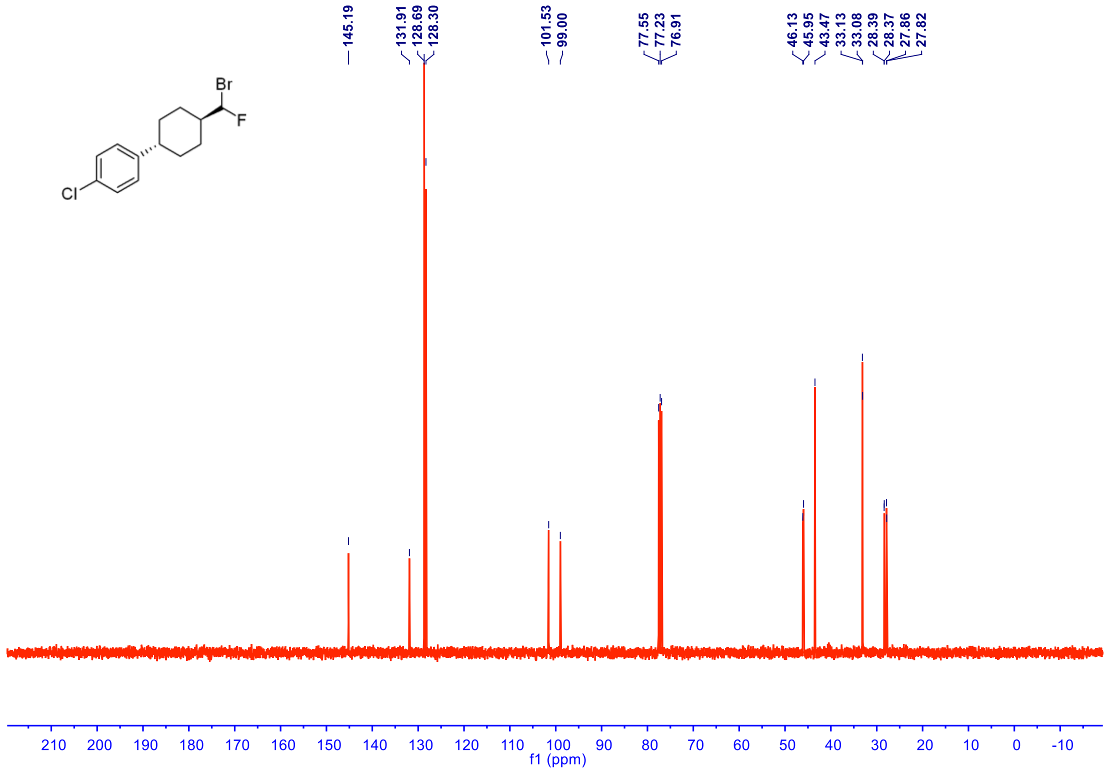

Supplementary Figure 299. ^{13}C NMR spectrum of **1-((1*S*,4*R*)-4-((*S*)-bromofluoromethyl)cyclohexyl)-4-chlorobenzene 30a** (101 MHz, $CDCl_3$)

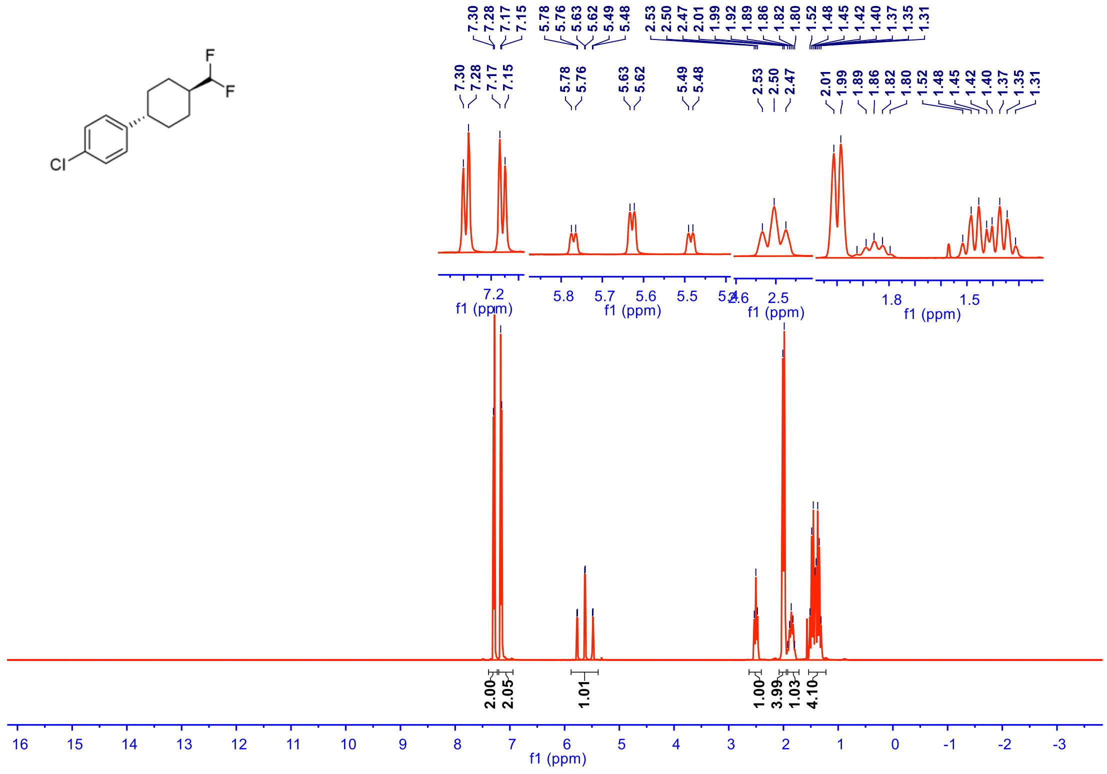

Supplementary Figure 300. ^{1}H NMR spectrum of compound **30** (400 MHz, $CDCl_3$)

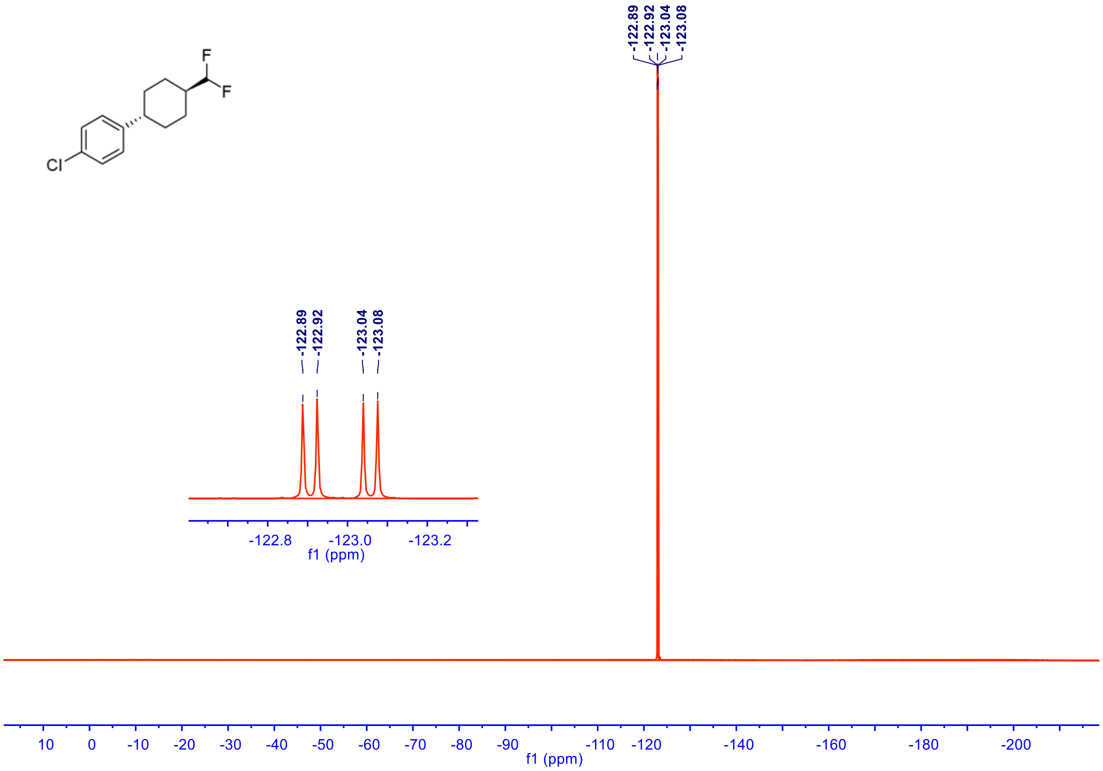

Supplementary Figure 301. ^{19}F NMR spectrum of compound **30** (376 MHz, $CDCl_3$)

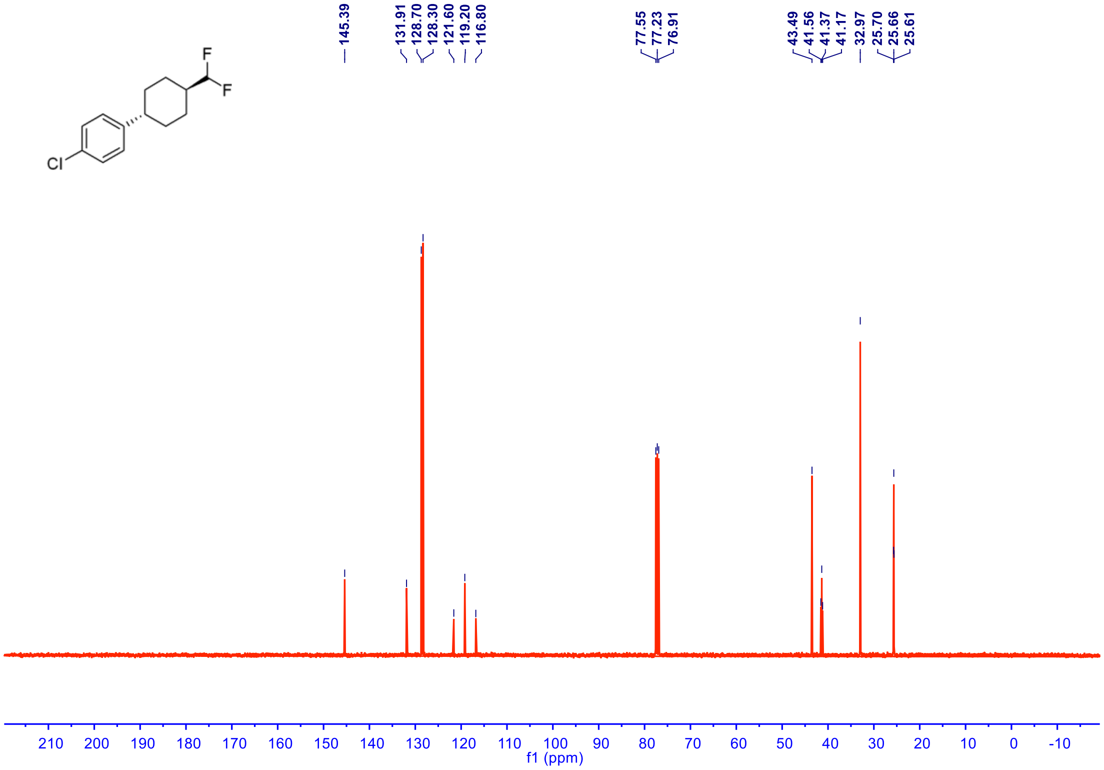

Supplementary Figure 302. ^{13}C NMR spectrum of compound **30** (101 MHz, $CDCl_3$)

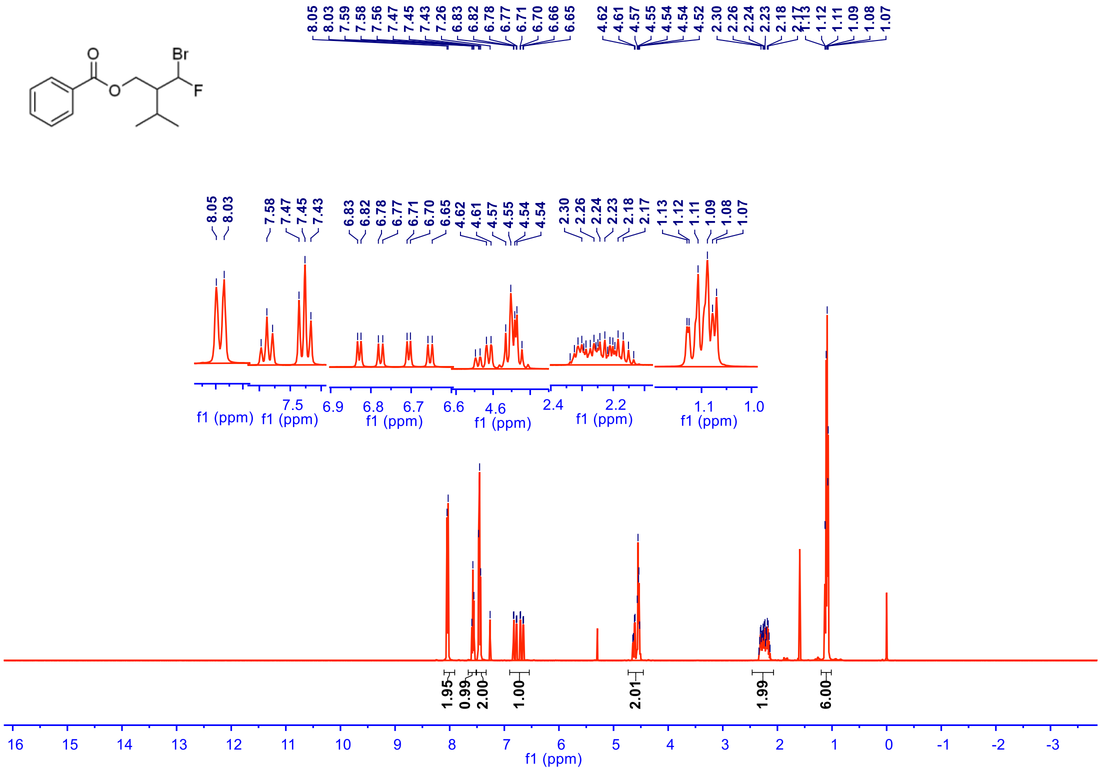

Supplementary Figure 303. ^{1}H NMR spectrum of **2-(bromofluoromethyl)-3-methylbutyl benzoate 31a** (400 MHz, $CDCl_3$)

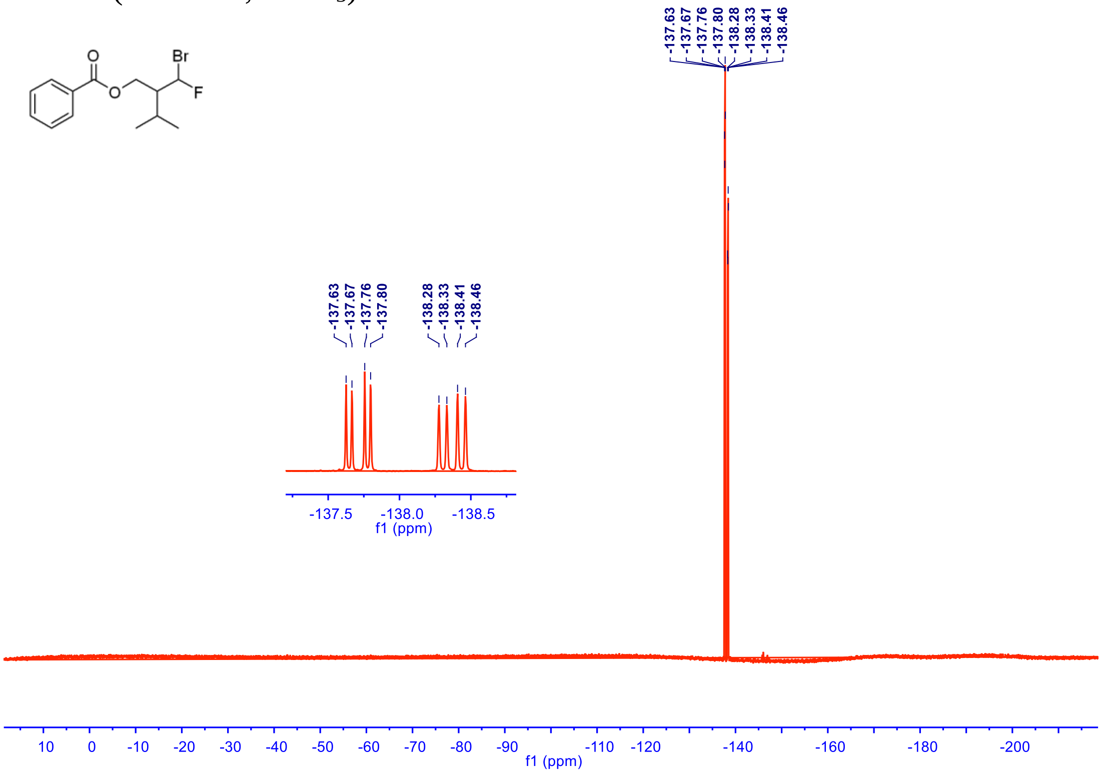

Supplementary Figure 304. ^{19}F NMR spectrum of **2-(bromofluoromethyl)-3-methylbutyl benzoate 31a** (376 MHz, $CDCl_3$)

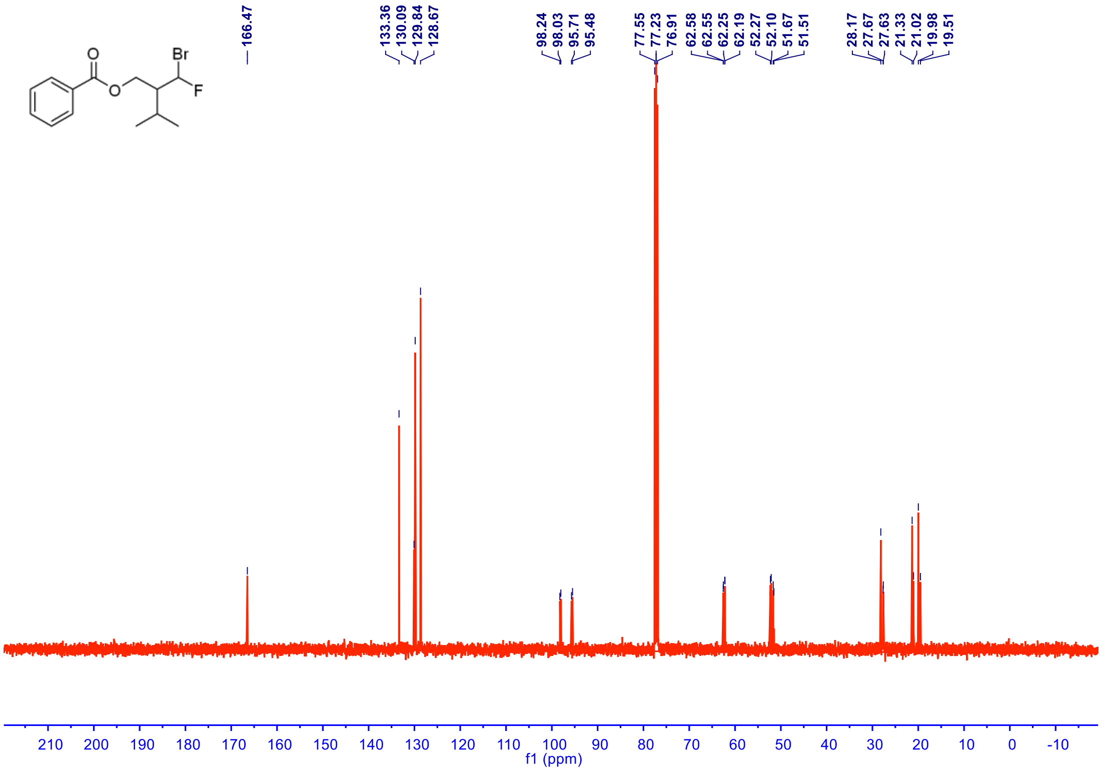

Supplementary Figure 305. ^{13}C NMR spectrum of **2-(bromofluoromethyl)-3-methylbutyl benzoate 31a** (101 MHz, $CDCl_3$)

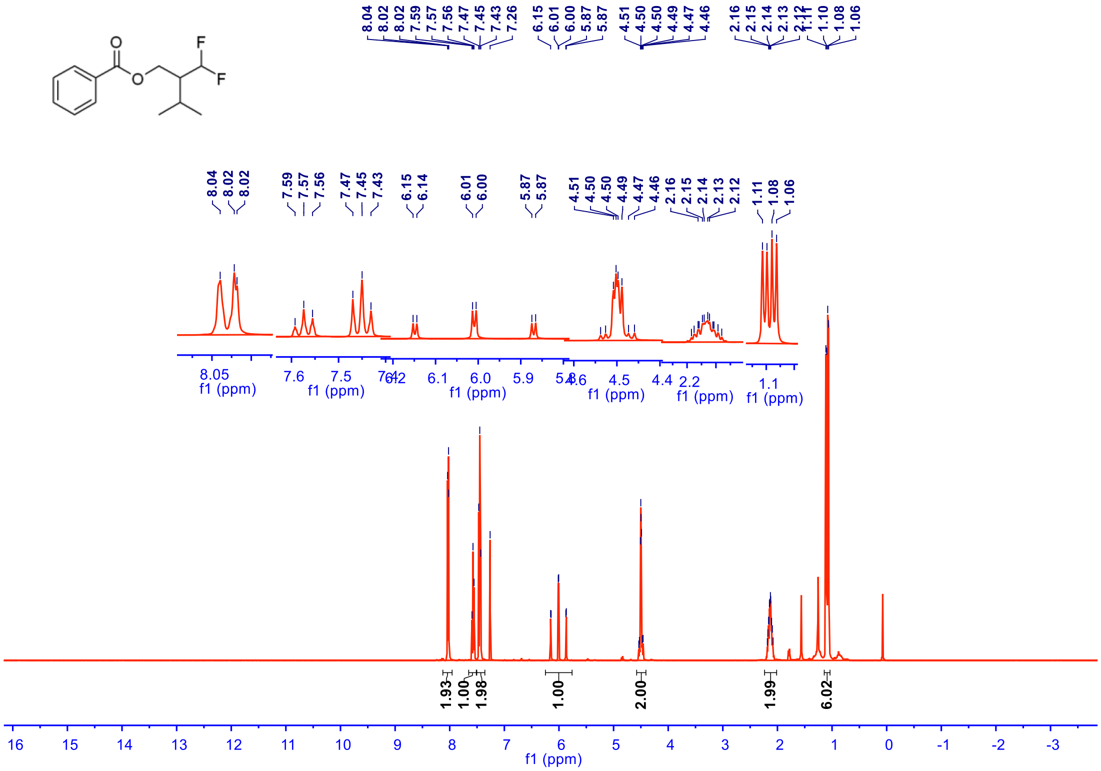

Supplementary Figure 306. ^{1}H NMR spectrum of compound **31** (400 MHz, $CDCl_3$)

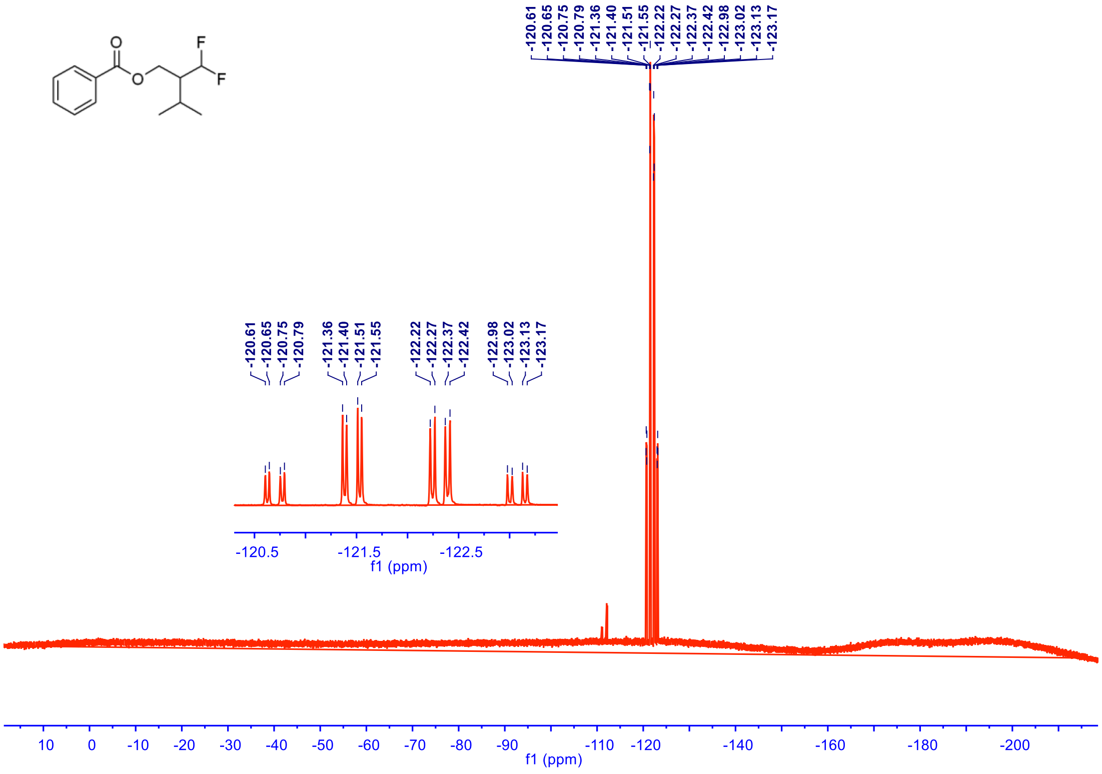

Supplementary Figure 307. ^{19}F NMR spectrum of compound **31** (376 MHz, $CDCl_3$)

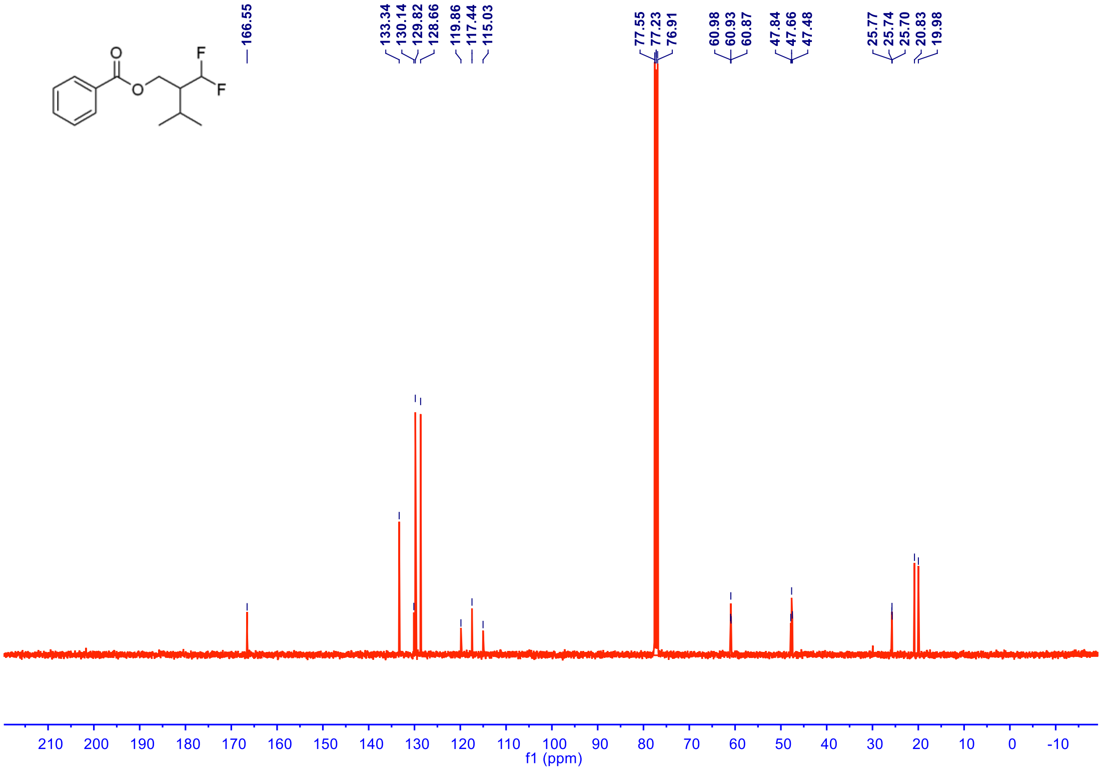

Supplementary Figure 308. ^{13}C NMR spectrum of compound **31** (101 MHz, $CDCl_3$)

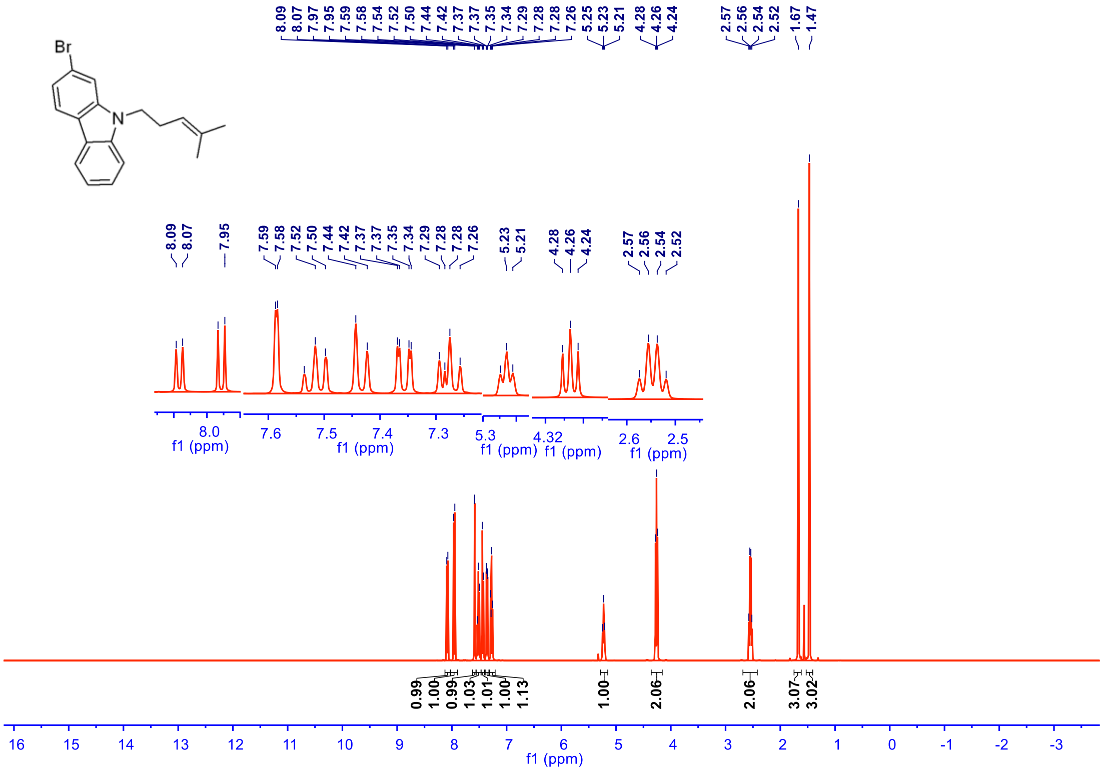

Supplementary Figure 309. ^{1}H NMR spectrum of **2-bromo-9-(4-methylpent-3-en-1-yl)-9*H*-carbazole 32b** (400 MHz, $CDCl_3$)

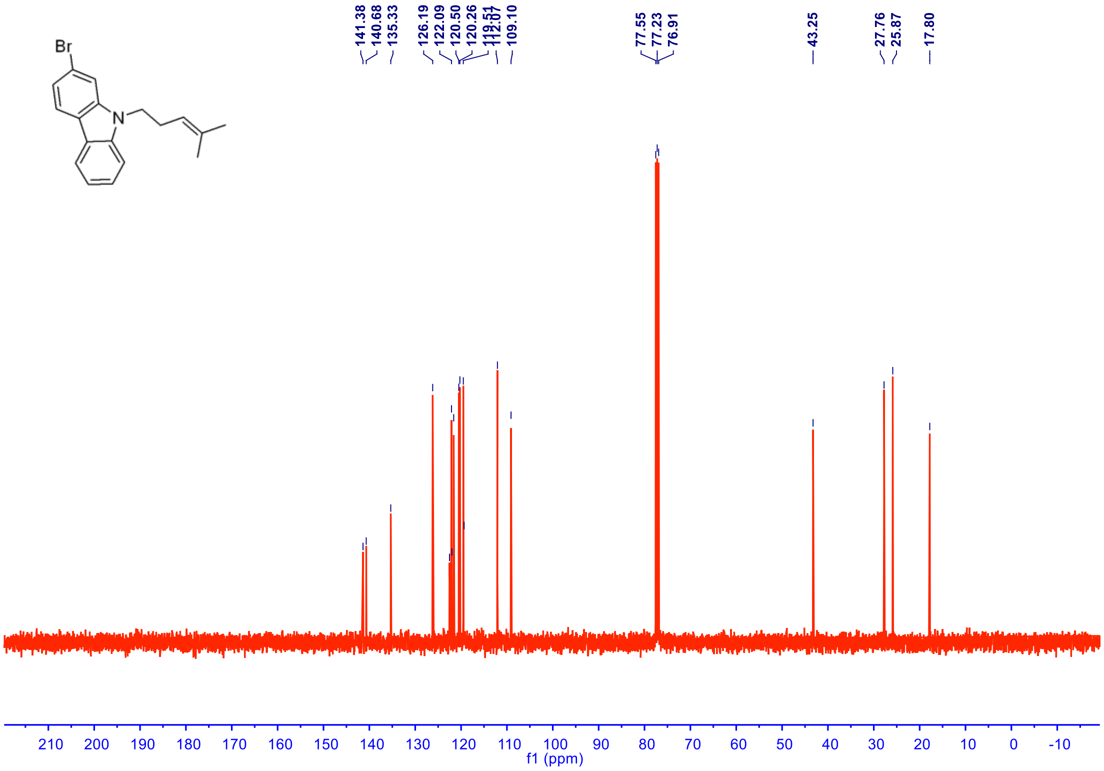

Supplementary Figure 310. ^{13}C NMR spectrum of **2-bromo-9-(4-methylpent-3-en-1-yl)-9*H*-carbazole 32b** (101 MHz, $CDCl_3$)

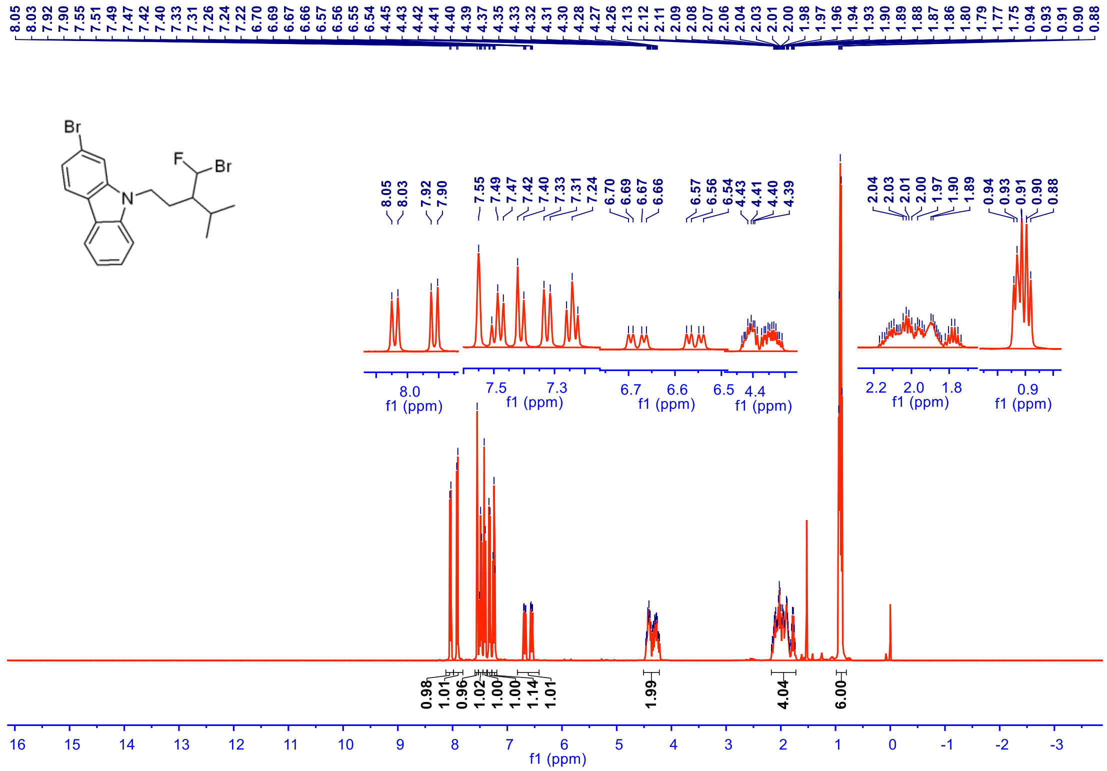

Supplementary Figure 311. ^{1}H NMR spectrum of **2-bromo-9-(3-(bromofluoromethyl)-4-methylpentyl)-9*H*-carbazole 32a** (400 MHz, $CDCl_3$)

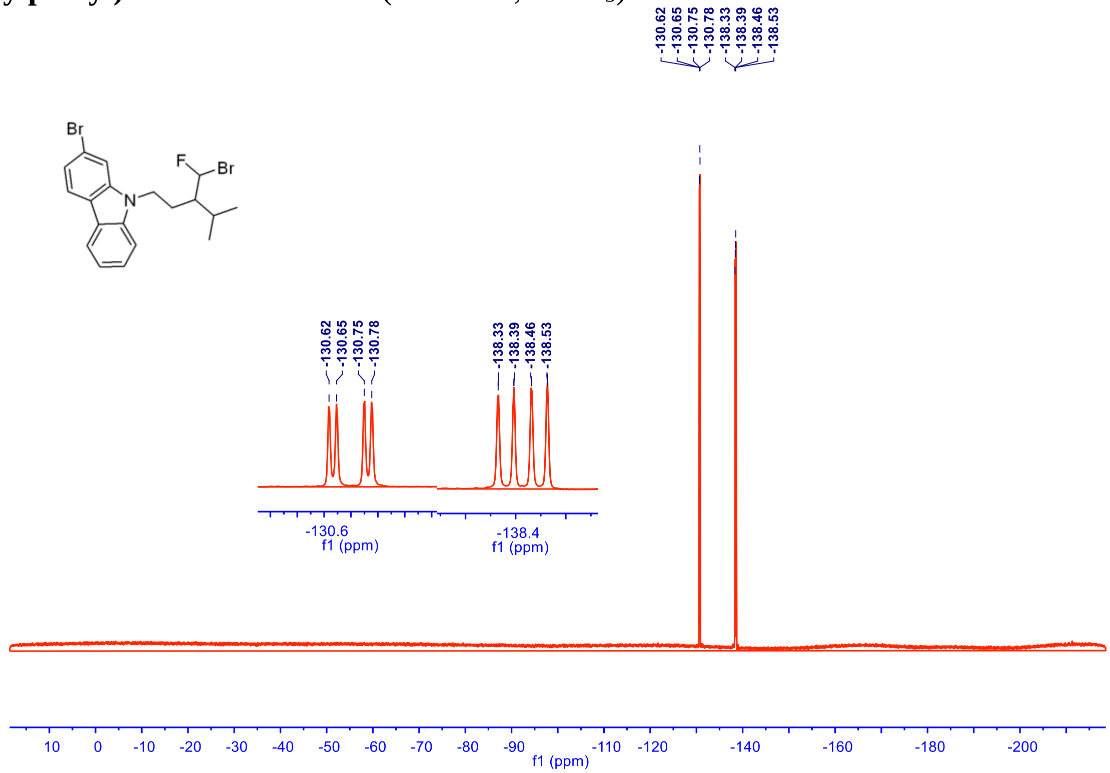

Supplementary Figure 312. ^{19}F NMR spectrum of **2-bromo-9-(3-(bromofluoromethyl)-4-methylpentyl)-9*H*-carbazole 32a** (376 MHz, $CDCl_3$)

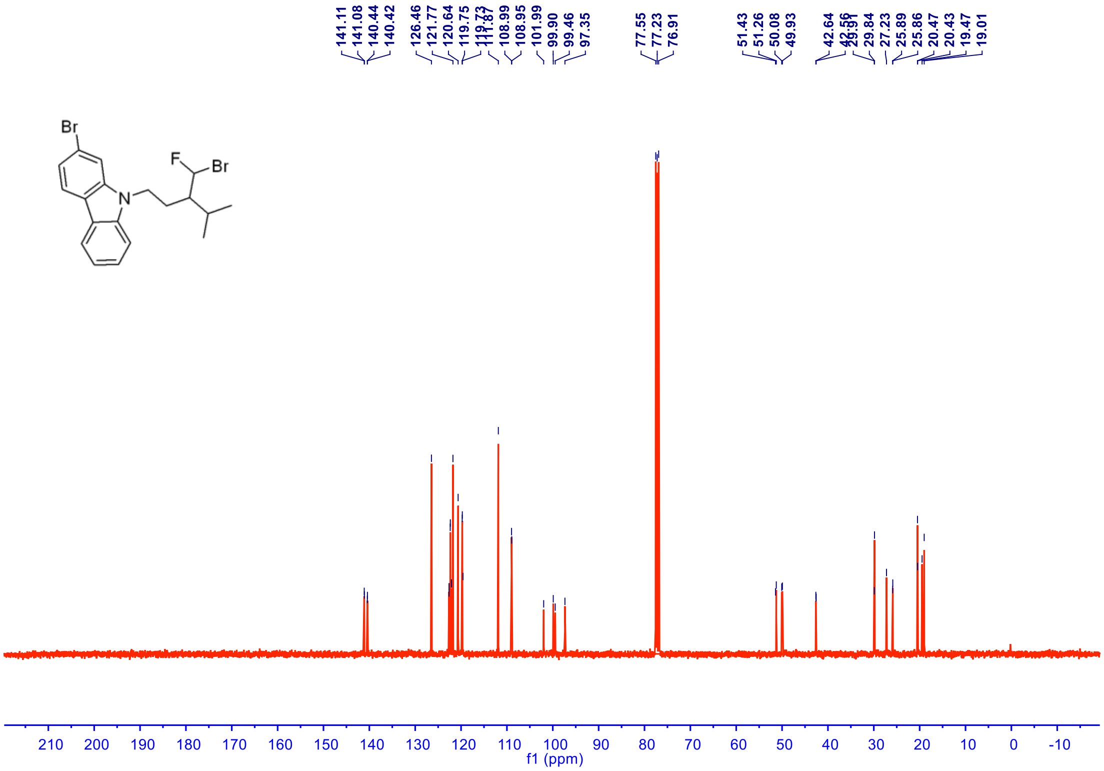

Supplementary Figure 313. ^{13}C NMR spectrum of **2-bromo-9-(3-(bromofluoromethyl)-4-methylpentyl)-9*H*-carbazole 32a** (101 MHz, $CDCl_3$)

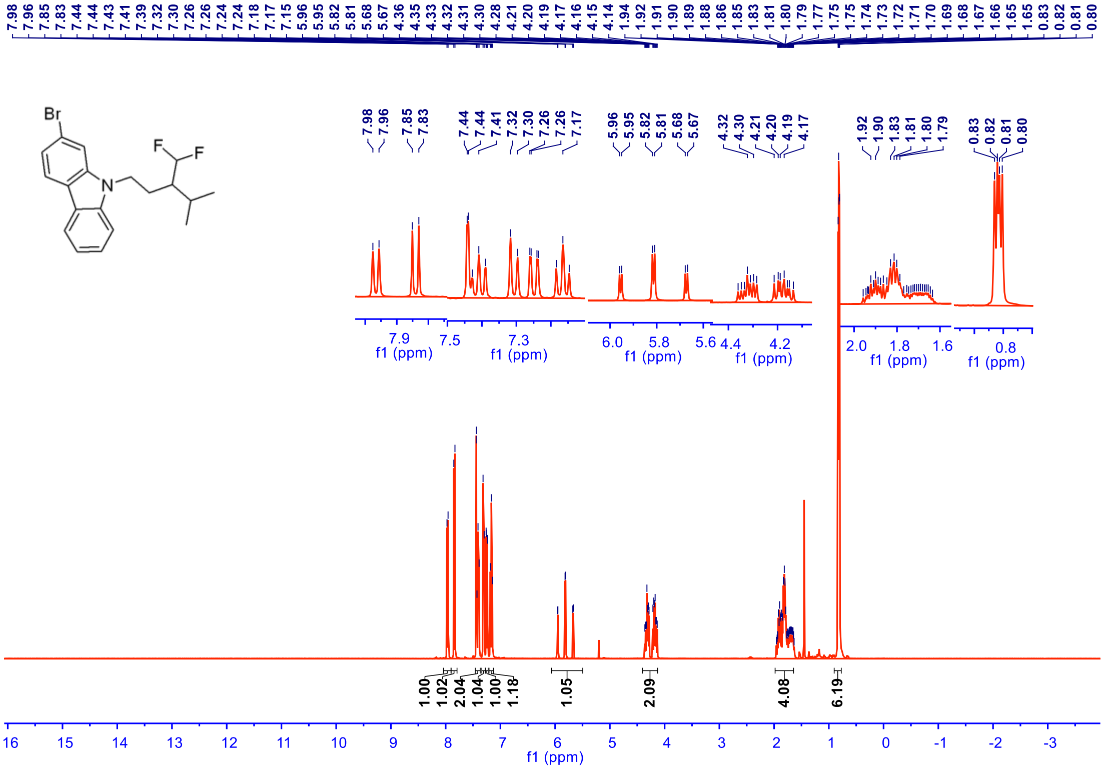

Supplementary Figure 314. ^{1}H NMR spectrum of compound **32** (400 MHz, $CDCl_3$)

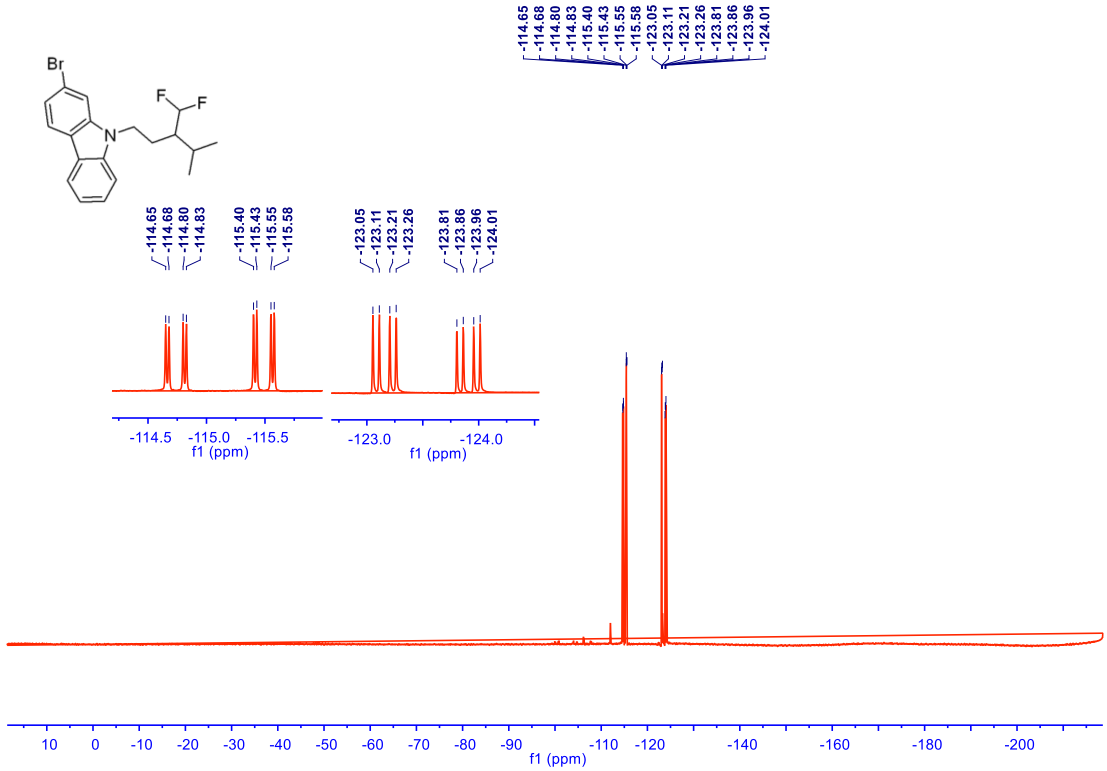

Supplementary Figure 315. ^{19}F NMR spectrum of compound **32** (376 MHz, $CDCl_3$)

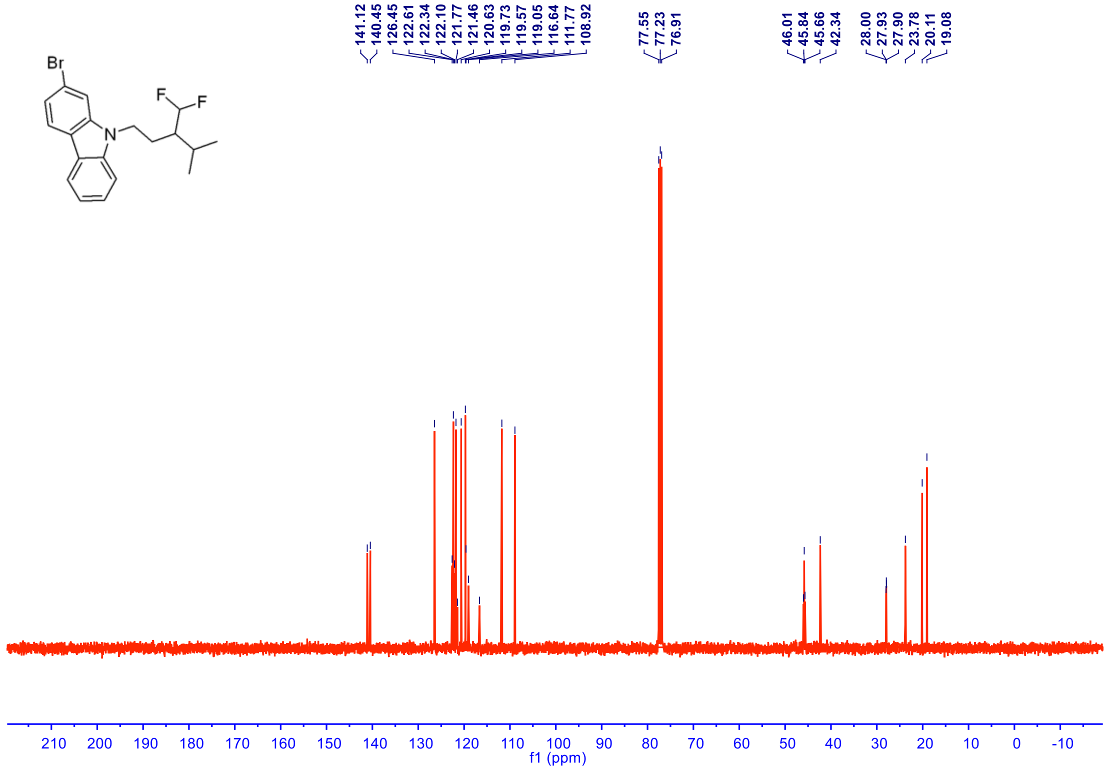

Supplementary Figure 316. ^{13}C NMR spectrum of compound **32** (101 MHz, $CDCl_3$)

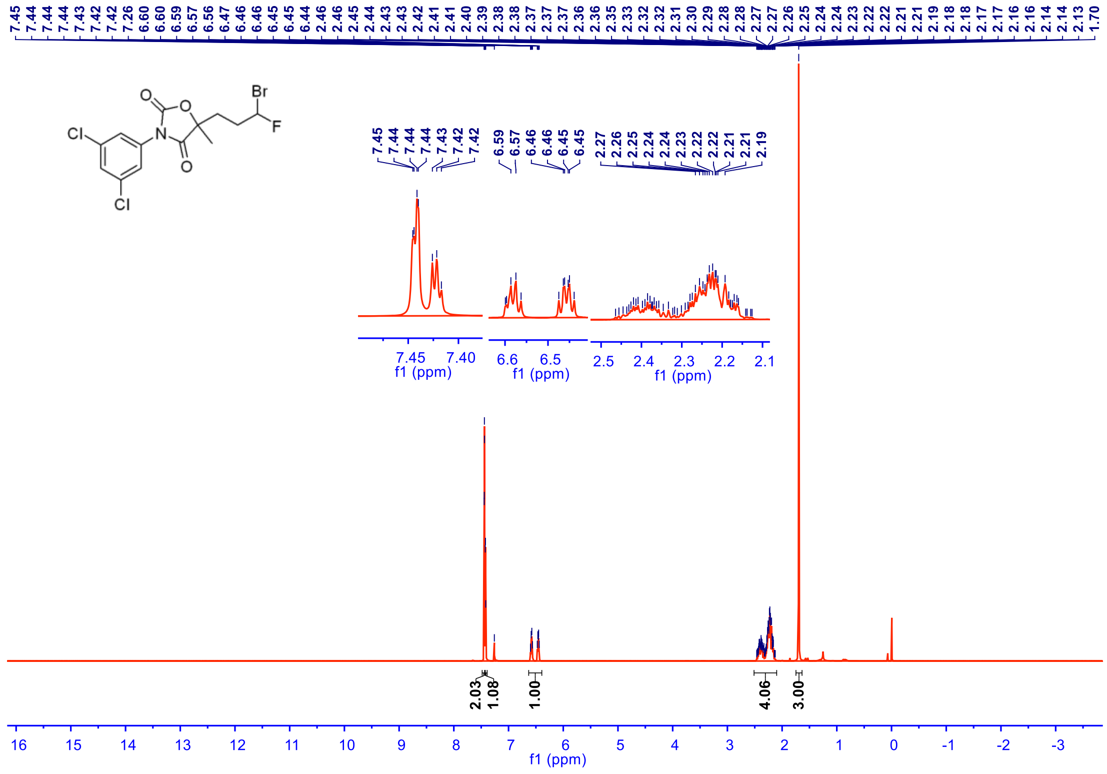

Supplementary Figure 317. ^{1}H NMR spectrum of **5-(3-bromo-3-fluoropropyl)-3-(3,5-dichlorophenyl)-5-methyloxazolidine-2,4-dione 33a** (400 MHz, $CDCl_3$)

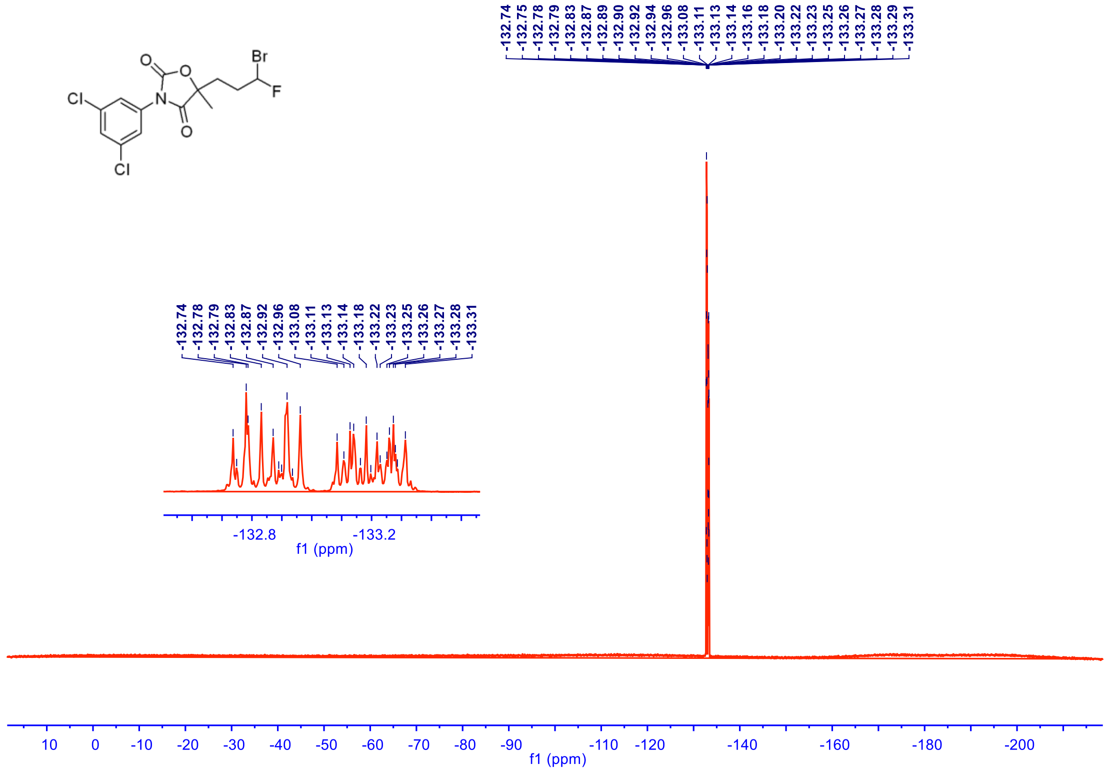

Supplementary Figure 318. ^{19}F NMR spectrum of **5-(3-bromo-3-fluoropropyl)-3-(3,5-dichlorophenyl)-5-methyloxazolidine-2,4-dione 33a** (376 MHz, $CDCl_3$)

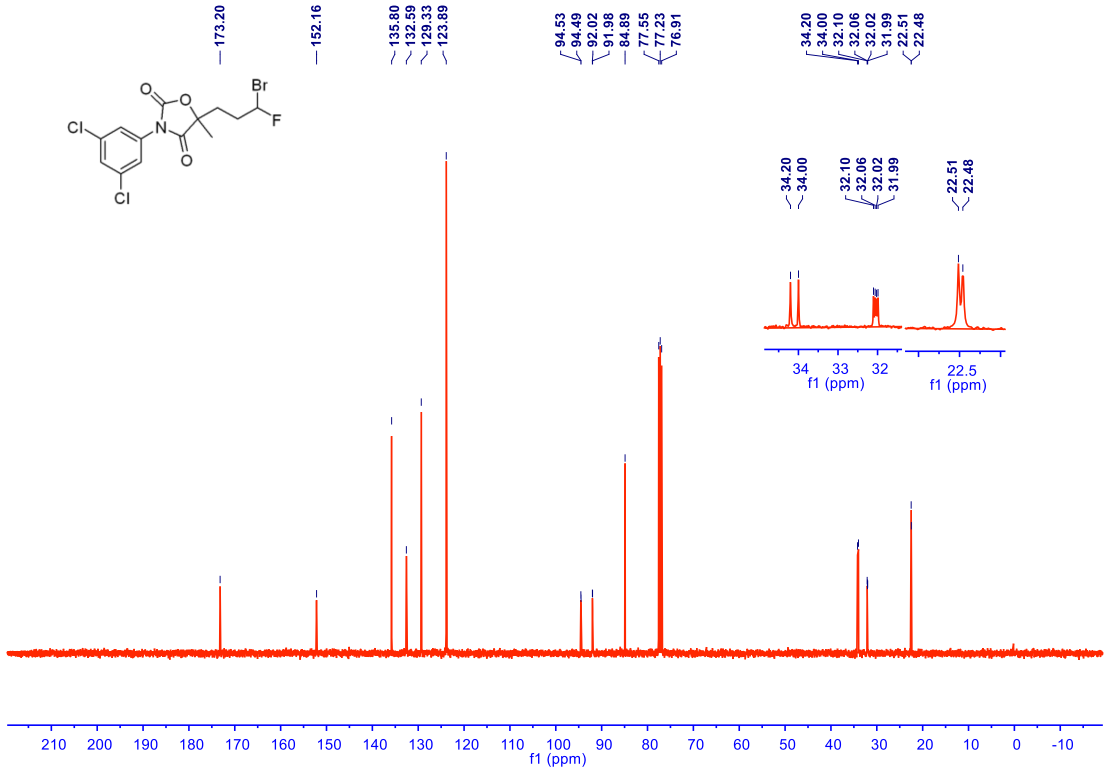

Supplementary Figure 319. ^{13}C NMR spectrum of **5-(3-bromo-3-fluoropropyl)-3-(3,5-dichlorophenyl)-5-methyloxazolidine-2,4-dione 33a** (101 MHz, $CDCl_3$)

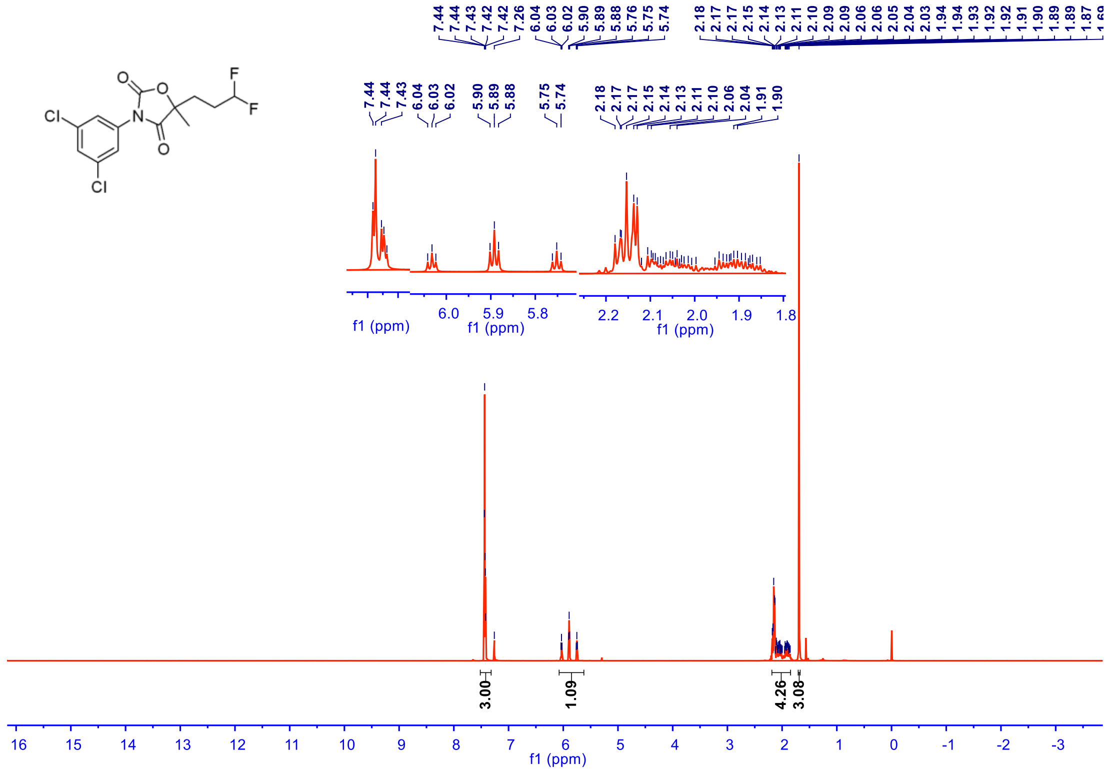

Supplementary Figure 320. ^{1}H NMR spectrum of compound **33** (400 MHz, $CDCl_3$)

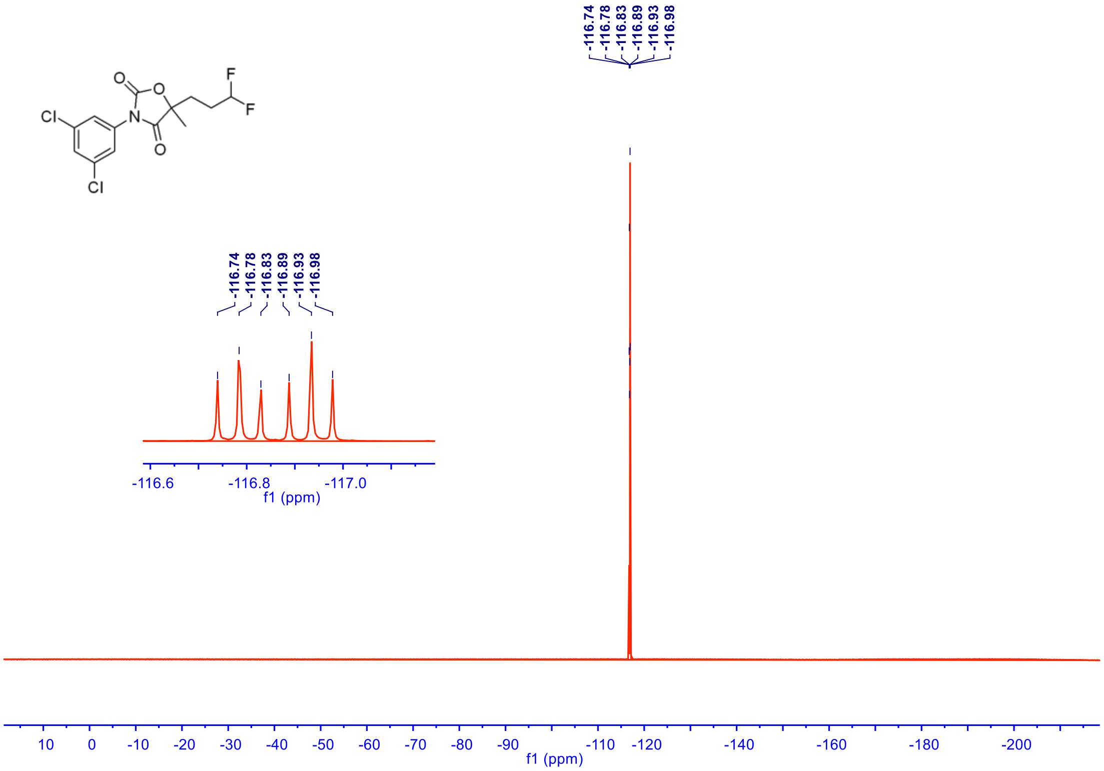

Supplementary Figure 321. ^{19}F NMR spectrum of compound **33** (376 MHz, $CDCl_3$)

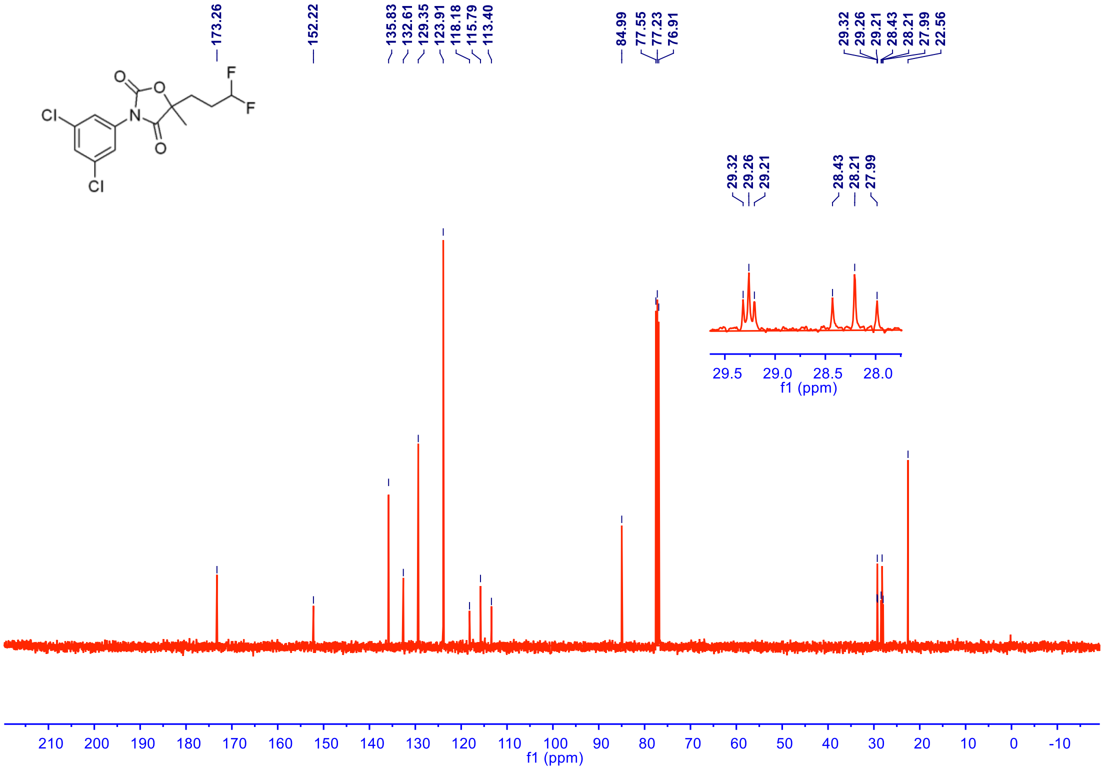

Supplementary Figure 322. ^{13}C NMR spectrum of compound **33** (101 MHz, $CDCl_3$)

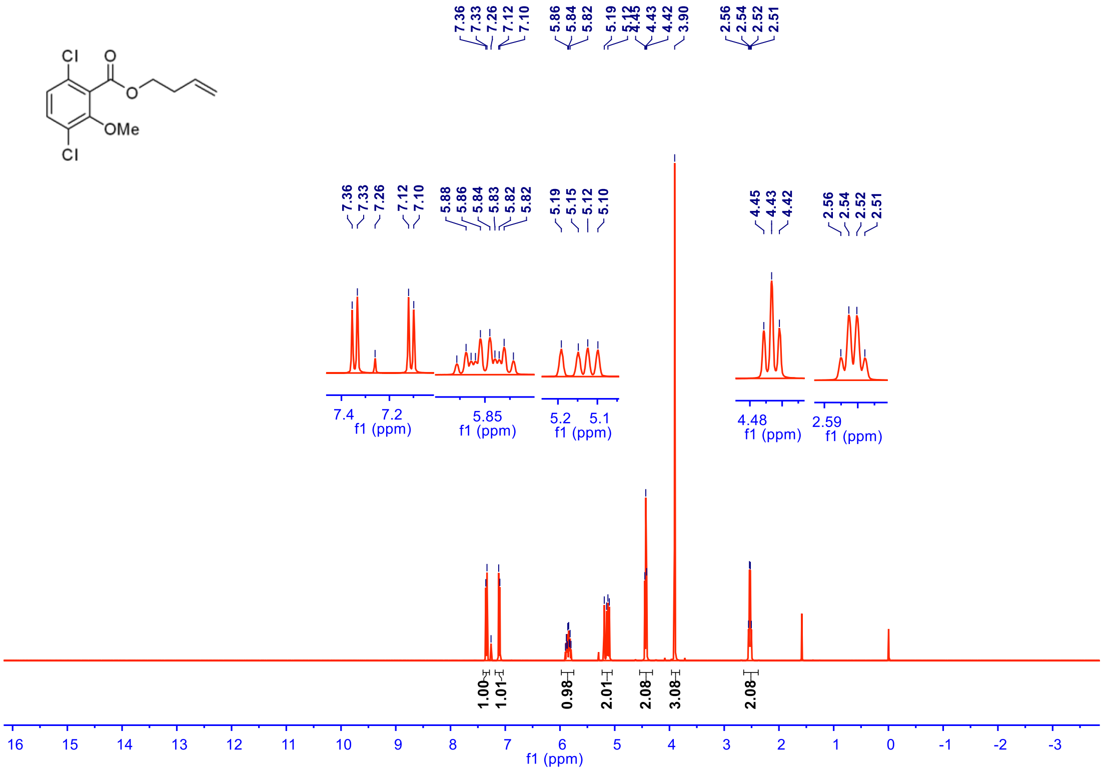

Supplementary Figure 323. ^{1}H NMR spectrum of **but-3-en-1-yl 3,6-dichloro-2-methoxybenzoate 34b** (400 MHz, $CDCl_3$)

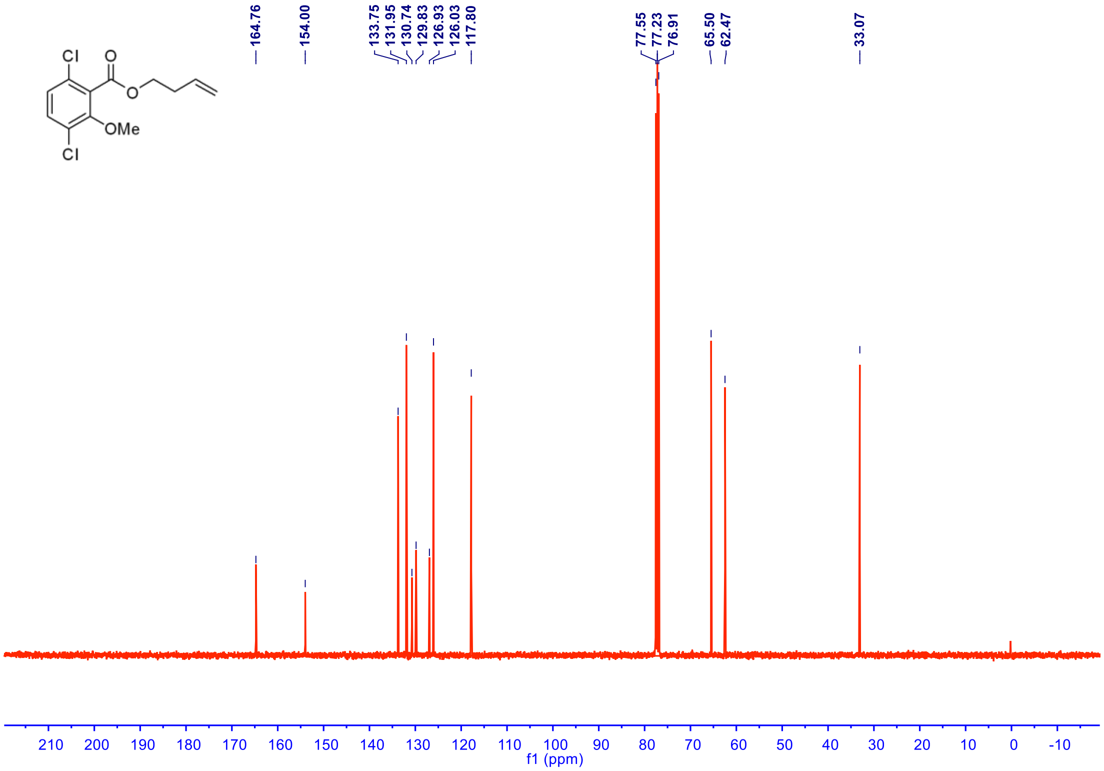

Supplementary Figure 324. ^{13}C NMR spectrum of **but-3-en-1-yl 3,6-dichloro-2-methoxybenzoate 34b** (101 MHz, $CDCl_3$)

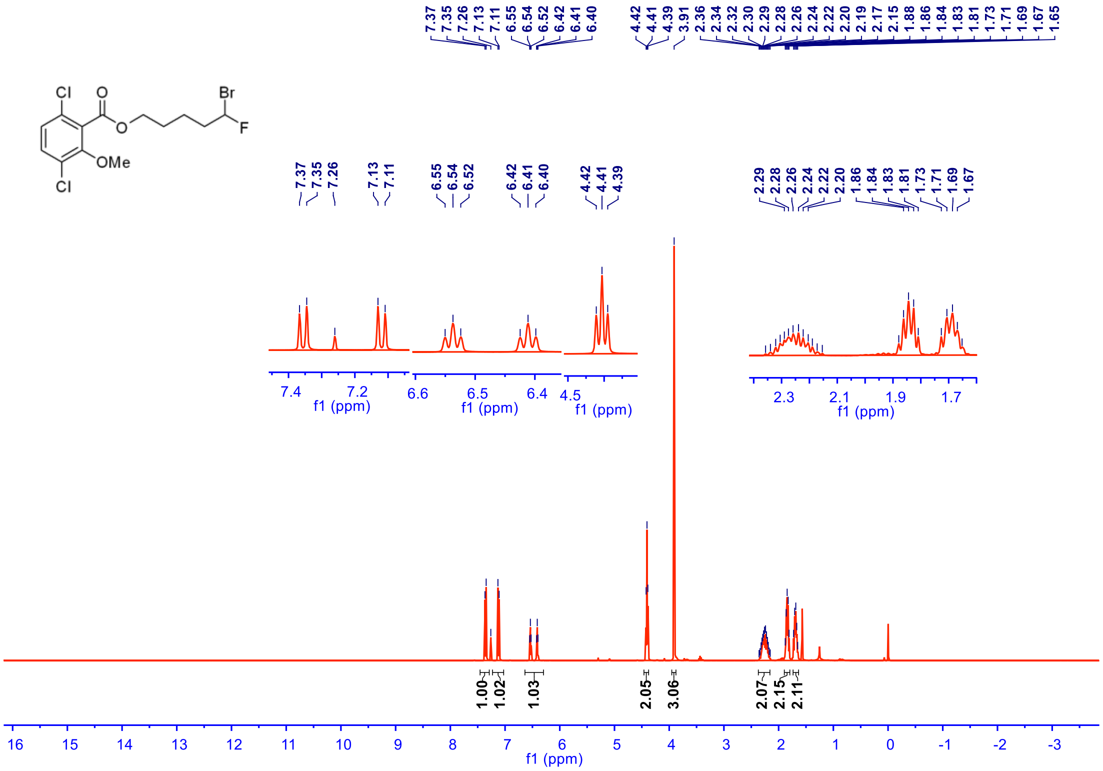

Supplementary Figure 325. ^{1}H NMR spectrum of **5-bromo-5-fluoropentyl 3,6-dichloro-2-methoxybenzoate 34a** (400 MHz, $CDCl_3$)

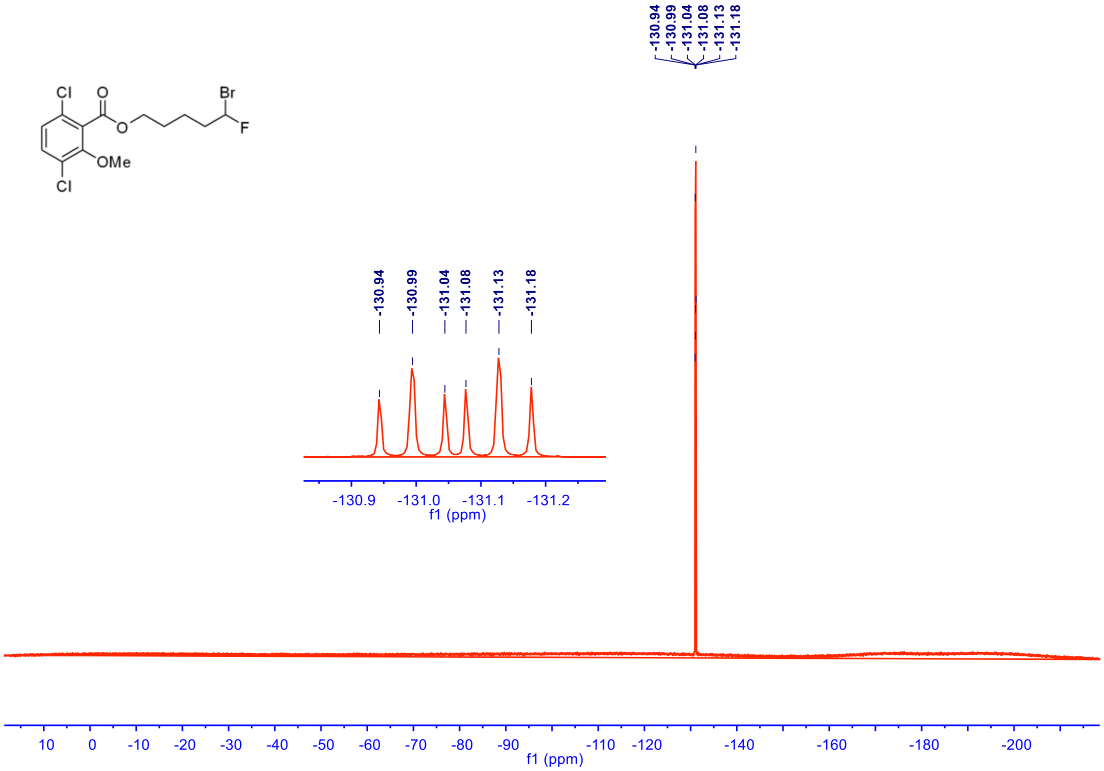

Supplementary Figure 326. ^{19}F NMR spectrum of **5-bromo-5-fluoropentyl 3,6-dichloro-2-methoxybenzoate 34a** (376 MHz, $CDCl_3$)

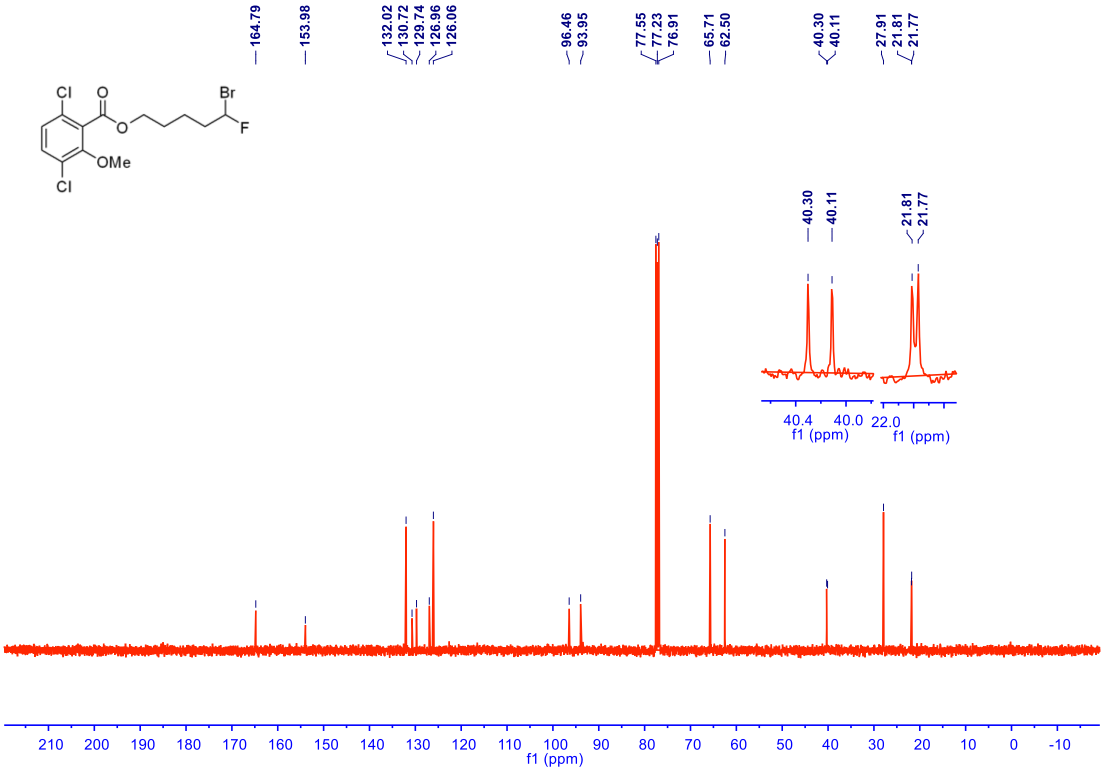

Supplementary Figure 327. ^{13}C NMR spectrum of **5-bromo-5-fluoropentyl 3,6-dichloro-2-methoxybenzoate 34a** (101 MHz, $CDCl_3$)

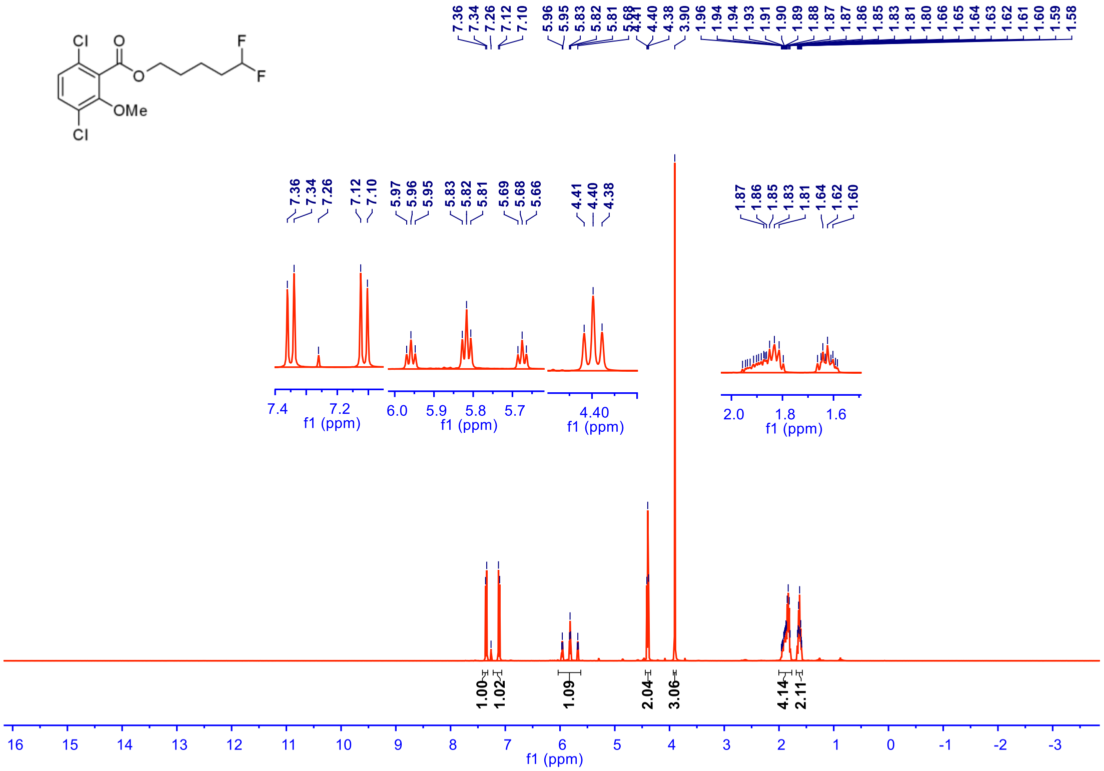

Supplementary Figure 328. ^{1}H NMR spectrum of compound **34** (400 MHz, $CDCl_3$)

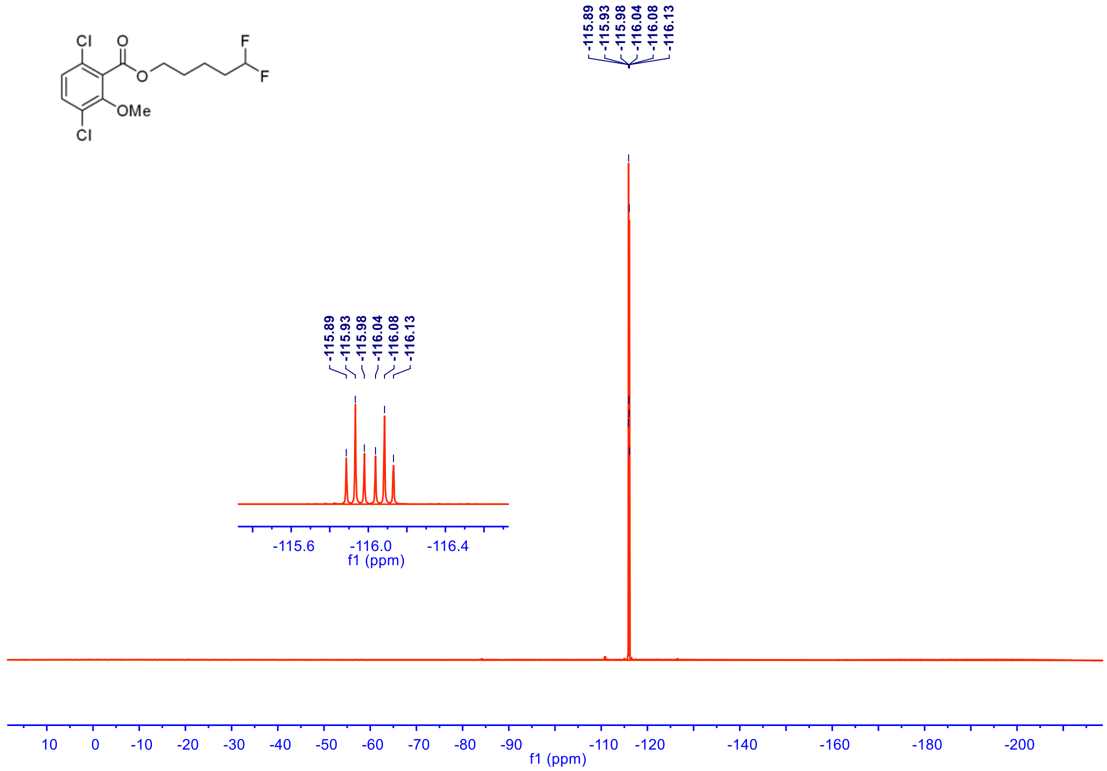

Supplementary Figure 329. ^{19}F NMR spectrum of compound **34** (376 MHz, $CDCl_3$)

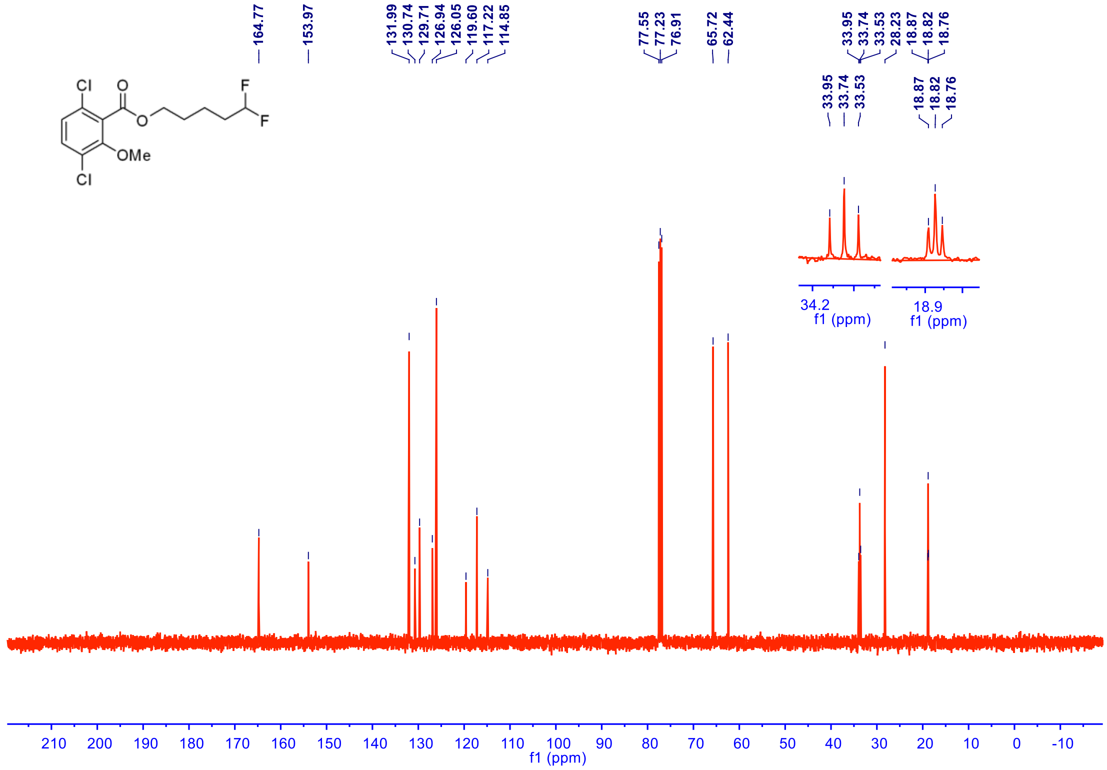

Supplementary Figure 330. ^{13}C NMR spectrum of compound **34** (101 MHz, $CDCl_3$)

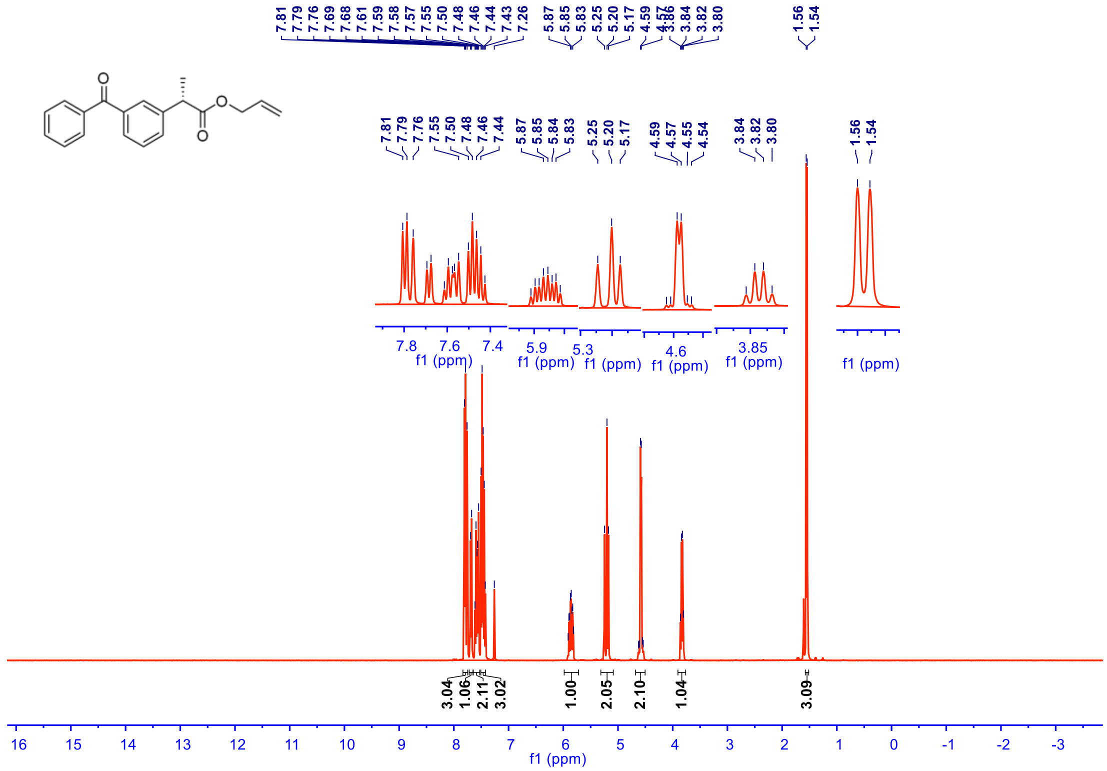

Supplementary Figure 331. ^{1}H NMR spectrum of **allyl (*S*)-2-(3-benzoylphenyl)propanoate 35b** (400 MHz, $CDCl_3$)

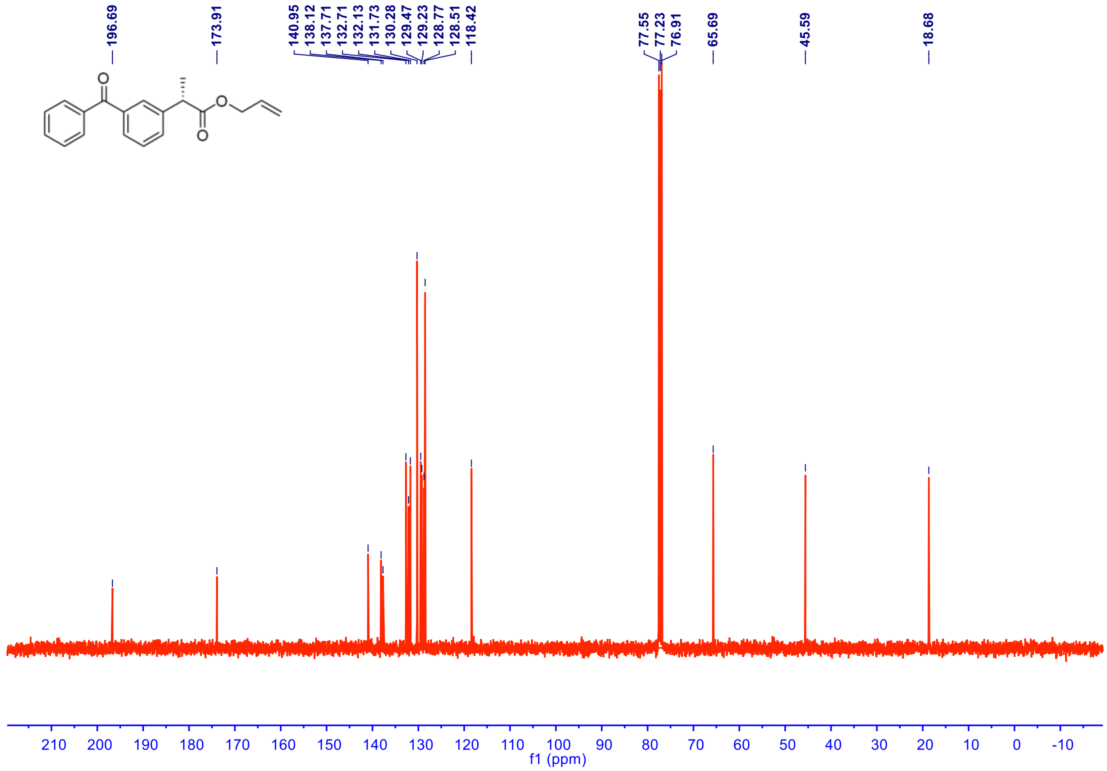

Supplementary Figure 332. ^{13}C NMR spectrum of **allyl (*S*)-2-(3-benzoylphenyl)propanoate 35b** (101 MHz, $CDCl_3$)

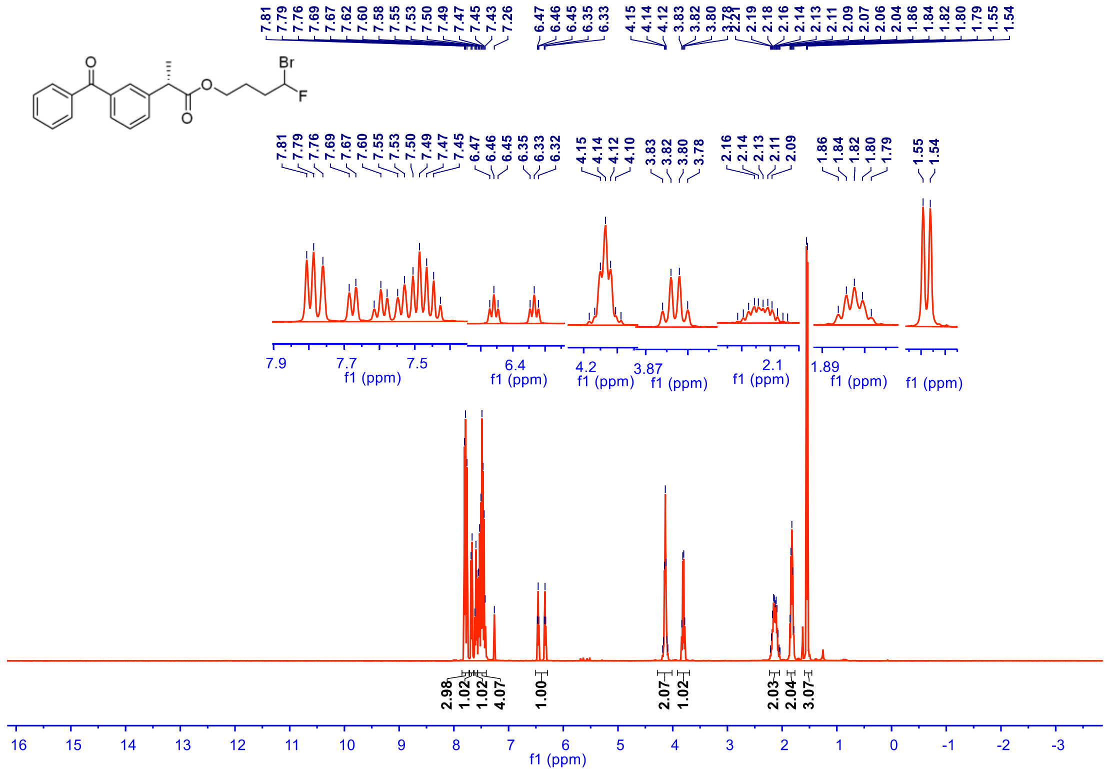

Supplementary Figure 333. ^{1}H NMR spectrum of **4-bromo-4-fluorobutyl (2*S*)-2-(3-benzoylphenyl)propanoate 35a** (400 MHz, $CDCl_3$)

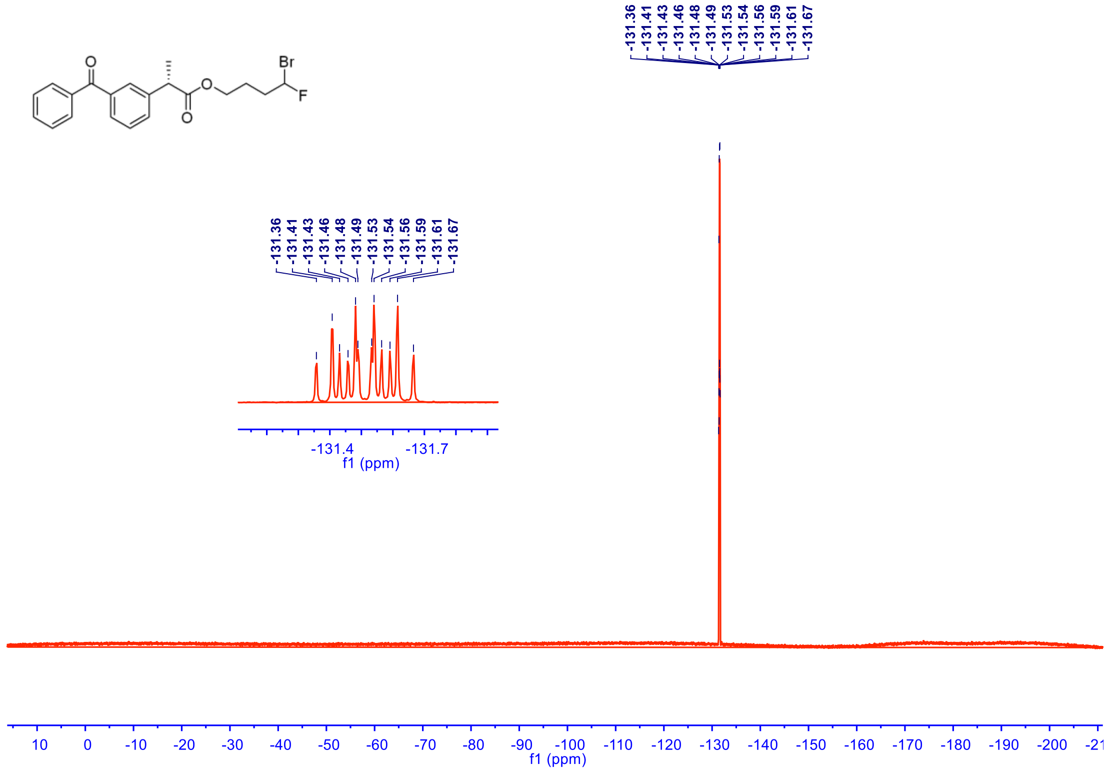

Supplementary Figure 334. ^{19}F NMR spectrum of **4-bromo-4-fluorobutyl (2*S*)-2-(3-benzoylphenyl)propanoate 35a** (376 MHz, $CDCl_3$)

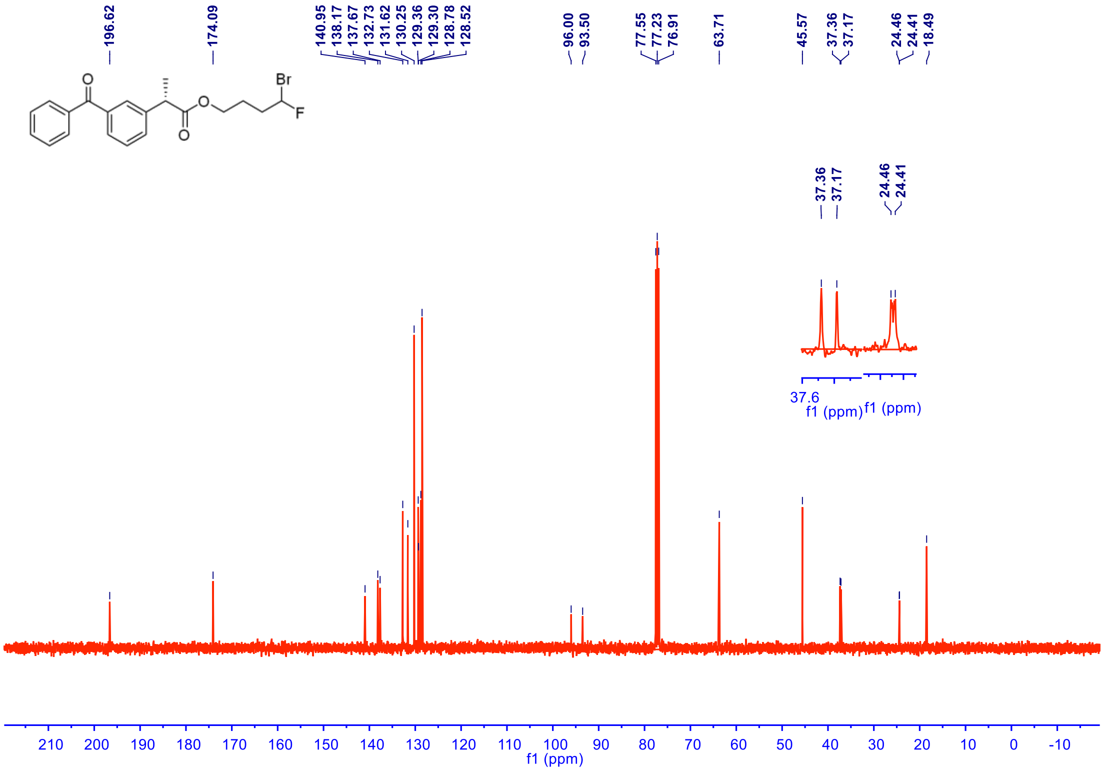

Supplementary Figure 335. ^{13}C NMR spectrum of **4-bromo-4-fluorobutyl (2*S*)-2-(3-benzoylphenyl)propanoate 35a** (101 MHz, $CDCl_3$)

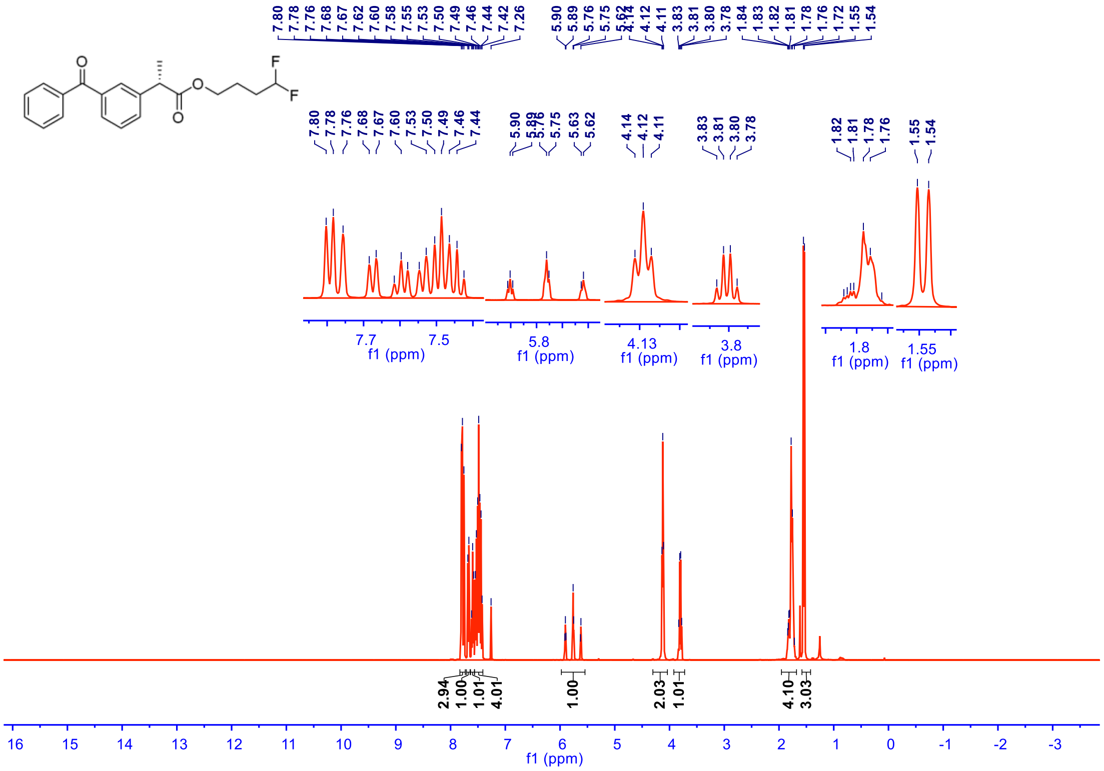

Supplementary Figure 336. ^{1}H NMR spectrum of compound **35** (400 MHz, $CDCl_3$)

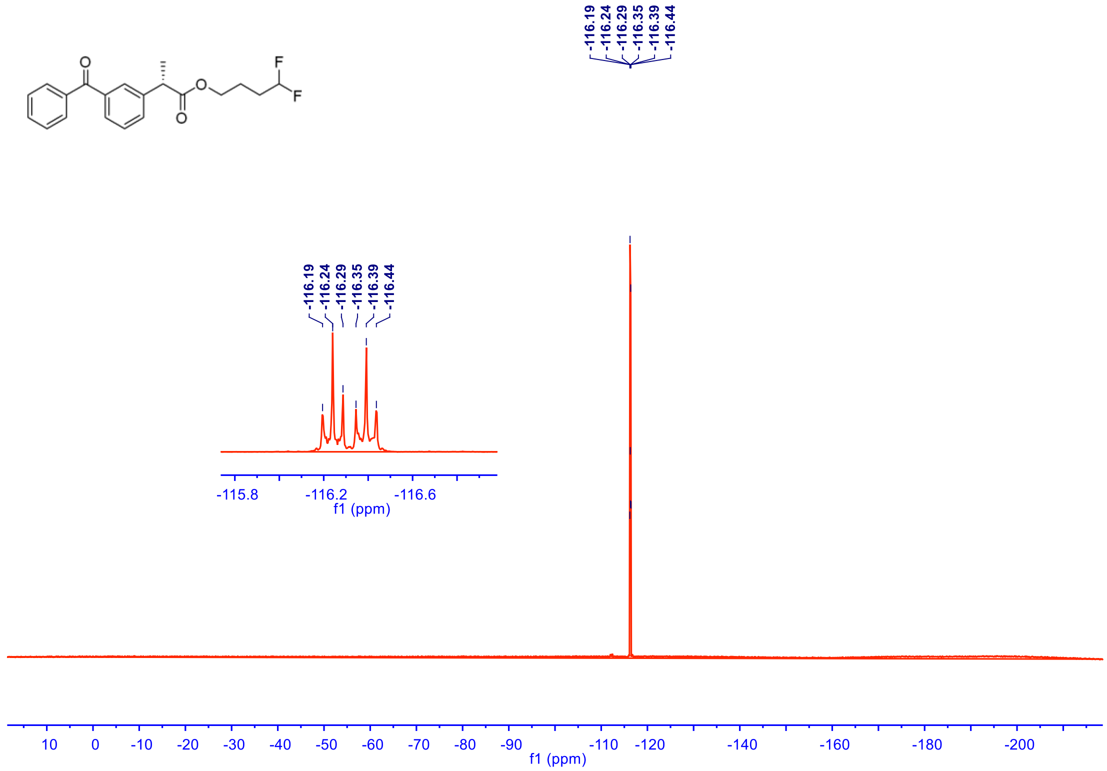

Supplementary Figure 337. ^{19}F NMR spectrum of compound **35** (376 MHz, $CDCl_3$)

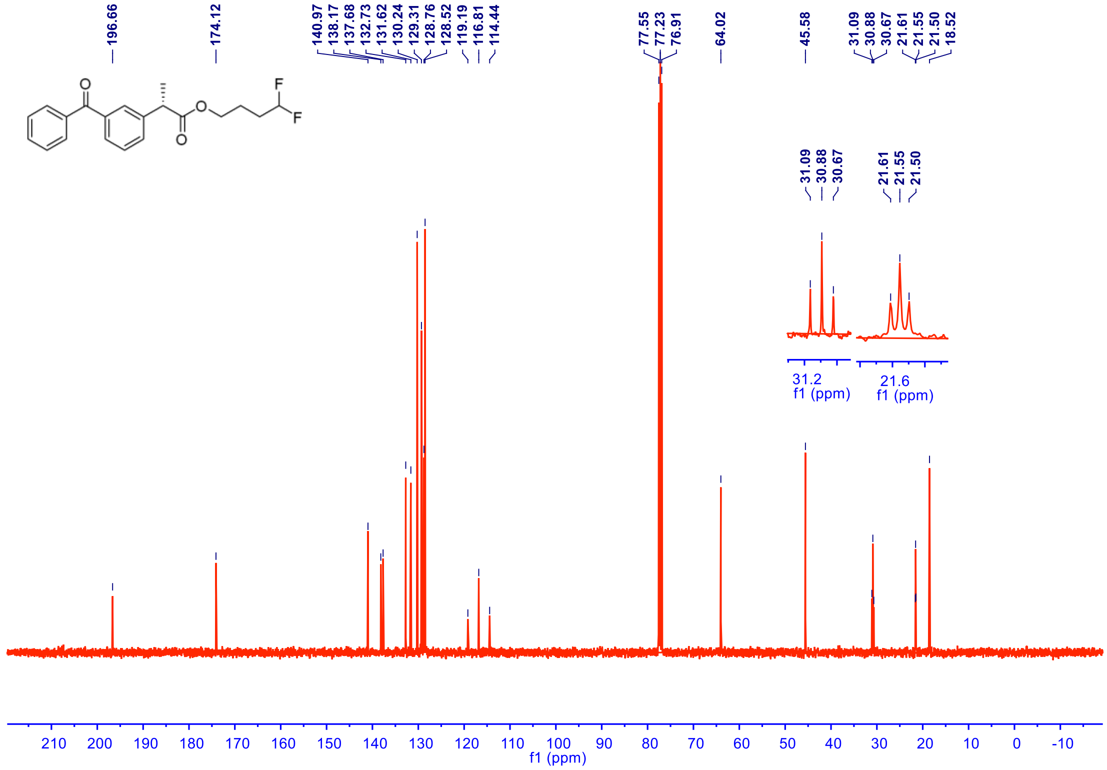

Supplementary Figure 338. ^{13}C NMR spectrum of compound **35** (101 MHz, $CDCl_3$)

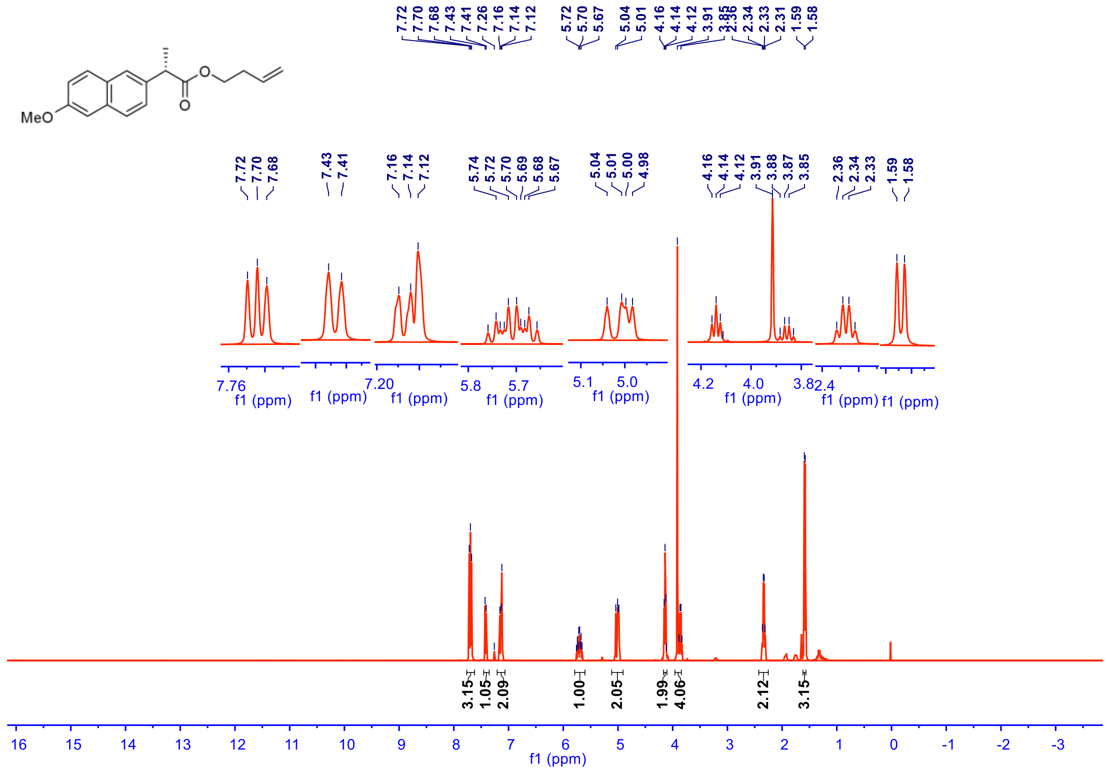

Supplementary Figure 339. ^{1}H NMR spectrum of **5-bromo-5-fluoropentyl (2*S*)-2-(6-methoxynaphthalen-2-yl)propanoate 36b** (400 MHz, $CDCl_3$)

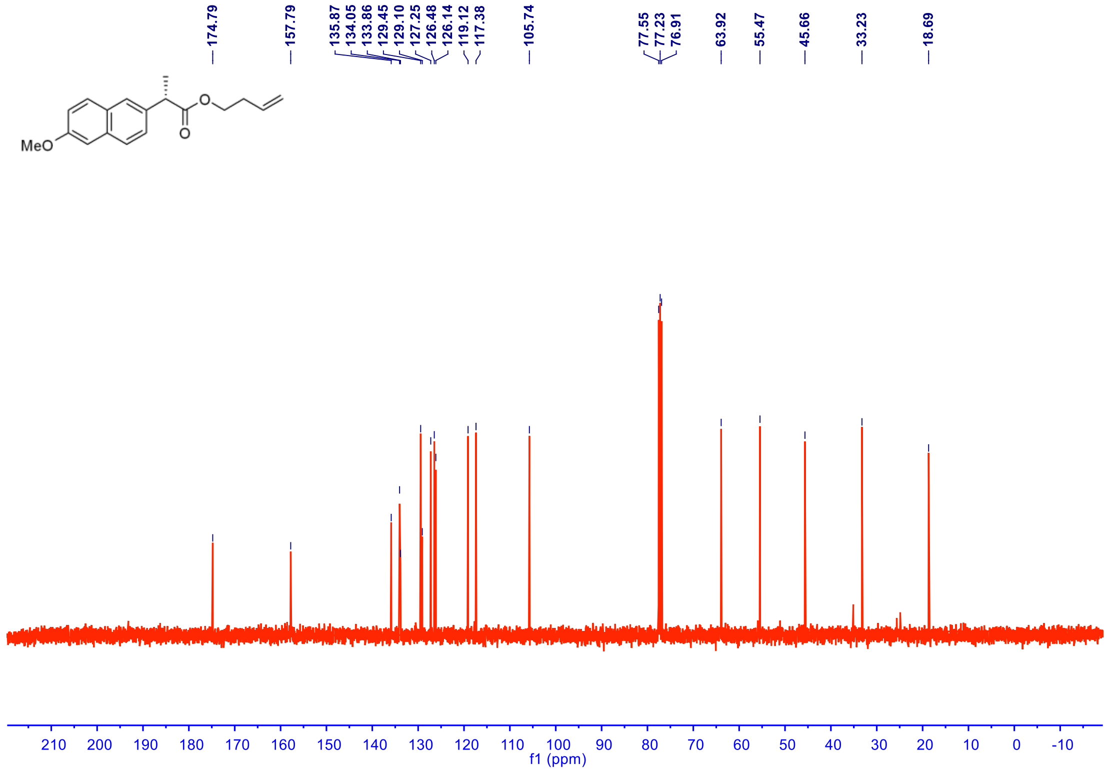

Supplementary Figure 340. ^{13}C NMR spectrum of **but-3-en-1-yl (*S*)-2-(6-methoxynaphthalen-2-yl)propanoate 36b** (101 MHz, $CDCl_3$)

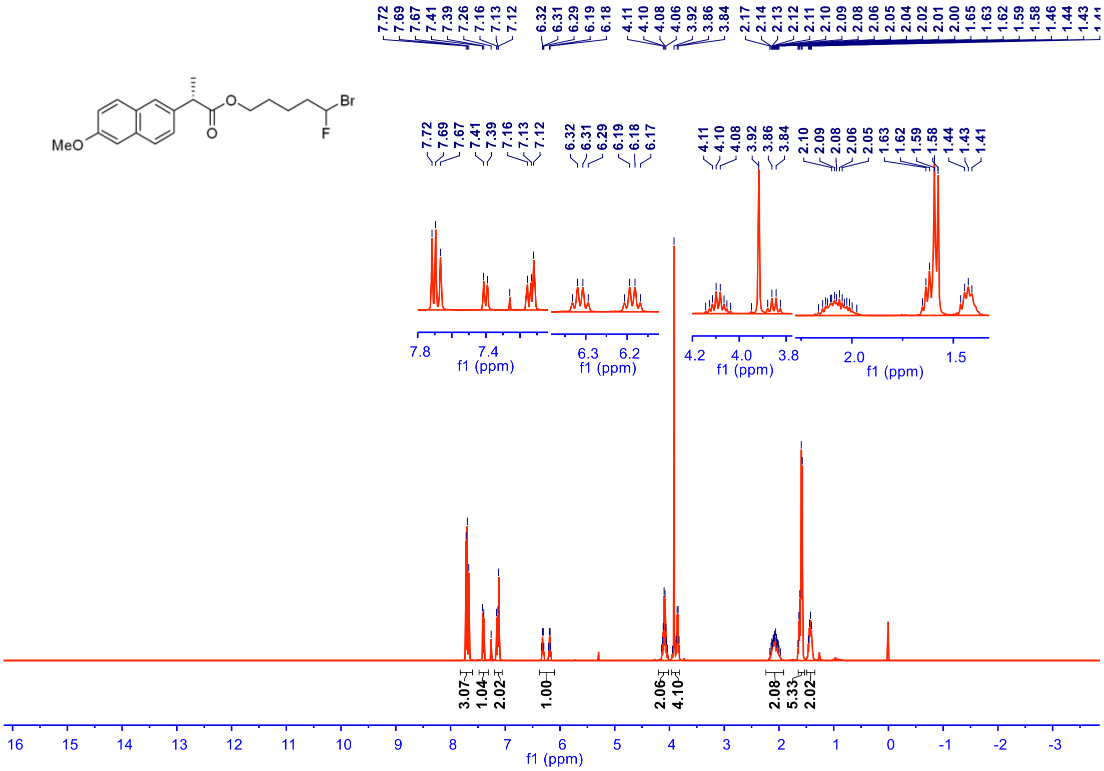

Supplementary Figure 341. ^{1}H NMR spectrum of **5-bromo-5-fluoropentyl (2*S*)-2-(6-methoxynaphthalen-2-yl)propanoate 36a** (400 MHz, $CDCl_3$)

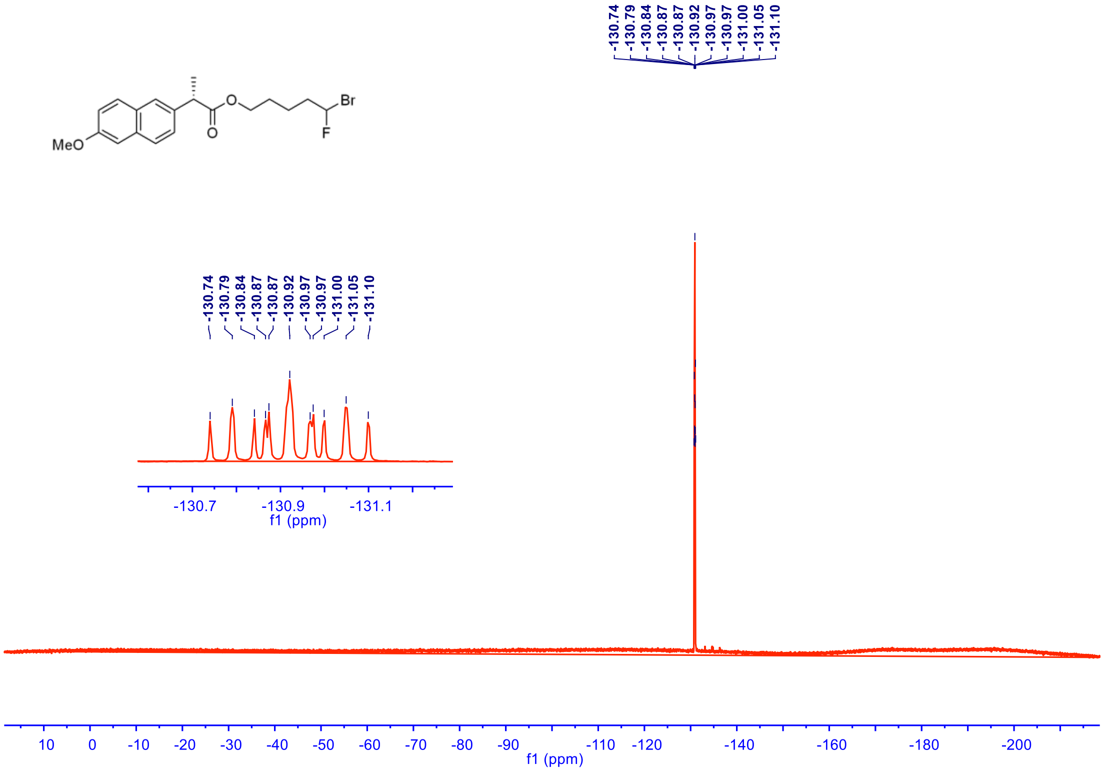

Supplementary Figure 342. ^{19}F NMR spectrum of **5-bromo-5-fluoropentyl (2*S*)-2-(6-methoxynaphthalen-2-yl)propanoate 36a** (376 MHz, $CDCl_3$)

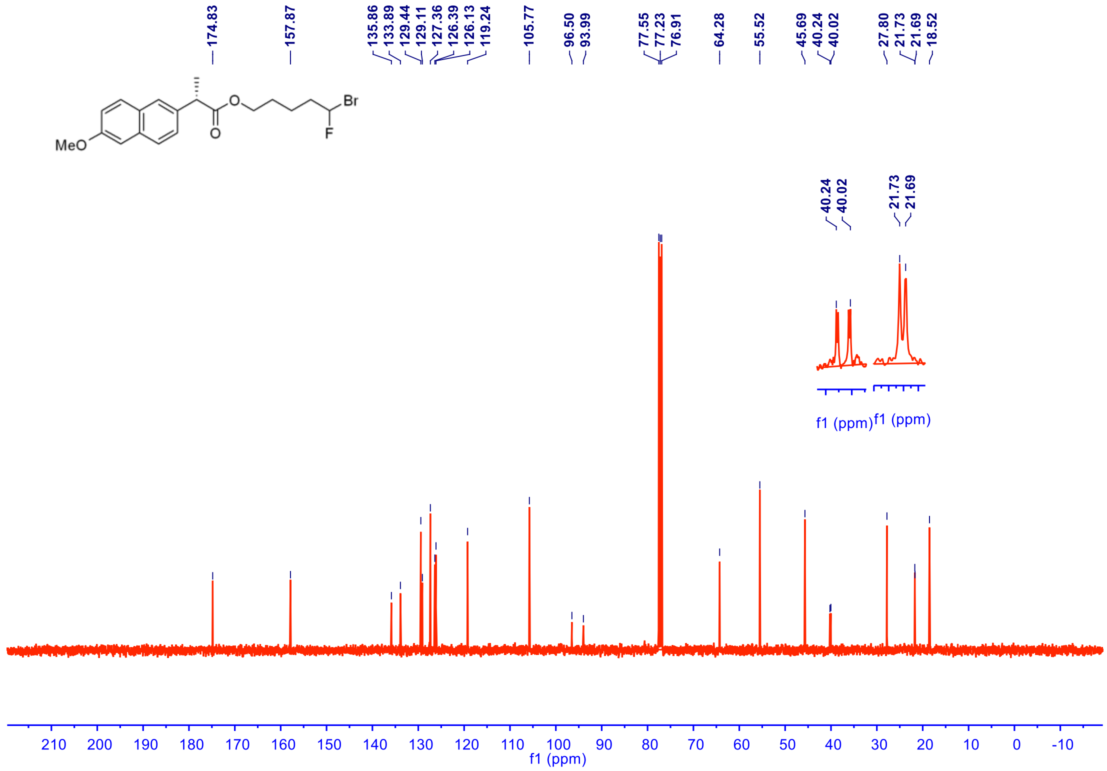

Supplementary Figure 343. ^{13}C NMR spectrum of **5-bromo-5-fluoropentyl (2*S*)-2-(6-methoxynaphthalen-2-yl)propanoate 36a** (101 MHz, $CDCl_3$)

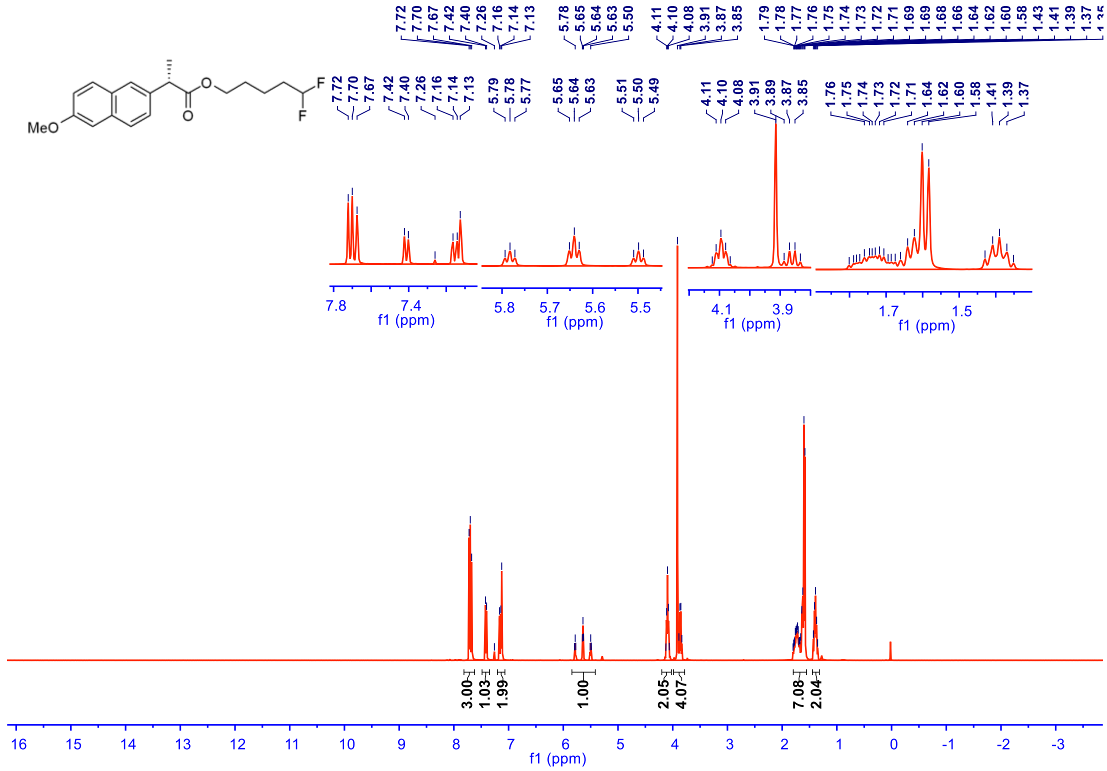

Supplementary Figure 344. ^{1}H NMR spectrum of compound **36** (400 MHz, $CDCl_3$)

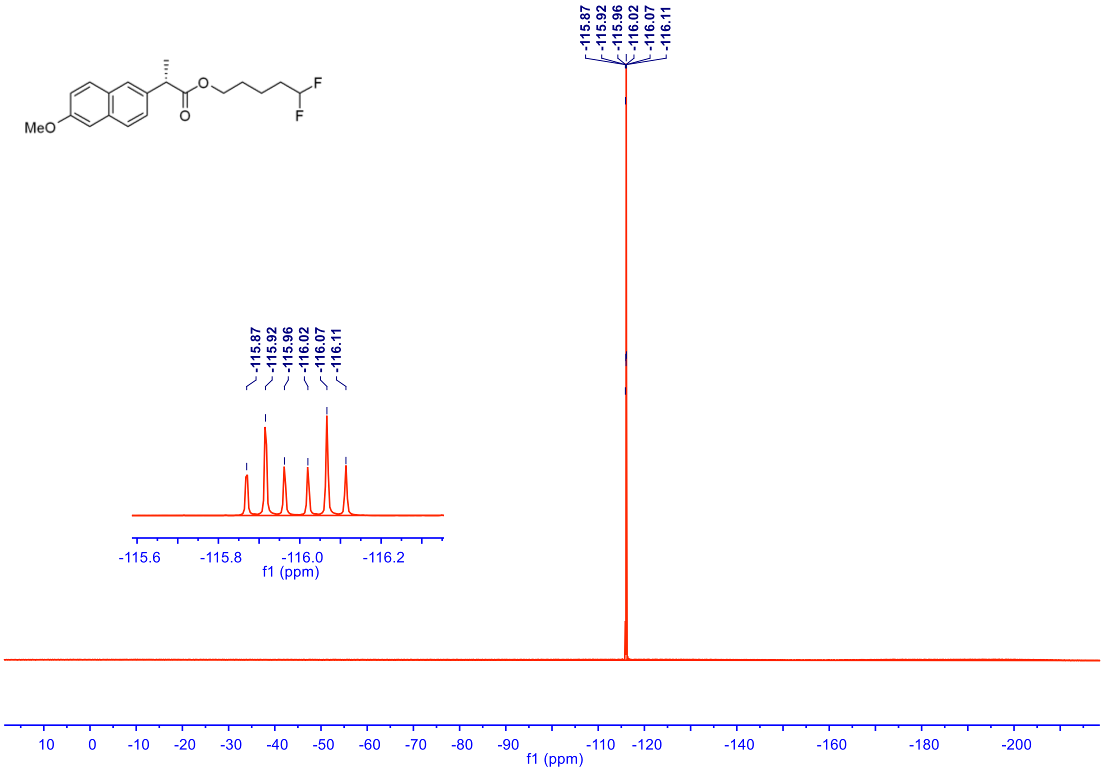

Supplementary Figure 345. ^{19}F NMR spectrum of compound **36** (376 MHz, $CDCl_3$)

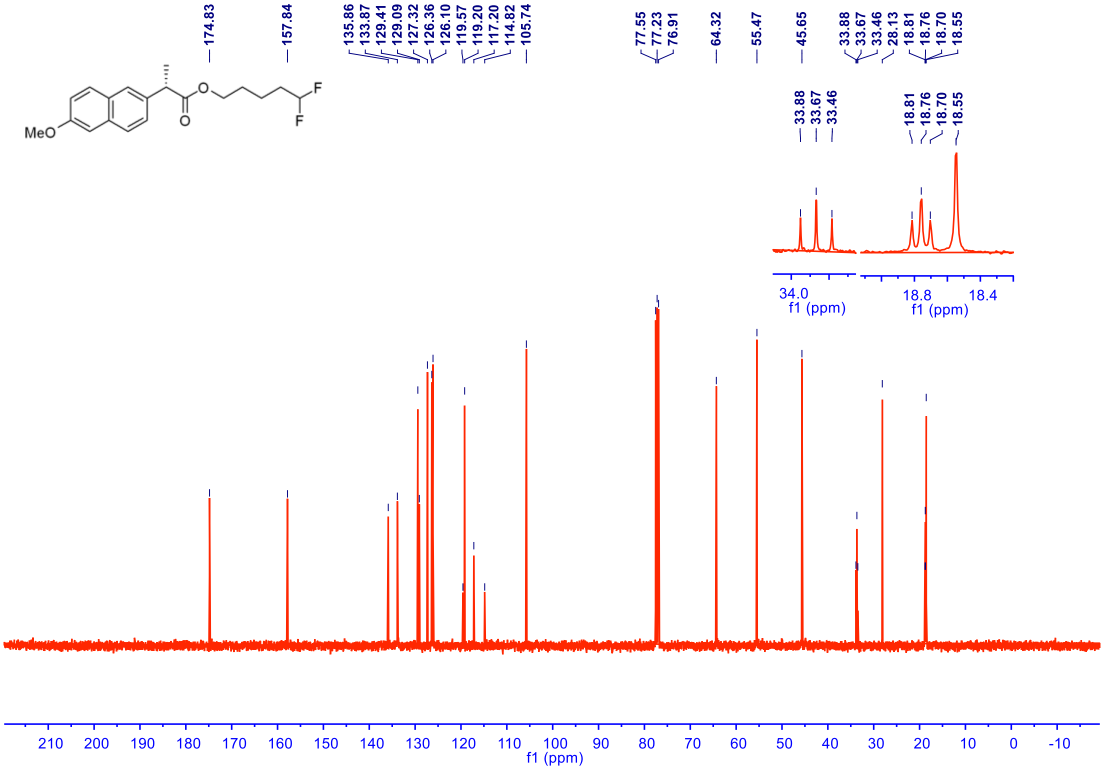

Supplementary Figure 346. ^{13}C NMR spectrum of compound **36** (101 MHz, $CDCl_3$)

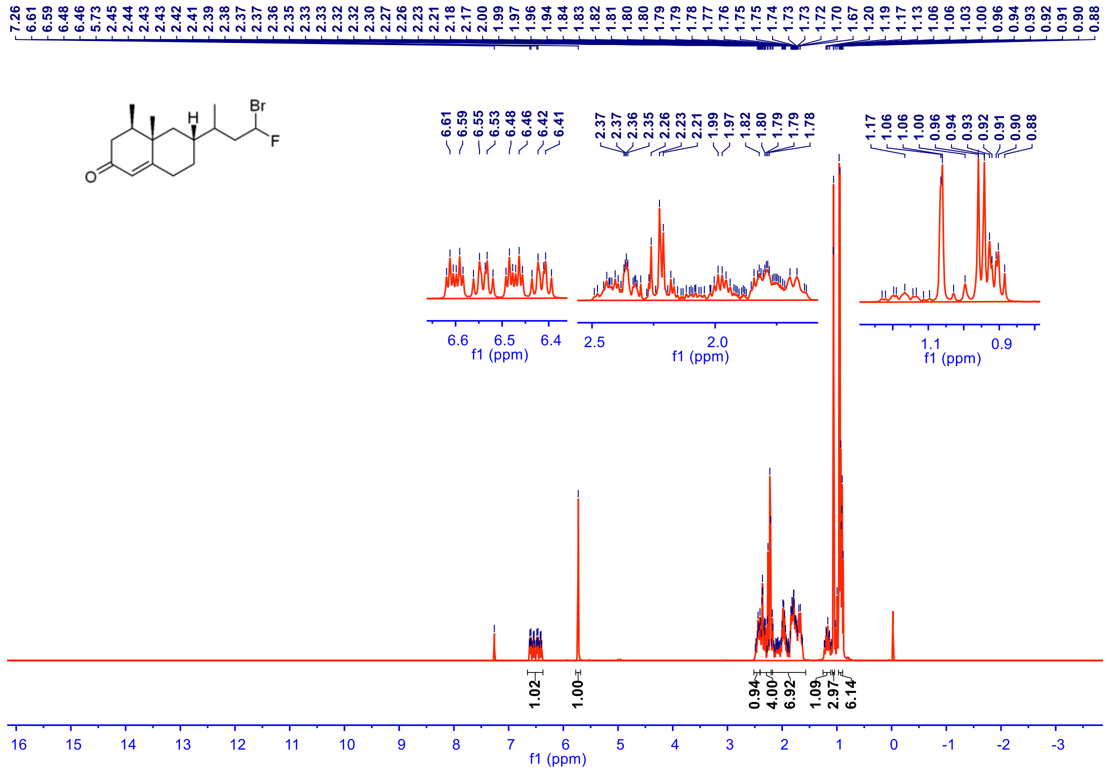

Supplementary Figure 347. ^{1}H NMR spectrum of **(4*R*,4a*S*,6*R*)-6-(4-bromo-4-fluorobutan-2-yl)-4,4a-dimethyl-4,4a,5,6,7,8 hexahydronaphthalen-2(3*H*)-one 37a** (400 MHz, $CDCl_3$)

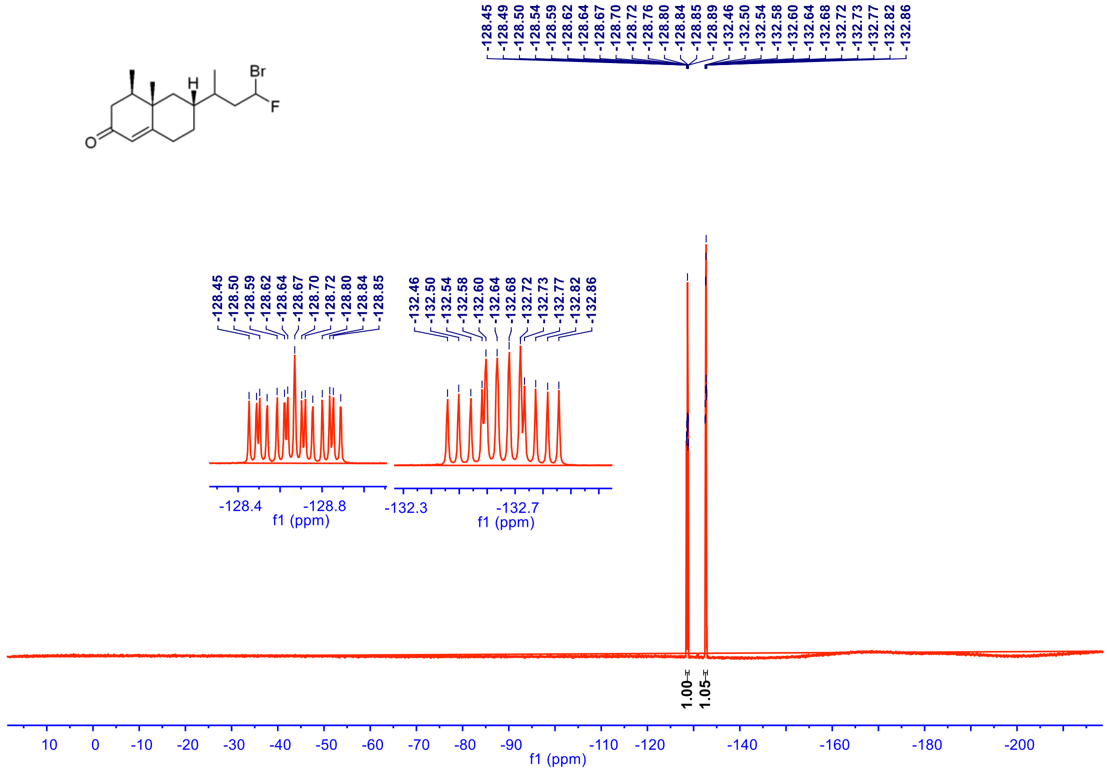

Supplementary Figure 348. ^{19}F NMR spectrum of **(4*R*,4a*S*,6*R*)-6-(4-bromo-4-fluorobutan-2-yl)-4,4a-dimethyl-4,4a,5,6,7,8 hexahydronaphthalen-2(3*H*)-one 37a** (376 MHz, $CDCl_3$)

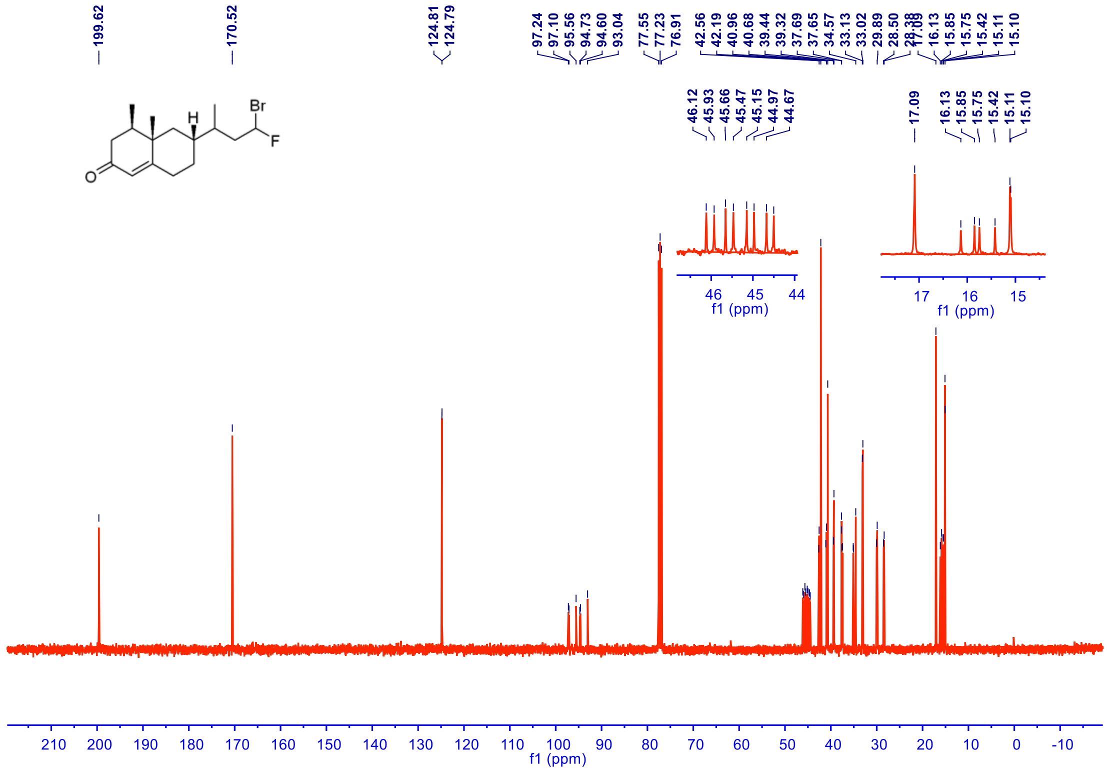

Supplementary Figure 349. ^{13}C NMR spectrum of **(4*R*,4a*S*,6*R*)-6-(4-bromo-4-fluorobutan-2-yl)-4,4a-dimethyl-4,4a,5,6,7,8 hexahydronaphthalen-2(3*H*)-one 37a** (101 MHz, $CDCl_3$)

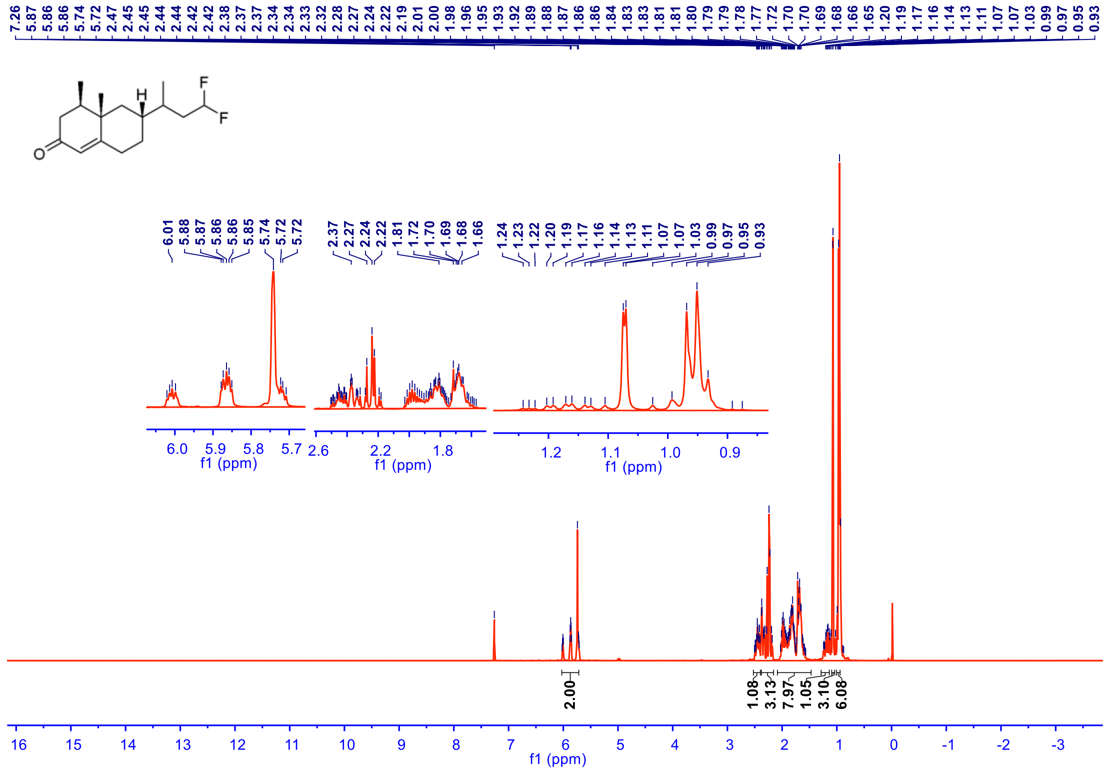

Supplementary Figure 350. ^{1}H NMR spectrum of compound **37** (400 MHz, $CDCl_3$)

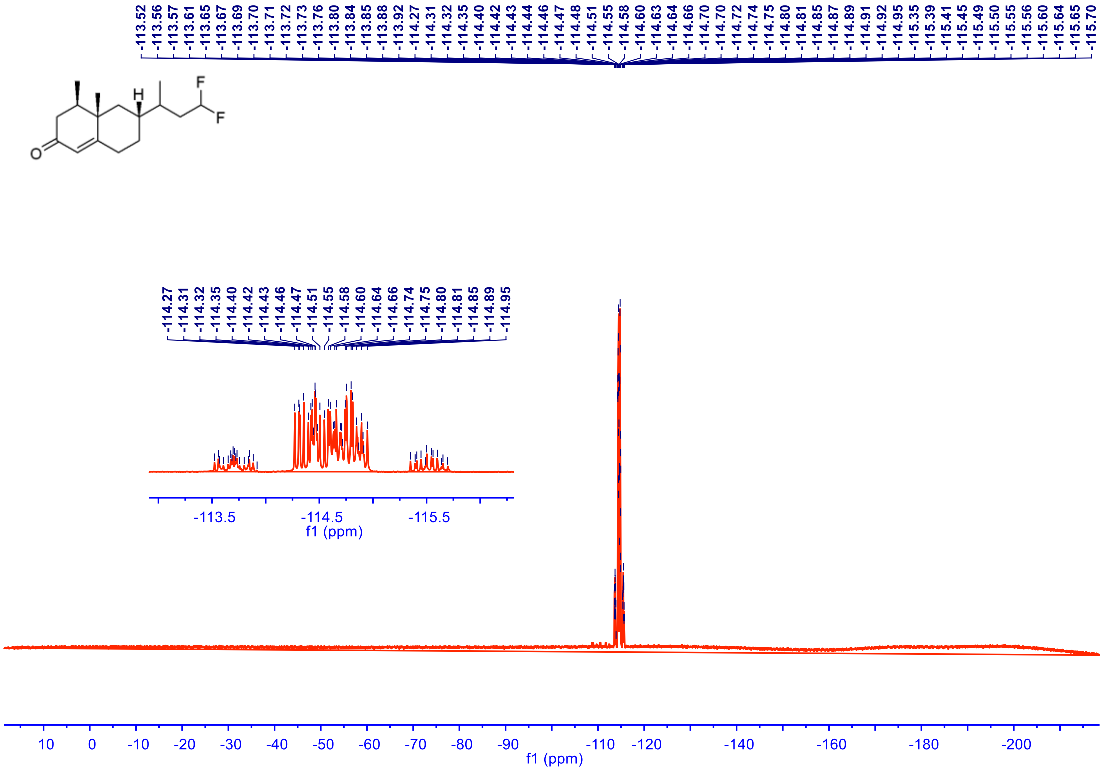

Supplementary Figure 351. ^{19}F NMR spectrum of compound **37** (376 MHz, $CDCl_3$)

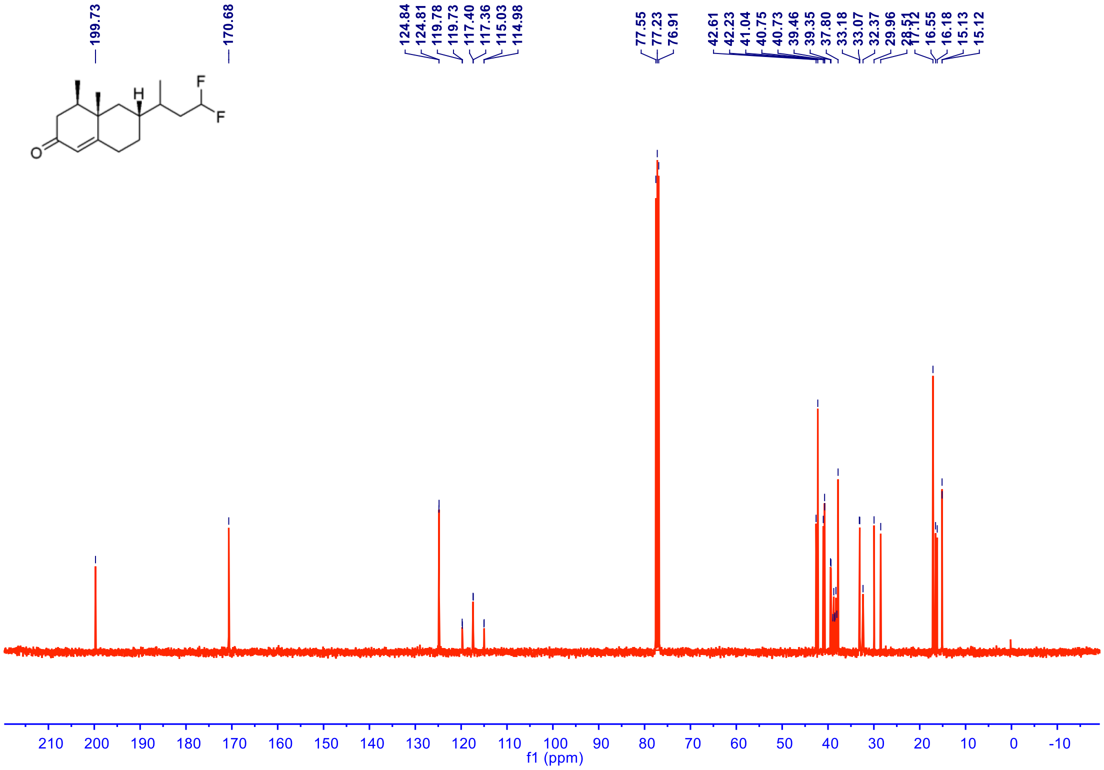

Supplementary Figure 352. ^{13}C NMR spectrum of compound **37** (101 MHz, $CDCl_3$)

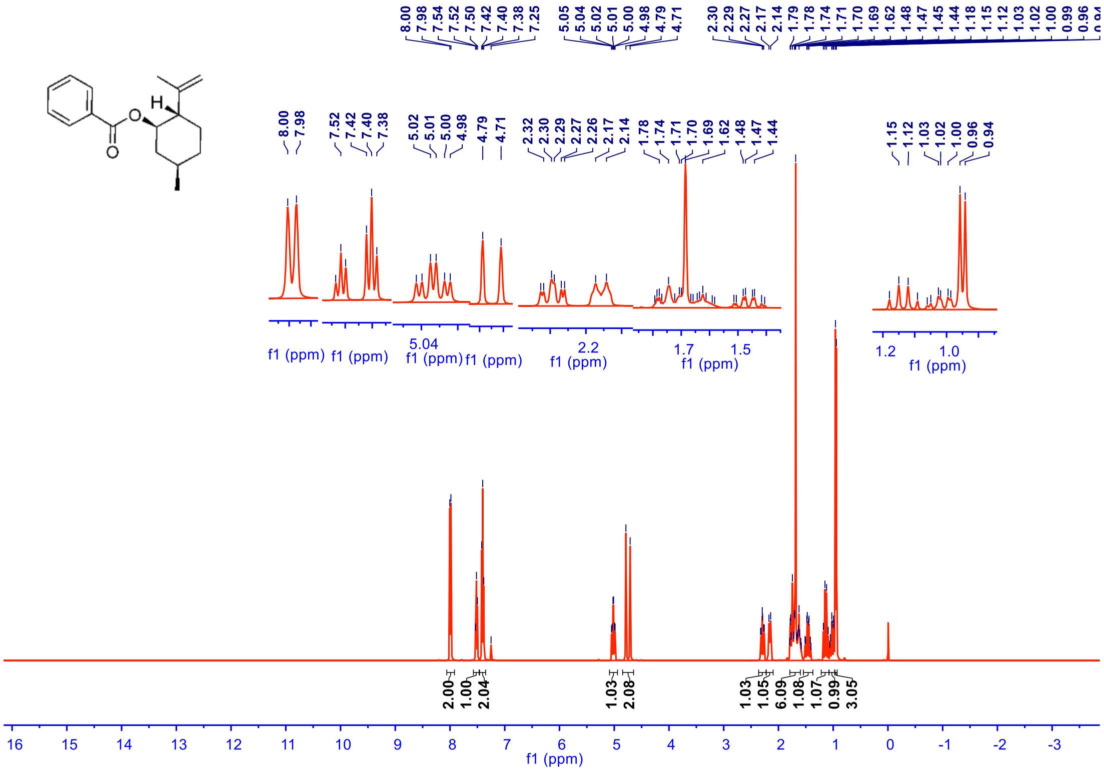

Supplementary Figure 353. ^{1}H NMR spectrum of **(1*R*,2*S*,5*R*)-5-methyl-2-(prop-1-en-2-yl)cyclohexyl benzoate 38b** (400 MHz, $CDCl_3$)

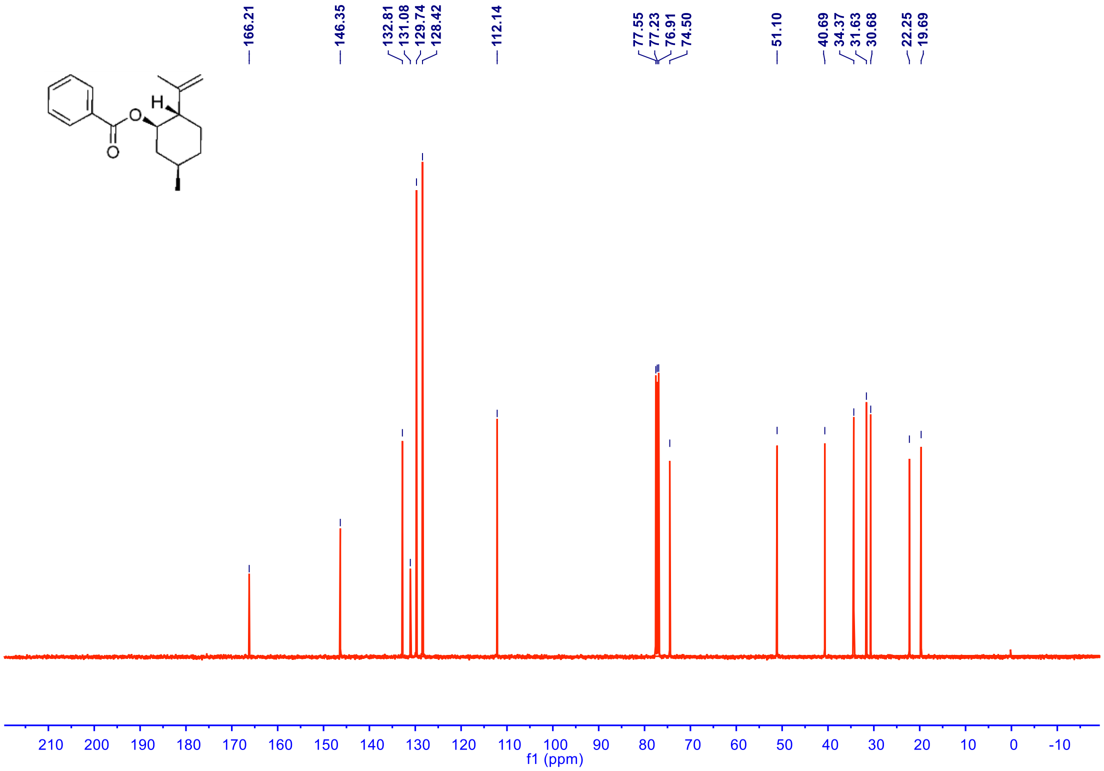

Supplementary Figure 354. ^{13}C NMR spectrum of **(1*R*,2*S*,5*R*)-5-methyl-2-(prop-1-en-2-yl)cyclohexyl benzoate 38b** (101 MHz, $CDCl_3$)

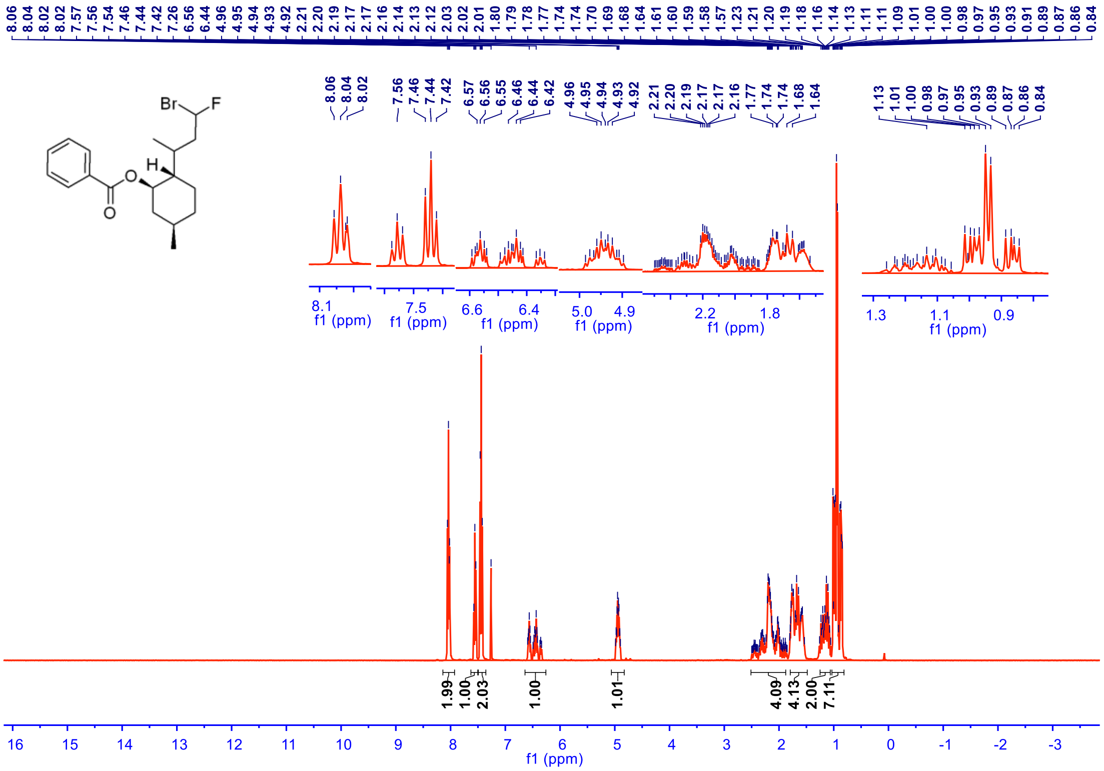

Supplementary Figure 355. ^{1}H NMR spectrum of **(1*R*,2*S*,5*R*)-2-(4-bromo-4-fluorobutan-2-yl)-5-methylcyclohexyl benzoate 38a** (400 MHz, $CDCl_3$)

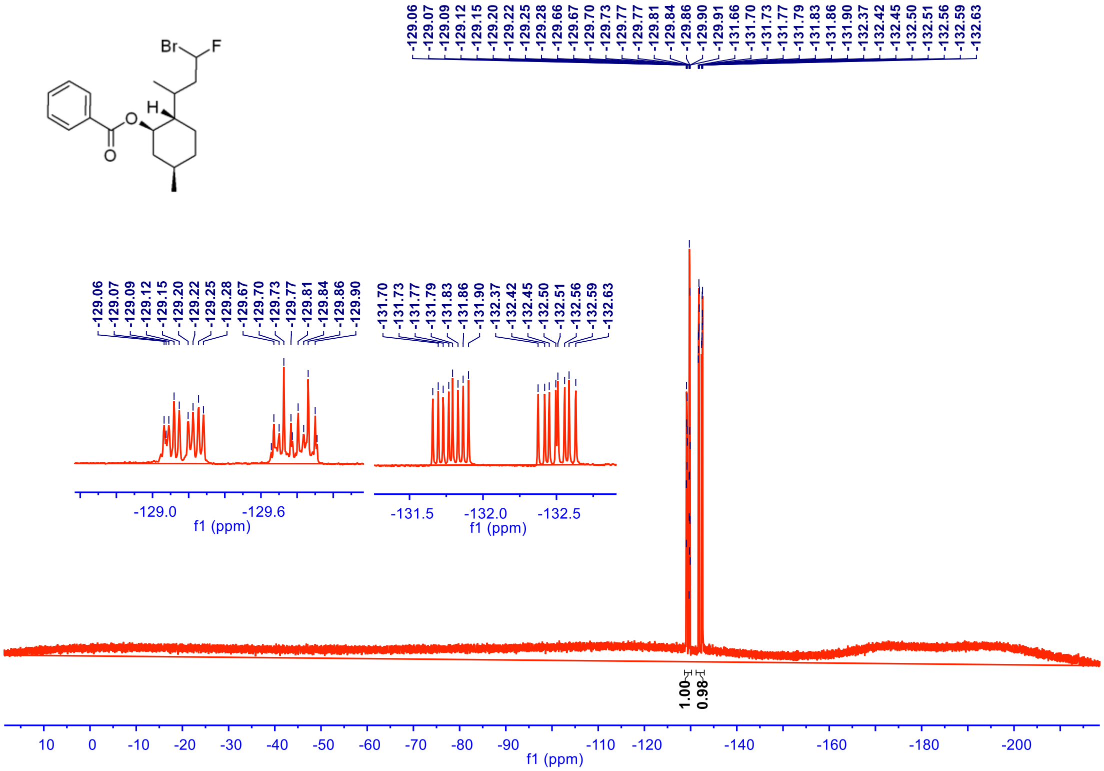

Supplementary Figure 356. ^{19}F NMR spectrum of **(1*R*,2*S*,5*R*)-2-(4-bromo-4-fluorobutan-2-yl)-5-methylcyclohexyl benzoate 38a** (376 MHz, $CDCl_3$)

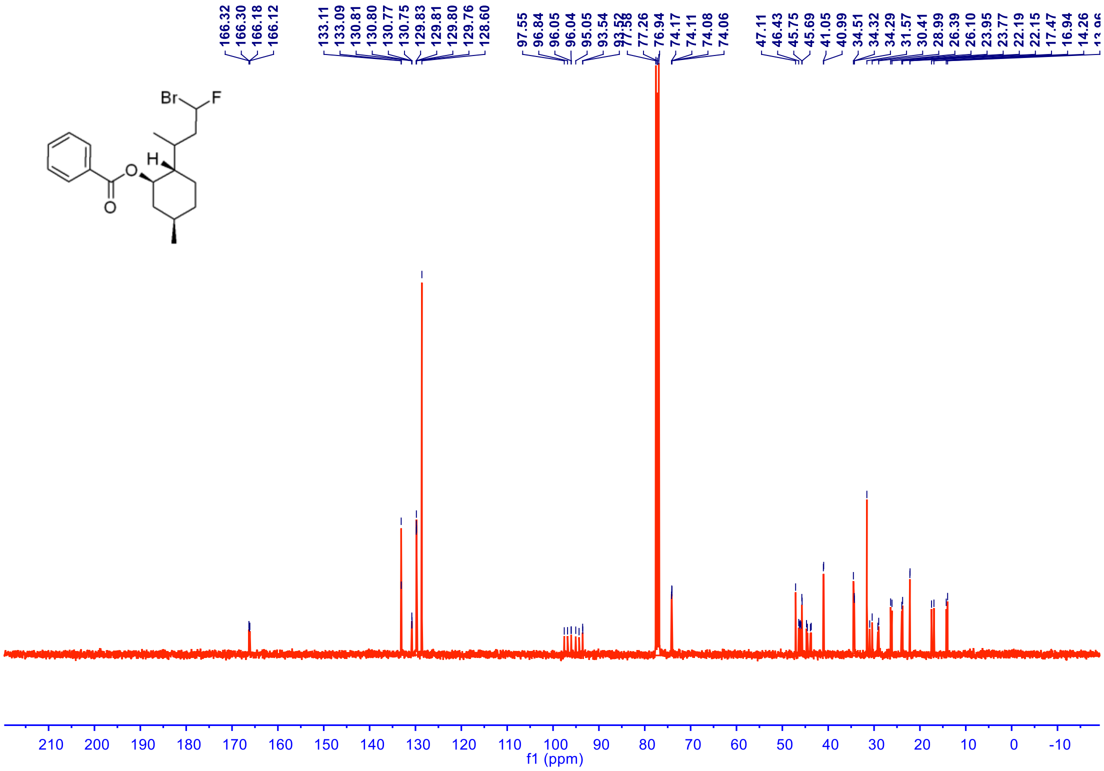

Supplementary Figure 357. ^{13}C NMR spectrum of **(1*R*,2*S*,5*R*)-2-(4-bromo-4-fluorobutan-2-yl)-5-methylcyclohexyl benzoate 38a** (101 MHz, $CDCl_3$)

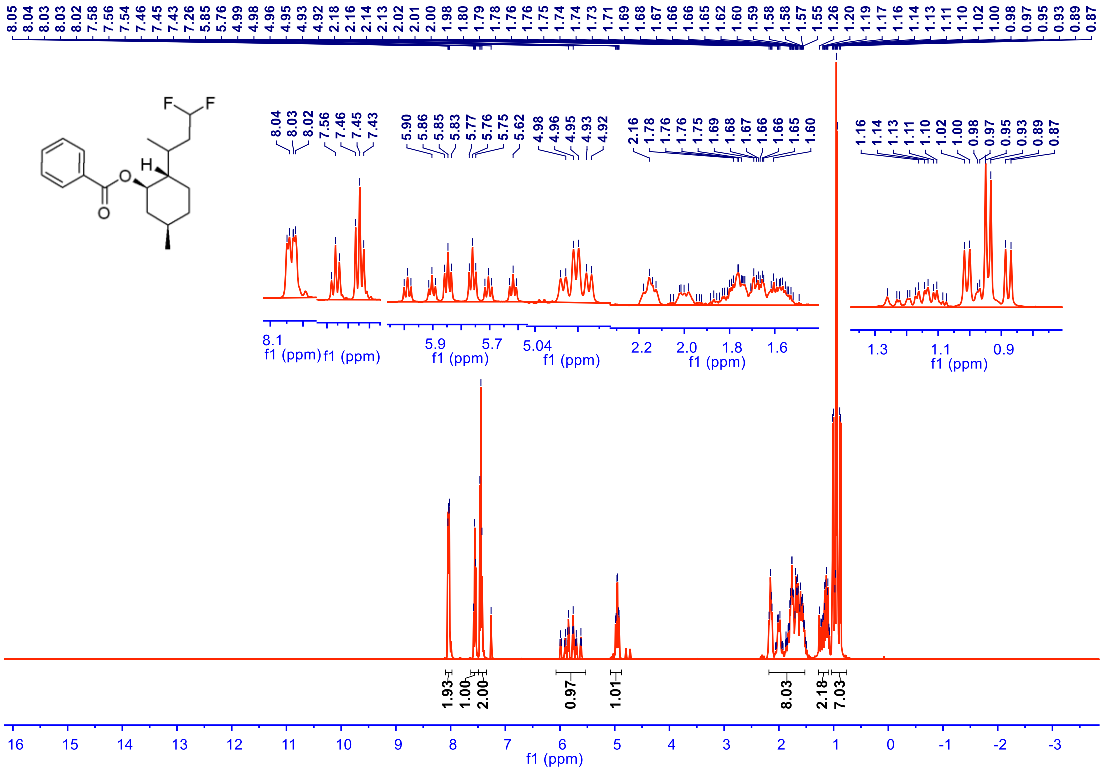

Supplementary Figure 358. ^{1}H NMR spectrum of compound **38** (400 MHz, $CDCl_3$)

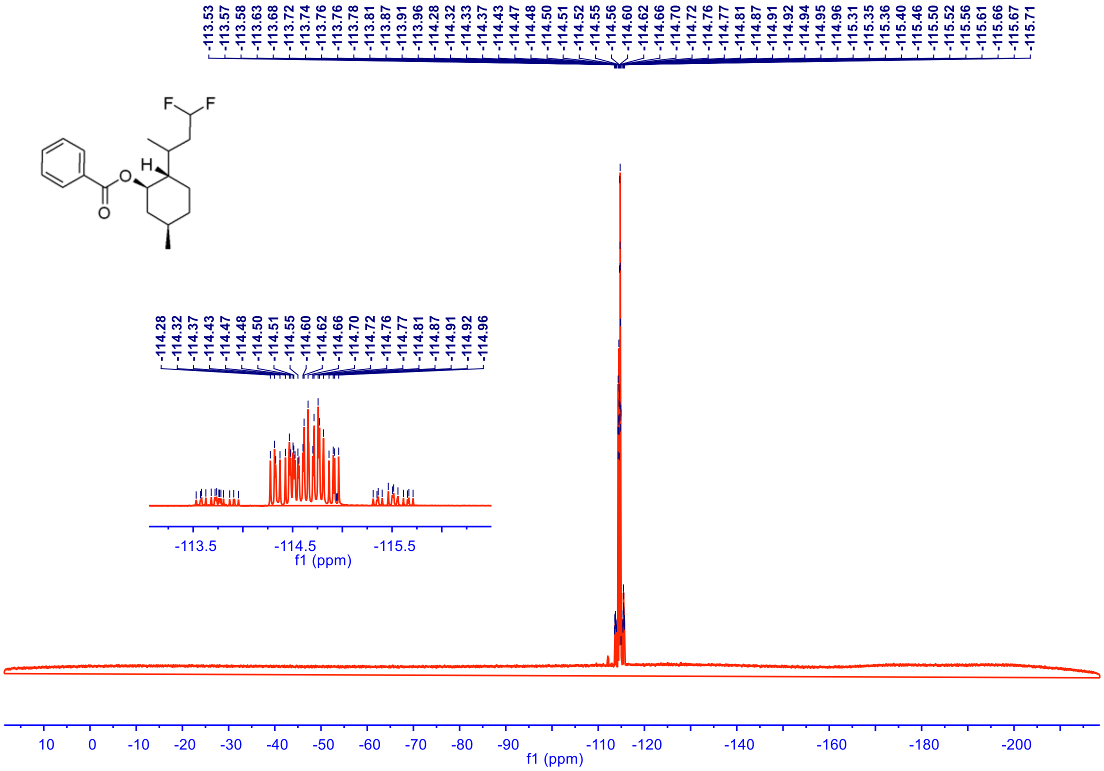

Supplementary Figure 359. ^{19}F NMR spectrum of compound **38** (376 MHz, $CDCl_3$)

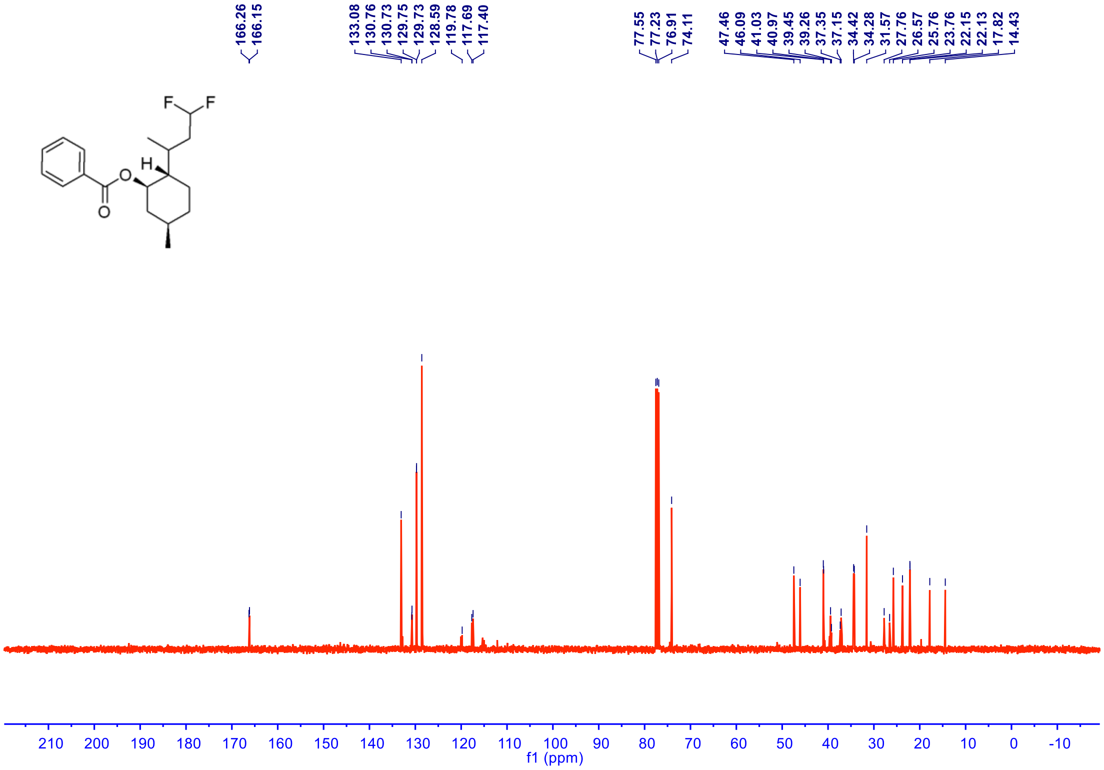

Supplementary Figure 360. ^{13}C NMR spectrum of compound **38** (101 MHz, $CDCl_3$)

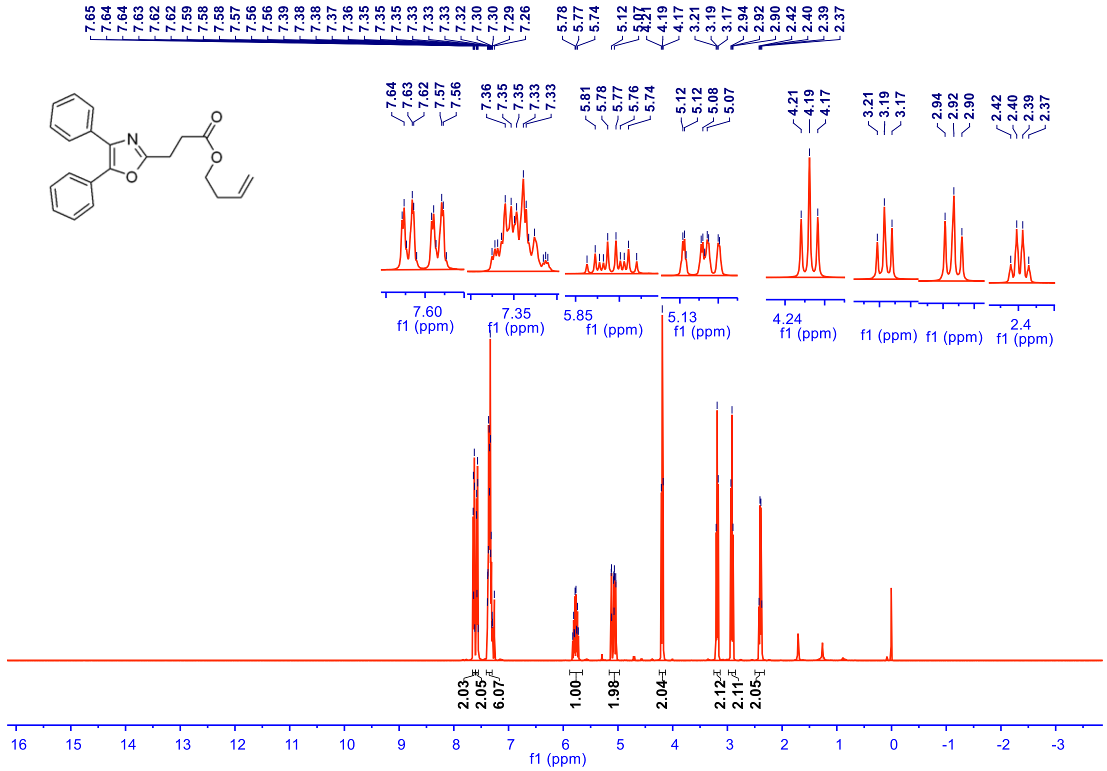

Supplementary Figure 361. ^{1}H NMR spectrum of **but-3-en-1-yl 3-(4,5-diphenyloxazol-2-yl)propanoate 39b** (400 MHz, $CDCl_3$)

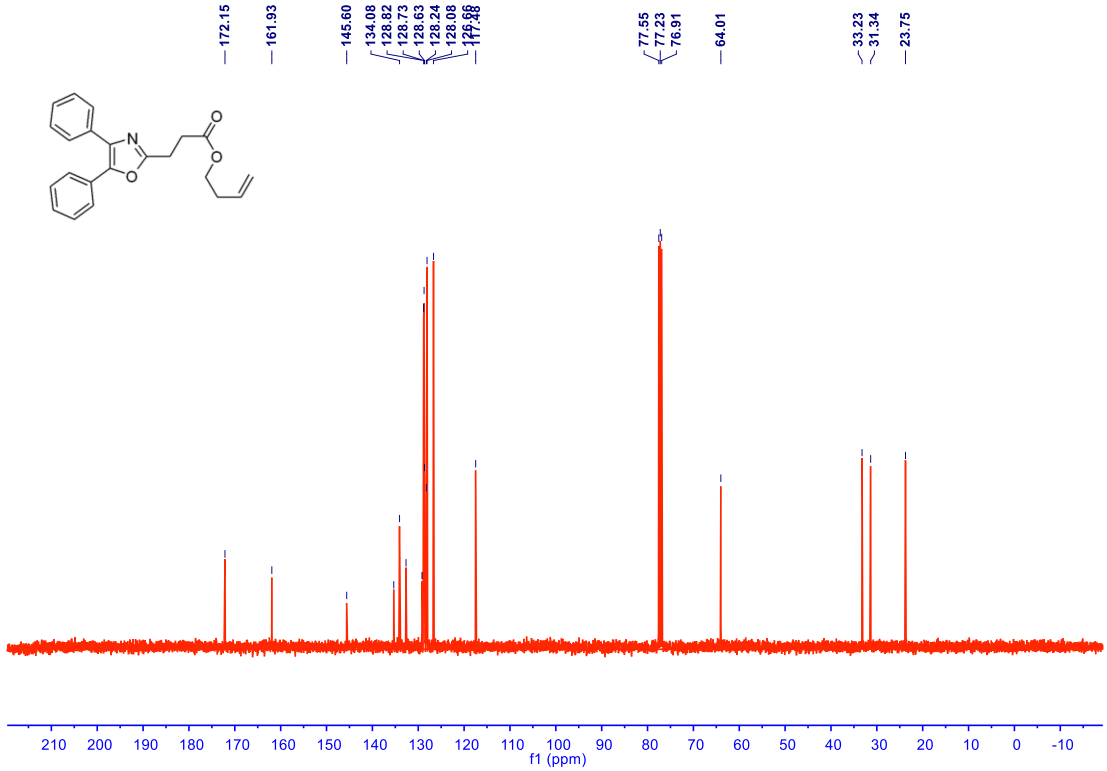

Supplementary Figure 362. ^{13}C NMR spectrum of **but-3-en-1-yl 3-(4,5-diphenyloxazol-2-yl)propanoate 39b** (101 MHz, $CDCl_3$)

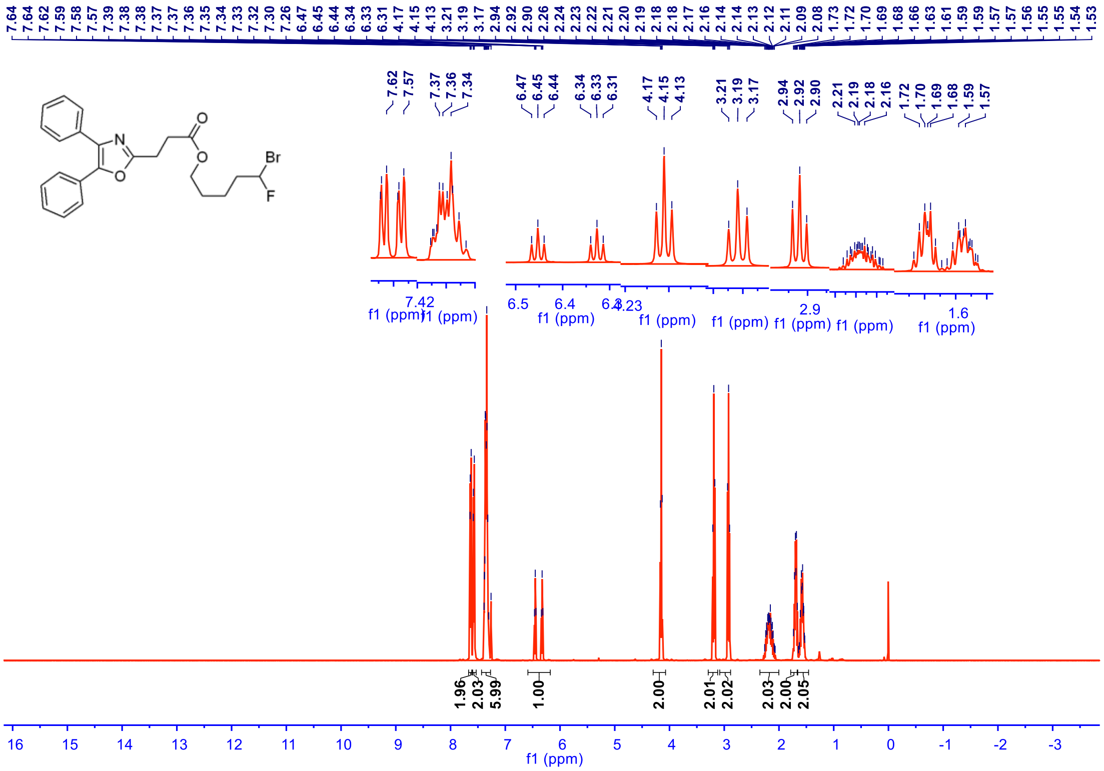

Supplementary Figure 363. ^{1}H NMR spectrum of **5-bromo-5-fluoropentyl 3-(4,5-diphenyloxazol-2-yl)propanoate 39a** (400 MHz, $CDCl_3$)

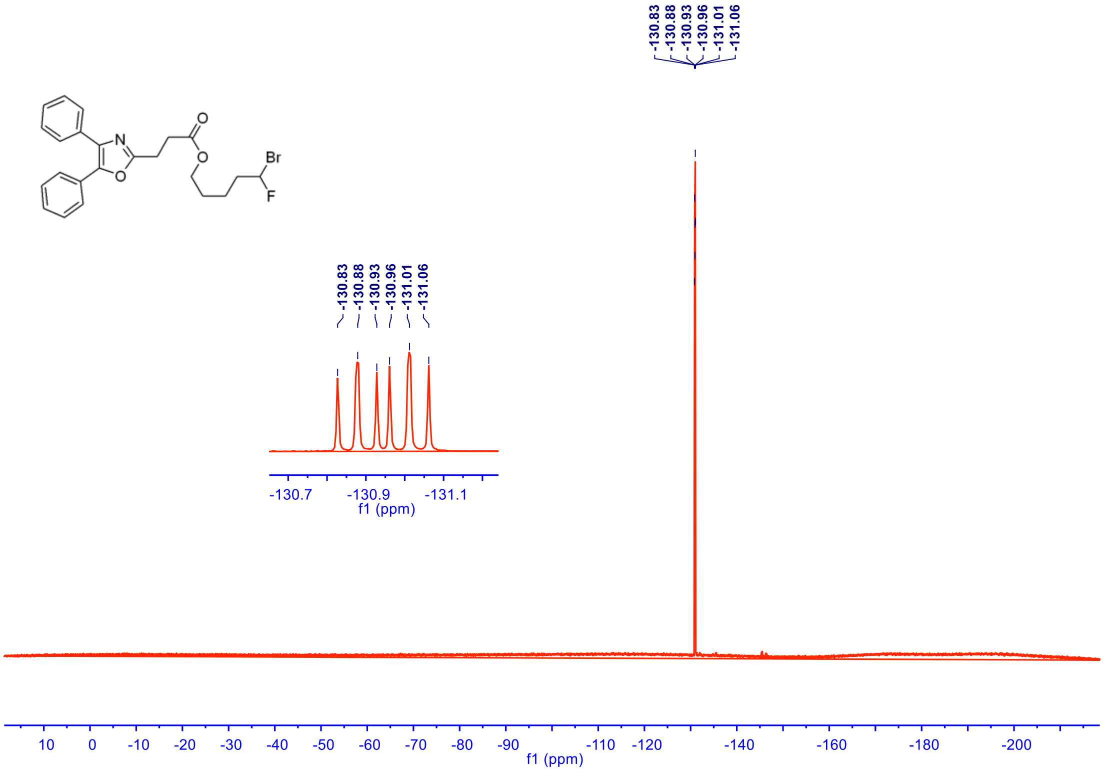

Supplementary Figure 364. ^{19}F NMR spectrum of **5-bromo-5-fluoropentyl 3-(4,5-diphenyloxazol-2-yl)propanoate 39a** (376 MHz, $CDCl_3$)

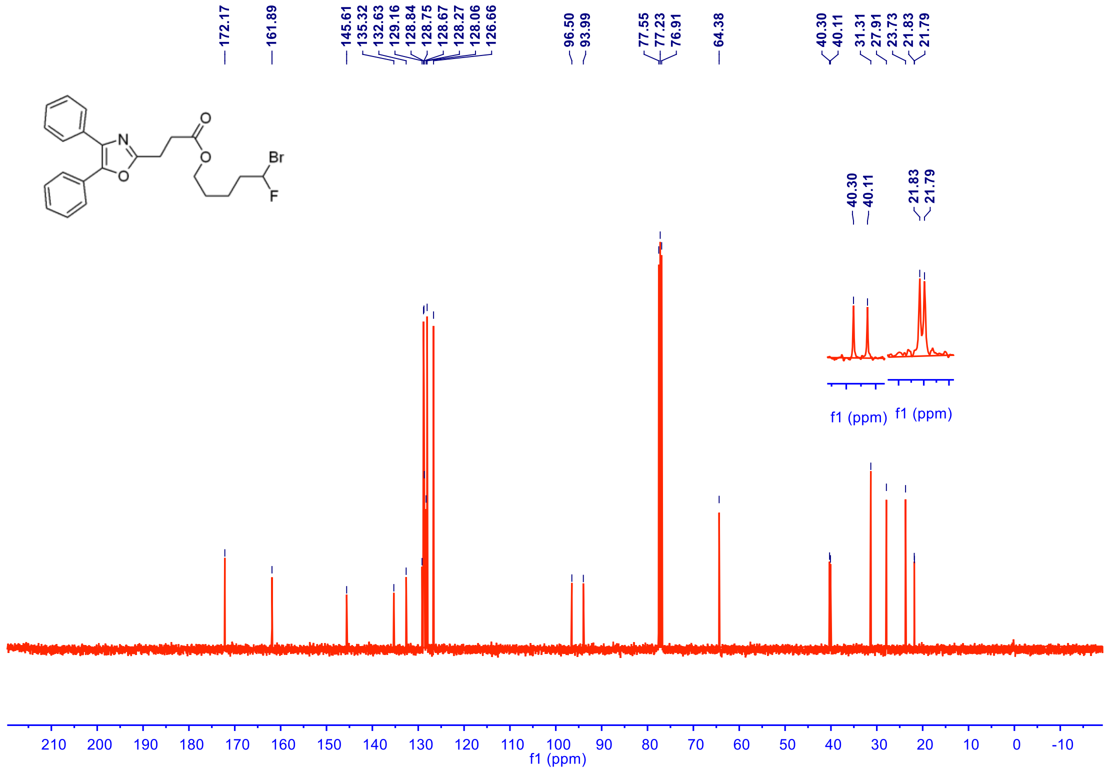

Supplementary Figure 365. ^{13}C NMR spectrum of **5-bromo-5-fluoropentyl 3-(4,5-diphenyloxazol-2-yl)propanoate 39a** (101 MHz, $CDCl_3$)

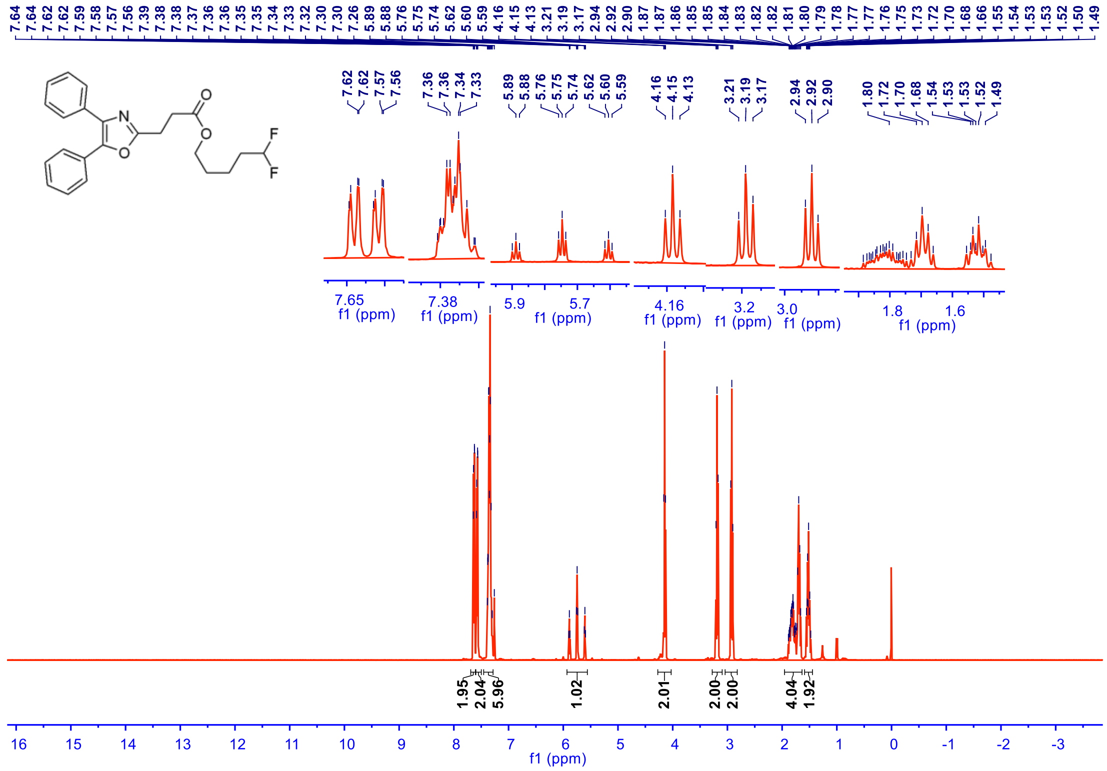

Supplementary Figure 366. ^{1}H NMR spectrum of compound **39** (400 MHz, $CDCl_3$)

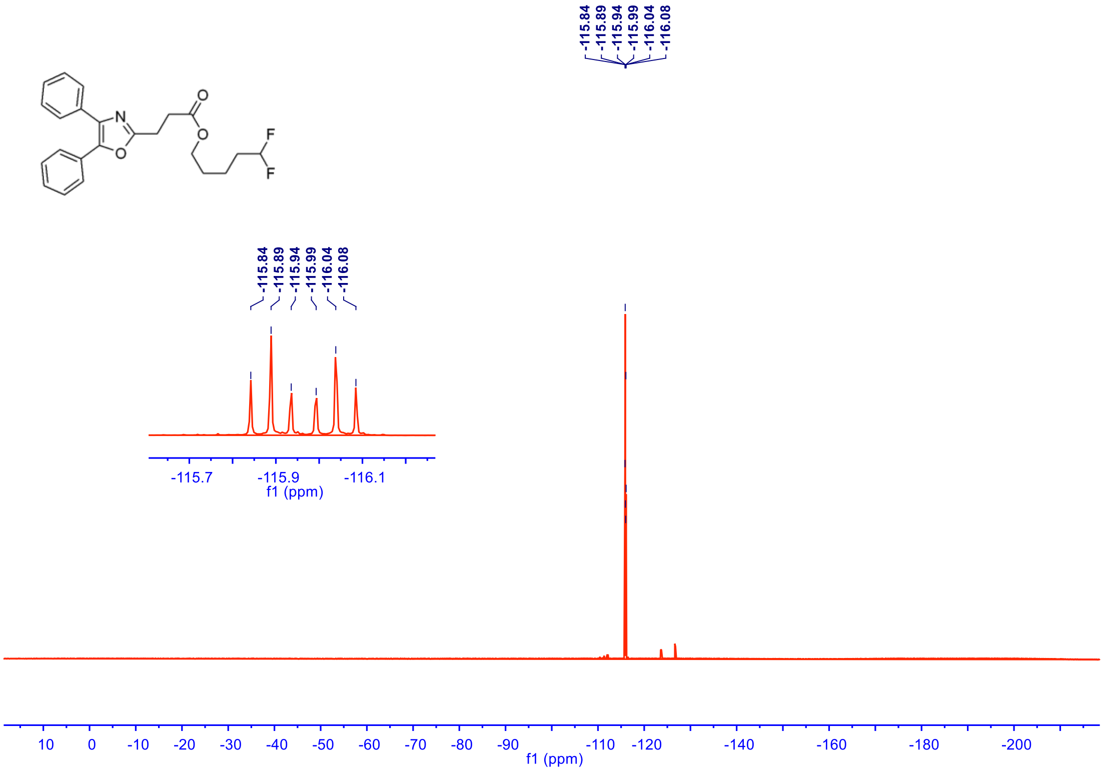

Supplementary Figure 367. ^{19}F NMR spectrum of compound **39** (376 MHz, $CDCl_3$)

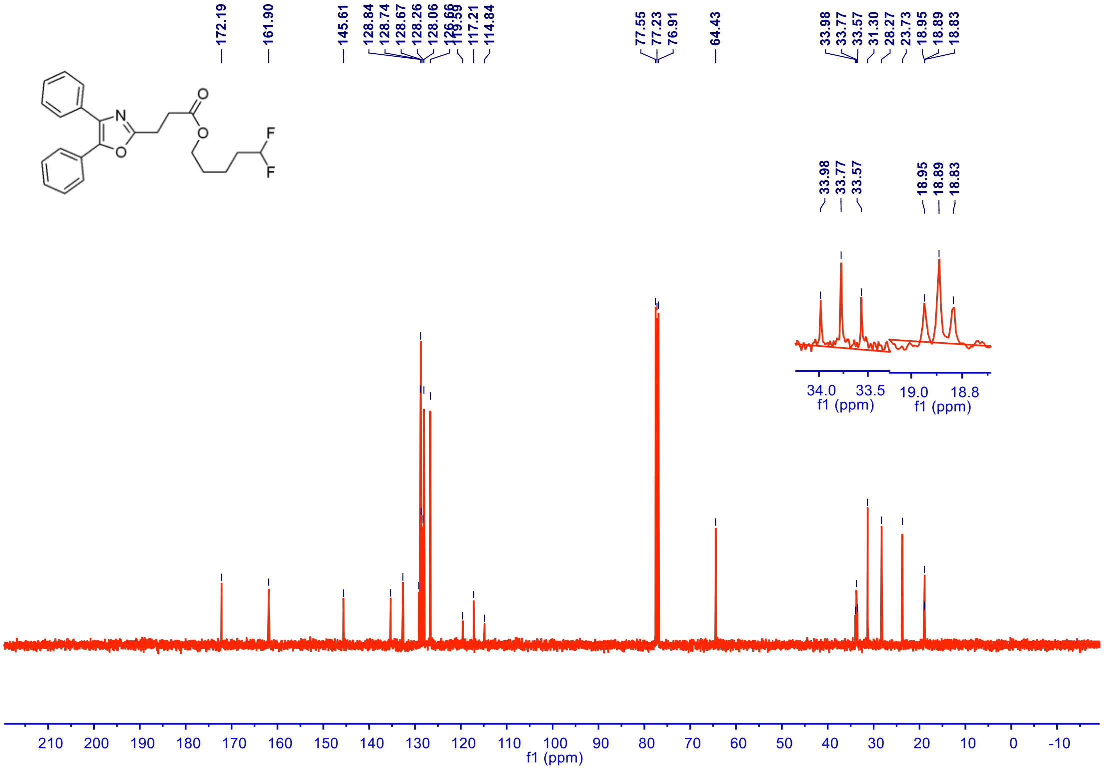

Supplementary Figure 368. ^{13}C NMR spectrum of compound **39** (101 MHz, $CDCl_3$)

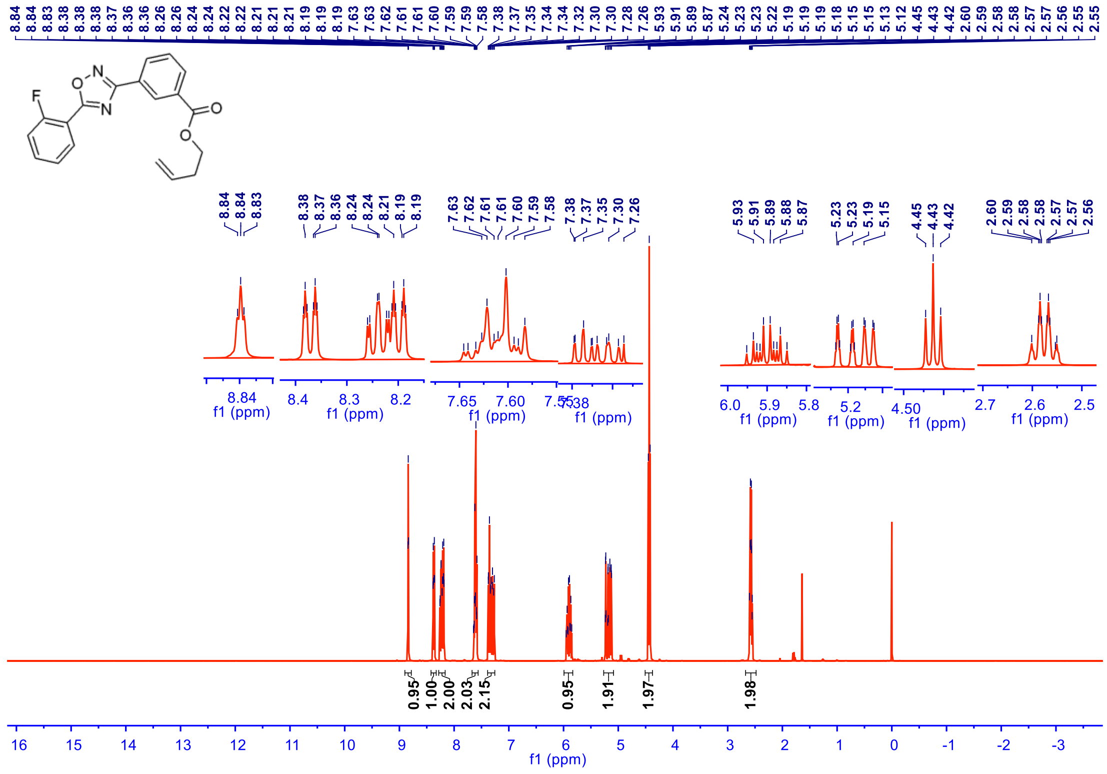

Supplementary Figure 369. ^{1}H NMR spectrum of **but-3-en-1-yl 3-(5-(2-fluorophenyl)-1,2,4-oxadiazol-3-yl)benzoate 40b** (400 MHz, $CDCl_3$)

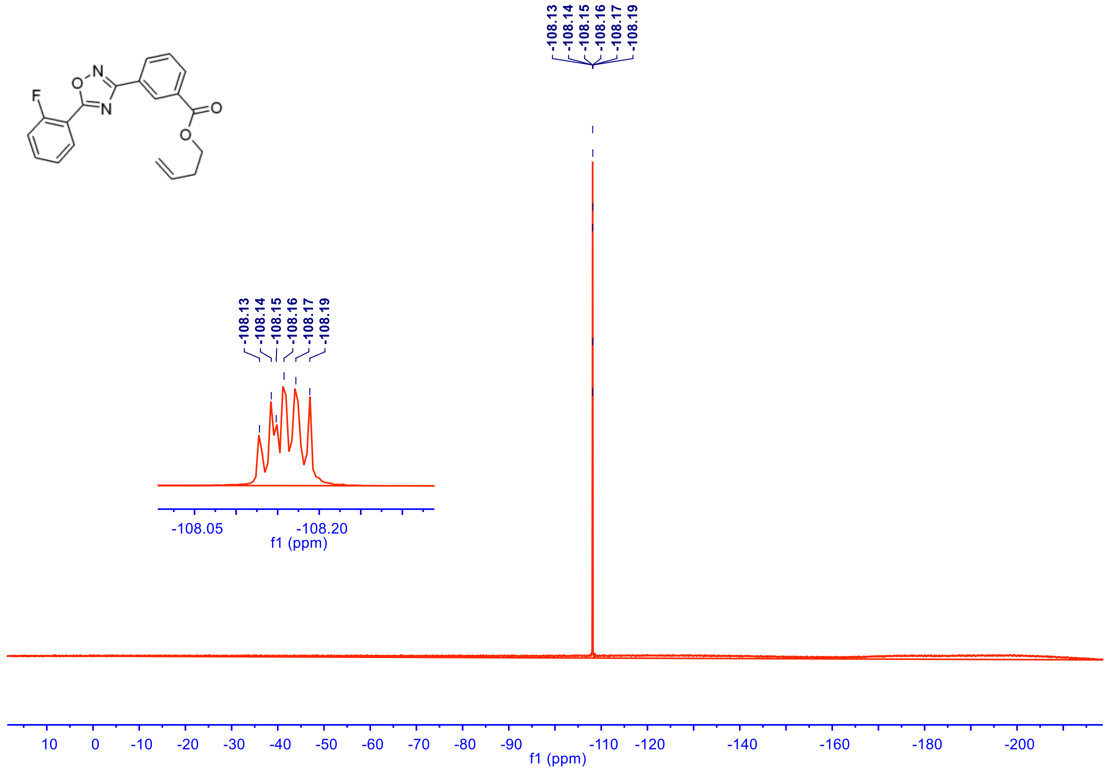

Supplementary Figure 370. ^{19}F NMR spectrum of **but-3-en-1-yl 3-(5-(2-fluorophenyl)-1,2,4-oxadiazol-3-yl)benzoate 40b** (376 MHz, $CDCl_3$)

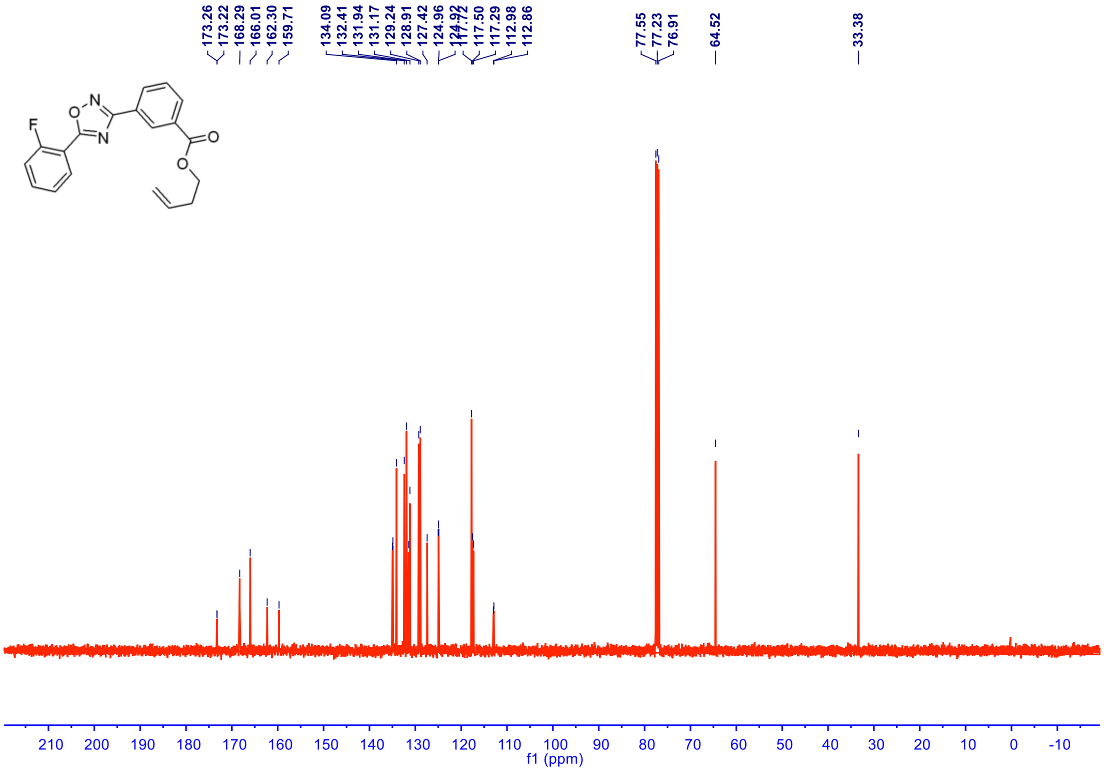

Supplementary Figure 371. ^{13}C NMR spectrum of **but-3-en-1-yl 3-(5-(2-fluorophenyl)-1,2,4-oxadiazol-3-yl)benzoate 40b** (101 MHz, $CDCl_3$)

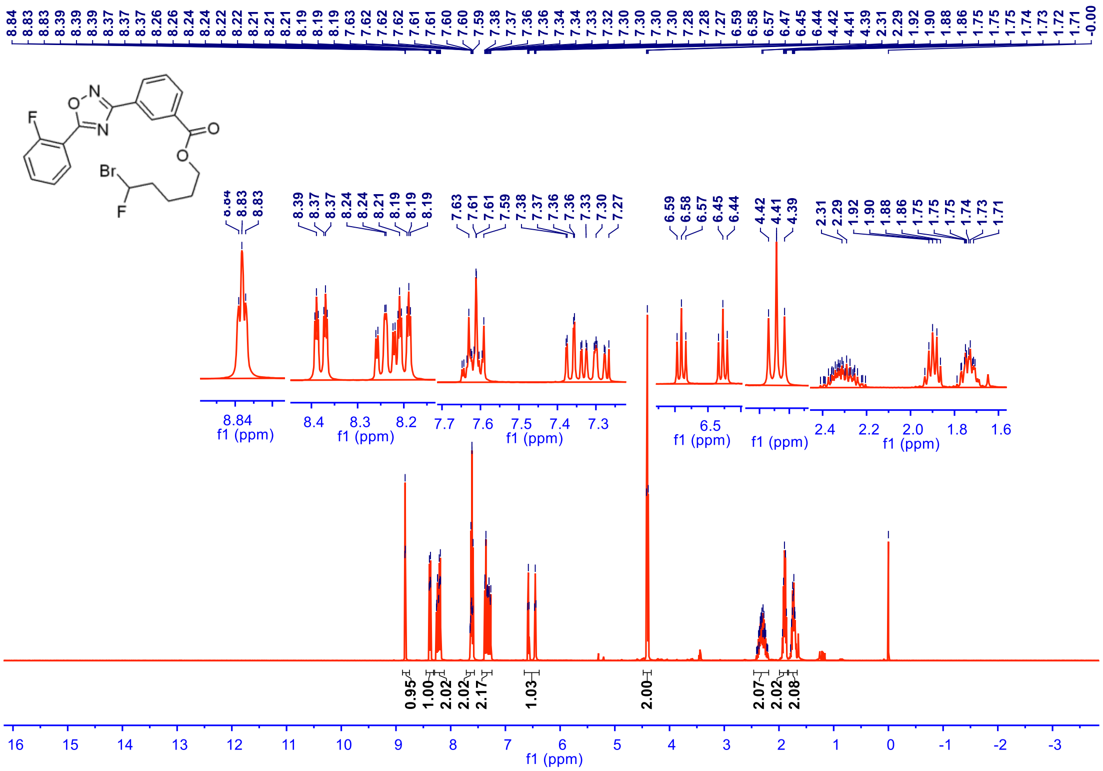

Supplementary Figure 372. ^{1}H NMR spectrum of **5-bromo-5-fluoropentyl 3-(5-(2-fluorophenyl)-1,2,4-oxadiazol-3-yl)benzoate 40a** (400 MHz, $CDCl_3$)

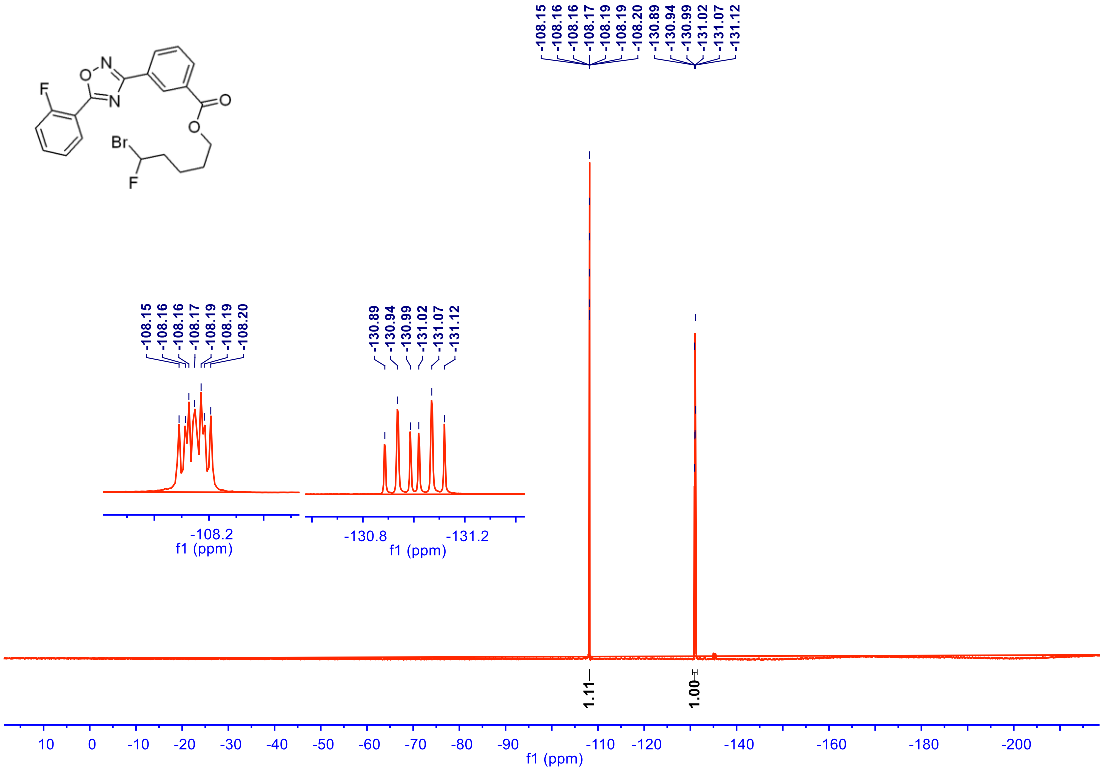

Supplementary Figure 373. ^{19}F NMR spectrum of **5-bromo-5-fluoropentyl 3-(5-(2-fluorophenyl)-1,2,4-oxadiazol-3-yl)benzoate 40a** (376 MHz, $CDCl_3$)

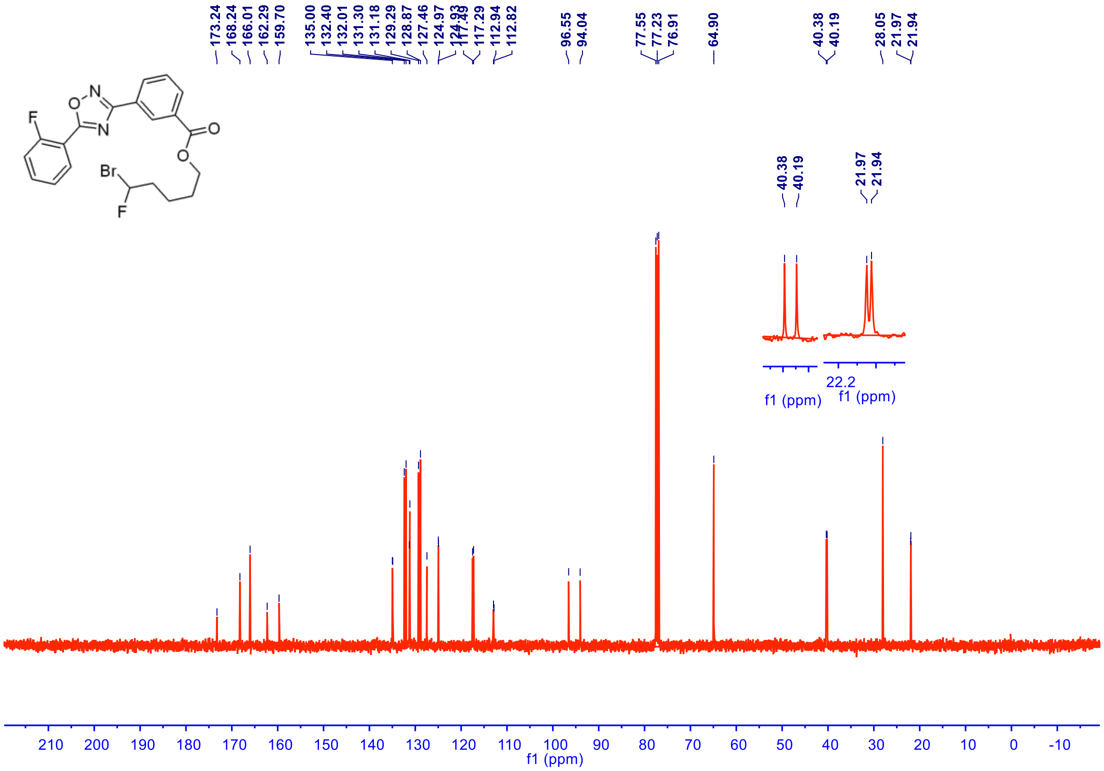

Supplementary Figure 374. ^{13}C NMR spectrum of **5-bromo-5-fluoropentyl 3-(5-(2-fluorophenyl)-1,2,4-oxadiazol-3-yl)benzoate 40a** (101 MHz, $CDCl_3$)

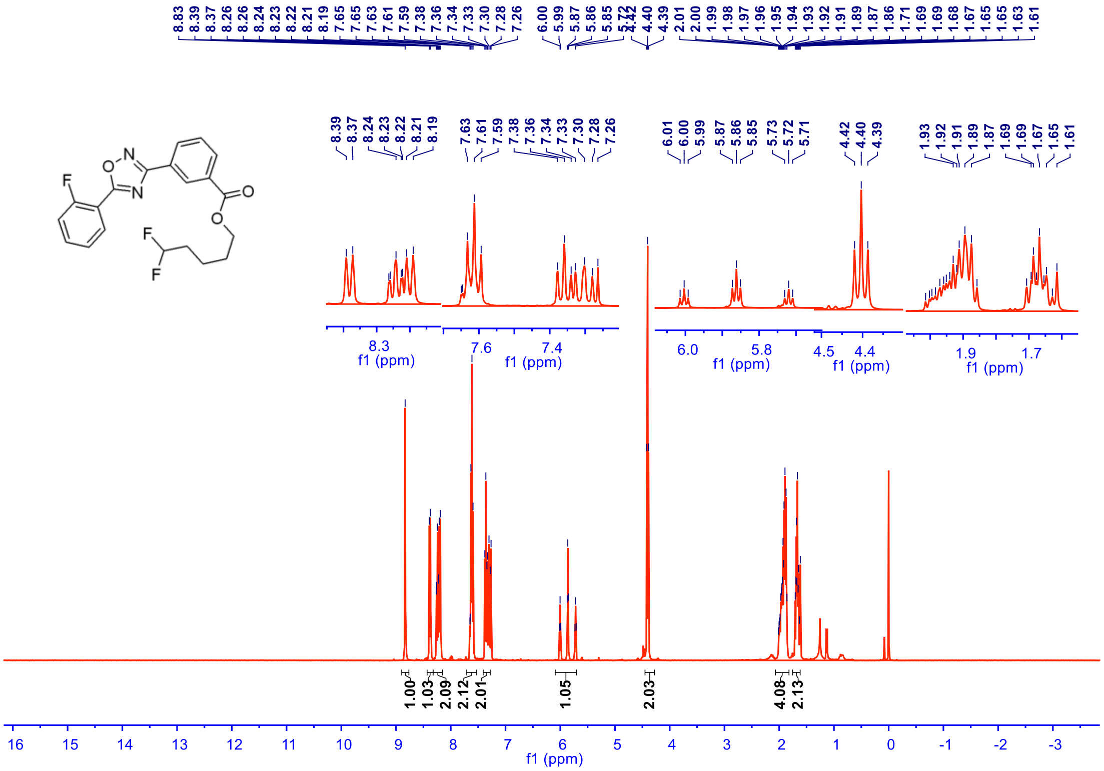

Supplementary Figure 375. ^{1}H NMR spectrum of compound **40** (400 MHz, $CDCl_3$)

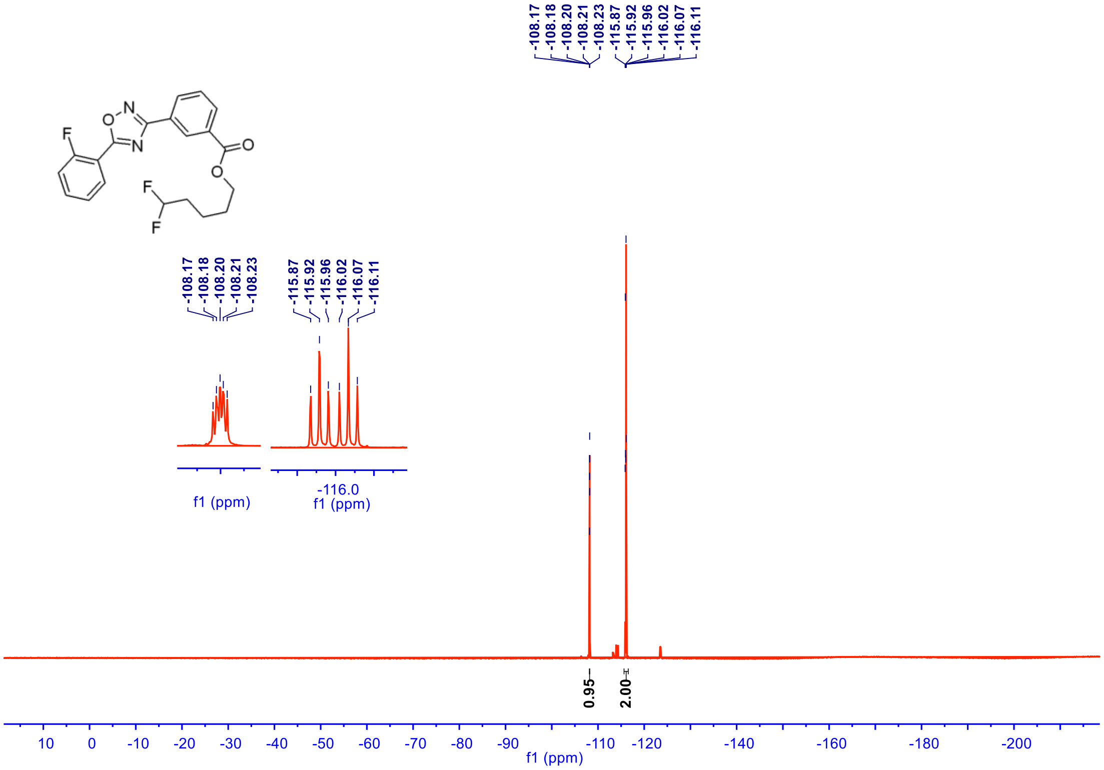

Supplementary Figure 376. ^{19}F NMR spectrum of compound **40** (376 MHz, $CDCl_3$)

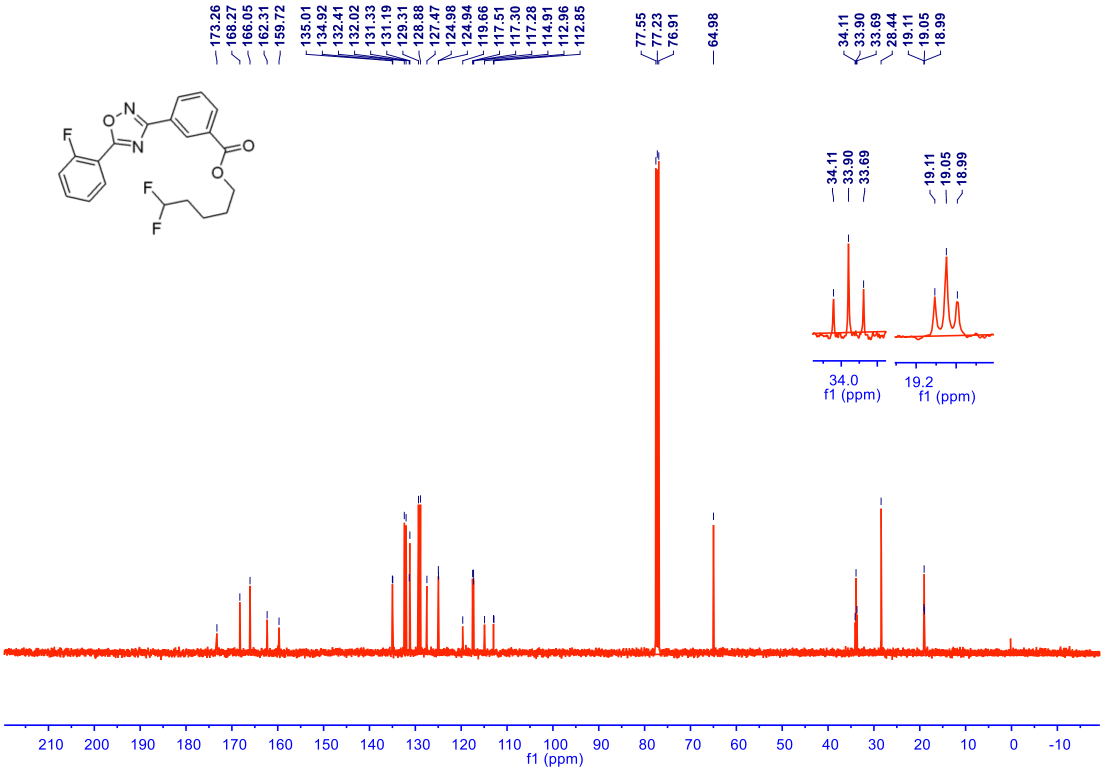

Supplementary Figure 377. ^{13}C NMR spectrum of compound **40** (101 MHz, $CDCl_3$)

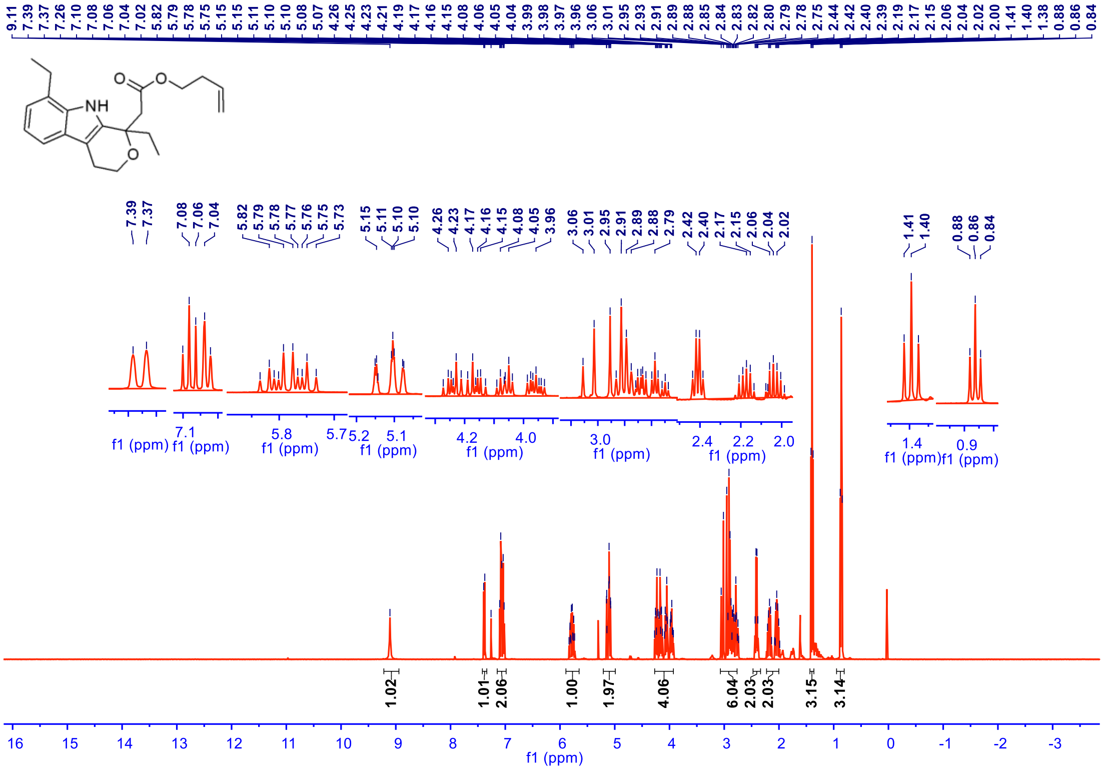

Supplementary Figure 378. ^{1}H NMR spectrum of **but-3-en-1-yl 2-(1,8-diethyl-1,3,4,9-tetrahydropyrano[3,4-*b*]indol-1-yl)acetate 41b** (400 MHz, $CDCl_3$)

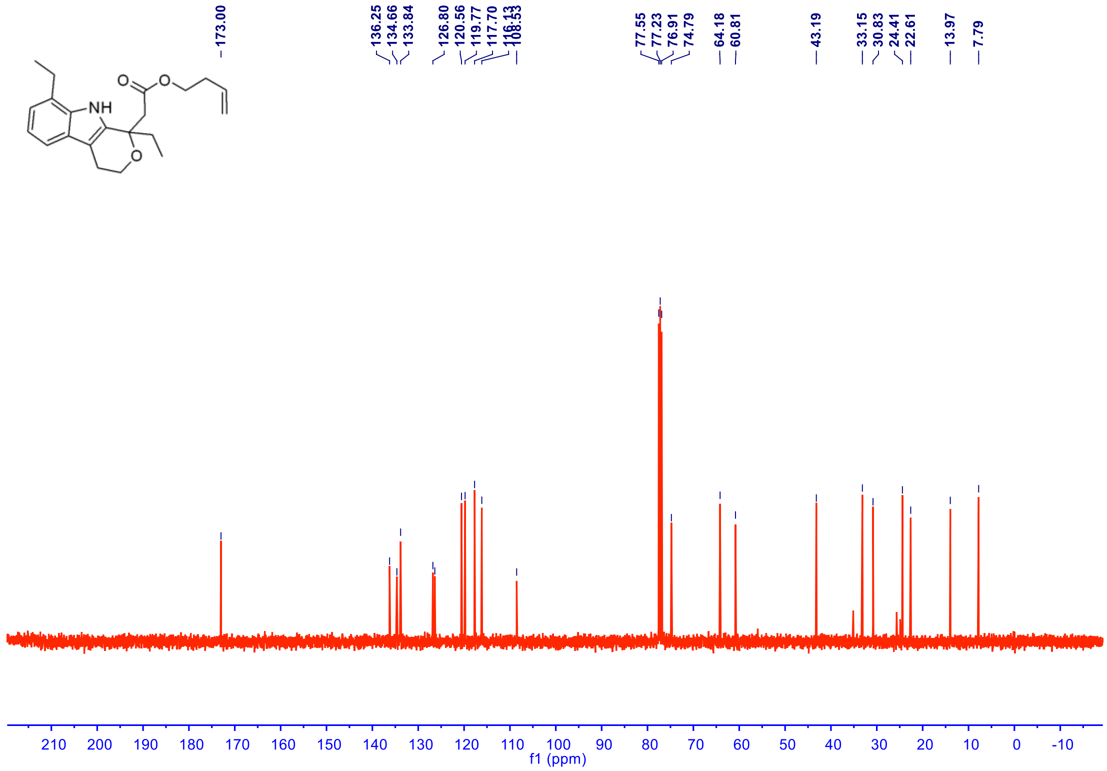

Supplementary Figure 379. ^{13}C NMR spectrum of **but-3-en-1-yl 2-(1,8-diethyl-1,3,4,9-tetrahydropyrano[3,4-*b*]indol-1-yl)acetate 41b** (101 MHz, $CDCl_3$)

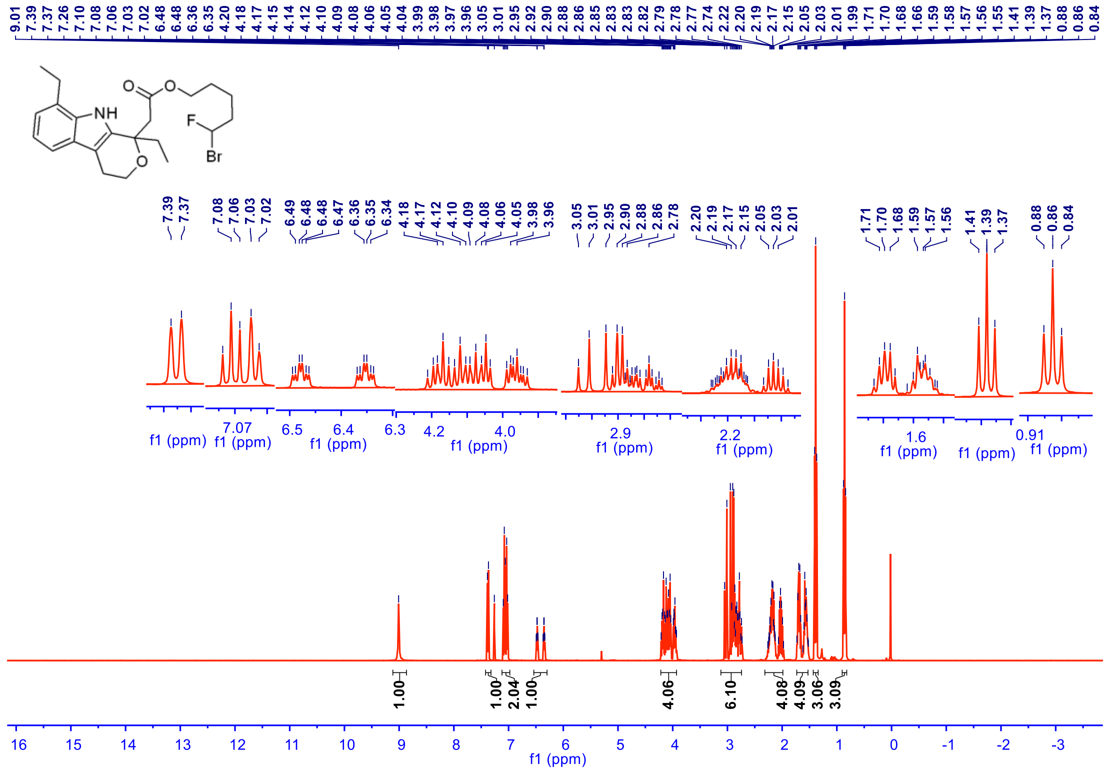

Supplementary Figure 380. ^{1}H NMR spectrum of **5-bromo-5-fluoropentyl 2-(1,8-diethyl-1,3,4,9-tetrahydropyrano[3,4-*b*]indol-1-yl)acetate 41a** (400 MHz, $CDCl_3$)

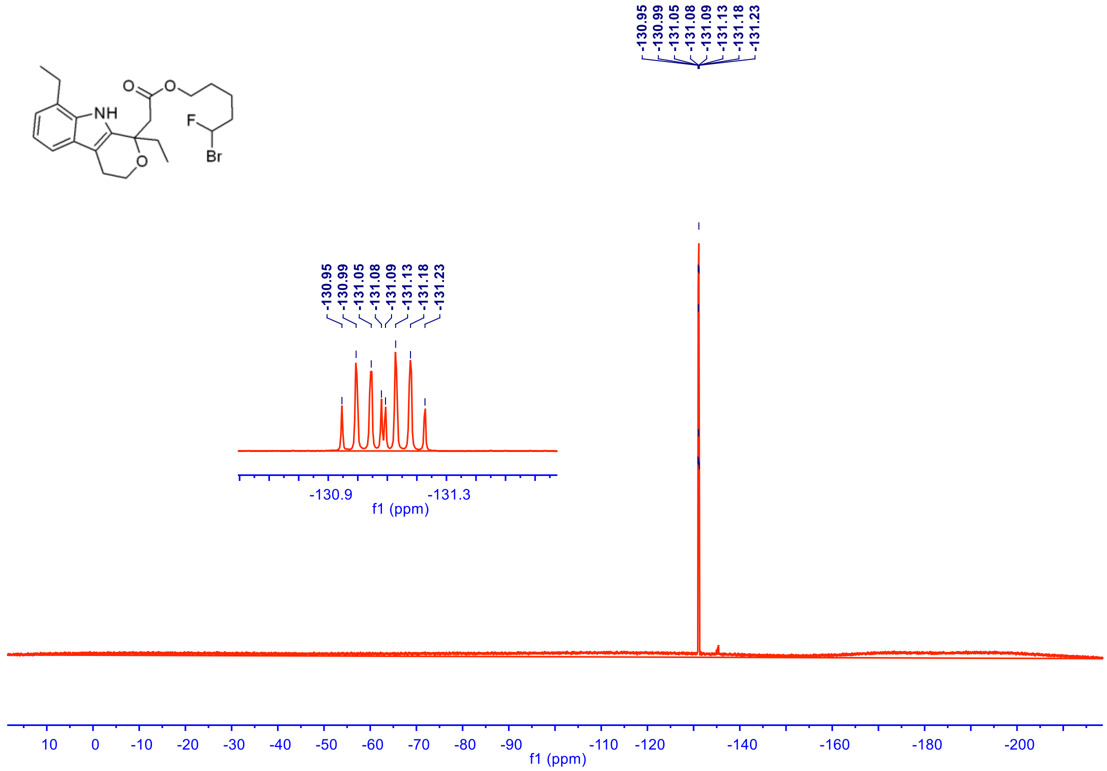

Supplementary Figure 381. ^{19}F NMR spectrum of **5-bromo-5-fluoropentyl 2-(1,8-diethyl-1,3,4,9-tetrahydropyrano[3,4-*b*]indol-1-yl)acetate 41a** (376 MHz, $CDCl_3$)

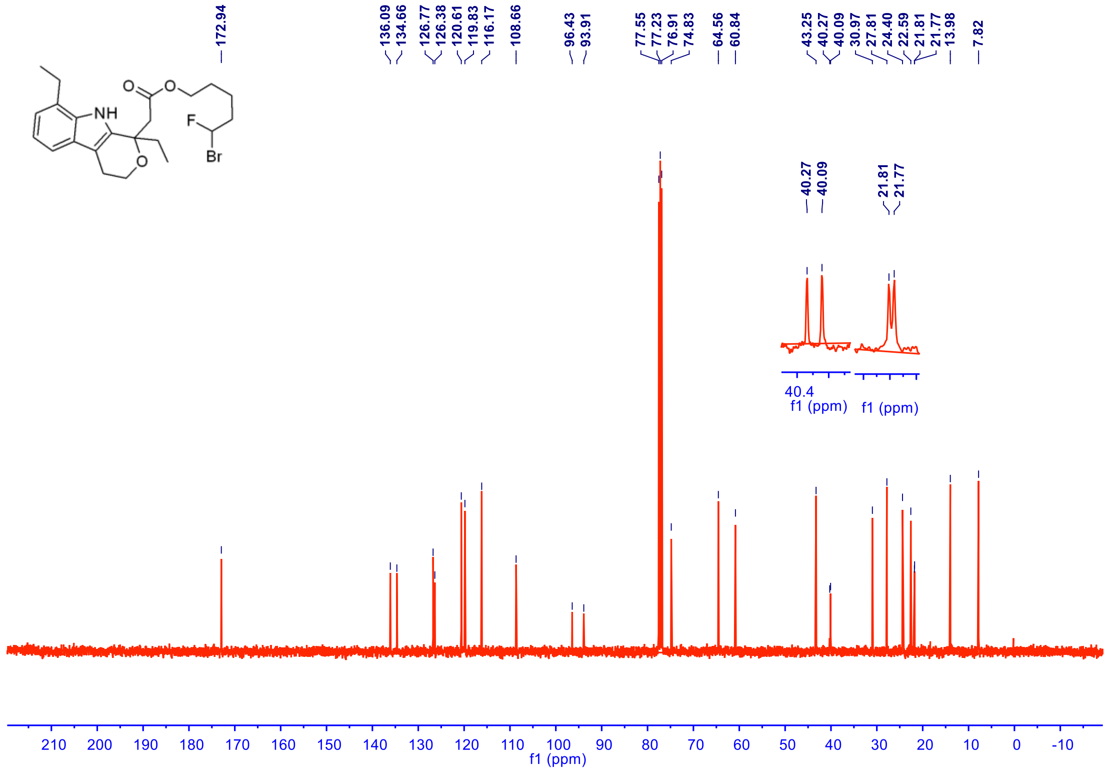

Supplementary Figure 382. ^{13}C NMR spectrum of **5-bromo-5-fluoropentyl 2-(1,8-diethyl-1,3,4,9-tetrahydropyrano[3,4-*b*]indol-1-yl)acetate 41a** (101 MHz, $CDCl_3$)

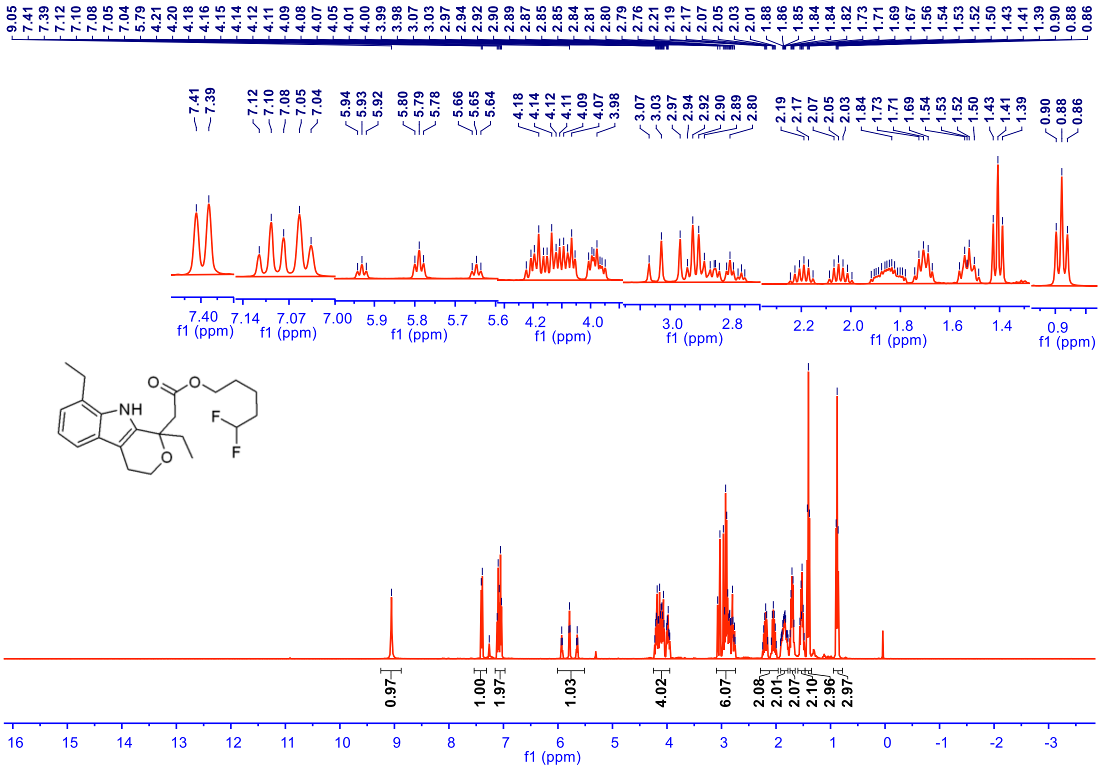

Supplementary Figure 383. ^{1}H NMR spectrum of compound **41** (400 MHz, $CDCl_3$)

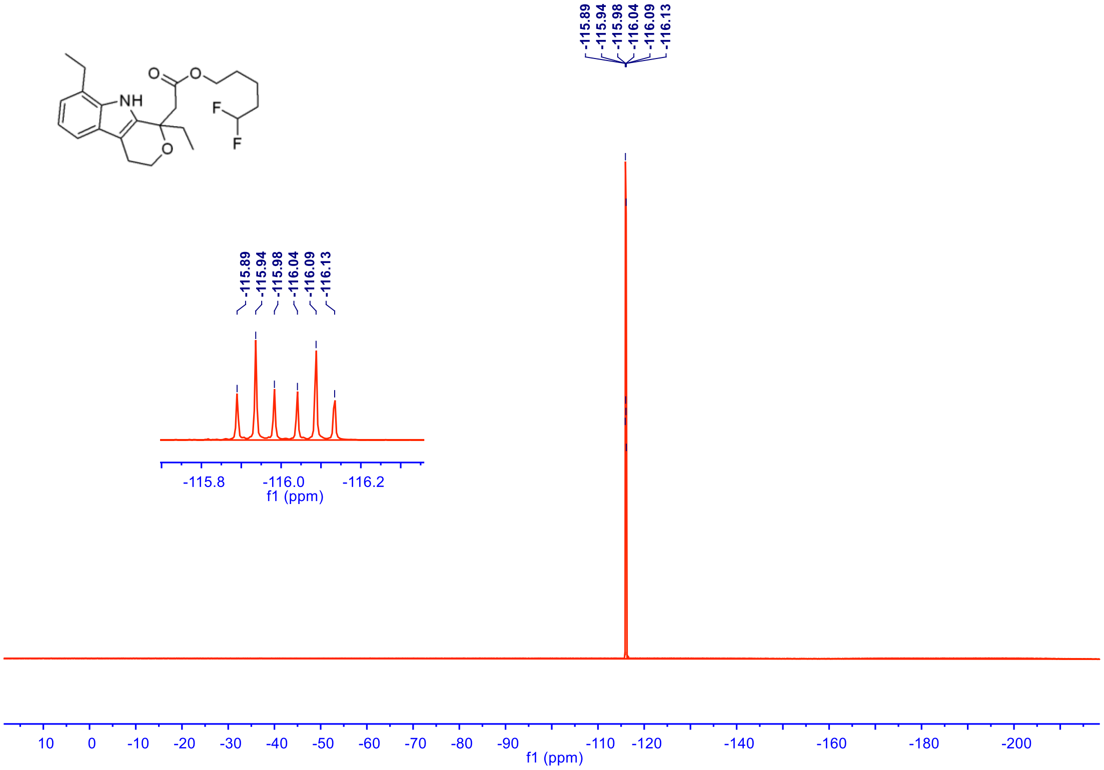

Supplementary Figure 384. ^{19}F NMR spectrum of compound **41** (376 MHz, $CDCl_3$)

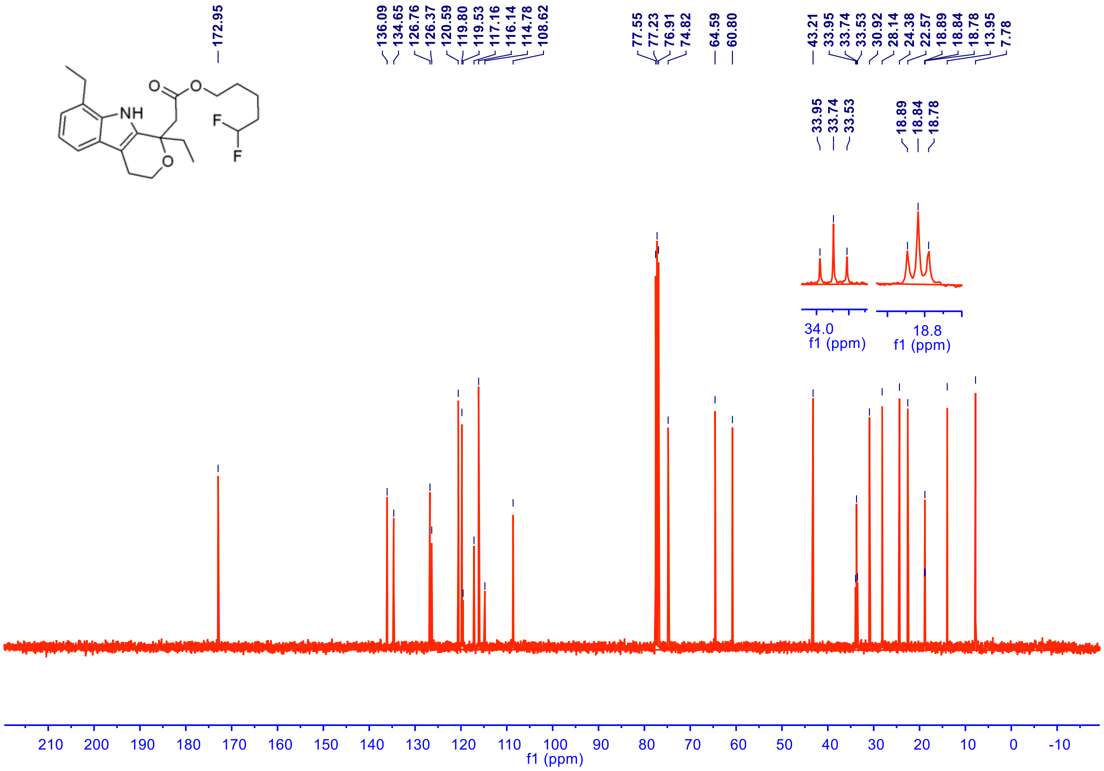

Supplementary Figure 385. ^{13}C NMR spectrum of compound **41** (101 MHz, $CDCl_3$)

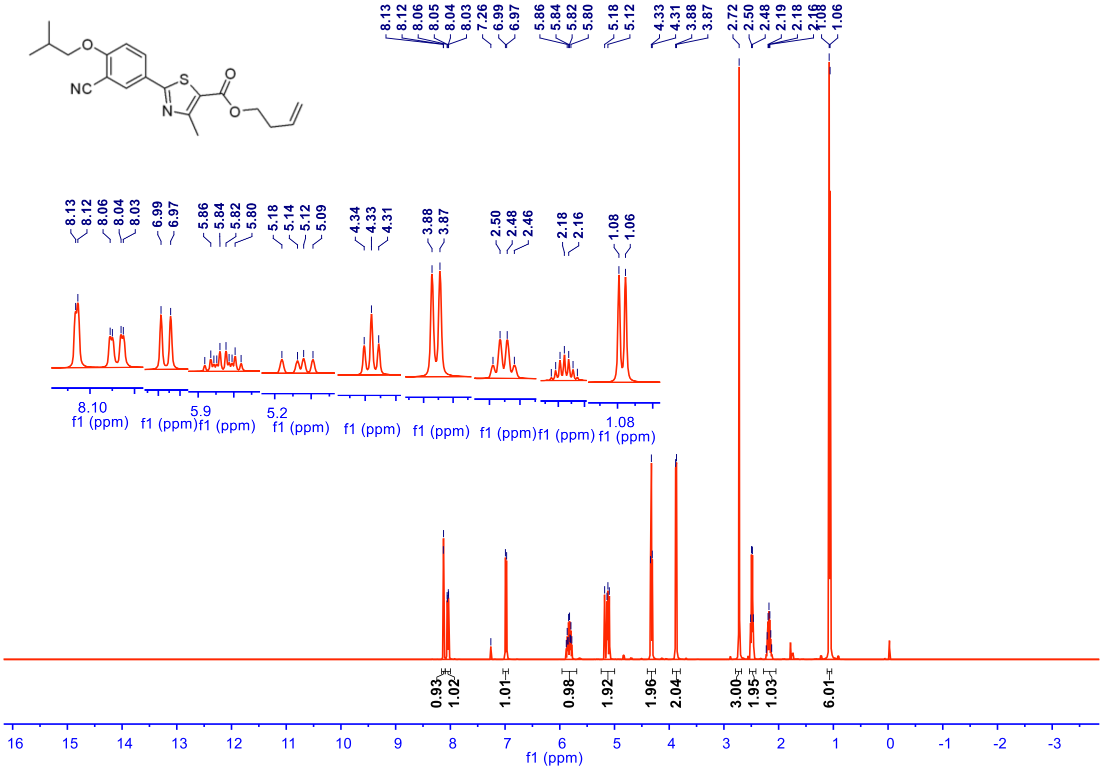

Supplementary Figure 386. ^{1}H NMR spectrum of **but-3-en-1-yl 2-(3-cyano-4-isobutoxyphenyl)-4-methylthiazole-5-carboxylate 42b** (400 MHz, $CDCl_3$)

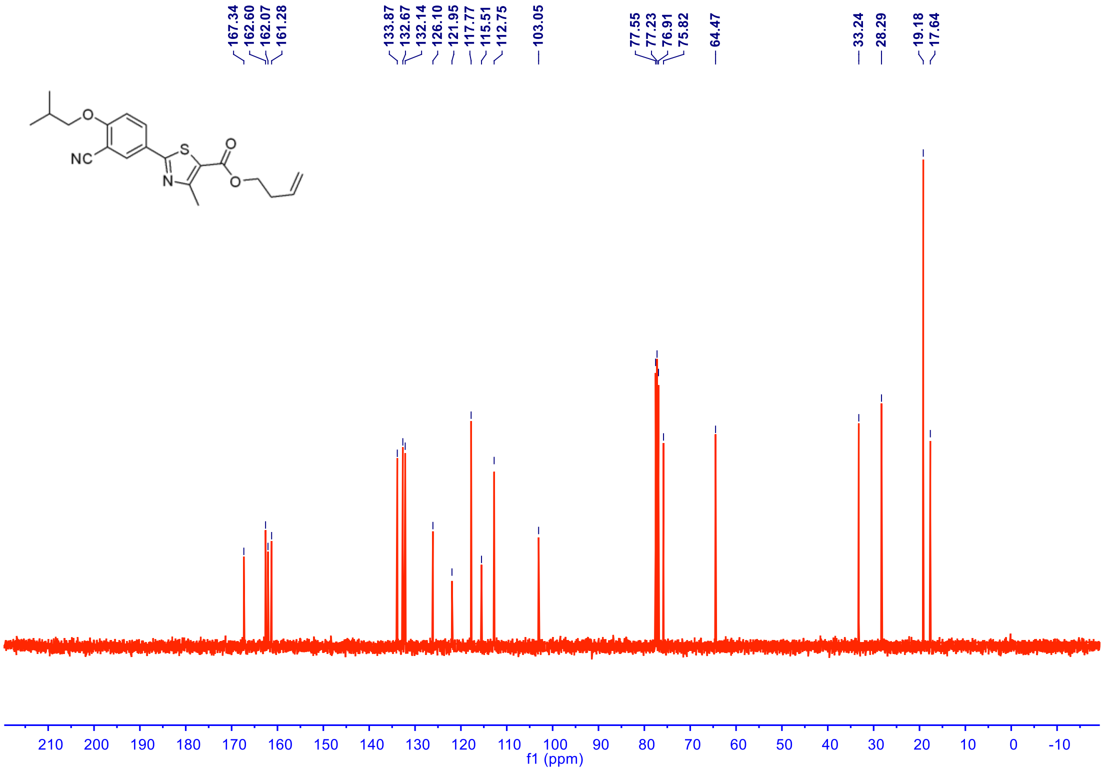

Supplementary Figure 387. ^{13}C NMR spectrum of **but-3-en-1-yl 2-(3-cyano-4-isobutoxyphenyl)-4-methylthiazole-5-carboxylate 42b** (101 MHz, $CDCl_3$)

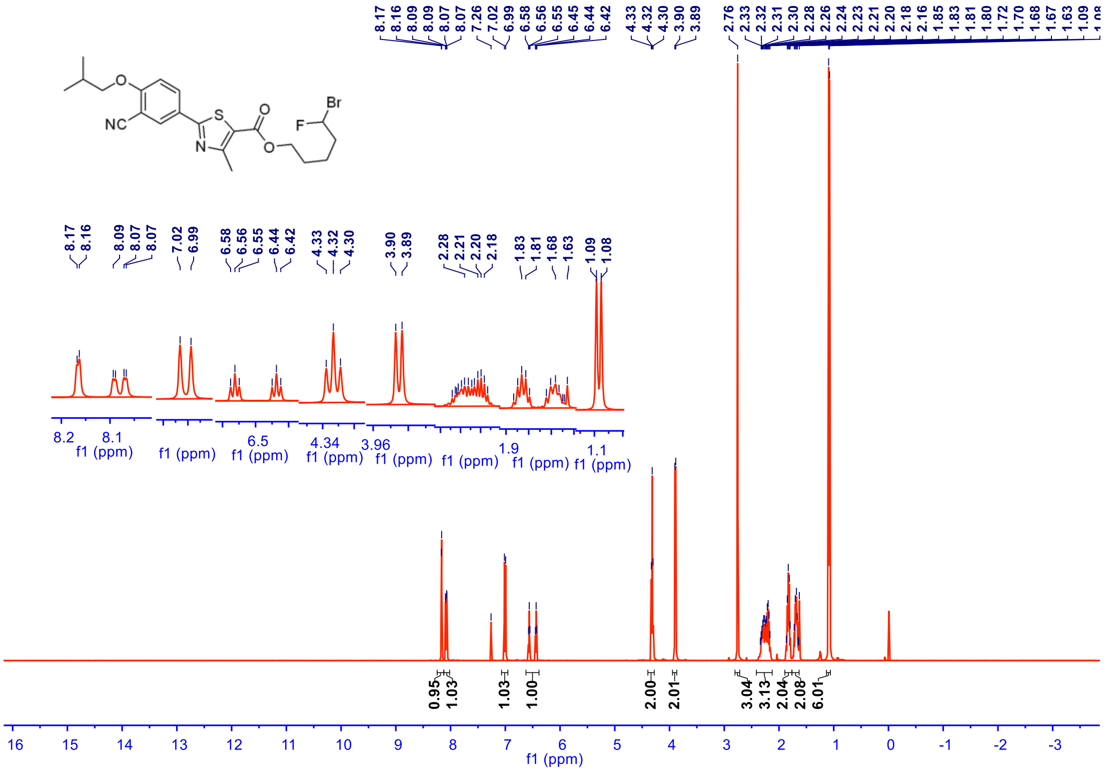

Supplementary Figure 388. ^{1}H NMR spectrum of **5-bromo-5-fluoropentyl 2-(3-cyano-4-isobutoxyphenyl)-4-methylthiazole-5-carboxylate 42a** (400 MHz, $CDCl_3$)

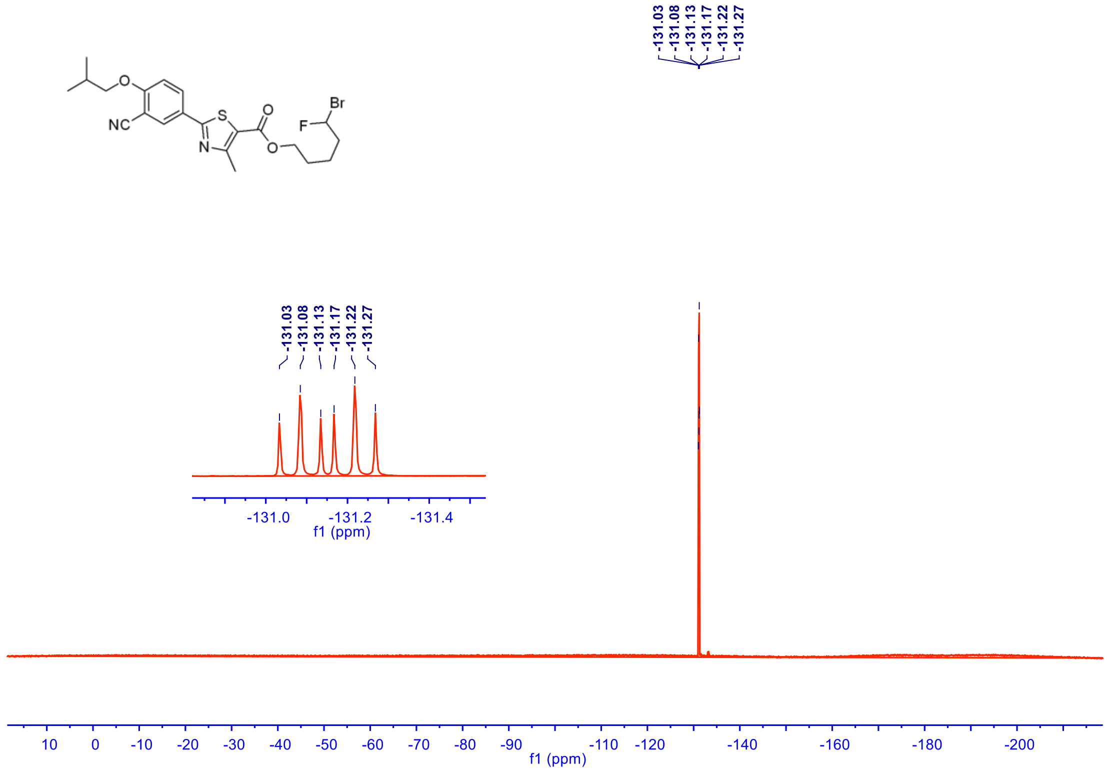

Supplementary Figure 389. ^{19}F NMR spectrum of **5-bromo-5-fluoropentyl 2-(3-cyano-4-isobutoxyphenyl)-4-methylthiazole-5-carboxylate 42a** (376 MHz, $CDCl_3$)

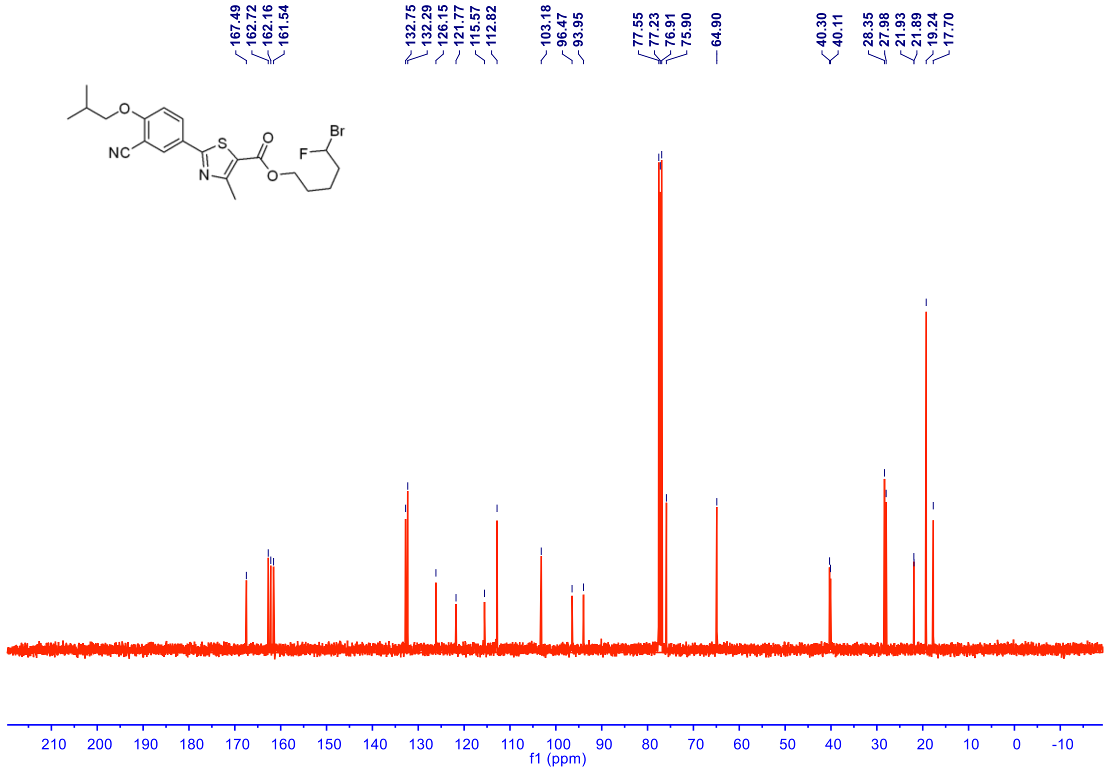

Supplementary Figure 390. ^{13}C NMR spectrum of **but-5-bromo-5-fluoropentyl 2-(3-cyano-4-isobutoxyphenyl)-4-methylthiazole-5-carboxylate 42a** (101 MHz, $CDCl_3$)

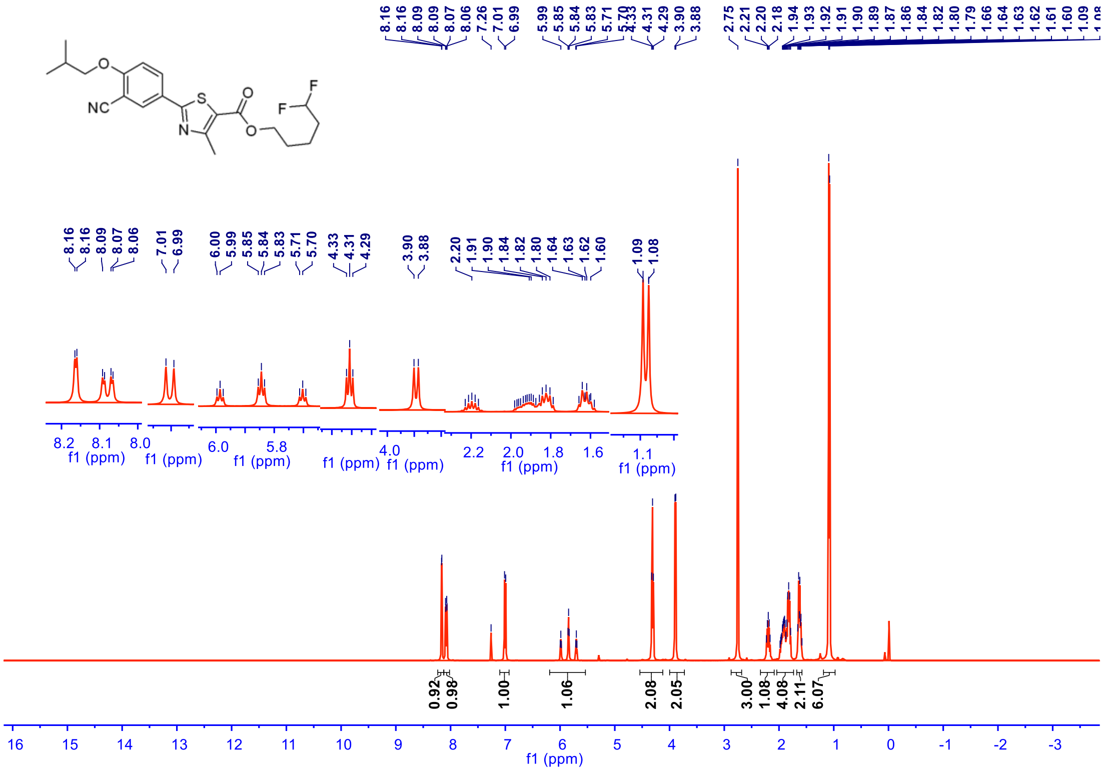

Supplementary Figure 391. ^{1}H NMR spectrum of compound **42** (400 MHz, $CDCl_3$)

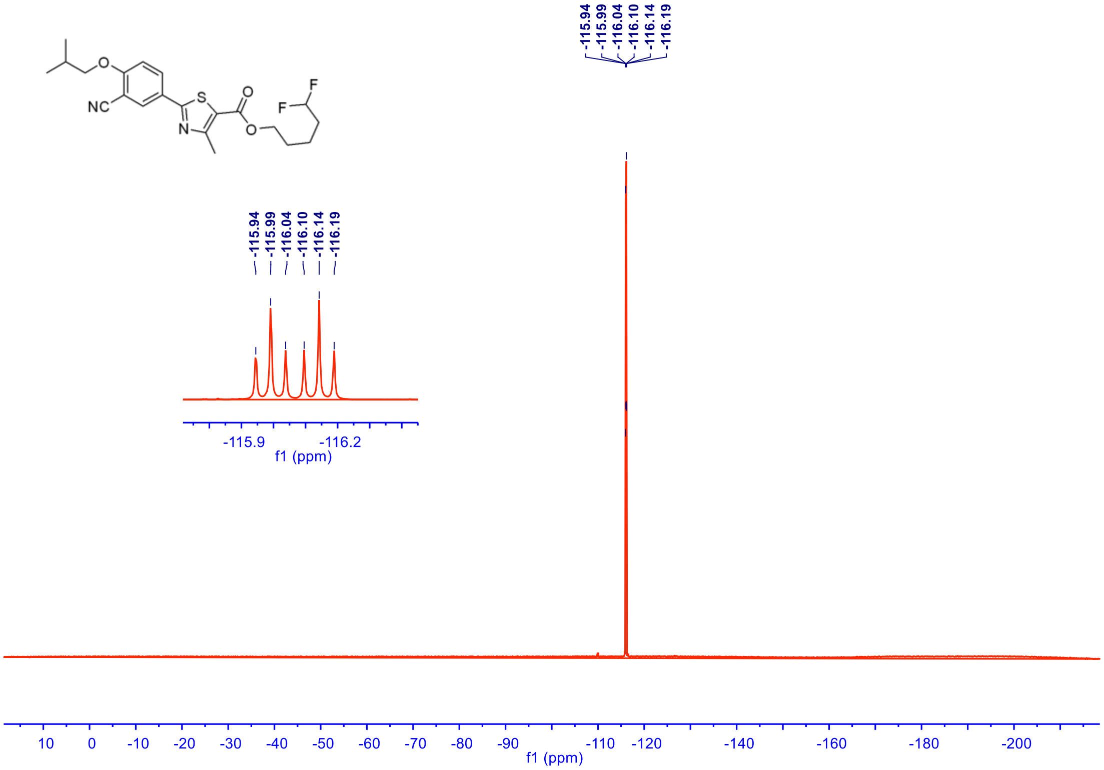

Supplementary Figure 392. ^{19}F NMR spectrum of compound **42** (376 MHz, $CDCl_3$)

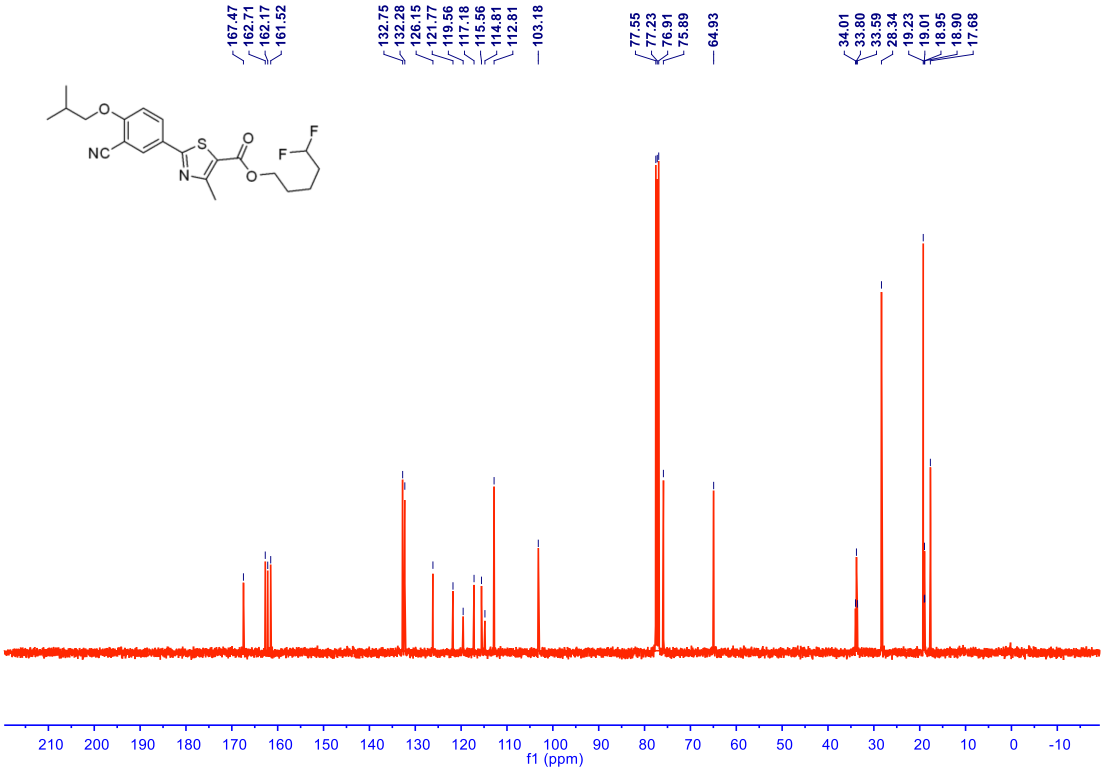

Supplementary Figure 393. ^{13}C NMR spectrum of compound **42** (101 MHz, $CDCl_3$)

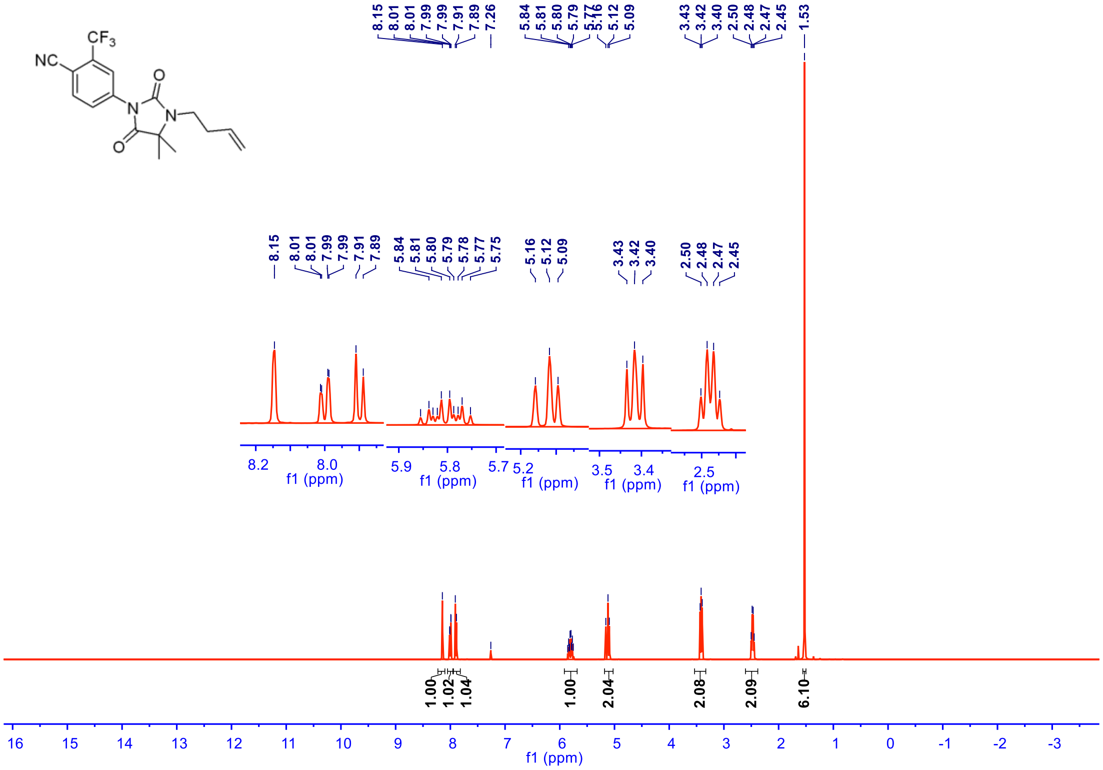

Supplementary Figure 394. ^{1}H NMR spectrum of **4-(3-(but-3-en-1-yl)-4,4-dimethyl-2,5-dioxoimidazolidin-1-yl)-2-(trifluoromethyl)benzonitrile 43b** (400 MHz, $CDCl_3$)

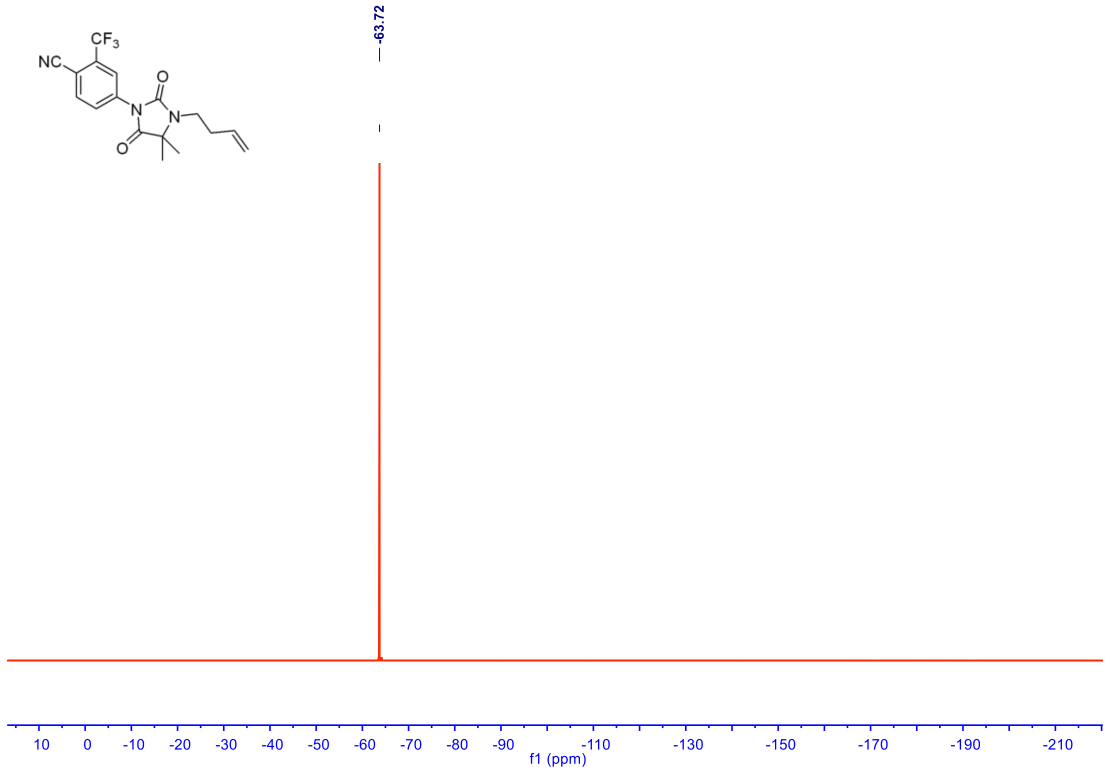

Supplementary Figure 395. ^{19}F NMR spectrum of **4-(3-(but-3-en-1-yl)-4,4-dimethyl-2,5-dioxoimidazolidin-1-yl)-2-(trifluoromethyl)benzonitrile 43b** (376 MHz, $CDCl_3$)

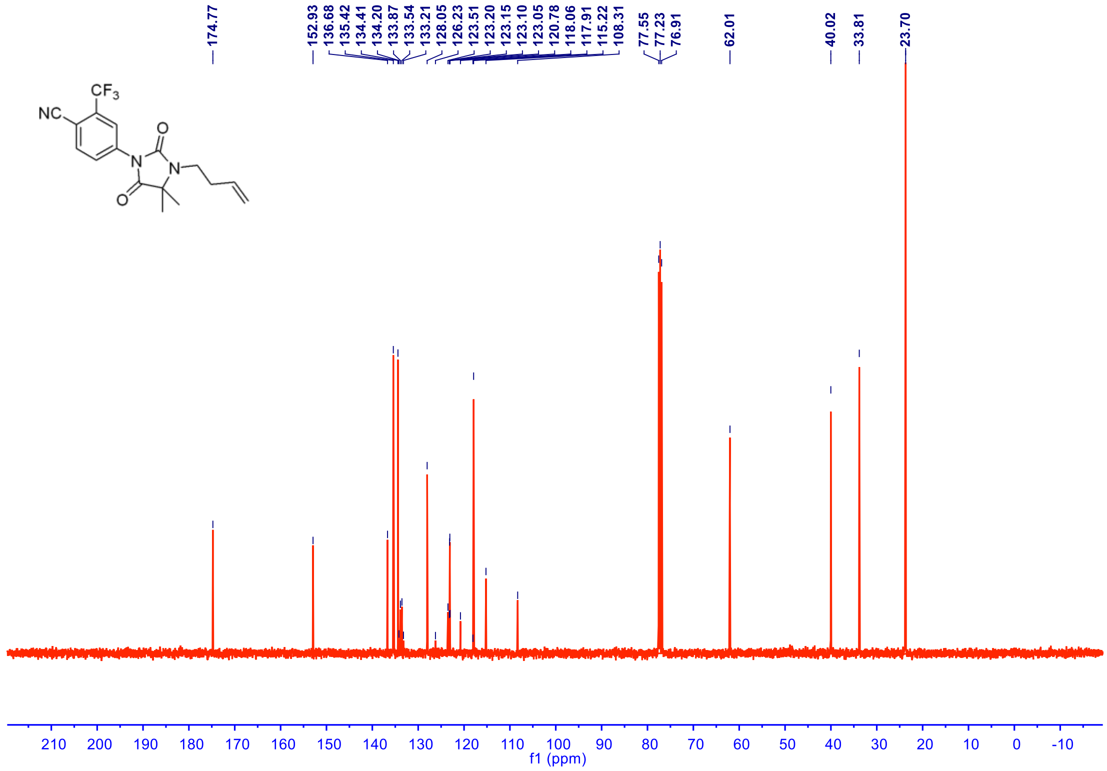

Supplementary Figure 396. ^{13}C NMR spectrum of **4-(3-(but-3-en-1-yl)-4,4-dimethyl-2,5-dioxoimidazolidin-1-yl)-2-(trifluoromethyl)benzonitrile 43b** (101 MHz, $CDCl_3$)

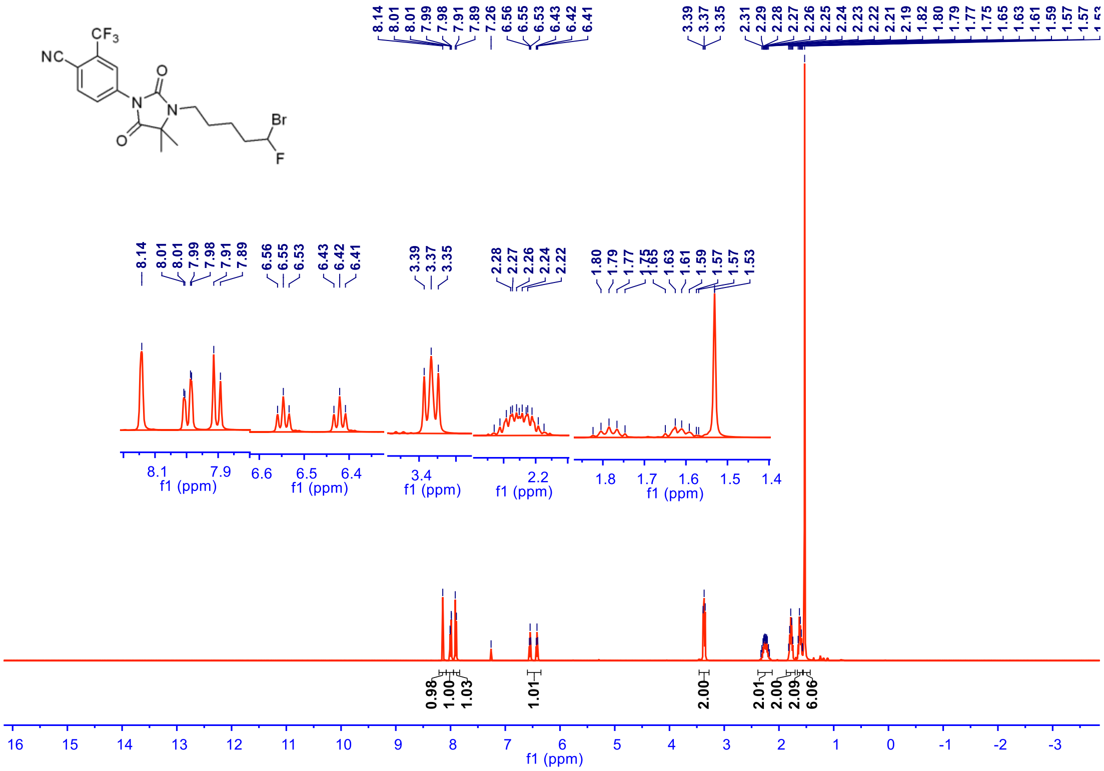

Supplementary Figure 397. ^{1}H NMR spectrum of **4-(3-(5-bromo-5-fluoropentyl)-4,4-dimethyl-2,5-dioxoimidazolidin-1-yl)-2-(trifluoromethyl)benzonitrile 43a** (400 MHz, $CDCl_3$)

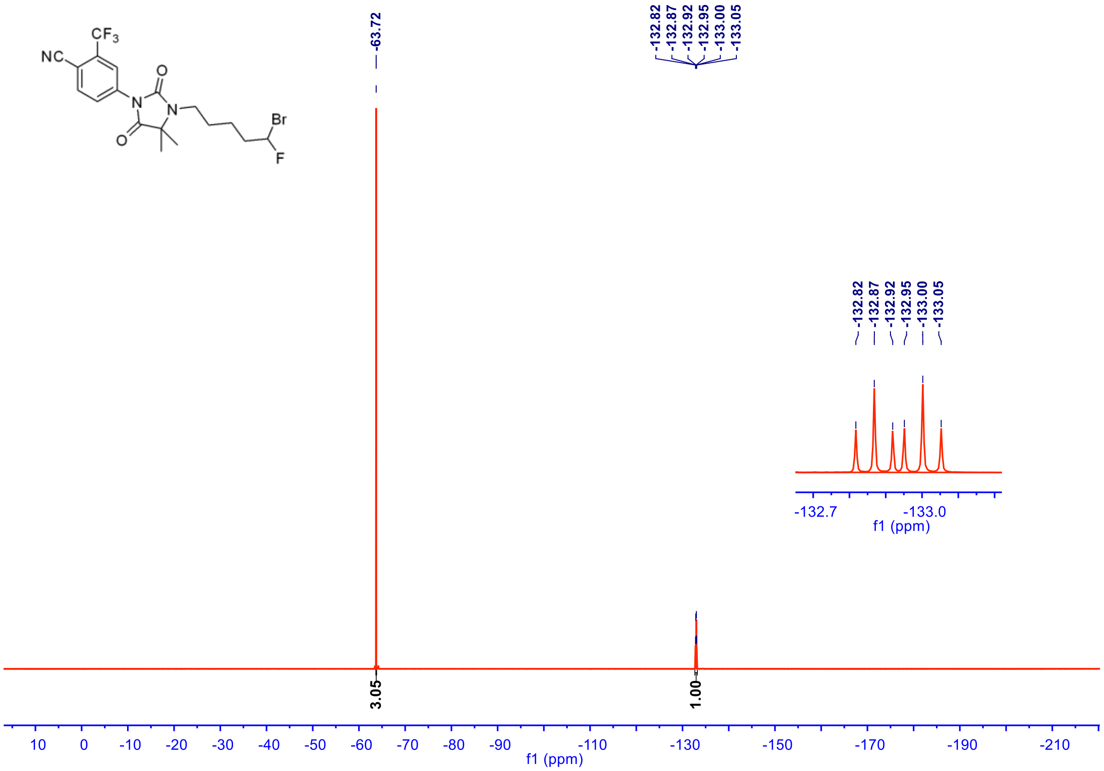

Supplementary Figure 398. ^{19}F NMR spectrum of **4-(3-(5-bromo-5-fluoropentyl)-4,4-dimethyl-2,5-dioxoimidazolidin-1-yl)-2-(trifluoromethyl)benzonitrile 43a** (376 MHz, $CDCl_3$)

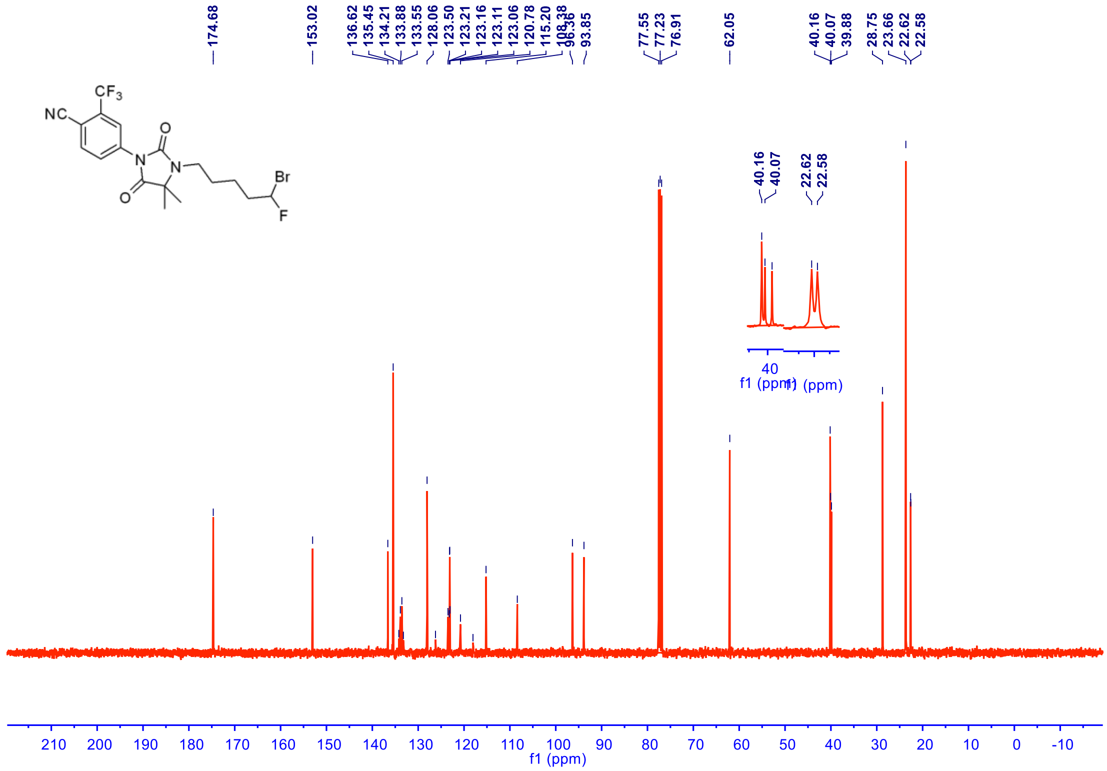

Supplementary Figure 399. ^{13}C NMR spectrum of **4-(3-(5-bromo-5-fluoropentyl)-4,4-dimethyl-2,5-dioxoimidazolidin-1-yl)-2-(trifluoromethyl)benzonitrile 43a** (101 MHz, $CDCl_3$)

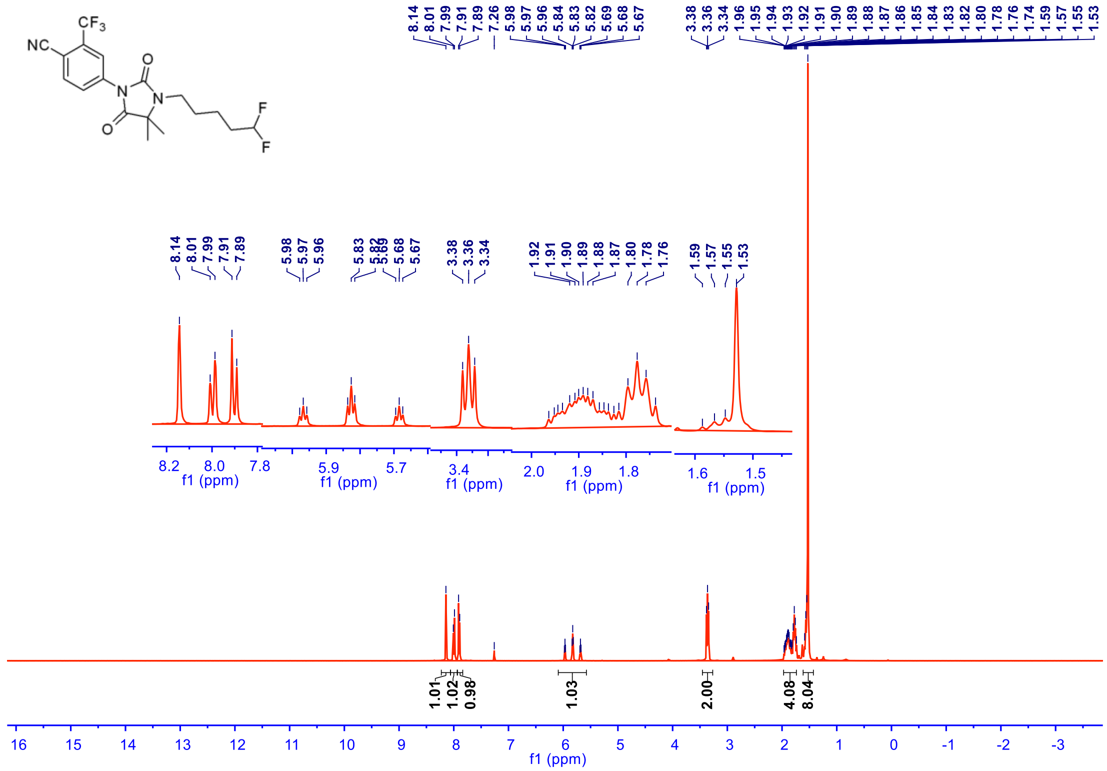

Supplementary Figure 400. ^{1}H NMR spectrum of compound **43** (400 MHz, $CDCl_3$)

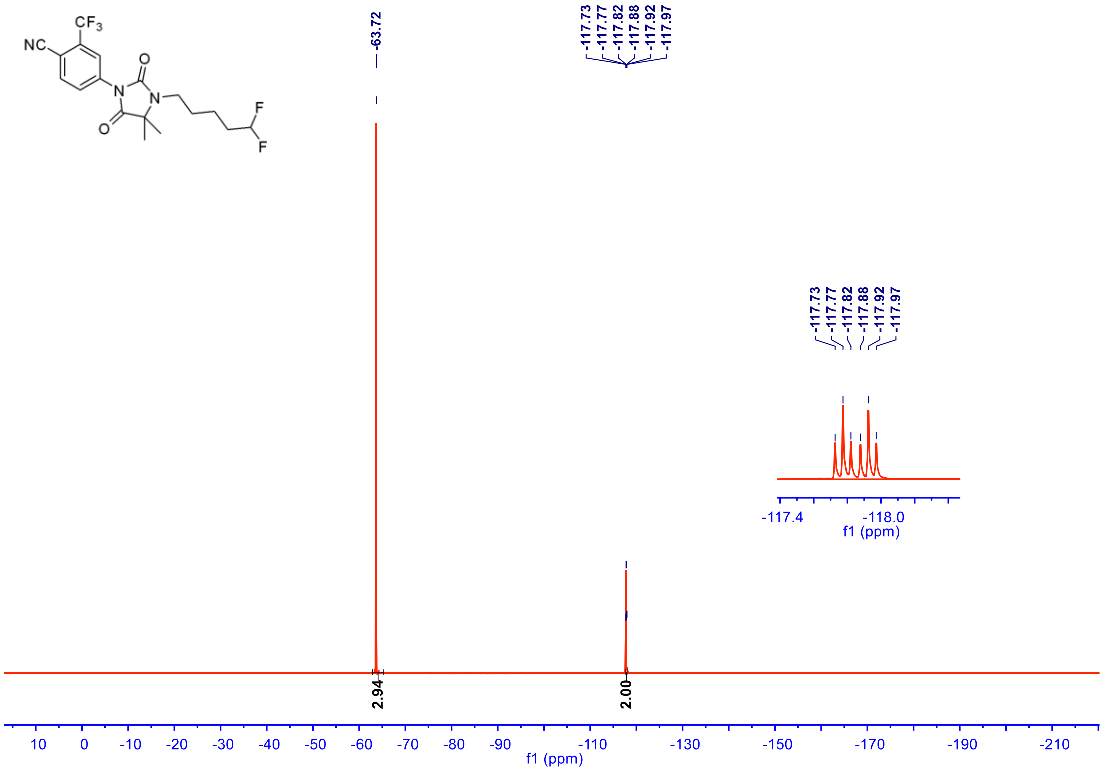

Supplementary Figure 401. ^{19}F NMR spectrum of compound **43** (376 MHz, $CDCl_3$)

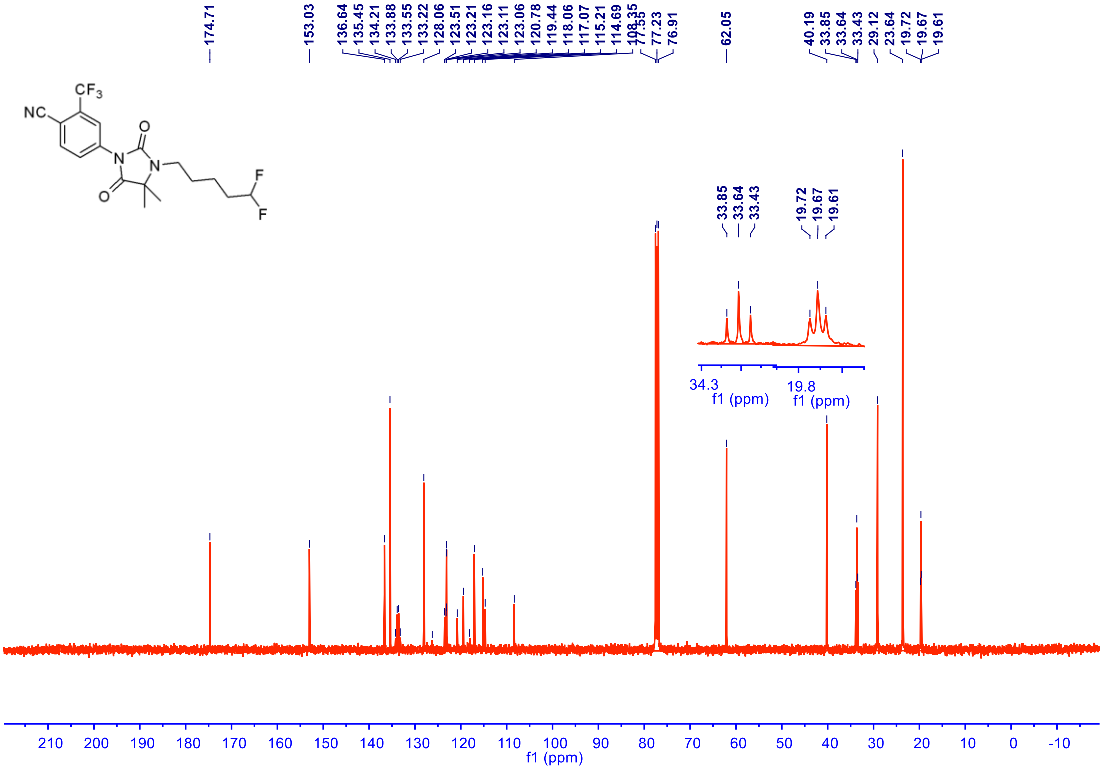

Supplementary Figure 402. ^{13}C NMR spectrum of compound **43** (101 MHz, $CDCl_3$)

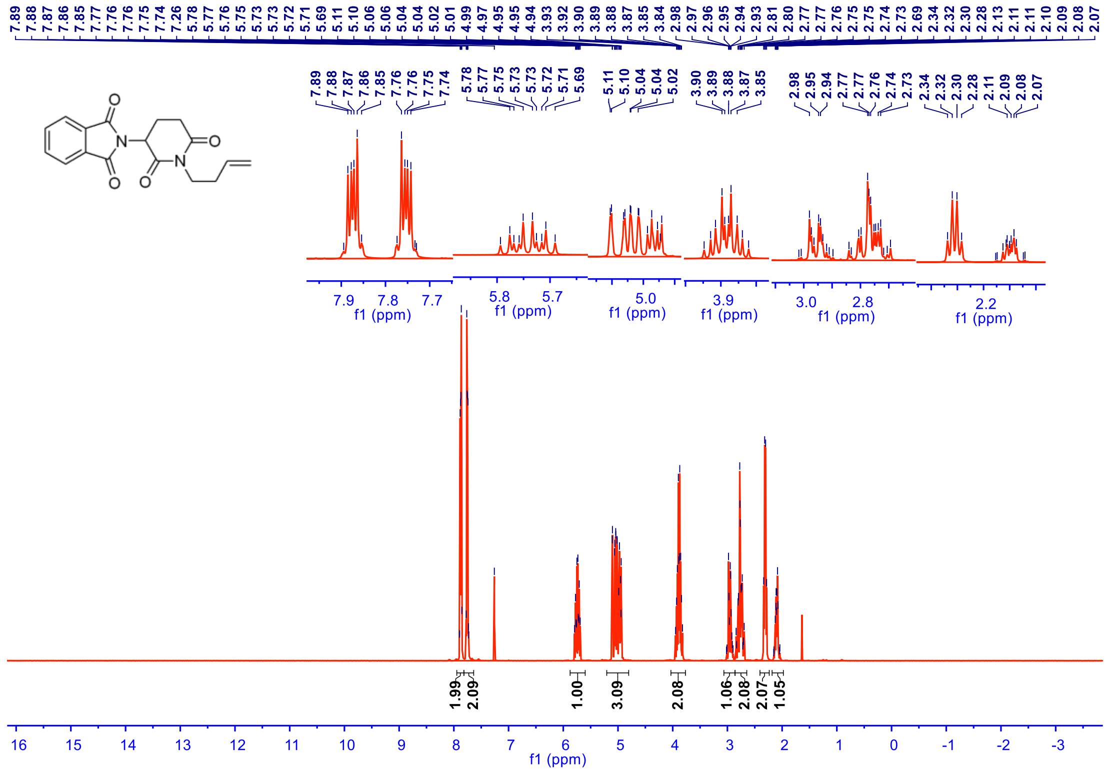

Supplementary Figure 403. ^{1}H NMR spectrum of **2-(1-(but-3-en-1-yl)-2,6-dioxopiperidin-3-yl)isoindoline-1,3-dione 44b** (400 MHz, $CDCl_3$)

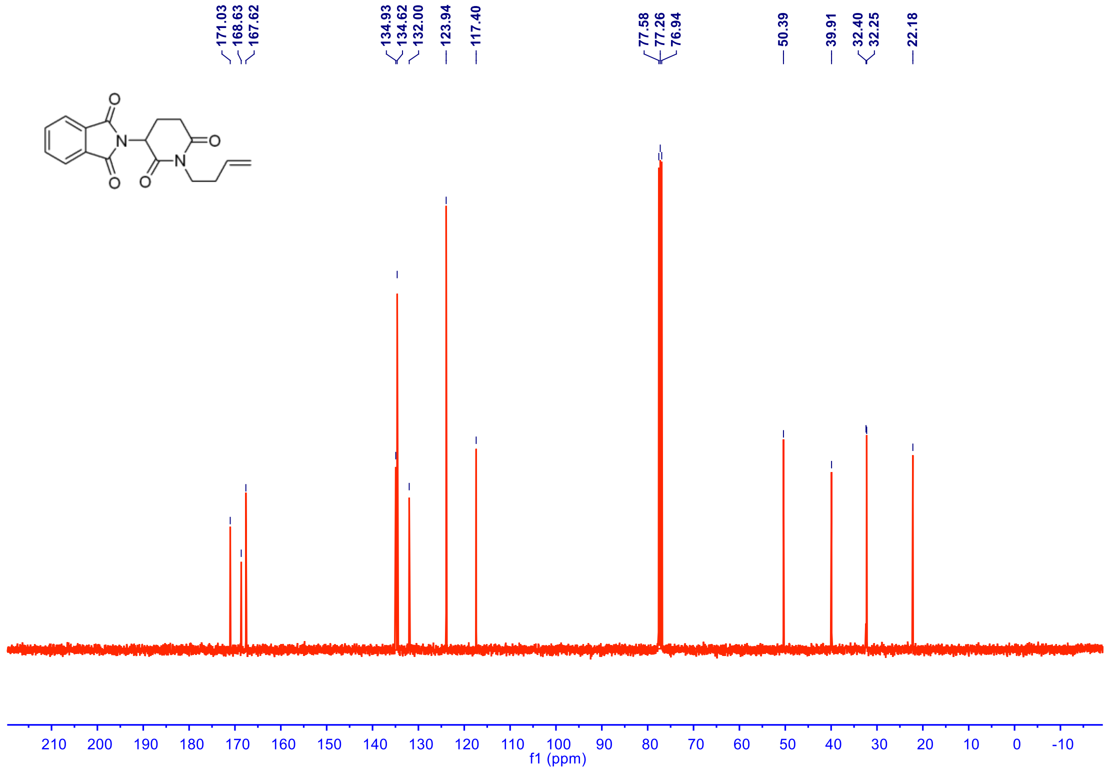

Supplementary Figure 404. ^{13}C NMR spectrum of **2-(1-(but-3-en-1-yl)-2,6-dioxopiperidin-3-yl)isoindoline-1,3-dione 44b** (101 MHz, $CDCl_3$)

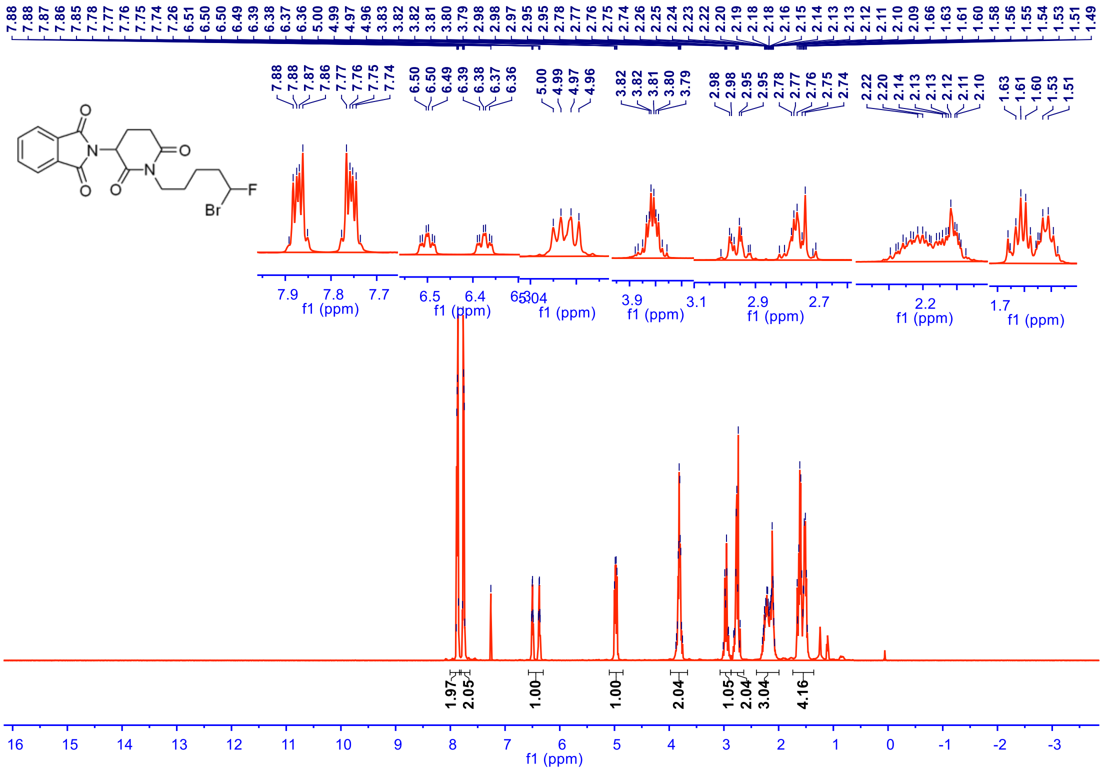

Supplementary Figure 405. ^{1}H NMR spectrum of **2-(1-(5-bromo-5-fluoropentyl)-2,6-dioxopiperidin-3-yl)isoindoline-1,3-dione 44a** (400 MHz, $CDCl_3$)

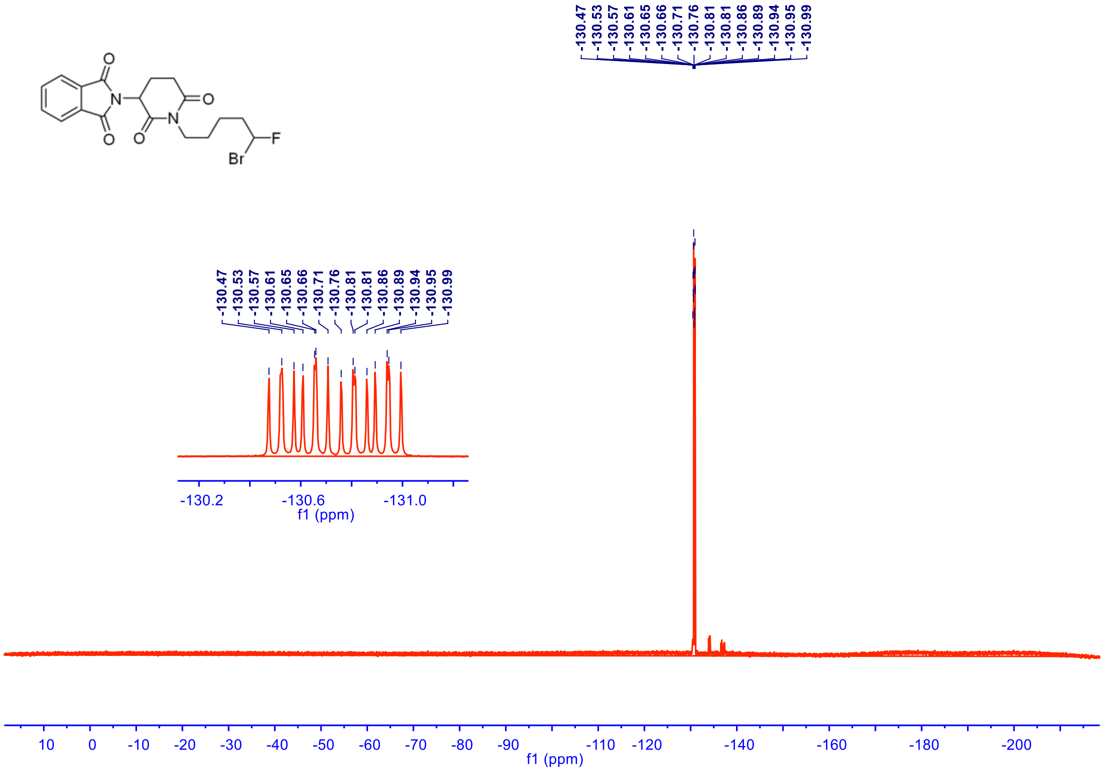

Supplementary Figure 406. ^{19}F NMR spectrum of **2-(1-(5-bromo-5-fluoropentyl)-2,6-dioxopiperidin-3-yl)isoindoline-1,3-dione 44a** (376 MHz, $CDCl_3$)

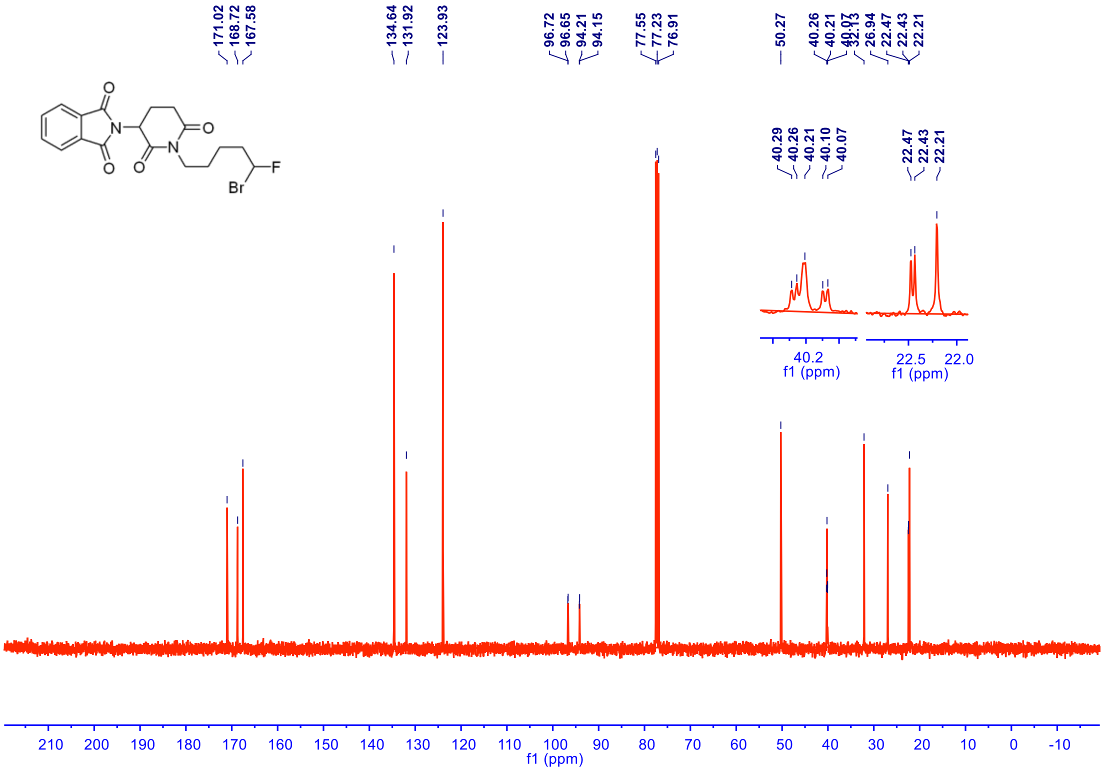

Supplementary Figure 407. ^{13}C NMR spectrum of **2-(1-(5-bromo-5-fluoropentyl)-2,6-dioxopiperidin-3-yl)isoindoline-1,3-dione 44a** (101 MHz, $CDCl_3$)

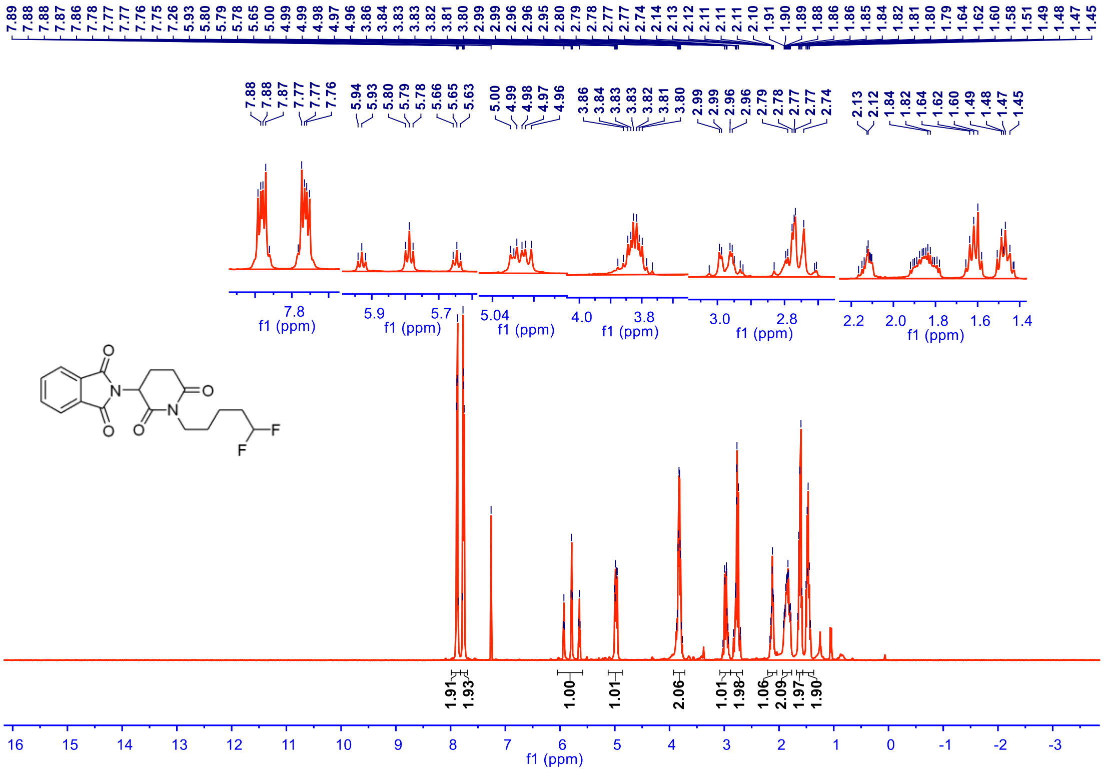

Supplementary Figure 408. ^{1}H NMR spectrum of compound **44** (400 MHz, $CDCl_3$)

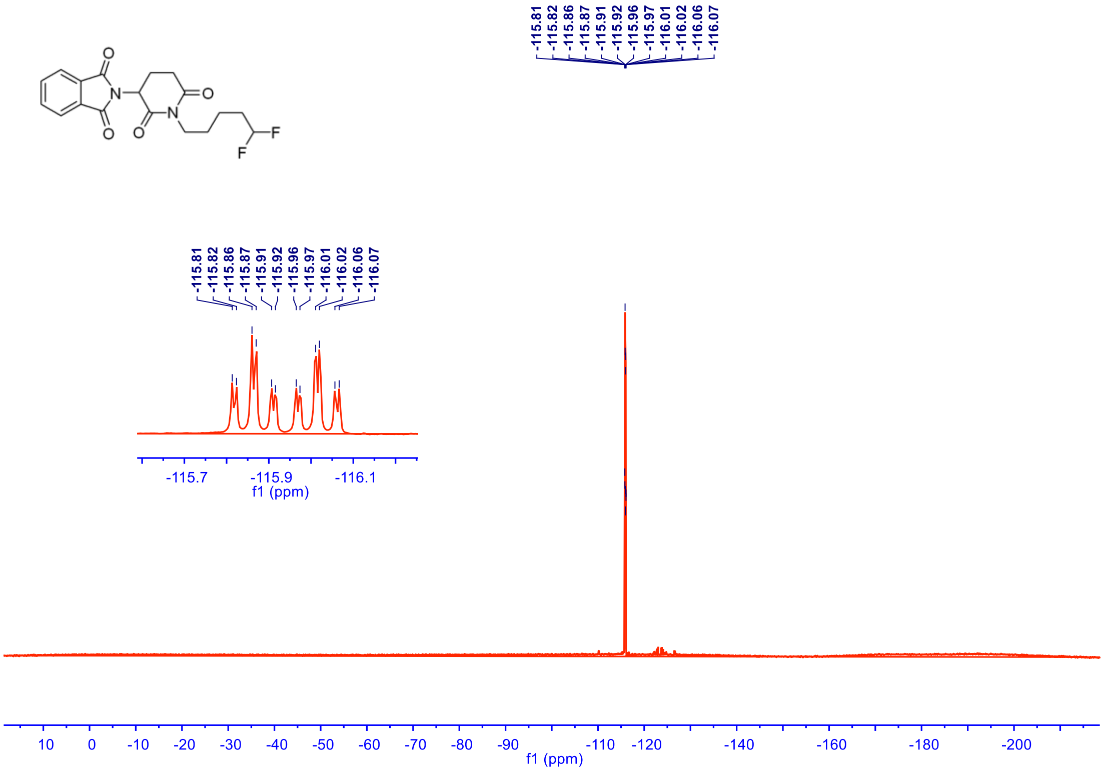

Supplementary Figure 409. ^{19}F NMR spectrum of compound **44** (376 MHz, $CDCl_3$)

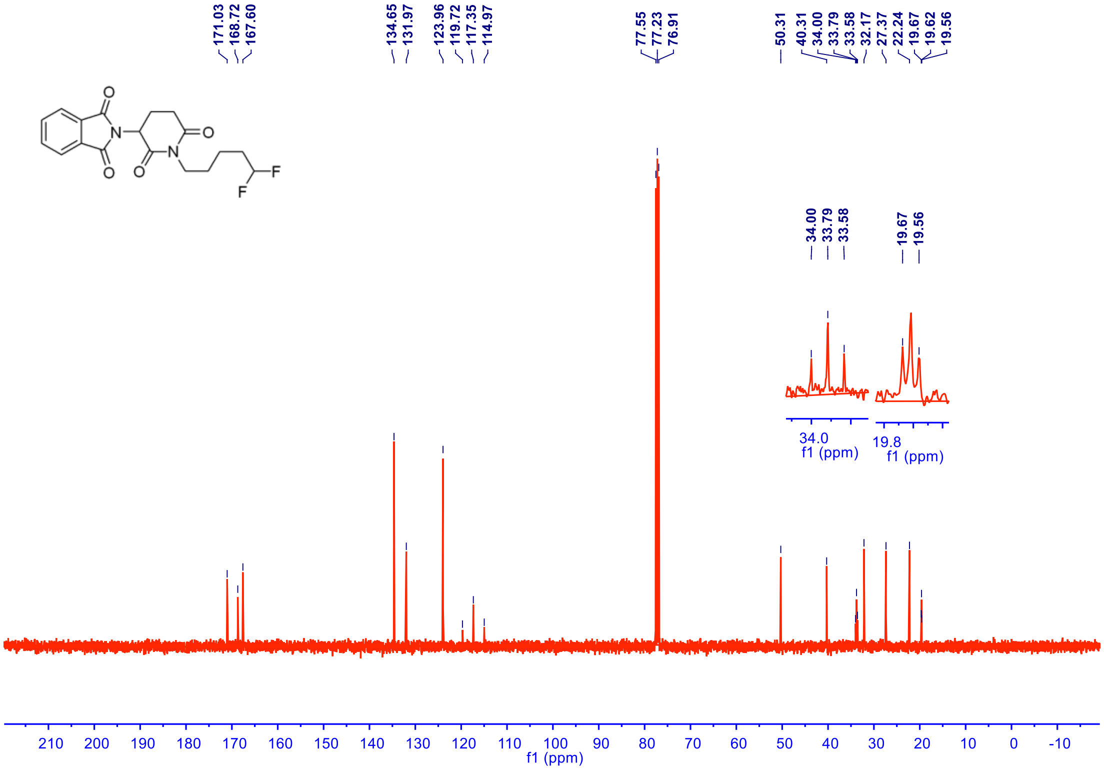

Supplementary Figure 410. ^{13}C NMR spectrum of compound **44** (101 MHz, $CDCl_3$)

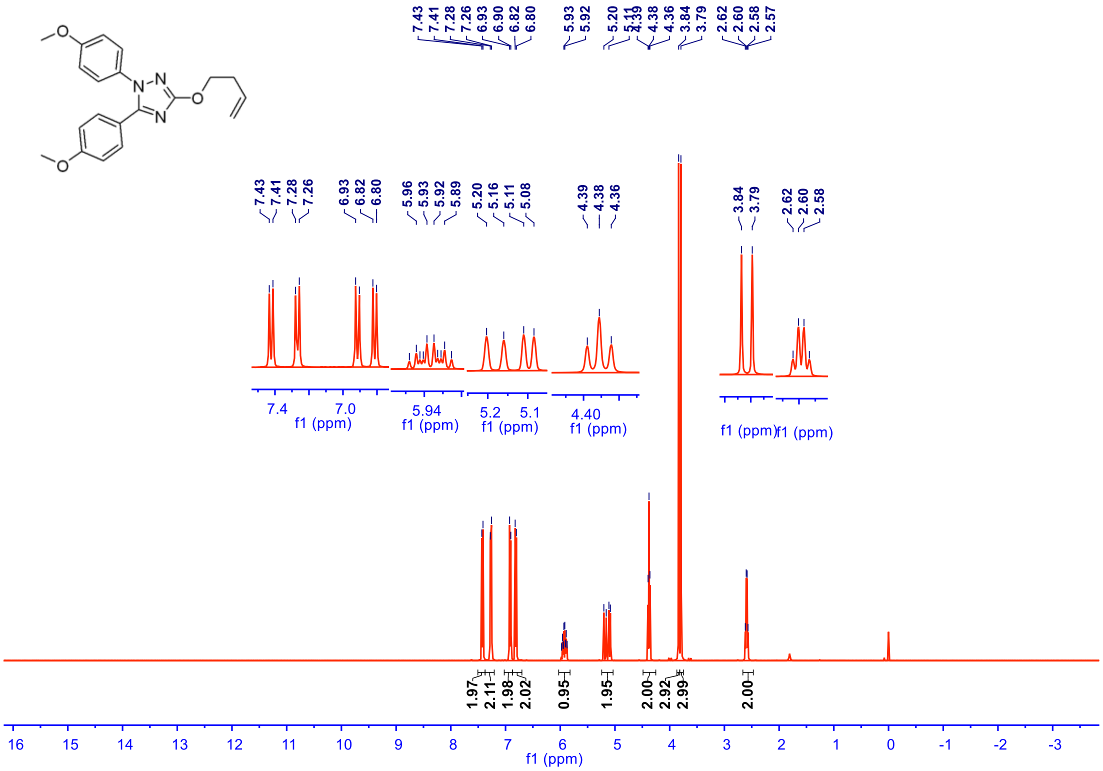

Supplementary Figure 411. ^{1}H NMR spectrum of **3-(but-3-en-1-yloxy)-1,5-bis(4-methoxyphenyl)-*1H*-1,2,4-triazole 45b** (400 MHz, $CDCl_3$)

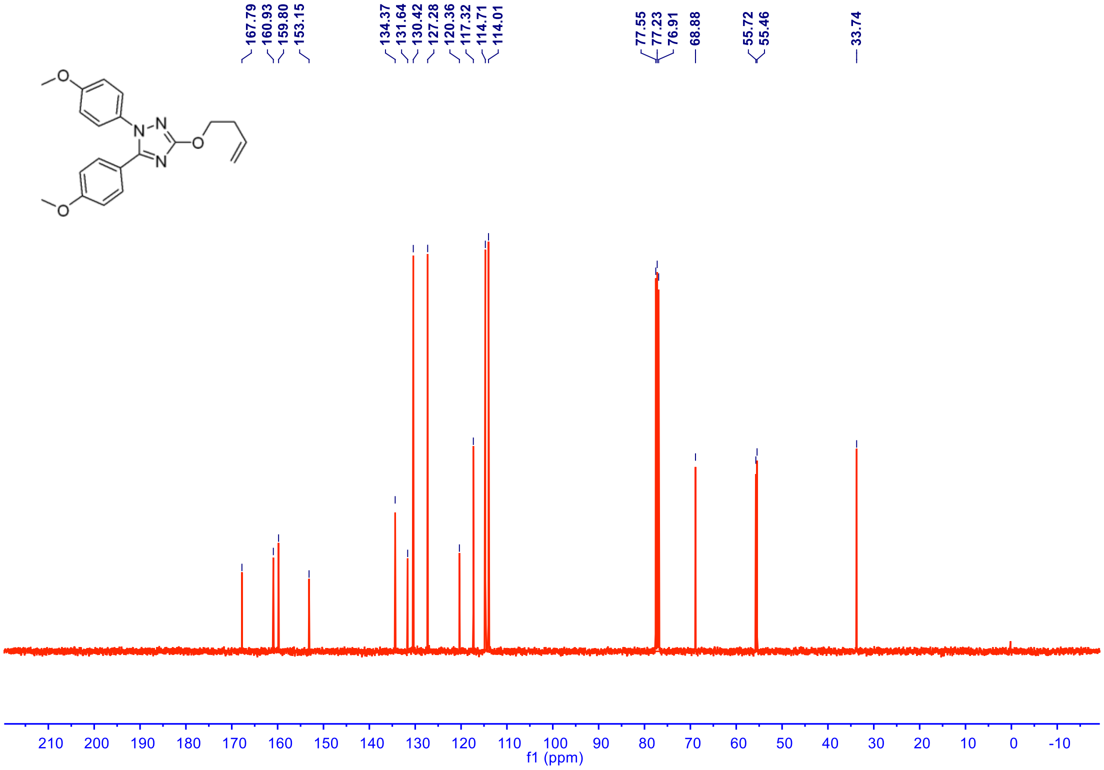

Supplementary Figure 412. ^{13}C NMR spectrum of **3-(but-3-en-1-yloxy)-1,5-bis(4-methoxyphenyl)-*1H*-1,2,4-triazole 45b** (101 MHz, $CDCl_3$)

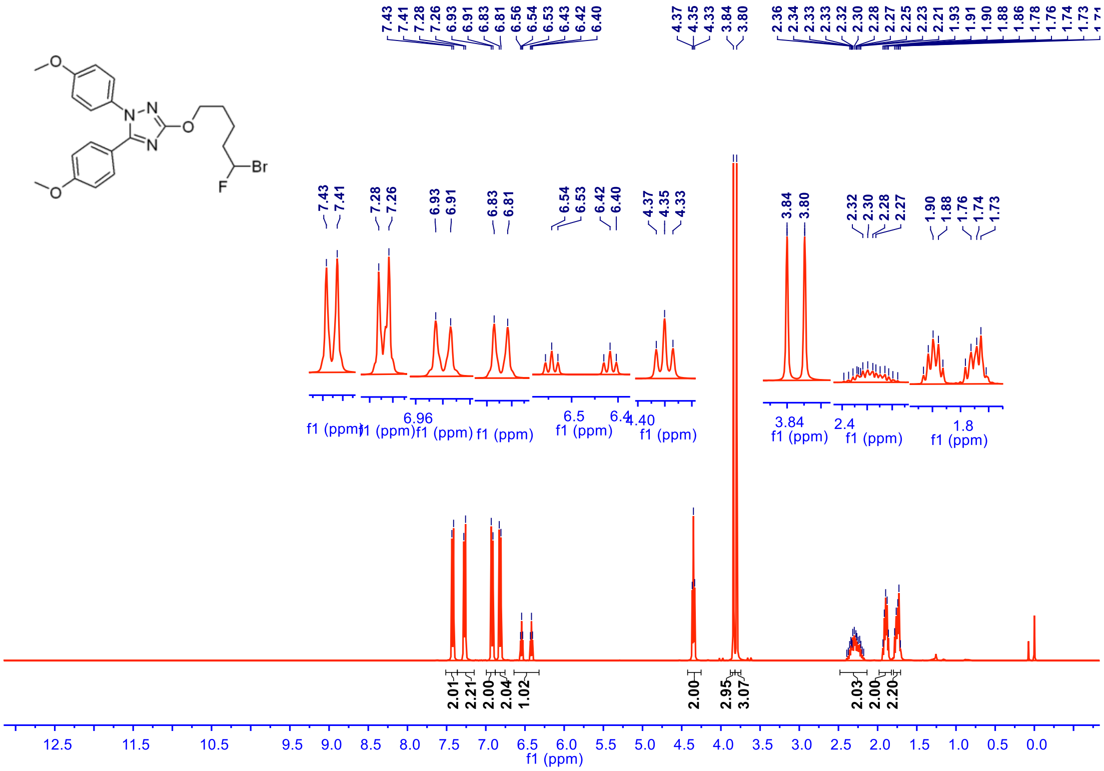

Supplementary Figure 413. ^{1}H NMR spectrum of **3-((5-bromo-5-fluoropentyl)oxy)-1,5-bis(4-methoxyphenyl)-1*H*-1,2,4-triazole 45a** (400 MHz, $CDCl_3$)

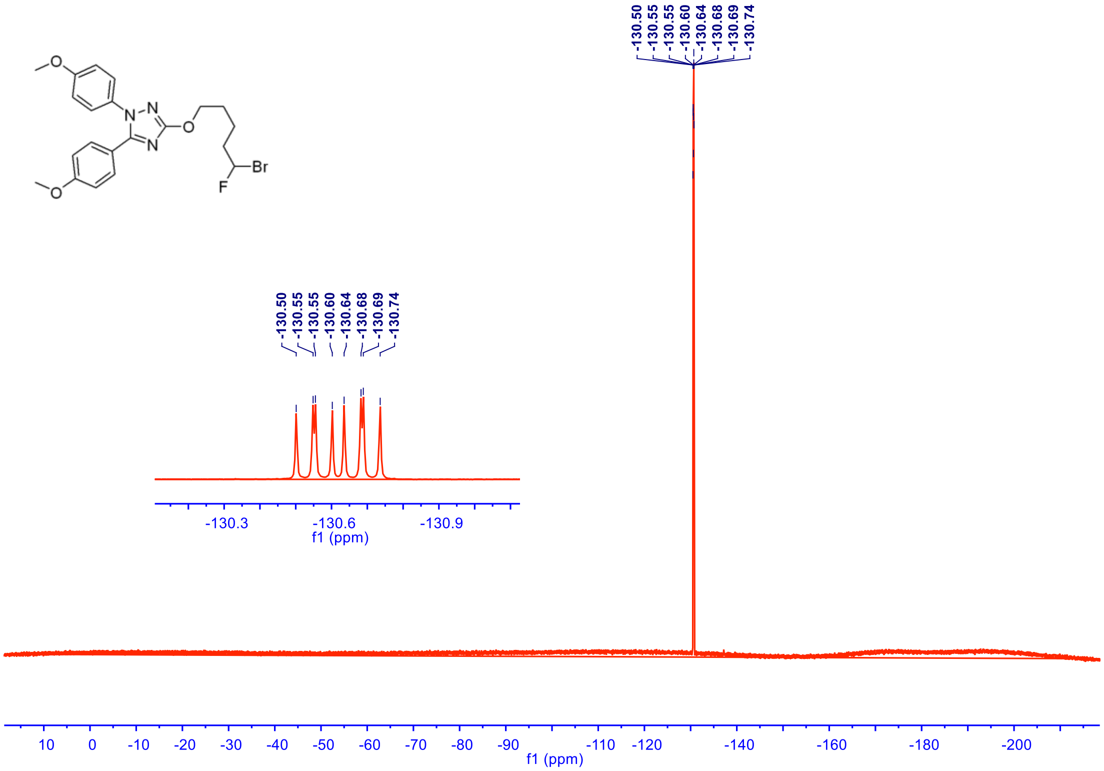

Supplementary Figure 414. ^{19}F NMR spectrum of **3-((5-bromo-5-fluoropentyl)oxy)-1,5-bis(4-methoxyphenyl)-1*H*-1,2,4-triazole 45a** (376 MHz, $CDCl_3$)

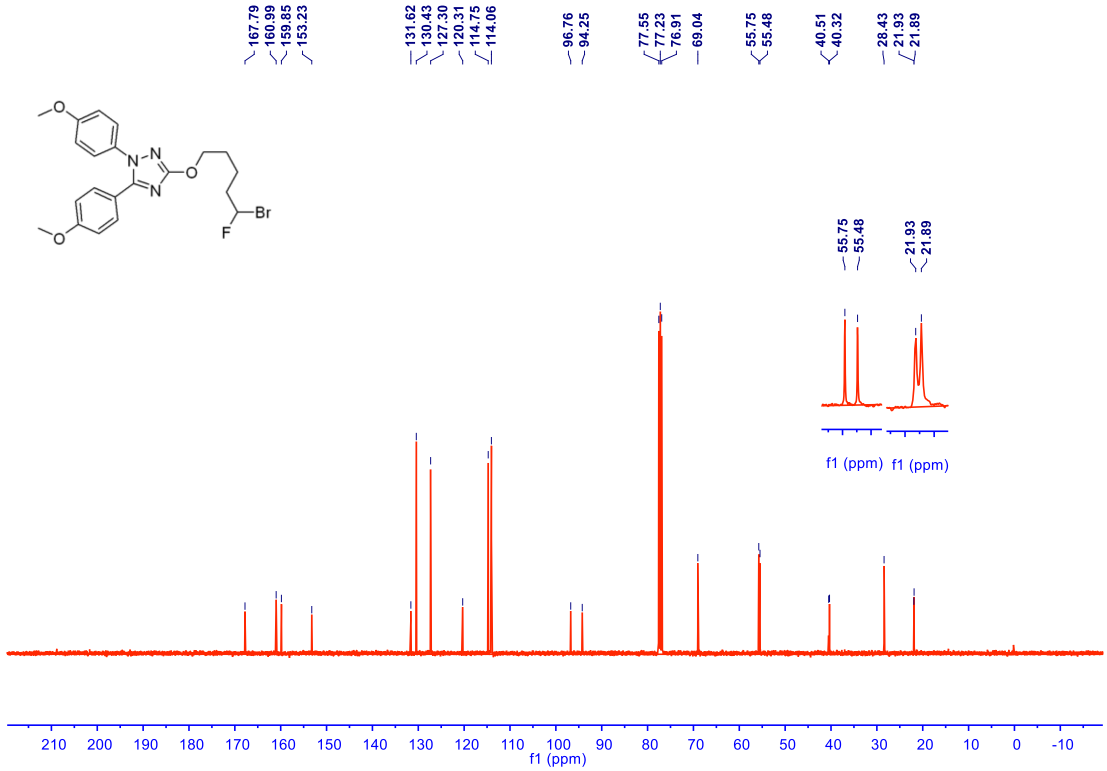

Supplementary Figure 415. ^{13}C NMR spectrum of **3-((5-bromo-5-fluoropentyl)oxy)-1,5-bis(4-methoxyphenyl)-1*H*-1,2,4-triazole 45a** (101 MHz, $CDCl_3$)

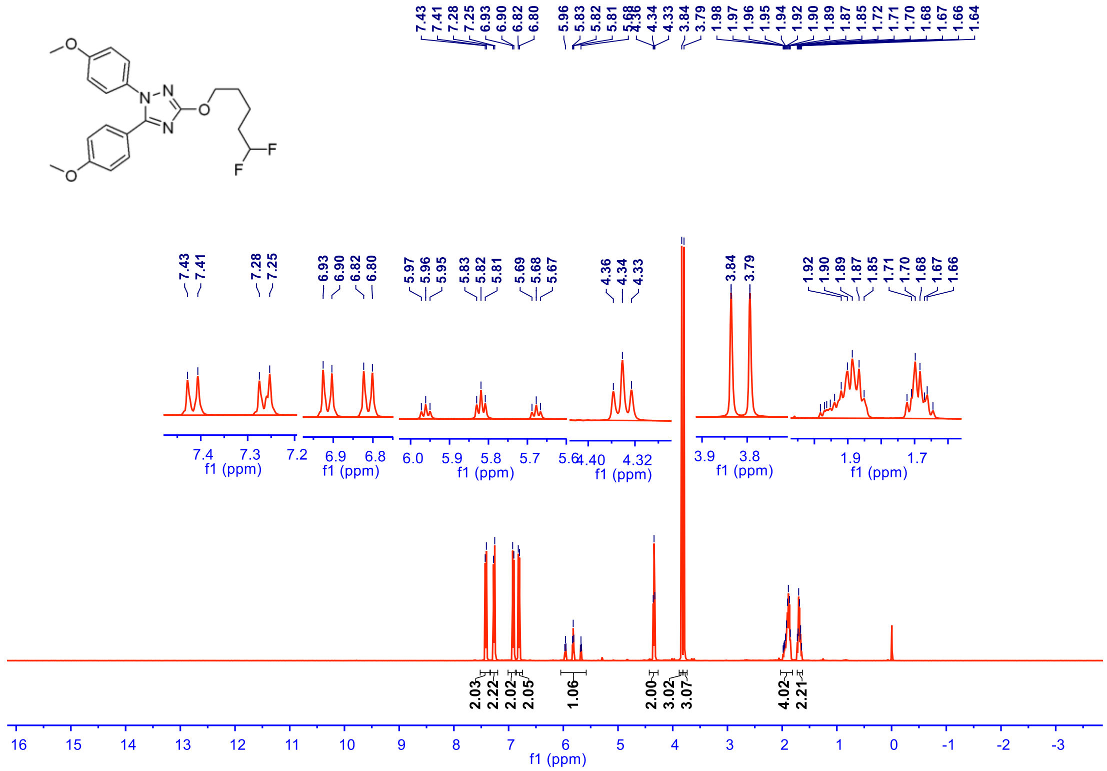

Supplementary Figure 416. ^{1}H NMR spectrum of compound **45** (400 MHz, $CDCl_3$)

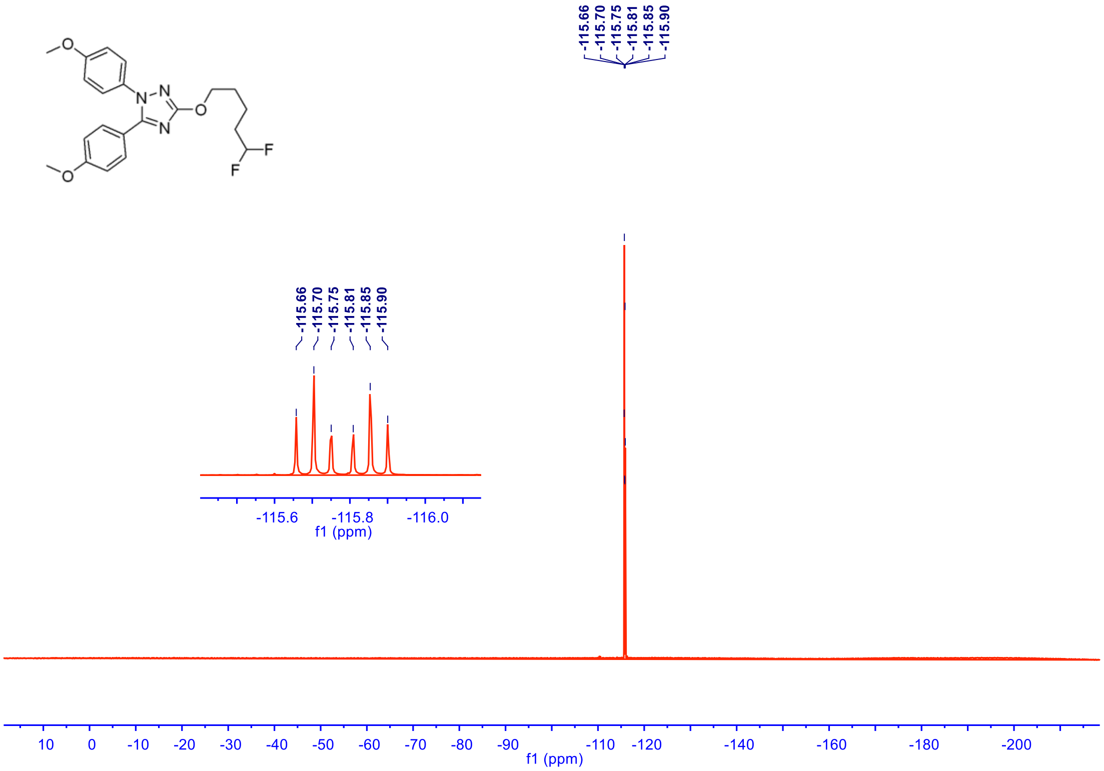

Supplementary Figure 417. ^{19}F NMR spectrum of compound **45** (376 MHz, $CDCl_3$)

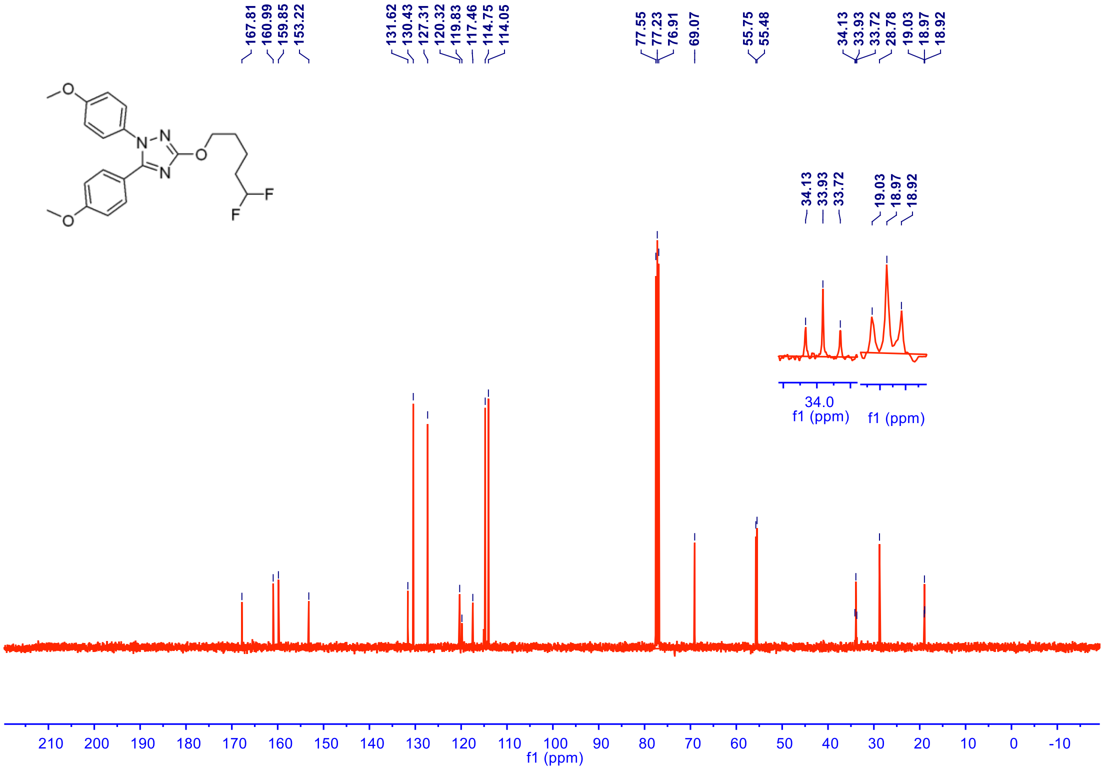

Supplementary Figure 418. ^{13}C NMR spectrum of compound **45** (101 MHz, $CDCl_3$)

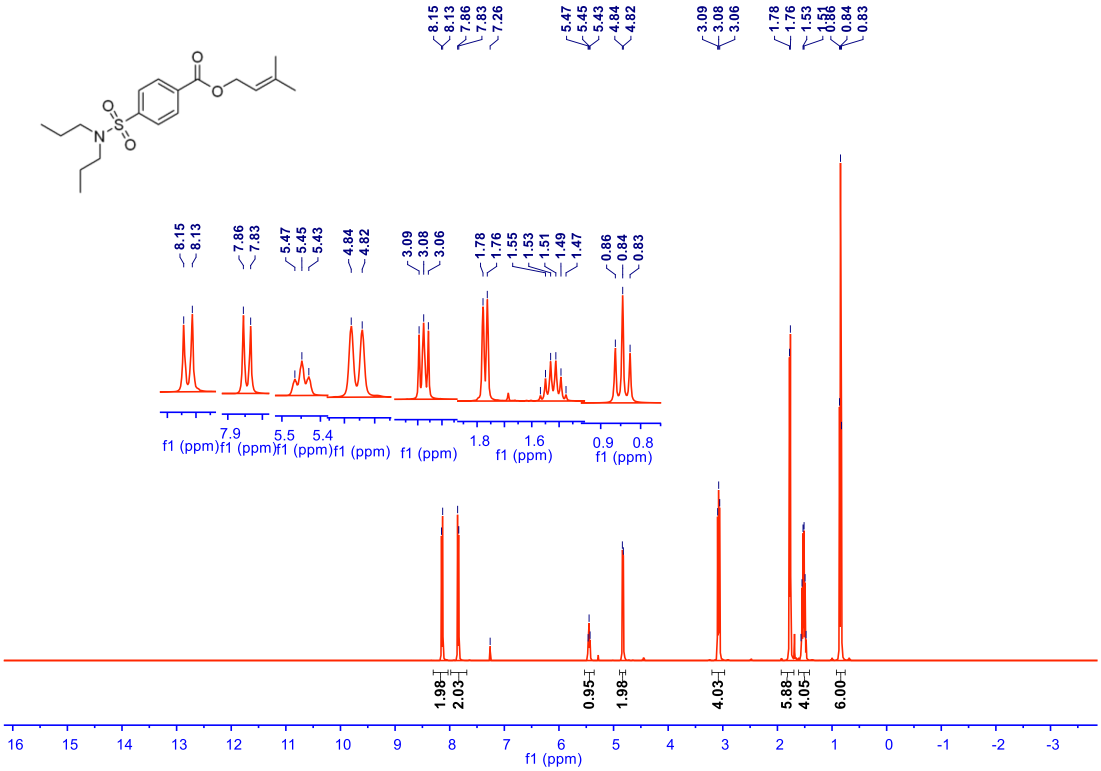

Supplementary Figure 419. ^{1}H NMR spectrum of **3-methylbut-2-en-1-yl 4-(*N*,*N*-dipropylsulfamoyl)benzoate 46b** (400 MHz, $CDCl_3$)

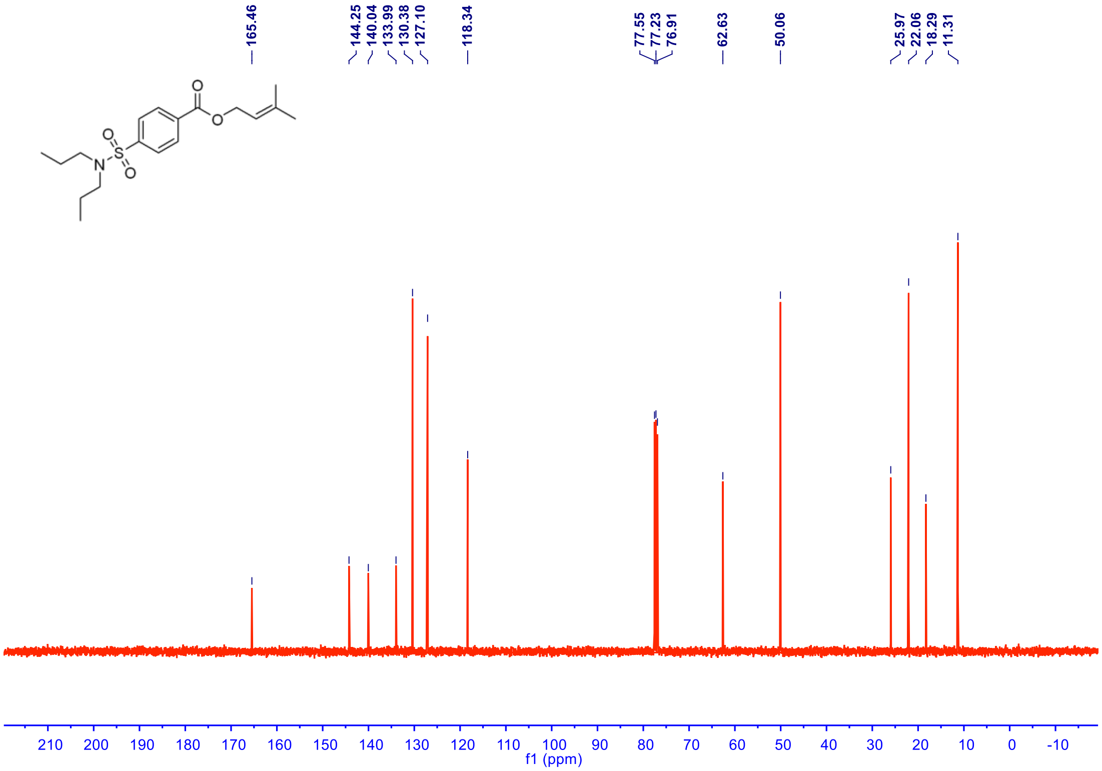

Supplementary Figure 420. ^{13}C NMR spectrum of **3-methylbut-2-en-1-yl 4-(*N*,*N*-dipropylsulfamoyl)benzoate 46b** (101 MHz, $CDCl_3$)

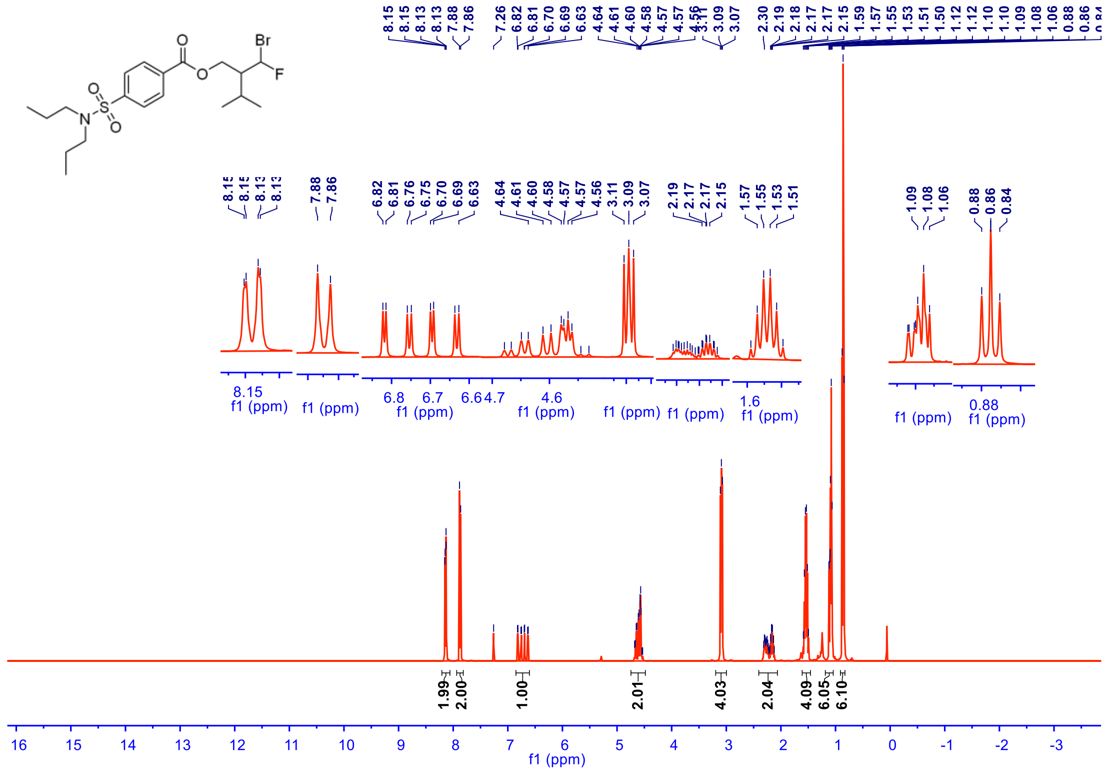

Supplementary Figure 421. ^{1}H NMR spectrum of **2-(bromofluoromethyl)-3-methylbutyl 4-(*N*,*N*-dipropylsulfamoyl)benzoate 46a** (400 MHz, $CDCl_3$)

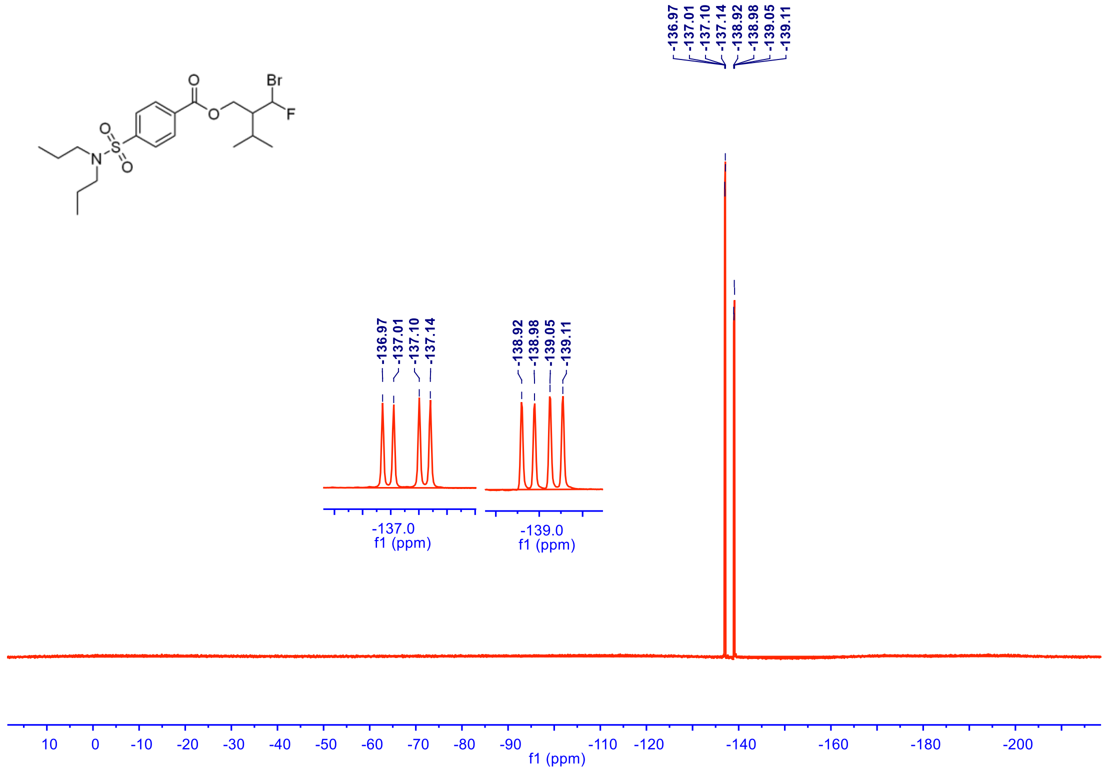

Supplementary Figure 422. ^{19}F NMR spectrum of **2-(bromofluoromethyl)-3-methylbutyl 4-(*N*,*N*-dipropylsulfamoyl)benzoate 46a** (376 MHz, $CDCl_3$)

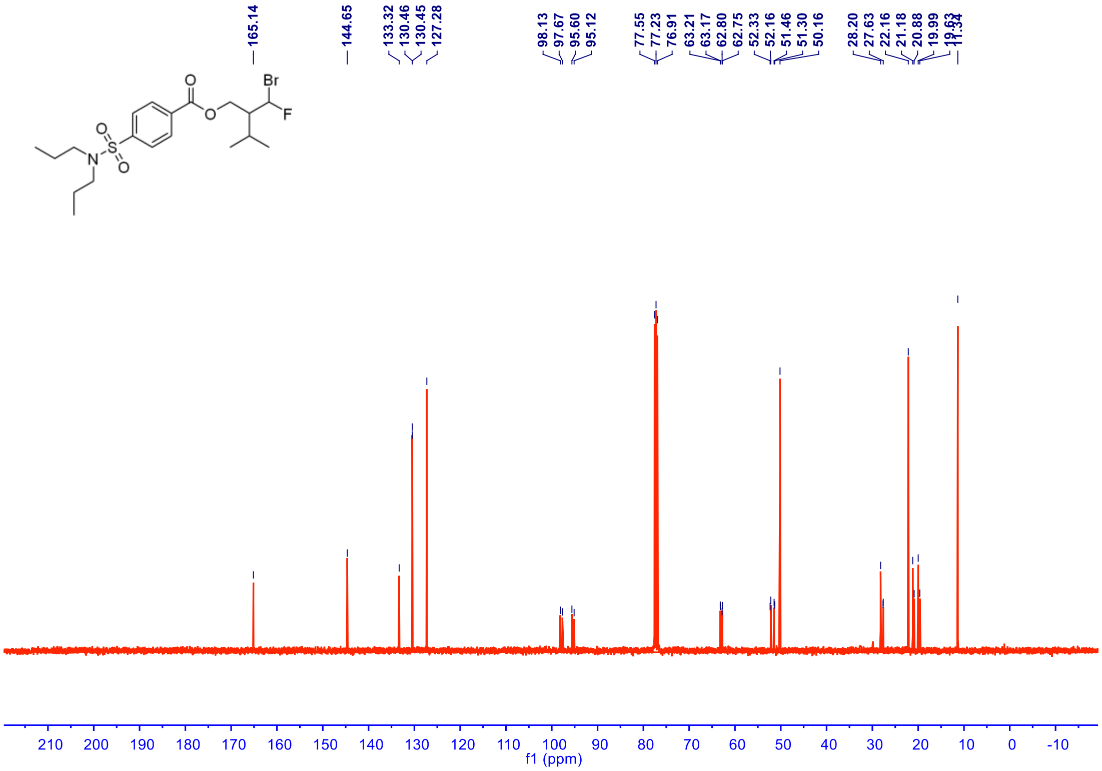

Supplementary Figure 423. ^{13}C NMR spectrum of **2-(bromofluoromethyl)-3-methylbutyl 4-(*N*,*N*-dipropylsulfamoyl)benzoate 46a** (101 MHz, $CDCl_3$)

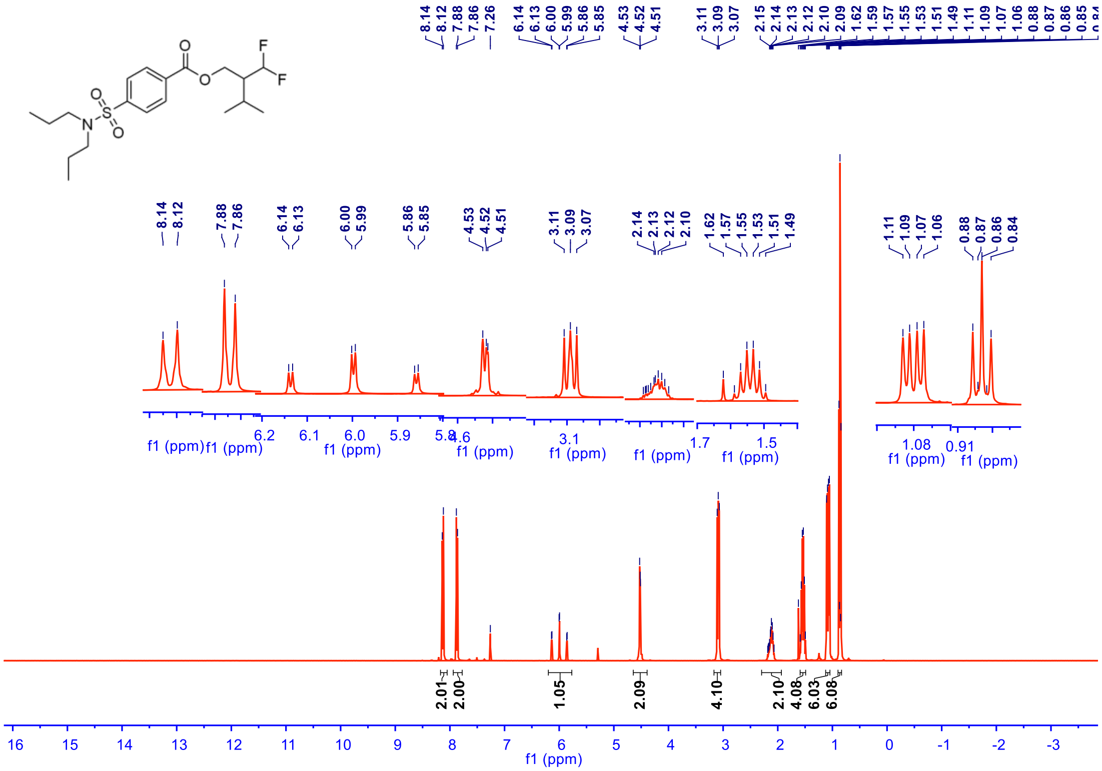

Supplementary Figure 424. ^{1}H NMR spectrum of compound **46** (400 MHz, $CDCl_3$)

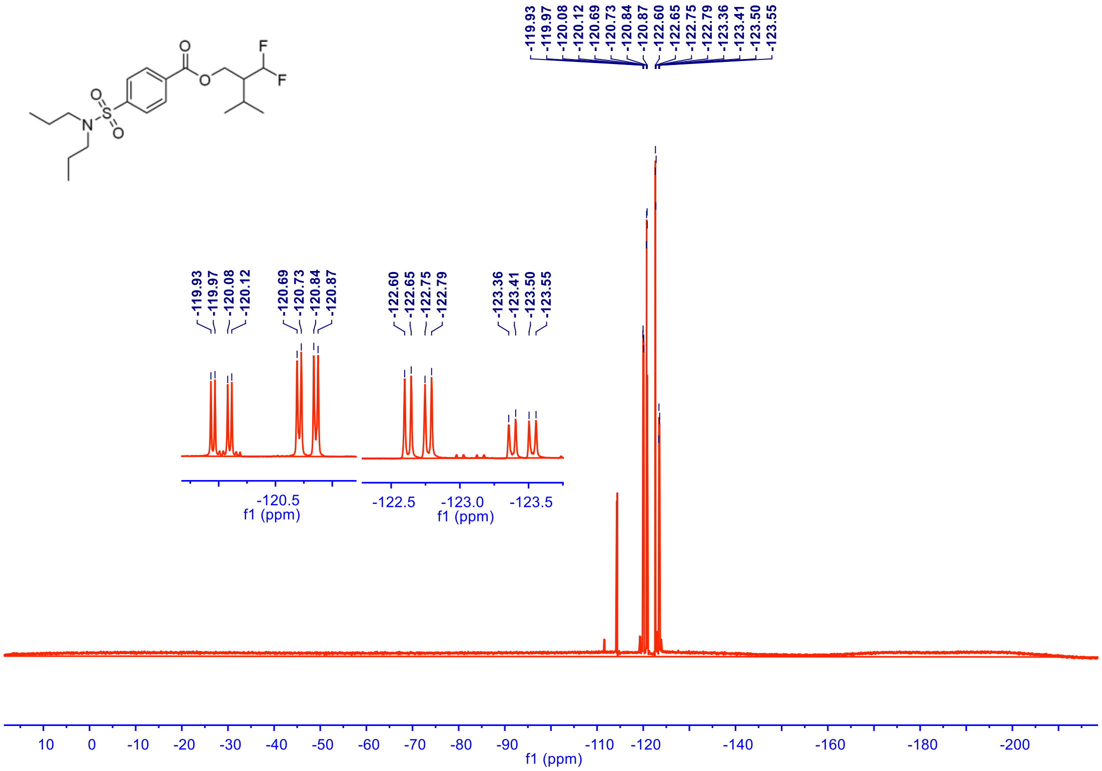

Supplementary Figure 425. ^{19}F NMR spectrum of compound **46** (376 MHz, $CDCl_3$)

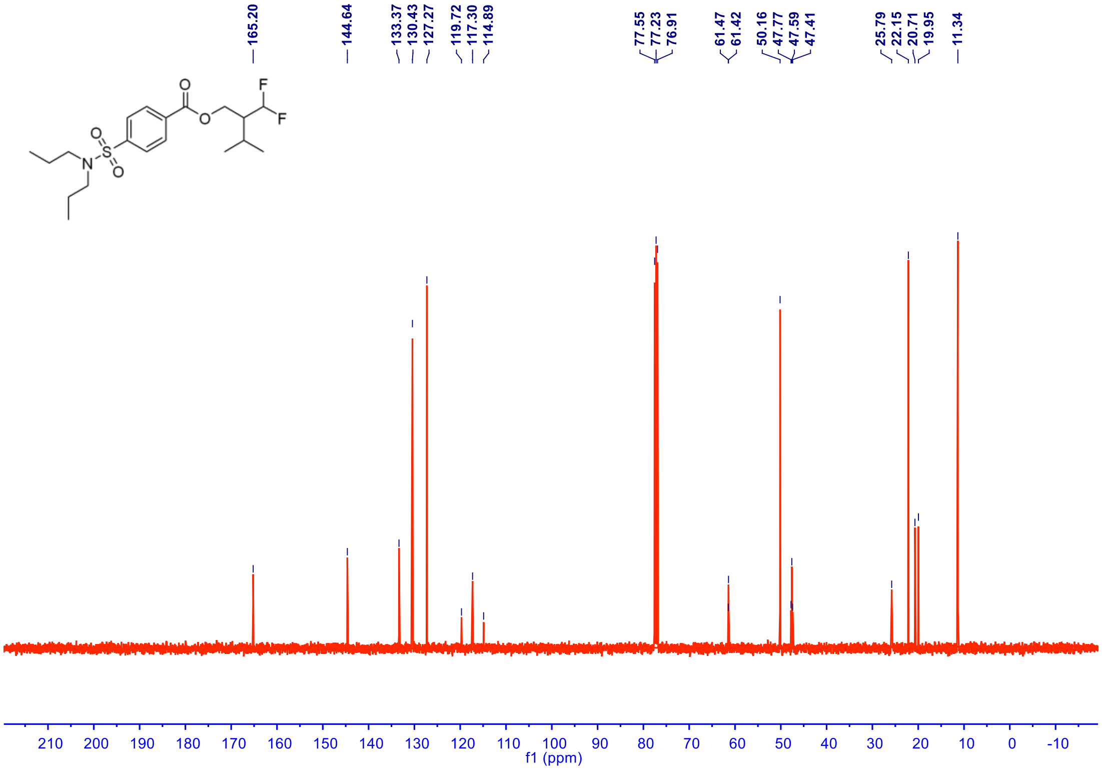

Supplementary Figure 426. ^{13}C NMR spectrum of compound **46** (101 MHz, $CDCl_3$)

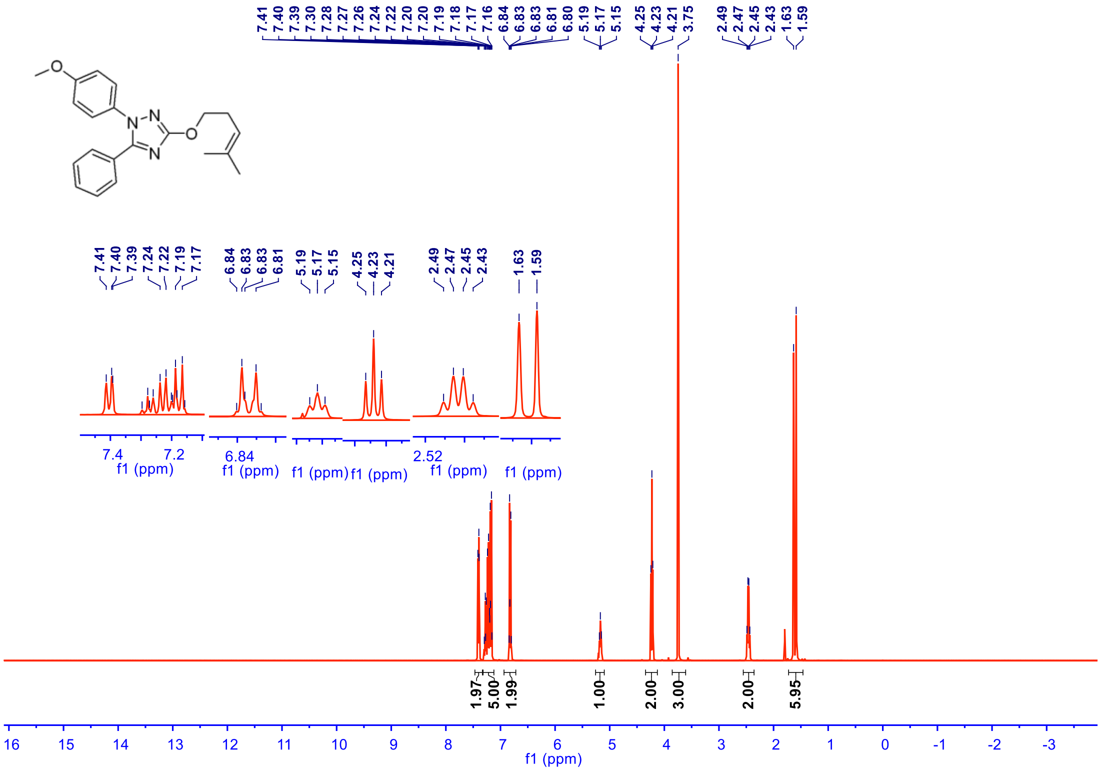

Supplementary Figure 427. ^{1}H NMR spectrum of **1-(4-methoxyphenyl)-3-((4-methylpent-3-en-1-yl)oxy)-5-phenyl-1*H*-1,2,4-triazole 47b** (400 MHz, $CDCl_3$)

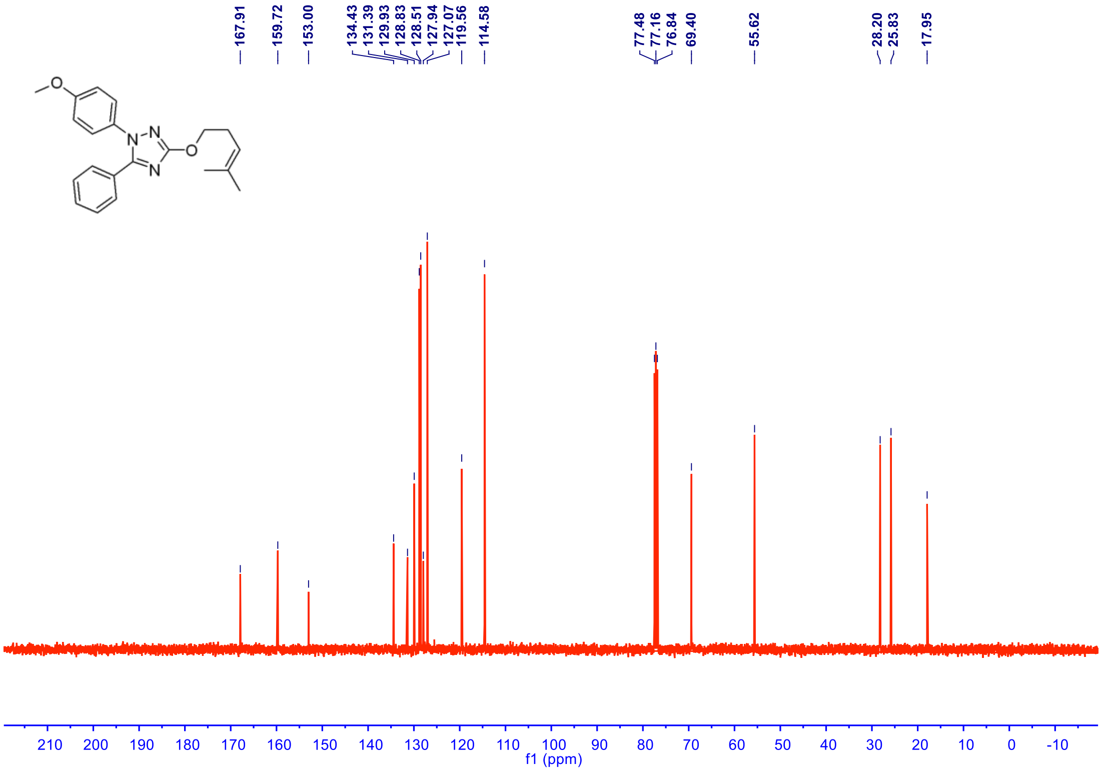

Supplementary Figure 428. ^{13}C NMR spectrum of **1-(4-methoxyphenyl)-3-((4-methylpent-3-en-1-yl)oxy)-5-phenyl-1*H*-1,2,4-triazole 47b** (101 MHz, $CDCl_3$)

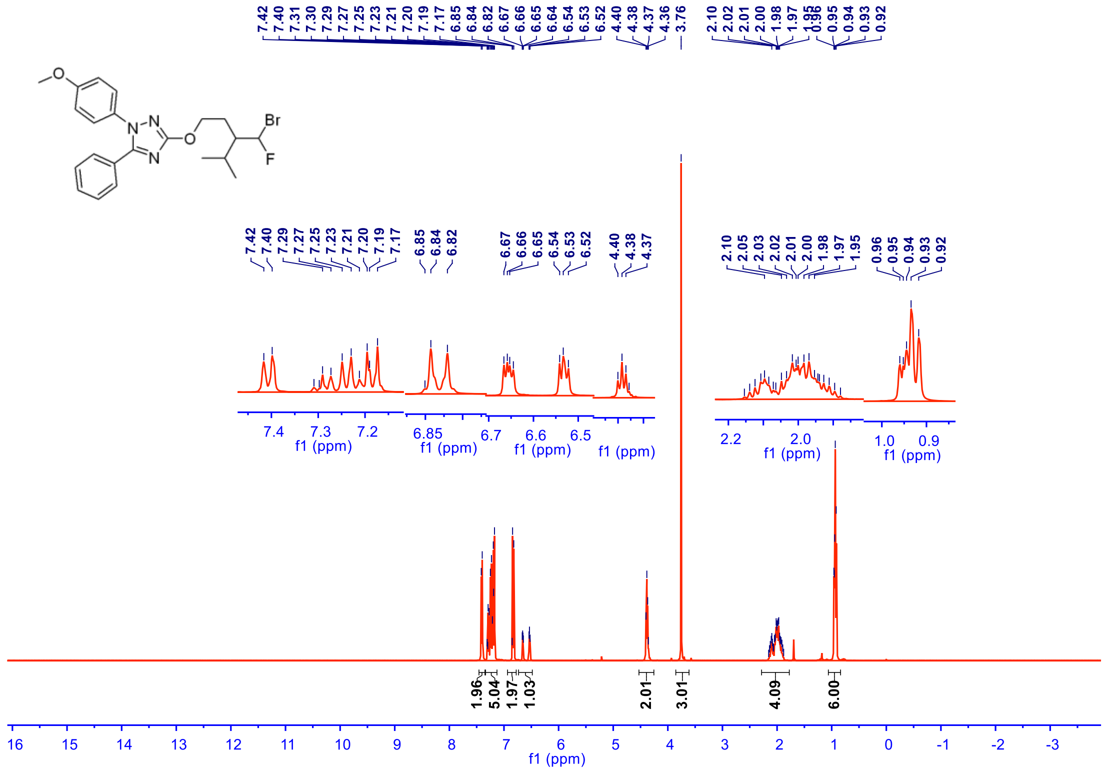

Supplementary Figure 429. ^{1}H NMR spectrum of **3-((3-(bromofluoromethyl)-4-methylpentyl)oxy)-1-(4-methoxyphenyl)-5-phenyl-1*H*-1,2,4-triazole 47a** (400 MHz, $CDCl_3$)

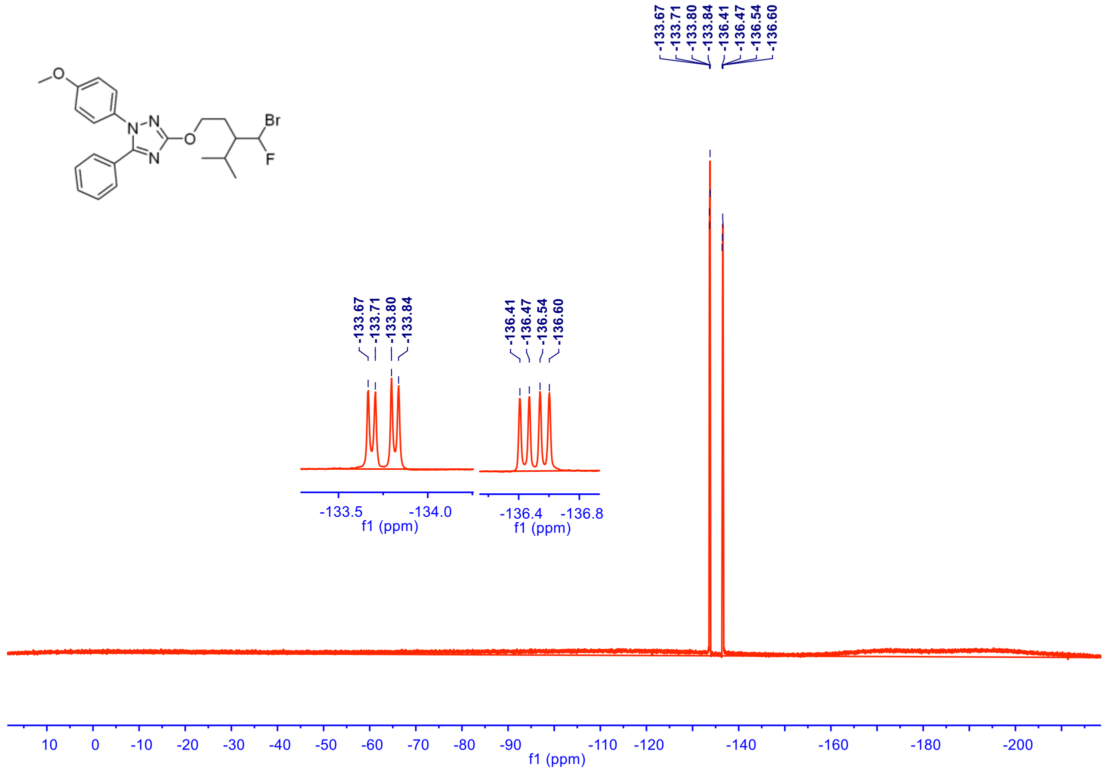

Supplementary Figure 430. ^{19}F NMR spectrum of **3-((3-(bromofluoromethyl)-4-methylpentyl)oxy)-1-(4-methoxyphenyl)-5-phenyl-1*H*-1,2,4-triazole 47a** (376 MHz, $CDCl_3$)

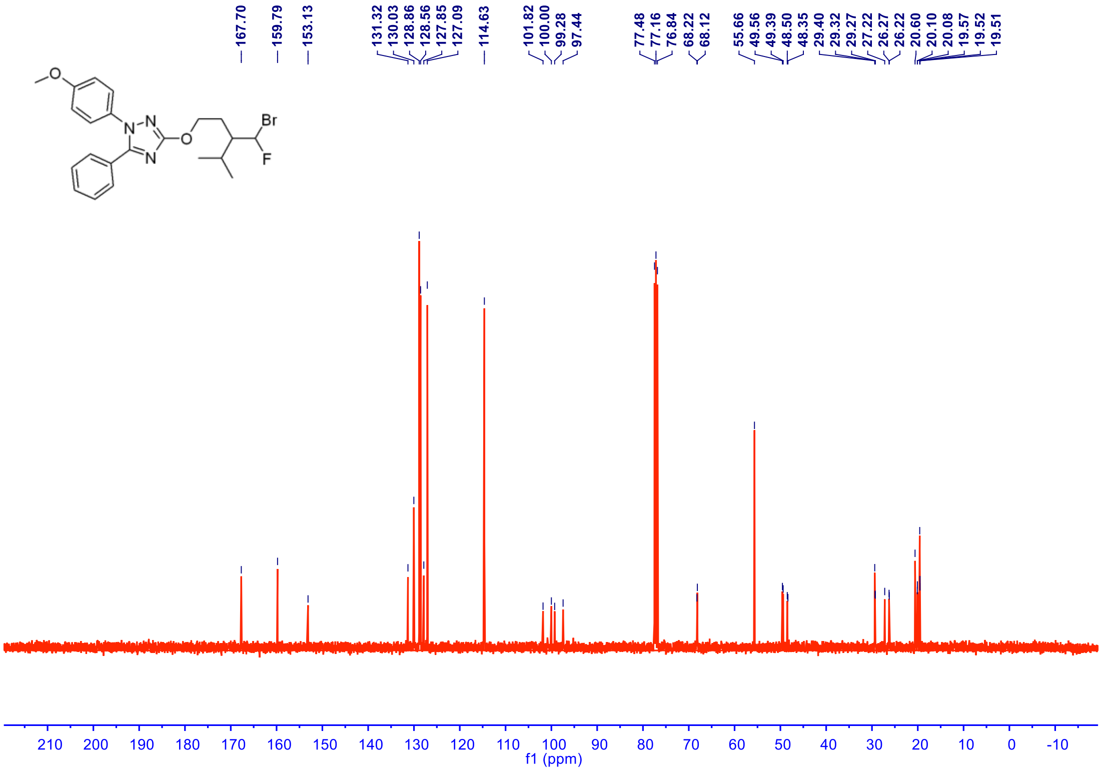

Supplementary Figure 431. ^{13}C NMR spectrum of **3-((3-(bromofluoromethyl)-4-methylpentyl)oxy)-1-(4-methoxyphenyl)-5-phenyl-1*H*-1,2,4-triazole 47a** (101 MHz, $CDCl_3$)

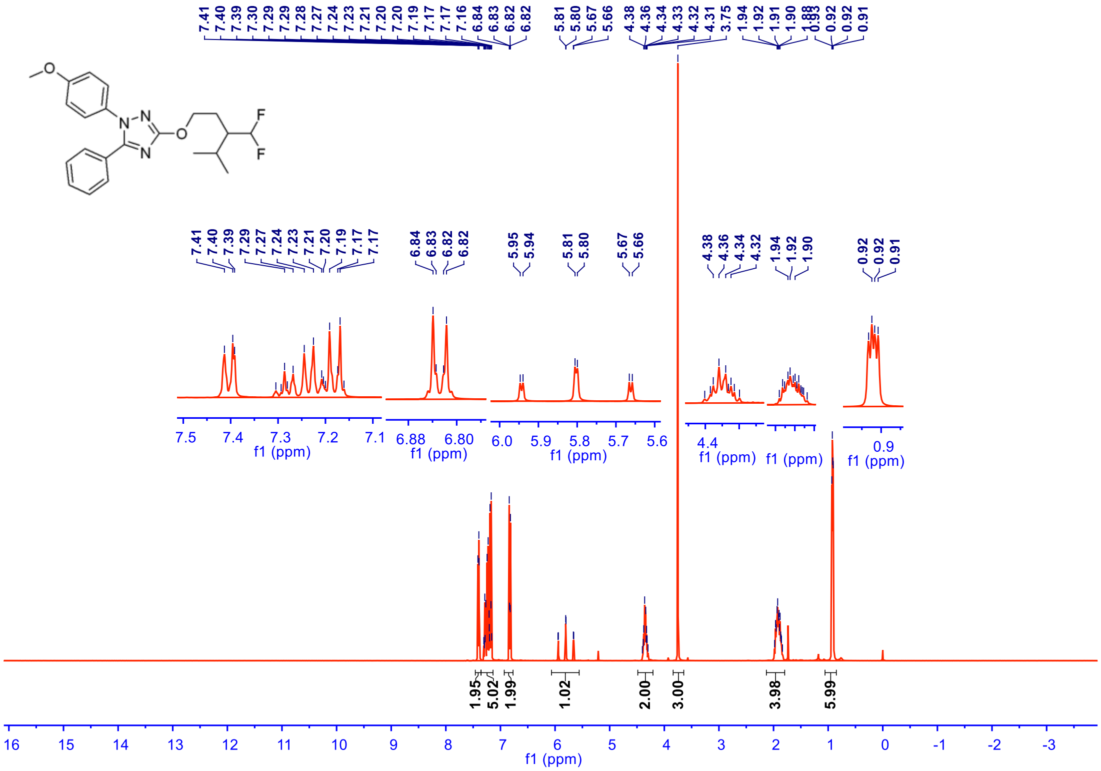

Supplementary Figure 432. ^{1}H NMR spectrum of compound **47** (400 MHz, $CDCl_3$)

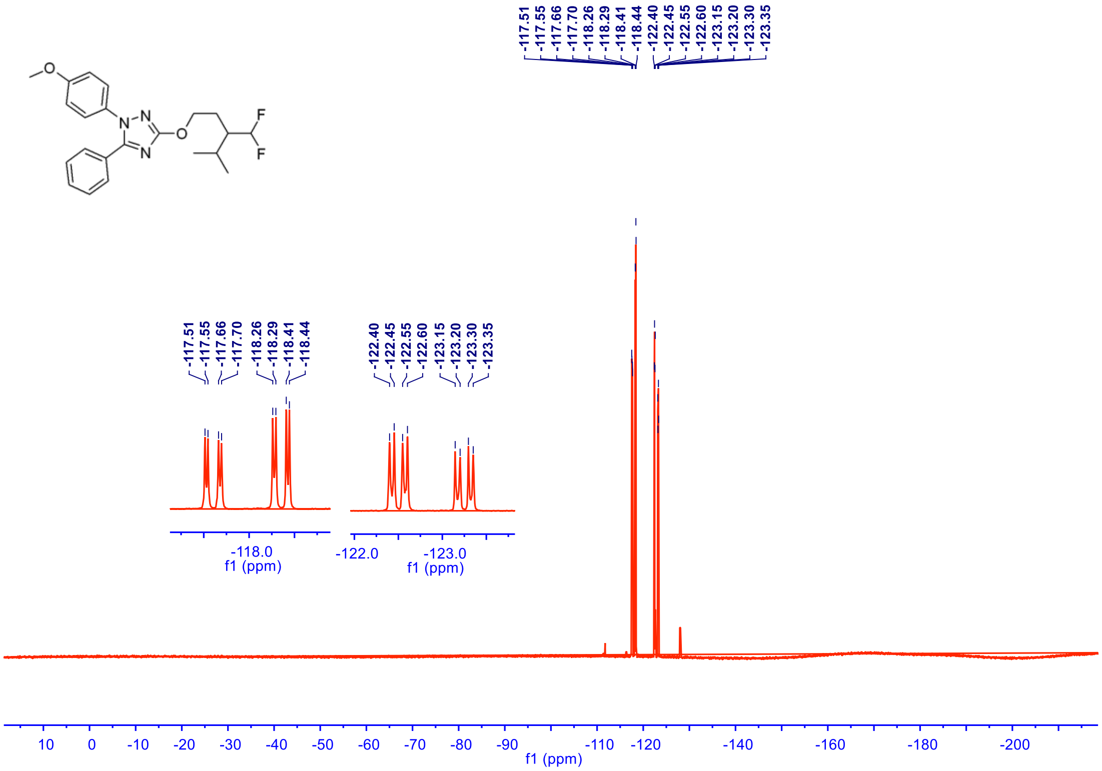

Supplementary Figure 433. ^{19}F NMR spectrum of compound **47** (376 MHz, $CDCl_3$)

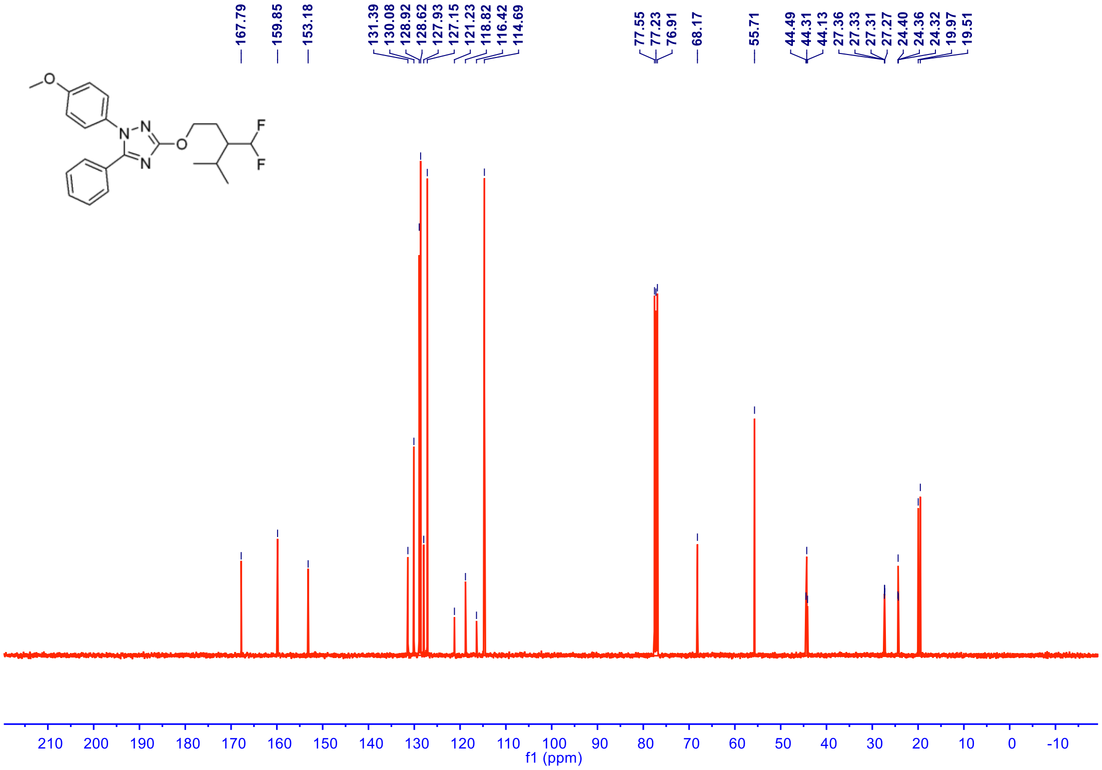

Supplementary Figure 434. ^{13}C NMR spectrum of compound **47** (101 MHz, $CDCl_3$)

6. References

1. Dolbier W. R., Okamoto M. Reactions of 1-fluoroalkyl triflates with nucleophiles and bases. *J. Fluorine Chem.* **179**, 33-41 (2015).

2. Wu B. B., *et al.* A General and efficient solution to monofluoroalkylation: Divergent synthesis of aliphatic monofluorides with modular synthetic scaffolds. *Angew. Chem. Int. Ed.* **61**, e202208938 (2022).

3. Chen F., Xu X. H., Qing F. L. Photoredox-Catalyzed Addition of Dibromofluoromethane to Alkenes: Direct Synthesis of 1-Bromo-1-fluoroalkanes. *Org. Lett.* **23**, 2364-2369 (2021).

4. Meyer C. F., Hell S. M., Misale A., Trabanco A. A., Gouverneur V. Hydrodifluoromethylation of alkenes with difluoroacetic acid. *Angew. Chem. Int. Ed.* **58**, 8829-8833 (2019).

5. Qi X. K., *et al.* Photoinduced hydrodifluoromethylation and hydromethylation of alkenes enabled by ligand-to-iron charge transfer mediated decarboxylation. *ACS Catalysis* **14**, 1300-1310 (2024).

6. Polterauer D., *et al.* Sulfur tetrafluoride (SF_4) as a deoxyfluorination reagent for organic synthesis in continuous flow mode. *Org. Proc. Res. Dev.* **27**, 2385-2392 (2023).

7. Zhang Z. Q., *et al.* Programmable synthesis of difluorinated hydrocarbons from alkenes through a photocatalytic linchpin strategy. *Chem. Sci.* **14**, 11546-11553 (2023).

8. Lin Q. Y., Xu X. H., Zhang K., Qing F. L. Visible-light-induced hydrodifluoromethylation of alkenes with a bromodifluoromethylphosphonium bromide. *Angew. Chem. Int. Ed.* **55**, 1479-1483 (2016).

9. Li W., Varenikov A., Gandelman M. Direct copper-catalyzed C-H monofluoroalkylation of benzoxazoles with 1-fluoro-1-haloalkanes. *Eur. J. Org. Chem.* **2020**, 3138-3141 (2020).

10. Liu J. D., Gong H. G., Zhu S. L. Nickel-Catalyzed, Regio- and Enantioselective Benzylic Alkenylation of Olefins with Alkenyl Bromide. *Angew. Chem. Int. Ed.* **60**, 4060-4064 (2021).

11. Zeng X. J., *et al.* Copper-catalyzed decarboxylative difluoromethylation. *J. Am. Chem. Soc.* **141**, 11398-11403 (2019).

12. Yedase G. S., Arif M., Kuniyil R., Yatham V. R. Photocatalytic hydro tri/difluoromethylation of alkenes with bench stable tri/difluoromethylating reagents. *Org. Lett.* **25**, 6200-6205 (2023).

13. Fasano V., Winter N., Noble A., Aggarwal V. K. Divergent, stereospecific mono- and difluoromethylation of boronic esters. *Angew. Chem. Int. Ed.* **59**, 8502-8506 (2020).

14. Zhang W., Lin J. H., Wu W. F., Cao Y. C., Xiao J. C. Dehydroxylative trifluoromethylthiolation, trifluoromethylation, and difluoromethylation of alcohols. *Chin. J. Chem* . **38**, 169-172 (2020).

15. Zhao H. W., Lu C. H., Herbert S., Zhang W., Shen Q. L. Difluoromethylation of alkyl bromides and iodides with $TMSCF_2H$. *J. Org. Chem.* **86**, 2854-2865 (2021).

16. Bian K. J., *et al.* Photocatalytic hydrofluoroalkylation of alkenes with carboxylic acids. *Nat. Chem.* **15**, 1683-1692 (2023).

17. Zhang Z. Q., *et al.* Difluoromethylation of unactivated alkenes using Freon-22 through tertiary amine-borane-triggered halogen atom transfer. *J. Am. Chem. Soc.* **144**, 14288-14296 (2022).
